# 3-Center-3-Electron σ-Adduct Enables Silyl Radical Transfer below the Minimum Barrier for Silyl Radical Formation

**DOI:** 10.1021/jacs.4c18445

**Published:** 2025-03-28

**Authors:** Zihang Qiu, Paolo Cleto Bruzzese, Zikuan Wang, Hao Deng, Markus Leutzsch, Christophe Farès, Sonia Chabbra, Frank Neese, Alexander Schnegg, Constanze N. Neumann

**Affiliations:** † Max-Planck-Institut für Kohlenforschung, Kaiser-Wilhelm-Platz 1, 45470, Mülheim an der Ruhr, Germany; ‡ Max-Planck-Institut für Chemische Energiekonversion, Stiftstrasse 34-36, 45470, Mülheim an der Ruhr, Germany

## Abstract

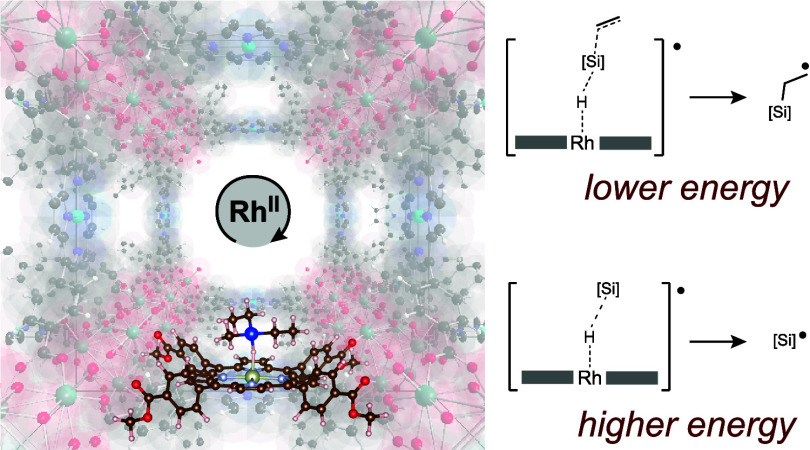

Transition-metal-catalyzed cleavage of the Si–H bond in silanes to yield silyl radicals requires substantial amounts of energy, which are commonly supplied by photons. For Rh(II) porphyrins, efficient hydrosilylation catalysis becomes accessible only upon site isolation in a metal–organic framework (MOF), and the formation of free silyl radicals likewise requires irradiation. Within the MOF, however, an uncommonly facile direct silyl radical transfer to olefin substrates is also possible, which makes thermal olefin hydrosilylation accessible at room temperature. The ability of MOF-supported Rh(II) metalloradicals to furnish an unprecedented 3-center-3-electron (3c-3e) Rh(II)-silane σ-adduct enables the assembly of a tricomponent transition state that is comprised of Rh(II), silane, and ethylene. The tricomponent transition state bypasses the high-energy silyl radical species and enables silyl radical transfer with an activation free energy ∼15 kcal·mol^–1^ below the minimum energy barrier for silyl radical formation. We report direct observation of the 3c-3e silane σ-adduct, which is a stable species in the absence of light and olefins. Furthermore, a combination of experiments and quantum chemical calculations shows that direct silyl radical transfer to ethylene is promoted by the temporary oxidation of the transition structure by a proximal Rh(II) center. Thus, the crucial role of the MOF matrix is to fix the inter-Rh separation in our catalyst at a value large enough for 3c-3e silane adduct formation but short enough for facile electron transfer.

## Introduction

**Figure 1:**
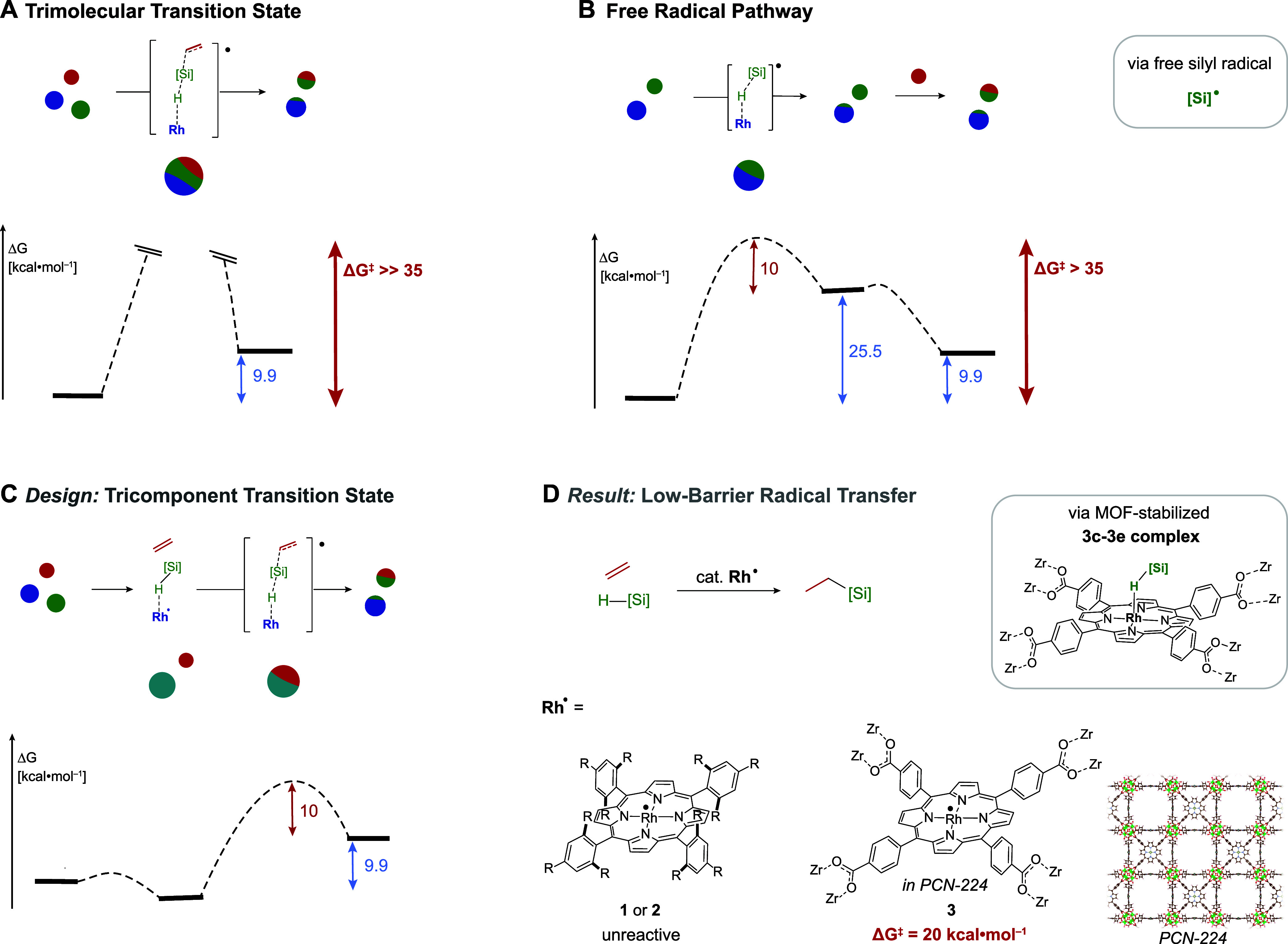
(A) Direct transfer of a silyl radical equivalent to an acceptor is associated with a prohibitively high entropic barrier, so that radical hydrosilylation instead proceeds in a stepwise manner via the intermediacy of free silyl radicals (B). If a 3c-3e σ-adduct between the catalyst and silane could be accessed, direct silyl radical transfer would be rendered entropically feasible (C). (D) A MOF-based Rh(II) catalyst that can stabilize a 3c-3e adduct is able to promote thermal hydrosilylation with a barrier of ΔG^⧧^ = 20 kcal·mol^–1^ while molecular analogues are unreactive. Spheres represent individual molecules that are present. Thin red arrows indicate the anticipated entropy contribution of 10 kcal·mol^–1^ to the minimum energy pathway; thick red arrows depict the total energy associated with the minimum energy pathway.

Photons are a necessary component of a rapidly increasing number of synthetic transformations.^1^ In the majority of cases, illumination is used to generate high-energy open-shell intermediates, which can engage in subsequent reaction steps with small kinetic barriers. The development of photocatalysts that permit the use of visible rather than UV light to cleave strong bonds vastly increased the scope and practical utility of photochemical reactions.^2^ Even visible light photons are highly energetic, however, with 450 nm photons carrying an energy of 63.6 kcal·mol^–1^. Since many synthetic reactions have low quantum yields, the high reactivity of radical intermediates comes at a substantial energy cost. Ideally, we could harness the versatile reactivity of high-energy radicals without having to generate them as free species. An energy-efficient direct transfer of an incipient radical to a radical acceptor could unlock more sustainable radical reactions. For a reaction involving a catalyst, a radical precursor, and a radical acceptor species, a direct transfer reaction would be possible if all three species could be assembled together. But triatomic collisions only take place with appreciable rates under extreme conditions, such as those present during the formation of a star.^3^ An additional challenge is that the catalyst and both reagents are not atoms but molecules of reasonable complexity. For multiatomic molecules, not only the minute collision frequency but also the need for correct alignment of all species renders it unlikely for termolecular steps to contribute noticeably to reaction rates (Figure 1A). Terrestrial chemical transformations thus generally proceed via successive uni- and bimolecular reaction steps.^4,5^ In the case of catalytic radical hydrosilylation reactions, an initial encounter between the catalyst and silane leads to the formation of a free silyl radical, which then reacts with an olefin in a separate reaction step (Figure 1B). For Rh(II) porphyrin catalysis, the difference in Gibbs free energy between the reagents (silane and Rh(II)) and the products (Rh(III)-H and silyl radical) is calculated to be 25.5 kcal·mol^–1^ (Figure S74). However, for hydrogen atom transfer (HAT) to take place, two molecules need to associate into a transition structure which results in an energy penalty due to the loss of translational and rotational degrees of freedom that corresponds to approximately 10 kcal·mol^–1^.^6−8^ A catalyst that is able to assemble a silane and an olefin into a transition state for direct radical transfer with an energy input of less than 25.5 + 10 = 35.5 kcal·mol^–1^ would thus permit a more energy-efficient transformation. Such a transition state would need to be assembled in a stepwise fashion to limit the entropic penalty. We define here the term “tricomponent” as a transition state that brings together three individual species but is not assembled via a termolecular collision and thus entropically accessible (Figure 1C). To render the formation of a tricomponent transition state feasible, the Rh(II) catalyst and the silane need to be able to form an adduct with sufficient stability that it survives under the reaction conditions for an appreciable amount of time. Here we show that a thermally stable 3c-3e Rh(II)-silane σ-adduct can be generated within a metal–organic framework (MOF) matrix. An in-depth mechanism study showed that the stability of the 3c-3e silane σ-adduct within the MOF enables MOF-supported Rh(II) porphyrins to promote thermal hydrosilylation at room temperature, while molecular Rh(II) porphyrin analogues proved ineffective in hydrosilylation, even at 140 °C (Figure 1D). Access to the 3c-3e silane σ-adduct permitted the assembly of a tricomponent transition state for direct silyl radical transfer with an activation barrier of 20 kcal·mol^–1^. An alternative pathway that proceeds via free silyl radicals is associated with a minimum energy barrier of ∼35 kcal·mol^–1^ (Figure 1B) and was realized experimentally with the assistance of 390 nm photons, which carry an energy of 73 kcal·mol^–1^.

**Figure 2:**
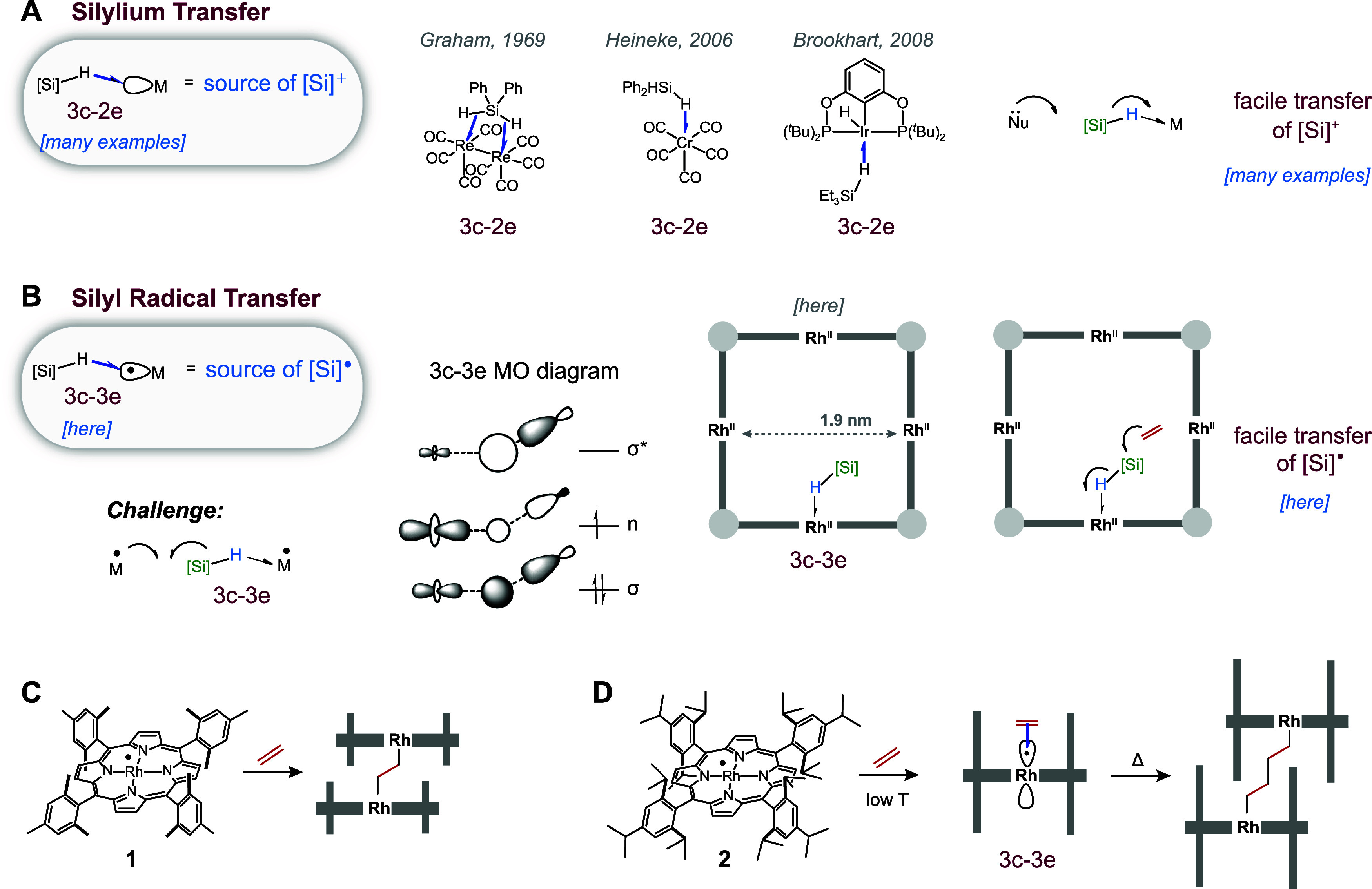
(A) 3c-2e interaction with transition metals activates silanes toward the transfer of silylium equivalents while (B) the 3c-3e interaction present in the MOF-supported Rh(II)-silane adduct activates silanes toward silyl radical transfer. In solution, 3c-3e adducts can readily react with another metal center with an unpaired spin to furnish two 2c-2e bonds in fast and favorable cleavage of the 3c-3e adduct. Moderate (C) or substantial (D) steric bulk does not prevent 2c-2e bond formation for homogeneous catalysts. The half-arrow notation introduced by Green and Parkin for 3-center bonding is used throughout this work.^27^

Prior examples in which the assembly of silanes and substrates by a transition metal center leads to transformations with a low activation barrier can be found for the formal transfer of silylium cations (Figure 2A). A mechanism put forth originally by Crabtree, and extensively refined by Brookhart and Oestreich invokes a 3-center-2-electron (3c-2e) interaction between a silane and an empty transition metal orbital as a crucial reaction intermediate.^9−12^ Binding of the silane to the metal center enables a low energy barrier transfer of a silylium equivalent to polar substrates such as ketones,^12,13^ alcohols,^14^ CO_2_,^15,16^ and even weakly nucleophilic alkyl halides and dialkylethers.^17−19^ Inspired by the role of the 3c-2e interaction in providing a low energy pathway for the formal transfer of silylium, we aimed to access a corresponding 3c-3e silane σ-adduct to facilitate silyl radical transfer (Figure 2B). While many metal complexes featuring 3c-2e silane adducts have been studied and structurally characterized,^14,20−24^ to the best of our knowledge, there has been no report of a 3c-3e silane adduct. A substantial challenge in the stabilization of 3c-3e interactions is that one of the two species participating in the interaction is a radical, which can undergo further reaction with the 3c-3e adduct to generate two strong 2c-2e bonds (Figure 2C,D). Kinetic barriers for the reactions of radicals tend to be low, so that the low prevalence of 3c-3e interactions is readily accounted for by a decomposition pathway that is thermodynamically favorable and, in the absence of steric or geometric restrictions, extremely facile. Ribas and co-workers reported a rare example of a 3c-3e adduct between a macrocycle-supported Cu(II) and a C–H bond on the ligand, which decomposes readily via a bimolecular pathway.^25^ Wayland previously demonstrated that the extreme steric bulk surrounding Rh(II) in **2** permitted EPR observation of 3c-3e π-adduct with ethylene (Figure 2D).^26^ Addition of ethylene to less sterically encumbered **1** leads to the rapid addition of the metalloradicals across the ethylene double bond, likely via the intermediacy of a 3c-3e ethylene adduct of **1** (Figure 2C). Even for bulky **2**, the 3c-3e adduct proved stable only at low temperature, and irreversible insertion of two ethylene molecules between a pair of Rh(II) porphyrin complexes was observed upon heating (Figure 2D). Prior work on Rh(II) porphyrin systems highlights four salient points: (i) spin density from the rhodium center is partially shifted to the carbon center of the ethylene 3c-3e adduct; (ii) this spin density can be used to form substrate–substrate bonds (Figure 2D) but (iii) if sterically accessible, formation of substrate-catalyst bonds is preferred (Figure 2C) and (iv) even if substrate–substrate bond formation occurs, two Rh(II) metalloradicals are converted into unreactive carbon ligated Rh(III) centers (Figure 2D). To stabilize a 3c-3e σ-adduct of a metalloradical, follow-up reactions with another metalloradical thus need to be prevented, which can readily be achieved via site-isolation in a MOF matrix (Figure 2B).

## Results and Discussion

**Figure 3:**
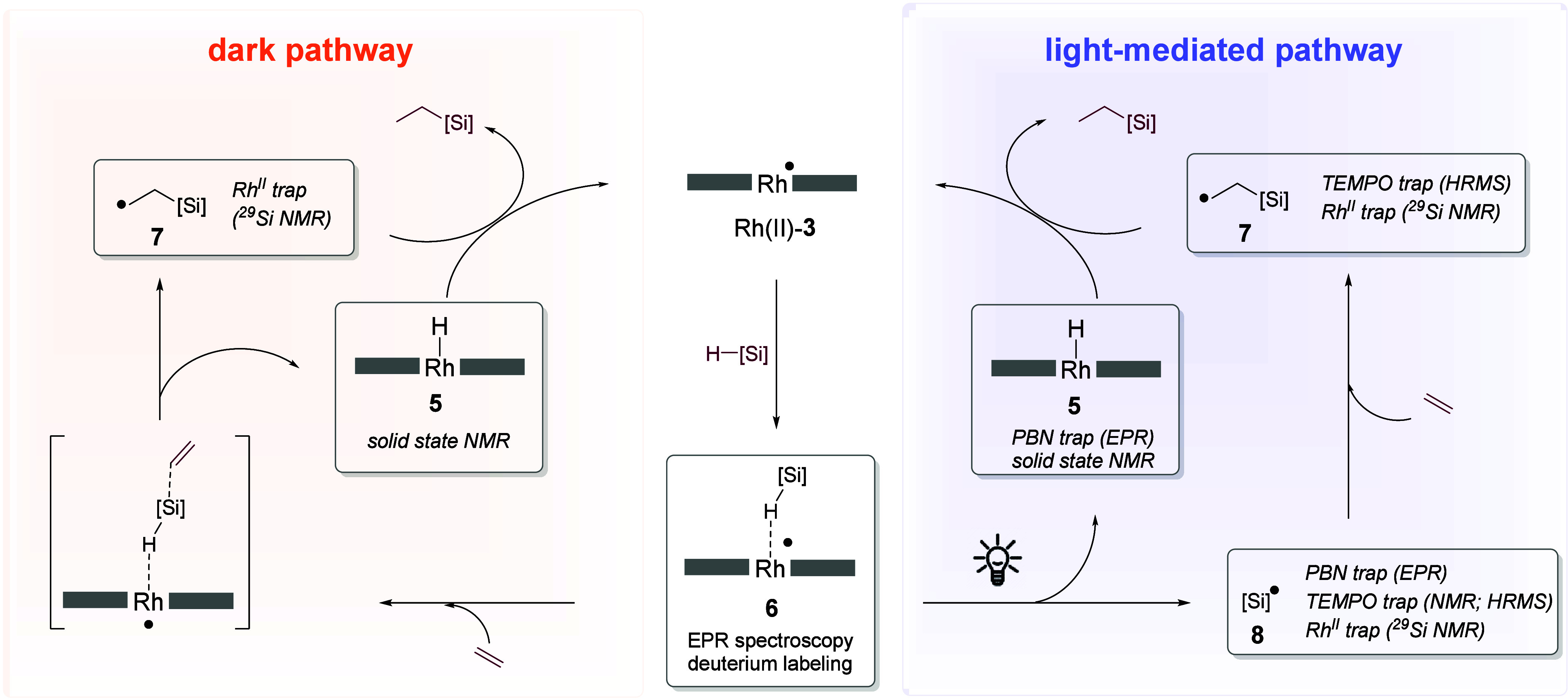
Proposed reaction mechanism that comprises distinct pathways available in the absence (left) and presence (right) of light (λ = 390 nm). Each intermediate is labeled with experimental evidence that supports its presence.

We have previously shown that Rh(II) centers site-isolated in the porphyrin-containing MOFs PCN-224 (Rh(II)-**3**) or PCN-222 (Rh(II)-**4**) are efficient hydrosilylation catalysts.^28^ Comparison with homogeneous model systems established that heterogenization substantially benefited both the catalyst’s activity and selectivity. In this work, we set out to elucidate the origin of the reactivity differences that emerge when Rh(II) porphyrin centers are placed in a MOF-environment. Prior to in-depth mechanistic studies, we excluded that hydrosilylation proceeded via a radical chain reaction initiated by Rh(II) based on the following evidence: (i) prior literature reports state that trialkylsilanes are unsuitable substrates for hydrosilylation via a radical chain mechanism,^29^ (ii) no reaction was observed when we replaced **3** with AIBN (Figures S1–S3), and (iii) an on–off experiment showed substantial differences in the reaction rate in the presence and absence of light (Figure S4). The active Rh(II) catalyst is generated by photolysis of a Rh(III)-Me precatalyst during which methyl radicals are released. We could exclude that methyl radicals contribute noticeably to the observed reactivity since no decrease in reaction rates was observed when reused catalyst (from which no methyl radicals can be released) was used to catalyze olefin hydrosilylation (Figures S5–S6). Based on detailed EPR analyses, labeling and trapping experiments, kinetic data, IR data, as well as solution and solid-state NMR experiments, which we will discuss below, we propose the catalytic cycles depicted in Figure 3, which are corroborated by quantum chemical calculations. Our proposal includes two different reaction pathways for the rate-limiting transfer of silyl radical from the silane to the olefin, of which one leads to the generation of free silyl radicals, while the second pathway achieves a direct silyl radical transfer to the olefin.

### Characterization of the Active Catalyst

While we have previously shown that photolysis of Rh(Me)-**3** gave rise to reactivity that was consistent with the formation of site-isolated Rh(II), no direct evidence for the formation of Rh(II) was provided. Here we present direct characterization of Rh(II)-**3** and Rh(II)-**4** via continuous-wave (CW) EPR spectroscopy (Figure 7A). The shape of the EPR signal arises from the coupling of the electronic spin *S* = 1/2 of Rh(II) with the nuclear spin of ^103^Rh *I* = 1/2 with *g*_⊥_ > *g*_e_ > *g*_||_ typical of a d_*z*^2^_ singly occupied molecular orbital (SOMO). Computer simulations of the spectrum show the contribution of a major species with spin Hamiltonian parameters consistent with the reported data for homogeneous porphyrin Rh(II) metalloradicals in a frozen solvent matrix (Figures S25 and S34).^30^ We also derived the average distance between Rh(II) centers in Rh(II)-**3** and Rh(II)-**4** from line shape analysis of the EPR spectra. By assuming that the broadening of EPR signals results from the interaction between adjacent Rh(II) centers and applying a simple dipolar coupling model (Figure S34 and Table S12), we obtained Rh(II)–Rh(II) intermolecular separation values of 1.81 nm in the case of Rh(II)-**3** and 1.24 nm for Rh(II)-**4**. Both values are in good agreement with the separation between porphyrin centers in the X-ray structures of PCN-224 (1.9 nm) and PCN-222 (1.06 nm for triangular pores).^31−35^ A larger discrepancy is expected for PCN-222 due to the presence of hexagonal as well as triangular pores so that the dipolar coupling observed by EPR is averaged over two sets of Rh(II) centers located at different distances (Table S12). The agreement between EPR-derived Rh(II)–Rh(II) separations and the crystallographically determined porphyrin spacing is consistent with a complete conversion of Rh(III)Me to Rh(II) sites upon irradiation. Complete conversion of Rh(III)-Me to Rh(II) was further confirmed by ^1^H NMR analysis of the metalloporphyrin linker that was recovered when Rh(II)-**3** or Rh(II)-**4** was subjected to digestion under basic conditions.^28^ We also observed the presence of methane and toluene after Rh(Me)-**3** was irradiated in benzene, which is consistent with the release of methyl radicals that undergo reaction with the benzene solvent, as previously described by Wayland and co-workers.^36^ Notably, EDX analysis of **3** recovered from catalytic reactions remained consistent with the complete metalation of all porphyrin sites by rhodium.^28^

**Figure 4:**
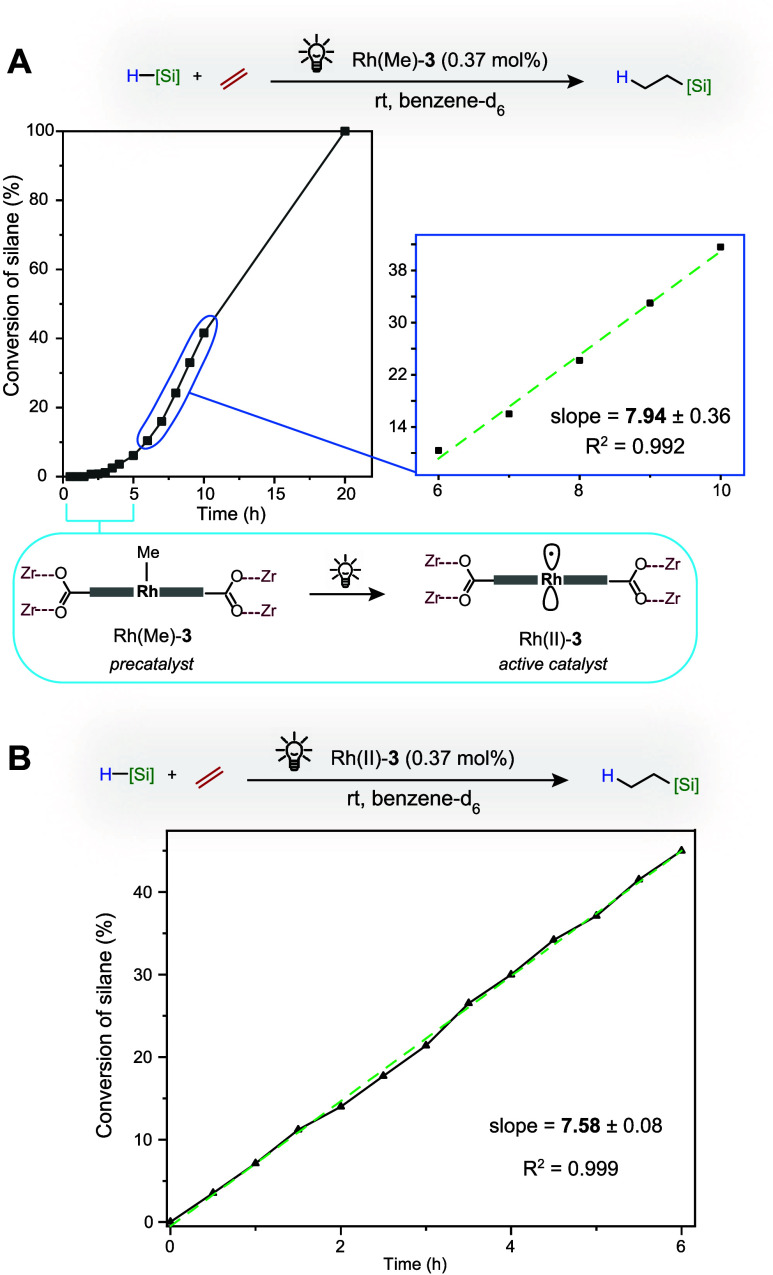
Silane conversion over time in the light-mediated hydrosilylation of ethylene with the Rh(Me)-**3** precatalyst (A) and with isolated Rh(II)-**3** (B).

To confirm that the metalloradical species, Rh(II)-**3**, observed by EPR spectroscopy constitutes a kinetically competent catalyst for olefin hydrosilylation, we monitored the rate of ethylene hydrosilylation (Figure 4). Direct use of Rh(Me)-**3** in light-promoted hydrosilylation showed an initiation period of close to 5 h, which we attribute to the conversion of the bench-stable Rh(III)-Me precursor to catalytically active Rh(II)-**3** (Figure 4A). Since site-isolation renders the Rh(II) catalyst stable to storage under inert conditions, direct use of Rh(II)-**3** in catalysis could also be tested (Figure 4B). As expected, the initiation period disappeared for Rh(II)-**3**, and the effective rate constant is identical within experimental error to that observed with Rh(Me)-**3** after reaction for 5 h (Figure 4A). Rh(II)-**3** is thus gradually formed under light-mediated reaction conditions and is a kinetically competent catalyst for olefin hydrosilylation.

### Oxygen Effect on Thermal Hydrosilylation

**Figure 5:**
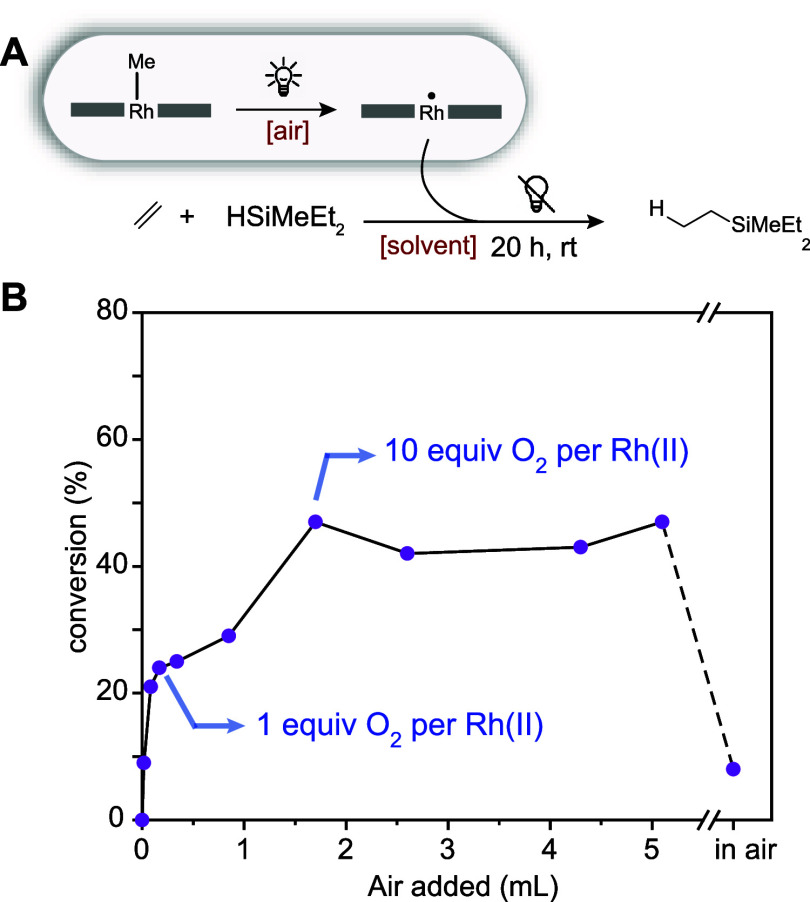
Evaluation of the effect of the presence of oxygen during the preparation of the active catalyst (A) on the performance of Rh(II)-**3** in thermal hydrosilylation (room temperature, 20 h) (B).

In order to compare the kinetic profile of the light-mediated and thermal hydrosilylation reactions catalyzed byRh(II)-**3**, we attempted numerous in situ NMR experiments under thermal conditions. Unfortunately, duplicate measurements frequently gave rise to notably different conversion rates (Figures S8–S10). During our search for the source of the rate variation, we employed increasingly rigorous means of excluding oxygen during the synthesis of active catalyst Rh(II)-**3**. When the reaction was set up in an argon-filled glovebox with freeze–pump–thaw deoxygenated benzene, we obtained Rh(II)-**3** that gave rise to only 4% conversion in hydrosilylation. Metalloradical Rh(II)-**3** is itself an effective oxygen trap, so we recycled the benzene for subsequent syntheses of the active catalyst. Rh(II)-**3** from the first recycle achieved 2% conversion and Rh(II)-**3** from the second recycle gave only 0.3% conversion, compared to ∼20% conversion for Rh(II)-**3** prepared with benzene that was only sparged with argon. To test whether the addition of small amounts of oxygen during the preparation of Rh(II)-**3** increased the catalyst’s hydrosilylation performance, we introduced controlled amounts of air (Figure 5A,B). Two plateaus were observed that corresponded to the addition of ∼1 equiv of O_2_ and more than 10 equiv of O_2_ per Rh(II) center, respectively. While addition of 10–33 equiv of O_2_ furnished Rh(II)-**3** that was able to achieve close to 50% conversion, Rh(II)-**3** that was prepared in air was substantially less active (Figure 5B). We confirmed that oxygen rather than any other components of non-dried air was responsible for the rate enhancement by repeating key experiments with dry O_2_ gas (Figure S16).

**Figure 6:**
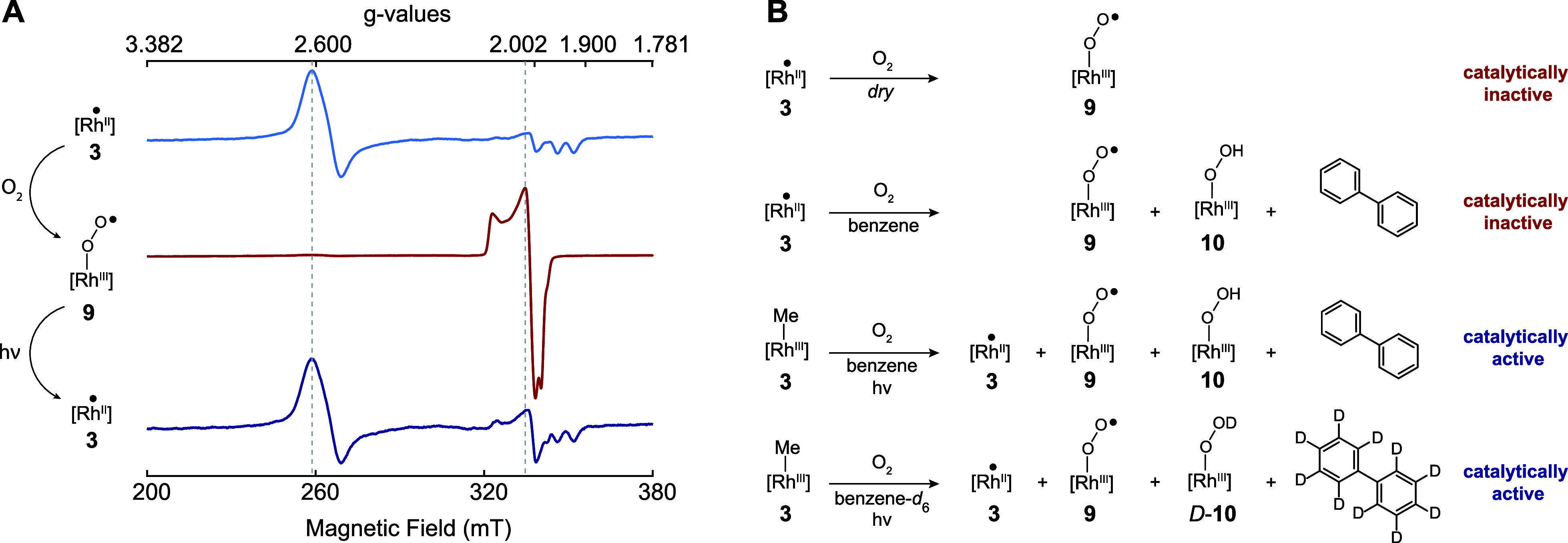
(A) In the presence of oxygen, the rapid formation of catalytically inactive rhodium superoxide **9** is observed. Formation of **9** from Rh(II)-**3** is irreversible in the dark and reversible in the presence of light. (B) The simultaneous presence of Rh(II)-**3** and **10** is required for catalytic activity. For a detailed discussion of the role and formation mechanism of **9** and **10** see section 8.4 of SI.

In a series of control experiments (Figures S13–S14), we determined that (i) while O_2_ must be present during active catalyst formation, the presence of O_2_ during the use of Rh(II)-**3** in hydrosilylation is detrimental, and that (ii) O_2_ and light must be present simultaneously. We knew from prior EPR experiments that exposure of Rh(II) to air in the absence of light furnished Rh(III)-superoxide **9**. We confirmed that superoxide formation is reversible in the presence of light and Rh(II)-**3** could be quantitatively recovered after 20 h of illumination (Figure 6A). Furthermore, we determined that Rh(III)-superoxide **9** was unable to promote olefin hydrosilylation. While Rh(III)-superoxide formation was not directly responsible for the beneficial effect of O_2_, we speculated that **9** may be converted into Rh(III)-hydroperoxide **10** in the presence of benzene (Figure 6B). HAT from the benzene solvent to **9** would give rise to **10** and phenyl radicals during the preparation of the active catalyst. In support of such a pathway, mass spectrometric analysis of the reaction solvent led to the detection of biphenyl-*h*_10_ when Rh(II)-**3** formation was carried out in benzene and biphenyl-*d*_10_ when benzene-*d*_6_ was used as a solvent (Figures S23–S24). Furthermore, FTIR analysis of Rh(II)-**3** generated in the presence of 1 mL air in benzene-*d*_6_ furnished a band at 813 cm^–1^ which is in good agreement with an O–O stretch of 823 cm^–1^ for a reported rhodium-hydroperoxide (Figure S18).^37^ The band in question was absent for Rh(II)-**3** generated in an air-free solvent, which is not active in catalysis. Exposure of dry Rh(II)-**3** crystallites to air only gave rise to a new band at 1251 cm^–1^, which is consistent with the values reported for O–O stretching vibrations of other mononuclear metal superoxide complexes.^38^ When a benzene suspension of Rh(II)-**3** was exposed to air, however, bands at 1251 cm^–1^ and 819 cm^–1^ were observed, which is in good agreement with our theoretical predictions for the O–O stretches of **9** (1280 cm^–1^) and **10** (865 cm^–1^), respectively. The structural assignment of **10** is further confirmed by the fact that the replacement of benzene with benzene-*d*_6_ gave rise to bands at 1251 cm^–1^ for **9** and 813 cm^–1^ for *D*-**10**. Additional evidence for the viability of HAT as means of generating **10** from **9** could be obtained by the immediate emergence of a band at 819 cm^–1^ when **9** was exposed to 1,4-cyclohexadiene or TEMPOH (1-hydroxy-2,2,6,6-tetramethylpiperidine; Figures S20, S21).^39,40^ We thus propose that diamagnetic Rh(III)-hydroperoxide **10** is formed alongside Rh(II)-**3** when the active catalyst was prepared in the presence of a limited amount of O_2_ (Figure 6B). The beneficial effect of converting a small number of Rh(II) centers to polar Rh(III)–O–OH groups raised the possibility that ethylene hydrosilylation may occur via a polar transition state, which is explored in detail in later sections. For consistency with our earlier work, kinetic experiments and experimental activation barriers presented in the later part of this work were determined for an oxygen content that corresponds to the first plateau in Figure 5B. EPR spectroscopic characterization of reaction intermediates presented in the main text refers to rigorously deoxygenated samples in order to minimize the signal intensity of **9**. However, all spectroscopic observations were also carried out for samples with varying oxygen content to verify that all intermediates discussed below (but no additional intermediates) were likewise generated in the presence of oxygen (see Supporting Information (SI) for a detailed discussion).

### Characterization of the Silane σ-Adduct

**Figure 7:**
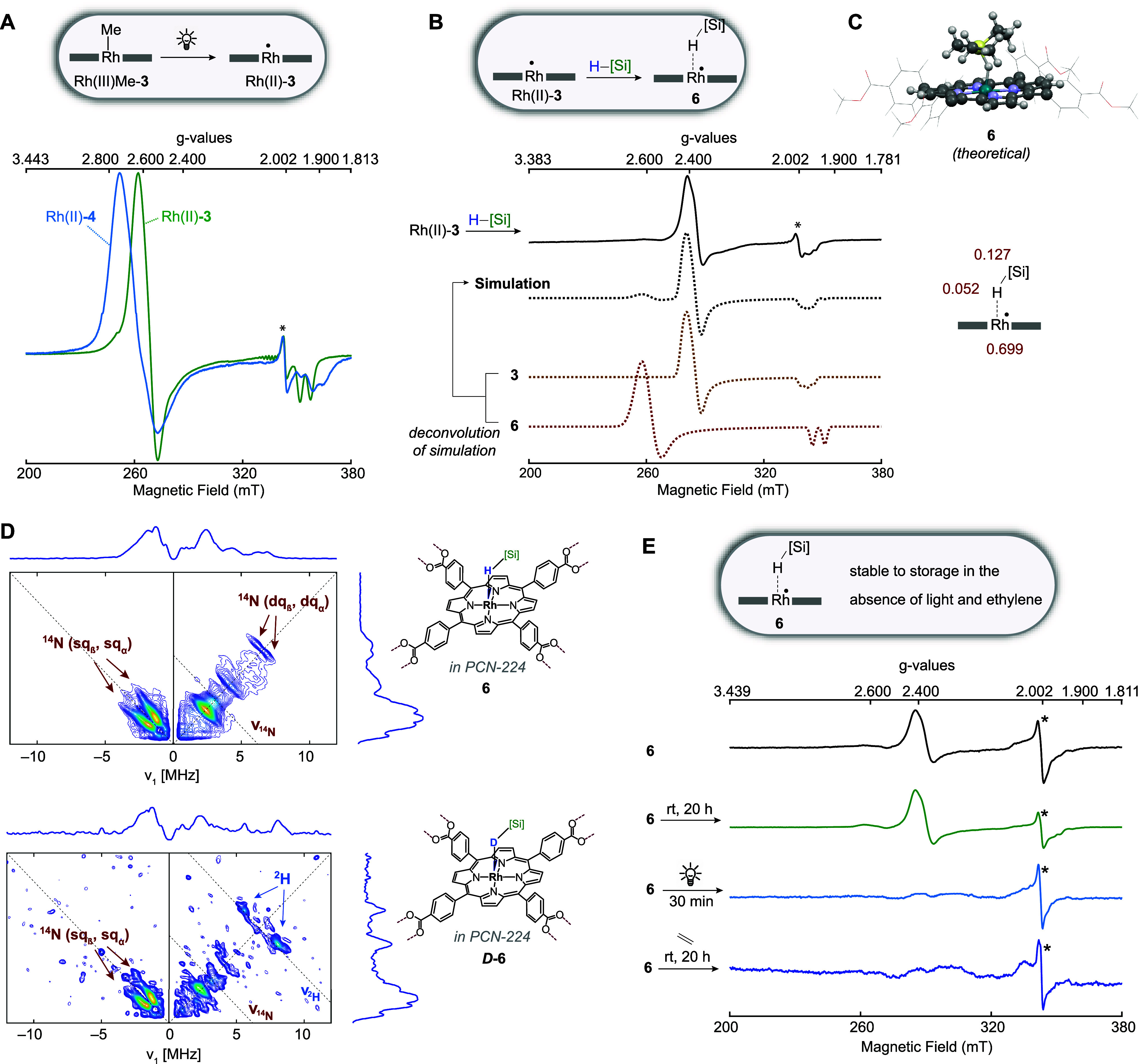
(A) X-band EPR spectra of the Rh(II) metalloradicals site isolated in PCN-224 (Rh(II)-**3**) or PCN-222 (Rh(II)-**4**) measured at 77 K. (B) Experimental and simulated X-band EPR spectrum of Rh(II)-**3** after exposure to silane measured at 77 K (calculated structure of **6** that was used in the simulation along with calculated spin populations depicted on the right). (C) Theoretical calculated structure of **6** obtained with the ORCA program package^44^ at the BP86-D3/SARC-ZORA-TZVP(Rh)/ZORA-def2-TZVP(Si)/ZORA-def2-SVP(C,H,O,N) level. (D) Q-band HYSCORE spectra recorded at 8 K at the magnetic field corresponding to *g*_*x*_ position of the HSiEt_3_ (top) and the DSiEt_3_ (bottom) σ-adducts of Rh(II) site-isolated in PCN-224. (E) EPR spectra of samples of **6** that were either stored in the dark for 20 h, exposed to light for 30 min, or exposed to ethylene in the dark for 20 h. Features marked with an asterisk were assigned to porphyrin-centered radical centers present within the metal-free PCN-224 seeds used in the MOF synthesis in accordance with previous literature reports.^45,46^

After ensuring that Rh(II) metalloradicals, the catalytically active centers in hydrosilylation, can be quantitatively generated throughout the MOF framework, we studied the interaction between the Rh(II) center with reagents: Addition of diethylmethylsilane to a suspension of Rh(II)-**3** gave rise to an immediate and drastic change in the appearance of the EPR spectrum, which was consistent with the formation of 3c-3e silane σ-adduct **6**. The EPR signal of **6** is associated with substantially different *g*-values compared to Rh(II)-**3**, which can be rationalized based on the bonding interactions predicted by first principle quantum chemical calculations of **6**. The increase in the electron density at the Rh center brought about by σ-donation from the silane to the Rh center lifts the energy of the d_*z*^2^_ orbital and thus increases the energy gap between the d_*z*^2^_ and d_*xz*_/ d_*yz*_ orbitals. Since  (where λ is the spin–orbit coupling constant and Δ*E*_*z*^2^←*xz,yz*_ is the energy gap between the d_*z*^2^_ and d_*xz*_/ d_*yz*_ orbitals) a lower *g*_⊥_ value is expected for **6** than for Rh(II)-**3**.^41,42^ Theoretical calculations located an energy minimum for an η^1^ σ-adduct that features a 146° Rh–H–Si angle (Figure 7C). The calculated Gibbs energy of Δ*G*_calc_ = −3.1 kcal·mol^–1^ obtained for σ-adduct **6**, and the predicted EPR parameters are in good agreement with experimental observation (Figure 7B, Table S7). The Si–H bond is only slightly elongated in **6** (1.56 Å) compared to the Si–H bond in the free silane (1.51 Å), and back-donation from the Rh(II) SOMO to the Si–H σ* orbital makes only a minimal contribution to the Rh-silane interaction in **6** (Table S16). Theoretical calculations also predicted a maximum hyperfine splitting of about 48 MHz due to the H atom that bridges the Rh and Si centers. Unfortunately, the spectral features expected due to the coupling to ^1^H were not fully resolved in the spectrum and generated only a broadening of the EPR signal around *g* = 1.980.

To verify experimentally that the 3c-3e silane σ-adduct adopts an η^1^ coordination mode, **6** and its deuterated variant D-**6** (Figure 7D) were interrogated by hyperfine sublevel correlation spectroscopy (HYSCORE).^43^ The predicted ^1^H hyperfine coupling for the H atom bridging Si and Rh atoms was outside the detection range of HYSCORE experiments at the Q-band frequency. However, the gyromagnetic ratio of deuterium, which is about 7 times smaller than that of ^1^H, rendered the ^2^H hyperfine interaction measurable. The Q-band HYSCORE spectra recorded at a magnetic field corresponding to the *g*_*x*_ of both **6** and *D*-**6** exhibit a complex pattern at low frequency in both the (−,+) and (+,+) quadrants due to the interaction of the unpaired electron with ^14^N nuclei (*I* = 1) from the porphyrin ring. The correlation peaks are further split into multiplets due to the quadrupole interaction. Therefore, single-quantum and nominally forbidden double-quantum transitions are observed in the spectra (Figures S32–S33). While the HYSCORE spectrum of **6** was characterized only by the presence of ^14^N signals, the spectrum of D-**6** also shows cross-peaks centered at the ^2^H Larmor frequency (ν_L_ = 6.7 MHz) separated by approximately 7 MHz. Measured ^2^H hyperfine coupling constants are in good agreement with the values calculated (Table S11) for the structural model proposed for **6** (Figure 7C).

EPR spectroscopy could also furnish direct evidence for the light-mediated cleavage of silane σ-adduct **6**. While **6** was still observed when a sample of it was stored at room temperature for 20 h, subjecting an identical sample to irradiation (390 nm) for 30 min led to the disappearance of the signal attributable to **6** (Figure 7E). Crucially, no Rh(II) signal was observed after irradiation, which is consistent with the silane σ-adduct undergoing HAT, but not with the dissociation of **6** into Rh(II) and silane. Theoretical calculations support that HAT from the silane to Rh(II) in the absence of an additional interaction with ethylene is thermally inaccessible at room temperature (Δ*G*^⧧^_calc_ = 39.1 kcal·mol^–1^), but silyl radical release from **6** becomes thermodynamically feasible in the presence of light (the energy of a 390 nm photon corresponds to 73.3 kcal·mol^–1^; see Figures S68 and S74 for a detailed discussion). In particular, many of the bright states of **6** were predicted to have significant charge transfer character from the SOMO, which is localized on the Rh–H–Si 3c-3e bond, to the porphyrin ligand (Figures S68–S69). The SOMO has bonding character for Si–H and antibonding character for Rh–H, so that excitation of one electron from the SOMO weakens the Si–H bond but strengthens the Rh–H bond. Light excitation thus facilitates Si–H bond cleavage, despite the fact that the Si–H bond (BDFE = 28.7 kcal·mol^–1^) is substantially stronger than the Rh–H bond (BDFE = 3.1 kcal·mol^–1^) in the ground state.

Notably, however, EPR spectroscopy also provided evidence for the presence of an additional thermally accessible pathway: the addition of ethylene led to the disappearance of 3c-3e silane adduct **6** within 20 h at room temperature under dark conditions (Figure 7E). The absence of signals attributable to Rh(II)-**3** or its ethylene complex supported the proposal that ethylene addition led to hydrosilylation along with formation of diamagnetic Rh(III)–H. To understand the interactions of Rh(II)-**3** with ethylene and silanes in more detail, we introduced ethylene to a benzene suspension of Rh(II)-**3**, which resulted in the formation of 3c-3e π-adduct **11**. Our assignment was based on the excellent agreement of the spectral features of **11** with those reported by Wayland and co-workers for the ethylene 3c-3e π-adduct generated from sterically shielded Rh(II) porphyrin **2**.^41^

**Figure 8:**
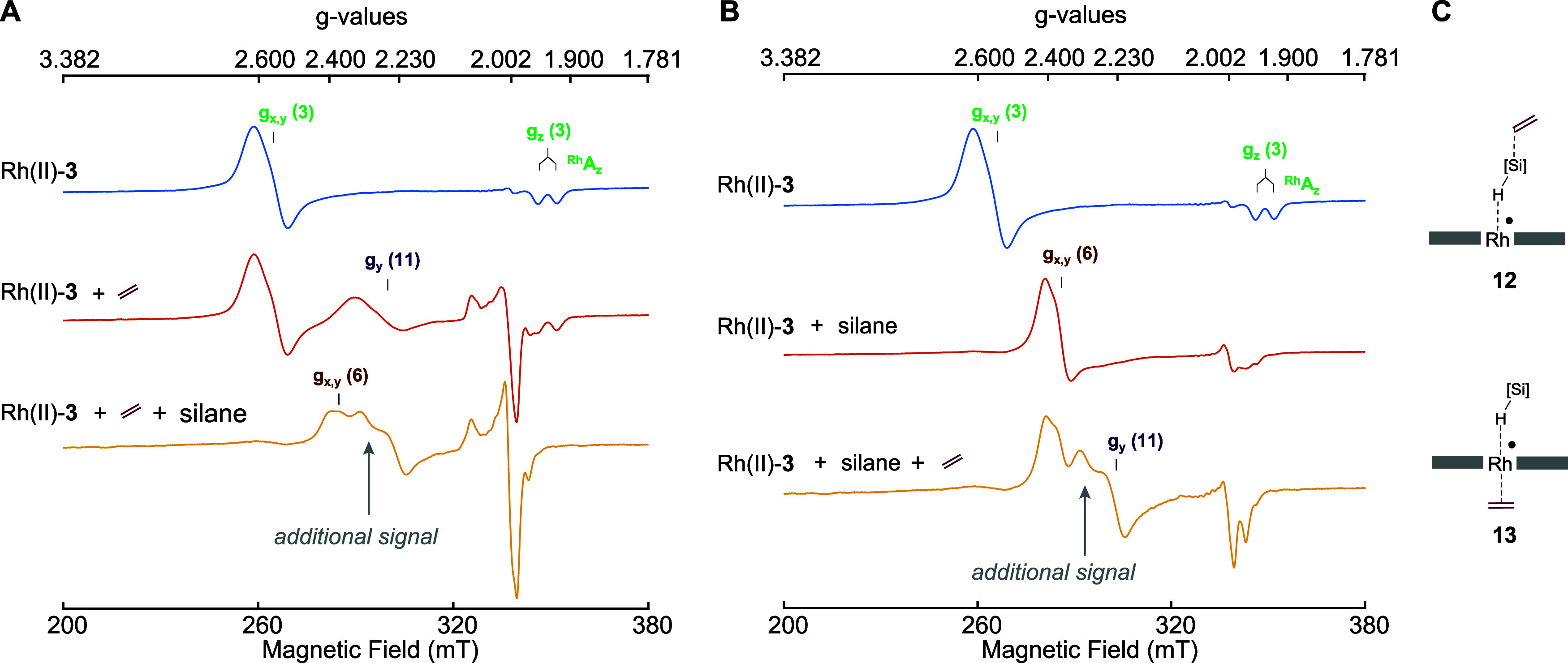
X-band EPR spectra measured at 93 K of Rh(II)-**3** in benzene to which (A) first ethylene and then silane was added and (B) first silane and then ethylene was added. (C) Proposed structures for the intermediate that gave rise to a signal at *g*_*x*_ = 2.322(5), which is indicated in (A) and (B) with gray arrows.

Next, we exposed active catalyst Rh(II)-**3** dispersed in benzene to both silane and ethylene in the absence of light and immediately froze the EPR sample in liquid nitrogen. The resulting sample contained a new species associated with *g*_*x*_ = 2.322(5), *g*_*y*_ = 2.233(5) and *g*_*z*_ = 1.984(3), in addition to ethylene adduct **11** and silane adduct **6**. We subsequently confirmed that the new signal was detected, irrespective of the order in which ethylene and silane were added to Rh(II)-**3** (Figure 8A,B). Having ruled out addition complexes of oxygen, water, or benzene solvent (Table S9), we considered that the signal may originate from van der Waals adduct **12** or bis-axial adduct **13** (Figure 8C). Theoretical calculations estimated a value of 2–4 MHz for the coupling between the unpaired spin and the ethylene protons in **13** (Table S8). However, the low signal-to-noise ratio of the echo-detected signal at the *g* = 2.322 position prevented experimental verification of the presence of weak coupling interactions. Theoretical calculations predicted, however, that the association of ethylene in adduct **12** is only fleeting (Figure S63), so that spectroscopic observation of **12** was deemed unlikely. We thus tentatively attribute the signal with *g*_*x*_ = 2.322, which was observed only when both ethylene and silane were present, to the formation of **13** (Table S8). Since we had found that the addition of limited amounts of O_2_ during the formation of Rh(II)-**3** led to an increased activity of the active catalyst during hydrosilylation, we tested whether the increased catalytic activity was associated with the formation of any new radical intermediates. However, no new species were observed by EPR spectroscopy when Rh(II)-**3** was generated in the presence of ∼10 instead of ∼1 equiv of O_2_, either for the active catalyst itself or in the presence of added reagents (Figures S30–S31). The intensities of the signals corresponding to Rh(II)-**3**, **6**, and **13** decreased with an increase in the amount of O_2_ present during Rh(Me)-**3** photolysis, however, due to competing formation of Rh(III)-superoxide **9**. For a sample of Rh(Me)-**3** that was subjected to photolysis under air, all Rh(II)-derived EPR signals were barely detectable, and only 6% conversion was achieved in thermal ethylene hydrosilylation (Figures 5B and S31). The sharp decrease in catalyst activity that was observed when the concentration of Rh(II)-**3** in the MOF was drastically reduced is consistent with the Rh(II) centers directly participating in the assembly of the transition state structure.

### Detection of Radical Intermediates

**Figure 9:**
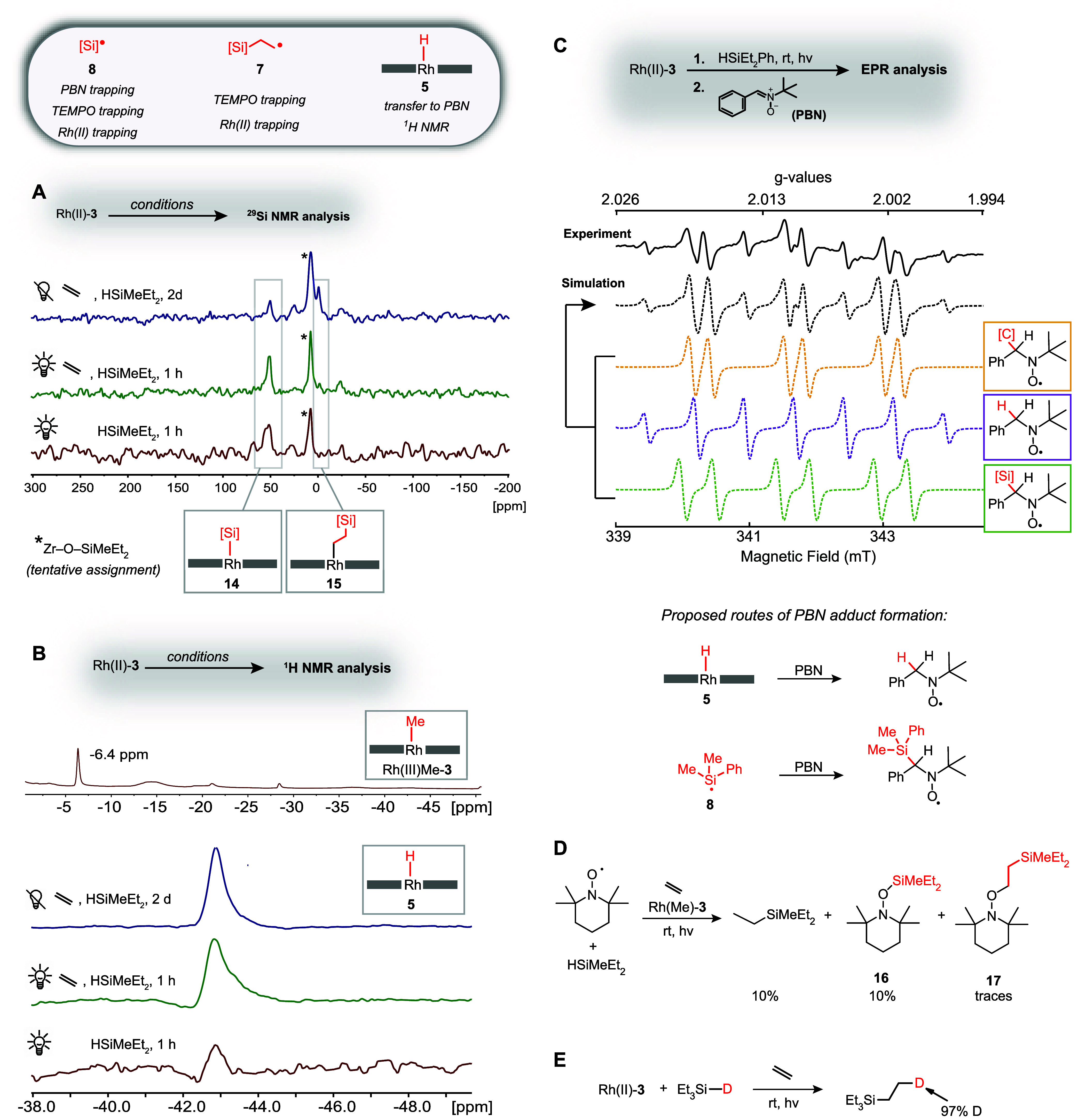
Experimentally observed reaction intermediates. (A) ^29^Si {^1^H} CP MAS NMR spectra of **14** and **15** generated as off-cycle intermediates via radical–radical recombination of silicon- and carbon-centered radicals with Rh(II)-**3**. (B) Solid state ^1^H NMR spectra of the Rh(III)Me-**3** catalyst precursor and **5** generated by exposure of Rh(II)-**3** to silane (and ethylene) in the presence or absence of light. (C) EPR data obtained when the Rh(II)-**3**-catalyzed hydrosilylation reaction was carried out in the presence of PBN along with the proposed route of formation for the PBN adducts that were observed by EPR. (D) Addition of TEMPO to hydrosilylation catalyzed by Rh(II)-**3**.^28^ (E) Deuterium originally attached to silane reagent is efficiently incorporated into the hydrosilylation product.

After analysis of the interaction of reagents with the Rh(II) center, we turned our attention to the detection of the proposed reaction intermediates. Since σ-adduct **6** is a stable species in the absence of light and ethylene, silyl radical **8** was only an expected intermediate for the light-mediated hydrosilylation mechanism, while the formation of carbon-centered radical **7** was expected for both the dark and the light-mediated reaction pathway (Figure 3). Indeed, addition of 1 equiv of TEMPO to the light-mediated hydrosilylation reaction permitted the trapping of both suspected key intermediates, **7** and **8**, as **16** and **17** (Figures 9D and S35–S36).^28^ TEMPO trapping experiments rely on the capture of fleeting radicals by a persistent radical. A persistent radical that is always present under the reaction conditions is Rh(II)-**3**, so we wondered if Rh(II) could be used directly as a radical trap, which would minimize the perturbation of the system.

A particular concern was that TEMPO may not only be able to capture free silyl radical **8**, but potentially also abstract a silyl radical equivalent from **6** (Figures S39–S42). Reliance on Rh(II) as a radical trap has the advantage that Rh(II) centers remain immobilized within the MOF framework and are unable to approach **6**, while free silyl radical **8** could react with Rh(II) centers. Only a very minor portion of the organic radicals could potentially undergo radical–radical recombination with Rh(II) (an irreversible process under thermal conditions) because efficient thermal turnover of the Rh(II) catalyst was observed. To enable a sufficient amount of radical trapping products to accumulate to permit detection, we thus carried out Rh(II)-catalyzed thermal hydrosilylation for 2 days before the MOF was recovered and analyzed by solid-state ^29^Si NMR spectroscopy. A signal for **15**, the product expected for the trapping of **7** by Rh(II), and to a lesser extent also **14**, the trapping product of **8** by Rh(II), could be observed (Figure 9A). An approximate quantification is possible via comparison of the intensity of the signals of **15** and **14** with the signal at 7.7 ppm (marked with an asterisk in Figure 9A), which we attribute to MOF node-bound silanol species. Silanol was formed as a minor (∼0.5%) byproduct in all hydrosilylation reactions. The exact amount of silanol observed depends on the level of water and oxygen contamination of the hydrosilylation reaction (Figures S43–S44). The chemical shift observed for the additional peak in Figure 9A is consistent with both Zr–O–SiMeEt_2_ and Et_2_MeSi–O–SiMeEt_2_, but because the species in question was not removed by washing the MOF prior to NMR analysis, we tentatively assign the peak to silanol interacting with the MOF nodes of **3**. Since a silanol-derived signal constituted the most intense feature in the solid-state ^29^Si NMR spectra of recovered catalysts, only a minute fraction of the silicon-centered and carbon-centered radicals were trapped by Rh(II) centers in the MOF.

Because the detection of Rh–[Si] **14** alongside Rh–CH_2_–CH_2_–[Si] **15** in the catalyst retrieved from thermal hydrosilylation was surprising, further comparative experiments were carried out to evaluate a potential contribution of silyl radicals to thermal hydrosilylation. Addition of **8** to Rh(II) could be discerned by solid-state ^29^Si NMR analysis of recovered **3** from light-mediated hydrosilylation (**14** in Figure 9A). When Rh(III)Me-**3** was irradiated in the presence of only silane, Rh–[Si] **14** could again be detected (Figure 9A), which substantiates that light-mediated silyl radical release from **6** was possible even in the absence of olefin. For both experiments under light-mediated reaction conditions, a reaction time of 1 h led to the accumulation of a larger amount of Rh–[Si] **14** than was observed in thermal hydrosilylation after 2 d, even though the formation of the Rh–[Si] bond in **14** is reversible in the presence of light. Solid-state NMR data thus indicated that the concentration of silyl radicals is substantially smaller under thermal conditions compared to photochemical conditions. Trapping of carbon-centered radical **7**, on the other hand, was only observed for thermal hydrosilylation. To gain further insight into the reaction-condition-dependent contribution of silyl radicals, we performed double-label experiments with DSiEt_3_ and HSiMeEt_2_ (Figure S45). Analysis of residual silane after a reaction time of 23 h in the absence of light furnished an Et_3_SiD:Et_2_MeSiD ratio of 4.5:1, while a 1:1 ratio was observed for photochemical conditions. Since a 1:1 ratio of Et_3_SiD and Et_2_MeSiD was already established after a reaction time of only 0.5 h in light-mediated hydrosilylation, we could conclude that H/D exchange proceeded very efficiently in the presence of silyl radicals. The limited H/D exchange observed under thermal reaction conditions thus indicates that silyl radicals are only formed in minute amounts under thermal hydrosilylation conditions, while direct formation of **7** constitutes the dominant pathway in the dark. In order to determine a potential route for the formation of **8** under the reaction conditions of thermal hydrosilylation, we considered that the active catalyst is composed of Rh(II)-**3**, **10** and minor amounts of **9**. Since we had previously shown that formation of **10** from **9** proceeds via HAT from the benzene solvent, we tested whether minute amounts of silyl radicals may be generated during the hydrosilylation reaction from the interaction of the silane reagent with residual **9**. FT-IR analysis of a sample of **9** immersed in solvent quantities of Et_2_MeSiH gave rise to a band assigned to **10** (Figure S22), so that HAT with **9** is a reasonable pathway for the formation of trace amounts of **8** under thermal hydrosilylation conditions.

Irrespective of whether **7** is formed directly from **6** (thermal pathway), or whether hydrosilylation proceeds via light-promoted expulsion of **8** from **6** (photochemical pathway), however, Rh(III)–H (**5**) is expected to constitute a key reaction intermediate in both pathways. The ability of C-centered radicals to abstract a hydrogen atom from H–Rh(III) porphyrin complexes was shown already in 1985 by Halpern and co-workers.^47^ Rh(III)–H could therefore undergo hydrogen atom abstraction by a carbon-based radical to yield the hydrosilylation product and regenerate Rh(II).

We thus set out to gather evidence for the formation of Rh(III)–H under both light-mediated and dark reaction conditions. Solid-state ^1^H NMR analysis of **3** recovered for hydrosilylation reactions under light-promoted or dark conditions both furnished a signal at −43 ppm, which indicates the presence of a hydride ligand trans to a vacant coordination site (Figure 9B).^21^ To provide an unambiguous assignment, we also prepared a Rh(III)–H complex derived from the homogeneous model system **18** for the MOF catalyst, the ^1^H NMR signal of which was in good agreement with that obtained from recovered **3** (Figure S46). We could furthermore show that Rh(III)–H **5** was also formed in the absence of ethylene, so that light-mediated release of silyl radical **8** from **6** is a viable reaction step in light-mediated olefin hydrosilylation. To obtain additional evidence that proposed intermediated **5** and silyl radical **8** are formed under the reaction conditions, we performed EPR spectroscopy after addition of the spin trap N-*tert*-butyl-α-phenylnitrone (PBN; Figure 9C). The formation of three different radical intermediates could be detected upon irradiation of a mixture of Rh(II)-**3** and HSiMe_2_Ph. Based on the respective hyperfine coupling constants of the ^1^H nucleus in the α position, we could conclude that PBN adducts of carbon-, silicon-, and hydrogen-centered radicals contribute to the experimentally observed signal. We attribute the formation of the PBN adduct of a hydrogen-centered radical to the interaction of PBN with the Rh(III)-H intermediate **5**. Our assignment is substantiated by a previous report which describes the successful transfer of a hydrogen atom from Au–H to PBN.^48^ To confirm that a PBN adduct of a silyl radical contributed to the experimentally observed signal we repeated the experiment with HSiEt_3_ (Figure S50), whereupon we observed noticeable changes in the EPR spectrum due to small differences in the Si hyperfine coupling with HSiMe_2_Ph versus HSiEt_3_.^49−51^

### Molecular Dynamics Simulations

Formation of carbon-centered radical intermediate **7**, via either addition of **8** to ethylene or direct silyl transfer, is expected to be irreversible: Quantum chemical calculations show that intermediate **7** is stabilized by Δ*G*_calc_ = 15.8 kcal·mol^–1^ relative to **8** and ethylene. We propose that **7** undergoes HAT with Rh(III)-H **5** to reform Rh(II)-**3**. Use of deuterated silane confirmed that the deuterium atom originally present in the silane was fully transferred to the hydrosilylation product (Figure 9E). Molecular dynamics simulations (GFN2-xTB with certain semiempirical parameters refitted against DFT data; see Figure S71) were able to provide additional insight into the fate of key intermediate **7**. Monitoring the evolution of **7** within the MOF pore over a period of 50 ps showed that more than 72% of the carbon-centered radicals were converted into the hydrosilylation product via HAT with Rh(III)–H. Interestingly, as our model contained only one Rh(III)–H bond, the observed product is exclusively formed from **7** that did not escape its solvent cage and reacted with “its own” Rh center. Only 8% of **7** left the solvent cage and thus could potentially undergo HAT with other Rh(III)–H bonds or be trapped by Rh(II) (Figure S72). The H and Si atoms present in the hydrosilylation product, therefore, predominantly originate from the same silane precursor molecule. As anticipated, ethylene expulsion from **7** to yield **8** was not observed. Half of the remaining 20% of **7** underwent addition to the porphyrin linker (Figure S72). Circumstantial experimental evidence that a small fraction of the radicals generated may undergo addition to the porphyrin ligand could be obtained from radical trapping experiments with PBN (Figure 9C). Specifically, trapping of a carbon centered radical was also observed for experiments in which only silane but no ethylene was present, so that the carbon-centered radical that was trapped could not be **7**. Instead, a porphyrin-based radical formed upon addition of **8** to the porphyrin linker may account for the carbon centered radical trapped by PBN. Alternatively, PBN could have undergone reaction with methyl radicals generated from residual Rh(Me)-**3** that was still present in the Rh(II)-**3** sample. Digestion of Rh(II)-**3** followed by solution ^1^H NMR analysis did not reveal the presence of any remaining Rh(III)-Me. However, considering the higher sensitivity of EPR compared with NMR, we cannot rule out that the carbon-based radical trapped by PBN corresponds to methyl radicals derived from Rh(Me)-**3** impurities in Rh(II)-**3**. EPR analysis of recovered catalyst samples also repeatedly showed peaks of varying intensity with *g* = 2, which could indicate the presence of a porphyrin centered radical or a Rh superoxo species.^52^ While prior EPR studies of porphyrin-containing MOFs have attributed similar features to porphyrin centered radicals,^45,53^ we also observed the presence of the signal in question for **3** which had never been subjected to hydrosilylation catalysis (Figure 7E). Detailed NMR studies of MOF linkers obtained by digestion of **3** that was recovered from hydrosilylation reactions could provide only tentative evidence for the addition of radical intermediates to the porphyrin ring (Figures S53–S54). The signals corresponding to new species were extremely weak, which debarred structural assignment, and we could not conclusively rule out the possibility that the linker structure was modified during the MOF digestion process (Figures S53–S55). Considering all available data, we conclude that radical addition to the porphyrin linker may take place, but we cannot comment to the extent or reversibility of this process. Should the porphyrin linker in **3** undergo derivatization, however, it does not appear to affect its catalytic performance, at least within the first 8 uses of the catalyst.

### Assistance by Adjacent Rh Center

**Figure 10:**
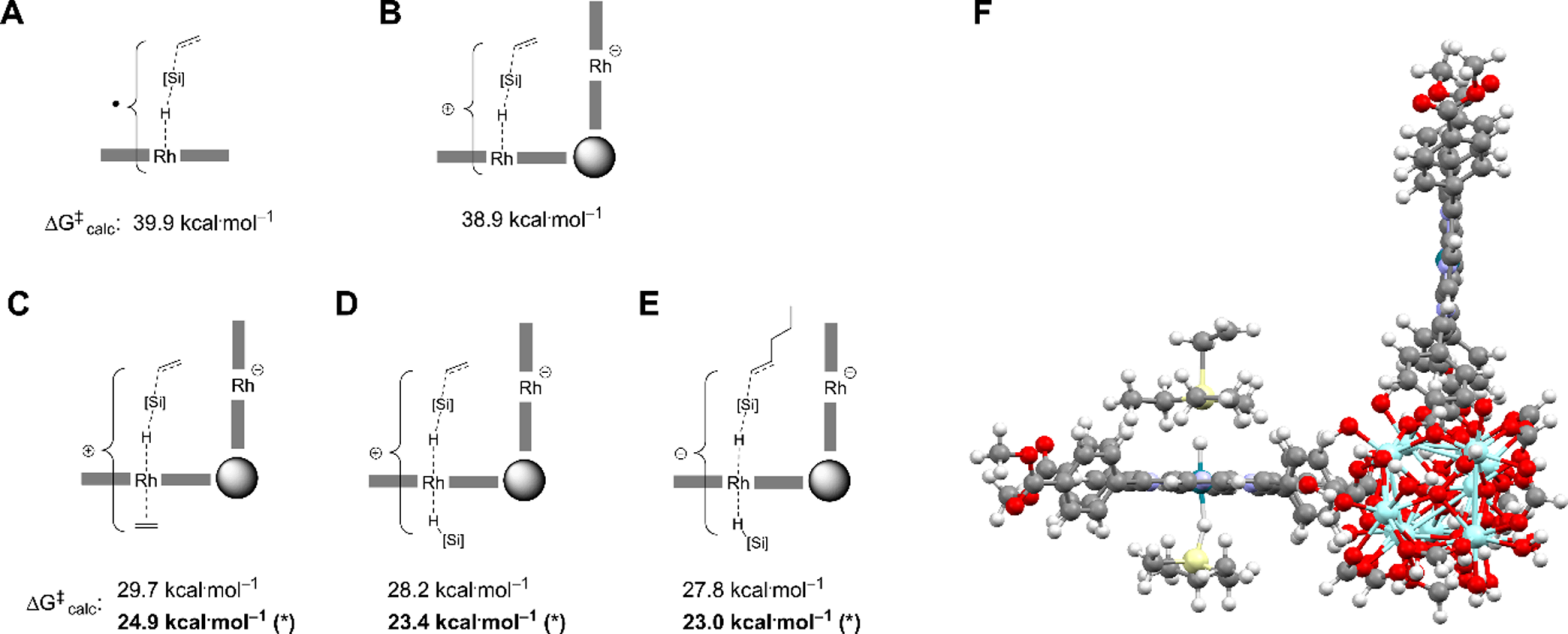
(A–E) Potential transition state structures along with the Gibbs energy of activation obtained for them with the ORCA program package^44^ at the DLPNO–CCSD(T)/SARC-ZORA-TZVP(Rh)/ZORA-def2-TZVP(C,H,O,N,Si)//BP86-D3/SARC-ZORA-TZVP(Rh)/ZORA-def2-TZVP(Si)/ZORA-def2-SVP(C,H,O,N) level. *Polarity of MOF environment considered in calculation. (F) Theoretical model of the proposed transition state structure (corresponds to model 10D).

While we had gathered various pieces of experimental evidence that the presence of ethylene enables H–Si bond cleavage at room temperature, a transition state model constructed based on the existing data delivered an unrealistically high calculated Gibbs energy of activation (Figure 10A). Considering that thermal hydrosilylation with Rh(II)-**3** can occur at room temperature, it cannot be associated with a Gibbs energy of activation that substantially exceeds ∼20 kcal·mol^–1^. Numerous alternative proposed transition structures also furnished unrealistically high activation barriers or could not be located by our computational approach (Figure S60). Since initial models considered only one isolated Rh(II) porphyrin center, we considered that the discrepancy may arise from the promotional effects of the extended environment. Interestingly, when a second Rh(II) porphyrin center was positioned at the distance set by the MOF framework, an electron transfer from the Rh–H–Si-ethylene adduct to the adjacent Rh(II) center could take place. Electron transfer gave rise to a zwitterionic transition state (Figure 10B) that was slightly lower in energy than the neutral transition state (Figure 10A). Prior EPR studies had shown that simultaneous addition of silane and ethylene to Rh(II)-**3** led to the formation of bis-adduct **13** (Figure 8). Trans-ligation of Rh by an electron-donating ligand would be expected to stabilize the cationic Rh center present in a zwitterionic transition state (Figure 10B) and thus lower the barrier associated with it. Theoretical models indicated that, while an ethylene adduct is accessible, it is 4.7 kcal·mol^–1^ less stable than a benzene adduct of **6**. However, the increased electron donation provided by ethylene compared to that of benzene as the trans ligand on Rh(II) led to superior stabilization of the transiently positively charged transition structure. Even taking into account the estimated energy penalty of 4.7 kcal·mol^–1^ for the formation of **13**, the transition state structure depicted in Figure 10C gave rise to the lowest barrier observed thus far (29.7 kcal·mol^–1^). Interestingly, an intrinsic reaction coordinate (IRC) analysis showed that the electron transfer from the ethylene adduct of **13** to an adjacent Rh(II) center occurs immediately prior to the transition state. Shortly after the barrier is crossed, a second electron transfer takes place, which returns both Rh centers to an oxidation state of two (Figure S67).

While the theoretical model now accounted for the promotional effect of an adjacent rhodium center, it did not reproduce the polarity of the extended environment. Since computation of an entire MOF pore at even the DFT level with a double-ζ basis set was too expensive, we accounted for the higher polarity of the MOF pore environment versus a benzene solvent environment via a correction to the dielectric constant in the solvent model (Table S23). Use of a dielectric constant consistent with the calculated polarizability of the MOF, as well as complete filling of the MOF’s pores by benzene, furnished a theoretical Gibbs energy of activation of 24.9 kcal·mol^–1^. Considering the importance of the trans ligand in enabling the electron transfer process that leads to an accessible reaction barrier, we also compared the effect of trans-ligation by different potential ligands (diethylmethylsilane and olefins such as ethylene and 1-pentene) on the reaction barrier. When the polarity of the MOF environment was taken into account, theoretical barriers of 24.9 kcal·mol^–1^ and 23.4 kcal·mol^–1^ were obtained for ethylene hydrosilylation with trans-ligation by ethylene (Figure 10C) and silane (Figure 10D, 10F), respectively. In the case of 1-pentene hydrosilylation, we likewise obtained comparable calculated barriers when either 1-pentene (see Section 18.4 of SI) or diethylmethylsilane (Figure 10E) occupied the trans-coordination site on the rhodium center. Interestingly, computational data predicted that displacement of a benzene ligand by silane is thermodynamically favored over displacement of benzene by ethylene by 2.3 kcal·mol^–1^, while ethylene adduct **13** rather than a silane adduct was observed by EPR spectroscopy. Because EPR data were collected at 93 K, however, and the calculations performed with a nominal temperature of 298 K, the disparate predictions are not necessarily inconsistent. Based on the available data, we concluded that ethylene, 1-pentene and silane all constitute potentially competent trans ligands, and we performed additional theoretical work with an ethylene trans ligand as a representative example (Figures S66, S67, S72, S73).

While the theoretical model clearly highlighted the beneficial effect of a second Rh(II) center in close proximity to the active site, the computed reaction pathway was not yet consistent with a transformation that could take place at room temperature. Notably, however, the theoretical model does not account for the presence of Rh(III)–O–OH within the MOF pore. While the beneficial effect of the presence of these polar groups could be clearly established experimentally, their absolute concentration or spatial distribution is not known, which precluded inclusion in the theoretical model. Importantly, however, a number of experimentally testable hypotheses result from the lowest energy pathway provided by theoretical calculations: (i) the involvement of ethylene in the highest energy transition state suggests that thermal hydrosilylation is associated with an ethylene order of at least 1, and potentially 2 (if ethylene serves as the trans ligand on Rh(II) as depicted in Figure 10C), (ii) a polar solvent environment would be expected to stabilize the partial charges created in the transition state of the thermal hydrosilylation reaction, and (iii) the amount of dehydrosilylated side product obtained would be expected to be higher for the thermal pathway due to the possibility of facile proton loss from the zwitterionic transition structure.

We had shown in our earlier work that thermal conditions led to a hydrosilylation selectivity of 92% whereas light-mediated conditions were associated with a selectivity of 99%. The light-mediated pathway, which proceeds via free silyl radicals, is substantially faster at room temperature than the thermal pathway, which proceeds by direct silyl transfer via a zwitterionic transition. The selectivity of a light-mediated reaction at room temperature is thus expected to be dominated by the selectivity associated with the silyl radical reaction pathway. When the photochemical reaction is carried out at 50 °C, the contribution of the thermal pathway becomes substantial, and the selectivity for the formation of the hydrosilylated product drops to 93%. The formation of an increased amount of dehydrosilylated product whenever the thermal reaction pathway is a dominant contributor to the reaction rate is consistent with the zwitterionic transition states depicted in Figure 10B-10F. We also investigated the origin of the substantially lower selectivity observed in light-mediated hydrosilylation with the molecular analogue of Rh(III)-**3**, the results of which are discussed in the Supporting Information (Figure S75).

### Kinetic Isotope Effect

To assess whether kinetic analysis of ethylene hydrosilylation would shed light on the rate-determining transition state or would instead be overshadowed by mass transport limitations, we determined the kinetic isotope effect. The formation of the σ-adduct, which is the only step preceding Si–H cleavage, is not associated with any notable weakening of the Si–H bond and therefore does not show a significant equilibrium isotope effect.^54^ Consequently, the experimental kinetic isotope effect (KIE) could be determined by direct competition of HSiEt_3_ and DSiEt_3_, which ensures higher accuracy for a heterogeneous (light-mediated) reaction than would be possible for the comparison of absolute rate constants.^55^ Determination of the H/D ratios for tetraethylsilane formed in the presence of both HSiEt_3_ and DSiEt_3_ furnished a KIE of 1.85 ± 0.24 for light-mediated hydrosilylation and 1.90 ± 0.25 for thermal hydrosilylation with Rh(II)-**3** (Tables S24–S25). The KIE was identical within experimental error for the light-mediated and the dark reaction. A KIE of 1.9 ± 0.25 substantiated that Si–H cleavage takes place in the turnover limiting step, which is the case for both reaction pathways we have outlined. Furthermore, the experimental kinetic isotope effect is in excellent agreement with the theoretically predicted value of 1.9 for the dark reaction (Figure S74). Notably, because the experimental intermolecular KIE is not substantially lowered compared with theoretical predictions, mass transfer of the silane through the MOF pore network is not limiting the reaction rate.^56^

### Reaction Orders and Eyring Analysis

During kinetic investigations of thermal olefin hydrosilylation, we had to rely on analysis of reaction aliquots because mass transfer limitations were observed when thermal hydrosilylation was carried out inside an NMR tube. Interestingly, even the dissolution of ethylene could become substantially inhibited within narrow vessels. In-situ monitoring of ethylene hydrosilylation reaction catalyzed by Rh(II)-**3** led to an abrupt cessation of conversion once the ∼0.6 equiv of ethylene initially dissolved in the reaction solvent were converted, despite the availability of additional ethylene in the headspace of the NMR tube (Figure S11). We could confirm ethylene dissolution limitations by EPR, where the concentration of ethylene-containing species derived from Rh(II)-**3** was strongly dependent on the extent to which EPR tubes were shaken (Figure S29). Determination of reaction orders was thus carried out in standard reaction vials from which aliquots were repeatedly collected for NMR analysis. A change in the olefin substrate from ethylene to 1-pentene was deemed necessary to prevent escape of ethylene during sampling. A catalyst reaction order of one, as well as a silane reaction order of 1.3 could be determined by reaction progress kinetic analysis via graphical overlay developed by Blackmond and Bures (Figures S79–S86).^57−59^ Reaction order analysis for 1-pentene led to a value of 0.7, but due to the larger variability within data sets collected for 1-pentene, we could only conclude that the 1-pentene order was nonzero and likely ∼1 (Figures S87–S89). The reaction orders determined are consistent with a rate-limiting transition state that involves Rh(II)-**3**, silane, and olefin. Notably, theoretical calculations suggest that the coordination of an electron-donating substituent such as ethylene or silane in the trans position of the Rh(II) porphyrin leads to a substantial reduction in calculated Gibbs energy of activation. The experimentally determined 1-pentene order of ∼1 excluded 1-pentene coordination in the trans position of Rh(II) during 1-pentene hydrosilylation. On the other hand, a silane order of 1.3 is consistent with the participation of more than one silane molecule in the generation of transition state, which supports a transition state model in which silane occupies the trans-axial coordination site on the rhodium center (Figure 10E).

Since all preceding experimental work was carried out with ethylene as a substrate, we determined the experimental Gibbs energy of activation for the hydrosilylation of ethylene itself. It was practically challenging to obtain temperature dependent kinetic data for a reaction that requires a solid catalyst, liquid, and gaseous reagents and for which the oxygen concentration has a substantial effect on the reaction rate, so that the quality of the data obtained was only modest (Tables S29–31). Nonetheless, experimental activation parameters of Δ*G*^⧧^_exp_ (298 K) = 19.4 ± 0.6 kcal·mol^–1^, and Δ*H*^⧧^_exp_ = 17.6 ± 2.7 kcal·mol^–1^ could be extracted from an Eyring analysis (Tables S32–33, see section 21.2 in SI). To consider the possibility that ethylene may serve as the trans ligand for Rh(II) in ethylene hydrosilylation, we calculated Δ*G*^⧧^_exp_ for both an olefin order of 1 (the experimentally determined value for 1-pentene) and 2 (feasible alternative), which led to values of Δ*G*^⧧^_exp_ = 20.0 kcal·mol^–1^ (ethylene order = 1) and Δ*G*^⧧^_exp_ = 18.8 kcal·mol^–1^ (ethylene order = 2), respectively. Consequently, even the upper end of the error range for Δ*G*^⧧^_exp_ is substantially below the minimum barrier required to promote hydrosilylation via silyl radical intermediates. It must be stated, however, that direct comparison of the numerical values for the barriers does not provide immediate information about the practical feasibility of the two reaction pathways due to a difference in reaction orders. Unlike the rate of olefin hydrosilylation via the intermediacy of silyl radicals, which would not be expected to depend on the olefin concentration, direct silyl transfer to olefins has a nonzero olefin order, so that low concentrations of olefin could lead to a low reaction rate even for low values of Δ*G*^⧧^_exp_.

### Solvent Effect

**Figure 11:**
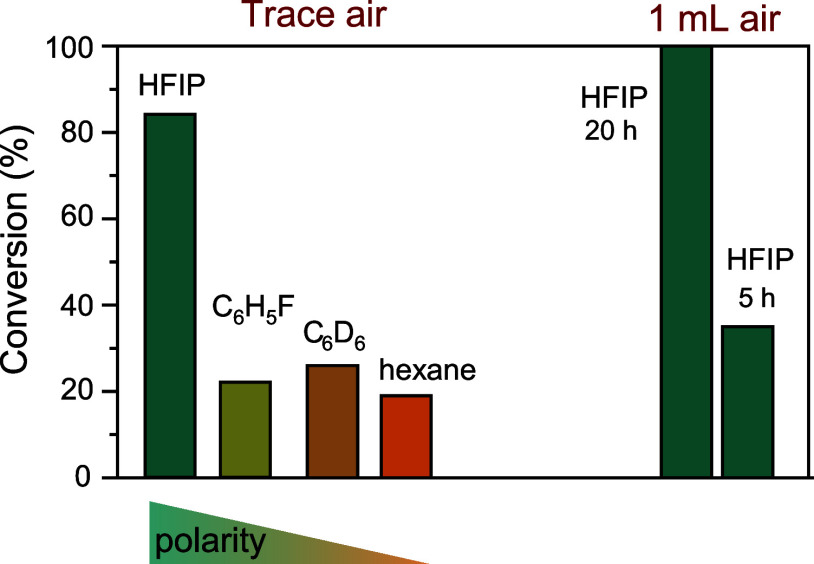
Solvent effect on thermal ethylene hydrosilylation catalyzed by Rh(II)-**3** (room temperature, 20 h reaction time, unless stated otherwise).

Based on the mechanism proposed for direct silyl radical transfer, a rate acceleration would be expected when the polarity of the solvent is increased: Electron transfer from the adjacent porphyrin center leads to the generation of charges in the transition state, so that a polar solvent environment would be expected to stabilize the transition structure. To ensure that any observed rate differences were not caused by varying ethylene concentrations in solution, we selected a number of solvents for which the experimentally determined ethylene solubility fell into a narrow range (Table S42).^60^ For *n*-hexane, benzene, chlorobenzene, and hexafluoroisopropanol (HFIP), the total amount of ethylene present during experiments corresponded to 2.2–2.5 equiv relative to silane, of which 0.5–0.8 equiv were present in solution. Hexane, which showed the highest ethylene solubility but has the lowest dielectric constant (*ε* = 1.88),^60^ gave rise to slower silane conversion (Figure 11).

On the other hand, replacement of benzene with HFIP (*ε* = 16.7) increased the conversion to above 80% after a reaction time of 20 h. We also examined ethylene hydrosilylation in HFIP with Rh(II)-**3** prepared in the presence of 1 mL of air: complete conversion was observed after 20 h, and even after 5 h, a conversion of 32% had already been reached (Figure 11). We attribute the rate enhancement to the formation of a larger number of Rh(III)–O–OH sites, which may provide internal solvation to polar transition structures assembled on adjacent Rh(II) centers. The observation of a pronounced oxygen effect in a polar solvent such as HFIP suggests that solvent molecules in the pore cannot fully compensate for the polar stabilization that Rh(III)–O–OH moieties provide to the zwitterionic transition state.

## Discussion

We embarked on a systematic study to understand why Rh(II) porphyrin centers site-isolated in a MOF host were able to promote thermal hydrosilylation of olefins, whereas structurally analogous molecular catalysts proved to be unreactive. One potential challenge with the molecular catalyst is that dimerization via Rh–Rh bond formation readily takes place, which depletes the concentration of Rh(II) porphyrin. Preserving a monomeric Rh(II) species in solution is insufficient, however, because no thermal hydrosilylation is observed with **1**, which is sterically shielded from Rh–Rh bond formation. While steric bulk is effective for preventing Rh–Rh bond formation, it offers poor protection for 3c-3e adducts: Even the extreme steric protection offered by four 2,6-diisopropyl phenyl substituents was able to stabilize only a porphyrin Rh(II)-ethylene 3c-3e adduct at low temperatures. The geometric protection offered by the MOF matrix permits the formation of a 3c-3e ethylene π-adduct and even a 3c-3e silane σ-adduct that both remain stable at room temperature. The combination of stability and steric accessibility of the novel 3c-3e silane σ-adduct **6** permits it to engage with other reaction partners, such as olefins. Preassembly of silane and Rh(II) to form **6** renders it entropically feasible to assemble a tricomponent transition state in which the C–Si bond is formed in concert with formation of the Rh–H bond and cleavage of the H–Si bond. For a molecular complex in solution, a corresponding tricomponent transition state is entropically inaccessible because no 3c-3e silane σ-adduct can be generated. Site-isolation alone is, however, insufficient to enable facile direct silyl radical transfer: We found that electron transfer that takes place as the system approaches the transition state is necessary to enable olefin hydrosilylation to take place at room temperature. While isolated Rh(II) centers could potentially be obtained by attachment of suitably functionalized Rh(II) porphyrin molecules to a support surface, the high degree of spatial organization provided by the MOF host is crucial for ensuring that a Rh(II) center is sufficiently close to a transition structure to engage in facile electron transfer. Additionally, we also found evidence of an unexpected stabilization of the transition structure due to the formation of Rh(III)–O–OH during photolysis of the Rh(Me) precatalyst. Small amounts of O_2_ exert a pronounced beneficial effect on thermal ethylene hydrosilylation, which we rationalized by considering that the cogeneration of minor amounts of Rh(III)–O–OH alongside Rh(II) would position polar groups in close spatial proximity to the active site. The promotional effect of Rh(III)–O–OH remained pronounced when benzene was replaced with HFIP as the reaction solvent, which suggests the effect of proximally located Rh(III)–O–OH is not trivial to recreate via variation of the solvent environment. Notably, both the observation of substantial rate increases in the presence of a polar solvent and an increase in the formation of the dehydrosilylated reaction products support that electron transfer from an adjacent rhodium center occurs during thermal hydrosilylation.

**Figure 12:**
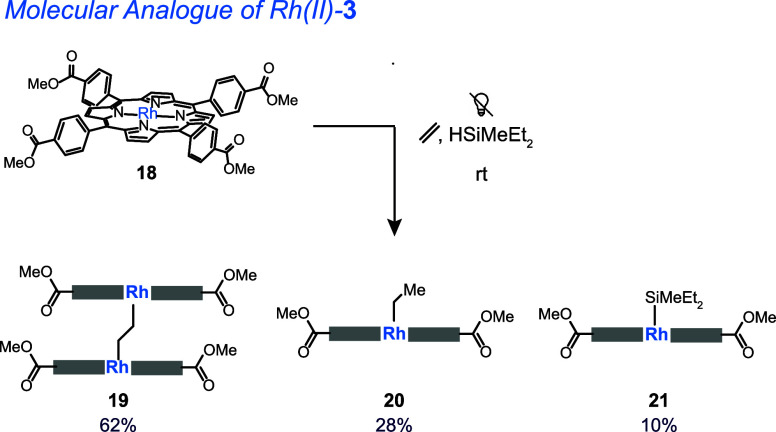
Complex **18**, a molecular analogue of Rh(II)-**3** does not catalyze thermal hydrosilylation.

In an attempt to quantify the beneficial effect that the MOF host imparts on olefin hydrosilylation catalysis, we considered the minimum energy pathways that would enable hydrosilylation to occur in the absence of MOF-specific effects. In solution, **18** rapidly generates **19**, **20** and **21** (Figure 12), and the Rh–Si BDFE for **21** was computationally estimated to be 67.4 kcal·mol^–1^. A thermal cleavage to generate silyl radicals that could engage in hydrosilylation is consequently not feasible. Next, we considered a system containing site-isolated Rh(II) centers that could stabilize 3c-3e silane σ-adducts. H–Si cleavage to release the silyl radical would be thermodynamically accessible (Δ*G*_calc_ = 28.7 kcal·mol^–1^), but kinetically inaccessible due to the entropic contribution to the activation barrier which results in Δ*G*^⧧^_calc_ = 39.1 kcal·mol^–1^. Furthermore, cleavage of the weak Rh–H bond (Δ*G*_calc_ = 3.1 kcal·mol^–1^) in σ-adduct **6** to reform Rh(II) and silane would be expected to take place in preference to silyl radical formation. Lastly, a direct transfer pathway, analogous to the one observed within the MOF host, is associated with Δ*G*^⧧^_calc_ = 39.9 kcal·mol^–1^ if no proximally located electron acceptor is available. The MOF platform thus not only permitted the stabilization of a highly unusual 3c-3e σ-adduct but also enabled a formal silyl radical transfer at room temperature that leverages the spatial organization of Rh(II) centers in the framework. An experimental activation barrier of Δ*G*^⧧^_exp_ = 19.4 ± 0.6 kcal·mol^–1^ was determined for Rh(II)-**3**-catalyzed ethylene hydrosilylation in benzene, which is substantially lower than the barriers associated with both observed and hypothetical reaction pathways outside the MOF.

## Conclusion

We have shown that olefin hydrosilylation catalyzed by site-isolated Rh(II) metalloradicals in **3** or **4** can proceed via two pathways, both of which involve 3c-3e silane σ-adduct **6**. In the presence of light, free silyl radicals are generated from **6**, which subsequently add to olefins. H atom transfer between the resulting carbon-centered radical and Rh(III)–H regenerates the active Rh(II) catalyst. In the absence of light, however, σ-adduct **6** is stable at room temperature both in the dry state and when suspended in benzene. Intermediate **6** preorganizes Rh(II) and silane for participation in a tricomponent transition structure with olefins that permits facile thermal hydrosilylation. By synchronizing Rh–H bond formation, H–Si bond cleavage and Si–C bond formation, the direct silyl transfer pathway limits the number of high-energy intermediates that need to be formed over the course of the reaction. The MOF-based catalyst, which has access to a direct silyl transfer pathway from intermediate **6** can thus promote hydrosilylation at room temperature, while a molecular analogue that was unable to stabilize a 3c-3e silane σ-adduct furnished negligible amounts of product even at 140 °C.

## Supporting Information Available

Detailed procedures for catalysis experiments, control experiments, kinetic data, additional IR and EPR spectroscopy data and discussion of the oxygen effect, data on radical trapping experiments, digestion NMR data for used catalysts, computational data, discussion of decreased selectivity obtained with molecular analogues, kinetic isotope effects, determination of reaction orders and Eyring analysis, additional data on solvent effect, DFT coordination and NMR spectra. (PDF)

## Abbreviations

TEMPO: (2,2,6,6-Tetramethylpiperidin-1-yl)oxyl
AIBN: Azobis(isobutyronitrile)
PBN: N-*tert*-Butyl-α-phenylnitrone
KIE: kinetic isotope effect
HAT: hydrogen atom transfer
