# Supplementary material for: 3-Center-3-Electron σ-Adduct Enables Silyl Radical Transfer below the Minimum Barrier for Silyl Radical Formation
Source: J Am Chem Soc. 2025 Mar 28;147(14):12024–39. doi: 10.1021/jacs.4c18445 (PMC11987034; doi:10.1021/jacs.4c18445)
Supplement: Supplementary file 1 — ja4c18445_si_001.pdf [file ja4c18445_si_001.pdf]

Supporting Information

3-center-3-electron σ-Adduct Enables Silyl Radical Transfer Below the Minimum Barrier for Silyl Radical Formation

Zihang Qiu[1#], Paolo Cleto Bruzzese[2#], Zikuan Wang[1#], Hao Deng[1#], Markus Leutzsch[1], Christophe Farès[1], Sonia Chabbra[2], Frank Neese[1], Alexander Schnegg[2*], and Constanze N. Neumann[1*]

[1]Max-Planck-Institut für Kohlenforschung, Kaiser-Wilhelm-Platz 1, 45470, Mülheim an der Ruhr, Germany.

[2]Max-Planck-Institut für Chemische Energiekonversion, Stiftstrasse 34-36, 45470, Mülheim an der Ruhr, Germany.

*alexander.schnegg@cec.mpg.de

*neumann@kofo.mpg.de

Contents

Supporting Information 1

1 General Information 5

1.1 Chemicals 5

1.2 Experimental Techniques 6

2 General Experimental Procedures 7

2.1 Light-mediated Hydrosilylation 7

2.2 Thermal Hydrosilylation in the Dark 7

2.2.1 Active Rh(II) Catalyst Preparation 7

2.2.2 Hydrosilylation 8

3 Attempted Initiation of Hydrosilylation with AIBN 8

3.1 Light-mediated Hydrosilylation at Room Temperature 8

3.2 Thermal Hydrosilylation in the Dark at Room Temperature 9

3.3 Thermal Hydrosilylation in the Dark at 70 °C 10

4 Light on-off Experiment 11

5 Comparison of Fresh and Recycled Catalyst 13

5.1 Fresh Catalyst 13

5.2 Recycled Catalyst 14

6 Kinetics of Light-mediated Hydrosilylation 15

6.1 Ethylene Hydrosilylation Catalyzed by Rh(III)Me-**3** 15

6.2 Ethylene Hydrosilylation Catalyzed by Rh(II)-**3** 17

7 In-Situ Kinetic Experiments 18

7.1 Experimental Procedure 18

7.2 Challenges Encountered with In-Situ Kinetic Experiments 19

7.3 Larger Scale Synthesis of Rh(II)-**3** 20

7.4 Kinetic Experiments using the Same Batch of Rh(II)-**3** 20

7.5 Mass Transfer Limitation in NMR tubes 22

8 The Oxygen Effect 23

8.1 Solvent Recovery Study 24

8.2 Effect of Air on Different Reaction Steps 25

8.3 Dependence of Catalyst Activity on Oxygen Concentration 26

8.4 Mechanistic Insight into the Oxygen Effect 28

9 EPR Spectroscopy 36

10 Determination of Rh-Rh Distance via CW-EPR 47
11 Radical Trapping Experiments with TEMPO 49
11.1 Light-mediated Conditions 49
11.1.1 In the Presence of Ethylene 49
11.1.2 In the Absence of Ethylene 51
11.2 In the Absence of Light 54
11.2.1 In the Presence of Ethylene 54
11.2.2 In the Absence of Ethylene 56
12 By-product Generation 59
13 Double Label Experiment 61
14 Solid-state NMR Studies 62
14.1 Silane and Light 1 h 62
14.2 Ethylene, Silane and Light 1 h 63
14.3 Ethylene and Silane 2 days 64
15 Radical Trapping Experiments with PBN 68
15.1 With $PhMe_2Si$-H 68
15.2 With Et_3Si-H 70
16 Isotopic Labelling of Silane 71
17 Stability of MOF Linker towards Attack by Radical Intermediates 73
17.1 Digestion Condition A 74
17.2 Digestion Condition B 75
18 Computational Details 78
18.1 Electronic Structure of the Rh-H-Si Complex (**6**) 80
18.1.1 Analysis of Frontier Molecular Orbitals 80
18.1.2 NBO Analysis 85
18.2 Attempt to Optimize Compound **12** 88
18.3 Electron Transfer of **6** to External Electron Acceptors 88
18.4 Role of Electron Transfer and Site Proximity in Reducing the Reaction Barrier 90
18.5 Extra Electrostatic Stabilization of **13-TS-MOF** by the MOF Linkers and Nodes 95
18.6 Si–H Bond Photolysis of **6** 96
18.7 Entropic Barrier of the H–Si Bond Dissociation of **6** 98
18.8 Reaction of Et_2MeSi-CH_2CH_2 Radical (**7**) with Rh(III)-H (**5**) 100
19 Lower Selectivity of Molecular Analogue 104

20 Kinetic Isotope Effect 106
20.1 Light-Mediated Hydrosilylation 107
20.2 Thermal Hydrosilylation in the Dark 111
21 Reaction Orders 113
21.1 Catalyst Order 113
21.2 Silane Order 115
21.3 1-Pentene Order 118
22 Eyring Analysis 120
22.1 Thermal Hydrosilylation at Different Temperatures 120
22.1.1 Reaction at 25 °C 120
22.1.2 Reaction at 50 °C 121
22.1.3 Reaction at 70 °C 121
22.2 Data Analysis 122
22.2.1 For an Ethylene Order of One 123
22.2.2 For an Ethylene Order of Two 129
23 Solvent Effect 134
23.1 Ethylene Solubility in Different Solvents 134
23.2 Solvent Effect with Individually Prepared Rh(II)-**3** 137
23.3 Solvent Effect with Rh(II)-3 from a Single Batch 138
24 List of DFT-Optimized Atomic Coordinates 139
25 Synthesis of **S1** 392
26 NMR Spectra of **S1** 393
26.1 ^{1}H NMR Spectrum in C_6D_6 393
26.2 ^{13}C NMR Spectrum in C_6D_6 394
26.3 ^{29}Si{^{1}H} NMR Spectrum in C_6D_6 395
26.4 ^{1}H-^{29}Si HMBC NMR Spectrum in C_6D_6 396
26.5 ^{1}H-^{103}Rh HMBC NMR Spectrum in C_6D_6 397
27 References 398

1 General Information

1.1 Chemicals

Unless otherwise indicated, chemicals and solvents were obtained from commercial suppliers (Table S1), and used as received. Rh(III)Me-**3**, Rh(II)-**3**, Rh(III)Me-**4** and Rh(II)-**4** were prepared according to procedures we reported previously, along with detailed characterization.[61] Deuterated solvents were obtained from Eurisotop. The 4 Å molecular sieves were activated at 300 °C under dynamic vacuum (5×10^{-6} mbar) for 1 day prior to use. All liquid silanes were degassed using the freeze-pump-thaw method, dried over activated 4 Å molecular sieves, and then stored in a glovebox prior to use. Unless otherwise indicated in the text, C_6D_6 was degassed via by sparging with argon for 30 minutes, then dried over activated 4 Å molecular sieves, and stored in a glovebox prior to use. During the course of this study, however, we found that the amount of residual oxygen can significantly influence the outcomes of catalytic hydrosilylation reactions, so that for some experiments, C_6D_6 was de-oxygenated thoroughly by repeated freeze-pump-thaw degassing, and the amount of air or oxygen indicated in the respective section was added deliberately using a microliter syringe. More details on the amount of oxygen used for particular experiments are provided in the experiment descriptions below. Dry and degassed solvents (diethylether, pentane, dichloromethane, tetrahydrofuran, and toluene) were dried by distillation from an appropriate drying agent in the technical laboratories of the Max-Planck-Institut für Kohlenforschung and stored in Schlenk flasks under argon. MeOH and EtOH were degassed using the freeze-pump-thaw method prior to use. VWR silica gel (40 – 63 µm) was used for filtration and separation. Dry argon was purchased from Air Liquide with >99.5% purity. Ethylene (>99.9% purity) was used for reactions as received from commercial suppliers.

**Chemical Name**	**Supplier**
Triethyisilane-d (97%)<br>*N*-tert-Butyl-α-phenylnitrone (PBN)<br>Azobisisobutyronitrile	Sigma-Aldrich Chemie GmbH
2,2,6,6-Tetramethylpiperidine 1-oxyl (TEMPO)	TCI Deutschland GmbH
Triethylsilane	Sigma-Aldrich Chemie GmbH and TCI Deutschland GmbH
Diethylmethylsilane	ABCR GmbH, Sigma-Aldrich Chemie GmbH and key organics
n-hexane<br>Fluorobenzene	Fisher Scientific GmbH
Hexafluoro-2-propanol (HFIP)	ChemPUR Feinchemikalien und Forschungsbedarf GmbH

Table S1. Reagents obtained from commercial suppliers.

1.2 Experimental Techniques

Mass Spectrometry (MS): mass spectrometry was performed at the mass spectrometry department of the Max-Planck-Institut für Kohlenforschung. ESI and APPI were performed in Q Exactive Plus (Thermo Scientific, Bremen, Germany); GC-EI or GC-CI were performed in ISQ Series Single Quadrupol GC-MS with Trace GC Ultra and AI 1310 Autosampler (Thermo Scientific, San Jose, CA, USA) or Q Exactive GC Orbitrap with Trace 1310 GC and TriPluse Autosampler (Thermo Scientific, San Jose, CA, USA).

Nuclear Magnetic Resonance (NMR) Spectroscopy: NMR data were measured at the NMR spectroscopy department of the Max-Planck-Institut für Kohlenforschung. NMR data were recorded using a Bruker AVIII HD 300 MHz, Bruker AVIII 500, Bruker AVNeo 600 MHz NMR spectrometer at 298/300 K unless indicated otherwise. Chemical shifts (δ) are given in ppm relative to TMS, coupling constants (J) in Hz. The solvent signals were used as internal references and the chemical shifts converted to the TMS scale ($CDCl_3$: δ_H = 7.26 ppm, δ_C = 77.16 ppm; CD_2Cl_2: δ_H = 5.32 ppm, δ_C = 53.84 ppm; C_6D_6: δ_H = 7.16 ppm; δ_C = 128.06 ppm DMSO-d_6: δ_H = 2.50 ppm, δ_C = 39.52 ppm; THF-d_8: δ_H = 3.58, 1.72 ppm, δ_C =67.21, 25.31 ppm).[62] Multiplicity (s = singlet, d = doublet, dd = doublet of doublets, t = triplet, td = triplet of doublets, q = quartet, quint = quintet, sext = sextet, sept = septet, m = multiplet, br = broad singlet) was used to report data. For $^{29}Si\{^1H\}$ NMR characterization, 1D NMR data was acquired via a refocused INEPT sequence.

^{103}Rh NMR shifts were referenced indirectly to the ^{1}H NMR frequency of the sample with the 'xiref'-macro in Bruker TOPSPIN. ^{103}Rh NMR shifts are referenced to $\Xi(^{103}Rh)$ = 3.16%. ^{103}Rh chemical shifts were extracted from the indirect dimension of a ^{1}H-^{103}Rh HMBC or ^{1}H-^{103}Rh HMQC spectrum optimized to J_{HRh} = 1Hz - 2Hz (for porphyrin backbone, Rh-[Si] or Rh-[C]) or J_{HRh} = 40 Hz (for Rh-H). Spectra were measured on a standard Bruker 500 MHz BBFO probe (^{1}H, BB (^{31}P-^{109}Ag)) with z-gradient coil which could be tuned on the broadband X-channel to ^{103}Rh beyond the specifications.

Solid-state NMR spectra were recorded on a Bruker Avance 500WB spectrometer using a double-bearing standard MAS probe (DVT BL4) at resonance frequencies of 500.2 MHz, 125.8 and 99.34 MHz for ^{1}H, ^{13}C and ^{29}Si, respectively. Samples were prepared by transferring ca. 15 mg of the powder sample into a ZrO_2 4 mm rotor inside a glove box and capping the rotor with a Vespel turbine cap with O-ring. For ^{1}H, a simple one-pulse experiment was executed with a 3.4 µs 90°-pulse, 5 s recycling delay and 64 scans. The experimental conditions for the CP MAS NMR spectra were as follows: For ^{13}C: 10 kHz spinning rate, 3.5-s recycle delay, 2k scans, 3 ms contact time, and 3.4 µs ^{1}H 90°-pulse. For ^{29}Si, 8 kHz spinning rate, 3.5-s recycle delay, 8k scans, 5 ms contact time, and 3.4 µs ^{1}H 90°-pulse. The chemical shift was referenced to neat TMS in a separate rotor.)

Powder X-ray diffraction (PXRD): For all measurements, 0.5 mm borosilicate capillaries with an outer diameter of 0.5 mm were used. PXRD measurements were conducted on a STOE STADI P diffractometer operating in Debye Scherrer geometry with a Cu Kα radiation source and a primary germanium monochromator.

Scanning Electron Microscopy (SEM)/ Energy-dispersive X-ray Spectroscopy (EDX): High magnification SEM-images were recorded by the electron microscopy department of the Max Planck Institut für Kohlenforschung on a S-5500 In-lens FE-SEM from Hitachi Europe with 30 kV of acceleration voltage. EDX measurements were conducted using a NORAN System 7 X-ray Microanalysis System with UltraDry EDS

Detector 30mm^2 both from Thermo Fisher Scientific. The samples were prepared by sprinkling on carbon Lacey-Film on copper grids.

Irradiation: All reactions with purple light were carried out using a LED Kessil® PR160L-390 nm as a light source. Reaction vessels were cooled by in-house compressed air flow to ensure that temperature of the reaction mixture was not noticeably elevated above the room temperature.

Infrared Spectroscopy: An Agilent Cary 630 FT-IR instrument with a diamond crystal was used to collect attenuated total reflectance-Fourier transform infrared spectroscopy of samples that were measured inside an argon-filled glovebox. A Nicolet Magna IR 560 instrument with a diamond crystal was used to collect attenuated total reflectance-Fourier transform infrared spectroscopy for samples measured outside the glovebox. Spectra were recorded in the range of 1300 cm^{-1} and 775 cm^{-1} (4 cm^{-1} resolution, at least 128 scans per spectrum) at room temperature.

Glovebox: Argon was used as the glovebox atmosphere with O_2 < 0.1 ppm and H_2O < 0.1 ppm.

2 General Experimental Procedures

2.1 Light-mediated Hydrosilylation

In an argon-filled glovebox, Rh(Me)-**3** (1.0 mg, 0.70 µmol, 0.37 mol%), C_6D_6 (1.0 mL), and diethylmethylsilane (27.7 µL, 191 µmol, 1.00 equiv) were added to a 9 mL glass vial containing a cross-type stir bar (5 x 10 mm). Then the vial was sealed with a screw cap that contains a septum, and transferred out of the glovebox. The reaction mixture was subjected to sonication for 1 minute in order to evenly suspend the MOF crystallites throughout the solvent. Ethylene was then bubbled into the reaction tube for 1 minute in order to saturate the solvent with ethylene. To prevent oxygen leaking into the reaction mixture while ethylene is introduced, the vent needle that releases excess ethylene from the reaction vial was connected to an inert gas manifold connected to an oil bubbler. Then the reaction was stirred at 500 rpm at room temperature and irradiated with a 390 nm purple LED, while the reaction mixture was cooled by in-house compressed air flow. For reaction progress analyses, a 0.01 mL aliquot of the reaction mixture was carefully removed using a syringe after the time indicated and added into an NMR tube alongside 0.5 mL C_6D_6 before the sample was analyzed by NMR spectroscopy.

2.2 Thermal Hydrosilylation in the Dark

2.2.1 Active Rh(II) Catalyst Preparation

In an argon-filled glovebox, Rh(Me)-**3** (1.0 mg, 0.71 µmol, 0.34 mol%) and C_6D_6 (1.0 mL), were added to a 9 mL glass vial containing a cross-type stir bar (5 x 10 mm), then the vial was sealed with a screw cap that contains a septum and transferred out of the glovebox. The reaction mixture was subjected to sonication for 1 minute in order to evenly suspend the MOF crystallites throughout the solvent. After that, Rh(Me)-**3** was subjected to photolysis to produce active catalyst Rh(II)-**3** via irradiation with a 390 nm purple LED for 20 h at room temperature and a stirring rate of 500 rpm. The reaction mixture was cooled during photolysis using in-house compressed air flow. After 20 h, the reaction vial was subjected to centrifugation

to ensure that the solid catalyst settles on the bottom of the vial. The vial containing the reaction mixture was then transferred into the glovebox to remove C_6D_6 before the remaining solid was dried under reduced pressure (~10^{-2} mbar) inside the glovebox.

2.2.2 Hydrosilylation

In the argon-filled glovebox, 1 mL C_6D_6 as well as diethylmethylsilane (30.0 µL, 207 µmol, 1.00 equiv) were added to a glass vial containing 1 mg active catalyst Rh(II)-**3** generated according to the procedure above (Section 2.2.1). The reaction mixture was subjected to sonication for 1 minute in order to evenly suspend the MOF crystallites throughout the solvent. Ethylene was then bubbled into the reaction vial for 1 minute in order to saturate the solvent with ethylene. To prevent oxygen leaking into the reaction mixture as ethylene was introduced, the vent needle that released excess ethylene from the reaction vial was connected to an inert gas manifold which was connected to an oil bubbler. The reaction mixture was then transferred into the glove box and furnished with a new cap to prevent escape of ethylene before it was left to react at room temperature for the time indicated. For reaction progress analyses, a 0.01 mL aliquot of the reaction mixture was carefully removed using a syringe after the time indicated and added into an NMR tube alongside 0.5 mL C_6D_6 before the sample was analyzed by NMR spectroscopy.

3 Attempted Initiation of Hydrosilylation with AIBN

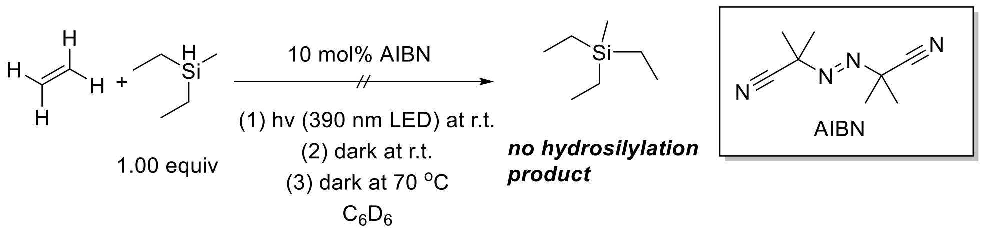

3.1 Light-mediated Hydrosilylation at Room Temperature

In an argon-filled glovebox, AIBN (3.1 mg, 0.019 mmol, 10 mol%), C_6D_6 (1.0 mL), and diethylmethylsilane (27.7 µL, 191 µmol, 1.00 equiv) were added to a 9 mL glass vial containing a cross-type stir bar (5 x 10 mm). Then the vial was sealed with a screw cap that contains a septum, and transferred out of the glovebox. Ethylene was then bubbled into the reaction vial for 1 minute in order to saturate the solvent with ethylene. To prevent oxygen leaking into the reaction mixture while ethylene is introduced, the vent needle that released excess ethylene from the reaction vial was connected to an inert gas manifold connected to an oil bubbler. Following the addition of ethylene, the reaction vial was swiftly transferred into a glovebox to replace the punctured septum cap with a screw cap. After removal from the glovebox, the reaction was stirred at 500 rpm at room temperature and irradiated with a 390 nm purple LED for 20 h, while the reaction was cooled by in-house compressed air flow. The C_6D_6 solution was carefully removed using a syringe and submitted to NMR spectroscopy for analysis.

No hydrosilylation product could be detected (Figure S1).

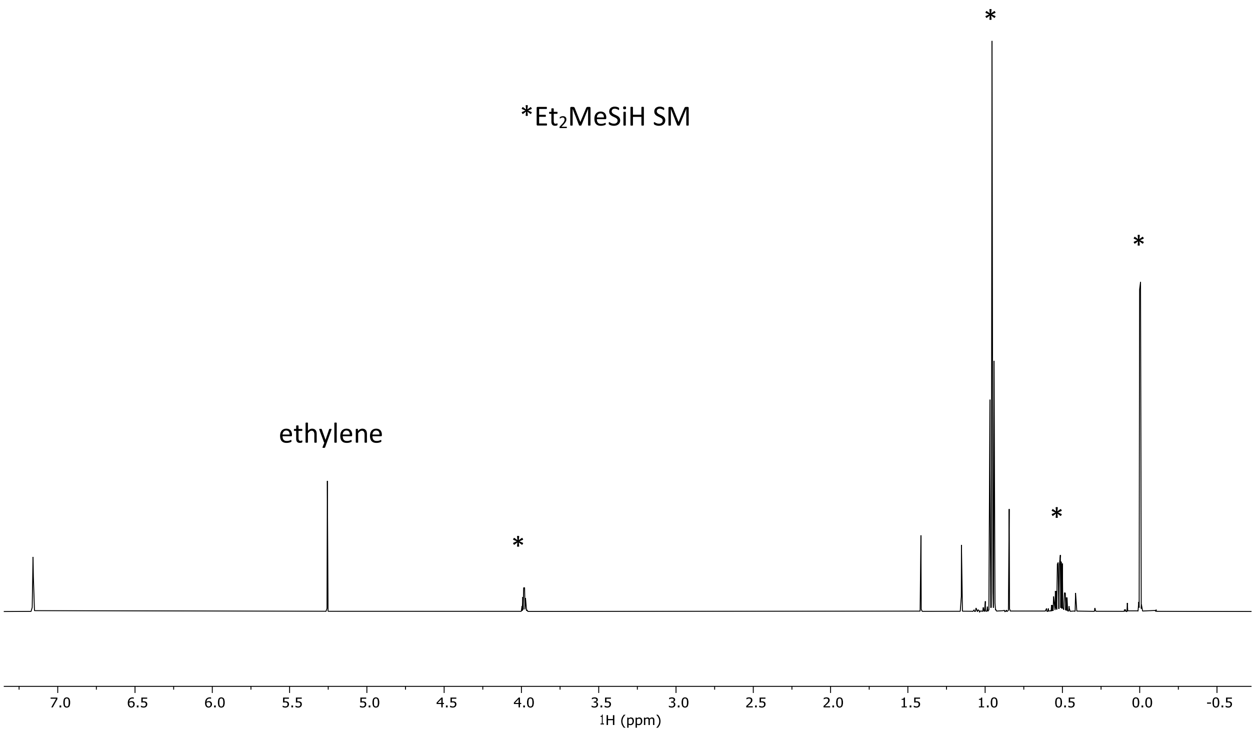

Figure S1. ^{1}H NMR spectrum of the reaction mixture in C_6D_6 after 20 h.

3.2 Thermal Hydrosilylation in the Dark at Room Temperature

The same experimental procedure as above was employed except that no light source was used and the reaction vial was covered with aluminum foil.

Based on ^{1}H NMR analysis of the reaction mixture, no hydrosilylation product was formed (Figure S2).

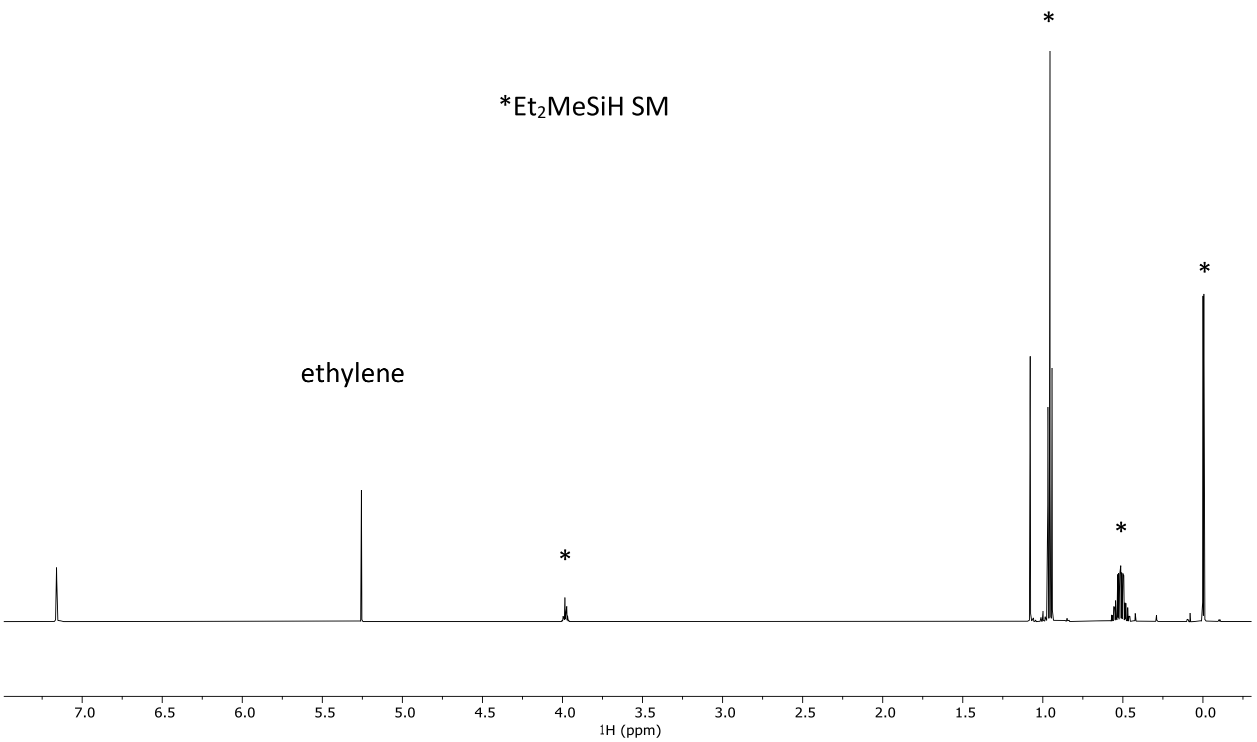

Figure S2. ^{1}H NMR spectrum of the reaction mixture in C_6D_6 after 20 h.

3.3 Thermal Hydrosilylation in the Dark at 70 °C

The same experimental procedure was used as for the light-mediated reaction, except that the reaction was not illuminated, the reaction vial was covered by aluminum foil and heated at 70 ^{o}C for the duration of the experiment.

Based on ^{1}H NMR analysis, no hydrosilylation product could be detected (Figure S3).

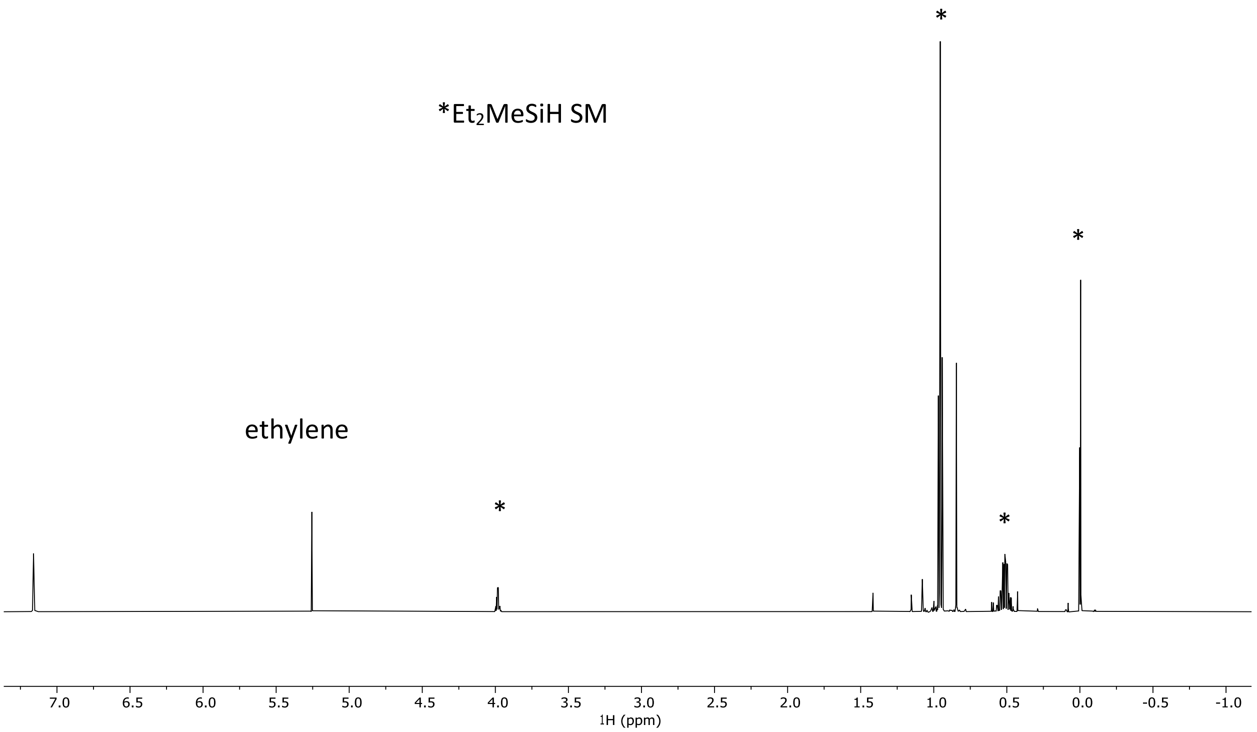

Figure S3. ^{1}H NMR spectrum of the reaction mixture in C_6D_6 after 20 h.

4 Light on-off Experiment

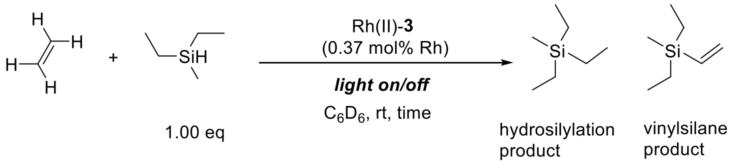

*Generation of active Rh(II)-**3** catalyst:* In an argon-filled glovebox, Rh(Me)-**3** (5.0 mg, 3.5 µmol, 0.37 mol%) and C_6D_6 (5.0 mL), were added to a 10 mL Schlenk glass tube containing a cross-type stir bar (5 x 10 mm), then the tube was sealed with screw cap that contains a septum, and transferred out of the glovebox. The reaction mixture was subjected to sonication for 1 minute in order to evenly suspend the MOF crystallites throughout the solvent. After that, Rh(Me)-**3** was subjected to photolysis to produce active catalyst Rh(II)-**3** via irradiation with a 390 nm purple LED for 20 h at room temperature and a stirring rate of 1000 rpm. The reaction mixture was cooled during photolysis using in-house compressed air flow.

*Use of Rh(II)-**3** in ethylene hydrosilylation:* The Schlenk glass tube containing active catalyst Rh(II)-**3** was transferred into an argon-filled glovebox, and diethylmethylsilane (139 µL, 954 µmol, 1.00 equiv) was added, and transferred out of the glovebox. The reaction mixture was subjected to sonication for another 1 minute in order to evenly suspend the MOF crystallites throughout the solvent. Ethylene was then

bubbled into the reaction tube for 1 minute in order to saturate the solvent with ethylene. To prevent oxygen leaking into the reaction mixture as ethylene was introduced, the vent needle that released excess ethylene from the reaction vial was connected to an inert gas manifold which was connected to an oil bubbler. Following the addition of ethylene for 1 min, a low flow of ethylene was maintained (around one bubble per second in oil bubbler) over the course of the experiment to ensure that sufficient reagent remained present throughout the experiment. Then the reaction was stirred at 1000 rpm at room temperature, and alternatingly subjected to periods of irradiation with a 390 nm purple LED or no irradiation as indicated in Figure S4. To prevent an increase in the temperature due to irradiation, the reaction vial was cooled by in-house compressed air flow. For reaction progress analyses, a 0.01 mL aliquot of the reaction mixture was carefully removed using a syringe after the time indicated and added into an NMR tube alongside 0.5 mL C_6D_6 before the sample was analyzed by NMR spectroscopy.

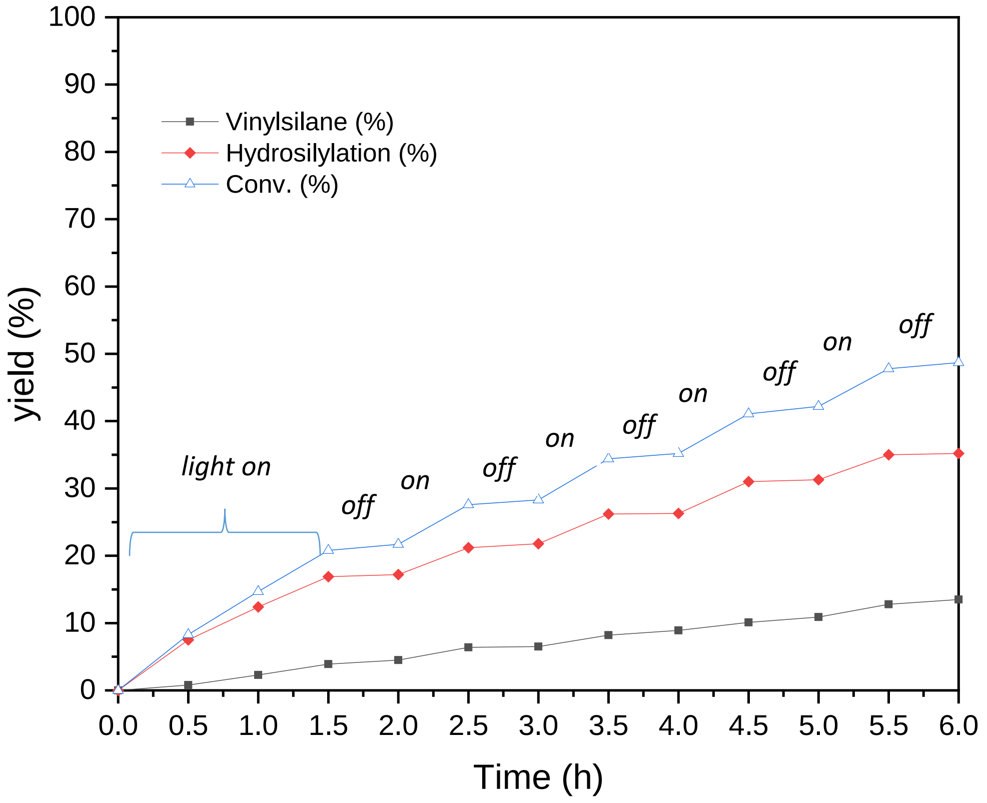

Figure S4. Light on/off kinetic profile of ethylene hydrosilylation reaction catalyzed by Rh(II)-**3**. An unusually large amount of vinylsilane formation was observed to accompany hydrosilylation which we attribute to the removal of multiple reaction aliquots outside the glovebox which led to oxygen contamination of the reaction mixture. The presence of oxygen during ethylene hydrosilylation leads to the formation of increased amounts of vinylsilane by-product formation, which was also observed in our prior work and is discussed further in subsequent sections. A decrease in the reaction rate was observed in the absence of light due to the slower rate of the thermal hydrosilylation reaction at room temperature compared to the light-mediated reaction.

Time (h)	Vinylsilane (%)	Hydrosilylation (%)	Conv. (%)
0	0	0	0
0.5	0.8	7.5	8.3
1	2.3	12.4	14.7
1.5	3.9	16.9	20.8
2	4.5	17.2	21.7
2.5	6.4	21.2	27.6
3	6.5	21.8	28.3
3.5	8.2	26.2	34.4
4	8.9	26.3	35.2
4.5	10.1	31	41.1
5	10.9	31.3	42.2
5.5	12.8	35	47.8
6	13.5	35.2	48.7

Table S2. Light on/off kinetic data of hydrosilylation reaction catalyzed by Rh(II)-**3**. The data points shown are graphically depicted in Figure S4.

5 Comparison of Fresh and Recycled Catalyst

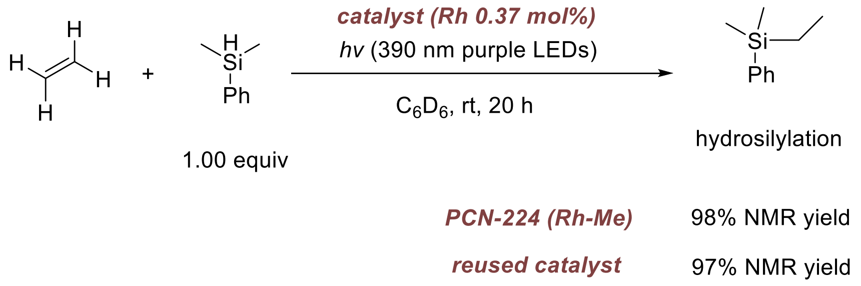

5.1 Fresh Catalyst

In an argon-filled glovebox, Rh(Me)-**3** (1.0 mg, 0.70 µmol, 0.37 mol%), C_6D_6 (1.0 mL), and dimethylphenylsilane (29.2 µL, 191 µmol, 1.00 equiv) were added to a 9 mL glass vial containing a cross-type stir bar (5 x 10 mm). Then the vial was sealed with a screw cap that contains a septum, and transferred out of the glovebox. The reaction mixture was subjected to sonication for 1 minute in order

to evenly suspend the MOF crystallites throughout the solvent. Ethylene was then bubbled into the reaction vial for 1 minute in order to saturate the solvent with ethylene. To prevent oxygen leaking into the reaction mixture as ethylene was introduced, the vent needle that released excess ethylene from the reaction vial was connected to an inert gas manifold connected to an oil bubbler. Following the addition of ethylene, the reaction vial was swiftly transferred into a glovebox to replace the punctured septum cap with a screw cap. After removal from the glovebox, the reaction was stirred at 500 rpm at room temperature and irradiated with a 390 nm purple LED for 20 h while the reaction was cooled by in-house compressed air flow.

The reaction vial was subsequently subjected to centrifugation at 4500 rpm for 5 min (to ensure that the MOF particles settle down on the bottom of reaction vial), and transferred into a glovebox. The C_6D_6 solution was carefully removed using a syringe and submitted to NMR for analysis. Based on ^{1}H NMR analysis, 98% yield of the hydrosilylated product was obtained (Figure S5).

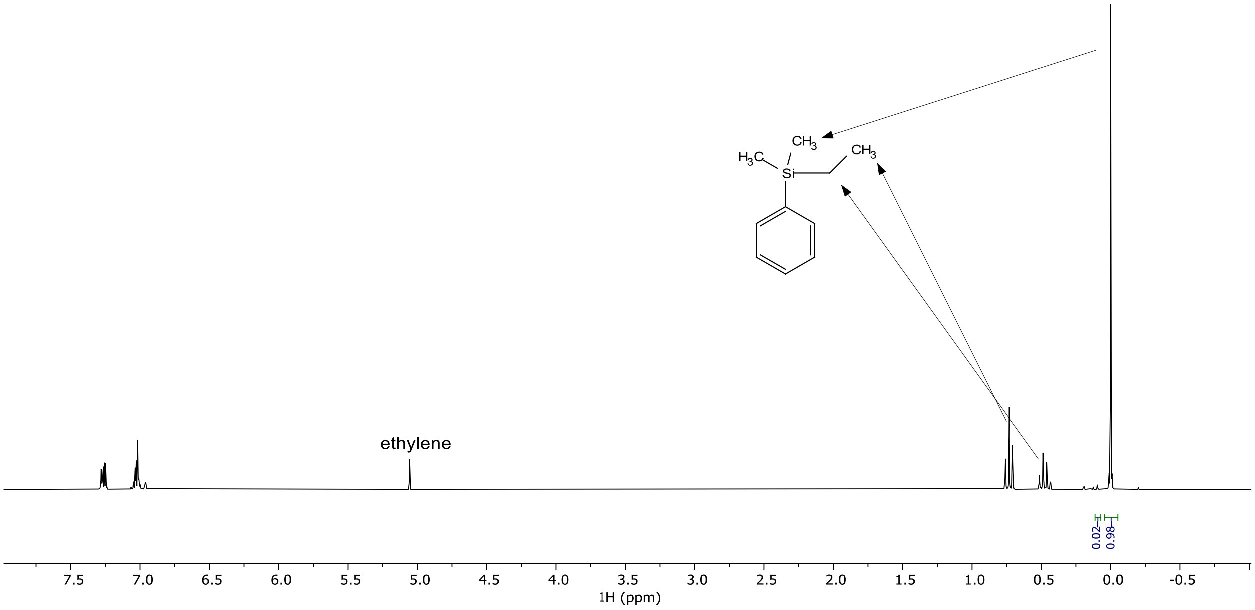

Figure S5. ^{1}H NMR spectrum of the reaction mixture in C_6D_6 catalyzed by fresh catalyst.

5.2 Recycled Catalyst

Catalyst recycling was carried out according to a procedure described in our previous report.[61] The solid catalyst recovered from the above hydrosilylation reaction was used to set up an analogous experiment. Based on ^{1}H NMR analysis, 97% yield of the hydrosilylated product was obtained (Figure S6).

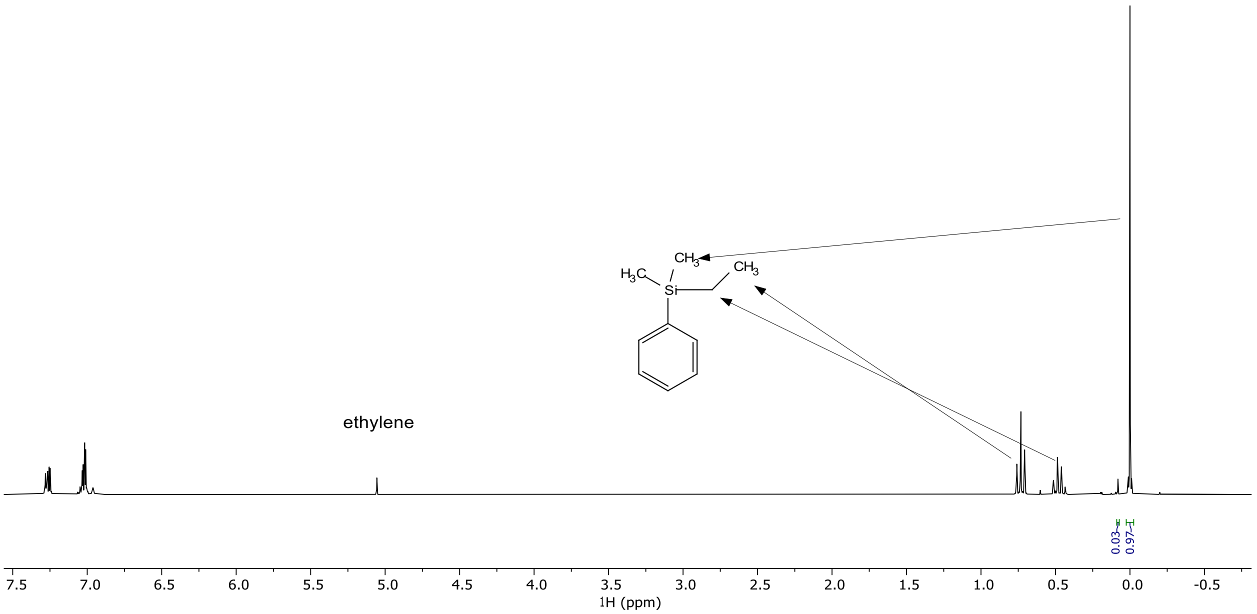

Figure S6. ^{1}H NMR spectrum of the reaction mixture in C_6D_6 catalyzed by reused catalyst.

6 Kinetics of Light-mediated Hydrosilylation

6.1 Ethylene Hydrosilylation Catalyzed by Rh(III)Me-3

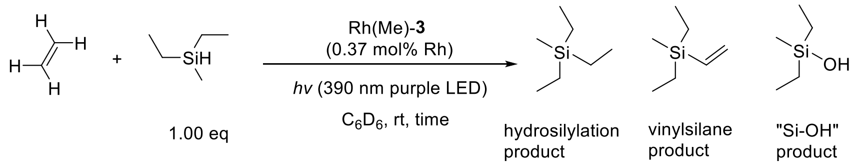

In an argon-filled glovebox, Rh(Me)-**3** (5.0 mg, 0.35 µmol, 0.37 mol%), C_6D_6 (5.0 mL), and diethylmethylsilane (139 µL, 954 µmol, 1.00 equiv) were added to a 10 mL Schlenk glass tube containing a cross-type stir bar (5 x 10 mm). Then the vial was sealed with a screw cap that contains a septum, and transferred out of the glovebox. The reaction mixture was subjected to sonication for 1 minute in order to evenly suspend the MOF crystallites throughout the solvent. Ethylene was then bubbled into the reaction tube for 1 minute in order to saturate the solvent with ethylene. To prevent oxygen leaking into the reaction mixture while ethylene is introduced, the vent needle that releases excess ethylene from the

reaction vial was connected to an inert gas manifold connected to an oil bubbler. Following the addition of ethylene for 1 min, a low flow of ethylene was maintained (around one bubble per second visible in the oil bubbler). Then the reaction was stirred at 1000 rpm at room temperature and irradiated with a 390 nm purple LED, while the reaction was cooled by in-house compressed air flow. After the indicated time had passed (see Table S3), 0.01 mL C_6D_6 solution was carefully removed using a syringe, added into an NMR tube alongside 0.5 mL C_6D_6, and the sample was by NMR spectroscopy**.**

Note: the formation of unusually large amounts of Si–OH side product was attributed to oxygen contamination of the reaction mixture because multiple aliquots were removed from the reaction mixture for analysis. For reactions in which no sampling was carried out, less than 1% silanol (< 0.5% in the majority of cases) was observed as a side product.

Table S3. Amount of respective reaction component detected by ^{1}H NMR analysis of an aliquot removed from the reaction mixture of light-mediated ethylene hydrosilylation with **3** after the time indicated.

Time (h)	Vinylsilane (%)	Hydrosilylation (%)	Si-OH (%)	Conv. (%)
0	0	0	0	0
0.5	0	0	0	0
1	0	0	0	0
1.5	0	0	0	0
2	0	0.7	0	0.7
2.5	0	0.8	0	0.8
3	0	1.2	0	1.2
3.5	0	2.5	0	2.5
4	0	3.6	0	3.6
5	0	6.1	0	6.1
6	0	10.4	0	10.4
7	0	16	0	16
8	0	24.2	0	24.2
9	0	33	0	33
20	8.6	82.9	8.5	100

6.2 Ethylene Hydrosilylation Catalyzed by Rh(II)-3

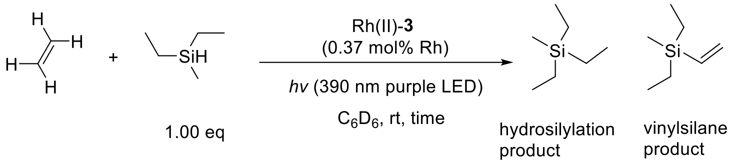

The active Rh(II) catalyst was prepared as follows: In an argon-filled glovebox, Rh(Me)-**3** (5.0 mg, 0.35 µmol, 0.37 mol%) and C_6D_6 (5.0 mL), was added to a 10 mL Schlenk glass tube containing a cross-type stir bar (5 x 10 mm), then the vial was sealed with screw cap that contains a septum and transferred out of the glovebox. The reaction mixture was subjected to sonication for 1 minute in order to evenly suspend the MOF crystallites throughout the solvent. After that, Rh(Me)-**3** was subjected to photolysis to produce active catalyst Rh(II)-**3** with a 390 nm purple LED for 20 h at room temperature and a stirring rate of 1000 rpm. The reaction mixture was cooled during photolysis using in-house compressed air flow.

*Use of Rh(II)-**3** in ethylene hydrosilylation:* The Schlenk glass tube containing active catalyst Rh(II)-**3** was transferred into an argon-filled glovebox, and diethylmethylsilane (139 µL, 954 µmol, 1.00 equiv) was added, before the Schlenk tube was again removed from the glovebox. The reaction mixture was subjected to sonication for another 1 minute in order to evenly suspend the MOF crystallites throughout the solvent. Ethylene was then bubbled into the reaction tube for 1 minute in order to saturate the solvent with ethylene. To prevent oxygen leaking into the reaction mixture as ethylene was introduced, the vent needle that released excess ethylene from the reaction vial was connected to an inert gas manifold which was connected to an oil bubbler. Following the addition of ethylene for 1 min, a tiny counter flow of ethylene was maintained (around one bubble per second in oil bubbler). Then the reaction was stirred at 1000 rpm at room temperature and irradiated with a 390 nm purple LED, where the reaction was cooled by in-house compressed air flow. After the given time passed (Table S4), 0.01 mL C_6D_6 solution was carefully removed using a syringe, added into an NMR tube alongside 0.5 mL C_6D_6, and the sample was analyzed by NMR spectroscopy to obtain the reaction progress profile shown in Table S4.

Time (h)	Vinylsilane (%)	Hydrosilylation (%)	Conv. (%)
0	0	0	0
0.5	0	3.5	3.5
1	0	7.1	7.1
1.5	1.1	10.1	11.2
2	1.4	12.6	14
2.5	2.2	15.5	17.7
3	3.3	18.1	21.4
3.5	4.5	22	26.5
4	5.6	24.4	30
4.5	7.6	26.6	34.2

5	8.3	28.8	37.1
5.5	9.5	32	41.5
6	10.8	34.2	45

Table S4. Amount of respective reaction component detected by ^{1}H NMR analysis of an aliquot removed from the reaction mixture of light-mediated ethylene hydrosilylation with Rh(II)-**3** after the time indicated.

7 In-Situ Kinetic Experiments

We attempted many in-situ experiments in which thermal ethylene hydrosilylation with Rh(II)-**3** was carried out directly inside an NMR tube to permit the collection of closely spaced data points without running the risk of oxygen contamination during sampling. Unfortunately, however, mass transfer limitations associated with the accumulation of the solid catalyst at the bottom of the NMR tube as well as the dissolution of ethylene from the headspace of the narrow reaction vessel prevented the collection of reproducible and reliable data sets. While the following section describes our attempts at direct sampling, please note that the data generated from which was not used for further analysis. To gas outlet from which excess ethylene was released during the dosing process was connected to an inert gas manifold which was connected to an oil bubbler. Reaction orders and activation barriers discussed in the main manuscript therefore relate to the data collected according to procedures described in Sections 19–21.

7.1 Experimental Procedure

Rh(II)-**3** was generated from Rh(Me)-**3** (1.0 mg, 0.71 µmol, 0.34 mol%) according to the general procedure described in Section 2.2.1.

The active catalyst Rh(II)-**3** suspended in C_6D_6 inside a glass vial was transferred into a J-Young NMR tube inside an argon-filled glovebox, and diethylmethylsilane (30.0 µL, 207 µmol, 1.00 equiv) was added. Then the J-Young tube was placed into a larger glass container with a side arm, sealed with a rubber septum and transferred out of the glovebox (Figure S7). Ethylene was then bubbled into solution with a long needle for 1 minute in order to saturate the solvent with ethylene. To prevent oxygen leaking into the reaction mixture as ethylene was introduced, to gas outlet from which excess ethylene was released during the dosing process was connected to an inert gas manifold which was connected to an oil bubbler. After the addition of ethylene directly into the reaction mixture for 1 min, the headspace of the NMR tube was saturate with ethylene before the inlet line was removed. The glass vessel containing the J-Young tube was then quickly transferred back into glovebox and the J-Young tube was sealed firmly with a cap.

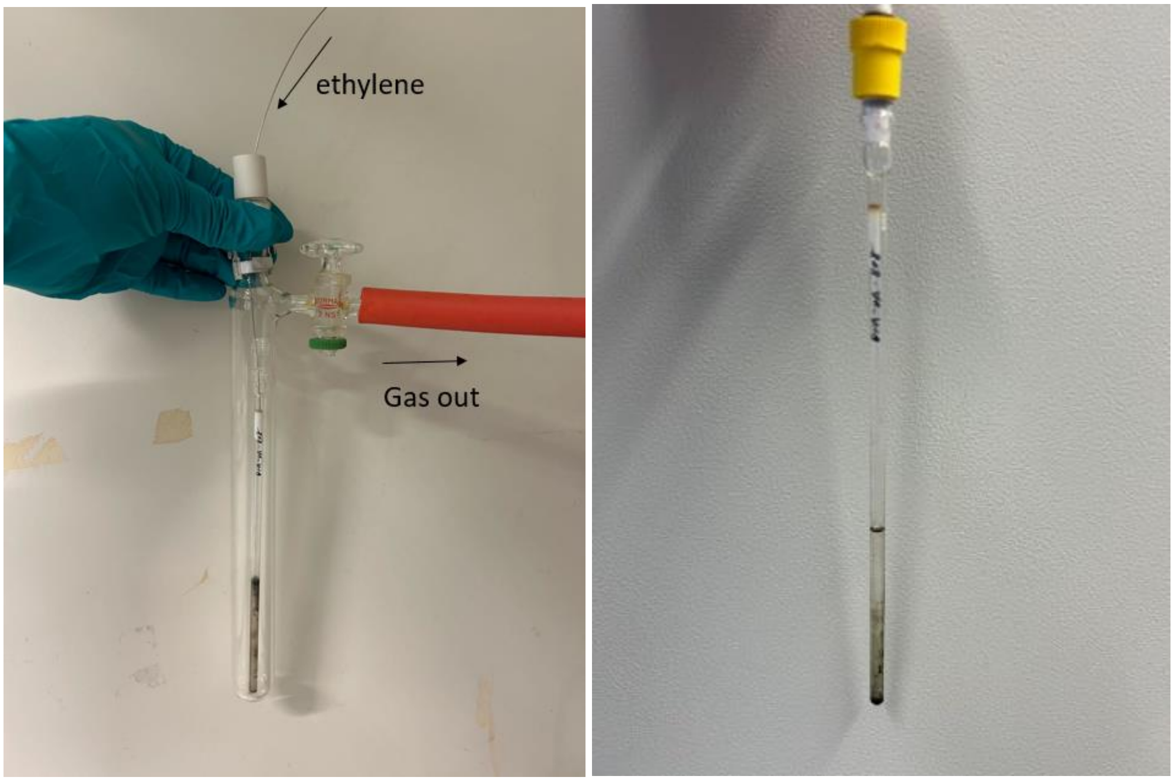

Figure S7. The set-up used for the introduction of ethylene into a J-Young NMR tube outside glovebox.

7.2 Challenges Encountered with In-Situ Kinetic Experiments

Smooth conversion of ethylene to the hydrosilylated product was observed (Figure S8), however, the conversion reached after 20 h was not consistent with batch reactions and varied between individual reactions carried out inside NMR tubes. To ensure that experimental variability was not due to variation in the amount of oxygen present during the generation of the active Rh(II) catalyst, we carried out the conversion of Rh(III)Me-**3** to Rh(II)-**3** on a larger scale (see Section 7.3.). To eliminate any variability that originates from differences in the activity of individual batches of Rh(II)-**3**, we set up multiple ethylene hydrosilylation reactions under identical reaction conditions and using the same batch of Rh(II)-**3** (see Figure S9 and S10).

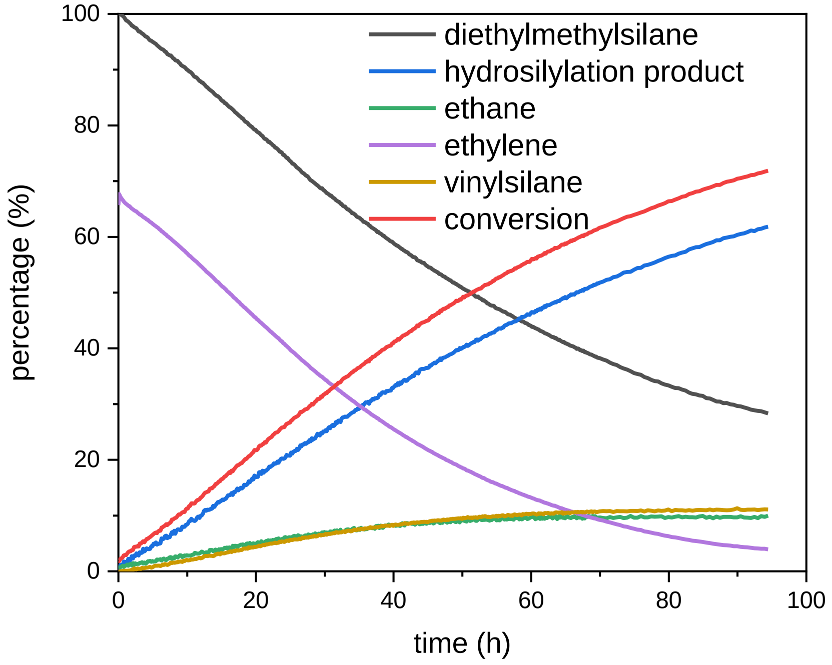

Figure S8. In situ kinetic data of hydrosilylation reaction catalyst by Rh(II)-**3**.

7.3 Larger Scale Synthesis of Rh(II)-3

In an argon-filled glovebox, Rh(Me)-**3** (20.0 mg, 14.1 µmol) and C_6D_6 (15.0 mL), were added to a 20 mL glass vial containing a cross-type stir bar (5 x 10 mm), then the vial was sealed with a screw cap that contains a septum, and transferred out of the glovebox. The reaction mixture was subjected to sonication for 1 minute in order to evenly suspend the MOF crystallites throughout the solvent. After that, Rh(Me)-**3** was subjected to photolysis to produce active catalyst Rh(II)-**3** with irradiation of a 390 nm purple LED for 1 d at room temperature with a stirring rate of 1000 rpm. The reaction mixture was cooled to room temperature during photolysis using in-house compressed air flow. After 1 d, the reaction vial was subjected to centrifugation and transferred into the glove box to remove the solvent and stir bar. About 20 mg dry Rh(II)-**3** catalyst could be obtained which was stored inside the glovebox. We confirmed that, for batch reactions, the yield of ethylene hydrosilylation after a reaction time of 20 h did not show any noticeable variation irrespective of whether Rh(II)-**3** generated immediately prior or Rh(II)-**3** that had been stored in the glove box for a week was used.

7.4 Kinetic Experiments using the Same Batch of Rh(II)-3

Inside an argon-filled glovebox, in a J-Young NMR tube, Rh(II)-**3** prepared according to Section 7.3. (1.0 mg, 0.71 µmol, 0.34 mol%), diethylmethylsilane (30.0 µL, 207 µmol, 1.00 equiv) and 1 mL C_6D_6 was added. Then the J-Young tube was placed into a larger glass container sealed with a rubber septum and transferred them out of the glovebox (Figure S7). Ethylene was then bubbled into solution with a long needle for 1 minute in order to saturate the solvent with ethylene. To prevent oxygen leaking into the reaction mixture while ethylene was introduced, another outlet that released excess ethylene from the dosing process was connected to an inert gas manifold which was connected to an oil bubbler. After the

addition of ethylene directly into the reaction mixture for 1 min, the headspace of the NMR tube was saturated with ethylene before the inlet line was removed. The glass vessel containing the J-Young tube was then quickly transferred back into glovebox and the J-Young tube was sealed firmly with a cap.

We performed two parallel experiments (run 1 and 2). Both experiments showed a substantial decrease in the reaction rate once a conversion of around 55% conversion was reached (Figure S9 and Figure S10). Notably, around 0.6 equivalents of ethylene were initially dissolved in the reaction solvent while additional ethylene was present during the experiment in the headspace of the J-Young tube. Furthermore, even the initial rate differed noticeably between the two experiments even though the experimental conditions were identical and Rh(II)-**3** from the same batch was used for both runs.

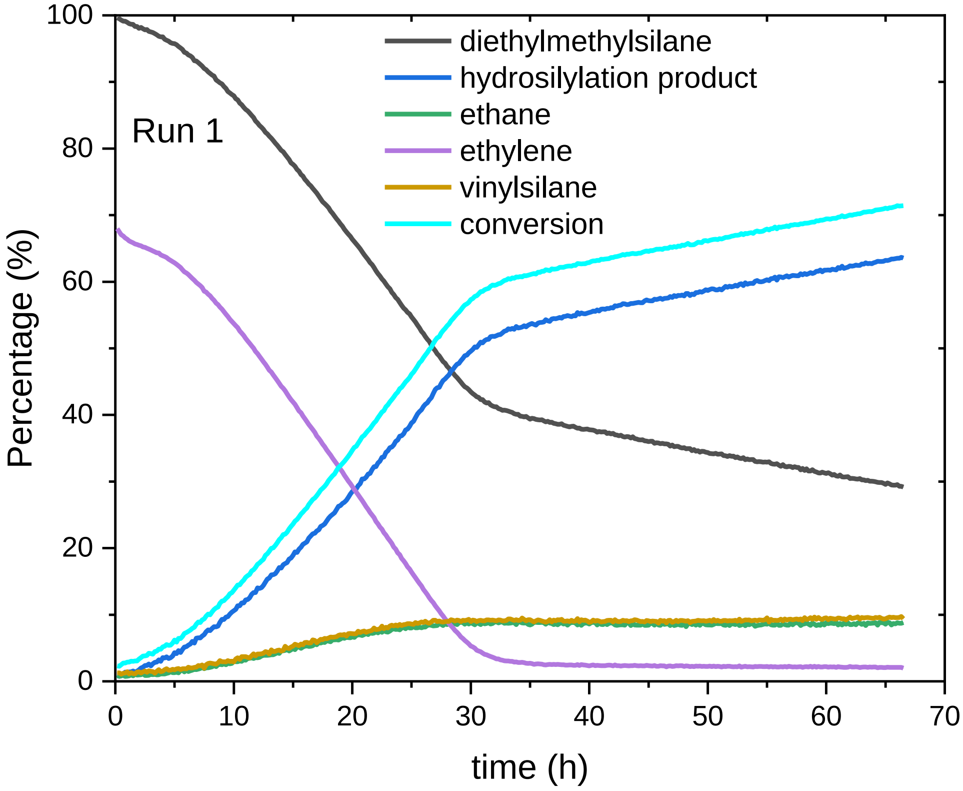

Figure S9. Amount of respective reaction components detected by in situ ^{1}H NMR from ethylene hydrosilylation with Rh(II)-**3**.

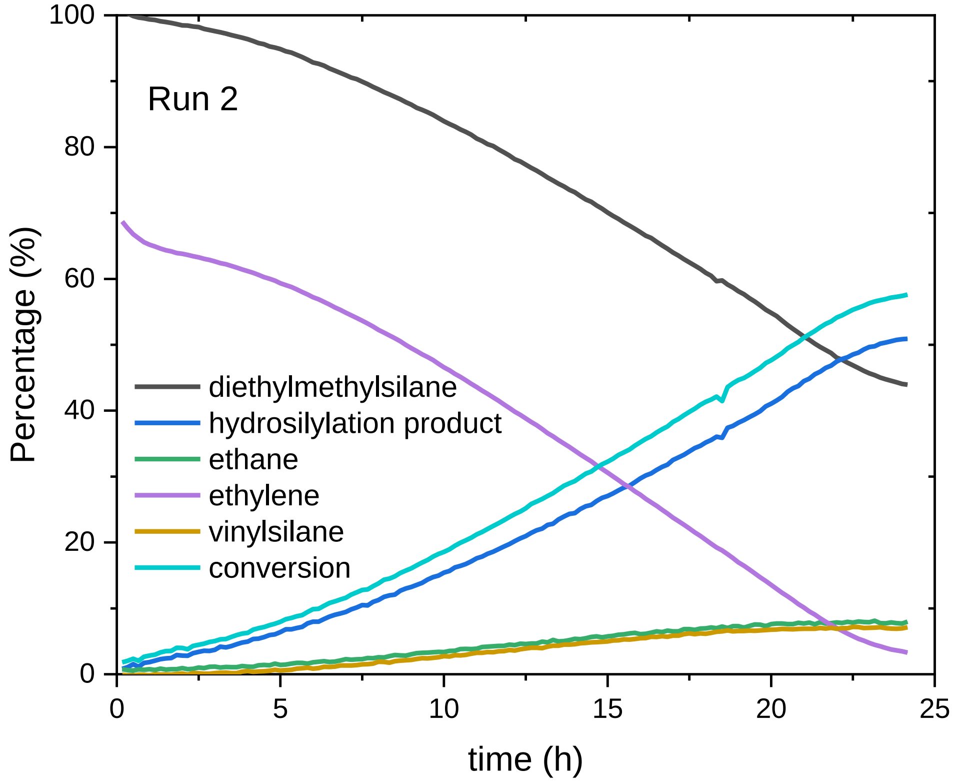

Figure S10. Amount of respective reaction components detected by in situ ^{1}H NMR from ethylene hydrosilylation with Rh(II)-**3**.

7.5 Mass Transfer Limitation in NMR tubes

We observed that the dissolution of ethylene that is present in the headspace of a J-Young NMR tube in C_6D_6 was associated with a substantial kinetic barrier. The cause for the sharp decrease in the reaction rate once a conversion of around 55% was reached could either be a drastic reduction in the activity of the catalyst or the unavailability of one or more reagents. Since we could directly quantify the amount of ethylene present in solution throughout the reaction (purple line in Figure S11), we could conclude that the concentration of ethylene in solution in fact becomes negligibly small after 30 h. The red line in Figure S11 labelled “sum of ethylene” shows the sum of the concentration of ethylene and all ethylene-derived products. The line is almost horizontal until a reaction time of 30 h is reached, and then only rises very slowly, which indicates that ethylene dissolution in the reaction mixture is almost absent at first (< 1%) and remains extremely slow even when all ethylene in solution has been depleted.

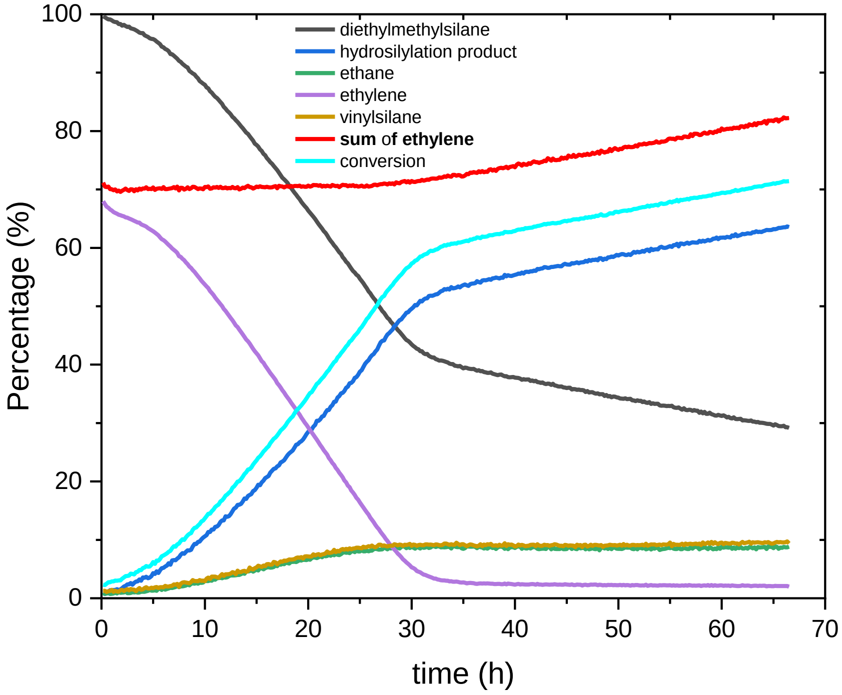

Figure S11. Amount of respective reaction components detected by in situ ^{1}H NMR from ethylene hydrosilylation with Rh(II)-**3**. The sum of ethylene and ethylene-derived reaction products are indicated by a red line.

8 The Oxygen Effect

In our attempts at performing in-situ kinetic experiments for thermal hydrosilylation catalyzed by Rh(II)-**3** we had found that variations in the amount of adventitious O_2 in different reactions could constitute a source of irreproducibility. We thus attempted to diligently remove residual oxygen from C_6D_6 solvent. In our prior work the reaction solvent had only been sparged with argon for 30 minutes prior to use. To evaluate the influence of oxygen we subjected C_6D_6 to several freeze-pump-thaw cycles. Since we had previously observed that Rh(II)-**3** is a very efficient oxygen trap which generates Rh(III)-superoxo (**9**) in the presence of trace oxygen, we came up with a solvent recycling approach to further reduce the oxygen content of freeze-pump-thaw degassed benzene. To this end, we recovered benzene solvent which had been used to activate one batch of Rh(Me)-**3** to Rh(II)-**3** and reused this (further deoxygenated) solvent in additional reactions (see Section 8.2).

8.1 Solvent Recovery Study

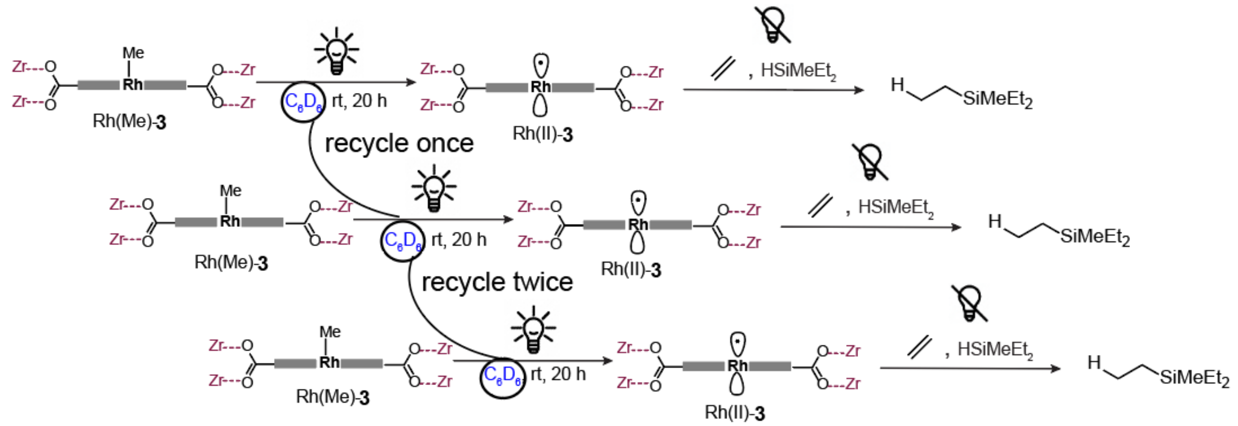

Preparation of the active catalyst: The same experimental procedure as section 2.2.1 was employed, except that Rh(Me)-**3** (10 mg, 7.1 µmol) was used in a 20 mL glass vial and that the C_6D_6 (10.0 mL) used in the reaction had been deoxygenated rigorously via repeated freeze-pump-thaw deaeration.

After the reaction was complete, the reaction vial was subjected to centrifugation and brought into the glovebox to separate the active catalyst and recover the C_6D_6 solvent. Recovered C_6D_6 was used for the preparation of the next batch of Rh(II)-**3**. All batches of the active catalyst thus generated were tested for their efficiency in ethylene hydrosilylation according to the general reaction procedure in section 2.2.2.

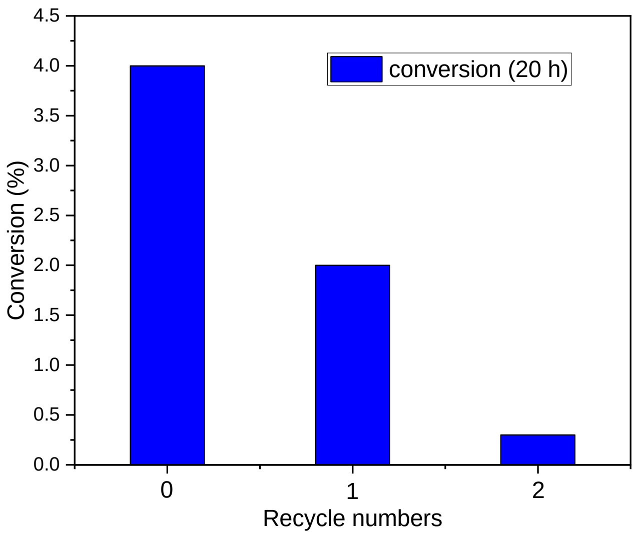

Figure S12. Comparison of the silane conversion observed in thermal ethylene hydrosilylation reactions depending on the number of times the reaction solvent used to generate Rh(II)-**3** had been recycled.

Rh(II)-**3** generated in solvent from the first recycle achieved 2% conversion and Rh(II)-**3** generated in solvent from the second recycle gave only 0.3% conversion (compared to ~20% for Rh(II)-**3** prepared with

benzene that was only sparged with argon) (Figure S12)). We attributed the progressive decrease in the activity of the catalyst that is generated to a steady decrease in the concentration of oxygen in the reaction solvent used for the generation of Rh(II)-**3**. To determine when, under which conditions, and in what amounts, oxygen needs to be present in order to permit the generation of active batches of Rh(II)-**3**, we carried out a number a follow-up experiments (vide infra).

8.2 Effect of Air on Different Reaction Steps

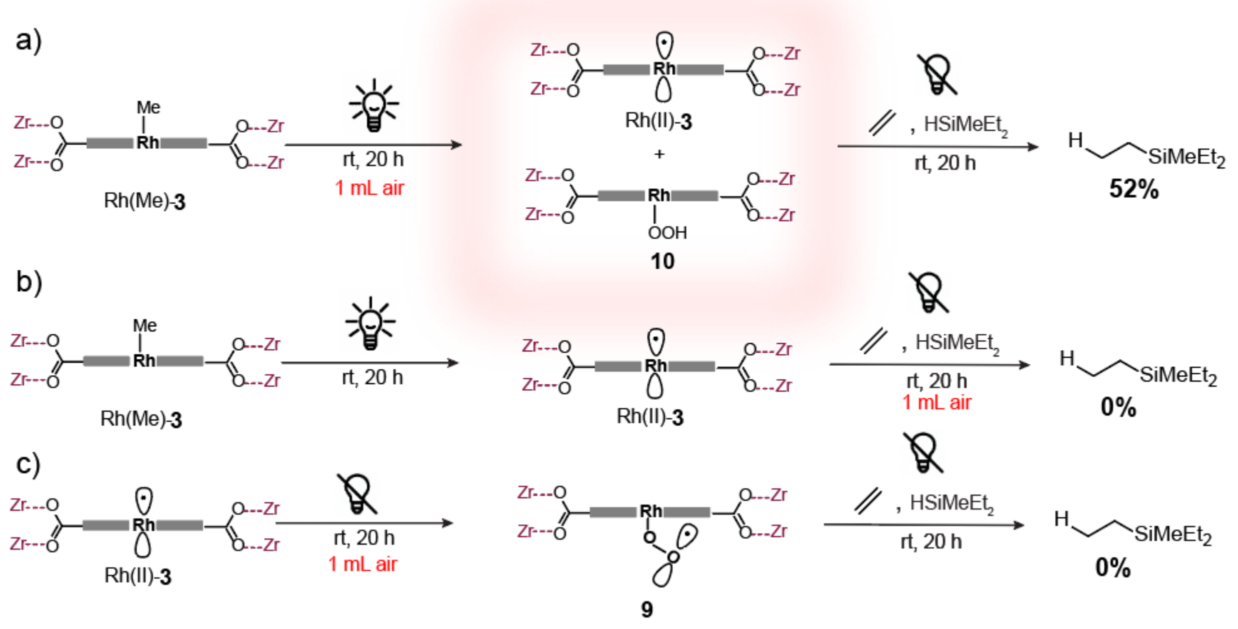

Figure S13. Investigation of the effect of air on catalyst generation for thermal ethylene hydrosilylation.

The presence of small amounts of air during the irradiation of Rh(III)Me-**3** leads to the formation of a catalyst that was active for thermal hydrosilylation (Figure S14a). However, if air was added to an ethylene hydrosilylation reaction catalyzed by a batch of Rh(II)-**3** that was generated in the absence of air, no product could be detected (Figure S14b). Furthermore, when Rh(II)-**3** was generated via photolysis in the absence of air, and the Rh(II)-**3** thus generated was exposed to air for 20 h in the dark, before the air was removed and the resulting catalyst used for ethylene hydrosilylation, no product was observed (Figure S14c). We thus concluded that oxygen and light had to be present simultaneously in order to permit the formation of MOF material that was catalytically competent for thermal olefin hydrosilylation. We could later show that the presence of air during the illumination step generates Rh(III)-O-OH (**10**) alongside Rh(II)-**3**. Experimental evidence for the formation of Rh(III)-O-OH (**10**) is provided in Figure S18, Figure S23 and Figure S24.

To determine the ideal amount of oxygen that should be present during the generation of the active catalyst, further experiments were carried out (vide infra).

8.3 Dependence of Catalyst Activity on Oxygen Concentration

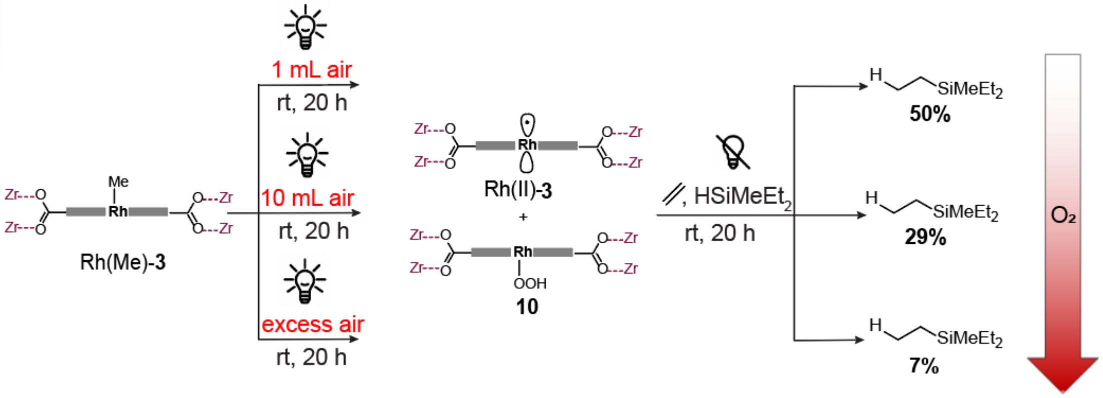

Figure S14. The presence of limited amounts of air during the formation of Rh(II)-**3** from Rh(III)Me-**3** leads to the formation of a catalyst that is active in thermal hydrosilylation.

The active Rh(II) catalyst was prepared as follows: In an argon-filled glovebox, Rh(Me)-**3** (1.0 mg, 0.71 µmol, 0.34 mol%) and C_6D_6 (1.0 mL), were added to a 9 mL glass tube containing a cross-type stir bar (5 x 10 mm), then the vial was sealed with screw cap that contains a septum, and transferred out of the glovebox. 1 ml or 10 mL of air was injected into different batches accordingly (for excess air batch, the headspace of vial was connected to the atmosphere vial a needle). The reaction mixture was subjected to sonication for 1 minute in order to evenly suspend the MOF crystallites throughout the solvent. After that, Rh(Me)-**3** was subjected to photolysis to produce active catalyst Rh(II)-**3** with a 390 nm purple LED for 20 h at room temperature and a stirring rate of 1000 rpm.

The activity of different batches of catalyst for ethylene hydrosilylation were subsequently evaluated via experiments set up according to the standard procedure for thermal ethylene hydrosilylation.

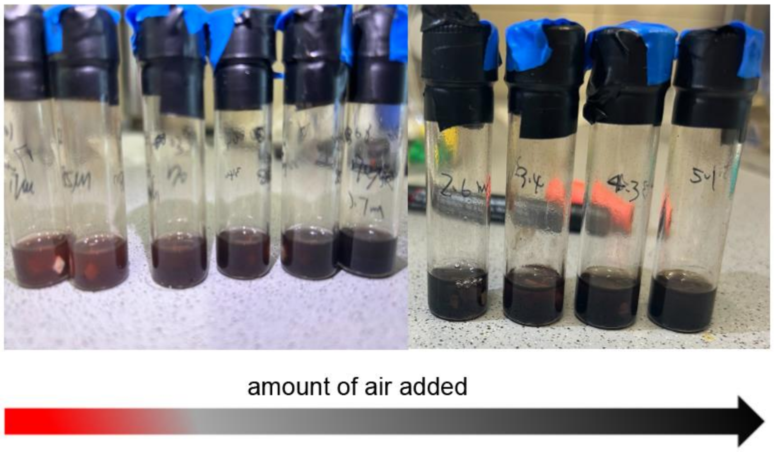

Figure S15. The color of the MOF changes from red to gray and finally to black after illumination in the presence of increasing amounts of air. From left to right, different amounts of air were added to each vial, ranging from 0.017 mL to 5.1 mL, in the following order: 0.017 mL, 0.085 mL, 0.17 mL, 0.34 mL, 0.85 mL, 1.7 mL, 3.4 mL, 4.3 mL, and 5.1 mL (Figure S15).

In the preceding experiments, undried air was added to the reaction vessels. In order to confirm that the effect observed indeed resulted from the presence of O_2 during the generation of Rh(II) rather than from the addition of water or any other components present in unpurified air, we carried out two reactions in which pure dry O_2 was added instead of air. We had previously calculated the amount of O_2 contained in the amount of air added to the reaction vessels and established a correlation between amount of O_2 added and performance of the resulting catalyst. Here we choose two representative O_2 amounts for which to repeat the experiments with pure dry O_2 (Figure S16). For batch 1 we added 39.4 µL O_2 which is equivalent to the amount of O_2 present in 0.17 mL air. For batch 2 we added 0.6 mL O_2 which is equivalent to the amount of O_2 present in 2.6 mL air. (Figure S16). Close agreement between the results obtained with dry oxygen and non-dried air indicated that the "air effect" was indeed an oxygen effect.

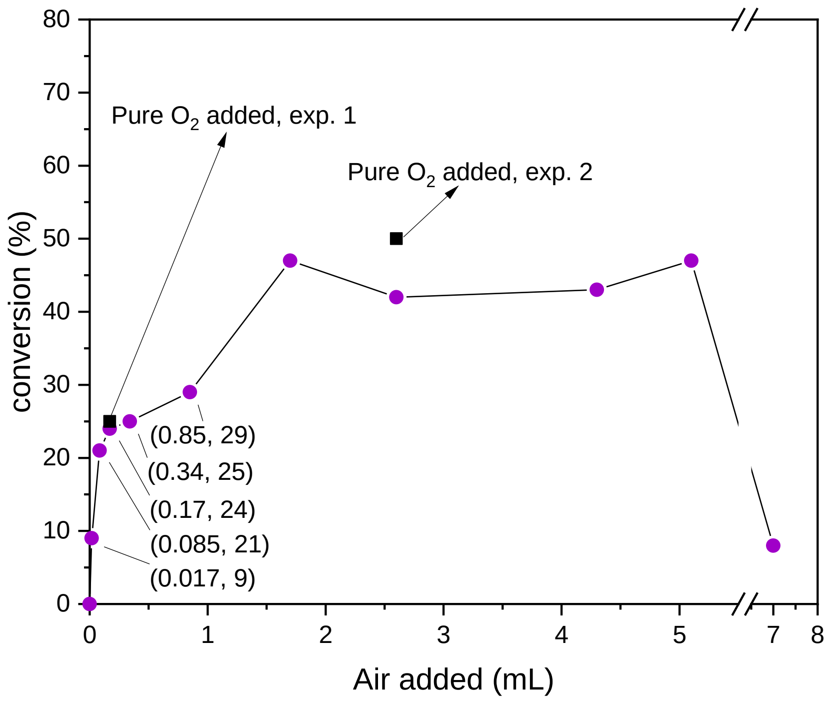

Figure S16. Comparison of the conversion obtained in ethylene hydrosilylation when controlled amounts of dry oxygen were added during the generation of the active catalyst (black data points) with the effect of adding controlled amounts undried air during the generation of the active catalyst (purple data points). Data point labels are provided for selected data points.

8.4 Mechanistic Insight into the Oxygen Effect

We had determined that the formation of batches of Rh(II)-**3** that are active for thermal hydrosilylation required the presence of limited quantities of oxygen during the irradiation of Rh(III)Me-**3** (which gives rise to Rh(II)-**3**). Notably, oxygen and light have to be present simultaneously for catalytically active material to be formed. If Rh(II)-**3** is quantitatively generated through irradiation and is then exposed to limited amounts of oxygen in the dark, the resulting catalyst is inactive. Likewise, exposure of Rh(III)Me-**3** to oxygen for 12 h in the dark, followed by removal of oxygen and then irradiation does not give rise to active catalyst. Light is required to generate Rh(II)-**3** from Rh(III)Me-**3**, and Rh(II)-**3** rapidly generates Rh(III)-superoxide **9** in the presence of air (no light is required for the conversion of Rh(II)-**3** to Rh(III)-superoxide **9**). In the presence of light, re-formation of Rh(II)-**3** from Rh(III)-superoxide **9** is possible ***(Figure 6).*** Rh(III)-superoxide **9** itself does not promote thermal hydrosilylation of olefins. MOF that contains a mixture of Rh(II)-**3** and Rh(III)-superoxide **9** (prepared by exposing Rh(II)-**3** prepared in the absence of oxygen to controlled amounts of air) does not promote thermal hydrosilylation of olefins. We proposed that the need for the simultaneous presence of light and O_2 to generate catalytically active material could be rationalized by the formation of Rh(III)-hydroperoxide **10** from Rh(III)-superoxide **9** through HAT reaction with the benzene solvent.

An IR spectrum of active catalyst (generated in the presence of 1 mL air) shows a band that can be attributed to the O-O stretch of a metal-bound hydroperoxide, while this band was not observed for inactive catalyst that was generated in the absence of air (Figure S18). Formation of **10** from **9** in the presence of benzene is expected to be an endergonic process, but it is facilitated by the presence of benzene in solvent quantities. To determine whether HAT between **9** and benzene to form **10** is itself a light-mediated process, we carried out a number of IR experiments. Unlike EPR spectroscopy, which can detect the formation of paramagnetic **9** but not diamagnetic **10**, O-O stretches corresponding to both **9** and **10** can be observed by IR. For a sample of dry Rh(II)-**3** that was exposed to O_2 in the absence of light, only superoxide formation (O-O stretch at 1250 cm^{-1}) could be observed. However, a sample of Rh(II)-**3** that was exposed to O_2 in the presence of benzene showed the presence of hydroperoxide (O-O stretch at 813 cm^{-1}) in addition to superoxide formation (O-O stretch at 1250 cm^{-1}, Figure S19). Since formation of hydroperoxide **10** was observed in the absence of light, irradiation is not required to enable HAT with benzene solvent. HAT from benzene to **9** generates phenyl radicals that are expected to undergo swift follow-up reaction with surrounding solvent molecules: expected reaction products such as biphenyl could be detected in solution (Figure S23 and Figure S24).

We attribute the need for the simultaneous presence of both air and light to generate the active catalyst to the fact that formation of **9** from Rh(II)-**3** is reversible in the presence of light but irreversible in the absence of light. We propose that Rh(II)-**3** is the active site at which the transition state for olefin hydrosilylation is assembled and that the presence of small amounts of **10** within the MOF pores provide crucial stabilization to the transition state structure. The conversion of **9** to **10** is an inefficient process, so if formation of **10** is attempted in the absence of light, it either leads to the formation of extremely small amounts of **10** (low oxygen content) or the conversion of the vast majority of Rh(II)-**3** to **9** (high oxygen content). If the synthesis of Rh(II)-**3** from Rh(III)Me-**3** via light-mediated Rh–C cleavage is directly carried out in the presence of limited amounts of O_2, an appreciable concentration of **9** is built up initially (the conversion of Rh(II)-**3** to **9** is essentially instantaneous) from which **10** can be formed. Irradiation renders the formation of **9** reversible, however, so that some of the remaining **9** reforms Rh(II)-**3**. We speculate that over the course of several hours the oxygen concentration in the reaction vial gradually decreases

because O_2 is partially consumed by e.g. reaction with phenyl radicals generated in the formation of **10** or methyl radicals generated in the conversion of Rh(III)Me-**3** to Rh(II)-**3**. Light-mediated synthesis in the presence of limited amounts of air is thus able to achieve the requisite balance between the amounts of Rh(II)-**3**, **9** and **10** in the sample that are required for it to function as an effective catalyst. Figure S31 confirms the simultaneous presence of Rh(II)-**3** and **9** (**10** is diamagnetic and thus not visible in EPR spectroscopy) for a catalyst sample prepared in the presence of 1 mL air while Figure S18 confirms the presence of both **9** and **10** in the sample (Rh(II)-**3** does not have a characteristic vibration that can be distinguished by IR spectroscopy). Additional data supporting the outlined mechanistic proposal are presented from Figure S17 to Figure S22.

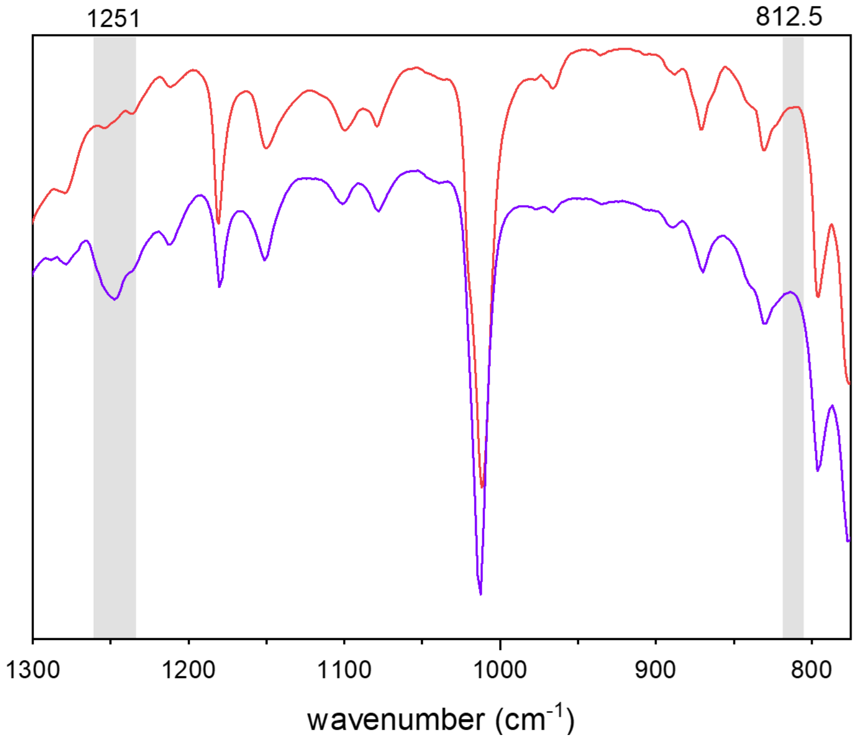

Figure S17. Comparison of FT-IR spectra of Rh(II)-**3** generated in the absence of air (red trace) and Rh(II)-**3** generated in the absence of air and then exposed to air (purple trace). Upon exposure to air, Rh(III)-superoxide **9** is generated which gives rise to a band at 1251 cm^{-1} for the O-O stretching vibration. No change was observed in the area of 812.5 cm^{-1} after dry Rh(II)-**3** was exposed to air. Both spectra were collected in an argon-filled glovebox.

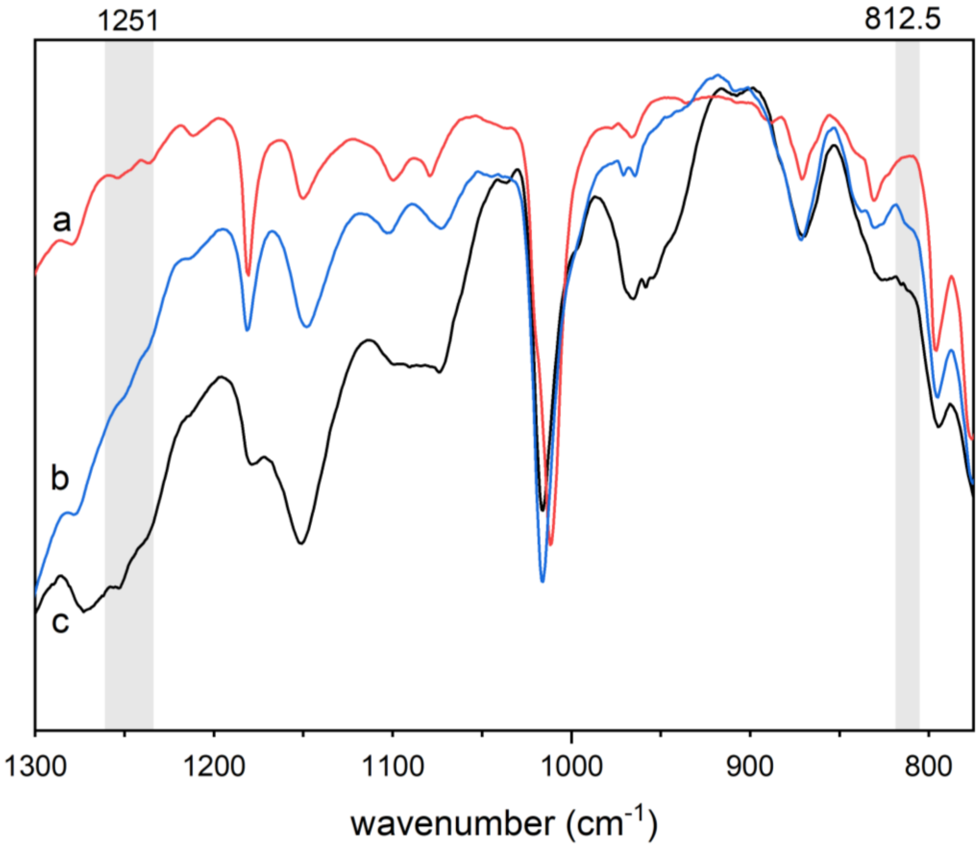

Figure S18. Comparison of FT-IR spectra collected of batches of Rh(II)-**3** generated in the absence of air (**a**), in the presence of 1 mL air (**b**), or in the presence of excess air (**c**) in benzene-d_6. The band at 813 cm^{-1} is in good agreement with the reported O-O stretching vibration of a rhodium-bound hydroperoxide.[63] For (**a**) which corresponds to an inactive sample, no clear band could be observed at either 813 cm^{-1} or 1251 cm^{-1}. For (**b**), which corresponds to the active catalyst, a small band at 813 cm^{-1} is visible indicating the presence of **9** in addition to a weak band at 1251 cm^{-1}. For (**c**), which corresponds to catalyst with poor performance in hydrosilylation, a band at 813 cm^{-1} was observed in addition to a strong band at 1251 cm^{-1}, which indicates the presence of large amounts of **10**. All three spectra were collected in an argon-filled glovebox.

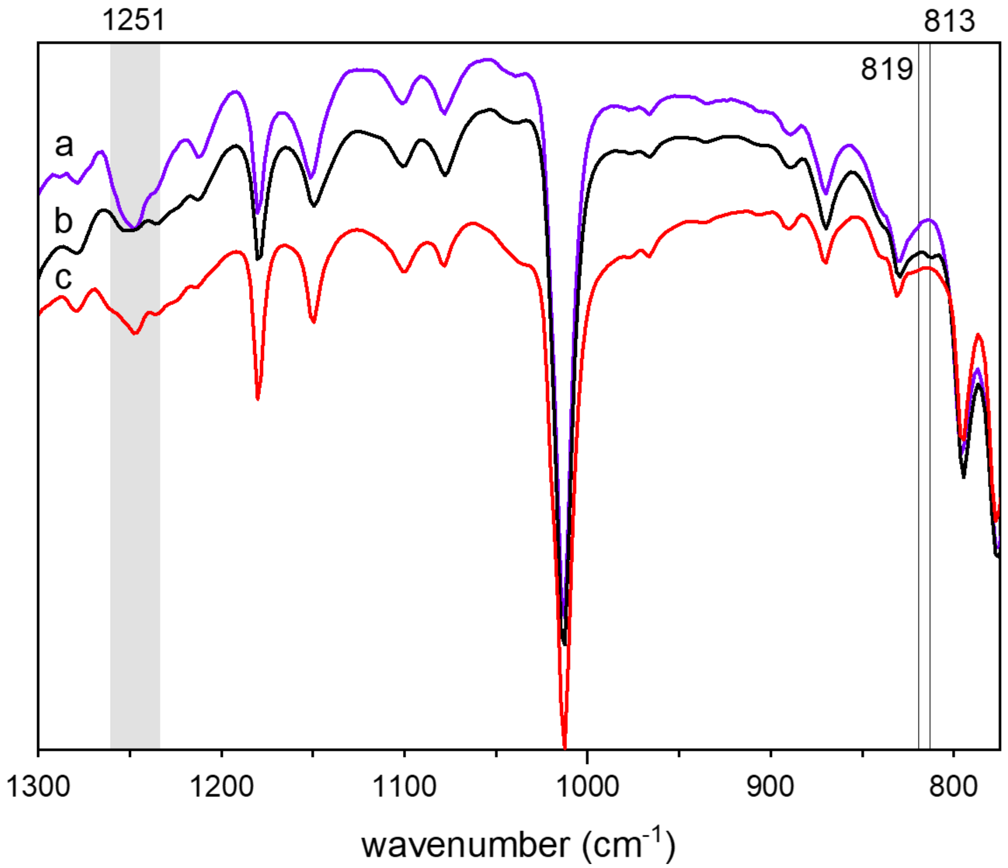

Figure S19. Comparison of FT-IR spectra of Rh(II)-**3** exposed to air either in the absence of solvent (**a**), in benzene-d_6 (**b**) or in benzene (**c**). For (**a**), a clear band corresponding to rhodium superoxide **9** is visible while rhodium hydroperoxide is not detected. For (**b**) and (**c**) the band corresponding to superoxide **9** is less intense while a band we attribute to rhodium hydroperoxide (at 812.5 cm^{-1} for D-**10** in (**b**) and at 819 cm^{-1} for **10** in (**c**)) was observed. Since all three experiments were carried out in the dark, the conversion of **9** to **10** via HAT with benzene can thus take place at room temperature in the absence of light.

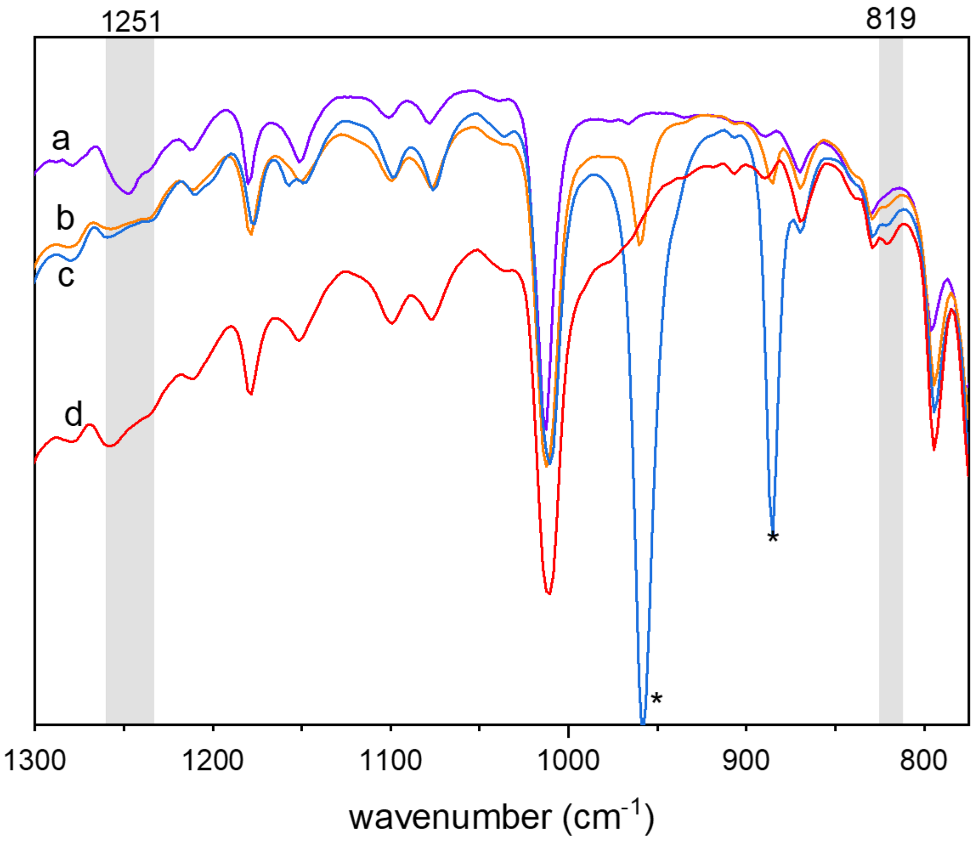

Figure S20. Comparison of the FT-IR spectra of a sample of **9** before (**a**) and after (**b-d**) increased exposure to cyclohexadiene. To obtain additional evidence for the formation of **10** via hydrogen atom transfer to **9**, we treated a sample of **9** (**a**) with cyclohexadiene, which is competent hydrogen atom donor due to the presence of a C–H bond with a BDE of only 76 kcal·mol^{-1}. Spectrum (**b**) and (**c**) were collected immediately after the addition of one or two drops of cyclohexadiene, respectively. Spectrum **d** was collected 15 minutes after spectrum (**c**) with no further cyclohexadiene addition. The rapid appearance of a band at 819 cm^{-1} in addition to a decrease in the intensity of the band at 1251 cm^{-1} after the addition of cyclohexadiene confirms that HAT between **9** and cyclohexadiene is rapid. *bands attributable to cyclohexadiene.

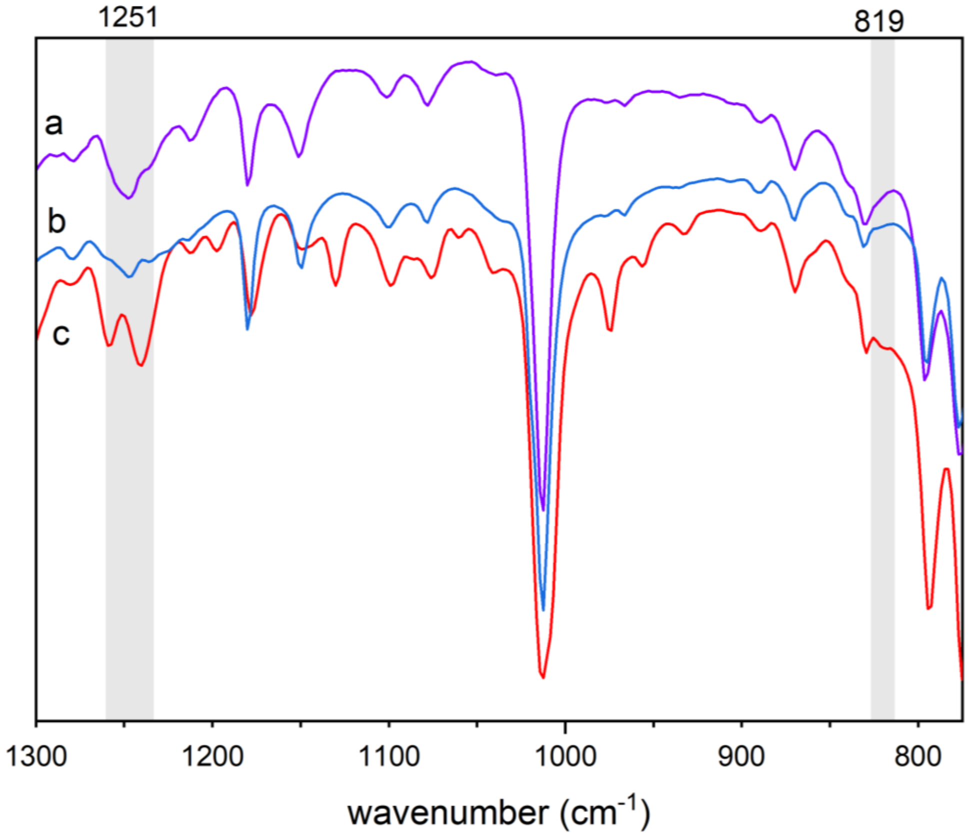

Figure S21. Comparison of the FT-IR spectra of a sample of **9** (**a**), a sample of **9** exposed to benzene (**b**) and a sample of **9** exposed first to benzene and then to TEMPOH (**c**). Upon addition of the competent H-atom donor TEMPOH (O–H BDE of 76 kcal·mol^{-1}) an increase in the intensity of the band at 819 cm^{-1} could be observed.

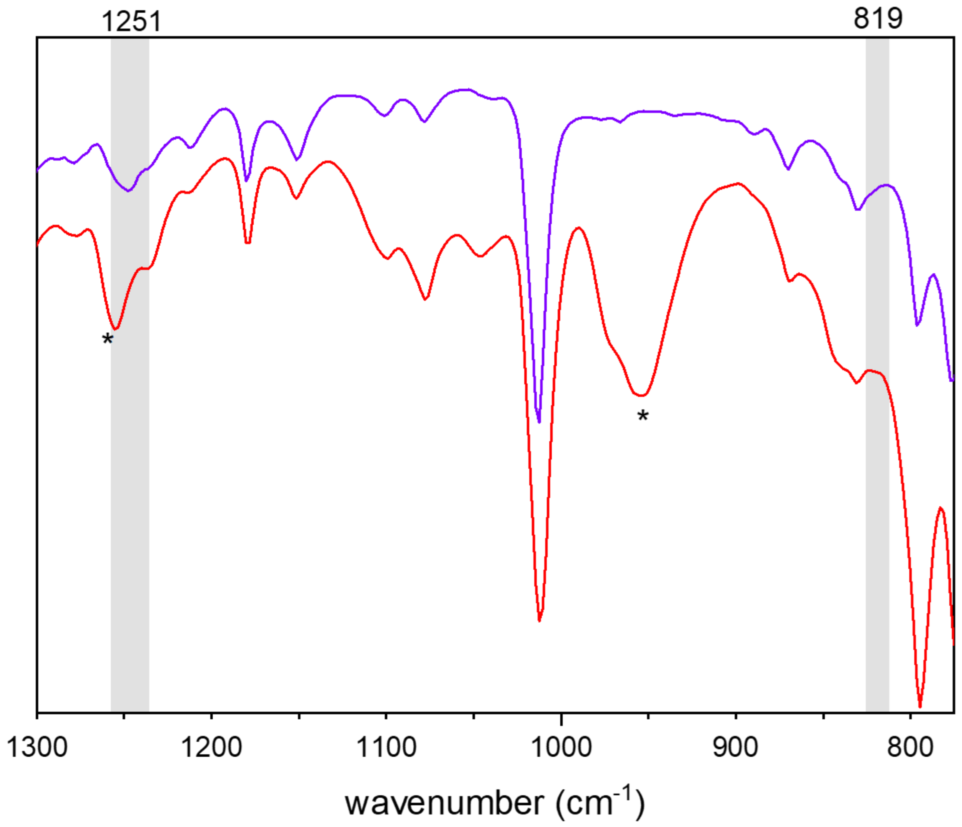

Figure S22. Comparison of the FT-IR spectra of a sample of **9** before (purple trace) and after (red trace) treatment with solvent quantities of Et_2MeSiH. Since a residual amount of **9** is present in samples of active catalyst that are added to the reaction mixture for olefin hydrosilylation we tested whether HAT between **9** and Et_2MeSiH was able to generate silyl radicals and **10**. While a weak band corresponding to **10** was observed, HAT with Et_2MeSiH appeared to proceed with comparable to lower efficiency than HAT with benzene. Notably, benzene is present in solvent quantities in the reaction mixture used for catalytic transformations while Et_2MeSiH is only present in stoichiometric amounts. We thus concluded that the residual amount of **9** present in samples of the active catalyst could only lead to the generation of silyl radicals in very minor amounts. Notably, solid-state NMR data and a double label experiment described in the main text also support the presence of very minor amounts of silyl radicals during thermal hydrosilylation reactions. We suppose that HAT with a fraction of the residual **9** present in the catalyst sample is the source of the minor amounts of silyl radicals that could be detected. *bands attributable to Et_2MeSiH.

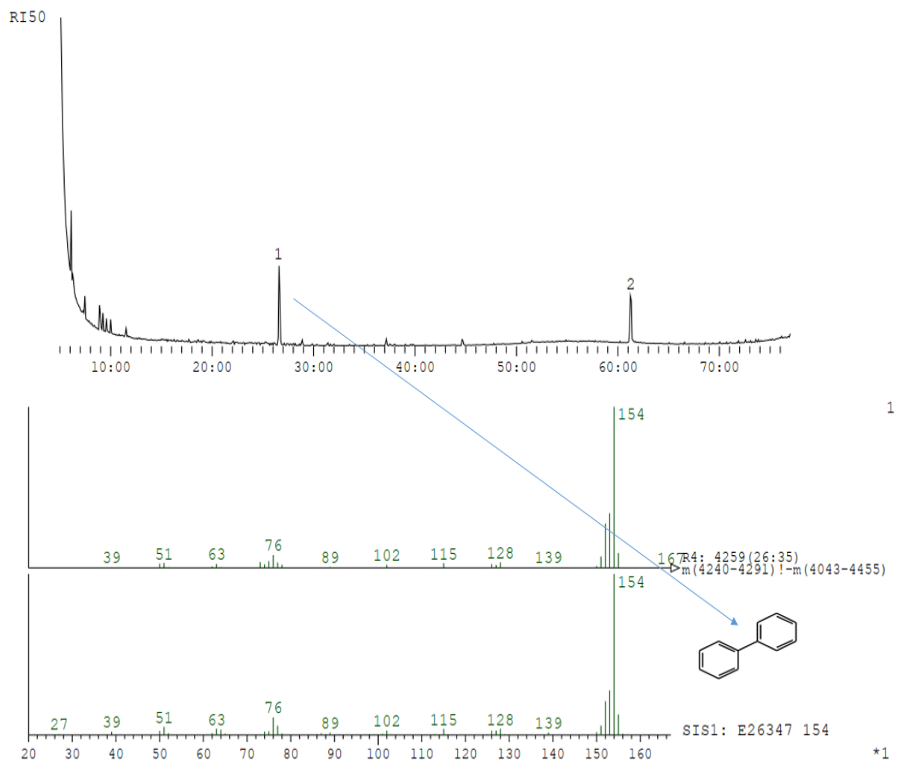

Figure S23. Biphenyl was detected by GC-MS in the liquid phase recovered from the reaction mixture in which Rh(III)Me-**3** was converted to Rh(II)-**3** in the presence of O_2 using C_6H_6 as the solvent.

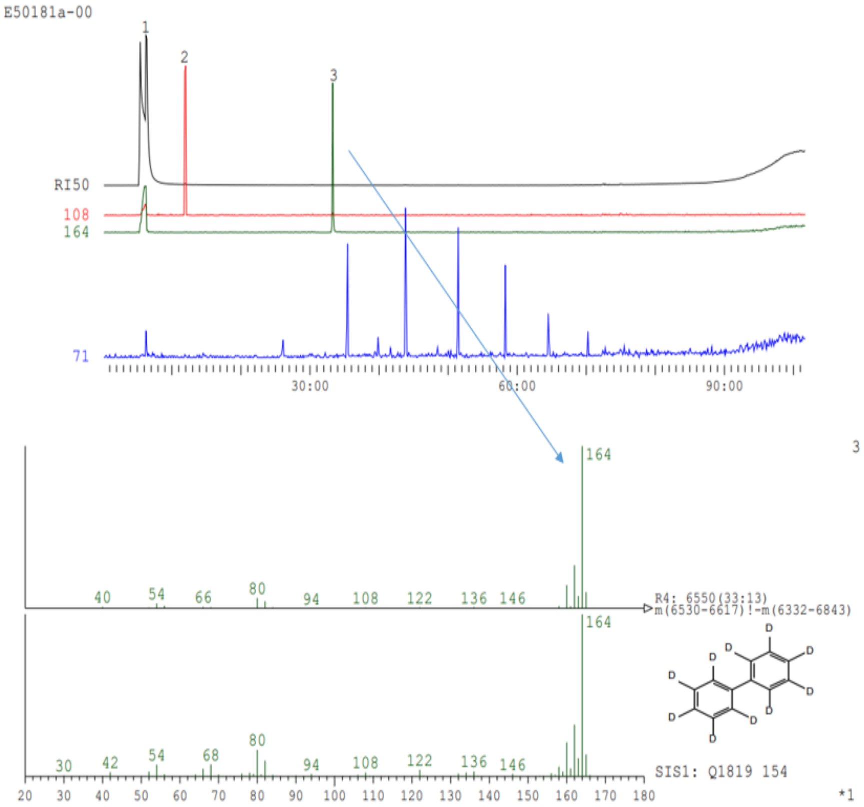

Figure S24. D_{10}-biphenyl was detected by GC-MS in the liquid phase recovered from the reaction mixture in which Rh(III)Me-**3** was converted to Rh(II)-**3** in the presence of O_2 using C_6D_6 as the solvent.

9 EPR Spectroscopy

Continuous wave (CW) X-band (~9.5 GHz) EPR spectra were measured at 77 K and 298 K employing a Bruker Elexsys E500 spectrometer equipped with a ER4116DM resonator and an Oxford ESR900 cryostat. Irradiation of the samples was always performed *ex-situ*. After every treatment, the reaction mixture was first transferred into a glovebox, and then loaded into an EPR tube in order to prevent oxygen contamination during transfer. EPR tubes were then taken out of the glovebox and immediately frozen in liquid nitrogen. Additional X-band CW-EPR spectra were recorded by using a MS-5000 (Magnettech GmbH, Freiberg Instruments) operating from 93 K up to room temperature (RT).

Q-band (~33.5 GHz) pulse and CW EPR experiments were measured by using a Bruker Elexsys E580 spectrometer with a SuperQ-FT microwave bridge and a home-built resonator described earlier.[64] Cryogenic temperatures (8 K) were obtained by an Oxford CF935 flow cryostat.

Hyperfine sublevel correlation spectroscopy (HYSCORE)[65] experiments were performed to extract the ^{14}N and ^{2}H hyperfine and quadrupole interactions using the standard HYSCORE pulse sequence: $\pi/2–\tau–\pi/2–t_1–\pi–t_2–\pi/2–\tau–$(echo). The length of the microwave $\pi/2$ and π pulses was set to 20 ns and 40 ns, respectively. The delay between the first two pulses (τ) was adjusted in order to avoid "blind spots" in the

region of the spectra corresponding to the Larmor frequency of ^{2}H at specific magnetic field. The starting t_1 and t_2 delays in all measurements were 16 ns. To suppress the effect of unwanted echoes, a four-step phase cycling of the microwave pulses was used.

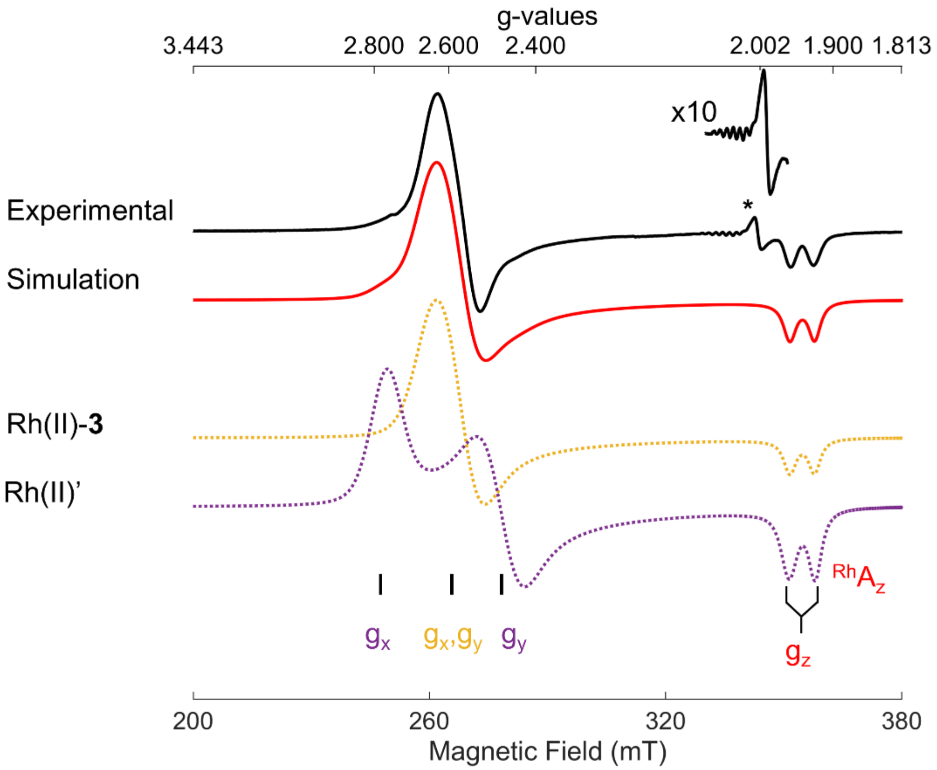

Figure S25. Experimental **s**pectrum (black) and computer simulation (red) of the CW-EPR spectrum of Rh(II)-3 measured at 77 K after irradiation for 20 hours. The simulation is composed of the weighted contributions of two Rh species shown in yellow and violet dotted traces. A zoom of the metal-free porphyrin radical signal is also shown, which arises due to the use of small amounts of rhodium-free PCN-224 seed crystals during the synthesis of the rhodium-containing PCN-224 MOF (Rh(III)Me-**3**). The asterisks indicate $RhOO^{\bullet}$ species generated due to O_2 contamination.

Table S5. Spin Hamiltonian parameters employed for the simulation of the CW-EPR spectrum reported of Rh(II)-**3**. A comparison with calculated values is also reported. The DFT results agree with the experimental ones, but only when the two benzene molecules closest to the Rh center are added. For the theoretical level used in the EPR calculations, see the Computational Details section 18.

Species	Weight	g_x	g_y	g_z	A_x (MHz)	A_y (MHz)	A_z (MHz)
Rh(II)-**3**	85 %	2.601(2)	2.601(2)	1.942(2)	Not resolved	Not resolved	170(4)
Rh(II)'	15 %	2.765(2)	2.475(2)	1.942(2)	Not resolved	Not resolved	170(4)
Rh(II)-**3**-$(C_6H_6)_2$ (DFT) [a]		2.431	2.421	1.998	-155	-156	-169
Rh(II)-**3** (DFT) [b]		3.755	3.624	2.001	-213	-334	-351

[a] Two explicit benzene molecules were included in the calculation, one above the Rh center and one below the Rh center.

[b] No explicit benzene molecules were added in the calculation.

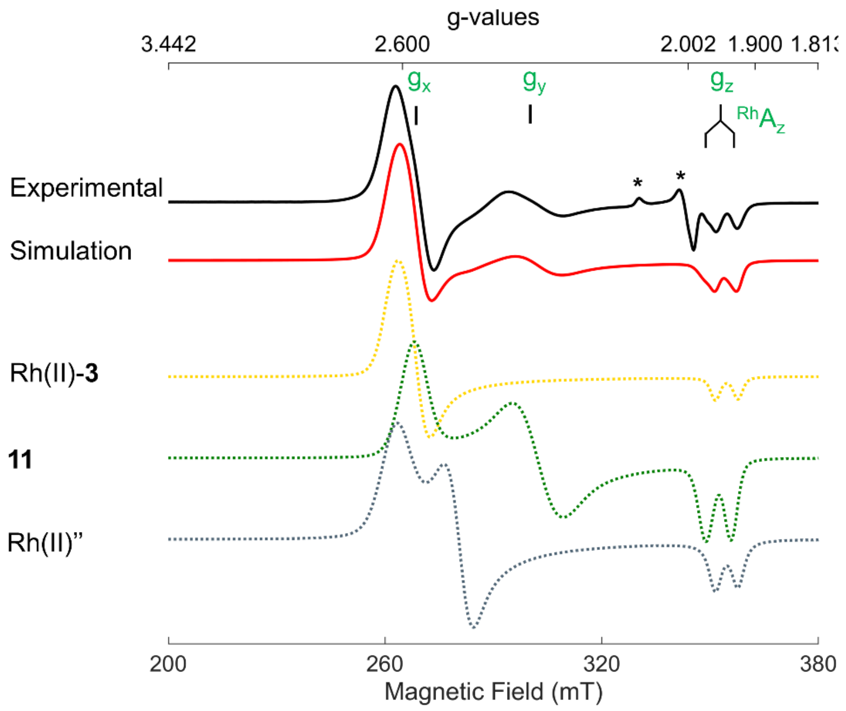

Figure S26. Experimental spectrum (black) and computer simulation (red) of the CW-EPR spectrum of Rh(II)-**3** + ethylene measured at 77 K. The simulation is composed by the weighted contribution of three Rh species shown in yellow, green and gray dotted traces. The asterisks indicate RhOO• species generated due to O_2 contamination.

Table S6. Spin Hamiltonian parameters employed for the simulation of the CW-EPR spectrum reported of Rh(II)-**3** in presence of ethylene. The Rh-ethylene adduct is identified in species **11**. A comparison of the spin Hamiltonian parameters relative to such species with calculated values is reported.

Species	Weight	g_x	g_y	g_z	A_x (MHz)	A_y (MHz)	A_z (MHz)
Rh(II)-**3**	55 %	2.590(2)	2.590(2)	1.942(2)	150.0(20)	150.0(20)	170.0(5)
**11**	40 %	2.570(5)	2.277(2)	1.955(2)	80.0(10)	60.0(10)	190.0(5)
Rh(II)''	5 %	2.620(2)	2.455(2)	1.942(2)	82.0(10)	80.0(10)	170.0(5)
**11**-C_6H_6 (DFT) [a]	/	2.286	2.220	1.999	-83	-92	-123
**11**-C_6H_6 (DFT) [a, b]	/	2.377	2.326	1.999	-123	-129	-148

[a] One explicit benzene molecule was added in the calculation, at the *trans* position of the ethylene ligand.

[b] The Rh-ethylene distance was increased by 0.2 Å with respect to the equilibrium distance.

The computational underestimation of the *g*-value anisotropy of **11** may be due to over-binding of ethylene at the level of theory used for geometry optimization (BP86-D3/SARC-ZORA-TZVP(Rh)/ZORA-def2-TZVP(Si)/ZORA-def2-SVP(C,H,O,N)). Increasing the Rh-ethylene distance (defined as the distance between Rh and the center of the ethylene C=C bond) by 0.2 Å leads to a slight decrease of the energy computed at the level of theory used for EPR calculations (ωB97M-V/x2c-TZVPPall-2c(decontracted)), and better agreement of the EPR parameters with respect to experiment.

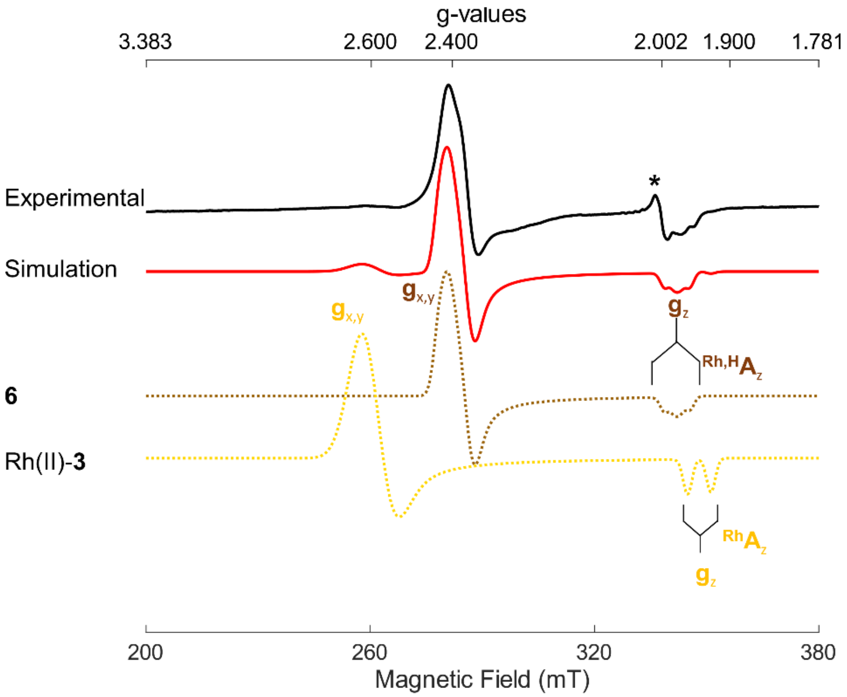

Figure S27. Experimental (black) and computer simulation (red) of the CW-EPR spectrum of Rh(II)-**3** + silane measured at 77 K. The simulation is composed by the weighted contribution of two Rh species shown in yellow and brown dotted traces. The asterisks indicate RhOO• species generated due to O_2 contamination**.**

Table S7. Spin Hamiltonian parameters employed for the simulation of the CW-EPR spectrum reported of Rh(II)-**3** in presence of silane. A comparison of the spin Hamiltonian parameters relative to such species with calculated values is reported.

**Species**	**Weight**	g_x	g_y	g_z		A_x **(MHz)**	A_y **(MHz)**	A_z **(MHz)**
Rh(II)-**3**	10 %	2.601(2)	2.601(2)	1.942(2)	^{103}Rh	Not resolved	Not resolved	170 (4)
**6**	90 %	2.419(5)	2.370(2)	1.978(3)	^{103}Rh	60(20)	60 (20)	120 (5)

				^{1}H	Not resolved	Not resolved	70 (10)
**6**-C_6H_6 (DFT) [a]	2.274	2.262	1.998	^{103}Rh	-96	-98	-127
				^{1}H	-17	-20	48

[a] One explicit benzene molecule is added in the calculation, at the *trans* position of the silane ligand.

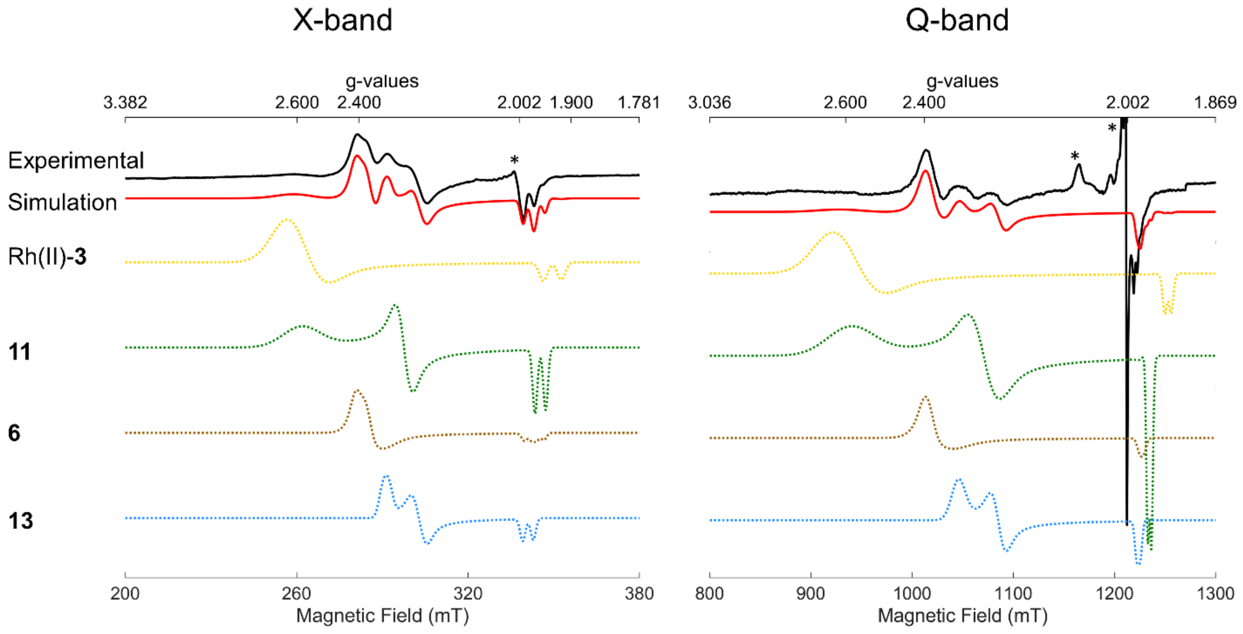

Figure S28. Experimental (black) and computer simulation (red) of the CW-EPR spectrum of Rh(II)-**3** + silane + ethylene measured at X-band (on the left) and Q-band (on the right) at 77 K. The simulations are composed by the weighted contribution of four Rh species shown in yellow, green, brown and cyan dotted traces. The spin parameters employed for the simulations simultaneously fit both the X-band and Q-band EPR spectra. The asterisk indicates RhOO$^{\bullet}$ (**9**) generated due to O_2 contamination.

Table S8. Spin Hamiltonian parameters employed for the simulation of the CW-EPR spectrum reported of Rh(II)-**3** in presence of silane and ethylene. A comparison of the spin Hamiltonian parameters relative to such species with calculated values is reported.

Species	Weight	g_x	g_y	g_z		A_x (MHz)	A_y (MHz)	A_z (MHz)
Rh(II)-**3**	5(1) %	2.601(2)	2.601(2)	1.942(2)	^{103}Rh	Not resolved	Not resolved	170 (4)
**11**	20(5) %	2.590(5)	2.275(2)	1.960(3)	^{103}Rh	Not resolved	60(20)	102(5)

**6**	40(5) %	2.390(5)	2.370(2)	1.972(3)	^{103}Rh	110(20)	Not resolved	120(5)
					^{1}H	Not resolved	Not resolved	60(10)
**13**	35(5) %	2.322(5)	2.233(5)	1.984(3)	^{103}Rh	60(10)	50(10)	102(10)
**13** (DFT)		2.227	2.192	1.998	^{103}Rh	-72	-76	-111
					^{1}H	-29	-31	33

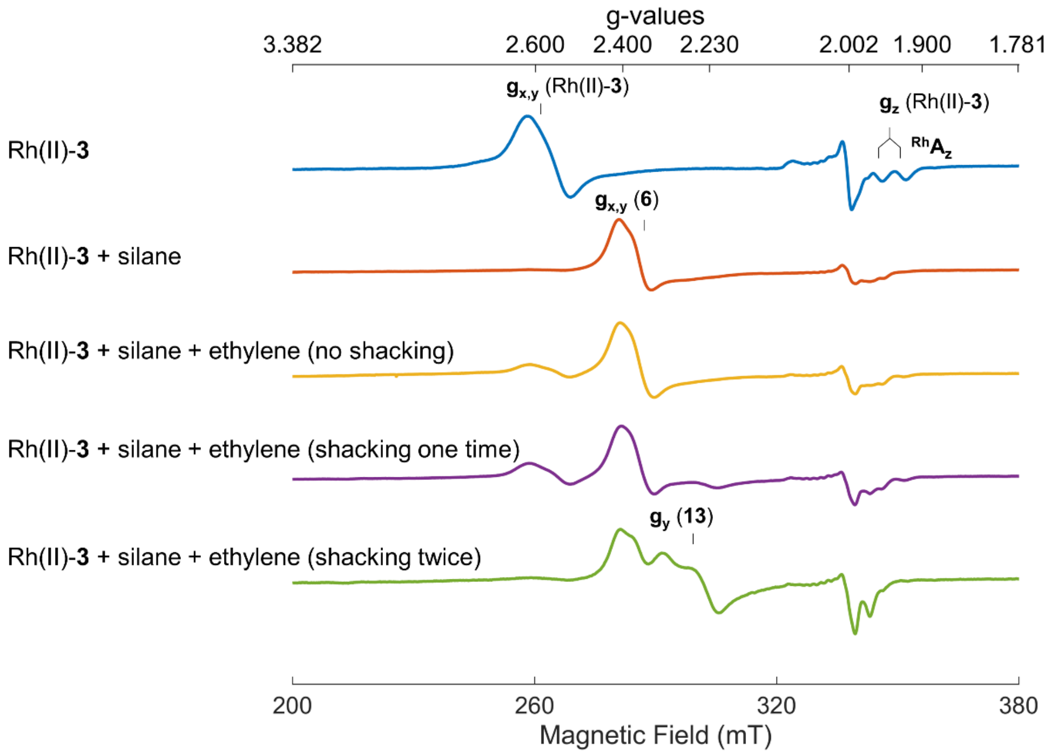

Figure S29. Comparison of experimental CW-EPR spectrum of Rh(II)-**3**, Rh(II)-**3**+silane and Rh(II)-**3**+silane+ethylene recorded at 93 K. Particular focus is put on the ethylene diffusion difficulties inside the EPR tube. The appearance of the signal attributable to **13** occurs only after repeated shaking of the EPR tube.

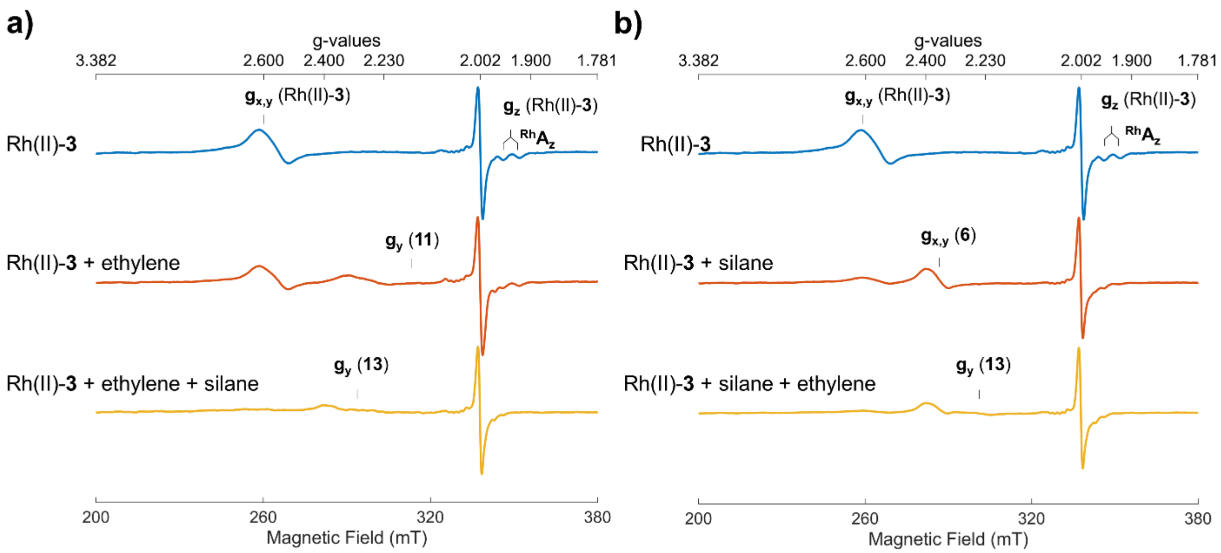

Figure S30. Comparison of experimental CW-EPR spectrum of Rh(II)-**3** illuminated in presence of 1 mL of air and its corresponding adducts with silane and/or ethylene. a) first ethylene and then silane was added and b) first silane and then ethylene was added.

To exclude the possibility that the new EPR signal observed in the presence of both silane and ethylene appeared due to O_2 or H_2O contamination, we have also computed the EPR parameters of multiple alternative structures, where the Rh center bears one O_2 or H_2O ligand and optionally another ligand (ethylene or $HSiMeEt_2$). For Rh complexes with only one ligand, a benzene molecule was added as the second axial ligand. As shown in **Table S9**, all species with O_2 coordination on Rh give *g*-values close to 2 with negligible anisotropy, regardless of the ligand *trans* to O_2, which is a consequence of the radical being mainly localized on the far oxygen atom (Rh-O-O·). Therefore, the change of the signals from Rh(II)-**3** to **11** cannot be due to species with coordinated O_2 ligands. The H_2O ligands reduce the *g*-anisotropy of the Rh(II), Rh-silane and Rh-ethylene complexes, and since the calculated *g*-anisotropy of the H_2O-free species are already systematically smaller than the experimental ones, we conclude that *trans*-H_2O coordination does not explain the **13** signal better than if *trans*-H_2O coordination is not present.

Table S9. Calculated spin Hamiltonian parameters of adducts of Rh(II)-**3** and O_2 or H_2O ligands.

Species	g_x	g_y	g_z		A_x (MHz)	A_y (MHz)	A_z (MHz)
Rh-O-O• (**9**)	2.048	1.990	1.938	^{103}Rh	34	35	36
Rh-OH$_2$	2.383	2.372	1.998	^{103}Rh	-128	-129	-150
Silane-Rh-O-O•	2.050	2.008	1.986	^{103}Rh	9	11	13
				^{1}H	-3	-3	-5

Silane-Rh-OH_2	2.195	2.193	1.995	^{103}Rh	-78	-78	-115
				^{1}H (Rh-H-Si)	-12	-13	58
				^{1}H (H_2O)	-5	6	-11
					-5	6	12
Ethylene-Rh-O_2	2.067	2.005	1.986	^{103}Rh	9	10	13
				^{1}H	0	0	2
					0	-1	2
					0	1	2
					0	0	1
Ethylene-Rh-OH_2	2.278	2.211	1.999	^{103}Rh	-75	-85	-117
				^{1}H (H_2O)	-3	5	-8
					-2	6	-10
				^{1}H (C_2H_4)	4	-5	-9
					4	-5	-9
					-4	5	-7
					-4	5	-7

Two other species that can possibly form when adding silane or ethylene to Rh(II)-**3** are the bis-adducts of silane or ethylene, respectively. Their calculated EPR parameters are shown in Table S10. As expected, their *g*-values differ noticeably from the corresponding mono-adducts, and also deviate significantly from 2.0 (so their signals should not overlap extensively with the signal of Rh-O-O • **9**). Therefore, if they are present in observable quantities, they should be visible on the EPR spectrum (Fig. 7) as separate signals. Since no such signals were found, we conclude that bis-adducts of silane or ethylene are not present in significant concentrations, if they are present at all.

Table S10. Calculated spin Hamiltonian parameters of the bis-silane and bis-ethylene adducts of Rh(II)-**3**.

**Species**	g_x	g_y	g_z		**A_x (MHz)**	**A_y (MHz)**	**A_z (MHz)**
Rh-(silane)$_2$	2.161	2.161	1.997	^{103}Rh	-68	-68	-105
				^{1}H	-8	-9	52
					-8	-9	52
Rh-(ethylene)$_2$	2.076	2.074	1.999	^{103}Rh	-71	-71	-115

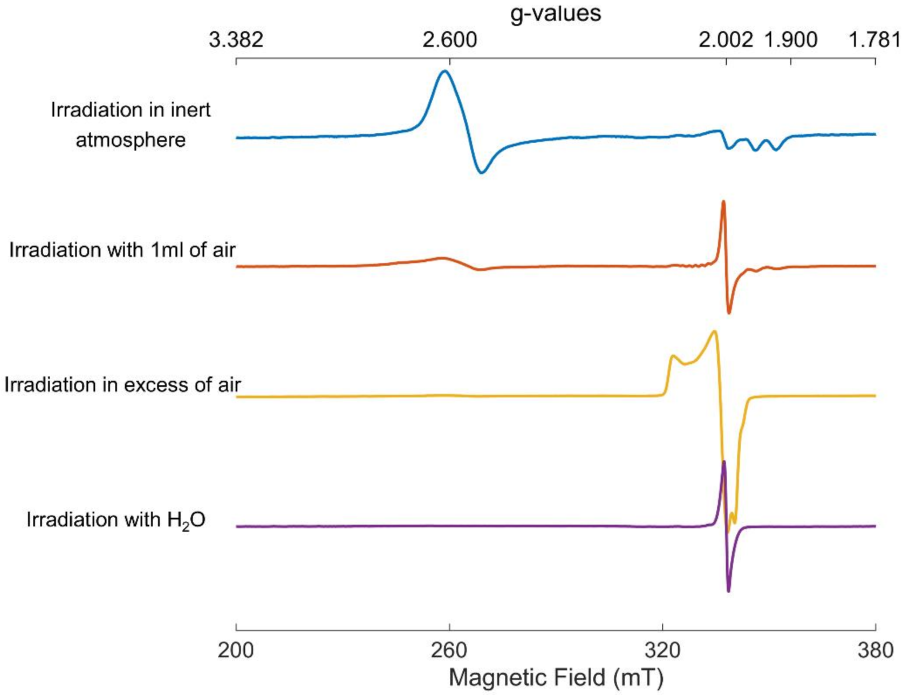

Figure S31. Experimental CW-EPR spectrum of Rh(II)-**3** (1 mg) measured after illuminating the sample in inert atmosphere (yellow line), in presence of 1 ml of air (blue line), in excess of air (red line) and in presence of water (violet line).

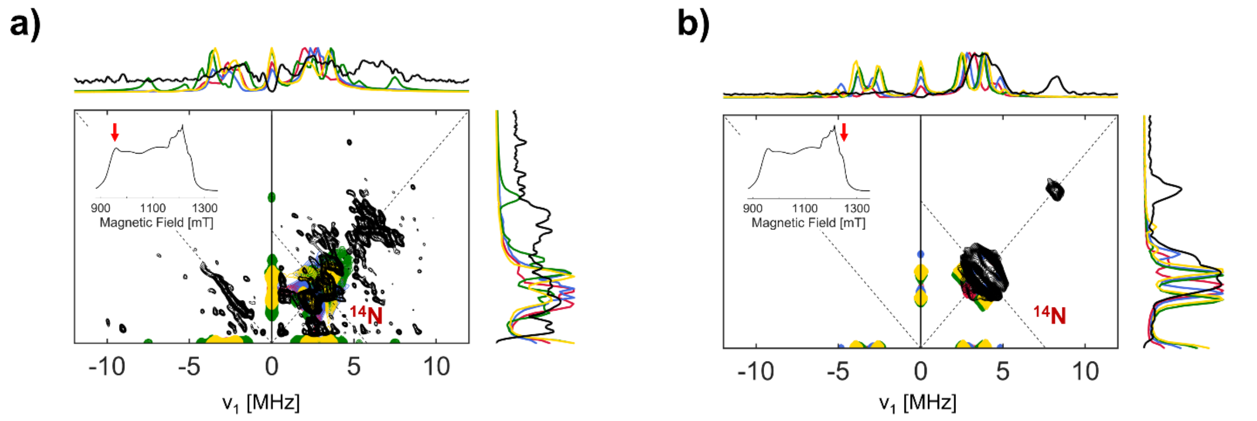

Figure S32. Experimental (black) and simulated (red, blue, yellow and green) ^{14}N Q-band HYSCORE spectra of irradiated Rh(II)-**3** acquired at 8 K and at different field positions indicated with red arrows. a) 958.2 mT and b) 1243.2 mT. The relative sum projections are reported for each quadrant of the spectra. Simulated patterns were obtained by using four spin systems each of them containing one ^{14}N nucleus and one unpaired electron (S=1/2 and I=1). Relative orientations of the ^{14}N hyperfine and quadrupole tensors were fixed from DFT calculations. The parameters used for obtaining the simulations are reported in Table S11.

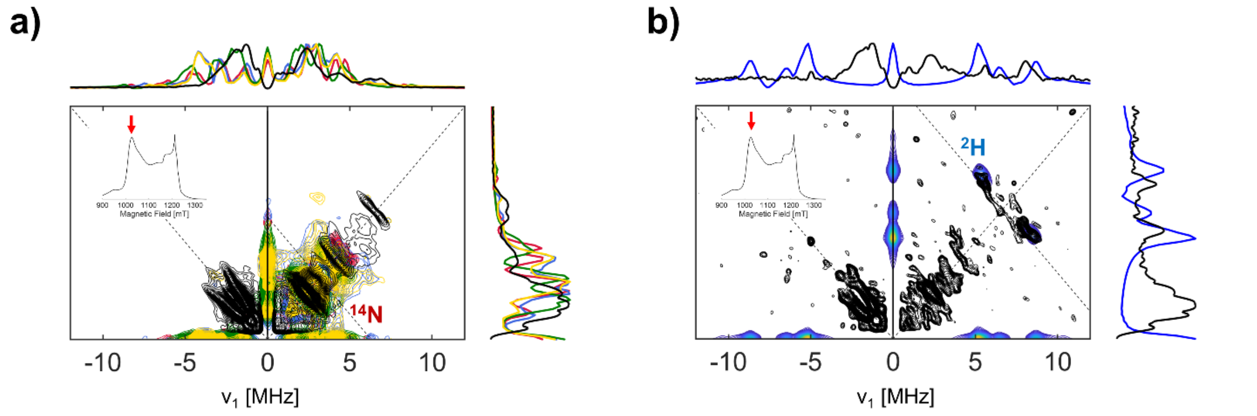

Figure S33. Experimental (black) and simulated Q-band HYSCORE spectra of $SiEt_3$ σ-adducts of Rh(II)-**3** acquired at 8 K. The field position at which the spectra were acquired is indicated with the red arrow. In a) Rh(II)-**3**+$HSiEt_3$; simulated patterns (red, blue, yellow and green) were obtained by using four spin systems each of them containing one ^{14}N nucleus and one unpaired electron (S=1/2 and I=1) while in b) Rh(II)-**3**+$DSiEt_3$; simulated patterns (blue traces) refer to one spin system containing one ^{2}H nucleus and one unpaired electron (S=1/2 and I=1). The relative sum projections are reported for each quadrant of the spectra. The parameters used for obtaining the simulations are reported in Table S11.

Table S11. Computed and experimental spin Hamiltonian parameters for the ^{14}N nuclei coupled to the unpaired electron in Rh(II)-**3** and ^{14}N and ^{2}H nuclei coupled to the unpaired electron in Rh(II)-**6**. Hyperfine and quadrupole couplings are given in MHz while Euler angles are given in degrees. For the simulation of the spectra the DFT computed g-frame and Euler angles for hyperfine (α,β,γ) and quadrupole tensors

(α’,β’,γ’) were adopted. The computed data for Rh(II)-3 refer to the Rh(II)-3-$(C_6H_6)_2$ model while the computed data for Rh(II)-**3**+silane refers to 6-C_6H_6 model.

System		Nucleus	Ax	Ay	Az	α	β	γ	|e²qQ/h|	η	α'	β'	γ'
Rh(II)-3	Experimental	$^{14}N_1$	0.4(3)	0.2(3)	1.5(3)	-99	45	103	1.5(5)	0.5(2)	-104	47	111
	Computed		-0.2	0.3	1.2				2.0	0.5			
	Experimental	$^{14}N_2$	0.2(3)	0.1(3)	1.2(3)	-80	45	92	1(5)	0.5(2)	-76	44	91
	Computed		-0.2	0.3	1.2				2.0	0.5			
	Experimental	$^{14}N_3$	-1.2(3)	0.3(3)	-1.5(3)	72	44	-88	1(5)	0.5(2)	-73	47	66
	Computed		-0.4	0.5	-0.9				2.0	0.5			
	Experimental	$^{14}N_4$	-1.2(3)	0.3(3)	-1.5(3)	-108	44	91	1(5)	0.5(2)	-107	45	91
	Computed		-0.4	0.5	-0.9				2.0	0.5			
Rh(II)-3+($^1H/^2H$)$SiEt_3$	Experimental	$^{14}N_1$	0.3(3)	-0.2(3)	1.4(3)	157	29	-158	4.0(5)	0.4(2)	36	18	-31
	Computed		0.2	-0.3	1.4				2.0	0.5			
	Experimental	$^{14}N_2$	0.5(3)	-0.1(3)	2.6(3)	60	11	-87	2.7(5)	0.2(2)	65	11	-81
	Computed		2.1	1.6	3.2				2.0	0.5			
	Experimental	$^{14}N_3$	0.1(3)	-0.5(3)	2.2(3)	31	21	-31	2.5(5)	0.2(2)	150	22	-155
	Computed		1.9	1.4	3.2				2.0	0.5			
	Experimental	$^{14}N_4$	0.4(3)	0.2(3)	2.3(3)	133	11	-107	2.6(5)	0.3(2)	130	12	-116
	Computed		1.6	1.1	2.8				2.0	0.5			
	Experimental	^2H	-1.6(3)	-1.9(3)	5.2(6)	84	18	-84	0.5(5)	0.2(1)			
	Computed		-1.3	-1.7	8.1				0.1	0.0			

10 Determination of Rh-Rh Distance via CW-EPR

An analysis of the broadening of the EPR signal of the Rh(II) species in PCN-224 and PCN-222 (Rh(II)-**3** and Rh(II)-**4**, respectively) allowed us to determine the average Rh(II)-Rh(II) distance by using the point-dipole

approximation model. By expressing the gyromagnetic ratio γ_n as a function of the g-values ($\gamma_n = \frac{g_n \beta_e}{\hbar}$), the dipolar interaction between species A and species B may be expressed with the following formula:[66-67]

$$\omega_{AB} = \frac{\mu_0}{4\pi h} \frac{g_A g_B \beta_e^2}{r_{AB}^3} (1 - 3\cos^2\theta)$$

where g_A and g_B are the g-values of species A and species B, μ_0 is the permeability of vacuum, h is the Planck constant and β_e is the Bohr magneton, θ is the angle between the interspin vector and the direction of the magnetic field $\mathbf{B}_0$ and r is the distance between species A and B. In this case, θ was set equal to 0. The Rh(II)-Rh(II) separations values calculated within the point-dipole approximation are in good agreement with the porphyrin-porphyrin distances derived from SC-XRD data of PCN-224 and PCN-222. Since the Rh(II) centers are located in the porphyrin plane, the distance between the centers of the porphyrin rings are equivalent to the distances between Rh(II) centers.

Table S12. Rh-Rh distances determined from the analysis of the CW-EPR spectra and XRD data for different PCN systems.

PCN framework	Rh-Rh distance from EPR	Porphyrin distance from XRD
Rh(II)-**3**	1.81 nm	1.9 nm
Rh(II)-**4**	1.24 nm	1.06 nm and 1.67 nm

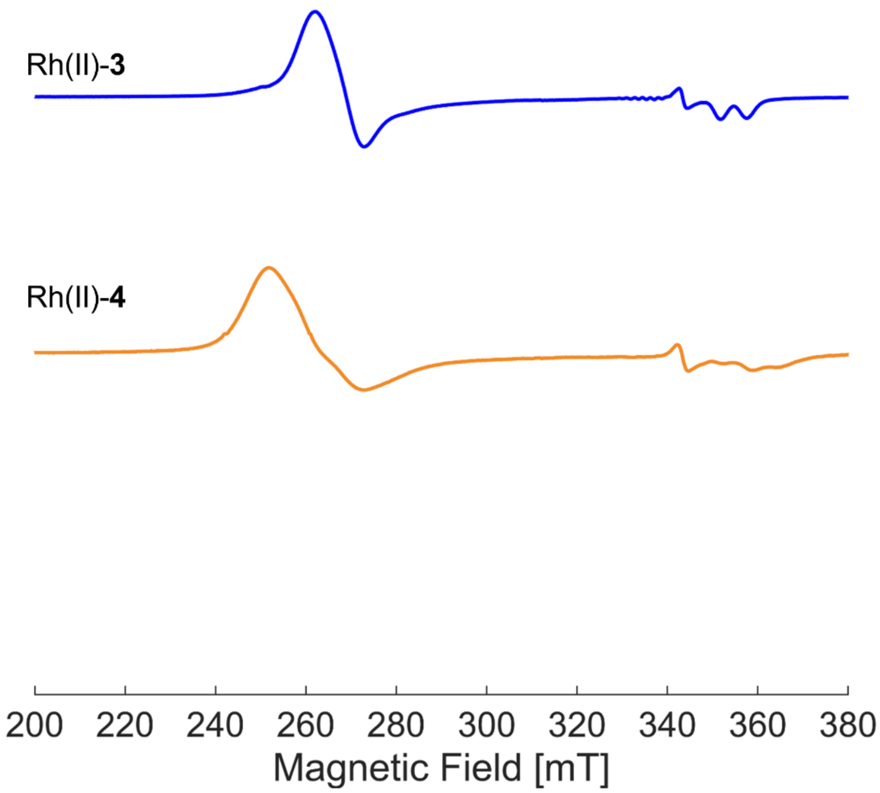

Figure S34. Experimental CW-EPR spectra of Rh^{II}-porphyrin species within different MOFs framework. We attribute the differential broadening of the EPR signals to differences in the spacing of Rh^{II} centers in the two frameworks.

11 Radical Trapping Experiments with TEMPO

11.1 Light-mediated Conditions

11.1.1 In the Presence of Ethylene

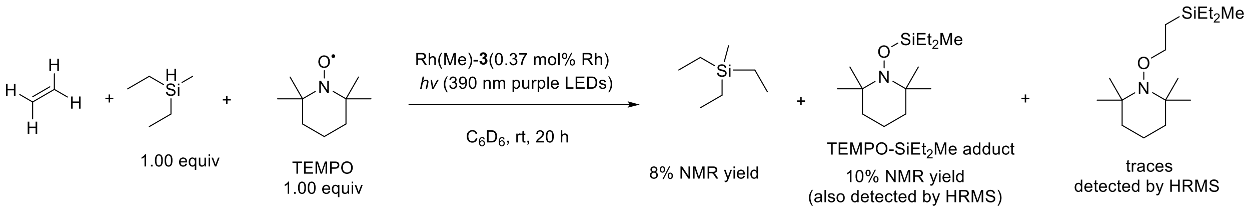

In an argon-filled glovebox, Rh(Me)-**3** (1.0 mg, 0.70 µmol, 0.37 mol%), C_6D_6 (1.0 mL), and diethylmethylsilane (27.7 µL, 191 µmol, 1.00 equiv), TEMPO (29.8 mg, 191 µmol, 1.00 equiv) were added to a 9 mL glass vial containing a cross-type stir bar (5 x 10 mm). Then the vial was sealed with a screw cap that contains a septum, and transferred out of the glovebox. The reaction mixture was subjected to sonication for 1 minute in order to evenly suspend the MOF crystallites throughout the solvent. Ethylene was then bubbled into the reaction vial for 1 minute in order to saturate the solvent with ethylene. To prevent oxygen leaking into the reaction mixture as ethylene was introduced, the vent needle that released excess ethylene from the reaction vial was connected to an inert gas manifold which was connected to an oil bubbler. Following the addition of ethylene, the reaction vial was swiftly transferred into a glovebox to replace the punctured septum cap with a screw cap. After removal from the glovebox, the reaction was stirred at 500 rpm at room temperature and irradiated with a 390 nm purple LED for 20 h, where the reaction was cooled by in-house compressed air flow. The reaction vial was subsequently subjected to centrifugation at 4500 rpm for 5 min (to ensure that the MOF particles settle down on the bottom of reaction vial), and transferred into a glovebox. The C_6D_6 solution was carefully removed using a syringe and submitted for further analysis.

Based on ^{1}H NMR analysis, the yield of the hydrosilylation product was reduced to 8% (Figure S35), which suggests that radical intermediates may be involved in the mechanism of light-mediated hydrosilylation. Furthermore, a TEMPO adduct of the presumed silyl radical intermediate **8**, TEMPO-$SiEt_2Me$ **16**, was detected by NMR in a yield of 10% (Figure S35). The identity of the TEMPO adduct was further confirmed by its ^{29}Si NMR chemical shift (23.1 ppm, Figure S36), which is consistent with an analogous reported compound TEMPO-***Si***Me_3 (22.1 ppm),[68] and HRMS.[*] Moreover, the carbon radical, formed by Si radical addition to ethylene, can be also trapped by TEMPO in trace amount, which is detected by HRMS.[†]

[*] HRMS GC-EI (m/z): calc'd for $C_{14}H_{31}N_1O_1Si_1$ $[M]^+$: 257.2169; Found: 257.2167. Deviation: 1.1 ppm

[†] HRMS ESI (m/z): calc'd for $C_{16}H_{36}N_1O_1Si_1$ $[M+H]^+$: 286.2561; Found: 286.2559. Deviation: 0.6 ppm

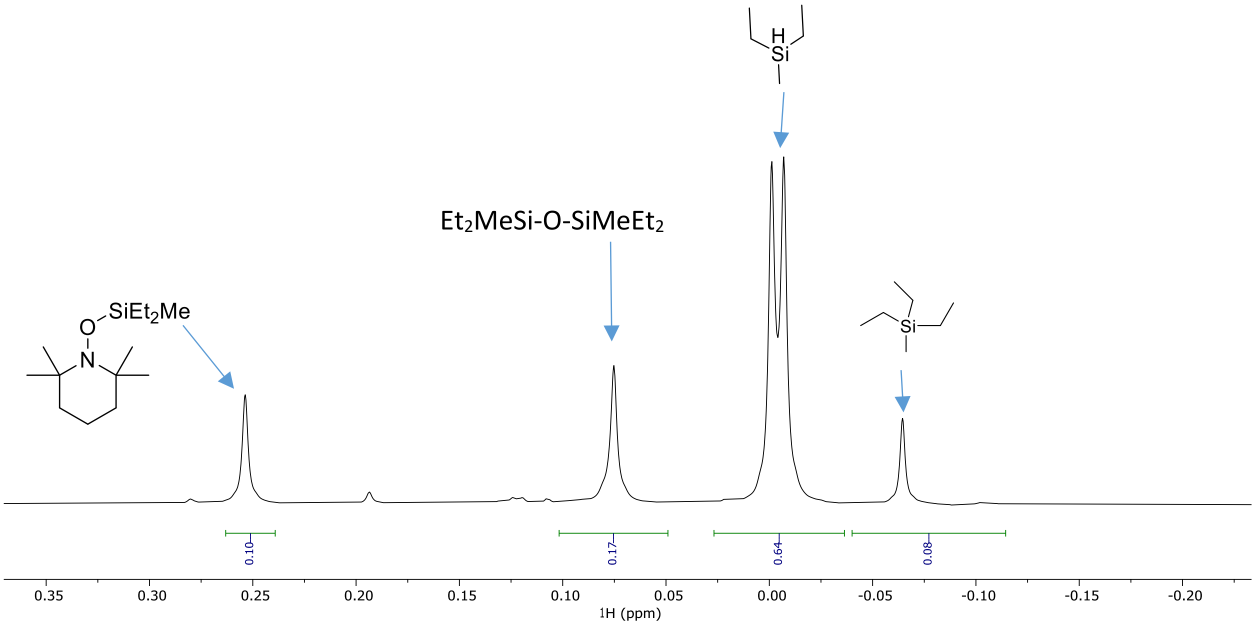

Figure S35. ^{1}H NMR spectrum of reaction mixture in C_6D_6.

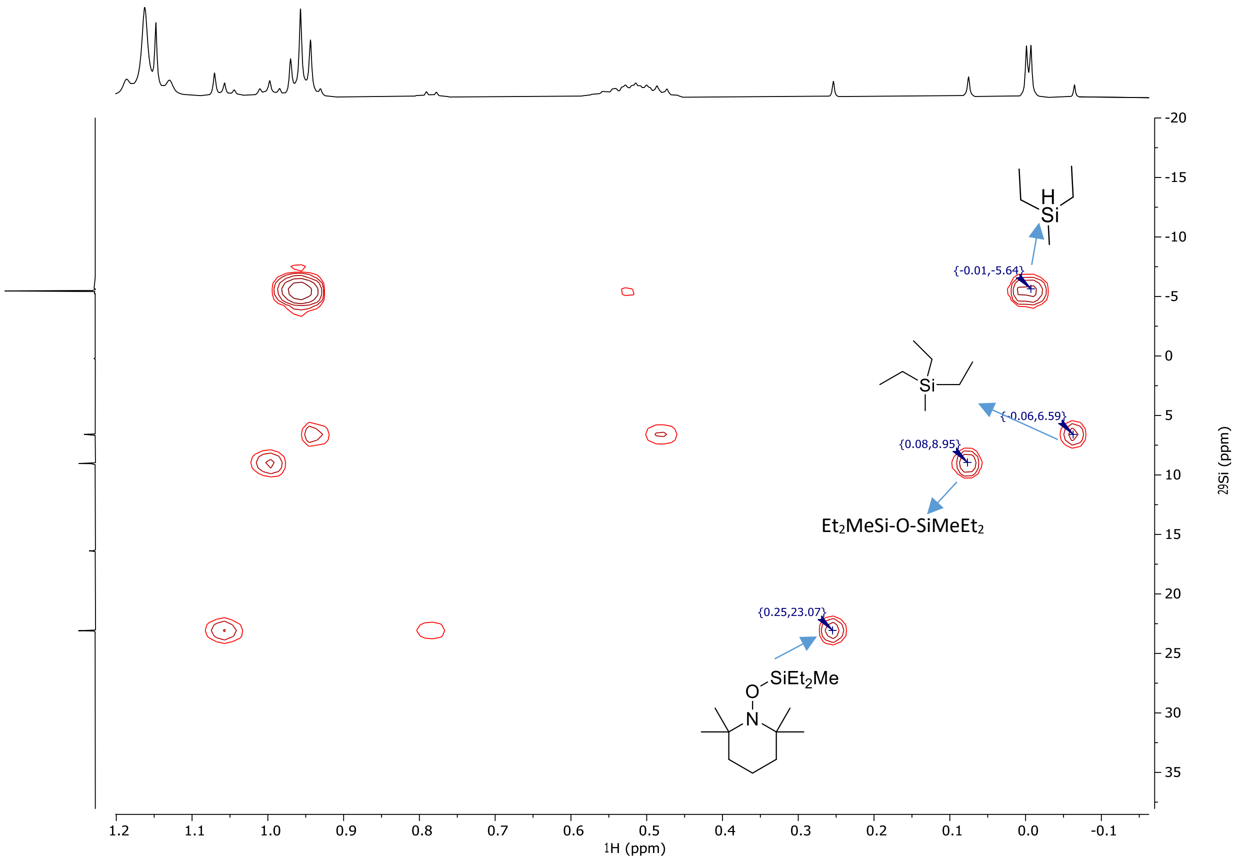

Figure S36. ^{1}H-^{29}Si HMBC spectrum of reaction mixture in C_6D_6.

11.1.2 In the Absence of Ethylene

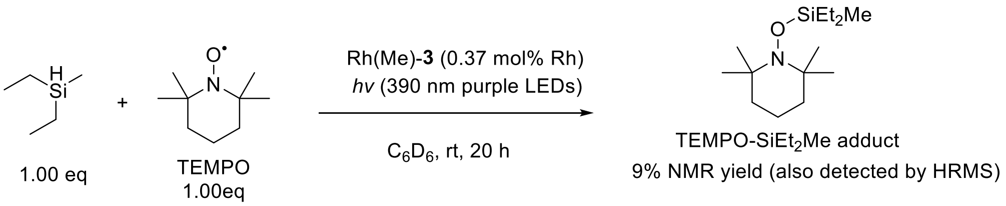

In an argon-filled glovebox, Rh(Me)-**3** (1.0 mg, 0.70 µmol, 0.37 mol%), C_6D_6 (1.0 mL), and diethylmethylsilane (27.7 µL, 191 µmol, 1.00 equiv), TEMPO (29.8 mg, 191 µmol, 1.00 equiv) were added to a 9 mL glass vial containing a cross-type stir bar (5 x 10 mm). Then the vial was sealed with a screw cap that contains a septum, and transferred out of the glovebox. The reaction mixture was subjected to sonication for 1 minute in order to evenly suspend the MOF crystallites throughout the solvent. After removal from the glovebox, the reaction was stirred at 500 rpm at room temperature and irradiated with a 390 nm purple LED for 20 h, where the reaction was cooled by in-house compressed air flow. The reaction vial was subsequently subjected to centrifugation at 4500 rpm for 5 min (to ensure that the MOF particles settle down on the bottom of reaction vial), and transferred into a glovebox. The C_6D_6 solution was carefully removed using a syringe and submitted for further analysis.

Based on ^{1}H-NMR analysis, TEMPO-$SiEt_2Me$ was detected in 9% NMR yield (Figure S37), and its composition was further confirmed by GC-HRMS. The detection of TEMPO-$SiEt_2Me$ in the absence of

ethylene indicated that the formation of Si radicals under light-mediated reaction conditions does not require the presence of ethylene. We propose that light-mediated H–Si bond cleavage in 3c-3e adduct **6** can give rise to the formation of silyl radicals. A control experiment was carried out in the absence of Rh(Me)-**3** catalyst but under otherwise identical conditions to those shown above. Based on ^{1}H-NMR analysis, no TEMPO-$SiEt_2Me$ adduct could be detected (Figure S38 and Figure S39), and GC-EI-HRMS analysis only showed traces of TEMPO-$SiEt_2Me$.

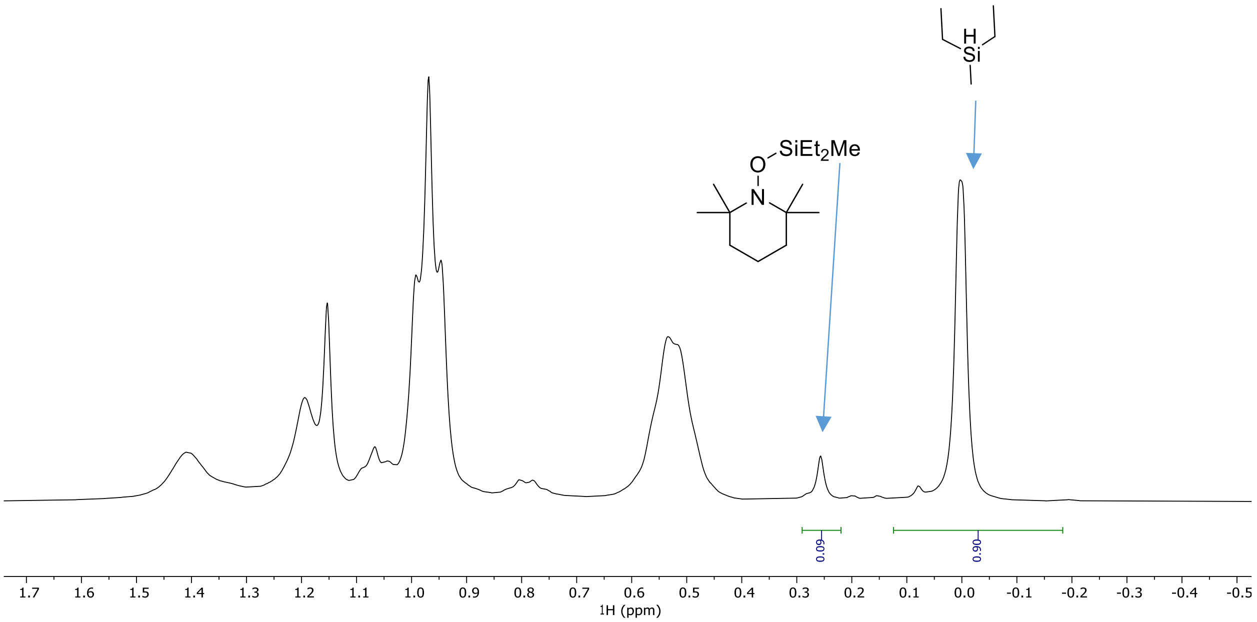

Figure S37. ^{1}H NMR spectrum of reaction mixture in C_6D_6.

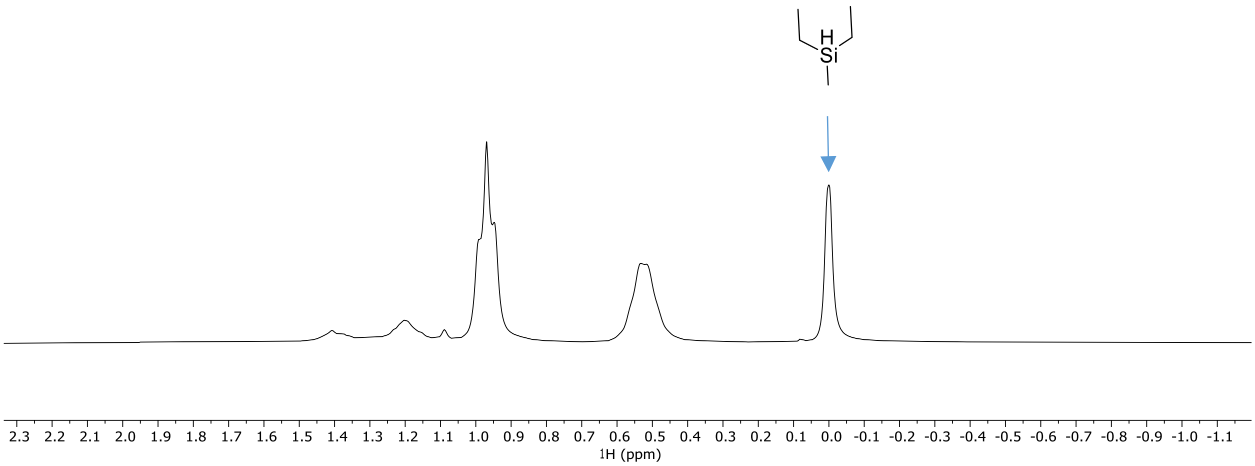

Figure S38. ^{1}H NMR spectrum of reaction mixture in C_6D_6.

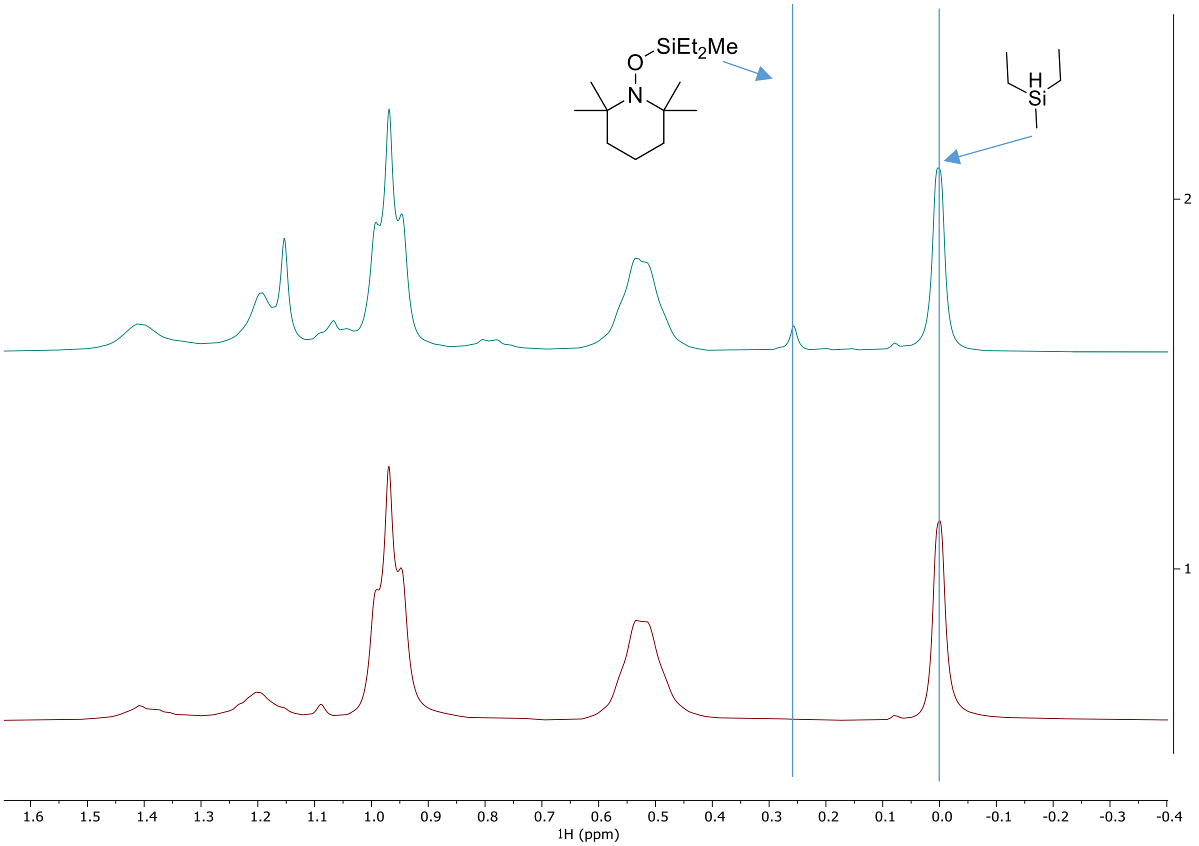

Figure S39. Stacked ^{1}H NMR spectra of reaction mixtures in C_6D_6: top-with Rh(Me)-**3** catalyst; bottom-without Rh(Me)-**3** catalyst.

11.2 In the Absence of Light

11.2.1 In the Presence of Ethylene

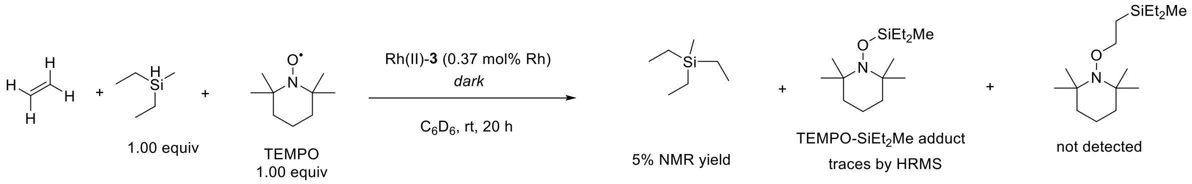

In an argon-filled glovebox, Rh(Me)-**3** (1.0 mg, 0.070 µmol, 0.37 mol%) and C_6D_6 (1.0 mL), were added to a 9 mL VWR glass vial containing a cross-type stir bar (5 x 10 mm). Then the vial was sealed with a screw cap that contains a septum, and transferred out of the glovebox. The reaction mixture was subjected to sonication for 1 minute in order to evenly suspend the MOF crystallites throughout the solvent. After that, Rh(Me)-**3** was subjected to photolysis to produce active catalyst Rh(II)-**3** with a 390 nm purple LED for 20 h at room temperature and a stirring rate of 500 rpm, where the reaction was cooled by in-house compressed air flow.

Then the vial was transferred into glovebox, diethylmethylsilane (27.7 µL, 191 µmol, 1.00 equiv) was added to a 9 mL glass vial containing a cross-type stir bar (5 x 10 mm). Then the vial was sealed with a

screw cap that contains a septum, and transferred out of the glovebox. The reaction mixture was subjected to sonication for 1 minute in order to evenly suspend the MOF crystallites throughout the solvent. Ethylene was then bubbled into the reaction vial for 1 minute in order to saturate the solvent with ethylene. To prevent oxygen leaking into the reaction mixture as ethylene was introduced, the vent needle that released excess ethylene from the reaction vial was connected to an inert gas manifold which was connected to an oil bubbler. Following the addition of ethylene, the reaction vial was swiftly transferred into a glovebox to replace the punctured septum cap with a screw cap. After removal from the glovebox, the reaction was stirred at 500 rpm at room temperature under dark (covered by aluminum foil) for 1 h. Then, transferred back into glovebox, TEMPO (29.8 mg, 191 µmol, 1.00 equiv) was added into the vial, and the vial was sealed with a screw cap. The reaction was stirred at 500 rpm at room temperature under dark (covered by aluminum foil) for 20 h. The reaction vial was subsequently subjected to centrifugation at 4500 rpm for 5 min (to ensure that the MOF particles settle down on the bottom of reaction vial), and transferred into a glovebox. The C_6D_6 solution was carefully removed using a syringe and submitted for further analysis.

Based on ^{1}H NMR analysis, the yield of the desired hydrosilylation product was around 5% (Figure S40). The addition of TEMPO thus did not lead to a significant reduction of the efficiency of the thermal ethylene hydrosilylation (a comparative experiment carried out in the absence of TEMPO with the given batch of low-oxygen content Rh(II) catalyst furnished 7% conversion). Free radicals that are able to be intercepted by TEMPO thus appear to be of lesser importance to the olefin hydrosilylation in the dark compared to olefin hydrosilylation under light-mediated conditions. The TEMPO adduct of silyl radical **8**, TEMPO-$SiEt_2Me$ **16**, was only detected in traces by GC-EI-HRMS, and could not be detected in the ^{1}H NMR spectrum.

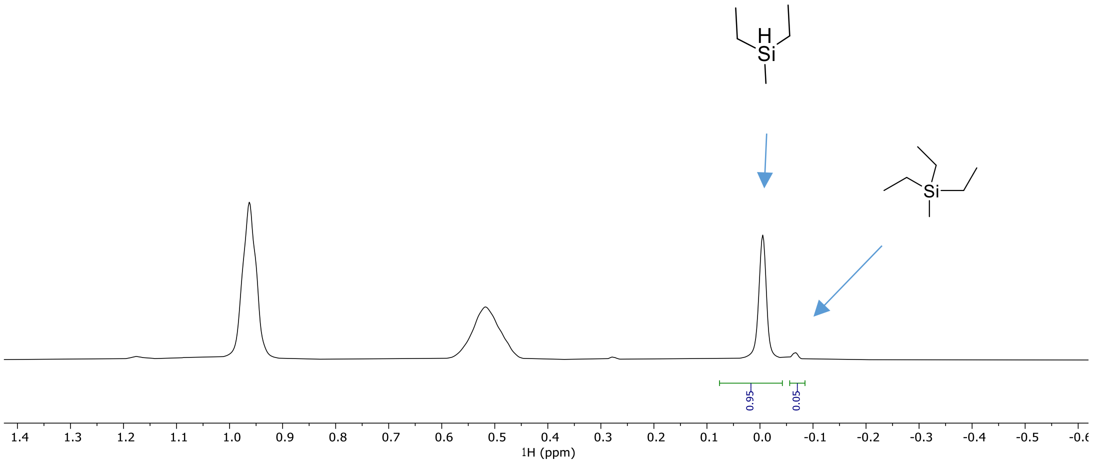

Figure S40. ^{1}H NMR spectrum of reaction mixture in C_6D_6.

11.2.2 In the Absence of Ethylene

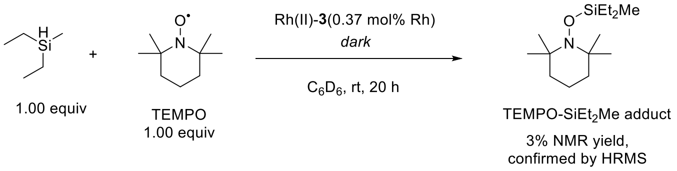

In an argon-filled glovebox, Rh(Me)-**3** (1.0 mg, 0.070 µmol, 0.37 mol%) and C_6D_6 (1.0 mL), were added to a 9 mL VWR glass vial containing a cross-type stir bar (5 x 10 mm). Then the vial was sealed with a screw cap that contains a septum, and transferred out of the glovebox. The reaction mixture was subjected to sonication for 1 minute in order to evenly suspend the MOF crystallites throughout the solvent. After that, Rh(Me)-**3** was subjected to photolysis to produce active catalyst Rh(II)-**3** with a 390 nm purple LED for 20 h at room temperature and a stirring rate of 500 rpm, where the reaction was cooled by in-house compressed air flow.

Then the vial was transferred into the glovebox, diethylmethylsilane (27.7 µL, 191 µmol, 1.00 equiv) was added to a 9 mL glass vial containing a cross-type stir bar (5 x 10 mm). Then the vial was sealed with a screw cap that contains a septum, and transferred out of the glovebox. The reaction mixture was subjected to sonication for 1 minute in order to evenly suspend the MOF crystallites throughout the solvent. The reaction was stirred at 500 rpm at room temperature under dark (covered by aluminum foil) for 1 h. Then, transferred back into glovebox, TEMPO (29.8 mg, 191 µmol, 1.00 equiv) was added into the vial, and the vial was sealed with a screw cap. The reaction was stirred at 500 rpm at room temperature under dark (covered by aluminum foil) for 20 h. The reaction vial was subsequently subjected to centrifugation at 4500 rpm for 5 min (to ensure that the MOF particles settle down on the bottom of reaction vial), and transferred into a glovebox. The C_6D_6 solution was carefully removed using a syringe and submitted for further analysis.

Based on ^{1}H NMR analysis, the TEMPO trapping product of Si radical **8**, TEMPO-$SiEt_2Me$ **16**, was detected in 3% NMR yield (Figure S41) and its structure and composition was further confirmed by ^{1}H-^{29}Si HMBC NMR (Figure S42) and GC-EI-HRMS. Successful detection of **16** in the absence of ethylene or light suggests that the TEMPO radical can abstract the silyl group from [Rh-H-Si] 3c-3e adduct **6** to form TEMPO-$SiEt_2Me$. Notably, though, abstraction of silyl radical from 3c-3e adduct **6** appears to be inefficient in the presence of ethylene (see Figure S38) – only traces of **16** were detected in the presence of ethylene.

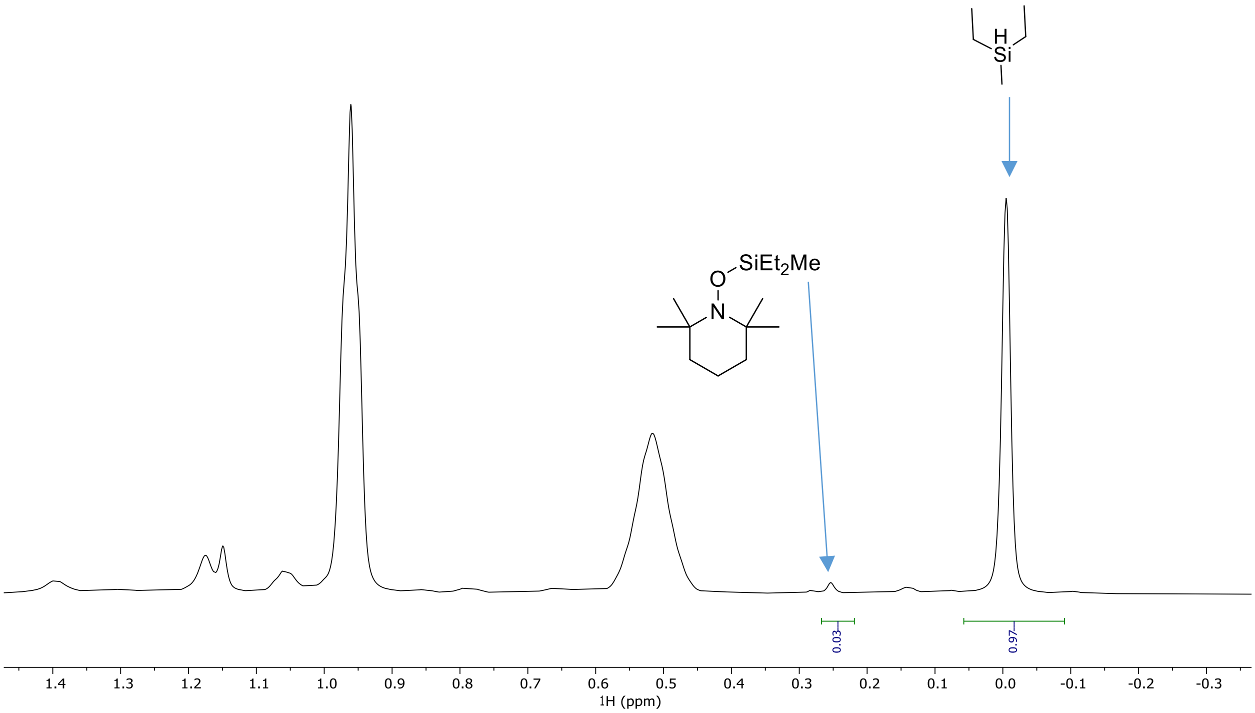

Figure S41. ^{1}H NMR spectrum of the reaction mixture in C_6D_6.

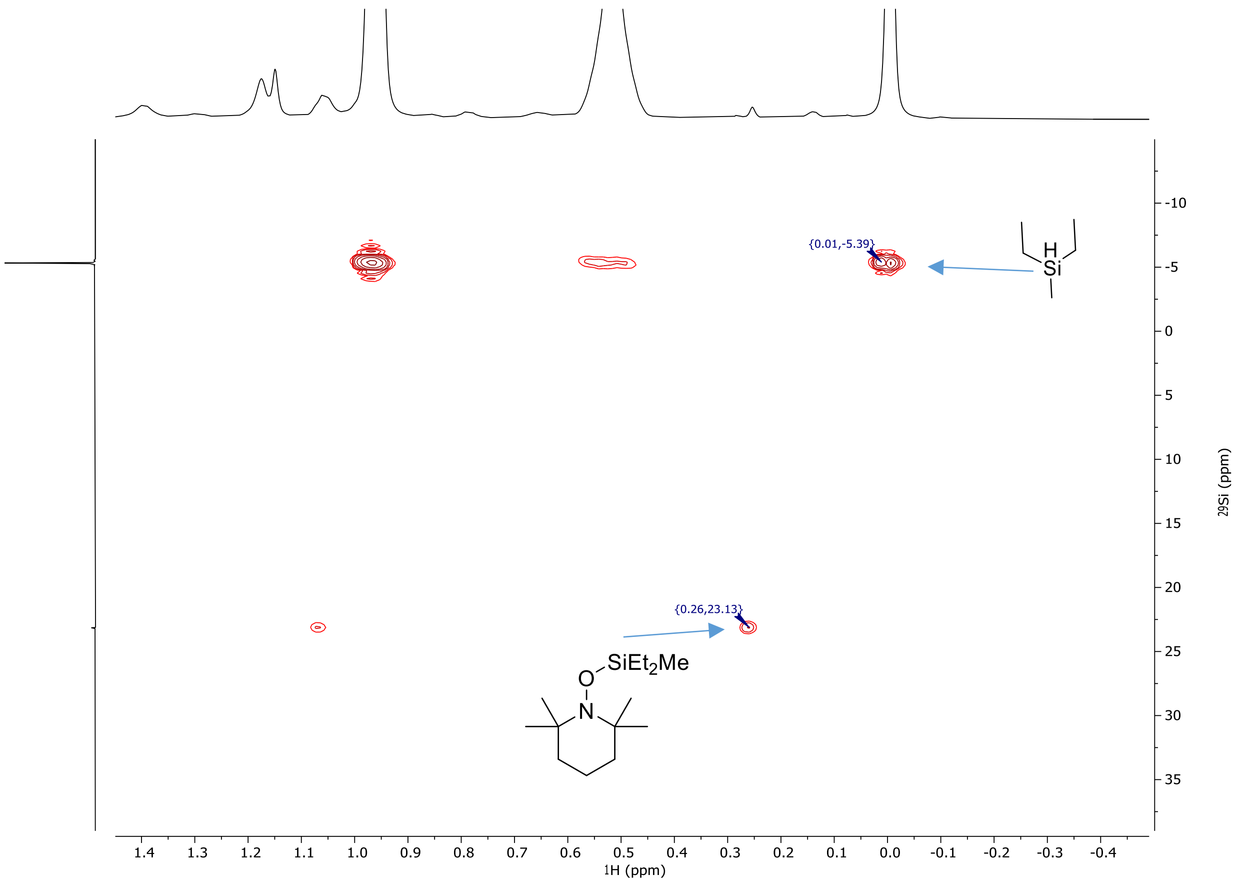

Figure S42. ^{1}H-^{29}Si HMBC spectrum of the reaction mixture in C_6D_6.

12 By-product Generation

The reaction of olefins with silanes catalyzed by **3** furnishes the olefin hydrosilylation product as the major product alongside minor amounts of the dehydrosilylated olefin, silanol as well as siloxane. We found that electron transfer from an adjacent Rh(II)porphyrin center in the MOF to the transition structure gives rise to an opportunity for proton loss in the transition state which leads to the formation of minor amounts of dehydrosilylated side product. A mechanistic cause for the formation of minor amounts of silanol and siloxane had not been identified, however. Based on experimental data that had been collected to date, we suspected that water and/or O_2 contamination of reaction mixtures could be to blame, so we carried out additional experiments to determine the cause of silanol and siloxane formation.

To determine whether 1,1,3,3-tetraethyl-1,3-dimethyldisiloxane and diethyl(methyl)silanol could be formed due to the presence of water in the reaction mixture, we carried out ethylene hydrosilylation with **3** according to our standard procedure but we used D_2O instead of C_6D_6 as the reaction solvent (Figure S43). D_2O was purged with Ar flow for half an hour to remove dissolved oxygen prior to use. After a reaction time of 9 h, we found 22% hydrosilylation product as well as 78% diethyl(methyl)silanol. While no siloxane was formed in the presence of solvent quantities of water, the presence of trace water in a reaction mixture may however favor the formation of siloxane. If water amounts in the solvent are limited, silanol would constitute the most potent nucleophile present in the reaction mixture once trace water is consumed, so that partial conversion of silanol to 1,1,3,3-tetraethyl-1,3-dimethyldisiloxane would be expected.

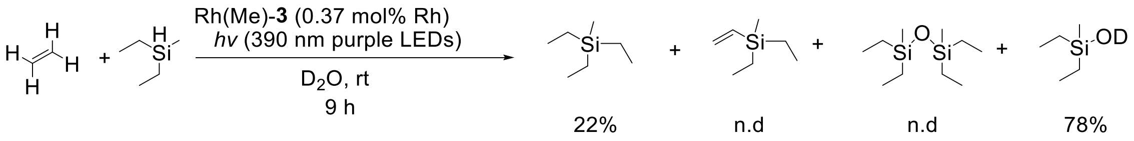

Figure S43. Ethylene hydrosilylation catalyzed by Rh(II)-**3** in D_2O.

While the presence of minor amounts of water could account for the formation of both oxidized silane by-products, observed, the presence of oxygen likely also contributes to their formation. We carried out multiple repetitions for a given set of reaction conditions on different days. Even though identical conditions were used and the same batch of benzene was employed, the amount of 1,1,3,3-tetraethyl-1,3-dimethyldisiloxane observed differed between runs (Figure S44). Since the water content of the reactions (which were all set up inside a glove box from identical batches of chemicals) is expected to be the same, we attribute the variations in siloxane formation observed to variations in oxygen content. While the presence of minor amounts of oxygen during the generation of the active catalyst is vital to ensuring optimal catalyst activity, the presence of oxygen during the use of Rh(II)-**3** does not aid reaction outcomes and likely induces the formation of side products.

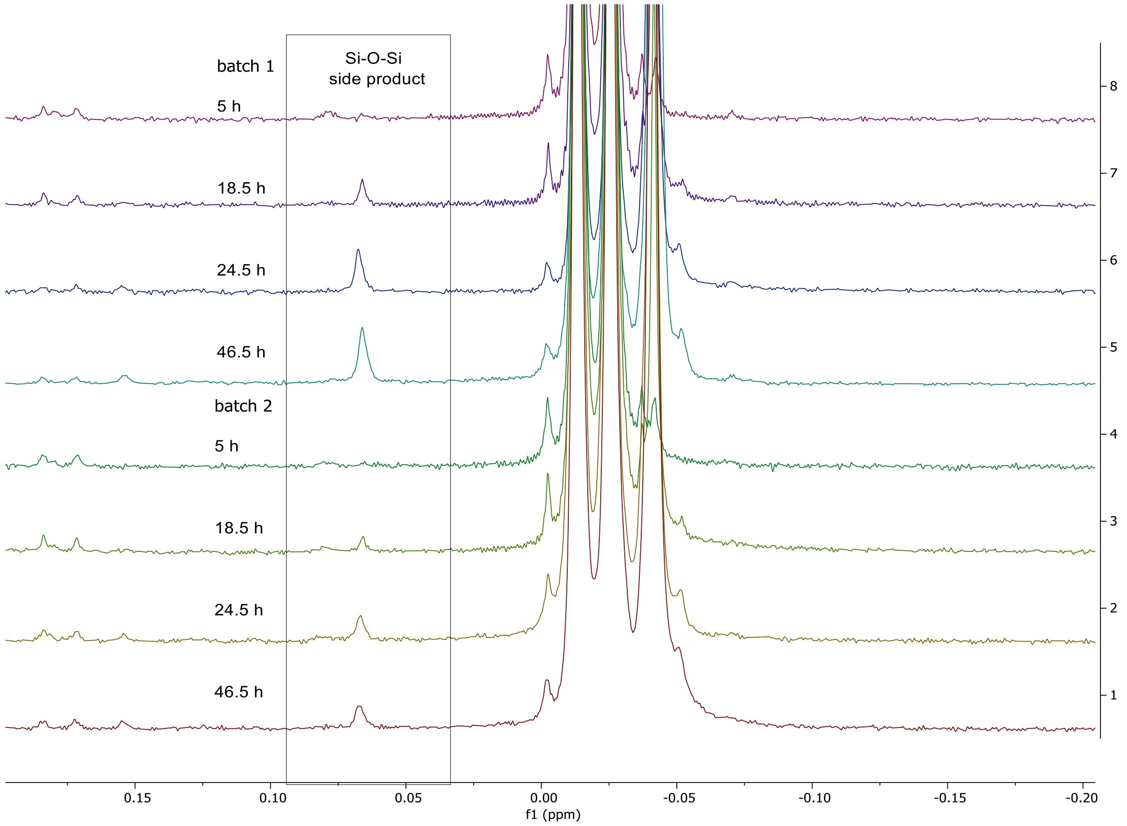

Figure S44. Comparison of ^{1}H NMR spectra of two different thermal hydrosilylation reactions mixtures analyzed at different time points. Signals corresponding to Si-O-Si are highlighted with a black box. Signals attributable to Si-OH would appear around –0.2 ppm so that formation of minor amounts of silanol would not be visible due to be presence of peaks corresponding to diethylmethylsilane at a similar chemical shift.

13 Double Label Experiment

a) dark, 23 h

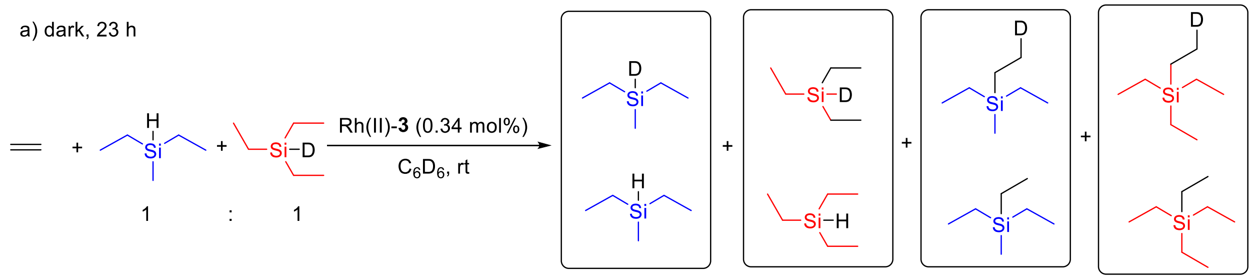

b) 390 nm LED, 0.5 h

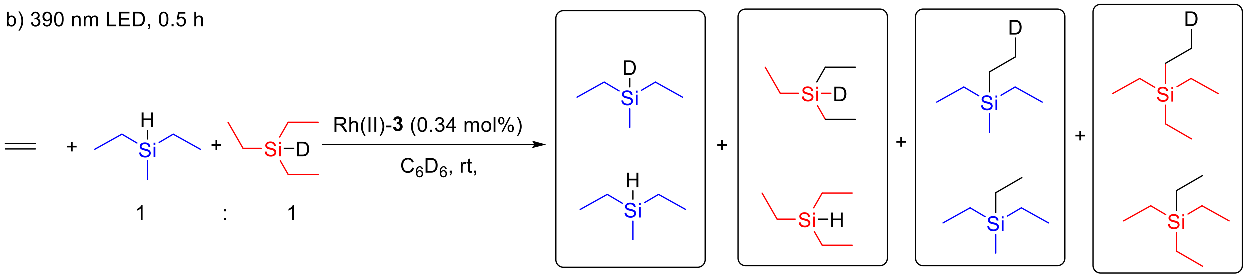

In an argon-filled glovebox, 1 mg Rh(II)-**3**, diethylmethylsilane (15.0 µL, 103 µmol, 1.00 equiv), triethylsilane-d (16.5 µL, 103 µmol, 1.00 equiv) and 1 mL C_6D_6 were added to a 9 ml glass vial which was removed from the glovebox. The reaction mixture was subjected to sonication for another 1 minute in order to evenly suspend the MOF crystallites throughout the solvent. Ethylene was then bubbled into the reaction tube for 1 minute in order to saturate the solvent with ethylene. Then ethylene hydrosilylation was carried out under either dark or light-mediated reaction condition at room temperature according to the general reaction procedures.

Analysis of remaining silane reagents after a reaction time of 23 h in the absence of light furnished a Et_3SiD : Et_2MeSiD ratio of 4.5 : 1, while a 1 : 1 ratio was already observed for photochemical conditions after 0.5 h (Figure S45).

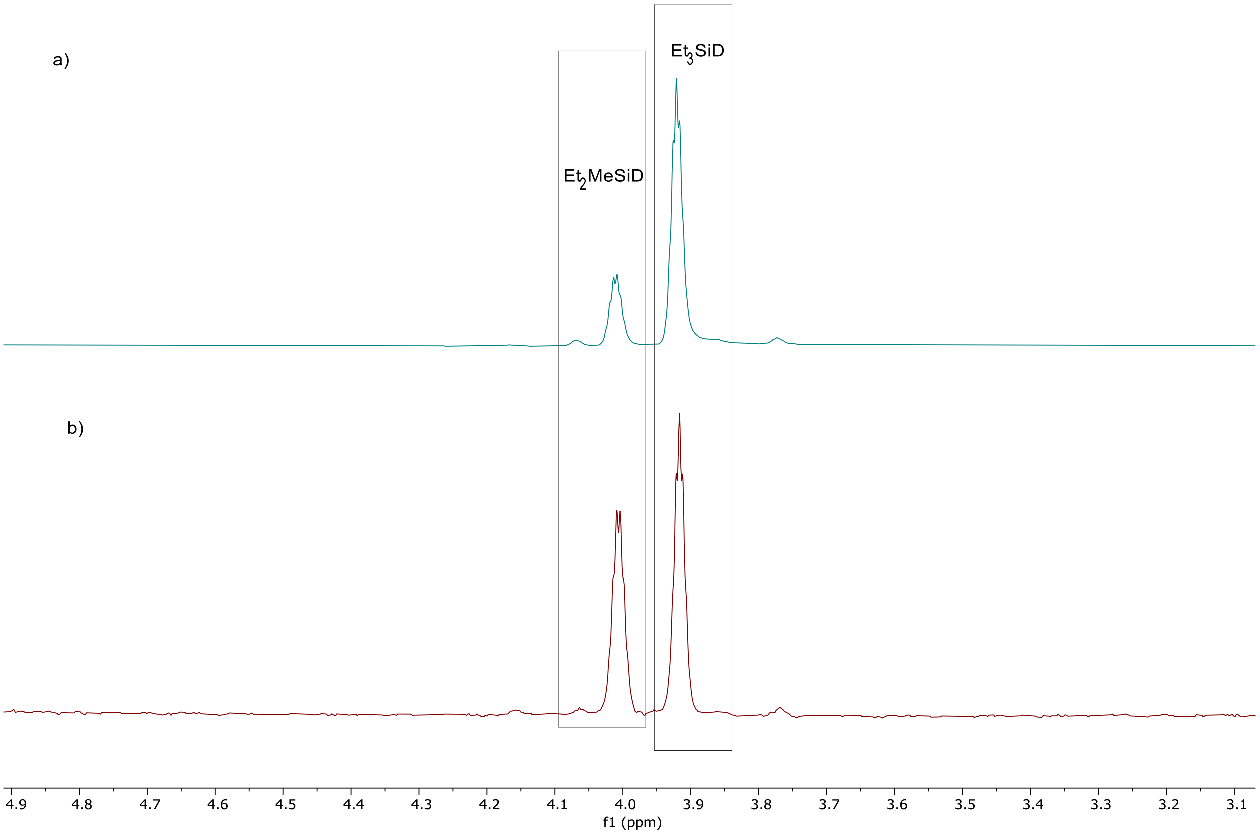

Figure S45. ^2H NMR spectra of a) the reaction mixture from ethylene hydrosilylation carried out in the dark for 23 h; and b) the reaction mixture from light-mediated ethylene hydrosilylation carried out for 0.5 h.

14 Solid-state NMR Studies

In order to gain insights into the reaction mechanism, the Rh-containing MOF catalysts were analyzed by solid-state NMR after being subjected to the reaction condition described in detail below. The headings used mirror the descriptions used in Figure 9A and 9B of the manuscript.

14.1 Silane and Light 1 h

In an argon-filled glovebox, Rh(Me)-**3** (5.0 mg, 0.35 µmol, 0.37 mol%) and C_6D_6 (5.0 mL), were added to a 9 mL VWR glass vial containing a cross-type stir bar (5 x 10 mm). Then the vial was sealed with a screw cap that contains a septum, and transferred out of the glovebox. The reaction mixture was subjected to sonication for 1 minute in order to evenly suspend the MOF crystallites throughout the solvent. After that, Rh(Me)-**3** was subjected to photolysis to produce active catalyst Rh(II)-**3** with a 390 nm purple LED for 20

h at room temperature and a stirring rate of 1000 rpm, where the reaction was cooled by in-house compressed air flow.

The reaction vial was transferred into glovebox, diethylmethylsilane (139 µL, 954 µmol, 1.00 equiv) were added to the 9 mL glass vial. Then the vial was sealed with a screw cap that contains a septum, and transferred out of the glovebox. The reaction mixture was subjected to sonication for 1 minute in order to evenly suspend the MOF crystallites throughout the solvent. The reaction was stirred at 1000 rpm at room temperature and irradiated with a 390 nm purple LED for 1 h, where the reaction was cooled by in-house compressed air flow. *Note: three parallel reactions were set-up simultaneously in order to collect enough MOF materials for solid state NMR analysis*. The three reaction vials were subsequently subjected to centrifugation at 4500 rpm for 5 min (to ensure that the MOF particles settle down on the bottom of reaction vial), and transferred into a glovebox. The C_6H_6 solution was carefully removed using a syringe, and the MOF material in the reaction vials were combined, and then dry and degassed C_6H_6 (5 mL) was added to wash MOFs, followed by 5 min centrifugation at 4500 rpm. The washing process was repeated for three times. Then the vial containing MOF material was transferred out glovebox, and further dried by argon flow for 1 h under dark. The MOF material was then transferred into glovebox, and was filled into an NMR rotor inside a glovebox for solid-state NMR analysis. The ^{1}H ssNMR spectrum is shown in Figure S46. The $^{29}Si\{^{1}H\}$ ssNMR spectrum is shown in Figure S47.

14.2 Ethylene, Silane and Light 1 h

In an argon-filled glovebox, Rh(Me)-**3** (5.0 mg, 0.35 µmol, 0.37 mol%) and C_6D_6 (5.0 mL), were added to a 9 mL VWR glass vial containing a cross-type stir bar (5 x 10 mm). Then the vial was sealed with a screw cap that contains a septum, and transferred out of the glovebox. The reaction mixture was subjected to sonication for 1 minute in order to evenly suspend the MOF crystallites throughout the solvent. After that, Rh(Me)-**3** was subjected to photolysis to produce active catalyst Rh(II)-**3** with a 390 nm purple LED for 20 h at room temperature and a stirring rate of 1000 rpm, where the reaction was cooled by in-house compressed air flow.

The reaction vial was transferred into glovebox, diethylmethylsilane (139 µL, 954 µmol, 1.00 equiv) were added to the 9 mL glass vial. Then the vial was sealed with a screw cap that contains a septum, and transferred out of the glovebox. The reaction mixture was subjected to sonication for 1 minute in order to evenly suspend the MOF crystallites throughout the solvent. Ethylene was then bubbled into the reaction vial for 1 minute in order to saturate the solvent with ethylene. To prevent oxygen leaking into the reaction mixture as ethylene was introduced, the vent needle that released excess ethylene from the reaction vial was connected to an inert gas manifold which was connected to an oil bubbler. Following the addition of ethylene, the reaction vial was swiftly transferred into a glovebox to replace the punctured septum cap with a screw cap. After removal from the glovebox, the reaction was stirred at 1000 rpm at room temperature and irradiated with a 390 nm purple LED for 1 h, where the reaction was cooled by in-house compressed air flow. *Note: three parallel reactions were set-up simultaneously in order to collect enough MOF materials for solid state NMR analysis*. The three reaction vials were subsequently subjected to centrifugation at 4500 rpm for 5 min (to ensure that the MOF particles settle down on the bottom of reaction vial), and transferred into a glovebox. The C_6H_6 solution was carefully removed using a syringe, and the MOF material in the reaction vials were combined, and then dry and degassed C_6H_6 (5 mL) was added to wash MOFs, followed by 5 min centrifugation at 4500 rpm. The washing process was repeated

for three times. Then the vial containing MOF material was transferred out glovebox, and further dried by argon flow for 1 h under dark. The MOF material was then transferred into glovebox, and was filled into solid state NMR rotor in glovebox for solid state NMR analysis. The ^{1}H ssNMR spectrum is shown in Figure S46. $^{29}Si\{^{1}H\}$ ssNMR spectrum is shown in Figure S47.

14.3 Ethylene and Silane 2 days

In an argon-filled glovebox, Rh(Me)-**3** (5.0 mg, 0.35 µmol, 0.37 mol%) and C_6D_6 (5.0 mL), were added to a 20 mL VWR glass vial containing a cross-type stir bar (5 x 10 mm). Then the vial was sealed with a screw cap that contains a septum, and transferred out of the glovebox. The reaction mixture was subjected to sonication for 1 minute in order to evenly suspend the MOF crystallites throughout the solvent. After that, Rh(Me)-**3** was subjected to photolysis to produce active catalyst Rh(II)-**3** with a 390 nm purple LED for 20 h at room temperature and a stirring rate of 1000 rpm, where the reaction was cooled by in-house compressed air flow.

The reaction vial was transferred into glovebox, diethylmethylsilane (139 µL, 954 µmol, 1.00 equiv) were added to the 20 mL glass vial. Then the vial was sealed with a screw cap that contains a septum, and transferred out of the glovebox. The reaction mixture was subjected to sonication for 1 minute in order to evenly suspend the MOF crystallites throughout the solvent. Ethylene was then bubbled into the reaction vial for 1 minute in order to saturate the solvent with ethylene. To prevent oxygen leaking into the reaction mixture as ethylene was introduced, the vent needle that released excess ethylene from the reaction vial was connected to an inert gas manifold which was connected to an oil bubbler. Following the addition of ethylene, ethylene balloon was attached into the reaction vial to keep the reaction under ethylene atmosphere. The reaction was stirred at 1000 rpm at room temperature under dark. *Note: three parallel reactions were set-up simultaneously in order to collect enough MOF materials for solid state NMR analysis*. The three reaction vials were subsequently subjected to centrifugation at 4500 rpm for 5 min (to ensure that the MOF particles settle down on the bottom of reaction vial), and transferred into a glovebox. The C_6H_6 solution was carefully removed using a syringe, and the MOF material in the reaction vials were combined, and then dry and degassed C_6H_6 (5 mL) was added to wash MOFs, followed by 5 min centrifugation at 4500 rpm. The washing process was repeated for three times. Then the vial containing MOF material was transferred out glovebox, and further dried by argon flow for 1 h under dark. The MOF material was then transferred into glovebox, and was filled into solid state NMR rotor in glovebox for solid state NMR analysis. ^{1}H ssNMR spectrum is shown in Figure S46. The $^{29}Si\{^{1}H\}$ ssNMR spectrum is shown in Figure S47.

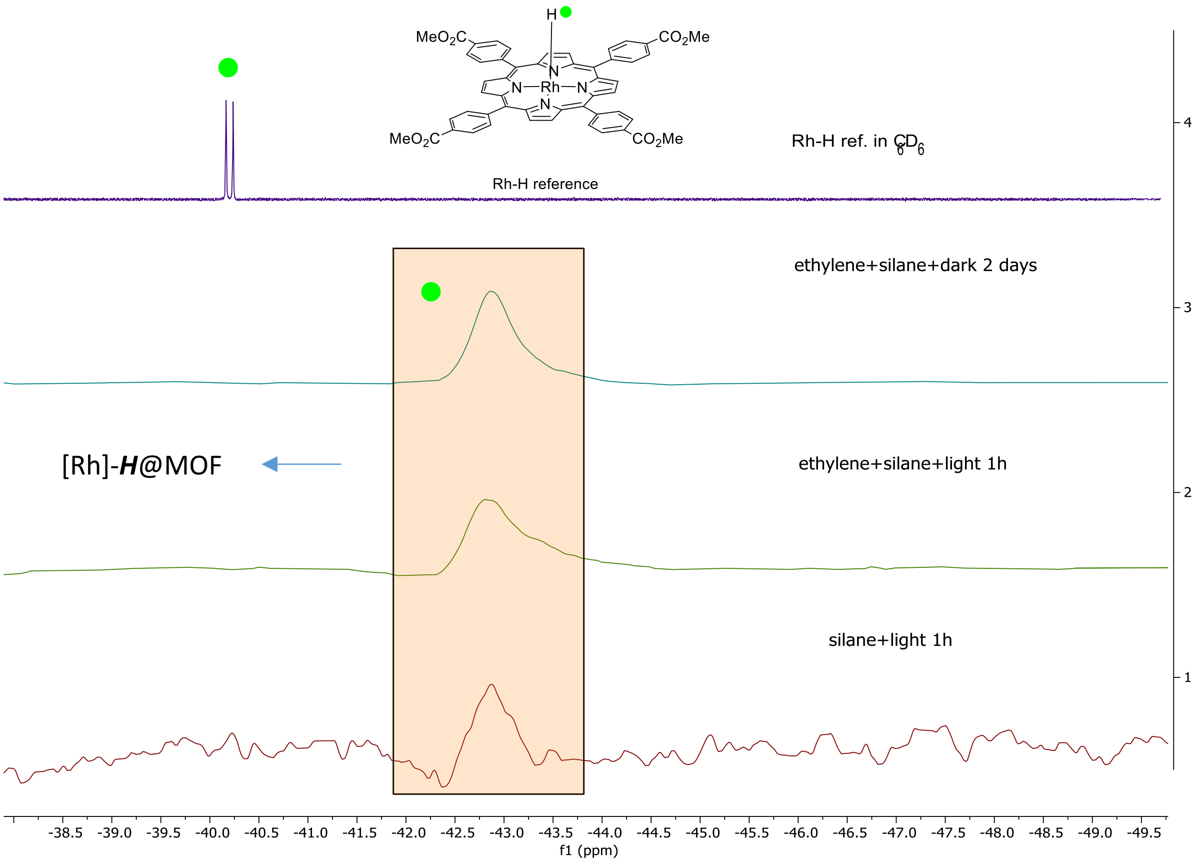

Figure S46. Comparison of solid-state NMR data shown in Fig. 8B with an authentic reference sample of the molecular analogue which was synthesized according to our previously reported procedure.[61]

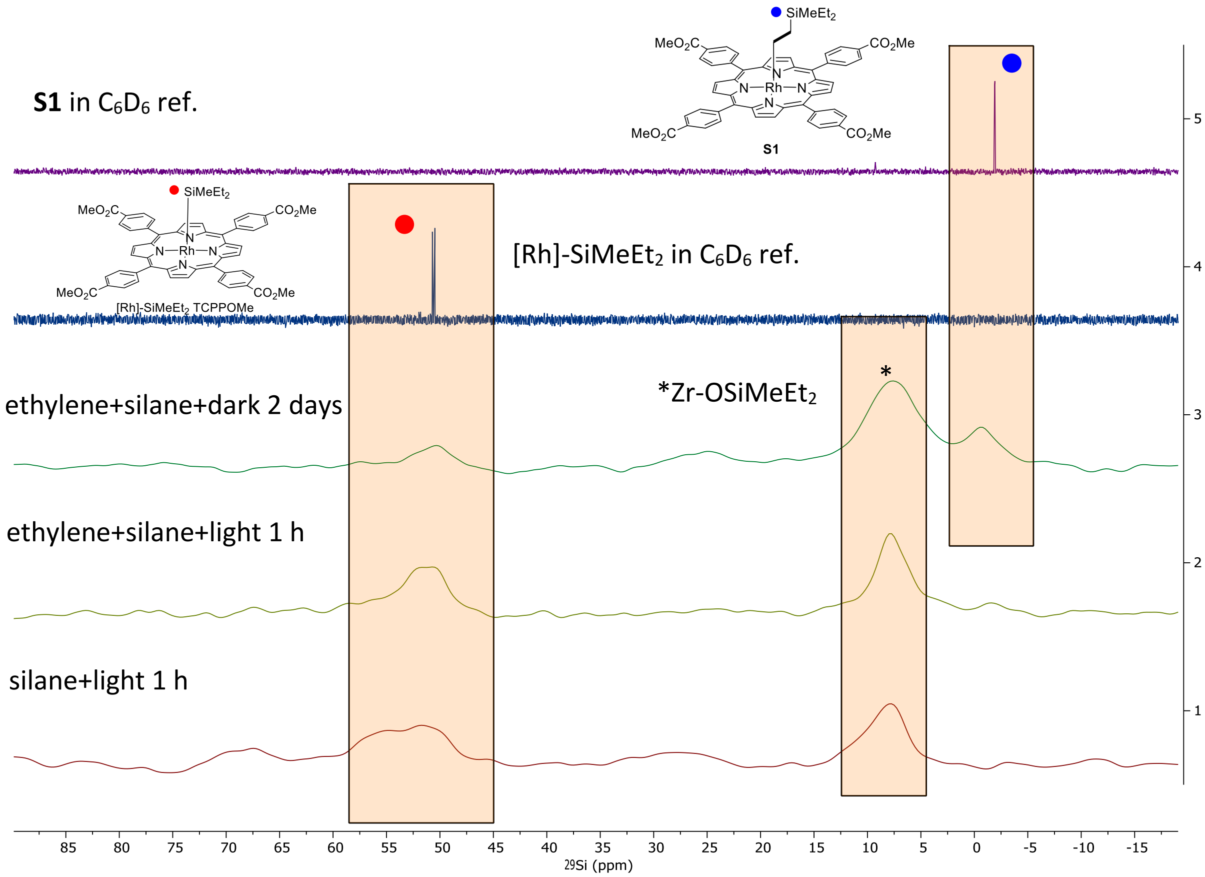

Figure S47. Stacked $^{29}Si\{^{1}H\}$ ssNMR spectrum of MOF catalyst exposed to different reaction conditions as well as the [Rh]-$SiMeEt_2$ and **S1** reference compounds.

*Note: For preparation and characterization data of **S1**, please see section 25. For preparation and characterization data of [Rh]-$SiMeEt_2$, please see our previous report.*[61]

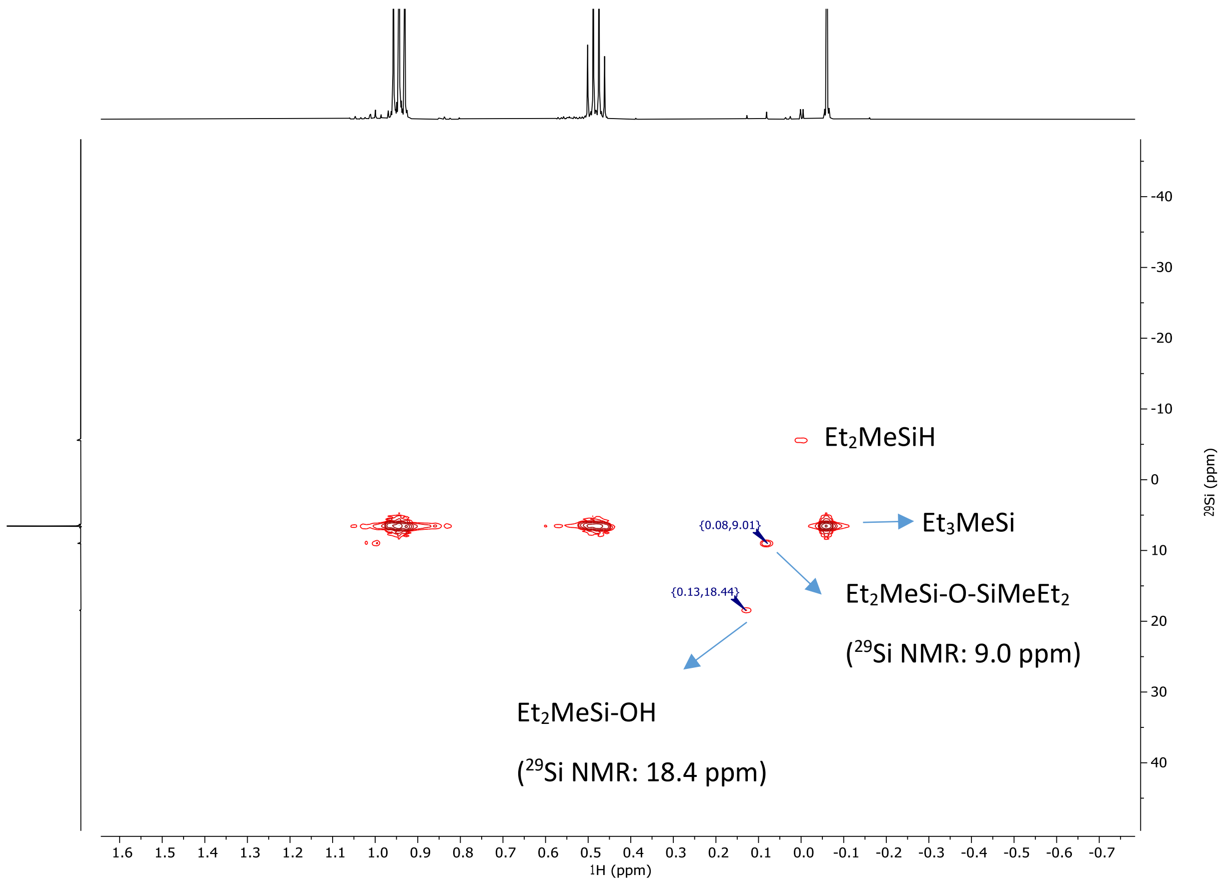

Figure S48. ^{1}H-^{29}Si HMBC spectrum of reaction mixture of hydrosilylation reaction with ethylene and diethylmethylsilane catalyzed by Rh(Me)-**3** under 390 nm LED for 20 h at room temperature

15 Radical Trapping Experiments with PBN

To further confirm the presence of suspected reaction intermediates with unpaired spins, the spin trapping reagent N-tert-butyl-α-phenylnitrone (PBN) was added to the reaction and the resulting mixture was probed by EPR spectroscopy.

15.1 With $PhMe_2Si$-H

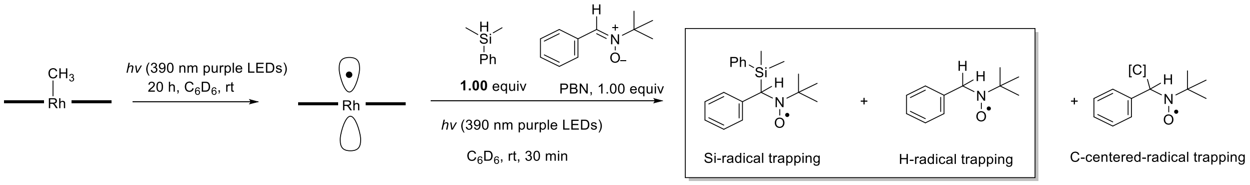

In an argon-filled glovebox, Rh(Me)-**3** (1.0 mg, 0.070 µmol, 0.37 mol%) and C_6D_6 (1.0 mL), were added to a 9 mL VWR glass vial containing a cross-type stir bar (5 x 10 mm). Then the vial was sealed with a screw cap that contains a septum, and transferred out of the glovebox. The reaction mixture was subjected to sonication for 1 minute in order to evenly suspend the MOF crystallites throughout the solvent. After that, Rh(Me)-**3** was subjected to photolysis to produce active catalyst Rh(II)-**3** with a 390 nm purple LED for 20 h at room temperature and a stirring rate of 500 rpm, where the reaction was cooled by in-house compressed air flow.

The reaction vial was transferred into glovebox, $PhMe_2Si$-H (29.2 µL, 191 µmol, 270 equiv) and PBN (33.8 mg, 191 µmol, 270 equiv) were added to the 9 mL glass vial. Then the vial was sealed with a screw cap that contains a septum, and transferred out of the glovebox. The reaction mixture was subjected to sonication for 1 minute in order to evenly suspend the MOF crystallites throughout the solvent. The reaction was stirred at 500 rpm at room temperature and irradiated with a 390 nm purple LED for 30 min, where the reaction was cooled by in-house compressed air flow. The reaction vial was subsequently subjected to centrifugation at 4500 rpm for 5 min (to ensure that the MOF particles settle down on the bottom of reaction vial). The reaction vial was subsequently transferred back to the glovebox, and around 0.5 mL solution was added into an EPR tube. For the EPR spectrum and its simulation, please see Figure S49 and Table S13.

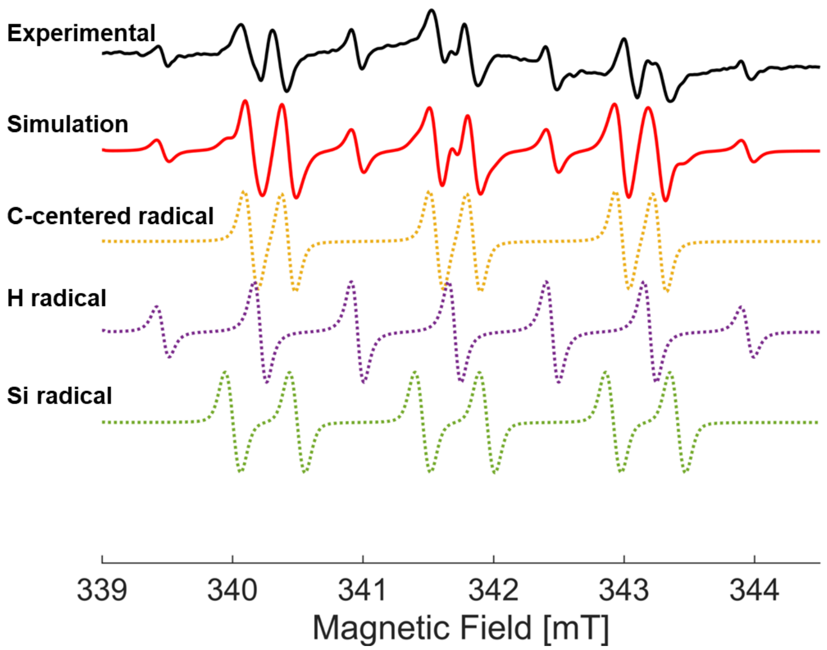

Figure S49. Experimental (black) and computer simulation (red) of the CW-EPR spectrum of the interaction between Rh(II)-**3** and $PhMe_2Si$-H in presence of the spin trap PBN at room temperature. The simulation is composed by the weighted contribution of three radical species shown in yellow, violet and green dotted traces.

Table S13. Spin Hamiltonian parameters employed for the simulation of the CW-EPR spectrum reported of PBN-trapped spectra. The assignment of the different species was performed according to previous literature reports.[69-71]

	**Species**	**Weight**	g_{iso}	$^{N}A_{iso}$ **(MHz)**	$^{H}A_{iso}$ **(MHz)**
Simulated	C-centered radical	65(5) %	2.010(1)	41(2)	7(2)
	H radical	25(5) %	2.010(1)	42(2)	21(2)
	Si radical	10(3) %	2.010(1)	41(2)	12(2)

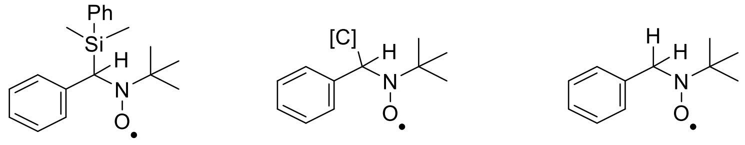

Si radical trapping

C-centered radical trapping

H radical trapping

15.2 With Et_3Si-H

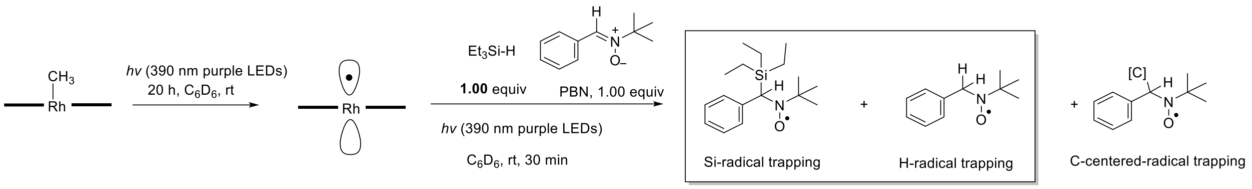

An identical experimental procedure was used compared to section 15.1. except that $PhMe_2Si$-H was replaced with Et_3Si-H (30.5 µL, 191 µmol, 270 equiv). For the resulting EPR spectrum, its simulation and interpretation, see Figure S50 and Table S14.

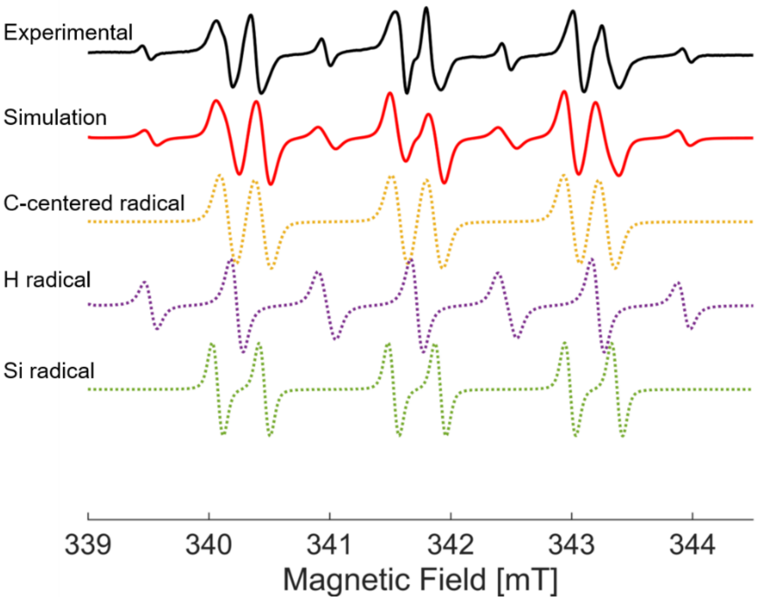

Figure S50. Experimental (black) and computer simulation (red) of the CW-EPR spectrum of the interaction between Rh(II)-**3** and Et_3Si-H in presence of the spin trap PBN at room temperature. The simulation is composed by the weighted contribution of three radical species shown in yellow, violet and green dotted traces.

Table S14. Spin Hamiltonian parameters employed for the simulation of the CW-EPR spectrum reported of PBN-trapped spectra. The assignment of the different species was performed according to previous literature reports.[69-71]

	**Species**	**Weight**	**g_{iso}**	**$^{N}A_{iso}$ (MHz)**	**$^{H}A_{iso}$ (MHz)**
Simulated	C-centered radical	65(5) %	2.010(1)	41(2)	7(2)
	H radical	25(5) %	2.010(1)	42(2)	21(2)

Si radical	10(3) %	2.010(1)	41(2)	12(2)

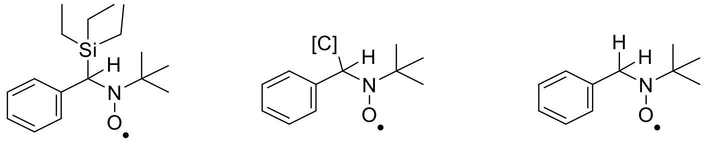

Si radical trapping   C-centered radical trapping   H radical trapping

16 Isotopic Labelling of Silane

To determine whether the proton that is originally associated with the silane reagent [Si]-***H*** is incorporated into the hydrosilylated product, ethylene hydrosilylation was carried out with deuterated silane [Si]-***D***.

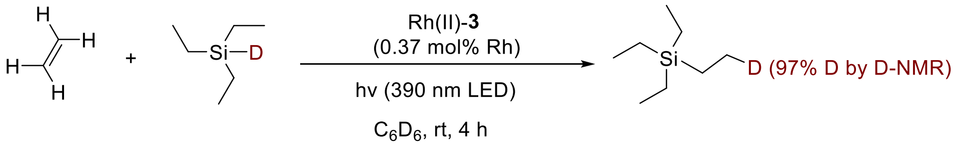

In an argon-filled glovebox, Rh(Me)-**3** (1.0 mg, 0.070 µmol, 0.37 mol%) and C_6D_6 (1.0 mL), were added to a 9 mL VWR glass vial containing a cross-type stir bar (5 x 10 mm). Then the vial was sealed with a screw cap that contains a septum, and transferred out of the glovebox. The reaction mixture was subjected to sonication for 1 minute in order to evenly suspend the MOF crystallites throughout the solvent. After that, Rh(Me)-**3** was subjected to photolysis to produce active catalyst Rh(II)-**3** with a 390 nm purple LED for 20 h at room temperature and a stirring rate of 1000 rpm, where the reaction was cooled by in-house compressed air flow.

The reaction vial was transferred into glovebox, Et_3Si-D (15.2 µL, 95.4 µmol, 1.00 equiv) was added to the 9 mL glass vial. Then the vial was sealed with a screw cap that contains a septum, and transferred out of the glovebox. The reaction mixture was subjected to sonication for 1 minute in order to evenly suspend the MOF crystallites throughout the solvent. Ethylene was then bubbled into the reaction vial for 1 minute in order to saturate the solvent with ethylene. To prevent oxygen leaking into the reaction mixture as ethylene was introduced, the vent needle that released excess ethylene from the reaction vial was connected to an inert gas manifold which was connected to an oil bubbler. Following the addition of ethylene, the reaction vial was swiftly transferred into a glovebox to replace the punctured septum cap with a screw cap. After removal from the glovebox, the reaction was stirred at 500 rpm at room temperature and irradiated with a 390 nm purple LED for 4 h, while the reaction was cooled by in-house compressed air flow. The reaction vial was subsequently subjected to centrifugation at 4500 rpm for 5 min (to ensure that the MOF particles settle down on the bottom of reaction vial), and transferred into a glovebox. The C_6D_6 solution was then carefully removed using a syringe, and transferred into an NMR tube.

From NMR analysis, the Et_3Si-D starting material and the hydrosilylation product are assigned in Figure S51, and from D-NMR, the D ratio in terminal position of the product is around 97% (Figure S52), confirming the D source is from D-$SiEt_3$.

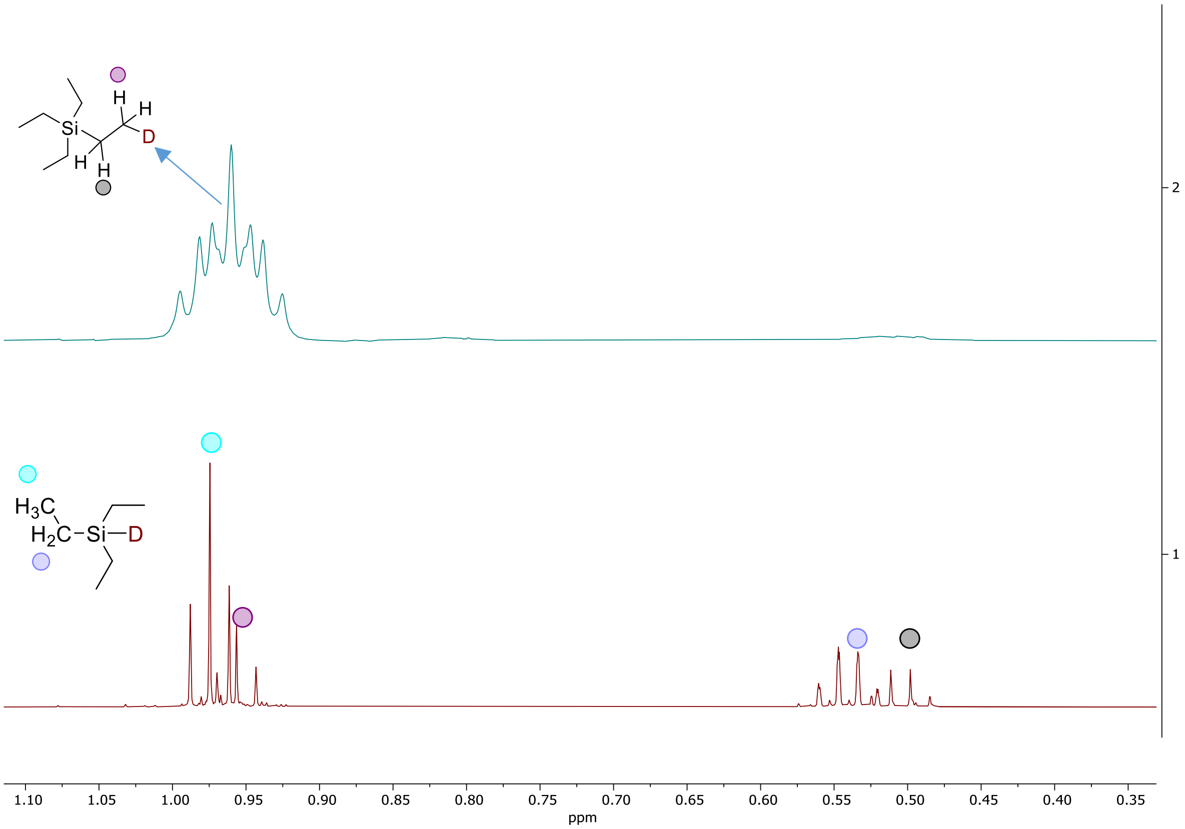

Figure S51. Stacked ^2H (top) and ^1H (bottom) NMR spectra of the reaction mixture in C_6D_6.

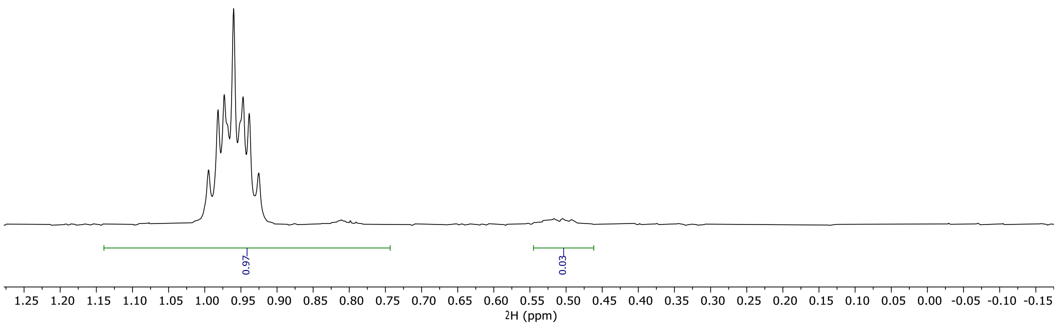

Figure S52. ^{2}H-NMR spectrum of the reaction mixture in C_6D_6.

17 Stability of MOF Linker towards Attack by Radical Intermediates

Molecular dynamics simulations that were carried out to interrogate the fate of **7** predicted that around 10 % of the **7** undergoes addition to the porphyrin ligand. Since turnover numbers >200 could be observed for Rh(II)-3 and the catalyst could be repeatedly recycled without performance loss, we expect that i) either our calculations overestimate the probability of the addition of **7** to the porphyrin ring or ii) that such addition is reversible on timescales longer than the 50 ps investigated in molecular dynamicas simulations. We set out to test, however, if any experimental evidence for the addition of **7** to the porphyrin ring of the MOF linker could be obtained.

Experimental procedure:

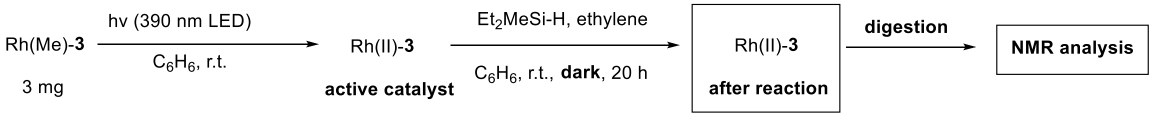

After completion of the thermal hydrosilylation reaction, the liquid portion of the reaction mixtures was removed via argon flow. The remaining solid was digested and the resulting solution subjected to NMR analysis using a 600 MHz NMR instrument equipped with a cryoprobe. The experiment was carried out in duplicate in order to evaluate two different digestion procedures (A and B). Condition A: 0.06 mL of NaOD (40 wt % solution in D_2O) and 0.5 mL CD_3OD. Condition B: 0.02 mL $NaHCO_3$ (1M solution in D_2O), 0.1 mL D_2O and DMSO-d6.

17.1 Digestion Condition A

Signals in the region of 8.7 ppm (Figure S53) can be assigned to the pyrrolic C–H bonds in (porphyrin)Rh(III)-X. The presence of multiple peaks in this region indicates the presence of (porphyrin)Rh(III)-X for which different X-type ligands are attached to the Rh center. We speculate that (porphyrin)Rh(III)-X with X = OD or X = OCD_3 were formed during digestion process. The signal at a chemical shift of 8.53 ppm can be assigned to the (porphyrin)Rh(I) anion.[72] The formation of (porphyrin)Rh(I) has previously been reported to take place when (porphyrin)Rh(III)-H is deprotonated under basic conditions. Since (porphyrin)Rh(III)-H is a known intermediate in the thermal hydrosilylation reaction, the formation of (porphyrin)Rh(I) under the basic MOF digestion conditions can readily be accounted for. Signals at chemical shifts of 8.4 and 8.2 ppm can be assigned to the aryl group attached to the *meso*-position of the porphyrin backbone. In addition, signals belonging to toluene were also observed in this region of the spectrum. Toluene is during the generation of the active Rh(II)-**3** catalyst in C_6H_6.[61] A residue of the reaction solvent of thermal hydrosilylation (benzene) was observed at chemical shift 7.25 ppm.

In additions to the signals assigned above, there are minor signals (highlighted by blue boxes) which cannot be assigned to a specific species. It is possible that these minor species originate from the formation of less symmetrical derivatives due to the addition of **7** to the porphyrin ring. Alternatively, the basic decomposition conditions necessary to enable solution NMR analysis may have caused partial decomposition of the rhodium-containing porphyrin ligand. While a range of additional NMR data was collected ($^{13}C\{^{1}H\}$, $^{29}Si\{^{1}H\}$, ^{1}H-^{13}C HSQC, HMBC, ^{1}H-^{1}H COSY and ^{1}H-^{29}Si HMBC) structural assignment of the species that give rise to the signals in blue boxes was not possible due to their low concentration.

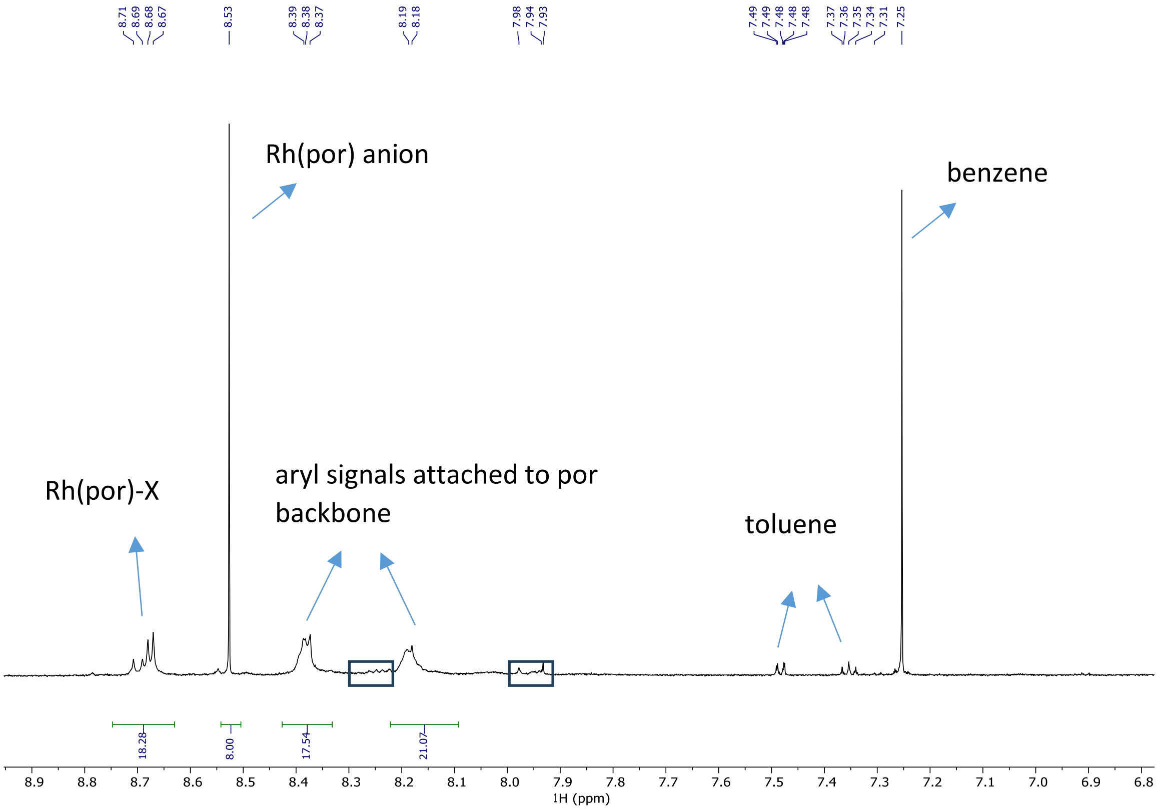

Figure S53. ^{1}H NMR spectrum of the Rh(II)-**3** catalyst after reaction in dark that was digested using 0.06 mL NaOD (40 wt% in D_2O) and 0.5 mL CD_3OD. The aryl region was presented in order to show the porphyrin signals.

17.2 Digestion Condition B

In an effort to determine whether the minor species observed for digestion condition A were caused by the digestion process itself, we adopted second digestion protocol.[73]

A clear change in the aryl region of the ^{1}H NMR spectrum was observed upon switching from digestion condition A to digestion condition B (Figure S54). In addition, very weak peaks were now also observed in the negative ppm range (Figure S55), which provides direct information on ligands attached to the rhodium center. In order to improve the signal-to-noise ratio for the minor signals observed, we employed a one hour acquisition time for ^{1}H NMR spectra shown in Figure S54 and Figure S55.

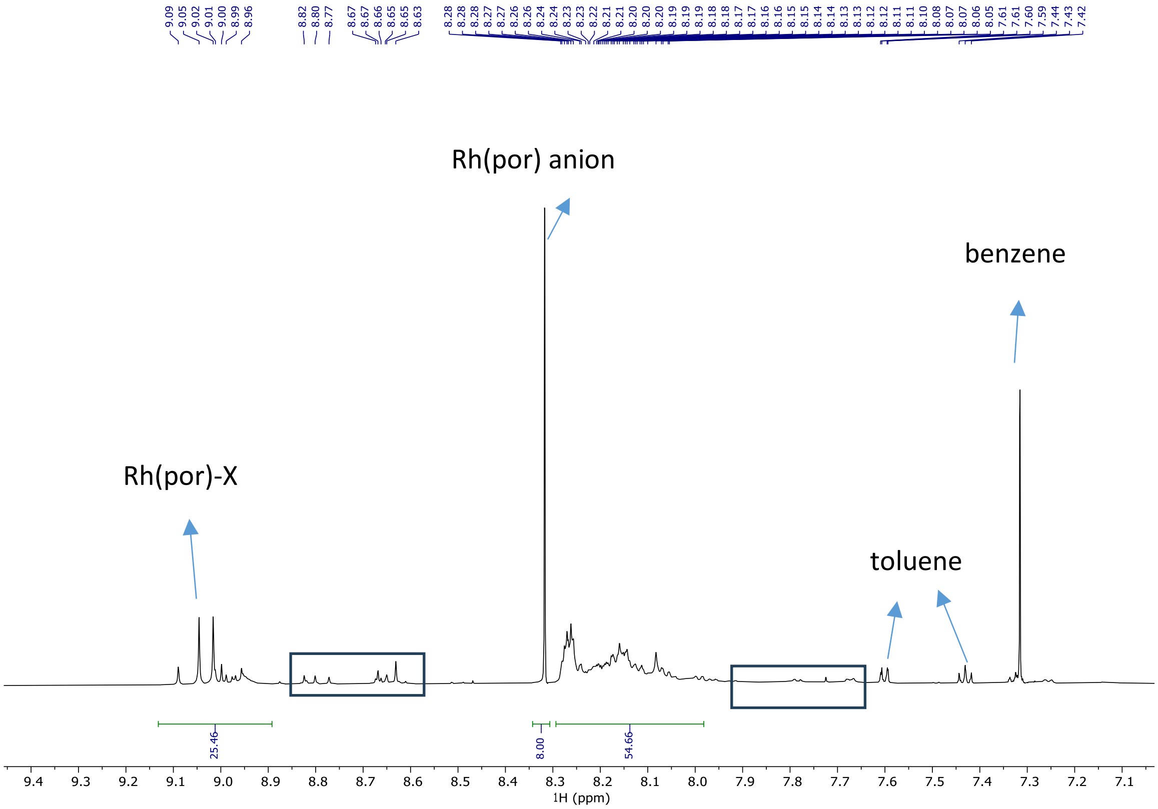

Figure S54. ^{1}H NMR spectrum of a sample of Rh(II)-**3** recovered after thermal hydrosilylation which was digested using condition B. The aryl region is presented in order to clearly show the porphyrin signals. The slight chemical shift differences compared to Figure S53 can be attributed to the use of different deuterated NMR solvents.

In addition to (porphyrin)Rh(III)-X and (porphyrin)Rh(I), which were also observed for condition A, additional aryl signals (highlighted by blue box) in the chemical shift range of 8.3 to 8.0 ppm. An increase in the number of species present is also apparent from the more complex pattern that is derived from aryl signals attached to the *meso*-position of the porphyrin backbone. These observations are consistent with the presence of linker molecules in which the porphyrin backbone has undergone modification, which would lead to a less symmetrical structure.

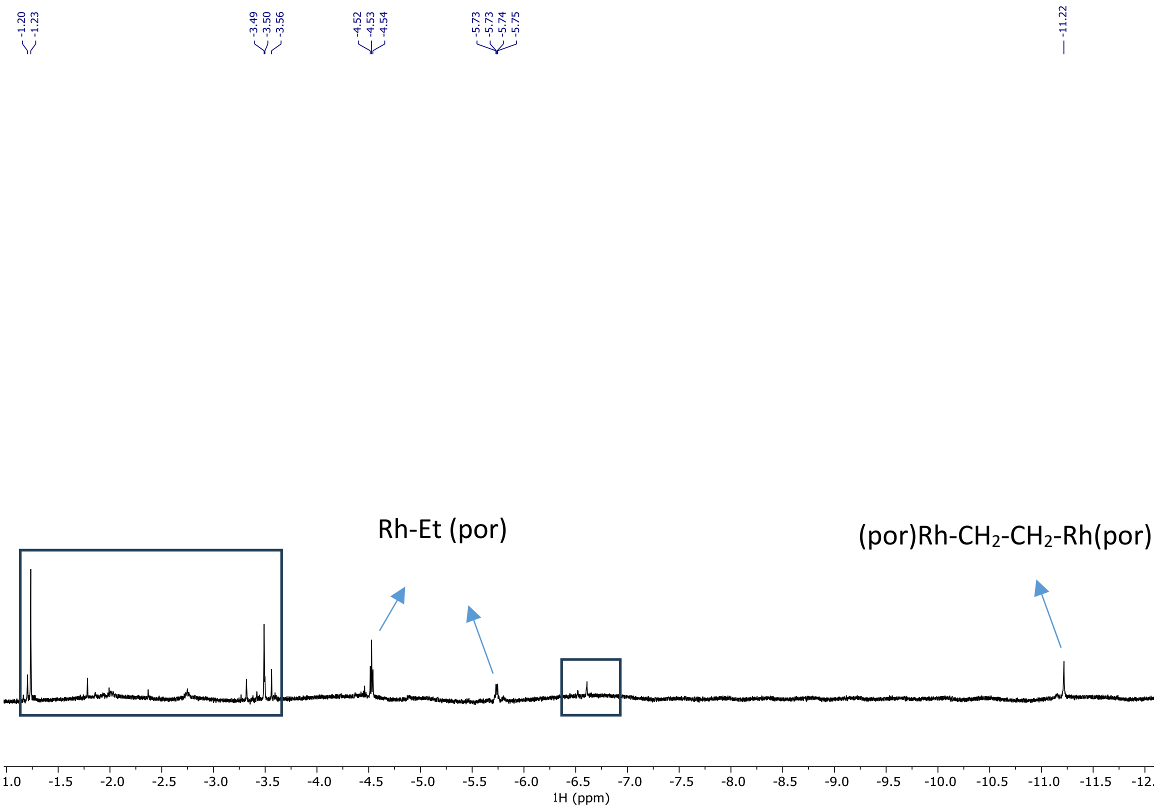

Figure S55. ^{1}H NMR spectrum of a sample of Rh(II)-**3** recovered after thermal hydrosilylation which was digested using condition B. The figure shows the negative ppm range of the spectrum in which species bound the rhodium show characteristic peaks.

As shown in Figure S55, trace amounts of (porphyrin)Rh(III)-Et and (porphyrin)Rh(III)-CH_2-CH_2-Rh(III)(porphyrin) could be detected.[61] We speculate that (porphyrin)Rh(III)-Et and (porphyrin)Rh(III)-CH_2-CH_2-Rh(III)(porphyrin) may have been formed during the digestion process since the formation of both species were previously reported for molecular Rh(II)porphyrins while an analogous reactivity has never been observed for a MOF-based Rh(II)poryphin.[61, 74-76] In addition, minor signals (in blue boxes) were also observed which could not be structurally assigned even when additional NMR data was collected (^{13}C {^{1}H}, ^{29}Si {^{1}H}, ^{1}H-^{13}C HSQC, HMBC, ^{1}H-^{1}H COSY, ^{1}H-^{29}Si HMBC and ^{1}H-^{103}Rh HMBC).

In summary, despite significant efforts to determine whether addition of **7** to the MOF linker takes place under the reaction conditions of thermal hydrosilylation, only very tentative evidence for such a process could be obtained.

18 Computational Details

All calculations were performed with a development version of ORCA.[77]

Before introducing the levels of theories employed, we briefly comment on the truncation of the MOF model. The initial structural model of the MOF was built from the experimental single crystal XRD structure of PCN-224 (Ni) by Zhou *et al.*[78] (CCDC number: 919444), where the Ni(II) metal center was changed to Rh(II) manually. For all calculations that involve only one rhodium site, the porphyrin linker was taken out of the MOF, and the four carboxylate groups of the linker were saturated with methyl groups. Thus, for example, Rh(II)-**3** was calculated as **18**. For all calculations that involve two rhodium sites, two adjacent porphyrin rhodium units are included in the calculations, where the porphyrin planes form a *ca*. 90° angle with respect to each other (see, e.g. Fig. 9 D-E). The two Zr_6O_8 nodes that join the two porphyrin ligands are retained, while all other Zr_6O_8 nodes that are connected to the two porphyrin ligands are replaced by methyl groups. Following literature suggestions,[79] we added one hydrogen atom to four of the eight μ_3-oxygen atoms of the Zr_6O_8 node, and then added enough hydrogen atoms to the 12 μ_1-oxygen atoms of the node to restore the correct charge and valence state of the node; as a result, 6 of the μ_1-oxygen atoms were turned into OH groups, and the other 6 were treated as coordinated H_2O ligands. The OH and H_2O ligands form extensive hydrogen bonds with each other, meaning that they cannot freely rotate to stabilize the transient charges during the hydrosilylation reaction. All other carboxylate ligands of the two Zr_6O_8 nodes were replaced by formates. To keep the correct distance between the two Rh centers, all carboxylate oxygen atoms of the porphyrin ligands were fixed at the single crystal XRD positions of Rh(II)-**3**.

To ensure that representative conformers are used in the calculations, all species that possess flexible groups (such as ethyl groups) in their axial ligands were subjected to conformational search at the GFN2-xTB[80] level of theory prior to the DFT geometry optimizations, using ORCA's newly developed conformational search tool, GOAT (to be published elsewhere). The conformations of the *p*-methoxycarbonylphenyl groups were sampled while calculating **18**, and unless the flexibility of the axial group necessitates a further conformational search, the conformations of the *p*-methoxycarbonylphenyl groups were not searched again in the calculations of subsequent species. The conformational searches of transition states (TSs) were done by first optimizing an arbitrary conformer of the TS using DFT (vide infra), freezing the lengths of the forming/breaking bonds, and performing conformational search of the remaining degrees of freedom using GFN2-xTB. In all cases, the conformer that has the lowest GFN2-xTB energy was used in the subsequent DFT calculations. No re-ranking of the conformers at the DFT level, nor conformational averaging, was done.

Geometry optimizations and frequency calculations were conducted at the BP86[81-82]-D3[83-84]/SARC-ZORA-TZVP[85](Rh)/ZORA-def2-TZVP[86](Si)/ZORA-def2-SVP[86](C,H,O,N) level of theory, with scalar relativistic effects accounted for at the ZORA level.[87-89] The RI approximation[90] was used to speed up the calculations, using SARC/J[91] as the auxiliary basis set. Solvation effects were considered at the CPCM level,[92] with benzene as solvent. The DefGrid3 grid[93] was used throughout to avoid spurious imaginary frequencies. Similar levels of theory have successfully been used in the optimization of the structures of porphyrin rhodium complexes, giving results that compared favorably with experiment.[94-95] The number of imaginary frequencies of all structures, except for those with two porphyrin rhodium units ("dimers", where

imaginary frequencies are neither avoidable nor meaningful, due to the oxygen atoms being constrained), have been verified to be the desired ones (0 for local minima, 1 for TSs).

Single point energies were calculated at the DLPNO-CCSD(T)[96-97]/SARC-ZORA-TZVP (Rh)/ZORA-def2-TZVP(C,H,O,N,Si) level of theory. Importantly, the reference wavefunction was generated using the ωB97X[98] functional, instead of Hartree-Fock (HF), since HF exhibits severe spin contamination for the systems studied herein. The RIJCOSX approximation[93, 99-101] was used to speed up the SCF part of the calculations, using SARC/J as the auxiliary basis set. The SMD solvation model[102] was used in all single point energy calculations. Considering the good accuracy of ωB97X for the wavefunctions of the presently studied systems, molecular orbital plots (including natural bonding orbital (NBO) plots, calculated with the help of NBO 7.0[103]) were all generated at the ωB97X/SARC-ZORA-TZVP (Rh)/ZORA-def2-TZVP(C,H,O,N,Si) level of theory.

For the calculation of dimer models, DLPNO-CCSD(T) proved to be unaffordable. Therefore, the Gibbs free energy of each dimer AB, consisting of a porphyrin rhodium complex where the reaction of the silane and ethylene takes place, A, and a porphyrin rhodium radical which only serves as an electron reservoir, B, were calculated as follows:

$$\begin{aligned} G(AB, \mathrm{DLPNO-CCSD(T)//BP86-D3}) \\ &\approx E(AB, \mathrm{\omega B97M-V//BP86-D3}) \\ &+ \big(G(A, \mathrm{DLPNO-CCSD(T)//BP86-D3}) - E(A, \mathrm{\omega B97M-V//BP86-D3})\big) \\ &+ \big(G(B, \mathrm{DLPNO-CCSD(T)//BP86-D3}) - E(B, \mathrm{\omega B97M-V//BP86-D3})\big) \end{aligned}$$

where $E(AB, \mathrm{\omega B97M-V//BP86-D3})$ stands for the single point energy at the ωB97M-V[104]/SARC-ZORA-TZVP(Rh)/ZORA-def2-TZVP(C,H,O,N,Si) level at the BP86-D3 geometry, and G is the Gibbs free energy. Note that the single point energies as well as the Gibbs free energy corrections of A and B are evaluated at their respective equilibrium geometries, instead of at their geometries in the AB dimer. In this way, we were able to get reasonable estimates of the Gibbs free energy correction of the AB dimer without doing costly frequency calculations of the AB dimer.

To ensure that we have found the globally optimal wavefunction at the ωB97M-V level, for the dimer models we always prepared two initial guess wavefunctions, one (broken symmetry singlet) where A and B are both in their neutral charge states, and the other (closed shell singlet) where an electron had transferred from A to B. The lower one of the two energies were reported. At the BP86-D3 level (which we use for geometry optimization), the closed-shell singlet wavefunction always has a higher energy than the broken symmetry wavefunction, and when ωB97M-V predicts a closed shell singlet (i.e. transfer of one full electron from A to B), BP86 predicts that only a fraction of one electron (~0.6 electrons) is transferred from A to B, and the wavefunction remains a broken symmetry singlet. Thus, we used the broken symmetry wavefunction for geometry optimization throughout. We are fully aware that the fractional charge transfer may be a defect of the BP86 functional, due to its self-interaction error; however, we could not afford to use hybrid functionals for the geometry optimization of our dimer models. Moreover, since the functional we used for single point energy calculations (ωB97M-V) predicts an integer number of transferred electrons, any defect of BP86 should have minimal influence on the calculated free energy results.

EPR calculations were conducted at the ωB97M-V/x2c-TZVPPall-2c[105](decontracted) level of theory. Scalar relativistic effects were treated at the spin free exact two component (sf-X2C)[106-109] level of theory, and

spin-orbit coupling (SOC) effects were described by the spin-orbit mean field (SOMF) method, with full account of picture change effects.[110] The decontraction of the x2c-TZVPPall-2c basis set was found to be necessary for accurate EPR hyperfine couplings (A values), [111] since x2c-TZVPPall-2c is generally not flexible enough in the core region for hyperfine coupling calculations. Solvation effects were described at the SMD level (solvent = benzene).

Excited states were computed at the ωB97X/SARC-ZORA-TZVP(Rh)/ZORA-def2-TZVP(C,H,N,O,Si) level of theory, with solvation effects described at the SMD level (solvent = benzene). The Tamm-Dancoff approximation (TDA) was used.

Polarizability (which was used to estimate the dielectric constant of the MOF) and dipole moment calculations were performed at the PBE0[112]/ma-def2-TZVPP[113-114] level of theory, with solvation effects described at the SMD level (solvent = benzene). The def2-TZVPPD[114-115] basis, which is generally believed to be suitable for dipole moment and polarizability calculations, led to severe SCF convergence difficulties; we were thus forced to use the slightly smaller ma-def2-TZVPP basis set.

MD simulations were performed using a re-fitted version of the GFN2-xTB method, to be detailed near the end of the computational section.

18.1 Electronic Structure of the Rh-H-Si Complex (**6**)

18.1.1 Analysis of Frontier Molecular Orbitals

As mentioned in the main text, **6** possesses a non-conventional, 3c-3e Rh-H-Si bond. Three AOs are expected to be involved in the bonding, namely Rh 4d_{z2}, H 1s and Si sp^3. They should linearly combine to form three molecular orbitals: one bonding orbital σ(Rh-H-Si), one essentially non-bonding orbital n(Rh-H-Si), and one anti-bonding orbital σ*(Rh-H-Si) (Figure S56):

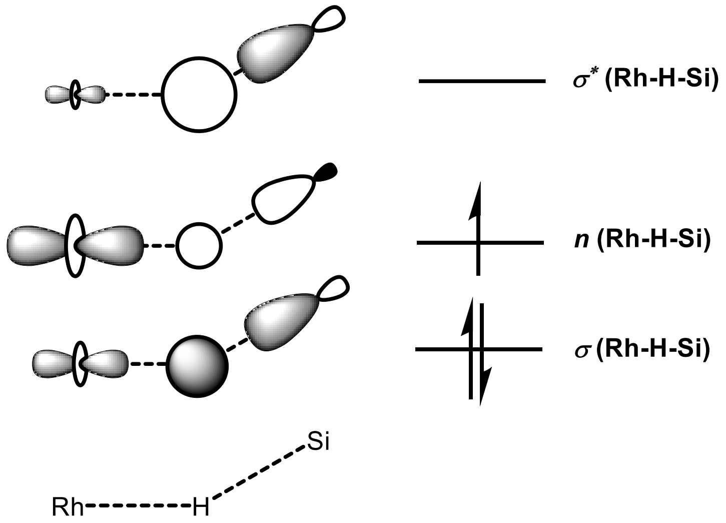

Figure S56. Schematic representation of the frontier orbitals of the Rh-H-Si fragment.

The orbitals σ(Rh-H-Si) and σ*(Rh-H-Si) mix extensively with orbitals of similar energies such that no canonical molecular orbital is dominated by Rh-H-Si bonding or Rh-H-Si antibonding character. However, a canonical orbital can be found that exhibits significant n(Rh-H-Si) character (Figure S57).

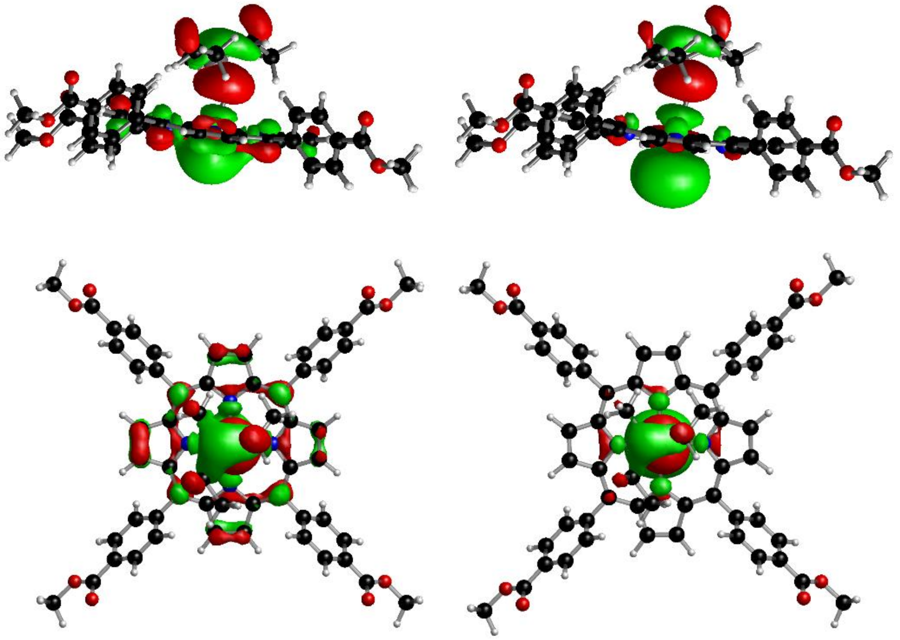

Figure S57. The alpha (left) and beta (right) canonical n(Rh-H-Si) orbitals of **6** (ωB97X/SARC-ZORA-TZVP(Rh)/ZORA-def2-TZVP(C,H,N,O,Si)). Upper panel: side view; lower panel: top view.

The alpha n(Rh-H-Si) orbital lies just below the two highest porphyrin π orbitals, and the beta n(Rh-H-Si) orbital lies just above the two lowest porphyrin π* orbitals (Table S15). Note that here we have used the irreducible representation (irrep) symbols under the D_{4h} group to label the four frontier ligand orbitals (the "Gouterman orbitals"[116]), as is conventionally done for porphyrin complexes,[95] even though the actual complex has only C_1 symmetry.

Table S15. Frontier orbital compositions and energies of **6**

Orbital	Alpha composition & energy	Beta composition & energy
HOMO-2	n(Rh-H-Si) (-8.08 eV)	Rh d_{xz} (-8.30 eV)
HOMO-1	porphyrin π (a_{1u}-like) (-7.25 eV)	porphyrin π (a_{1u}-like) (-7.30 eV)
HOMO	porphyrin π (a_{2u}-like) (-7.03 eV)	porphyrin π (a_{2u}-like) (-7.15 eV)
LUMO	porphyrin π* (e_g-like) (-1.22 eV)	porphyrin π* (e_g-like) (-1.19 eV)
LUMO+1	porphyrin π* (e_g-like) (-1.21 eV)	porphyrin π* (e_g-like) (-1.18 eV)
LUMO+2	*p*-methoxyphenyl π* (0.35 eV)	n(Rh-H-Si) (-0.05 eV)

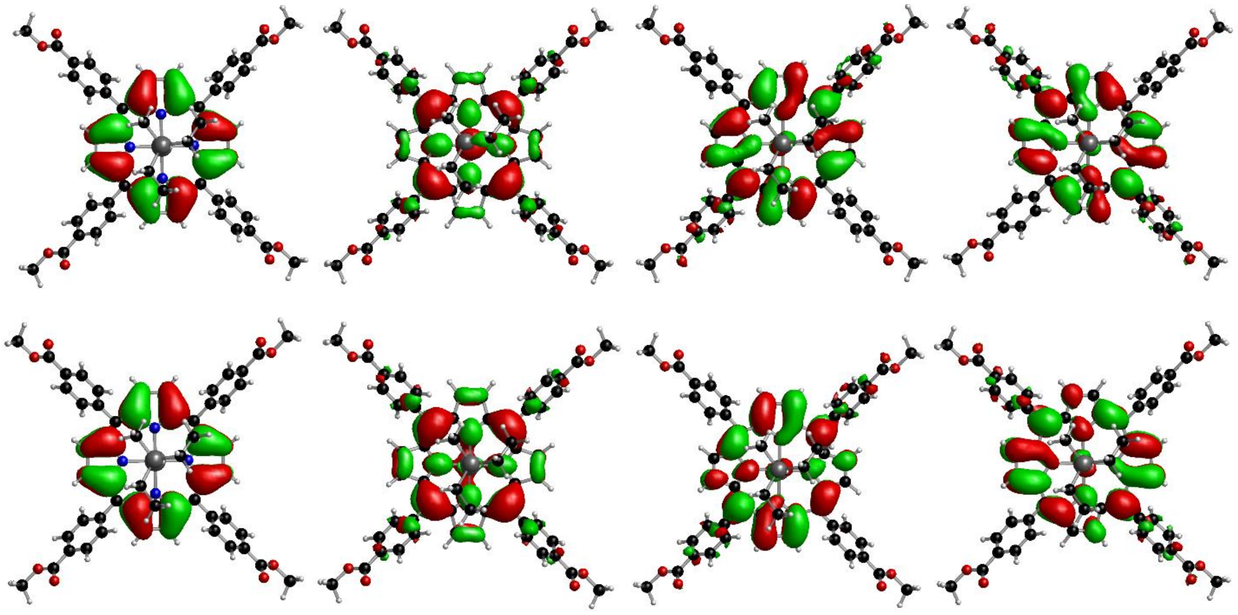

Figure S58. Frontier orbitals of **6**. Upper panel: alpha orbitals; lower panel: beta orbitals. From left to right: HOMO-1, HOMO, LUMO, LUMO+1.

From Figure S57, it is obvious that n(Rh-H-Si) also mixes appreciably with the porphyrin orbitals, as well as the Si–C and C–H orbitals of the silyl group. The "porphyrin part" of the n(Rh-H-Si) orbital has a_{2u} symmetry, and therefore resembles the HOMO (Figure S58). This is due to the fact that the n(Rh-H-Si) has approximate σ symmetry along the Rh-H axis, and among the four Gouterman orbitals of the porphyrin ligand, only the a_{2u} orbital possess σ symmetry along the same axis. Therefore, even though n(Rh-H-Si) is not the HOMO of the system, removing one electron from the HOMO (by either single electron oxidation or light excitation) may still result in electron depletion of the n(Rh-H-Si) orbital, since the latter can mix with the HOMO due to matching spatial symmetry. On the contrary, adding one electron to the LUMO (or LUMO+1) does not immediately lead to an increase of the n(Rh-H-Si) occupation number, due to the LUMO/LUMO+1 orbitals having e_g symmetry.

The contributions of σ(Si-H) and Rh d_{z2} orbitals to this orbital are comparable in magnitude in the alpha n(Rh-H-Si) orbital, and the latter dominates over the former in the beta n(Rh-H-Si) orbital. Therefore, frontier orbital theory predicts that the major direction of ethylene attack on **6** should be from below (pathway (a) in Figure S59), yielding the ethylene adduct **13** reversibly. A naïve calculation of the coordination of **6** with C_2H_4 in implicit benzene solvent yields a slightly unfavorable binding Gibbs free energy of ΔG = 2.2 kcal·mol^{-1}. No barrier was found for the binding process. However, **6** also coordinates favorably with benzene with ΔG = – 2.5 kcal·mol^{-1}. Thus, displacing the coordinated benzene with C_2H_4 is unfavorable by 4.7 kcal·mol^{-1} at room temperature. Despite this, as C_2H_4 is a gas, lowering the temperature may make the displacement less entropically unfavorable and eventually lead to measurable EPR concentrations of **13**, as experimentally observed in Fig. 7.

Two alternative reaction pathways would be the attack of the middle (pathway (b)) and top (pathway (c)) lobes of the n(Rh-H-Si) orbital (Figure S59). The former leads to the experimentally observed products **5** and **7**, but with a very high barrier (**6-TS-b**), possibly due to steric congestion of the transition state. The latter leads to the same products with a lower barrier (**6-TS**), but still too high for reacting at room

temperature. We also tried several alternative pathways, including: (d) ethylene reacts with **6** through a concerted Si…H…C…C four-membered ring transition state (**6-TS-d**); (e) silane reacts with the Rh-ethylene complex with a concerted Si…H…C…C four-membered ring transition state (**6-TS-e**); (f) silane reacts with the Rh-ethylene complex with a linear Si…H…C transition state (**6-TS-f-1**), yielding a rhodium ethyl complex and the silyl radical **8**; ethyl abstraction from rhodium by **8** (**6-TS-f-2**) yields the final product. However, for (d) many possible orientations of the ethylene substrate invariably led to **6-TS-b** after transition state optimization, which strongly suggests that pathway (d) does not exist. For (e), we only located a transition state leading to the Rh-Et complex and the ·$SiMeEt_2$ radical (**8**), which is the first transition state of pathway (f) (**6-TS-f-1**), and the latter two species cannot react to form the product Et_3SiMe due to the inertness of the Rh-C bond (transition state searches led back to **6-TS-f-1**). Despite the fact that the activation free energy of pathway (f) (1.1 + 33.4 = 34.5 kcal·mol^{-1}) is lower than that of pathway (c) (39.9 kcal·mol^{-1}), it is still too high to allow for a room-temperature reaction. Thus, we conclude that without further aid from neighboring porphyrin rhodium sites, **6** cannot react with ethylene at room temperature in the dark.

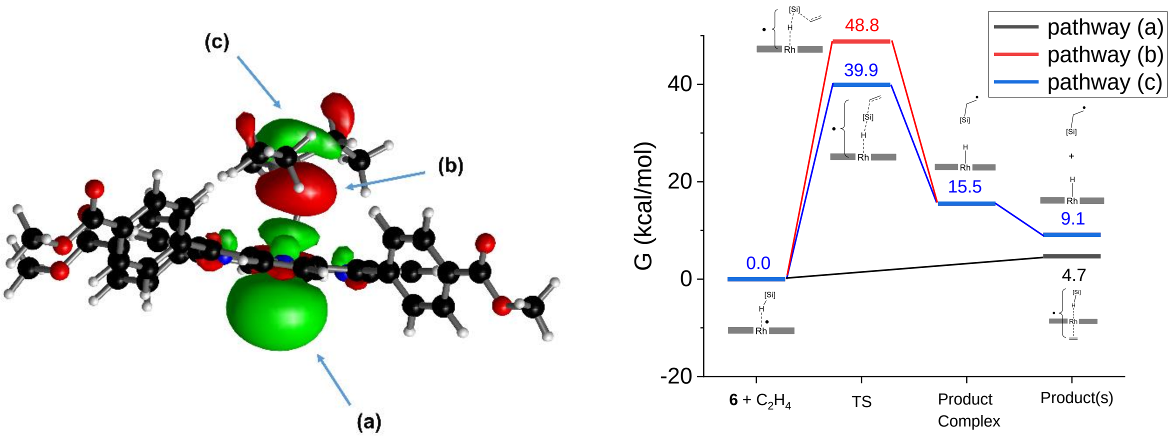

Figure S59. Left: possible ethylene attack pathways of **6**. Right: barriers and transition state structures of the three pathways.

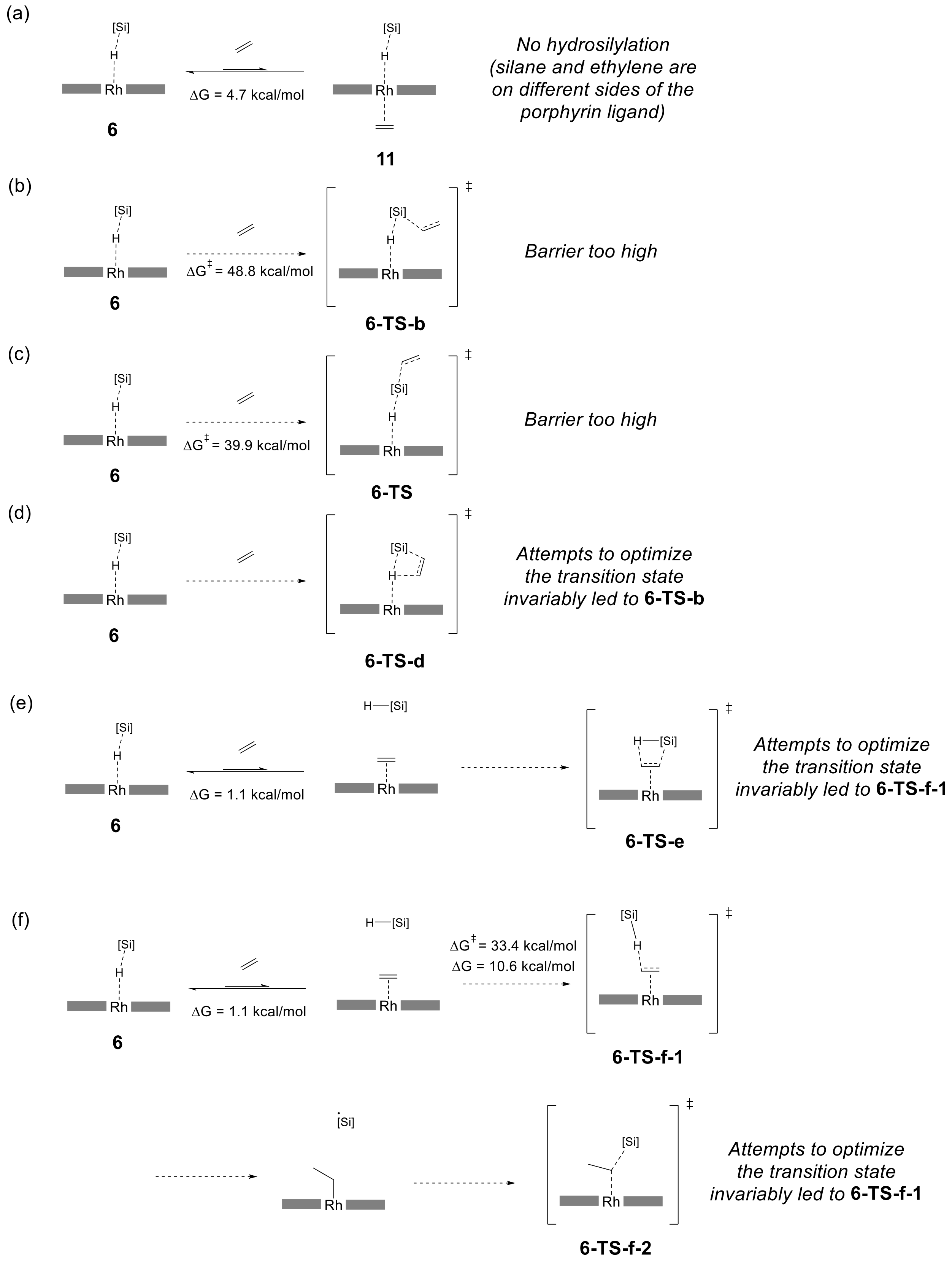
(a)
[Si]
H
Rh
6
ΔG = 4.7 kcal/mol
11
No hydrosilylation (silane and ethylene are on different sides of the porphyrin ligand)
(b)
ΔG‡ = 48.8 kcal/mol
6-TS-b
Barrier too high
(c)
ΔG‡ = 39.9 kcal/mol
6-TS
Barrier too high
(d)
6-TS-d
Attempts to optimize the transition state invariably led to 6-TS-b
(e)
H—[Si]
ΔG = 1.1 kcal/mol
6-TS-e
Attempts to optimize the transition state invariably led to 6-TS-f-1
(f)
ΔG‡ = 33.4 kcal/mol
ΔG = 10.6 kcal/mol
6-TS-f-1
6-TS-f-2
Attempts to optimize the transition state invariably led to 6-TS-f-1

Figure S60. Possible mechanisms of the hydrosilylation reaction catalyzed by a single porphyrin rhodium site.

18.1.2 NBO Analysis

The NBOs related to the Rh-H-Si 3c-3e interaction are shown in Figure S61. However, since the NBO program treats the Si-H→Rh interaction as an ionic interaction, it gives orbitals that are best described as σ(Si-H), σ*(Si-H) and n(Rh d_{z2}). In other words, it does not mix σ(Si-H) with n(Rh d_{z2}) as in the frontier orbitals.

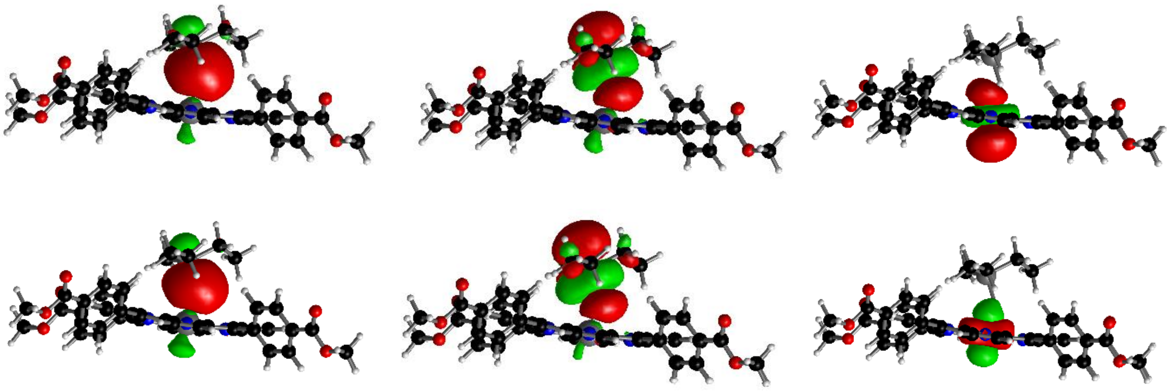

Figure S61. Alpha (top) and beta (bottom) NBOs of **6**. From left to right: σ(Si-H), σ*(Si-H) and n(Rh d_{z2}).

The alpha and beta NBOs are very similar to each other, except for the Rh d_{z2} orbital, where spin polarization effects make the alpha and beta orbitals differ noticeably. The energy expectation values of the σ(Si-H), σ*(Si-H) and n(Rh d_{z2}) NBOs are listed in Table S16:

Table S16. Orbital energy expectation values of the σ(Si-H), σ*(Si-H) and n(Rh d_{z2}) NBOs

Orbital	Alpha orbital energy/eV	Beta orbital energy/eV
σ(Si-H)	-12.83	-11.36
σ*(Si-H)	8.00	8.12
n(Rh d_{z2})	-9.96	-1.93

To estimate the mixings between these NBOs, we diagonalized the alpha and beta Fock matrices formed by these NBOs. The Fock matrices are given in Table S17:

Table S17. Alpha and beta Fock matrix elements between the σ(Si-H), σ*(Si-H) and n(Rh d_{z2}) NBOs

Alpha matrix elements/eV	σ(Si-H)	σ*(Si-H)	n(Rh d_{z2})

σ(Si-H)	-12.83	-0.09	-2.02
σ*(Si-H)	-0.09	8.00	-1.56
n(Rh d_{z2})	-2.02	-1.56	-9.96

Beta matrix elements/eV	σ(Si-H)	σ*(Si-H)	n(Rh d_{z2})
σ(Si-H)	-11.36	0.86	3.77
σ*(Si-H)	0.86	8.12	1.23
n(Rh d_{z2})	3.77	1.23	-1.93

The above 3x3 Fock matrices can be diagonalized to give the "quasi-canonical" molecular orbitals as well as their energy expectation values, shown in Table S18. In some sense, they are the frontier orbitals of the Rh-H-Si moiety that would be formed if the NBOs σ(Si-H), σ*(Si-H) and n(Rh d_{z2}) were not allowed to mix with other orbitals, but were allowed to mix within themselves.

Table S18. Energy expectation values and compositions of quasi-canonical orbitals formed by the σ(Si-H), σ*(Si-H) and n(Rh d_{z2}) NBOs

Orbital	Alpha orb. ene./eV	Alpha composition	Beta orb. ene./eV	Beta composition
σ(Rh-H-Si)	-13.92	0.88 σ(Si-H) + 0.04 σ*(Si-H) + 0.47 n(Rh d_{z2})	-12.70	-0.94 σ(Si-H) + 0.02 σ*(Si-H) + 0.33 n(Rh d_{z2})
n(Rh-H-Si)	-9.10	0.48 σ(Si-H) - 0.08 σ*(Si-H) - 0.88 n(Rh d_{z2})	-0.84	0.32 σ(Si-H) - 0.16 σ*(Si-H) + 0.93 n(Rh d_{z2})
σ*(Rh-H-Si)	8.13	-1.00 σ*(Si-H) + 0.09 n(Rh d_{z2})	8.36	0.07 σ(Si-H) + 0.99 σ*(Si-H) + 0.14 n(Rh d_{z2})

The energy expectation values of the alpha and beta n(Rh-H-Si) quasi-canonical orbitals agree qualitatively with the canonical frontier orbital energies (Table S15), and the remaining ~1 eV error can be attributed to contributions from other orbitals, such as the porphyrin π orbitals and the silyl σ(C-Si) and σ(C-H) orbitals. Moreover, the energy expectation values of the quasi-canonical orbitals σ(Rh-H-Si) and σ*(Rh-H-Si) are much lower and higher than the HOMO and LUMO, respectively, which explains why they are mixed so strongly with other orbitals such that we failed to find frontier orbitals dominated by these compositions. This highlights the benefit of using quasi-canonical orbitals compared to frontier orbitals in elucidating the electronic structure of **6**, in which we can forbid the delocalization of the Rh-H-Si orbitals into irrelevant orbitals (except for the minimal delocalization necessary for the orthogonality between

MOs), and obtain MOs that purely consist of a mixture of σ(Si-H), σ*(Si-H) and n(Rh d_{z2}), at the expense that the MOs are no longer exact eigenfunctions of the Fock operator.

The quasi-canonical orbitals can be plotted as shown in Figure S62:

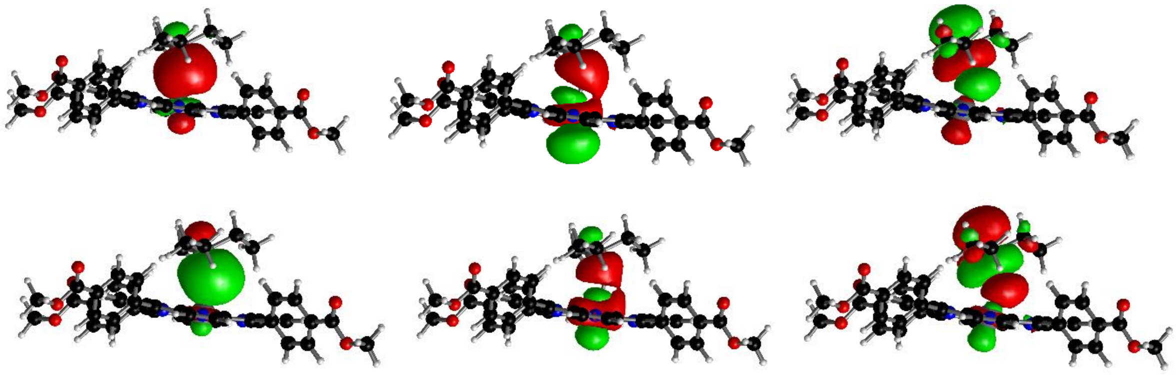

Figure S62. Alpha (top) and beta (bottom) quasi-canonical MOs of **6**. From left to right: σ(Rh-H-Si), n(Rh-H-Si) and σ*(Rh-H-Si).

Again, we confirm our conclusion that frontier molecular orbital theory alone would predict that the ethylene attacks **6** from either below (pathway (a) of Figure S59), right (pathway (b)), or above (pathway(c)).

Finally, second order perturbation theory analysis (E2PERT) shows that there are strong orbital interactions between the Rh center and H-$SiMeEt_2$. All orbital interactions between the Rh center and H-$SiMeEt_2$ that give > 1 kcal·mol^{-1} of contribution to the interaction energy are listed in Table S19**.**

Table S19. Selected E2PERT interaction energies between the silane ligand of **6** with the Rh center.

Orbital interaction (electron donor → electron acceptor)	Energy contribution (kcal·mol^{-1})
H-Si σ → Rh $4d_{z2}$	β electron: 18.24
H-Si σ → Rh 5s	α electron: 2.12
	β electron: 7.96
H-Si σ → Rh 5p	α electron: 2.47
	β electron: 1.19
Rh $4d_{z2}$ → H-Si σ*	α electron: 3.92

It is evident that the complex can be described as a σ complex of the silane on a Rh(II) center. The main Rh atomic orbital responsible for accepting the coordination is the Rh $4d_{z2}$ orbital, but the 5s and (to a lesser extent) the 5p orbitals also have important contributions. The α electron on the Rh $4d_{z2}$ orbital

furthermore back-donates into the H–Si σ* orbital of the silane, which is a rare mode of back-bonding considering that the acceptor orbitals of back-bonding interactions are typically of π* character. Nevertheless, σ back-bonding only accounts for a small fraction of the total interaction energy, and σ(H-Si)→Rh donation dominates the interaction.

18.2 Attempt to Optimize Compound 12

To see whether **6** and ethylene can form adducts other than **13**, we performed intrinsic reaction coordinate (IRC) analysis of **6-TS**, and optimized the reactant structure that is connected directly with the transition state via the IRC. This gives a reactant complex (**12**) with a Si…C(ethylene) distance of 3.941 Å (Figure S63), which is more than the sum of their van der Waals radii (2.10 + 1.70 = 3.80 Å). Meanwhile, its Gibbs free energy is 6.5 kcal·mol^{-1} above the separated reactants **6** + ethylene. There is thus no specific Si…C(ethylene) interaction present to stabilize **12**, and **12** should be best described as a fleeting intermediate. As we located **12** by following the IRC, we conclude that there is no other reactant complex along the reaction pathway with a shorter Si…C distance, and therefore there is no stable non-covalent complex of **6** and ethylene where the ethylene interacts with **6** through the Si atom.

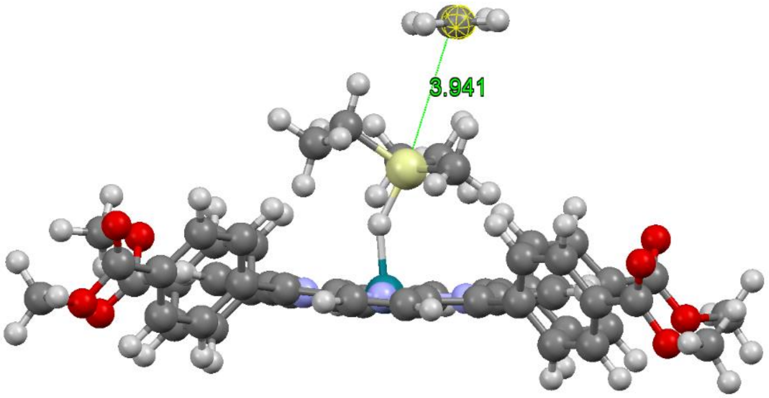

Figure S63. Optimized structure of **12**, with the Si…C distance shown (Å)

18.3 Electron Transfer of 6 to External Electron Acceptors

As illustrated in Figure S59, the direct ethylene attack of **6** only results in coordination of ethylene to the Rh center (i.e. formation of **13**), and does not lead to a hydrosilylation pathway with a reasonable barrier. To achieve hydrosilylation at room temperature, further activation of the Si–H bond would be necessary.

Table S18 shows that the n(Rh-H-Si) orbital has larger contribution from σ(Si–H) than from σ*(Si–H), i.e. electrons on the n(Rh-H-Si) orbital contribute positively to the Si-H bond order. Removing an electron from **6** should result in a depletion of the n(Rh-H-Si) occupation (thanks to the symmetry allowed mixing of HOMO and n(Rh-H-Si)), and thus a weakened Si–H bond. This is indeed corroborated by NBO Wiberg bond order analyses of **6** and its monocation **6**$^{+}$ (Table S20): while in **6** the Si–H bond has a much higher bond order than the Rh–H bond, their difference is greatly reduced after ionizing one electron from **6**, and the sign of the difference is eventually reversed after the geometry is allowed to relax. The equilibrium structure of **6**$^{+}$ (Figure S64) is characterized by a much more pronounced Rh-H-Si bending (99°) than in **6**

(146°), and significant Si-Rh bonding as suggested by both the Si…Rh bond length and Si-Rh Wiberg bond order. Even considering the enhanced Si…Rh bonding in **6⁺** compared to **6**, **6⁺** still features a smaller total Si-H + Si-Rh bond order (0.547) than **6** (0.775), suggesting that the silyl group of **6⁺** can be more easily transferred to the incoming substrates than the one in **6**.

Table S20. NBO Wiberg bond orders of **6** and its monocation **6⁺**.

	Si-H	Rh-H	Si-Rh
**6** [a]	0.691	0.197	0.084
**6⁺** [a]	0.482	0.355	0.091
**6⁺** [b]	0.273	0.464	0.274

[a] Evaluated at the equilibrium geometry of **6**.

[b] Evaluated at the equilibrium geometry of **6⁺**.

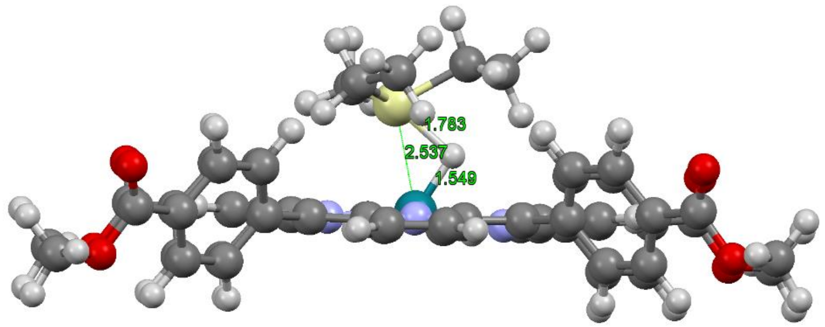

Figure S64. Structure of **6** in its cationic state. Key bond distances given in Angstrom.

What remains to be investigated is the thermodynamic feasibility of removing one electron from **6** under the reaction conditions. We thus calculated the thermodynamics of the electron transfer from **6** to a few potential oxidants present in the system, assuming that the oxidants are infinitely far from **6** (Table S21). One can easily see that Rh(II)-**3** is thermodynamically the strongest oxidant (entry 1), and is even stronger than the superoxo species Rh-O-O· (**9**) and hydroperoxo species Rh-O-OH (**10**) (entries 2-3). However, even with Rh(II)-**3** as the oxidant, the electron transfer is still 43.0 kcal·mol^{-1} uphill.

Nevertheless, the electron transfer can be made thermodynamically more favorable, if extra ligands are coordinated to **6** to make it more electron rich and reduce its redox potential. Of the two substrates (C_2H_4, $HSiMeEt_2$) and the solvent (C_6H_6), C_2H_4 coordination (which yields **13**) is the most effective in reducing the endergonicity of the electron transfer (entry 6), and reduces the Gibbs free energy demand by 14.1 kcal·mol^{-1}. Even more, when the C_2H_4 molecule instead approaches the Si atom and forms a Rh…H…Si…C_2H_4 transition state (Figure S59, pathway (c)), the electron transfer can be made more favorable by 31.4 kcal·mol^{-1} (entry 9). While we cannot calculate the electron transfer Gibbs free energy of **13-TS** to Rh(II)-**3**, due to the absence of the corresponding cationic transition state **13⁺-TS**, a simple estimate based on an additive model suggests that electron transfer from **13-TS** to Rh(II)-**3** would be slightly downhill. Therefore, Rh(II) sites near **13**, which are always available in the experimental MOF

system, will lead to a decrease of the reaction barrier by temporarily oxidizing **13-TS** when the system approaches the TS, and then giving the electron back to **13-TS** after the system traverses the TS (*vide infra*).

1-pentene (which is used in the kinetic experiments in place of ethylene) is almost as potent as ethylene for promoting the ET of **6** to Rh(II)-**3** (entry 11), via forming the adduct **6**-pentene.

Table S21. Electron transfer Gibbs free energy changes between a few pairs of electron donors and electron acceptors. The electron donor and acceptor are assumed to be infinitely far from each other both before and after the electron transfer.

Entry	Electron donor	Electron acceptor	ΔG ($kcal \cdot mol^{-1}$)
1	**6**	Rh(II)-**3**	+43.0
2	**6**	**9**	+62.2
3	**6**	**10**	+59.5
4	**6**	O_2	+78.0
5	**6**	Zr_6O_8 cluster [a]	+79.3
6	**13**	Rh(II)-**3**	+28.9
7	**6**-C_6H_6	Rh(II)-**3**	+40.7
8	**6**-silane	Rh(II)-**3**	+33.3
9	TS of **6** + C_2H_4 (**6-TS**)	Rh(II)-**3**	+11.6 [b]
10	TS of **13** + C_2H_4 (**13-TS**)	Rh(II)-**3**	-2.5 [c]
11	TS of **6**-pentene	Rh(II)-**3**	+29.3

[a] The Zr_6O_8 node of the MOF, approximated as a $Zr_6O_4(\mu_3\text{-}OH)_4(\mu_1\text{-}OH)_6(OH_2)_6(OOCPh)_6$ cluster.

[b] The single electron oxidation of **6-TS** can yield two possible transition states, which are very close in energy (*vide infra*); the one with the lower Gibbs free energy is used here in calculating ΔG.

[c] The Gibbs free energy after the electron transfer cannot be calculated, since the reaction of $\mathbf{13}^+$ and C_2H_4 is a purely uphill reaction and therefore does not have a TS. Instead, the figure is estimated from (entry 6) + (entry 9) – (entry 1), i.e. by assuming that the effects of the two C_2H_4 molecules in **13-TS** are additive.

18.4 Role of Electron Transfer and Site Proximity in Reducing the Reaction Barrier

The 2.5 $kcal \cdot mol^{-1}$ barrier reduction computed in the previous subsection is still rather modest compared to the 41.3 $kcal \cdot mol^{-1}$ barrier (relative to **13**) of the **13** + C_2H_4 reaction (**Figure S65**), and does not suffice to make the reaction occur near room temperature. However, up to now we have not accounted for the interaction of **13** and the electron acceptor Rh(II)-**3**, particularly after the electron transfer. Both reactants are electric neutral, non-polar complexes before the electron transfer reaction, and they are separated by a long distance (13.6 Å as measured by the experimental single crystal structure; 13.7 Å as given by DFT calculations of **13** and a neighboring Rh(II)-**3** site). Thus, we expect that their interactions are minimal before the electron transfer. After the electron transfer, however, **13** (in the form of **13-TS**) acquires a

positive charge, and Rh(II)-**3** acquires a negative charge. Their electrostatic interaction through the dielectric medium (with dielectric constant ε) yields an energy stabilization of:

$$E = \frac{1}{\epsilon R}$$

Where R is the distance between the two porphyrin rhodium sites. Plugging in the dielectric constant of benzene, ε=2.27, and the Rh…Rh distance of the MOF, R=13.6 Å, yields an estimate of the stabilizing energy as 10.7 kcal·mol^{-1}.

To verify whether the extra stabilization indeed contributes to a reduction of the barrier, we calculated the Gibbs free energy profiles of the reaction **13** (or **6**) with C_2H_4 under a variety of assumptions (**Figure S65**). As mentioned before, the formation of **13** from **6** is uphill by 4.7 kcal·mol^{-1}. Direct reaction of **6** with C_2H_4 to yield the product complex **6-P** has an unfavorable barrier (39–40 kcal·mol^{-1}) regardless of whether a neighboring Rh(II)-**3** is taken into account into the calculation or not, since the electron transfer of **13-TS** to Rh(II)-**3** is thermodynamically unfavorable even after subtracting the Coulomb stabilization energy 10.7 kcal·mol^{-1} (11.6 – 10.7 = + 0.9 kcal·mol^{-1}). Reaction of **13** with C_2H_4 likewise has a very high barrier (**13-TS**, 46.0 kcal·mol^{-1} relative to **6**) when not aided by another Rh(II) center, with the difference between **6-TS** mainly attributed to the free energy increase upon going from **6** to **13**. However, with the presence of a neighboring Rh(II) site, **13** reacts with C_2H_4 with a much lower barrier (**13-TS-MOF**, 29.7 kcal·mol^{-1}), which is lower than **6-TS** even given the unfavorable coordination of C_2H_4 to **6**.

The reaction of **13**$^+$ with C_2H_4 is purely uphill, and therefore does not have a TS or product complex; thus, it is not shown on **Figure S65**. Despite this, we can see the effect of single electron oxidation on reducing the hydrosilylation reaction barrier by studying the **6**$^+$ + C_2H_4 reaction. The reaction is characterized by a Rh…H…Si…C_2H_4 structure that is a local minimum on the potential energy surface (**6**$^+$**-IM**), which are connected to the reactant and product complex, respectively, by almost barrierless reaction paths. The overall reaction free energy barrier is only 11.9 kcal·mol^{-1}, much lower than that of **6-TS** (39.9 kcal·mol^{-1}), confirming our hypothesis that single electron oxidation of **6** significantly activates the Si-H bond towards attack by C_2H_4. However, **6**$^+$ is too energy costly to generate using Rh(II)-**3** as the oxidant, such that while **6**$^+$**-TS1** and **6**$^+$**-TS2** are not much higher than **6**$^+$ in terms of Gibbs free energy, they are still higher than **6-TS** or even **13-TS**, making the **6**$^+$ pathway inaccessible.

Alternatively, the silane $HSiMeEt_2$ can also act as a ligand that promotes the reaction, in place of the ethylene ligand in **13**. Coordination of $HSiMeEt_2$ to **6** forming **6**-silane is uphill by 2.3 kcal/mol (again when considering that a benzene molecule has to be displaced from **6**), which is 2.4 kcal/mol less unfavorable than the coordination of ethylene to **6**. Meanwhile, although the ET from **6**-silane to Rh(II)-**3** is 4.4 kcal/mol more unfavorable than the ET from **13** to Rh(II)-**3** (**Table S21**), the barrier of the reaction of **6**-silane with C_2H_4 is only 0.9 kcal/mol higher than that of **13** with C_2H_4. Therefore, relative to **6**, silane coordination gives a 2.4 – 0.9 = 1.5 kcal/mol lower reaction barrier than ethylene coordination. For 1-pentene we found similar results (not shown on **Figure S65** for clarity): the coordination of 1-pentene to **6** is uphill by 4.0 kcal/mol, which is 1.7 kcal/mol higher than the coordination of silane, while the reaction of **6**-silane with 1-pentene has a 0.5 kcal/mol higher barrier than that of **6**-pentene with 1-pentene. Therefore, with 1-pentene as the substrate, our calculation still predicts that silane coordination is the dominant mechanism, although again the difference is small and on the same order as computational error. This is consistent with the experimental kinetic order of 0.7 (predicted 1) with respect to 1-pentene, and 1.3 (predicted 1, as although two molecules of $HSiMeEt_2$ are involved, the coordination of the first $HSiMeEt_2$ to Rh(II)-**3** is

downhill by 3.1 kcal/mol and does not contribute to the reaction order) with respect to $HSiMeEt_2$, for the hydrosilylation of 1-pentene with $HSiMeEt_2$.

Since (1) we initially performed all calculations with ethylene as a *trans*-ligand, (2) redoing all calculations with silane as the *trans*-ligand is very costly, and (3) the qualitative conclusions (except for kinetic order with respect to the substrates) obtained using silane as a *trans*-ligand should be identical to those using ethylene as a *trans*-ligand, in many of the subsequent calculations we will still use ethylene instead of silane as the *trans*-ligand of **6**.

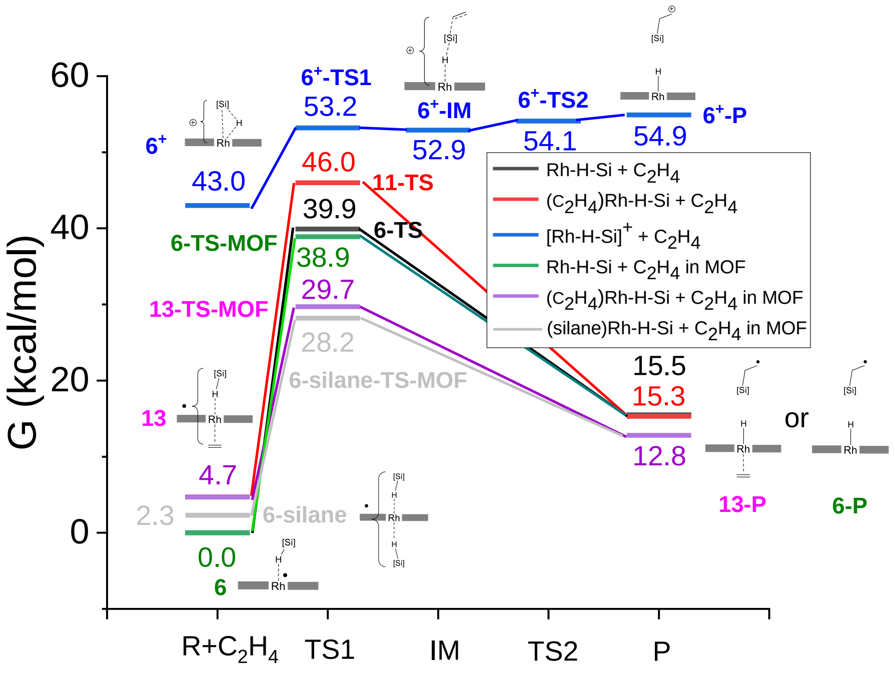

Figure S65. Reaction profiles of the Rh-H-Si complex **6**, its cation, and its C_2H_4 adduct with C_2H_4. Lines labeled "in MOF" denote reactions where another porphyrin Rh(II) site is included and available for accepting one electron from the Rh-H-Si-ethylene reaction center. Benzene (ε = 2.27) is used as the solvent in all the calculations.

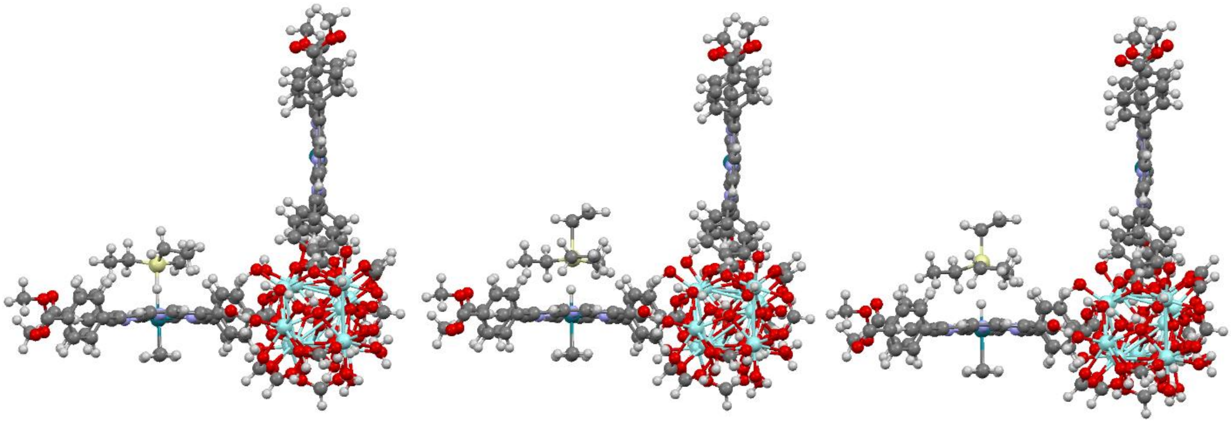

Figure S66. From left to right: structural models of **13**, **13-TS-MOF** and **13-P**, where the neighboring porphyrin Rh(II) site and the two Zr_6O_8 nodes bridging them are taken into account.

Table S22. Key bond distances along the reaction path of **13** and C_2H_4, with the presence of a neighboring porphyrin Rh(II) site.

	**13**	C_2H_4	**13-TS-MOF**	**11-P**
Rh-(C_2H_4) [a]	2.274, 2.282	N.A.	2.474, 2.477	2.577, 2.578
Rh-H	1.790	N.A.	1.589	1.527
H-Si	1.555	N.A.	1.932	2.483
Si-CH_2CH_2·	N.A.	N.A.	2.319	1.981
(Si)CH_2-CH_2·	N.A.	1.343 [b]	1.379	1.440
SiCH_2CH_2· … Rh(II)-**3** [c]	N.A.	N.A.	9.289	9.155

[a] The Rh-C bond lengths between the Rh atom and its coordinated ethylene ligand.

[b] The CH_2=CH_2 bond length.

[c] The distance between the radical carbon of the reacting C_2H_4 molecule, and the Rh center of Rh(II)-**3**.

To better understand the electronic structure change along the reaction pathway, we have plotted the energies and Hirshfeld spin populations along the IRC (Figure S67). At each IRC step, we could locate two wavefunctions at the ωB97M-V level: one of them is a broken symmetry singlet, with both Rh sites in their neutral states, and the other is a closed shell singlet, with the **13-TS** part of the system carrying a positive charge and the Rh(II)-**3** part carrying a negative charge, making the total system zwitterionic. The energy of the neutral wavefunction has a well-defined maximum (at the TS), and then drops quickly after the TS as the Si-H bond is broken and the spin density on Rh is transferred to the ethylene carbon atom highlighted by the cyan circle. By comparison, the energy of the zwitterionic wavefunction increases almost monotonically throughout the reaction, paralleling our previous observation that the reaction of $\mathbf{13}^+$ with C_2H_4 is purely uphill. The potential energy curve of the zwitterionic state intersects with that of

the neutral state at two points, one at step 8 and the other just after the TS at step 15. Therefore, the energetically optimal reaction pathway involves the following steps:

(1) C_2H_4 approaches the neutral **13** complex from the direction of the Si atom;
(2) When C_2H_4 is sufficiently close to **13**, electron transfer to a neighboring Rh(II) site becomes thermodynamically feasible, and electron transfer takes place;
(3) The Si…C_2H_4 distance continues to decrease. Although the energy continues to rise, the energy increase is much slower than if the electron transfer from **13** to Rh(II)-**3** did not take place;
(4) At one point, when the Si-H bond is sufficiently elongated, it becomes again thermodynamically favorable to transfer one electron back from Rh(II)-**3** to **13**, after which the energy starts to decrease until the product complex is formed. Thus, instead of forming the cation $Et_2MeSi-CH_2CH_2^+$, the reaction yields the radical $Et_2MeSi-CH_2CH_2\cdot$, i.e. the same product as if the electron transfer did not occur (Figure S67).

To summarize, the reaction between **13** and C_2H_4 is sped up by two electron transfers along the pathway, such that **13** is temporarily oxidized before the TS and immediately reduced back to its neutral form after the TS, to circumvent the high barrier on the neutral potential energy surface.

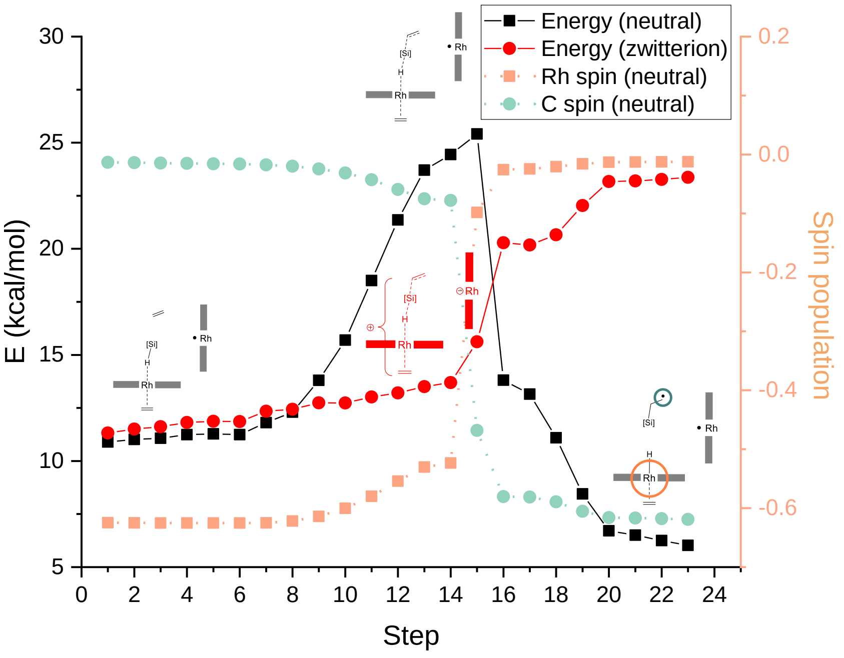

Figure S67. ωB97M-V/SARC-ZORA-TZVP(Rh)/ZORA-def2-TZVP(C,H,O,N,Si) energy and Hirshfeld spin populations along the IRC of the **13** + C_2H_4 reaction, with the presence of another nearby Rh(II) site. The atoms for which spin populations of the neutral wavefunction are plotted are highlighted by circles (the spin populations of the zwitterionic wavefunction are everywhere zero). The energy zero point is chosen as the total energy of the separated reactants.

18.5 Extra Electrostatic Stabilization of **13-TS-MOF** by the MOF Linkers and Nodes

Despite the large size of our structural models, which are composed of two porphyrin rhodium units (346 atoms for **13-TS-MOF**), they are still expected to underestimate the tendency of electron transfer from **13-TS** to Rh(II)-**3**, due to the neglect of other MOF units (porphyrin linkers and Zr_6O_8 nodes). Charges formed within a MOF are stabilized by not only the dielectric response of the solvent (benzene in the present case), but also the dielectric response of the MOF itself. Since the MOF used in the present study (PCN-224) possesses highly polarizable linkers (porphyrin), it should be more polarizable by electric charges than benzene molecules placed at a similar distance, and therefore yields a larger energy stabilization of electric charges than if the charge were situated in pure benzene.

To estimate the dielectric constant of the MOF, ε (including the benzene solvent that fills its pores), we employed the Clausius-Mossotti relation:

$$\frac{\epsilon - 1}{\epsilon + 2} = \frac{4\pi\alpha}{3V}$$

Where V is the volume per unit cell, and α is the polarizability per unit cell. The volume V is easily calculated from the experimental unit cell parameters (a = b = c = 38.512(2) Å, α = β = γ = 90°) as 3.85×10^5 au. Part of V (and α) is contributed by the MOF scaffold, while the rest is contributed by the benzene solvent. As periodic calculations of the MOF would be too costly, we estimated V from:

$$V = 12V(\mathbf{18}) + 8V\left(\mathrm{Zr_6O_4}\left(\mu_3 - \mathrm{OH}\right)_4\left(\mu_1 - \mathrm{OH}\right)_6(\mathrm{OH_2})_6(\mathrm{OOCH})_6\right) - 48V(\mathrm{HCOOMe})$$

And similar for α. In essence, we used methyl and formate groups to saturate the dangling bonds of the porphyrin linker and the Zr_6O_8 cluster, respectively, compute their respective molecular volumes and polarizabilities, and finally subtract the correct number of methyl formate molecules to remove the contribution of the capping groups. For simplicity, it was assumed that all the Rh sites in the MOF are Rh(II) radicals. The GEPOL solvent-excluded volumes were used for the calculations, as they represent the volume of solvent that the MOF displaces when the MOF is immersed into the solvent. The total polarizability of the benzene solvent within the MOF's pores was estimated from the experimental dielectric constant of benzene (2.27) and the free volume of the MOF per unit cell (by subtracting the total GEPOL volume of the MOF from the unit cell volume), by applying the Clausius-Mossotti relation in the reverse direction.

The polarizabilities and volumes of the different components of the MOF are listed in Table S23. It can be seen that the MOF linkers and nodes have a higher polarizability per volume than benzene, such that while the linkers and nodes account for less than 1/4 of the total MOF volume, they provide almost as much polarizability as the benzene that fills its pores. Therefore, while the "empty" MOF (with vacuum instead of benzene in its pores) was predicted to have a dielectric constant of only 1.62, the benzene-filled MOF has a predicted dielectric constant of 3.00, significantly higher than pure benzene (2.27).

Table S23. Computationally estimated polarizability and volume contributions of Rh-PCN-224

	α (au)	V (au)
$Zr_6O_4(\mu_3\text{-}OH)_4(\mu_1\text{-}OH)_6(OH_2)_6(OOCH)_6$	494	5682

**18**	1137	6389
HCOOMe	38	660
MOF total (without benzene)	15774	90425
Benzene	20996	295040

While a higher dielectric constant weakens the Coulomb attraction between the positive and negative charges produced by the electron transfer from **13-TS** to Rh(II)-**3**, it also stabilizes the charges themselves better than a lower dielectric constant environment does, and the latter effect was found to dominate over the former effect. Thus, the 29.7 kcal·mol^{-1} free energy barrier calculated for the ethylene coordination pathway with ε = 2.27 (Fig. 9C) was reduced to 24.9 kcal·mol^{-1} after re-calculating the single point energies with ε = 3.00, i.e. a 4.8 kcal/mol reduction. The 28.2 kcal·mol^{-1} barrier of the silane coordination pathway (**Figure S65**) is thus expected to reduce to 23.4 kcal·mol^{-1}. It must be noted, however, that the current estimate of the dielectric constant of the MOF is rather crude, and especially we did not take into account the inhomogeneity of the MOF. The main message here is that the reaction barrier is reduced drastically with a modest increase of the dielectric constant, and that the dielectric constant of the benzene-filled MOF is higher than pure benzene. The numerical value of the barrier reduction may however be associated with an error of a couple of kcal·mol^{-1}.

18.6 Si–H Bond Photolysis of 6

We have shown in the main text that the photon energy of the experimentally used light source (73.3 kcal·mol^{-1}) is sufficient for overcoming not only the total ΔG (28.7 kcal·mol^{-1}) but also the free energy barrier ΔG‡ (39.1 kcal·mol^{-1}) of the H–Si bond cleavage. In this subsection, we further provide preliminary computational results on why H–Si bond photolysis is observed despite the fact that it is not the weakest bond in the complex.

Thermodynamically, **6** decomposes primarily by dissociating its silane ligand (ΔG = 3.1 kcal·mol^{-1}); by comparison, dissociating the silyl radical to yield the Rh-H complex **5** is significantly more difficult (ΔG = 28.7 kcal·mol^{-1} relative to **6**; or 25.5 kcal·mol^{-1} relative to Rh(II)-**3** + $HSiMeEt_2$). The higher Si–H bond strength compared to Rh–H is also reflected by the NBO Wiberg bond orders (Table S20). However, the bond strength difference may be partially evened out by light excitation, making the Rh–H bond stronger and the Si-H bond weaker. To test this hypothesis, we calculated the Rh–H and H–Si bond orders of all excited states of **6** with excitation energy within 4.5 eV (Figure S68). Most excited states have similar bond orders as the ground state, and a few excited states have notably stronger or weaker H–Si bonds than the ground state. Whenever a state has a stronger H-Si bond than the ground state, its Rh–H bond is usually weaker than the ground state, and vice versa; this shows that the Rh–H and H-Si bond orders are mostly determined by the occupation number of the n(Rh-H-Si) orbital, with higher n(Rh-H-Si) occupation numbers weakening the Rh–H bond and strengthening the H–Si bond, and lower n(Rh-H-Si) occupation numbers having the opposite effect.

Interestingly, bright states (states with oscillator strength > 0.01) tend to have weaker H-Si bonds and stronger Rh-H bonds than the ground state, as exemplified by the D_{13} and D_{21} states at 424 and 352 nm,

respectively, highlighted in Figure S68 by arrows. In the case of the D_{13} state, the H-Si bond order reaches its lowest value among all excited states studied (0.56), and the Rh-H bond order accordingly reaches its highest value (0.29). Both the 352 and 424 nm peaks are close to the experimental excitation wavelength (390 nm), so they are likely to be the main excitation pathways of **6** under the experimental conditions. Therefore, our calculations suggest that light excitation of **6** at 390 nm should increase the likelihood of Si-H bond breaking compared to the ground state, consistent with the experimental observation of the $\cdot SiMeEt_2$ radical when photolyzing **6**.

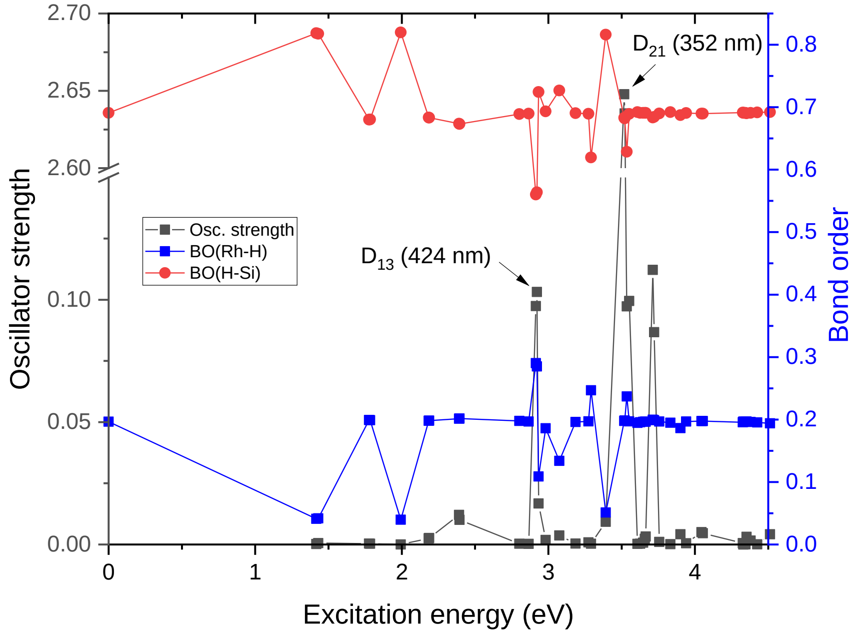

Figure S68. Oscillator strengths, as well as the Rh-H and H-Si NBO Wiberg bond orders of the excited states of **6**, plotted as a function of excitation energy. The bond orders of the excited states were calculated from the unrelaxed TDDFT excited state densities. The bond orders in the ground state are shown at zero excitation energy for comparison. The absorption wavelengths of the strongest absorptions are shown.

To explain why bright states of **6** tend to lead to weaker Si–H bonds and stronger Rh–H bonds, we recall that only one of the four frontier orbitals of the porphyrin ligand (the a_{2u} orbital) has the same symmetry with the n(Rh-H-Si) orbital, and the a_{2u} orbital is an occupied orbital. Meanwhile, only excited states with significant porphyrin → porphyrin local excitation character can have large oscillator strengths, because only the porphyrin part of **6** possesses a large conjugated system. Therefore, any low-lying bright state of the system must contain substantial contributions from the $a_{1u} \rightarrow e_g$ and $a_{2u} \rightarrow e_g$ excitations (which is a well-known prediction of the Gouterman four-orbital model[116]). As only the a_{2u} (but not the e_g) orbital is symmetrically allowed to mix with n(Rh-H-Si), such excitations can only deplete the n(Rh-H-Si) occupation number, but not enrich it. Difference density plots of the two bright states, D_{13} and D_{21}, indeed confirm that electron density is transferred from the n(Rh-H-Si) orbital to the porphyrin ligand orbitals during excitations from the ground state to these states (Figure S69). As repeatedly mentioned above, depleting the n(Rh-H-Si) electrons would result in weakening of the H–Si bond and strengthening of the Rh-H bond, which nicely explains our observations. Finally, we note that a similar orbital symmetry argument has

been successfully applied to explain the selective homolytic photolysis of a porphyrin Rh-alkyl bond in the presence of a weaker Rh–CO bond.[95]

We must however point out two limitations of the present analysis, namely that (1) it is possible that the H-Si bond weakening is not sufficient for causing its cleavage, and (2) the H–Si bond cleavage may occur from an excited state different from the light absorbing state. Accounting for these two effects will necessitate a non-adiabatic molecular dynamics simulation, which is beyond the scope of the present paper. Also, our experimental evidence only shows that H–Si bond cleavage exists under light irradiation, but not that H–Si bond cleavage dominates over Rh–H bond cleavage. Even if some photons result in the Rh–H bond cleavage of **6**, the dissociated fragments (Rh(II)-**3** and $EtMe_2SiH$) can rapidly recombine to yield **6**, so that the Rh–H bond photolysis of **6** cannot be observed with the currently used techniques.

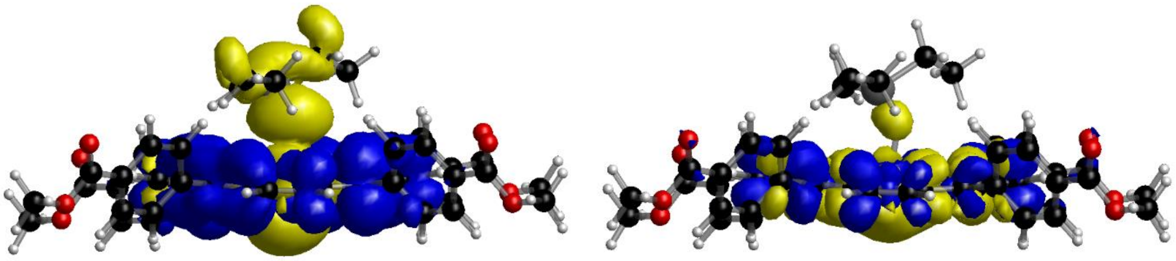

Figure S69. Difference density plots of the D_{13} (left) and D_{21} (right) states of **6** (isovalue: 0.0002). Yellow: electron density depletion upon excitation. Blue: electron density accumulation upon excitation.

18.7 Entropic Barrier of the H–Si Bond Dissociation of **6**

As mentioned in the main text, the H-Si bond of the Rh-H-Si complex **6** has a bond dissociation free energy (BDFE) of ΔG = 28.6 kcal·mol^{-1}, and the dissociation process is energetically purely uphill at the BP86-D3 level of theory. However, this does not imply that the Gibbs free energy barrier for the bond dissociation ($\Delta G^{\ddagger}$) is 28.6 kcal·mol^{-1}, because the free energy surface may still have a barrier. During the H–Si bond dissociation, the silyl radical gradually acquires more and more degrees of freedom, making a negative contribution to the free energy of the system by increasing the system entropy. If the entropy only starts to significantly increase after the energy reaches its plateau, then the free energy of the system may have a maximum along the dissociation curve even if the energy does not have a maximum.

Since a rigorous treatment would necessitate costly ab-initio molecular dynamics (AIMD) simulations to accurately capture the entropy contributions, a simpler approach proposed by Baik et *al.* was used here.[117] We first performed a relaxed scan of the H–Si bond at the BP86-D3/SARC-ZORA-TZVP(Rh)/ZORA-def2-TZVP(Si)/ZORA-def2-SVP(C,H,O,N)/CPCM(benzene) level of theory from 2.0 Å to 6.0 Å, with a step length of 0.2 Å. Then, we performed harmonic vibrational analyses on each of the structures, at the same level of theory. The resulting Gibbs free energy corrections were then added onto high-level single point energies (calculated at the ωB97M-V/SARC-ZORA-TZVP(Rh)/ZORA-def2-TZVP(C,H,O,N,Si) level) to give the Gibbs free energies along the relaxed scan path. The ωB97M-V method was chosen instead of DLPNO-CCSD(T) (which we used for all other single point energy calculations of single-porphyrin systems) due to computational cost considerations. Subtracting the Gibbs free energies of the separated products **5** + **8** (computed at the same level of theory) from the highest Gibbs free energy of all the structures along the reaction path, gives the estimated difference of $\Delta G^{\ddagger}$ and ΔG. Adding this onto the ΔG calculated at the

DLPNO-CCSD(T)//BP86-D3 level of theory yields our final estimate of ΔG‡. Note that although this method involves calculating harmonic Gibbs free energy corrections at non-stationary structures, which is in principle not rigorous, it has been argued by Baik et al.[117] that the present method is at least much more accurate than the simple approximation ΔG‡ ≈ ΔG.

The Gibbs free energy (as well as its components) as a function of the H–Si bond length is shown in Figure S70. The ωB97M-V//BP86-D3 energy has a shallow minimum at a H–Si bond length of 4.2 Å, but continues to increase when further stretching the H-Si bond. The entropy contribution to the Gibbs free energy (-TS) has a few local maxima along the reaction path, with the most pronounced one being the one at 5.6 Å. The thermal correction of the energy (U(T)-U(0)) and the zero point energy (ZPE) are almost constant throughout the bond length range. Summing the terms together, one obtains a maximum of the Gibbs free energy at 5.6 Å, where the Gibbs free energy is 10.5 kcal·mol^{-1} above the summed Gibbs free energy of the products **5** and **8**. Adding this onto the ΔG of the reaction (28.6 kcal·mol^{-1}) yields the final estimate of the free energy barrier, 39.1 kcal·mol^{-1}.

We note that a similar but smaller difference between ΔG‡ and ΔG (6.58 ± 0.01 kcal·mol^{-1}) has been experimentally measured for the dissociation of a bulky four-coordinate stannyl radical from a cobalt(II) porphyrin complex.[118] For **6**, the somewhat larger difference between ΔG‡ and ΔG (10.5 kcal·mol^{-1}) can be rationalized by the smaller size of the silyl radical **8**, whose van der Waals attraction with **5** is relatively weak. The weak van der Waals attraction between **5** and **8** leads to a less negative interaction energy E, and thus a more positive ΔG‡.

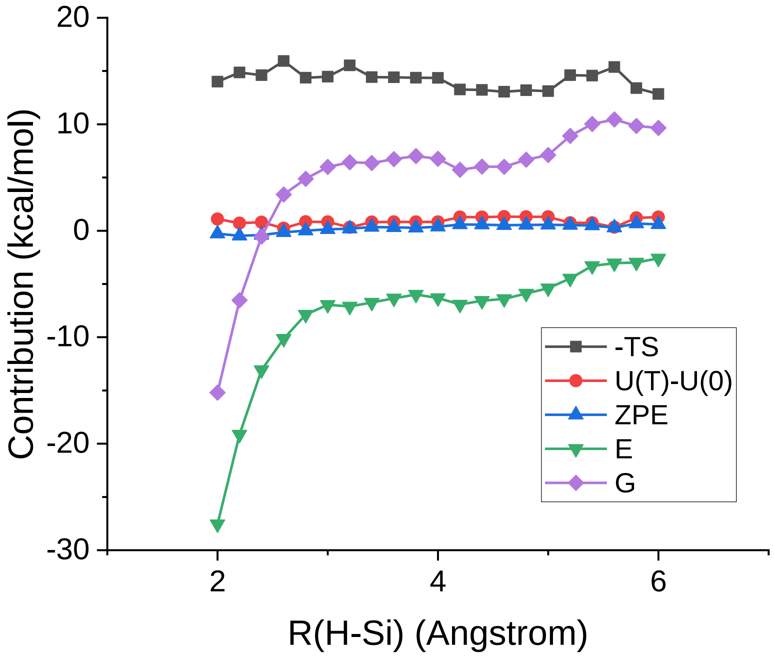

Figure S70. Relative Gibbs free energy of **6** with respect to 5 + 8, as well as its components (entropy, thermal correction of the energy, zero-point energy, single point energy), as a function of the H–Si distance.

18.8 Reaction of $Et_2MeSi\text{-}CH_2CH_2$ Radical (7) with Rh(III)-H (5)

Once the silyl radical **8** is formed, it can add barrierlessly and in an exergonic fashion to C_2H_4 to yield the $Et_2MeSi\text{-}CH_2CH_2\cdot$ radical **7**. **7** can then abstract the Rh-H hydrogen atom of **5** to regenerate Rh(II)-**3** and give the final product $Et_2MeSi\text{-}CH_2CH_3$. The reaction undergoes barrierlessly if the radical carbon of **7** points towards the Rh-H bond when **7** approaches **5**, but alternative side reactions can happen as well if the initial orientation of **7** is not optimal (*vide infra*).

The $Et_2MeSi\text{-}CH_2CH_2\cdot$ radical (**7**) formed after the reaction of **13** with C_2H_4 has its radical carbon pointing away from the Rh-H bond of **5** formed behind (Figure S66). To abstract the Rh-H hydrogen of **5**, **7** has to either turn around to point its radical carbon towards **5**, or leave the solvent cage and meet another Rh-H site in the MOF. To investigate the relative ratio of the two reaction pathways as well as other possible side reactions, we performed molecular dynamics (MD) simulations of the product complex **13-P**.

To reduce computational cost, we only included one porphyrin rhodium unit in our MD simulations, and capped the four carboxyl groups of the porphyrin ligand by methyl groups. Even then, we could only afford to use semiempirical methods for the MD simulations. As no semiempirical method appears to be accurate enough for the Rh-H and Si-C bonds, we employed the "H_0-tuning" approach by Mewes et al.[119] to improve the accuracy of the GFN2-xTB method specifically for the porphyrin Rh-H and $Si\text{-}CH_2CH_2\cdot$ bonds, and used the resulting re-tuned method for the MD simulations. Specifically:

(1) We performed relaxed scans of the Rh-H bond of **5**, as well as the $Si\text{-}CH_2CH_2\cdot$ bond of **7**, at the (unchanged) GFN2-xTB level of theory. The scan range was 1.1~1.9 and 1.5~2.3 Å, respectively, with a step length of 0.1 Å. Note that we intentionally extended the scan to bond lengths that are much shorter than the equilibrium bond lengths, to ensure that we do not encounter artificial potential energy minima at very short bond lengths, as we have previously observed for some other uncommon types of bonds.[120]

(2) For each structure along the scanned path, we calculated the reference single point energy at the BP86-D3/SARC-ZORA-TZVP(Rh)/ZORA-def2-TZVP(Si)/ZORA-def2-SVP(C,H,O,N) level. For **5**, we did not use a broken symmetry treatment because the closed shell singlet wavefunction is stable throughout the studied bond length range.

(3) We scaled the off-diagonal H_0 matrix elements between hydrogen and rhodium, as well as between silicon and carbon, by various factors, using the "$pairpar" field of the GFN2-xTB parameter file, and then re-calculated the single point energies along the scanned path using the tuned GFN2-xTB method. Specifically, we:

    a. copied the file "param_gfn2-xtb.txt" under the share/xtb folder of the xtb 6.5.1 program package to the folder where the ORCA input file (*vide infra*) was situated, and renamed it to "param.txt";

    b. added the following lines in "param.txt" after the first occurrence of "$end":

        $pairpar
        6 14 ***x***
        1 45 ***y***
        $end

    where ***x*** and ***y*** are numbers ("scale factors") that are close to 1;

c. added the following line to an ORCA input file of a GFN2-xTB single point calculation, and executed the input file to obtain the energy at a given structure:

%xtb xtbinputstring "--vparam param.txt" end

A grid search of the scale factors was then carried out with a step length of 0.01, and the scale factors that gave the best agreement (in the least-squares sense) with the DFT curves were then used in the actual MD simulations (due to the large difference in the absolute DFT and GFN2-xTB energies, we shifted the GFN2-xTB curve so that its minimum coincided with the DFT one before comparing the two curves). The best values were found to be $\boldsymbol{x}$ = 1.04 and $\boldsymbol{y}$ = 0.91, corresponding to increasing the Si-C off-diagonal H_0 matrix elements by 4% and decreasing the Rh-H off-diagonal H_0 matrix elements by 9%. Figure S71 shows the difference of the GFN2-xTB potential energy curves with the DFT ones, before and after the tuning of the $\boldsymbol{x}$ and $\boldsymbol{y}$ parameters. It can be seen that tuning the two parameters improves the agreement of the GFN2-xTB and DFT potential energy curves dramatically, especially near the equilibrium structures; in particular, the parameter tuning reduced the Rh-H bond length underestimation and Si-CH_2CH_2· bond length overestimation of the original GFN2-xTB method. Preliminary MD simulations showed that the latter in particular leads to dissociation of C_2H_4 from **7** to yield the silyl radical **8** within a few picoseconds, even when DLPNO-CCSD(T)//BP86-D3 gives a ΔG of +15.8 kcal·mol^{-1} for this process (i.e. no such reaction should be observable on the picosecond timescale at room temperature). We believe the current $\boldsymbol{x}$ and $\boldsymbol{y}$ parameters may be also applicable to other reactions involving rhodium(III) hydrides or the addition of silyl radicals to alkenes. To be on the safe side, however, we suggest benchmarking our tuned GFN2-xTB method against DFT energy data of the system to be studied, before using our parameters for a different molecular system.

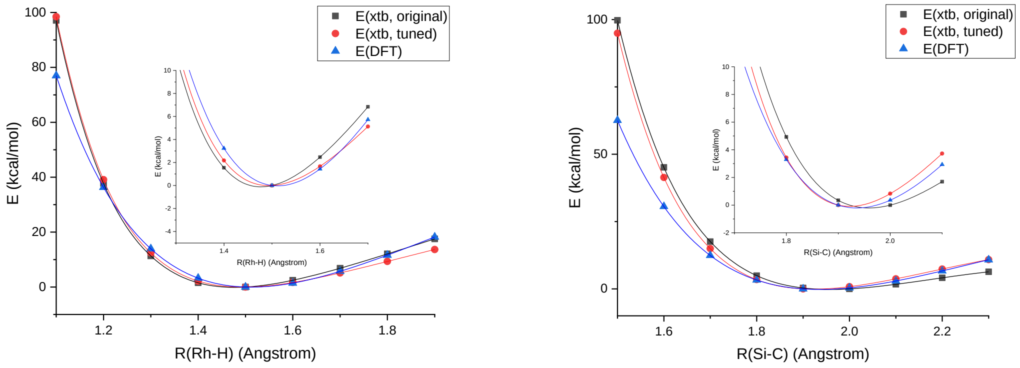

Figure S71. Comparison of the GFN2-xTB potential energy curves of **5**'s Rh-H bond (left) and **7**'s Si-CH_2CH_2· bond (right) with DFT results, before and after tuning the semiempirical parameters $\boldsymbol{x}$ and $\boldsymbol{y}$. The raw data were interpolated by 3rd-order splines. Insets: zoomed-in views around the equilibrium structures.

MD simulations were then carried out using the improved GFN2-xTB method (the ALPB solvation model[121-122] was used, with benzene as solvent). For each of **6-P** and **13-P**, 100 MD trajectories with different random seeds but otherwise identical settings were collected. In each MD simulation, we took **6-P** or **13-P** as the initial structure, and equilibrated the system with a 10 ps run at 298 K, with the RhH…Si distance fixed (so that no reaction could not take place, because the radical **7** could not flip over to point its radical carbon towards the Rh-H bond, nor can **7** drift away from **5**); this serves to obtain a representative Boltzmann sample of the complex of **5** and **7**. Then, the distance constraint was lifted, and a 50 ps

production run was carried out. MD runs that failed due to e.g. SCF convergence failure were discarded; since they consist of only 3 % of all trajectories, this procedure is not expected to introduce an appreciable bias into the results. The end products of the successful trajectories were then classified by manual inspection (Figure S72 and Figure S73).

We note that our results predict that about 10 % of the **7** radicals undergo addition to the porphyrin ligand. Since the catalyst Rh(II)-**3** can achieve turnover numbers much larger than 10, we conclude that either our calculations overestimated the probability of these reaction pathways (which is expected since we did not retune the semiempirical parameters related to C-C bond formation), or the radical additions are reversible at timescales much longer than 50 ps.

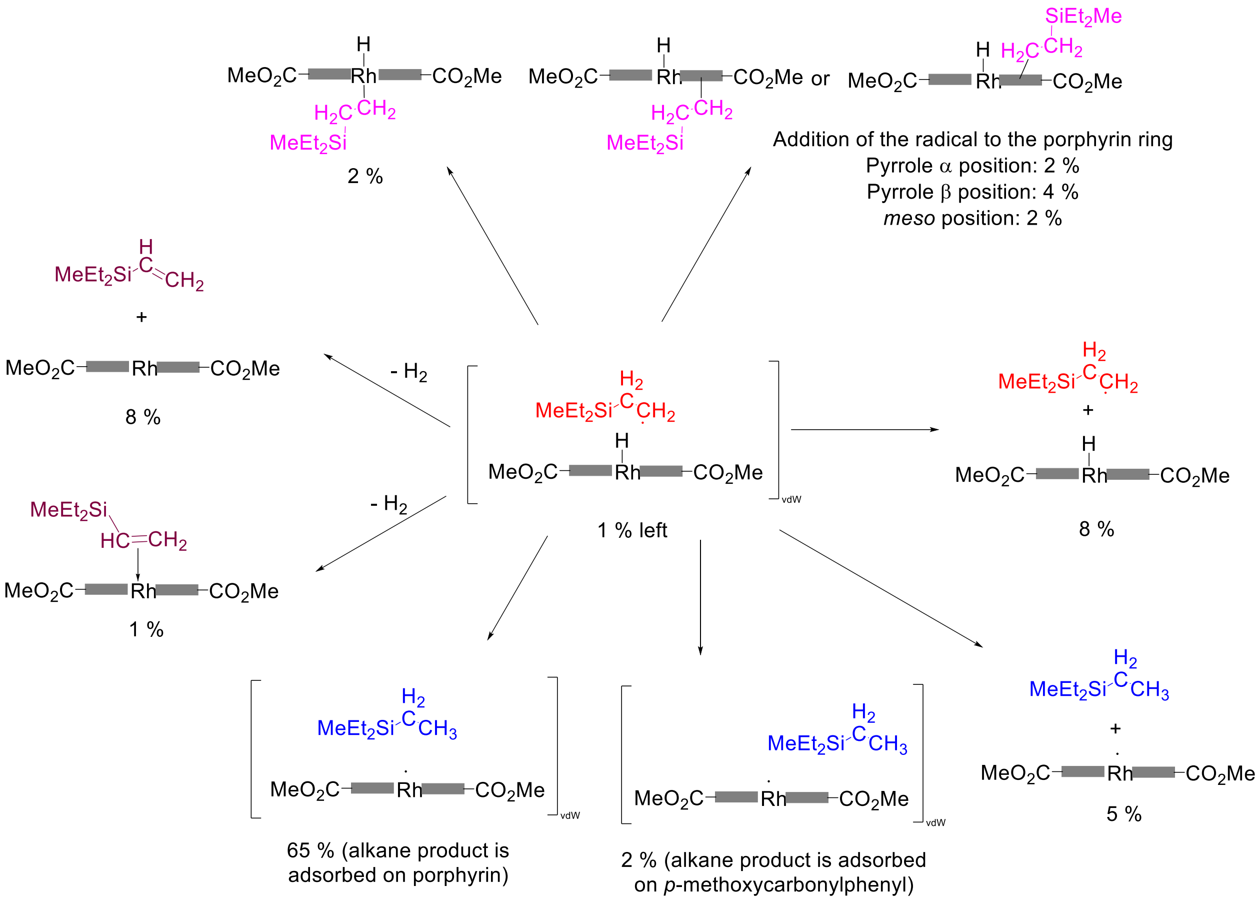

Figure S72. Fate of **6-P** after 50 ps of MD simulation.

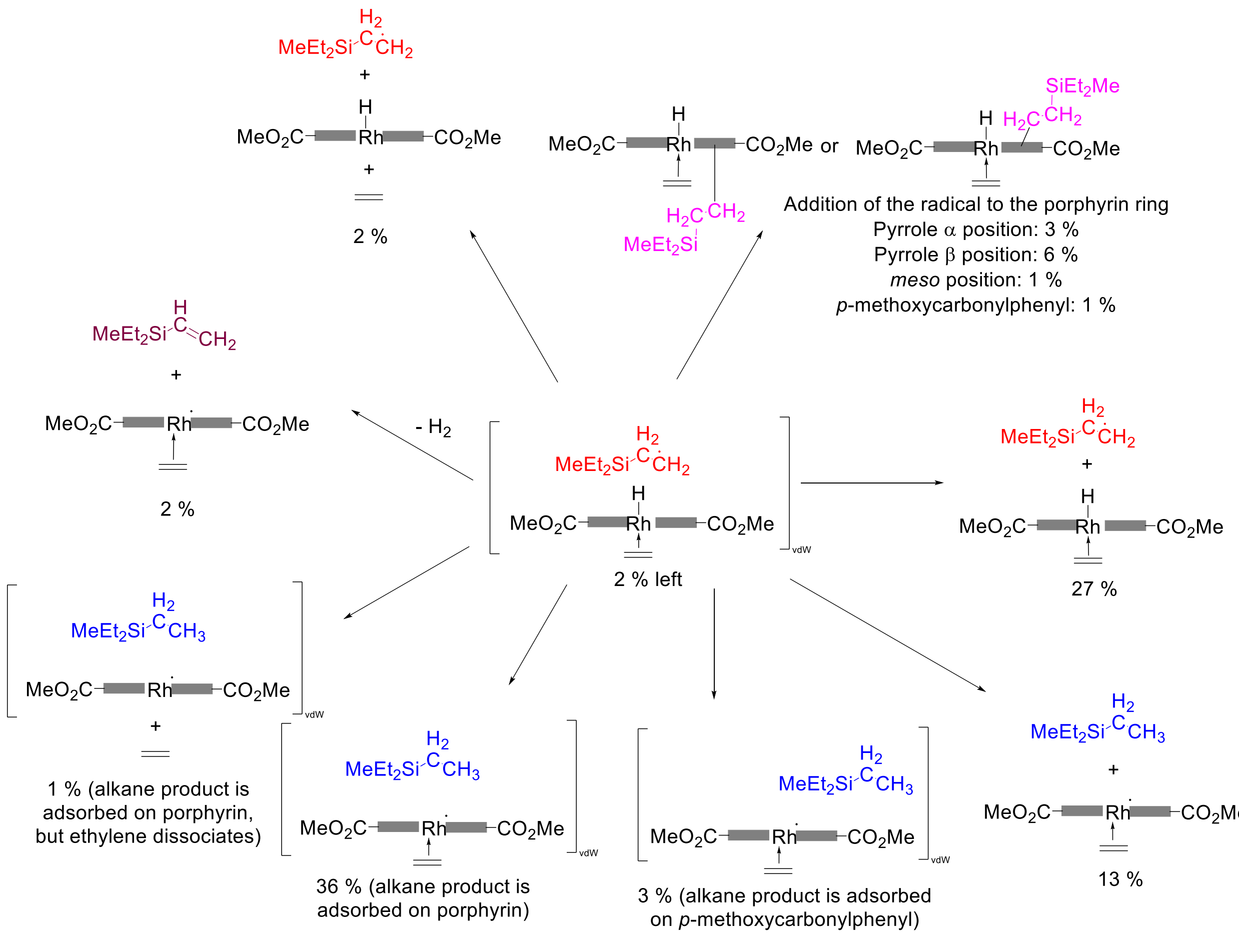

Figure S73. Fate of **13-P** after 50 ps of MD simulation.

The overall reaction profile of Rh(II)-**3** under dark and light conditions can thus be summarized in Figure S74. Both pathways begin from the formation of **6** from Rh(II)-**3** and silane. The light pathway then traverses a high H-Si bond breaking barrier (**6-TS-light**) that is beyond reach of room-temperature dark reactions, but can be crossed with the help of a 390 nm photon (which has an energy of 73.3 kcal·mol^{-1}). Subsequently, the silyl radical **8** is formed, which adds barrierlessly to ethylene to yield **7**; **7** then abstracts the Rh-H hydrogen atom from **5** in another barrierless process to regenerate Rh(II)-**3** and give the hydrosilylation product. By comparison, the dark pathway involves coordinating an ethylene molecule onto **6** to yield **13**, or coordinating a silane molecule onto **6** to yield **6**-silane; both can react with another ethylene molecule, taking advantage of inter-site electron transfer and the polarization effects of the MOF to reduce the barrier to a value that permits near-room-temperature reaction. The product complex **13-P** is a non-covalent complex of **7** and the ethylene complex of **5**. While **7** can dissociate and react with another molecule of **5**, it is more common for the radical **7** to abstract the Rh-H hydrogen atom within the same non-covalent complex, and yield the hydrosilylation product essentially barrierlessly within picosecond timescale.

As the rate determining step of the dark reaction is the reaction of **13** with C_2H_4, the KIE can be calculated by substituting the $SiMeEt_2$ group of this reaction to $SiEt_3$ groups (consistent with the experimental use of the $HSiEt_3$ and $DSiEt_3$ substrates for the KIE experiments), and calculating the ratio of the transition state theory rate constants using $HSiEt_3$ and $DSiEt_3$. This gives a KIE of 1.9.

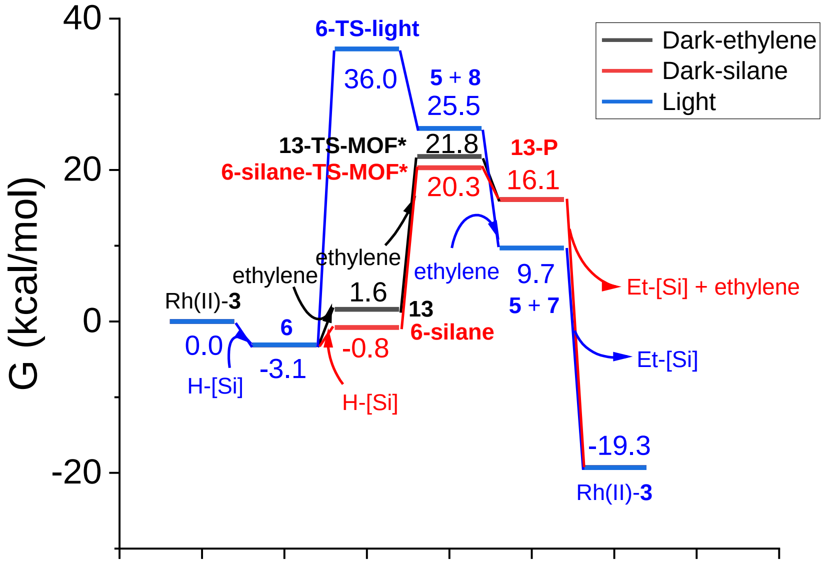

Figure S74. Reaction profiles of Rh(II)-**3**-catalyzed reaction of $HSiMeEt_2$ (H-[Si]) and ethylene. *The dielectric constant of the MOF (3.00) was used, but without considering the effect of Rh-OOH groups.

19 Lower Selectivity of Molecular Analogue

In addition to the substantial effect on the efficiency of catalytic hydrosilylation, site-isolation in the MOF also has a notable effect on the selectivity of the reaction. We showed earlier that light-mediated hydrosilylation with MOF-based catalysts **3** or **4** proceeded with 99% selectivity, while homogeneous mimic **18** only furnished the hydrosilylation product with 67 – 74% selectivity (Figure S75). Homogeneous mimic **18** is unable to catalyze thermal hydrosilylation, and in the absence of light, **18** was converted to a mixture of **19**, **20** as well as small amounts of the rhodium silyl complex, **21**. Based on our studies with MOF-based catalysts **3** and **4**, we could conclude that isolated Rh(II) sites provide 95% conversion and 99% selectivity for the formation of the hydrosilylation product after irradiation for 20 h. For the molecular model system, **18**, however, the concentration of Rh(II) porphyrin is constantly depleted via the rapid formation of **19**, **20** and **21**. Since the formation of **19**, **20** and **21** is irreversible at moderate temperatures in the absence of light, no thermal hydrosilylation is observed. In the presence of light, the formation of Rh(III)–C and Rh(III)–Si becomes reversible, but the reaction rates remain low because the concentration of the catalytically active species is constantly depleted via the exothermic and kinetically facile formation of **19**–**21**. Since not only the rate, but also the selectivity is substantially reduced when a molecular catalyst

is used, a MOF-analogous mechanism proceeding at **18** cannot be the only pathway that gives rise to conversion. To test whether additional reaction pathways are operative based on **19** and **20**, which are the dominant Rh-containing species present in solution, we prepared **19** and **20**. Use of **19** as the catalyst in light-mediated hydrosilylation of ethylene furnished 7% conversion after 20 h and 57% selectivity, while **20** provided 7% conversion after 20 h and 62% selectivity. The results are consistent with a fast and selective pathway that is promoted by **18**, which is only present in very low concentrations, in addition to slower and less selective hydrosilylation catalysis provided by the dominant species in solution, **19** and **20.**

Interestingly, we also observed substantial variation in the selectivity obtained with MOF-based catalyst Rh(II)-**3** depending on the reaction conditions. Hydrosilylation in the absence of light proceeded with 84 % selectivity for the hydrosilylated product at 50 °C, compared with 99% for the light-promoted reaction at room temperature. When we carried out the light-mediated reaction in the absence of external cooling a vessel temperature of 45 – 50 °C was reached. Under these conditions, hydrosilylation proceeded with 93% selectivity. We account for the different selectivity outcomes in the following manner: In the dark, only the direct silyl radical transfer pathway is energetically accessible and this pathway is associated with only moderate selectivity. Light-mediated transformations at room temperature proceed predominantly via the intermediacy of free silyl radicals, while light-mediated transformations at 50 °C have include a more substantial thermal contribution to the reaction rate. In the thermal pathway, electron transfer between adjacent Rh centers in the MOF lowers the activation barrier and introduces a partial positive charge on the terminal carbon atom, which favors the loss of a proton to generate the dehydrosilylated product (Fig. 9C). Interestingly, in 1983 Faltynek and coworkers reported a notably higher selectivity for the 1-pentene hydrosilylation under photochemical compared to thermal conditions for the same catalyst.[123] The authors attributed the selectivity difference to a switch from an organometallic mechanism for the photochemical pathway to a radical mechanism for the thermal pathway. In our case the mechanistic differences between the light-mediated and thermal pathway are more subtle, but a similar mechanism-driven selectivity difference is observed. Attributing an increase in the formation of the dehydrosilylated product to the temporary removal of an electron from the transition structure is consistent with our earlier observation that more dehydrosilylated product was observed for reactions in which oxygen was accidentally introduced.[61]

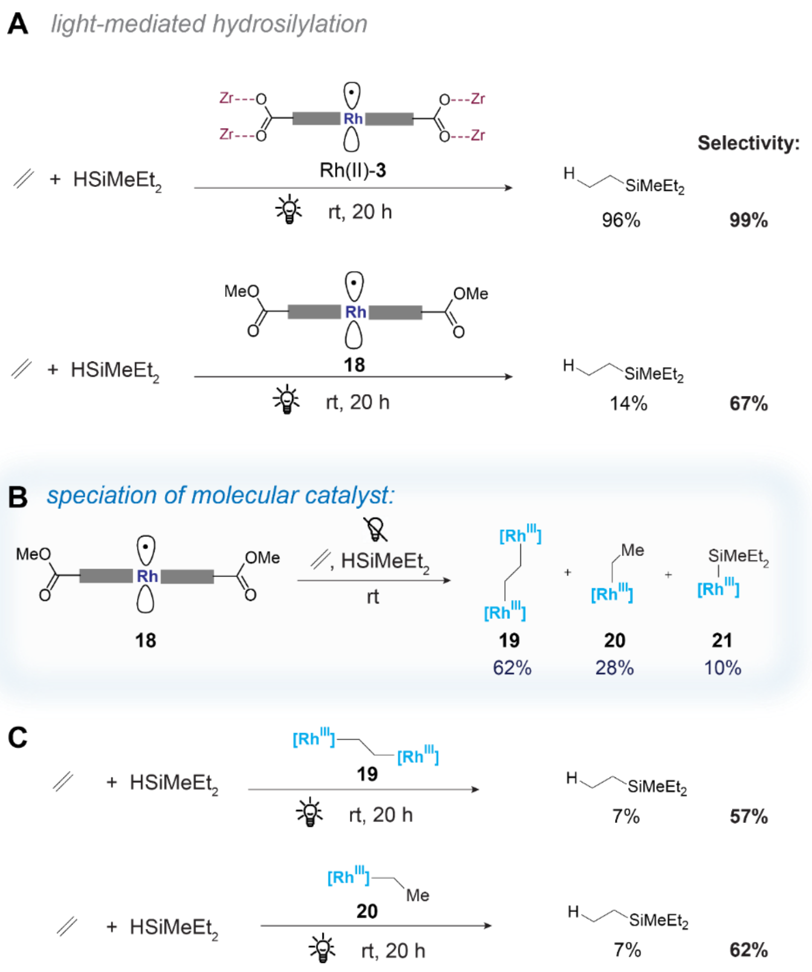

Figure S75. (A) Light-mediated hydrosilylation with molecular or MOF-supported Rh(II) in benzene provides highly divergent selectivity. (B) Rh speciation in the presence of ethylene and silane. (C) Selectivity dependence on structure of pre-catalyst. For the sake of simplicity, **18** is depicted as a monomer, which is the minority species in solution due to the reversible formation of a dimer that is held together by a weak Rh–Rh bond (~16.5 kcal·mol^{-1}).[124]

20 Kinetic Isotope Effect

The kinetic isotope effect (KIE) was determined by intermolecular competition: an equimolar amount of labelled and unlabeled silane was allowed to react with olefin in the presence of Rh(II)-**3** and the amount of hydrogen and deuterium incorporated into the product was determined by mass spectrometry.

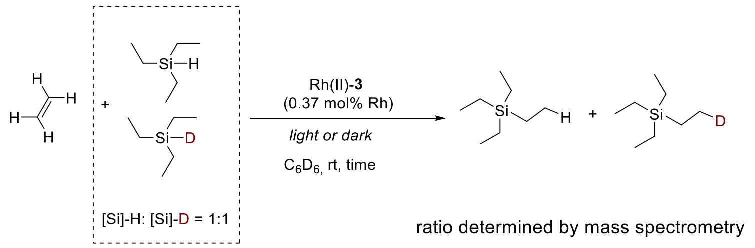

20.1 Light-Mediated Hydrosilylation

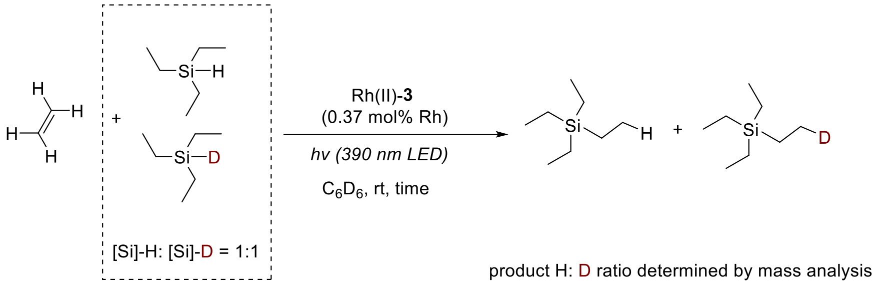

In an argon-filled glovebox, Rh(Me)-**3** (1.0 mg, 0.070 µmol, 0.37 mol%) and C_6D_6 (1.0 mL), were added to a 9 mL VWR glass vial containing a cross-type stir bar (5 x 10 mm). Then the vial was sealed with a screw cap that contains a septum, and transferred out of the glovebox. The reaction mixture was subjected to sonication for 1 minute in order to evenly suspend the MOF crystallites throughout the solvent. After that, Rh(Me)-**3** was subjected to photolysis to produce active catalyst Rh(II)-**3** with a 390 nm purple LED for 20 h at room temperature and a stirring rate of 500 rpm, while the reaction was cooled by in-house compressed air flow.

The reaction vial was transferred into glovebox, Et_3Si-H (15.2 µL, 95.4 µmol, 135 equiv) and Et_3Si-D (15.2 µL, 95.4 µmol, 135 equiv) were added to the 9 mL glass vial. Then the vial was sealed with a screw cap that contains a septum, and transferred out of the glovebox. The reaction mixture was subjected to sonication for 1 minute in order to evenly suspend the MOF crystallites throughout the solvent. Ethylene was then bubbled into the reaction vial for 1 minute in order to saturate the solvent with ethylene. To prevent oxygen leaking into the reaction mixture as ethylene was introduced, the vent needle that released excess ethylene from the reaction vial was connected to an inert gas manifold which was connected to an oil bubbler. The reaction was stirred at 500 rpm at room temperature and irradiated with a 390 nm purple LED, where the reaction was cooled by in-house compressed air flow. At 15 min, 30 min, 45 min, and 60 min reaction time, the reaction vial was subsequently subjected to centrifugation at 4500 rpm for 2 min (to ensure that the MOF particles settle down on the bottom of reaction vial), and 0.05 mL clear solution sample was removed via syringe from the reaction vial under the protection of ethylene gas. The four samples were subjected to GC-MS analysis to determine the H/D ratio in the hydrosilylated product via a comparison of the molecular weight (MW) patterns. An example of the mass spectroscopic analysis is shown in Figure S76. As the overall reaction conversion was low (only around 21% after 60 min, determined by ^{29}Si NMR in Figure S77), the hydrosilylated product H/D ratios in the above four samples could be directly used to represent $KIE_{H/D}$ values. The corresponding data is summarized in Table S24, and the KIE value was calculated to be KIE = 1.90 ± 0.25.

```
No.  MW.  Comment

 1   142  Overlapping with MW:150, Heteroatoms: Cl1
          Compare NJ109932
 2   144  Compare U68088
          D0: 61.8 +/- 1.5
          D1: 37.3 +/- 1.5
```

```
21.06.2023
File: E43986a-00.raw
Analyse: QIZ-QB-153-02
NEC: Qiu, Zihang
Messung:        GC-MS
Ionisierung:    GC-EI
Spektrometer:   ISQ
Säule:          MS 26 Vf-1ms
Länge:          30
Temp.:          30-10-10-280
GC-Nr.:         -
MS-Nr.:         -
Auswerter:      Margold (2242)
```

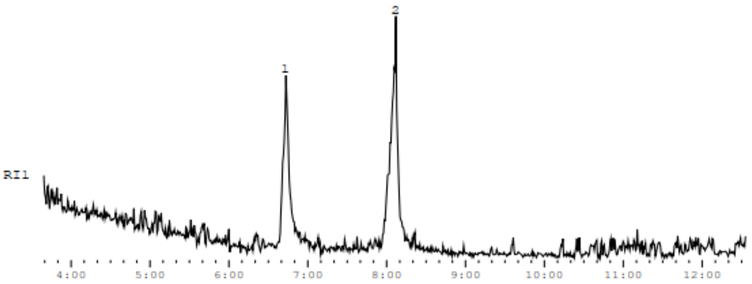

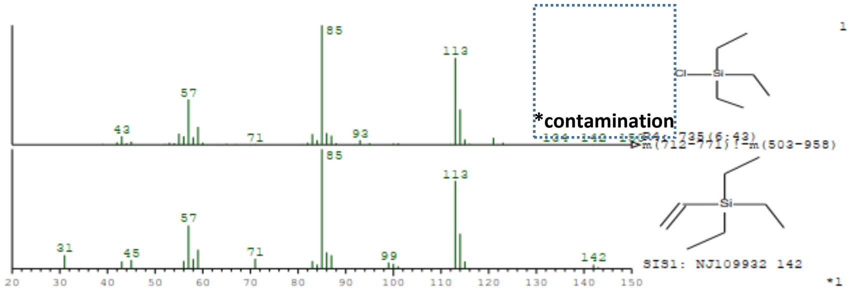

37	0.02	61	0.28	83	8.97	107	0.03	142	0.38
38	0.01	62	0.07	84	3.90	108	0.00	143	0.01
39	0.57	63	0.32	85	100.00	111	0.09	144	0.03
41	0.04	64	0.19	86	9.83	112	0.49	147	0.02
42	1.97	65	0.70	87	7.62	113	72.63	148	0.07
43	7.18	66	0.12	88	1.36	114	29.58	150	0.36
44	1.25	67	0.44	89	0.11	115	4.46	151	0.03
45	2.60	68	0.04	91	0.12	116	0.67	152	0.10
46	0.07	69	0.18	92	0.31	117	0.02	153	0.05
48	0.03	70	0.30	93	3.88	118	0.00	155	0.01
49	0.02	71	0.49	94	0.09	119	0.00	156	0.01
51	0.01	72	0.05	95	1.28	120	0.08	159	0.03
52	0.55	73	0.06	96	0.10	121	5.83		
53	1.72	74	0.09	97	0.04	122	0.38		
54	1.21	76	0.04	98	0.01	123	1.69		
55	9.29	77	0.01	99	0.13	124	0.10		
56	7.11	78	0.01	100	0.95	125	0.03		
57	38.01	79	0.01	101	1.17	133	0.01		
58	6.35	80	0.08	102	0.13	134	0.02		
59	14.93	81	0.40	105	0.05	138	0.01		
60	1.70	82	1.72	106	0.03	140	0.01		

R4: 735(6:43) m(712-771) E43986a-00! -m(503-958) 2 28% E43986a-00 * QIZ-QB-153-02/GC-MS/GC-

22.06.2023 09:41 p.2/4 *** Angegebene Mol.-Gewichte u. Massenzahlen basieren auf dem häufigsten Isotop der Elemente *** **MassLib**

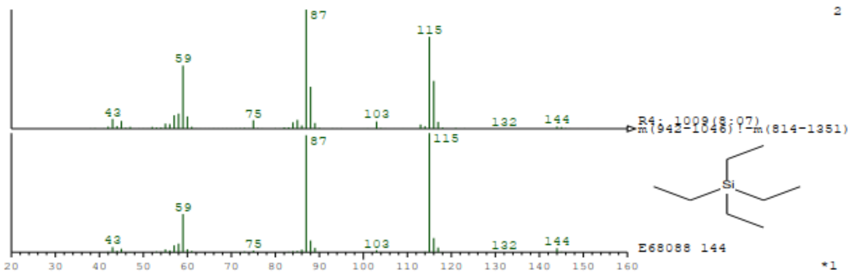

34	0.00	59	53.03	81	0.08	105	0.12	131	0.03
38	0.16	60	10.34	82	1.09	107	0.03	132	0.11
39	0.25	61	1.59	83	1.11	108	0.00	133	0.01
41	0.09	62	0.13	84	5.35	109	0.06	134	0.00
42	1.74	63	0.07	85	7.41	110	0.02	136	0.02
43	8.21	64	0.04	86	2.73	111	0.02	143	0.02
44	2.18	65	0.05	87	100.00	112	0.02	144	1.84
45	6.53	66	0.15	88	35.22	113	3.57	145	1.37
46	0.74	67	0.11	89	4.79	114	2.01	146	0.24
47	1.52	68	0.08	90	0.61	115	77.13	147	0.04
48	0.03	69	0.12	91	0.06	116	40.15	150	0.03
49	0.12	70	0.12	93	0.18	117	5.77	151	0.04
50	0.14	71	0.20	94	0.10	118	0.90	152	0.01
51	0.11	72	0.27	95	0.14	119	0.04	158	0.01
52	1.48	73	0.50	96	0.01	120	0.01		
53	0.89	74	0.24	98	0.02	121	0.50		
54	1.02	75	7.16	99	0.01	122	0.03		
55	4.31	76	0.35	100	0.04	123	0.15		
56	3.93	77	0.12	101	0.15	124	0.03		
57	11.28	79	0.03	103	6.06	128	0.01		
58	12.70	80	0.20	104	0.37	130	0.01		

R4: 1009(8:07) m(942-1046) E43986a-00! -m(814-1351) 2 23% E43986a-00 * QIZ-QB-153-02/GC-MS/

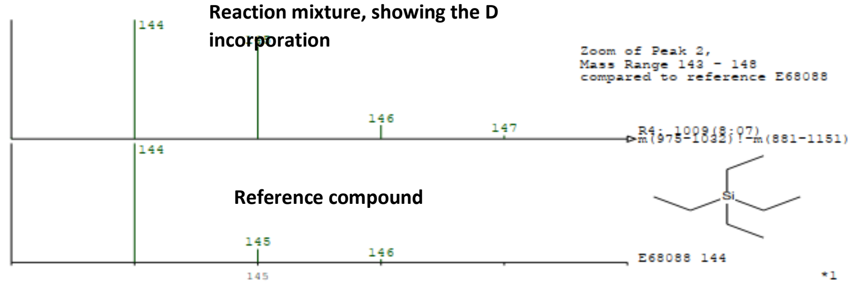

MassLib V9.4 *** Angegebene Mol.-Gewichte u. Massenzahlen basieren auf dem häufigsten Isotop der Elemente *** MPI für Kohlenforschung

Figure S76. GC-MS spectrum for determining the H/D ratio in the hydrosilylated reaction product.

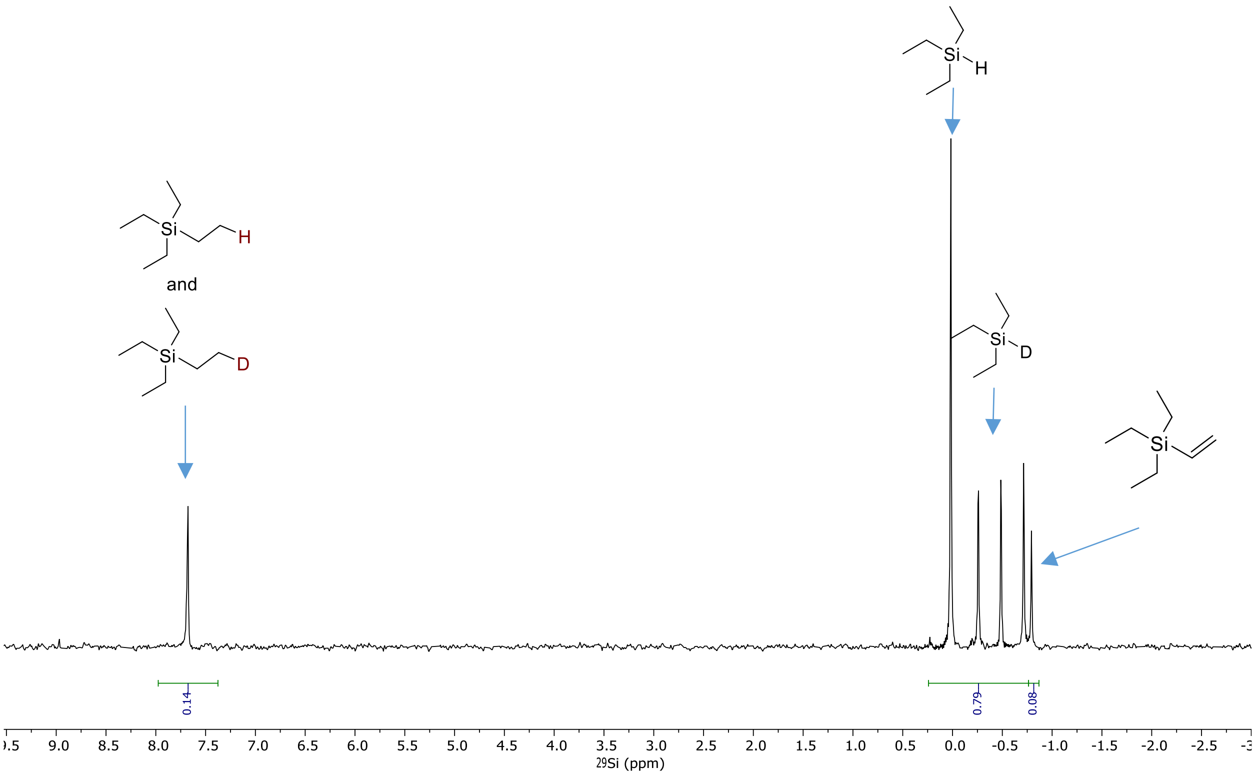

Figure S77. $^{29}Si\{^1H\}$ NMR spectrum of reaction mixture.

Table S24. Product H/D ratios detected at different time points.

Entries	15 min	30 min	45 min	60 min	Average
H (D0)-product (%)	65.9	61.8	67.9	68.9	66.125
D (D1)-product (%)	36.6	37.3	33.5	32.1	34.875
H (D0)-product uncertainty (%)	4.8	1.5	3.7	2	3
H (D0)-product fractional uncertainty	0.0728	0.0242	0.0545	0.0290	0.0454
D (D1)-product uncertainty (%)	4.8	1.5	3.7	2	3
D (D1)-product fractional uncertainty	0.131	0.0402	0.110	0.0623	0.0860
(for calculation of KIE value see below)					

$$KIE = \frac{average\ H\ proudct}{average\ D\ product} = \frac{66.125}{34.875} = 1.90$$

$$KIE\ fraction\ \text{uncertainty} = 0.0454 + 0.0860 = 0.1314$$

$$KIE\ \text{uncertainty} = 1.90 * 0.1314 = 0.25$$

$$So\ KIE\ =\ 1.90\ \pm\ 0.25$$

20.2 Thermal Hydrosilylation in the Dark

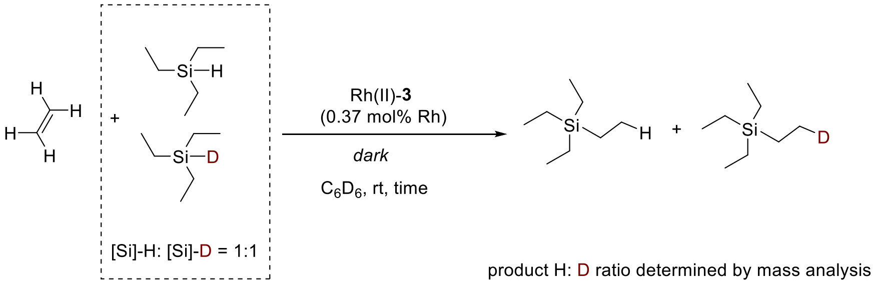

In an argon-filled glovebox, Rh(Me)-**3** (1.0 mg, 0.070 µmol, 0.37 mol%) and C_6D_6 (1.0 mL), were added to a 9 mL VWR glass vial containing a cross-type stir bar (5 x 10 mm). Then the vial was sealed with a screw cap that contains a septum, and transferred out of the glovebox. The reaction mixture was subjected to sonication for 1 minute in order to evenly suspend the MOF crystallites throughout the solvent. After that, Rh(Me)-**3** was subjected to photolysis to produce active catalyst Rh(II)-**3** with a 390 nm purple LED for 20 h at room temperature and a stirring rate of 500 rpm, while the reaction was cooled by in-house compressed air flow.

The reaction vial was transferred into glovebox, Et_3Si-H (15.2 µL, 95.4 µmol, 135 equiv) and Et_3Si-D (15.2 µL, 95.4 µmol, 135 equiv) were added to the 9 mL glass vial. Then the vial was sealed with a screw cap that contains a septum, and transferred out of the glovebox. The reaction mixture was subjected to sonication for 1 minute in order to evenly suspend the MOF crystallites throughout the solvent. Ethylene was then bubbled into the reaction vial for 1 minute in order to saturate the solvent with ethylene. To prevent oxygen leaking into the reaction mixture as ethylene was introduced, the vent needle that released excess ethylene from the reaction vial was connected to an inert gas manifold which was connected to an oil bubbler. The reaction was stirred at 500 rpm at room temperature under dark covered by aluminum foil. At 10 h, 15 h, 20 h, and 34 h reaction time, the reaction vial was subsequently subjected to centrifugation at 4500 rpm for 2 min (to ensure that the MOF particles settle down on the bottom of reaction vial), and 0.05 mL clear solution sample was removed via syringe from the reaction vial under the protection of ethylene gas. The four samples were submitted to GC-MS analysis to determine the H/D ratio in the hydrosilylated product via comparing the molecular weight (MW) patterns. An example of mass analysis is shown in Figure S76. As the overall reaction conversion was low (only around 7% after 34 h, determined by ^{29}Si NMR in Figure S78), the hydrosilylated product H/D ratios in the above four samples could be directly used to represent $KIE_{H/D}$ values. The corresponding data is summarized in Table S25, and the KIE value was calculated to be KIE = 1.85 ± 0.24.

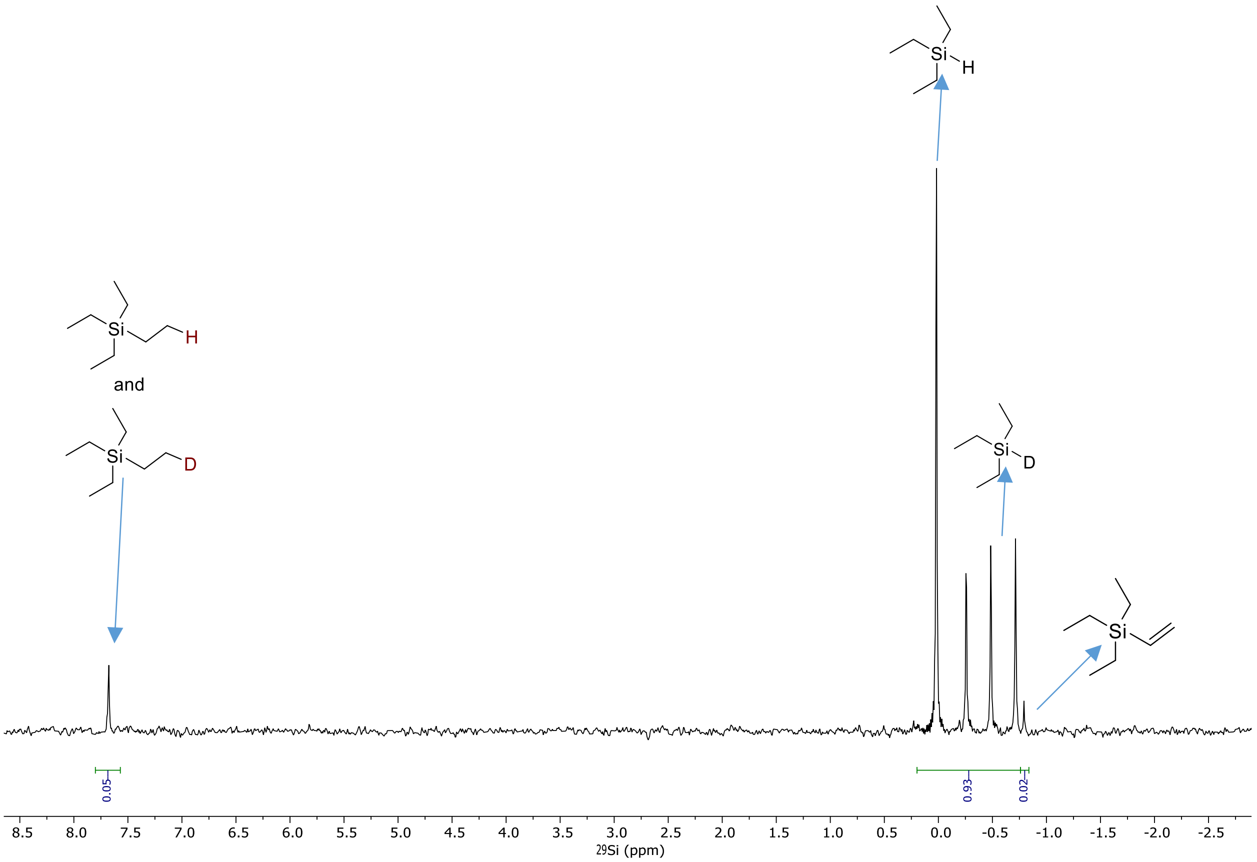

Figure S78. $^{29}Si\{^{1}H\}$ NMR spectrum of the reaction mixture.

Table S25. Summary of the H/D ratio of the products.

Entries	10 h	15 h	20 h	34 h	Average
H (D0)-product (%)	64.7	63.9	62.8	65.3	64.175
D (D1)-product (%)	34.3	35.4	35.4	33.6	34.675
H (D0)-product uncertainty (%)	3.1	1	2.8	4.8	2.925
H (D0)-product fractional uncertainty	0.0479	0.0156	0.0446	0.0735	0.0456
D (D1)-product uncertainty (%)	3.1	1	2.8	4.8	2.925
D (D1)-product fractional uncertainty	0.0904	0.0282	0.0791	0.143	0.0844

$$KIE = \frac{average\ H\ proudct}{average\ D\ product} = \frac{64.175}{34.675} = 1.85$$

$$KIE\ fraction\ \text{uncertainty} = 0.0456 + 0.0844 = 0.130$$

$$KIE\ \text{uncertainty} = 1.85 * 0.130 = 0.24$$

$$So\ KIE\ = \ 1.85\ \pm\ 0.24$$

21 Reaction Orders

Note: Throughout this section 'silane' stands for diethylmethylsilane; [hydrosilane] stands for the concentration of triethylmethylsilane; [cat.] stands for concentration of the catalyst.

21.1 Catalyst Order

In an argon-filled glovebox, Rh(Me)-**3** (10 mg, 7.1 µmol) and C_6D_6 (10 mL), were added to a 20 mL glass tube containing a cross-type stir bar (5 x 10 mm), then the viral was sealed with screw cap that contains a septum, and transferred out of the glovebox. Then 0.85 mL air was injected into the vial via syringe. The reaction mixture was subjected to sonication for 1 minute in order to evenly suspend the MOF crystallites throughout the solvent. After that, Rh(Me)-**3** was subjected to photolysis to produce active catalyst Rh(II)-**3** with a 390 nm purple LED for 20 h at room temperature and a stirring rate of 1000 rpm. The reaction mixture was cooled during photolysis using in-house compressed air flow. After 20 h, the reaction vial was subjected to centrifugation and transferred into the glovebox. C_6D_6 was removed and the remaining powder was dried under reduced pressure glovebox to generate the active Rh(II) catalyst.

In an argon-filled glovebox, the active Rh(II) catalyst (for amounts see Table S26) was added into a 4 ml glass vial along with diethylmethylsilane (90 µL, 0.62 mmol, 1.0 equiv), 1-pentene (163 µL, 1.49 mmol, 2.40 equiv) and 3 mL C_6D_6 and the vial was sealed with a cap. The reaction mixture was subjected to sonication for another 1 minute in order to evenly suspend the MOF crystallites throughout the solvent. The reaction was stirred at 50 ^{o}C at the stirring rate of 100 ppm in the dark inside the glovebox. After the given time passed (see Table S26), the vial was left to cool to room temperature, and 0.01 mL of the reaction mixture was carefully removed using a syringe and added into an NMR tube along with 0.5 mL C_6D_6.

Table S26. Kinetic data of hydrosilylation reaction catalyzed by different amounts of Rh(II)-**3**.

t(h)	Conversion (1.5 mg catalyst)	Conversion (3 mg catalyst)	Conversion (4.3 mg catalyst)
0	0	0	0
12.5	0.07	0.14	0.25
20	0.13	0.29	0.38
27	0.2	0.39	0.47
38	0.32	0.49	0.59
44.5	0.38	0.54	0.64

The Variable Time Normalization Analysis (VTNA) method was used to determine reaction orders.[125-126] According to the visualization analysis below (Figure S79, Figure S80, Figure S81 and Figure S82), a catalyst order of 0.9 was determined.

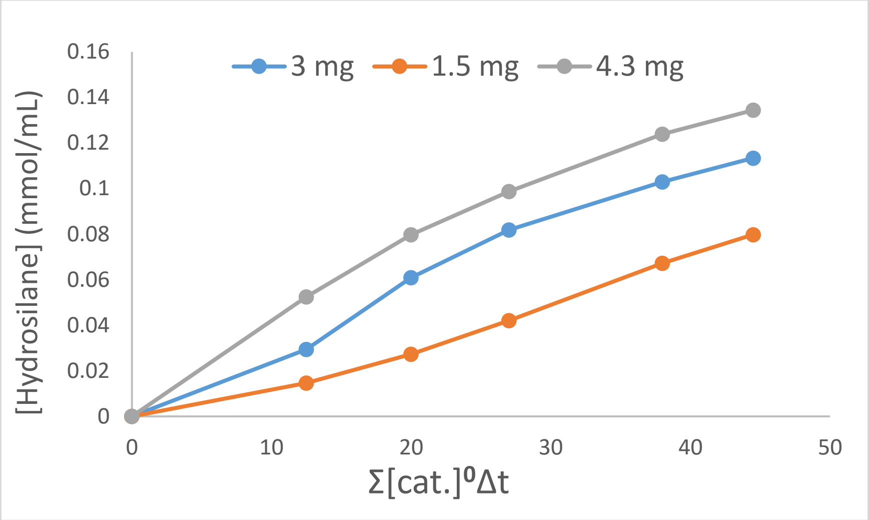

Figure S79. The time scale is substituted by Σ[cat.] $^{\alpha}$ Δt according to the VTNA method. The value of α that produces the overlay of all three reaction profiles is the catalyst order. Here α = 0.

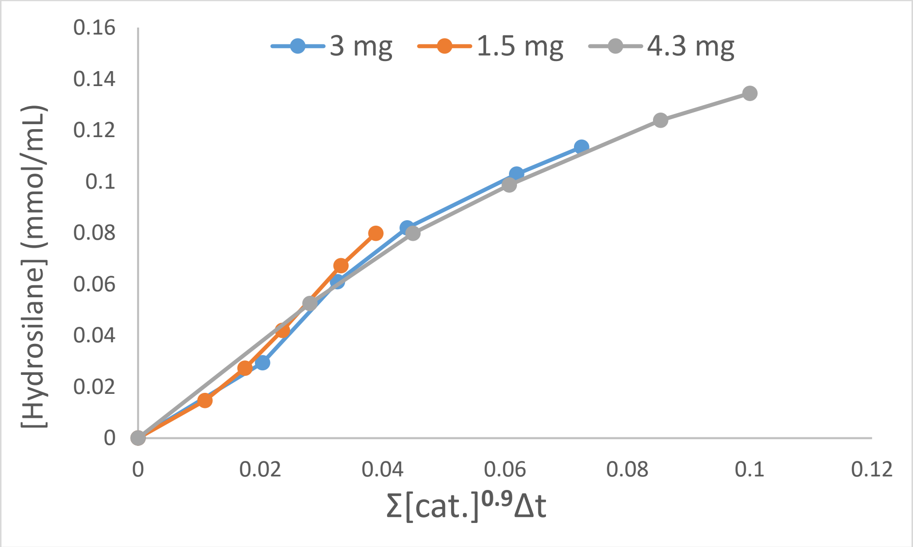

Figure S80. The time scale is substituted by Σ[cat.] $^{\alpha}$ Δt according to the VTNA method. The value of α that produces the overlay of all three reaction profiles is catalyst order. Here α = 0.9.

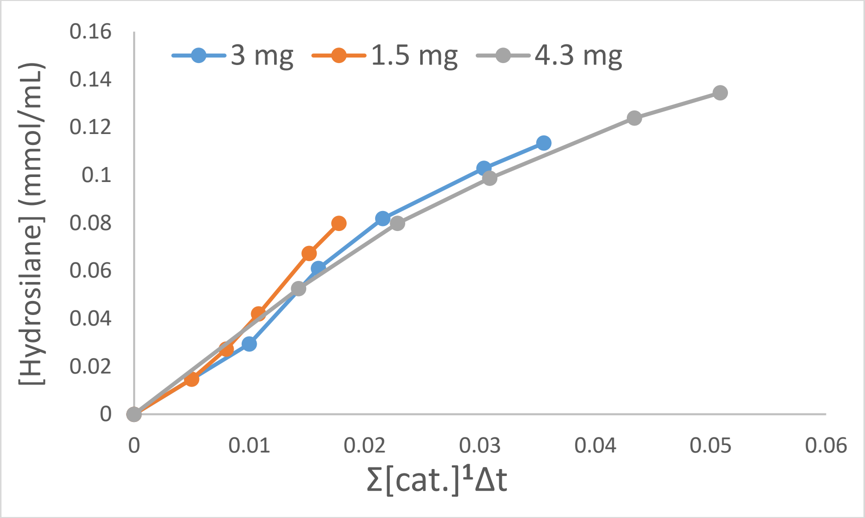

Figure S81. The time scale is substituted by Σ[cat.] $^{\alpha}$ Δt according to the VTNA method. The value of α that produces the overlay of all three reaction profiles is catalyst order. Here α = 1.

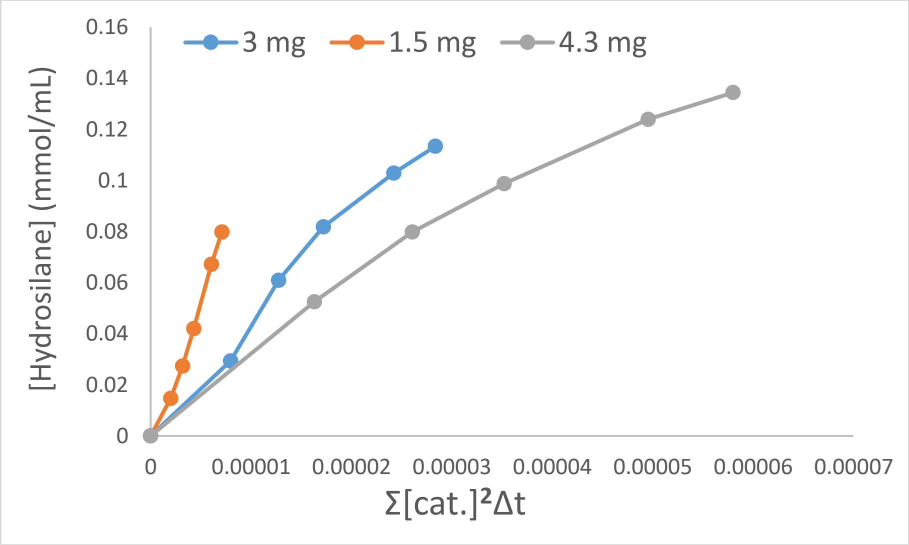

Figure S82. The time scale is substituted by Σ[cat.] $^{\alpha}$ Δt according to the VTNA method. The value of α that produces the overlay of all three reaction profiles is catalyst order. Here α = 2.

21.2 Silane Order

In an argon-filled glovebox, Rh(Me)-**3** (10 mg, 7.1 μmol) and C_6D_6 (10 mL), were added to a 20 mL glass tube containing a cross-type stir bar (5 x 10 mm), then the vial was sealed with screw cap that contains a septum, and transferred out of the glovebox. Then 0.85 mL air was injected into the vial via syringe. The reaction mixture was subjected to sonication for 1 minute in order to evenly suspend the MOF crystallites throughout the solvent. After that, Rh(Me)-**3** was subjected to photolysis to produce active catalyst Rh(II)-**3** with a 390 nm purple LED for 20 h at room temperature and a stirring rate of 1000 rpm. The reaction

mixture was cooled during photolysis using in-house compressed air flow. After 20 h, the reaction vial was subjected to centrifugation and transferred into the glovebox. C_6D_6 was removed and the remaining powder was dried under reduced pressure glovebox to generate the active Rh(II) catalyst.

In an argon-filled glovebox, the active Rh(II) catalyst (1 mg) was added into a 4 ml glass vial along with diethylmethylsilane (for amounts see Table S27), 1-pentene (163 µL, 1.49 mmol, 2.40 equiv) and 3 mL C_6D_6 and the vial was sealed with a cap. The reaction mixture was subjected to sonication for another 1 minute in order to evenly suspend the MOF crystallites throughout the solvent. The reaction was stirred at 50 °C at the stirring rate of 100 ppm in the dark inside the glovebox. After the given time passed (see Table S27), the vial was left to cool to room temperature, and 0.01 mL of the reaction mixture was carefully removed using a syringe and added into an NMR tube along with 0.5 mL C_6D_6.

Table S27. Kinetic data of hydrosilylation reaction catalyzed by Rh(II)-**3** using different amount of silane.

t(h)	Conversion (30 µL Silane)	t(h)	Conversion (72 µL Silane)	Conversion (108 µL Silane)
0	0	0	0	0
7	0.03	21.5	0.21	0.35
17.5	0.1	28	0.28	0.47
24	0.15	32.5	0.32	0.49
30	0.2	40.5	0.39	0.65
42	0.26	48	0.44	0.69
		55	0.51	0.78

The Variable Time Normalization Analysis (VTNA) method was used to determine reaction orders.[125-126] According to the visualization analysis below (Figure S83, Figure S84, Figure S85 and Figure S86), a silane order of 1.3 was determined.

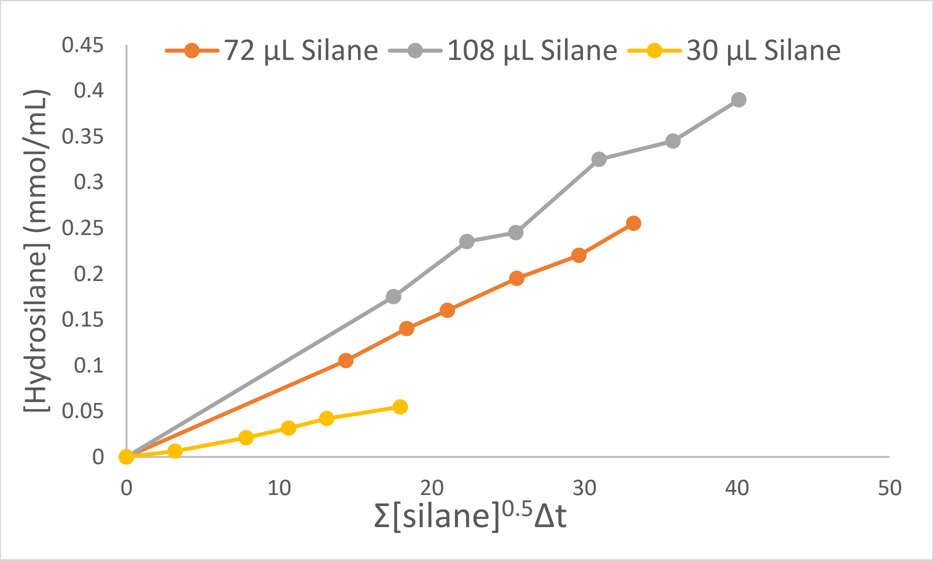

Figure S83. The time scale is substituted by Σ[silane]$^{\beta}$Δt according to the VTNA method. The value of β that produces the overlay of all three reaction profiles is silane order. Here β = 0.5.

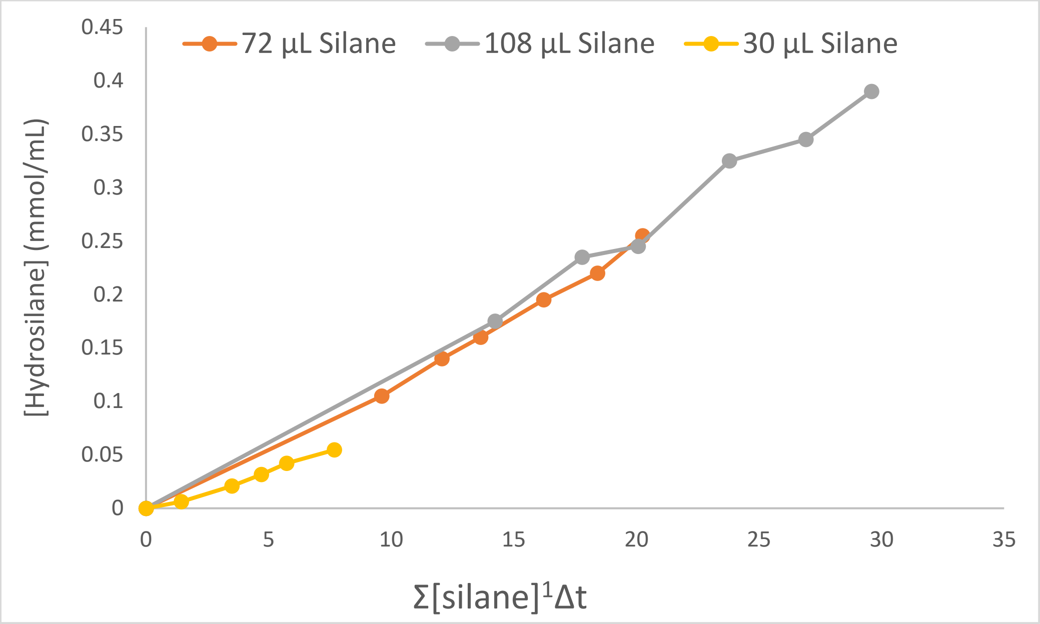

Figure S84. The time scale is substituted by Σ[silane]$^{\beta}$Δt according to the VTNA method. The value of β that produces the overlay of all three reaction profiles is silane order. Here β = 1.

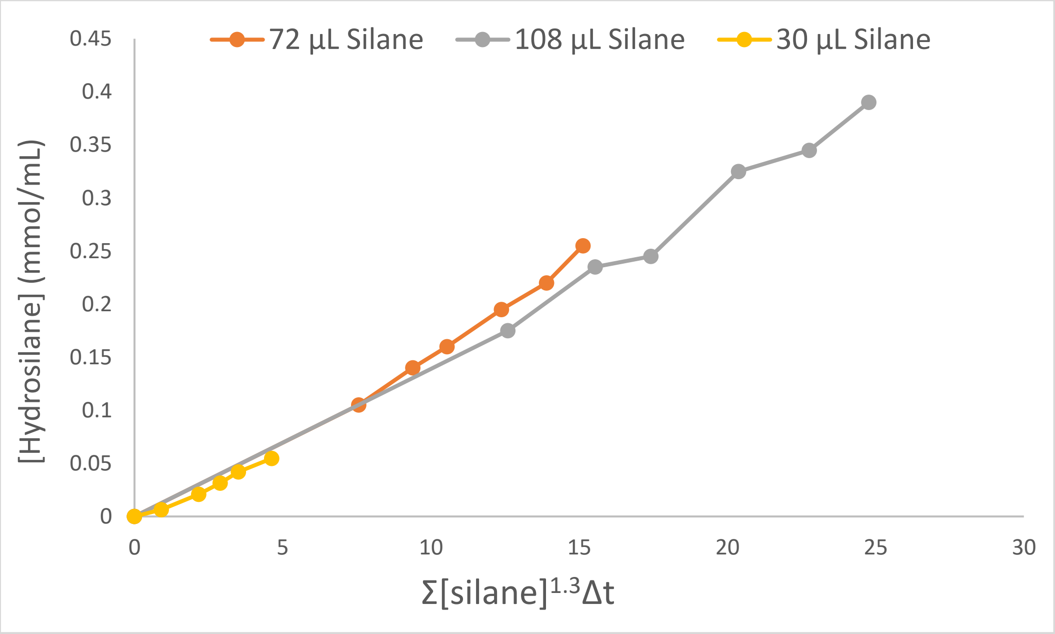

Figure S85. The time scale is substituted by Σ[silane]$^{\beta}$Δt according to the VTNA method. The value of β that produces the overlay of all three reaction profiles is silane order. Here β = 1.3.

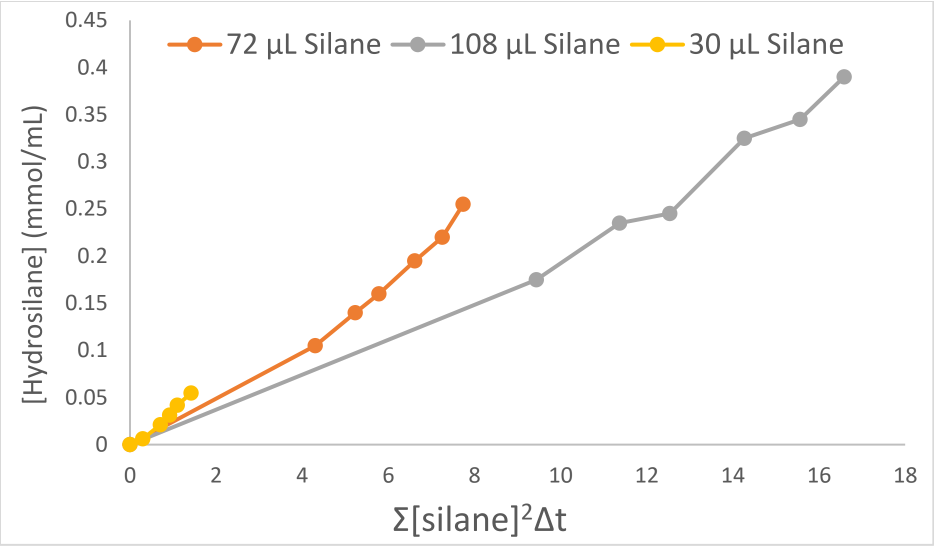

Figure S86. The time scale is substituted by $\Sigma[\text{silane}]^{\beta}\Delta t$ according to the VTNA method. The value of β that produces the overlay of all three reaction profiles is silane order. Here β = 2.

21.3 1-Pentene Order

In an argon-filled glovebox, Rh(Me)-**3** (15.0 mg, 11.8 µmol) and C_6D_6 (15 mL), were added to a 20 mL glass tube containing a cross-type stir bar (5 x 10 mm), then the vial was sealed with screw cap that contains a septum, and transferred out of the glovebox. Then 1.3 mL air was injected into the vial via syringe. The reaction mixture was subjected to sonication for 1 minute in order to evenly suspend the MOF crystallites throughout the solvent. After that, Rh(Me)-**3** was subjected to photolysis to produce active catalyst Rh(II)-**3** with a 390 nm purple LED for 20 h at room temperature and a stirring rate of 1000 rpm. The reaction mixture was cooled during photolysis using in-house compressed air flow. After 20 h, the reaction vial was subjected to centrifugation and transferred into the glovebox. C_6D_6 was removed and the remaining powder was dried under reduced pressure glovebox to generate the active Rh(II) catalyst.

In an argon-filled glovebox, the active Rh(II) catalyst (3 mg) was added into a 4 ml glass vial along with diethylmethylsilane (90 µL, 0.62 mmol, 1.0 equiv), 1-pentene (for amounts see Table S28) and 3 mL C_6D_6 and the vial was sealed with a cap. The reaction mixture was subjected to sonication for another 1 minute in order to evenly suspend the MOF crystallites throughout the solvent. The reaction was stirred at 50 °C at the stirring rate of 100 ppm in the dark inside the glovebox. After the given time passed (see Table S28), the vial was left to cool to room temperature, and 0.01 mL of the reaction mixture was carefully removed using a syringe and added into an NMR tube along with 0.5 mL C_6D_6.

Table S28. Kinetic data of hydrosilylation catalyzed by Rh(II)-**3** using different amounts of 1-pentene.

t(h)	Conversion (163 µL 1-pentene)	Conversion (271 µL 1-pentene)	Conversion (407 µL 1-pentene)
0	0	0	0
5	0.010	0.015	0.018
18.5	0.074	0.117	0.142

24.5	0.106	0.160	0.198
40.5	0.180	0.281	0.377
46.5	0.207	0.321	0.402

The Variable Time Normalization Analysis (VTNA) method was used to determine reaction orders.[125-126] According to the visualization analysis below (Figure S87, Figure S88 and Figure S89), a 1-pentene order of ~0.7 was determined.

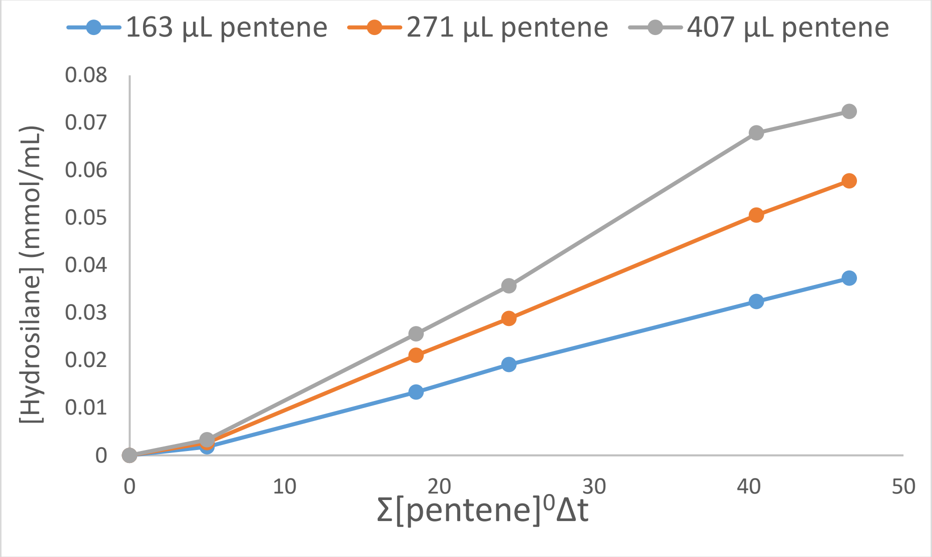

Figure S87. The time scale is substituted by Σ[silane]$^\gamma$Δt according to the VTNA method. The value of γ that produces the overlay of all three reaction profiles is 1-pentene order. Here γ = 0.

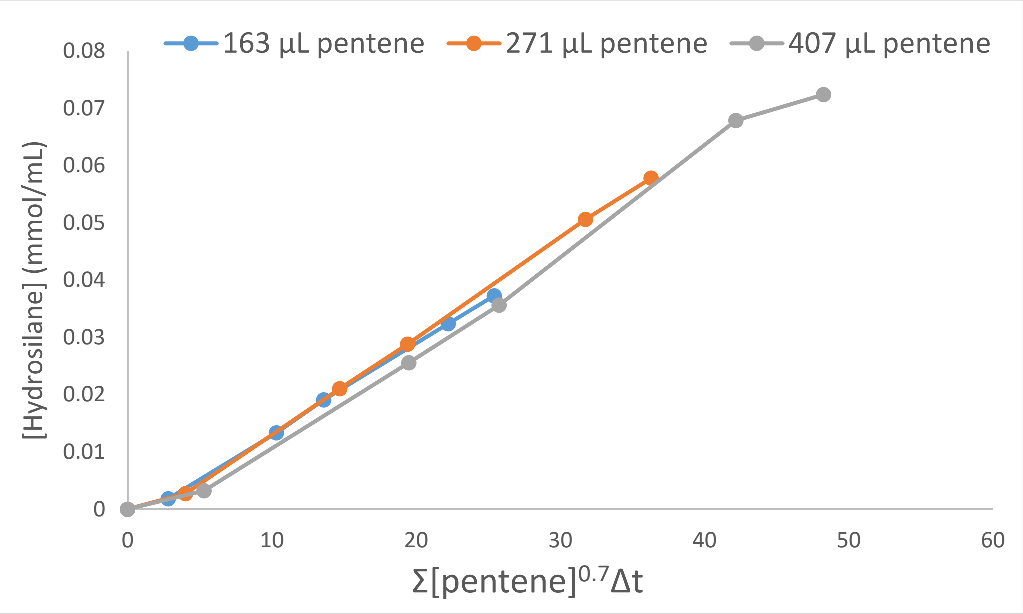

Figure S88. The time scale is substituted by Σ[silane]$^\gamma$Δt according to the VTNA method. The value of γ that produces the overlay of all three reaction profiles is 1-pentene order. Here γ = 0.7.

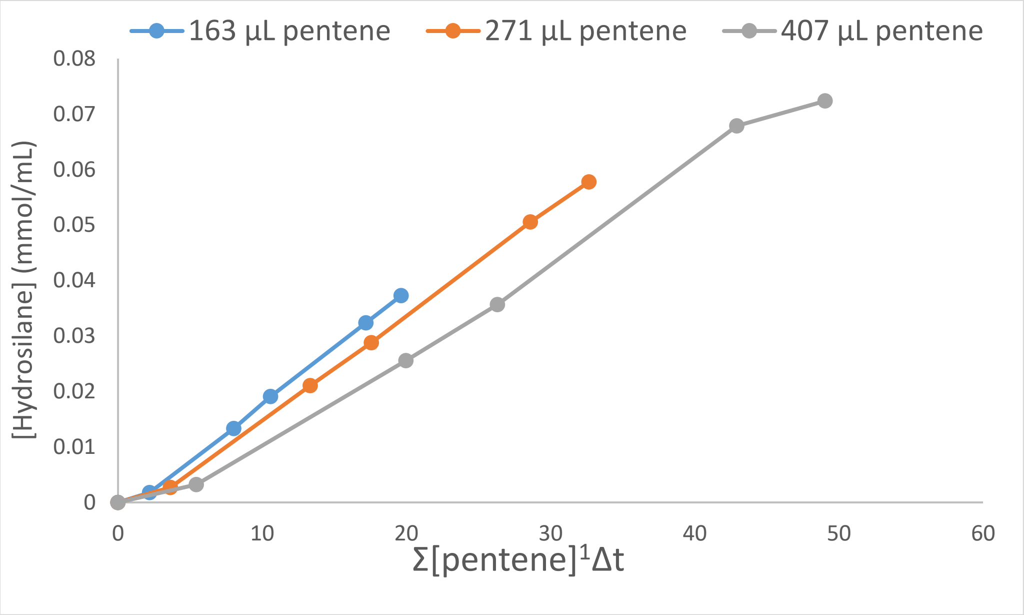

Figure S89. The time scale is substituted by Σ[silane]$^{\gamma}$Δt according to the VTNA method. The value of γ that produces the overlay of all three reaction profiles is 1-pentene order. Here γ = 1.

22 Eyring Analysis

22.1 Thermal Hydrosilylation at Different Temperatures

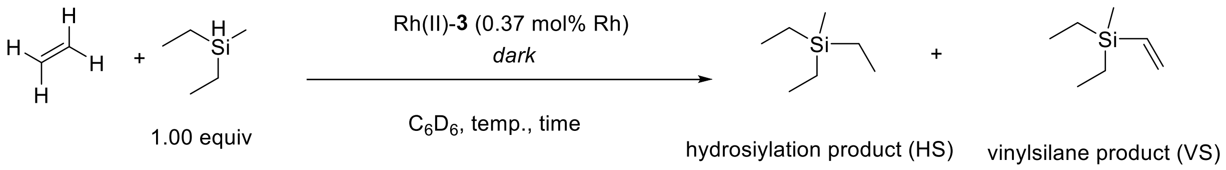

22.1.1 Reaction at 25 °C

In an argon-filled glovebox, Rh(Me)-**3** (1.0 mg, 0.070 µmol, 0.37 mol%) and C_6D_6 (1.0 mL), were added to a 9 mL VWR glass vial containing a cross-type stir bar (5 x 10 mm). Then the vial was sealed with a screw cap that contains a septum, and transferred out of the glovebox. The reaction mixture was subjected to sonication for 1 minute in order to evenly suspend the MOF crystallites throughout the solvent. After that, Rh(Me)-**3** was subjected to photolysis to produce active catalyst Rh(II)-**3** with a 390 nm purple LED for 20 h at room temperature and a stirring rate of 500 rpm, where the reaction was cooled by in-house compressed air flow.

The reaction vial was transferred into glovebox, diethylmethylsilane (27.7 µL, 191 µmol, 270 equiv) was added to the 9 mL glass vial. Then the vial was sealed with a screw cap that contains a septum, and

transferred out of the glovebox. The reaction mixture was subjected to sonication for 1 minute in order to evenly suspend the MOF crystallites throughout the solvent. Ethylene was then bubbled into the reaction vial for 1 minute in order to saturate the solvent with ethylene. To prevent oxygen leaking into the reaction mixture as ethylene was introduced, the vent needle that released excess ethylene from the reaction vial was connected to an inert gas manifold which was connected to an oil bubbler. Following the addition of ethylene, the reaction vial was swiftly transferred into a glovebox to replace the punctured septum cap with a screw cap. After removal from the glovebox, the reaction was stirred at 500 rpm at room temperature under dark (covered by aluminum foil) for 1 h. The reaction vial was subsequently subjected to centrifugation at 4500 rpm for 5 min (to ensure that the MOF particles settle down on the bottom of reaction vial), and transferred into a glovebox. The C_6D_6 solution was carefully removed using a syringe and submitted to NMR for further analysis.

Another two identical reactions were set-up using the same procedure.

Since repeated sampling was not possible when ethylene was used as a substrate, different time points were collected from independent experiments.

Table S29. Kinetic data at room temperature analyzed by ^{1}H-NMR.

Time (h)	HS (%)	VS (%)	Conv. (%)	HS conc. (M)	VS conc. (M)	Conv. conc. (M)
0	0.0%	0.0%	0.0%	0	0	0
1	1.3%	0.0%	1.3%	0.002483	0	0.002483
20	7.0%	2.9%	9.9%	0.01337	0.005539	0.018909
50	17.1%	5.4%	22.5%	0.032661	0.010314	0.042975

22.1.2 Reaction at 50 °C

The 50 °C reactions were set-up using an analogous experimental procedure. Three reactions were run at 50 °C for 1 h, 2 h, and 3 h, respectively (Table S30).

Table S30. Kinetic data at 50 °C analyzed by ^{1}H-NMR.

Time (h)	HS (%)	VS (%)	Conv. (%)	HS conc. (M)	VS conc. (M)	Conv. conc. (M)
0	0.0%	0.0%	0	0	0	0
1	3.9%	1.3%	5.2%	0.007449	0.002483	0.009932
2	7.8%	2.6%	10.4%	0.014898	0.004966	0.019864
3	18.6%	6.5%	25.1%	0.035526	0.012415	0.047941

22.1.3 Reaction at 70 °C

The 70 °C reactions were set-up using an analogous experimental procedure. Three reactions were run at 70 °C for 0.5 h, 1 h, and 1.5 h, respectively (Table S31).

Table S31. Kinetic data at 70 °C analyzed by ^{1}H-NMR.

Time (h)	HS (%)	VS (%)	Conv. (%)	HS conc. (M)	VS conc. (M)	Conv. conc. (M)
0	0.0%	0.0%	0.0%	0	0	0
0.5	10.0%	4.2%	14.2%	0.0191	0.008022	0.027122
1	13.7%	6.4%	20.1%	0.026167	0.012224	0.038391
1.5	24.0%	11.0%	35.0%	0.04584	0.02101	0.06685

22.2 Data Analysis

The reaction constant was derived from Variable Time Normalisation Analysis (VTNA) method,[125-126] where $\mathrm{tT} = \sum([\mathrm{ethylene}]^x[\mathrm{silane}]^y[\mathrm{Rh}]^z)\,\Delta\mathrm{t}$ (x,y and z represented the reaction orders of ethylene, silane and Rh catalyst, respectively) , and thus the slope was equal to the reaction constant (*k*). For the calculation of tT by VTNA method, the following mathematical equation was used:

$$\mathrm{tT} = \sum([\mathrm{ethylene}]^x[\mathrm{silane}]^y[\mathrm{Rh}]^z)\Delta\mathrm{t}$$
$$= \sum_{1}^{i}\left(\frac{[ethylene]_i + [ethylene]_{i-1}}{2}\right)^x\left(\frac{[silane]_i + [silane]_{i-1}}{2}\right)^y [Rh]^z(t_i - t_{i-1})$$

The ethylene concentration ([ethylene]) kept constant through the reaction kinetic profile obtained above, because only around 0.7 equiv. dissolved in the reaction mixture.[61] When a fraction of the ethylene was converted, ethylene in the headspace of the vial can dissolve into the reaction mixture so that a constant concentration of 0.7 equiv. (0.1337 M) was maintained throughout the experiment. Unlike in our *in situ* NMR reaction reactions (Figure S8), no decrease in the reaction rate has ever observed once a conversion of ~55% was reached when thermal ethylene hydrosilylation was carried out in glass vials, so that ethylene dissolution appears not be kinetically inhibited. For details regarding the calculation of the rate constant (*k*), please refer to the tables and figures below. The rate constants obtained for the various temperature were be used to determine $\Delta H^‡$, $\Delta S^‡$, and $\Delta G^‡$ via the Eyring equation.[127]

Reaction orders of ~1 were determined for both silane and the Rh catalyst (Figure S80, Figure S81 and Figure S85). As indicated in Figure S88, the experimentally determined 1-pentene order was around 0.7, however the data quality was only modest, therefore a 1st order dependence of ethylene was assumed for simplicity. The olefin order had to be determined with a longer chain olefin due to experimental challenges associated with the use of ethylene. The use of a longer chain olefin could potentially change the olefin order, and quantum chemical calculations suggest an ethylene order of 2 (see discussion in manuscript). The experimental Gibbs energy of activation was therefore calculated assuming either an ethylene order of 1 ($\Delta G^‡$ = 19.94 ± 0.01 kcal·mol^{-1}, Table S32) or an ethylene order of 2 ($\Delta G^‡$ = 18.75± 0.01 kcal·mol^{-1},

Table **S33**) (see below for calculations details). Since the ethylene order could not be determined with certainty, the value given for the experimental Gibbs energy of activation refers to the average of the values determined for ethylene orders of one and two. The error stated for the Gibbs energy of activation refers to half of the span of the possible values of $\Delta G^‡$ (from the largest $\Delta G^‡$ value obtained for an ethylene

order of one and the lowest ΔG‡ value obtained for an order of two). Consequently, the manuscript lists the Gibbs energy of activation as ΔG‡ = 19.4 ± 0.6 kcal·mol^{-1}. We also note that challenges associated with the collection of kinetic data for a tri-phasic reaction in which the concentration of O_2 altered the reaction rate increased the error associated with our Eyring analysis so that the value obtained for ΔS‡ (Table S32 and Table S33) likely underestimates the real entropy of activation of the reaction.

Table S32. ΔH‡, ΔS‡, and ΔG‡ values for an ethylene order of one at 25 °C.

ΔG‡ (kcal·mol^{-1})	ΔH‡ (kcal·mol^{-1})	ΔS‡ (kcal·mol^{-1})
19.94 ± 0.01	17.60 ± 2.67	-0.0083 ± 0.0085

Table S33. ΔH‡, ΔS‡, and ΔG‡ values for an ethylene order of two at 25 °C.

ΔG‡ (kcal·mol^{-1})	ΔH‡ (kcal·mol^{-1})	ΔS‡ (kcal·mol^{-1})
18.75 ± 0.01	17.60 ± 2.67	-0.0043 ± 0.0085

For detailed calculations of rate constants, and the derivation of ΔH‡, ΔS‡, and ΔG‡ from Eyring analyses, please see the subsequent sections.

22.2.1 For an Ethylene Order of One

22.2.1.1 Rate Constant at 25°C

**rt (25 °C), *r* = *k*[ethylene]^1[silane]^1[Rh]^1**								
		t (h)	tT	[silane] (M)	[ethylene] (M)	[Conv] (M)	[Rh] (M)
**order in silane**	**1**	0	0.0000000	0.19100	0.1337	0.00000	0.0007
**order in ethylene**	**1**	1	0.0000178	0.18852	0.1337	0.00248	0.0007
**order in Rh**	**1**	20	0.0003384	0.17209	0.1337	0.01891	0.0007
		50	0.0007878	0.14803	0.1337	0.04298	0.0007

Table S34. Room temperature (25 °C) reaction kinetic profile via VTNA method. tT is equal to Σ[ethylene]^1 [silane]^1[Rh]^{1}Δt.

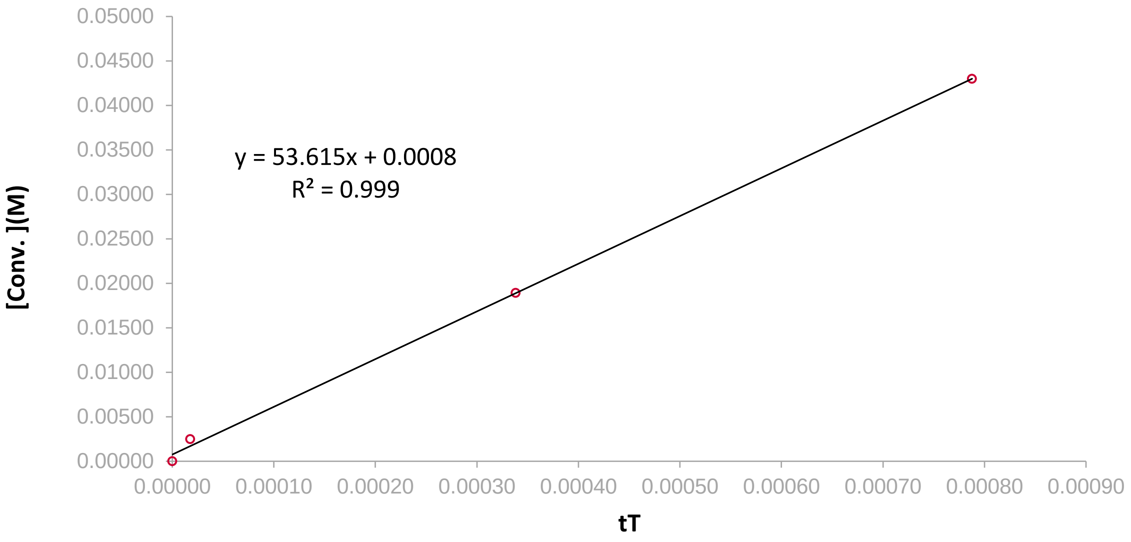

Figure S90. Plot of the change in silane concentration against of tT at 25 °C with the slope being the rate constant. tT is equal to $\sum$[ethylene]^1 [silane]^1[Rh]^{1}Δt. From the slope of the best linear fit, the rate constant k = 0.0149 ± 0.0003 M^{-2}/s.

22.2.1.2 Rate Constant at 50 °C

**50 °C, $r = k$[ethylene]^1[silane]^1[Rh]^1**								
**order in silane**	**1**	t (h)	tT	[silane] (M)	[ethylene] (M)	Conv. (M)	[Rh] (M)
**order in ethylene**	**1**	0	0.0000000	0.19100	0.1337	0.00000	0.0007
**order in Rh**	**1**	1	0.0000174	0.18107	0.1337	0.00993	0.0007
		2	0.0000339	0.17114	0.1337	0.01986	0.0007
		3	0.0000486	0.14306	0.1337	0.04794	0.0007

Table S35. 50 °C reaction kinetic profile via VTNA method. tT is equal to $\sum$[ethylene]^1 [silane]^1[Rh]^{1}Δt.

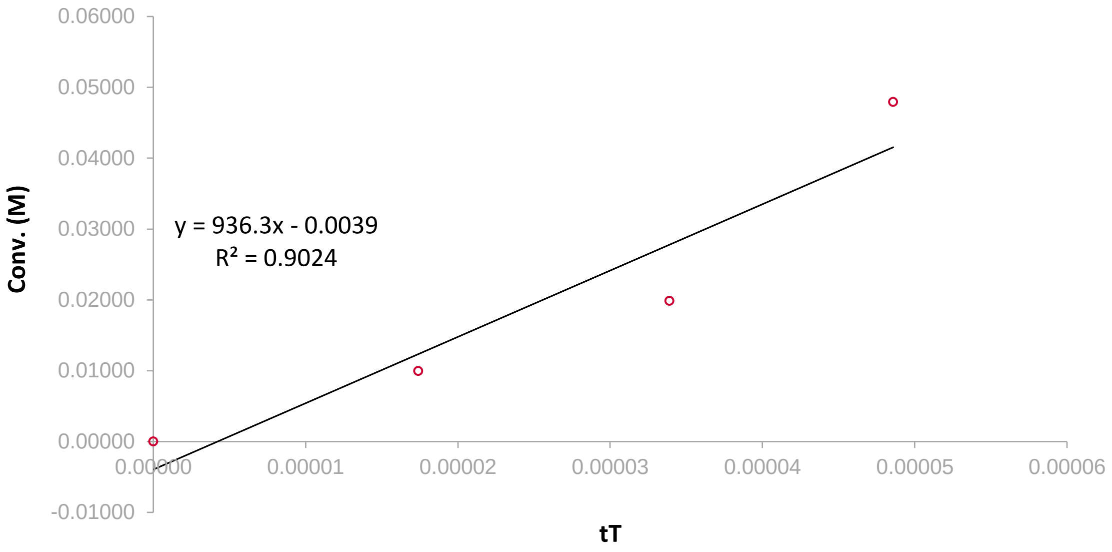

Figure S91. Plot of the change in silane concentration against of tT at 50 °C with the slope being the rate constant. tT is equal to $\sum$[ethylene]^1 [silane]^1[Rh]$^1\Delta$t. From the slope of the best linear fit, the rate constant k = 0.26 ± 0.06 M^{-2}/s

22.2.1.3 Rate Constant at 70 °C

**70 °C, *r* = *k*[ethylene]^1[silane]^1[Rh]^1**								
		t (h)	tT	[silane] (M)	[ethylene] (M)	[Conv] (M)	[Rh] (M)
**order in silane**	**1**	0.0	0.0000000	0.19100	0.1337	0.00000	0.0007
**order in ethylene**	**1**	0.5	0.0000083	0.16388	0.1337	0.02712	0.0007
**order in Rh**	**1**	1.0	0.0000157	0.15261	0.1337	0.03839	0.0007
		1.5	0.0000222	0.12415	0.1337	0.06685	0.0007

Table S36. 70 °C reaction kinetic profile via VTNA method. tT is equal to $\sum$[ethylene]^1 [silane]^1[Rh]$^1\Delta$t.

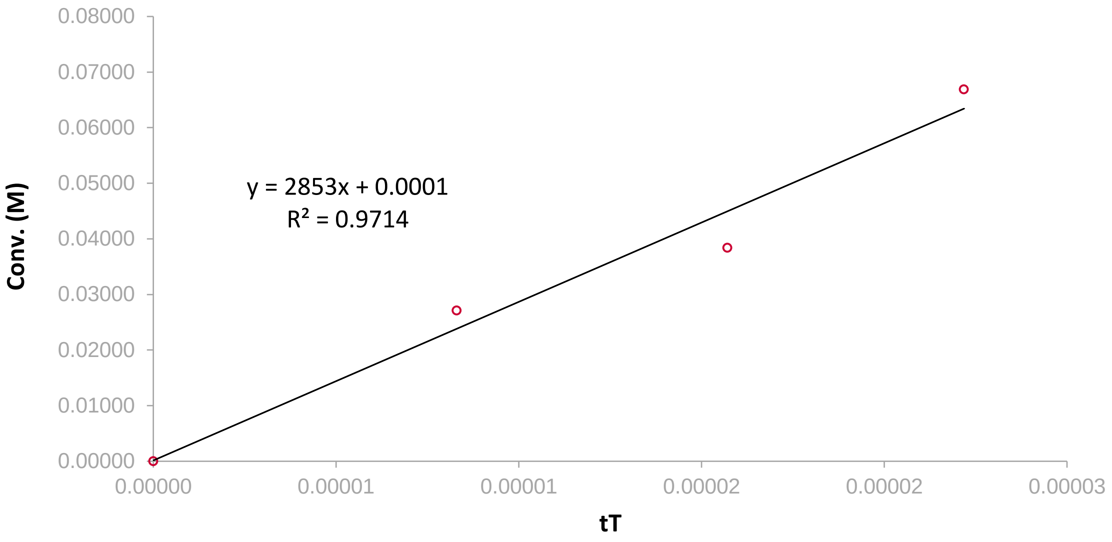

Figure S92. Plot of the change in silane concentration against of tT at 70 °C with the slope being the rate constant. tT is equal to $\sum$[ethylene]^1 [silane]^1[Rh]$^1\Delta$t. From the slope of the best linear fit, the rate constant k = 0.792 ± 0.096 M^{-2}/s.

22.2.1.4 Eyring Analysis for an Ethylene Order of One

Table S37. Summary of parameters in Eyring equation by assuming 1st order of ethylene.

T (K)	k (M^{-2}/s)	σ_k	1/T	ln(k/T)	$\sigma_{\ln(k/T)}$	Txln(k/T)	$\sigma_{T\text{xln}(k/T)}$
298.15	1.48932E-02	3.32831E-04	0.003354	-9.90445	2.23479E-02	-2953.01	6.66302E+00
323.15	2.60083E-01	6.04866E-02	0.003095	-7.12487	2.32566E-01	-2302.4	7.51538E+01
343.15	7.92494E-01	9.60916E-02	0.002914	-6.07074	1.21252E-01	-2083.17	4.16077E+01

The Eyring analysis was performed analogously to reported methods.[127] The enthalpy of activation, ΔH‡, was derived from the following equation:

$$\ln\frac{k}{T} = -\frac{\Delta H‡}{RT} + \frac{\Delta S‡}{R} + \ln\frac{k_B}{h}$$

where:

- R is the gas constant (8.314 J/mol/K)
- T is the temperature in Kelvin
- k is the rate constant

- h is the Planck constant (6.626×10^{-34} Js)
- k_B is the Boltzmann constant (1.381×10^{-23} J/K)

From a plot of ln(*k*/*T*) against 1/T (Figure S93), ΔH‡ = 17.6 ± 2.7 kcal·mol^{-1} was derived from the slope, where $\sigma_{\Delta H‡}$ was derived from the following equation: $\sigma_{\Delta H‡} = R\sigma_{slope}$

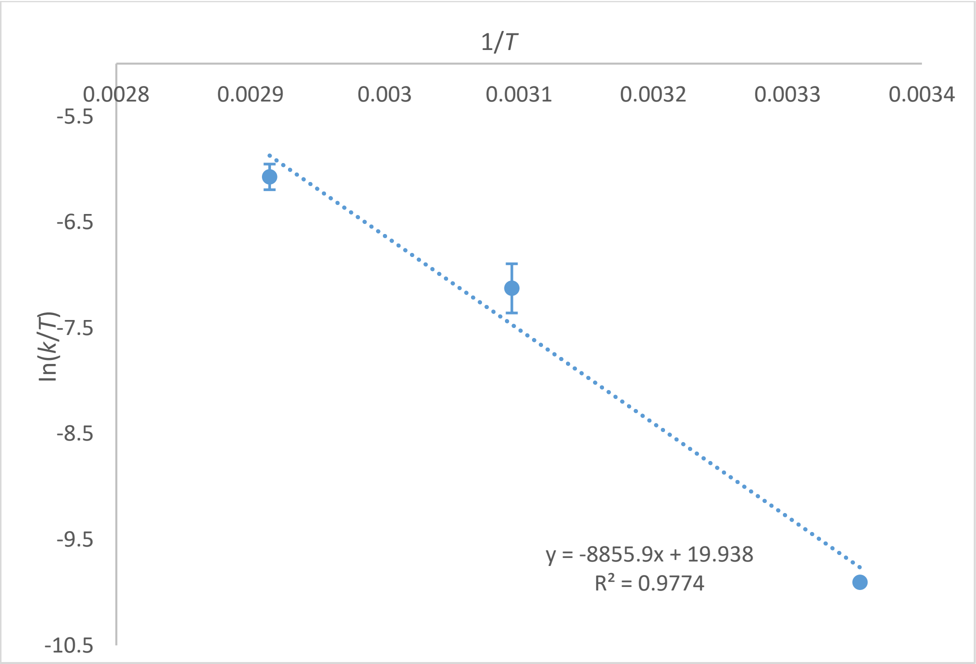

Figure S93. Eyring analysis to determine the enthalpy of activation (for an ethylene order of one).

The entropy of activation, ΔS‡, was derived from the following equation:

$$T \times \ln\frac{k}{T} = T \times \left(\frac{\Delta S‡}{R} + \ln\frac{k_B}{h}\right) - \frac{\Delta H‡}{R}$$

From a plot of *T* x ln(*k*/*T*) against *T*, ΔS‡ = –0.0083 ± 0.0085 kcal·mol^{-1}·K^{-1} was derived from the slope (Figure S94),where $\sigma_{\Delta S‡}$ was derived from the following equation: $\sigma_{\Delta S‡} = R\sigma_{slope}$.

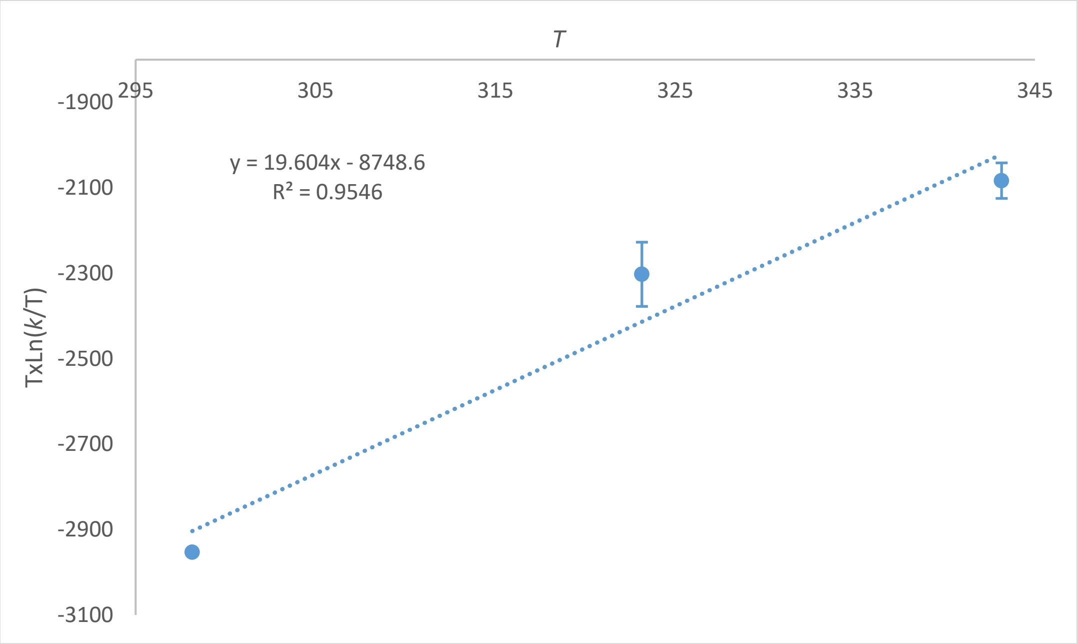

Figure S94. Eyring analysis to determine the entropy of activation (for an ethylene order of one).

The Gibbs energy of activation at 25 °C (under the assumption of an ethylene order of one) was obtained directly from the measured rate constant at 25 °C via:

$$k = \frac{k_B T}{h} e^{\left(\frac{-\Delta G‡}{RT}\right)}$$

Therefore, $\Delta G‡ = -RT \ln \frac{k}{\frac{k_B T}{h}}$, where $k_B/h \approx 2.0836612 \times 10^{10}\ K^{-1} \cdot s^{-1}$

$\sigma_{\Delta G‡}$ was derived from the following equation: $\sigma_{\Delta G‡} = RT \cdot \left(\frac{\sigma_k}{k}\right)$

At 25 °C (T = 298.15 K), k = 0.0149 ± 0.0003 $M^{-2} \cdot s^{-1}$

Which results in $\Delta G^‡$ (25 °C) = **19.94 ± 0.01 kcal·mol^{-1}**

22.2.2 For an Ethylene Order of Two

22.2.2.1 Rate constant at 25 °C

Table S38. Room temperature (25 °C) reaction kinetic profile via VTNA method. tT is equal to Σ[ethylene]^2 [silane]^1[Rh]$^1\Delta$t.

		rt (25 °C), $r = k$[ethylene]^2[silane]^1[Rh]^1					
		t (h)	tT	[silane] (M)	[ethylene] (M)	[Conv] (M)	[Rh] (M)
**order in silane**	**1**	0	0.0000000	0.19100	0.1337	0.00000	0.0007
**order in ethylene**	**2**	1	0.0000024	0.18852	0.1337	0.00248	0.0007
**order in Rh**	**1**	20	0.0000452	0.17209	0.1337	0.01891	0.0007
		50	0.0001053	0.14803	0.1337	0.04298	0.0007

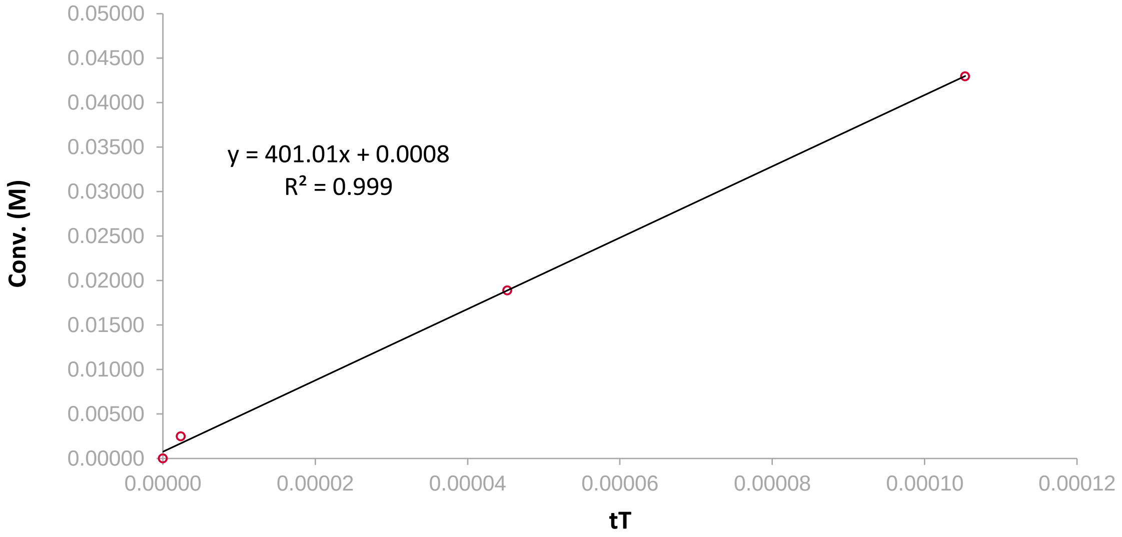

Figure S95. Plot of the change in silane concentration against of tT at 25 °C with the slope being the rate constant. tT is equal to Σ[ethylene]^2 [silane]^1[Rh]$^1\Delta$t. From the slope of the best linear fit, the rate constant k = 0.111 ± 0.002 M^{-3}/s.

22.2.2.2 *Rate constant at 50 °C*

Table S39. 50 °C reaction kinetic profile via VTNA method. tT is equal to $\sum$[ethylene]^2 [silane]^1[Rh]$^1\Delta$t.

**50 °C, $r = k$[ethylene]^2[silane]^1[Rh]^1**								
		t (h)	tT	[silane] (M)	[ethylene] (M)	Conv. (M)	[Rh] (M)
**order in silane**	**1**						
**order in ethylene**	**2**	0	0.00000000	0.19100	0.1337	0.00000	0.0007
**order in Rh**	**1**	1	0.00000233	0.18107	0.1337	0.00993	0.0007
		2	0.00000453	0.17114	0.1337	0.01986	0.0007
		3	0.00000650	0.14306	0.1337	0.04794	0.0007

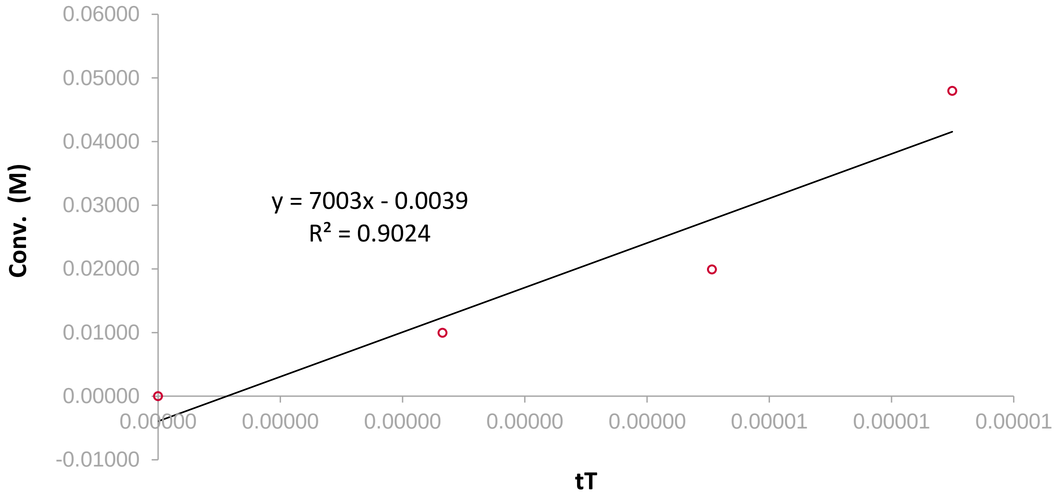

Figure S96. Plot of the change in silane concentration against of tT at 50 °C with the slope being the rate constant. tT is equal to $\sum$[ethylene]^2[silane]^1[Rh]$^1\Delta$t. From the slope of the best linear fit, the rate constant k = 1.95 ± 0.45 M^{-3}/s.

22.2.2.3 Rate constant at 70 °C

Table S40. 70 °C reaction kinetic profile via VTNA method. tT is equal to $\sum$[ethylene]^2[silane]^1[Rh]^{1}Δt.

**70 °C, *r* = *k*[ethylene]^2[silane]^1[Rh]^1**							
		t (h)	tT	[silane] (M)	[ethylene] (M)	[Conv] (M)	[Rh] (M)
**order in silane**	**1**	0.0	0.00000000	0.19100	0.1337	0.00000	0.0007
**order in ethylene**	**2**	0.5	0.00000111	0.16388	0.1337	0.02712	0.0007
**order in Rh**	**1**	1.0	0.00000210	0.15261	0.1337	0.03839	0.0007
		1.5	0.00000297	0.12415	0.1337	0.06685	0.0007

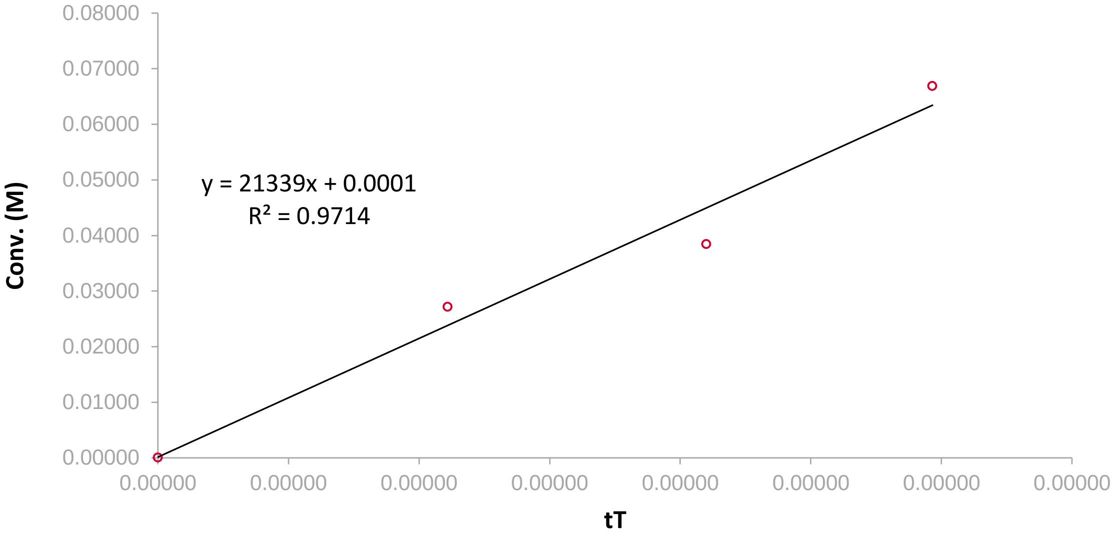

Figure S97. Plot of the change in silane concentration against of tT at 70 °C with the slope being the rate constant. tT is equal to $\sum$[ethylene]^2 [silane]^1[Rh]^{1}Δt. From the slope of the best linear fit, the rate constant k = 5.92 ± 0.72 M^{-3}/s.

22.2.2.4 Eyring Analysis for an Ethylene Order of Two

Table S41. Summary of values used in Eyring analysis under the assumption of an ethylene order of two.

T (K)	k (M^{-3}/s)	σ_k	1/T	ln(k/T)	$\sigma_{\ln(k/T)}$	Txln(k/T)	$\sigma_{T\text{xln}(k/T)}$
298.15	1.114E-01	2.489E-03	0.00335	-7.89229	2.235E-02	-2353.09	6.663E+00
323.15	1.945E+00	4.524E-01	0.00309	-5.11271	2.326E-01	-1652.17	7.515E+01
343.15	5.927E+00	7.187E-01	0.00291	-4.05858	1.213E-01	-1392.7	4.161E+01

The Eyring analysis was performed following the literature report method.[127]

$$\ln\frac{k}{T} = -\frac{\Delta H‡}{RT} + \frac{\Delta S‡}{R} + \ln\frac{k_B}{h}$$

$\Delta H^‡$ = 17.6 ± 2.7 kcal·mol^{-1} was derived from the slope of a plot of ln(k/T) against 1/T (Figure S98), and $\sigma_{\Delta H‡}$ was obtained from the following equation: $\sigma_{\Delta H‡} = R\sigma_{slope}$.

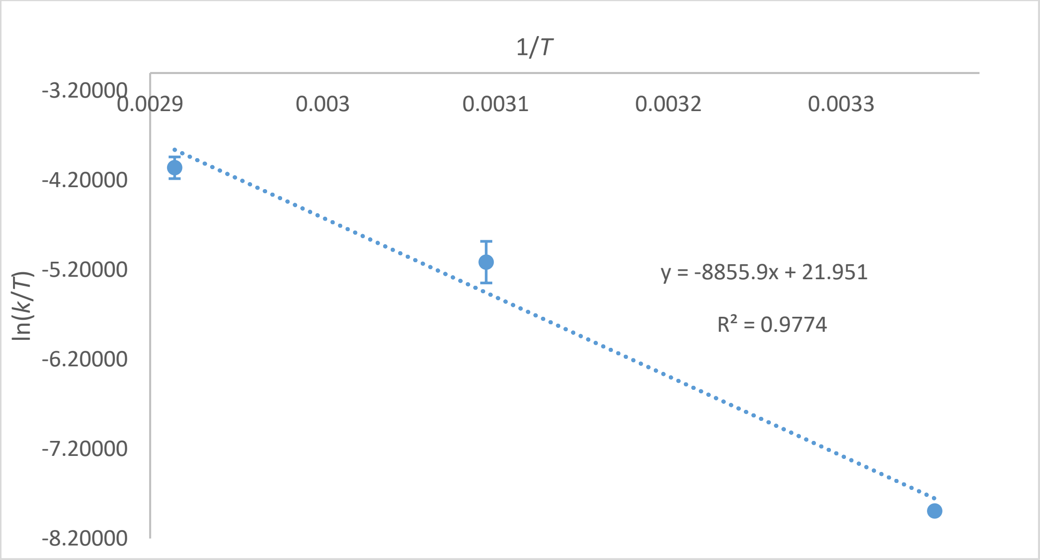

Figure S98. Eyring analysis to determine the entropy of activation (ethylene order of two).

$\Delta S^‡$ was derived from the following equation:

$$T \times \ln\frac{k}{T} = T \times \left(\frac{\Delta S‡}{R} + \ln\frac{k_B}{h}\right) - \frac{\Delta H‡}{R}$$

$\Delta S^‡$ = -0.0043 ± 0.0085 kcal·mol^{-1}·K^{-1} was derived from the slope of a plot of T ln(k/T) against T (Figure S99),[127] and $\sigma_{\Delta S‡}$ was obtained from the following equation: $\sigma_{\Delta S‡} = R\sigma_{slope}$.

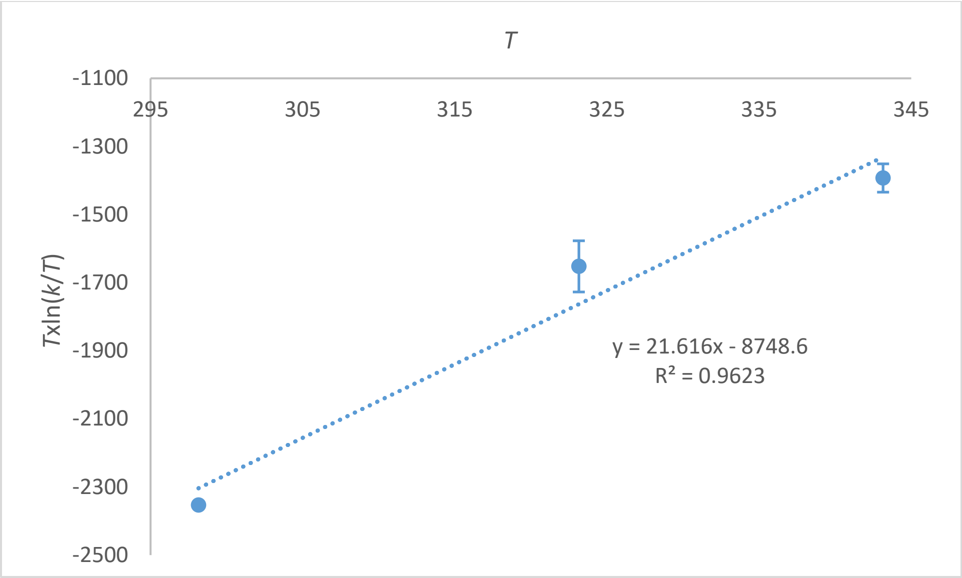

Figure S99. Eyring analysis to determine the entropy of activation (ethylene order of two).

The Gibbs energy of activation at 25 °C, $\Delta G_{298K}^{\ddagger}$; was obtained directly from the measured rate constant (under the assumption of an ethylene order of two) at 25 °C via:

$$k = \frac{k_B T}{h} e^{\left(\frac{-\Delta G^{\ddagger}}{RT}\right)}$$

Therefore, $\Delta G^{\ddagger} = -RT \ln \frac{k}{\frac{k_B T}{h}}$, where $k_B/h \approx 2.0836612 \times 10^{10}\ K^{-1} \cdot s^{-1}$

And $\sigma_{\Delta G^{\ddagger}}$ was derived from the following equation: $\sigma_{\Delta G^{\ddagger}} = RT \cdot \left(\frac{\sigma_k}{k}\right)$

At 25 °C (T = 298.15 K), k = 0.111 ± 0.002 $M^{-3} \cdot s^{-1}$

Which results in **ΔG‡ (25 °C) = 18.75 ± 0.01 kcal·mol^{-1}**

23 Solvent Effect

23.1 Ethylene Solubility in Different Solvents

Quantitative ^{1}H NMR was used to estimate the amounts of ethylene dissolved in C_6D_6, HFIP, C_6H_5F and n-hexane. Because ethylene and propylene are small symmetric molecules, the relaxation time of their protons was expected to be long. Therefore, a T1 analysis was carried out, and NMR spectra used for quantification were acquired with a d1 delay that exceeded 7 x longest T1. For details, please see below.

Procedure: In an argon-filled glovebox, related solvent (1.0 mL) and diethylmethylsilane (27.7 µl , 0.191 mmol, 1.00 equiv) were added to into a J-Young NMR tube. Then the J-Young NMR tube was placed into a larger glass container sealed with a rubber septum and transferred them out of the glovebox (same set up in Figure S7). Ethylene was then bubbled into solution with a long needle for 1 minute in order to saturate the solvent with ethylene. To prevent oxygen leaking into the reaction mixture as ethylene was introduced, another outlet that released excess ethylene from the dosing process was connected to an inert gas manifold which was connected to an oil bubbler. Following the addition of ethylene for 1 min, a tiny counter flow of ethylene was maintained. Then the set up was quickly transferred into the glovebox. The J-Young NMR tube was rapidly sealed, and subjected to quantitative ^{1}H NMR analysis using a 600 MHz NMR spectrometer.

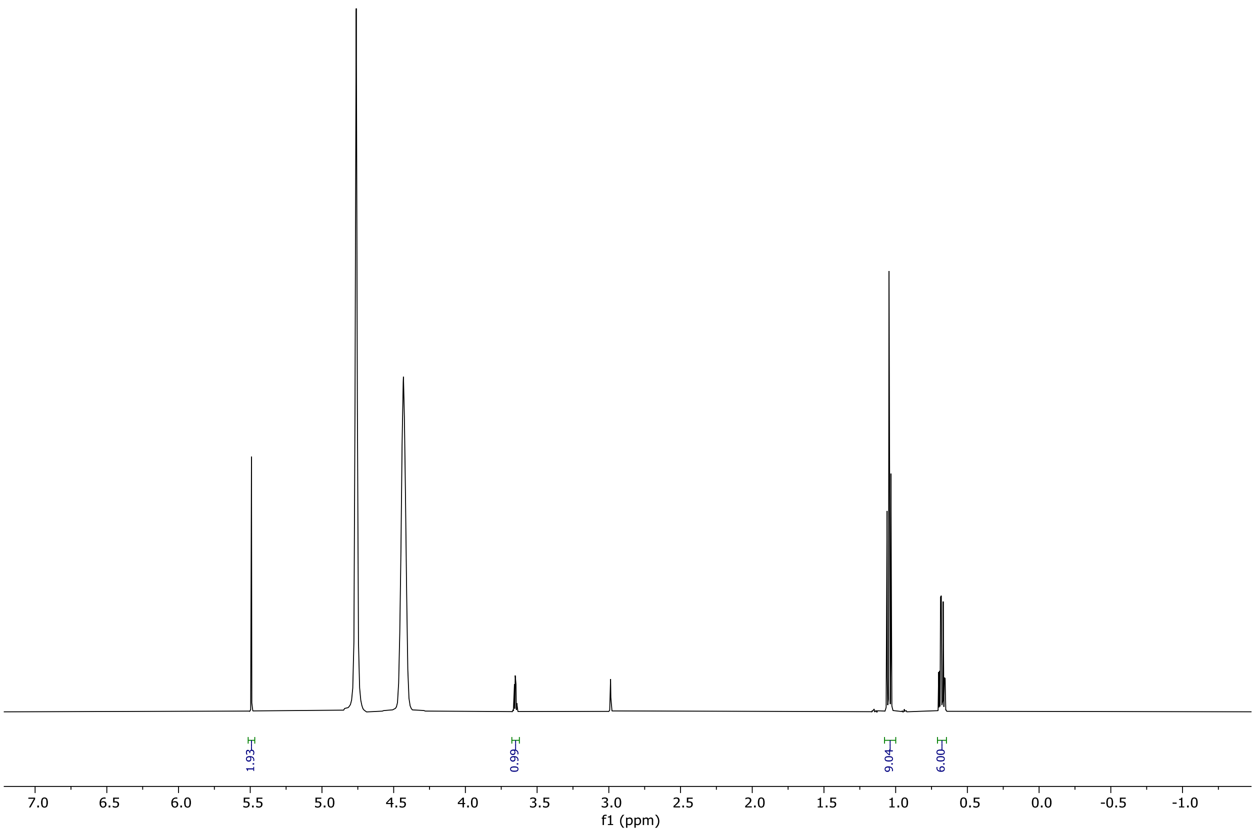

Figure S100. Quantitative ^{1}H NMR analysis to determine the ethylene amount dissolved in HFIP. Et_2MeSiH was used as an internal standard for quantification with acquisition parameter T1 is ~14.4 s, D1 is ~112 s.

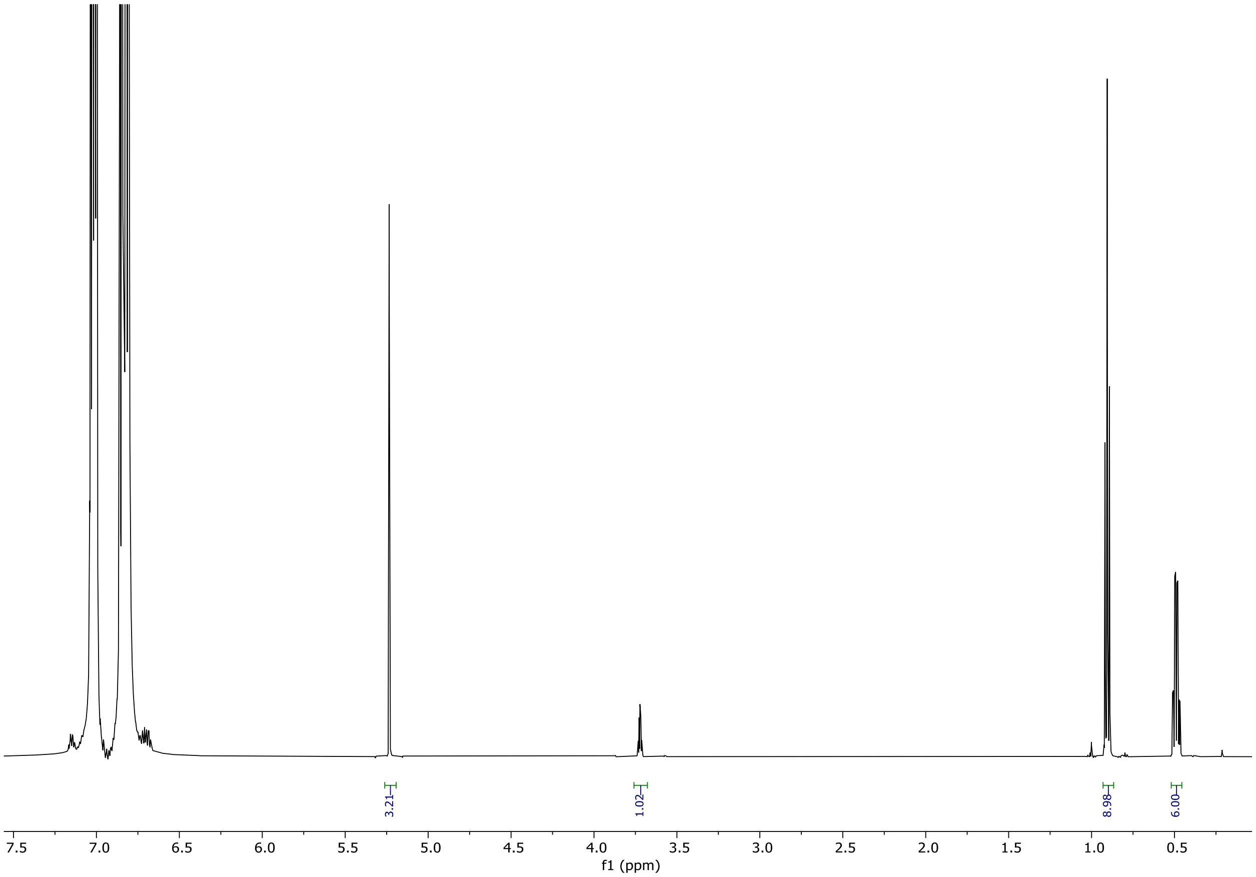

Figure S101. Quantitative ^{1}H NMR analysis to determine the ethylene amount dissolved in C_6H_5F. Et_2MeSiH was used as an internal standard for quantification with acquisition parameter T1 is ~24.7 s, D1 is ~210 s.

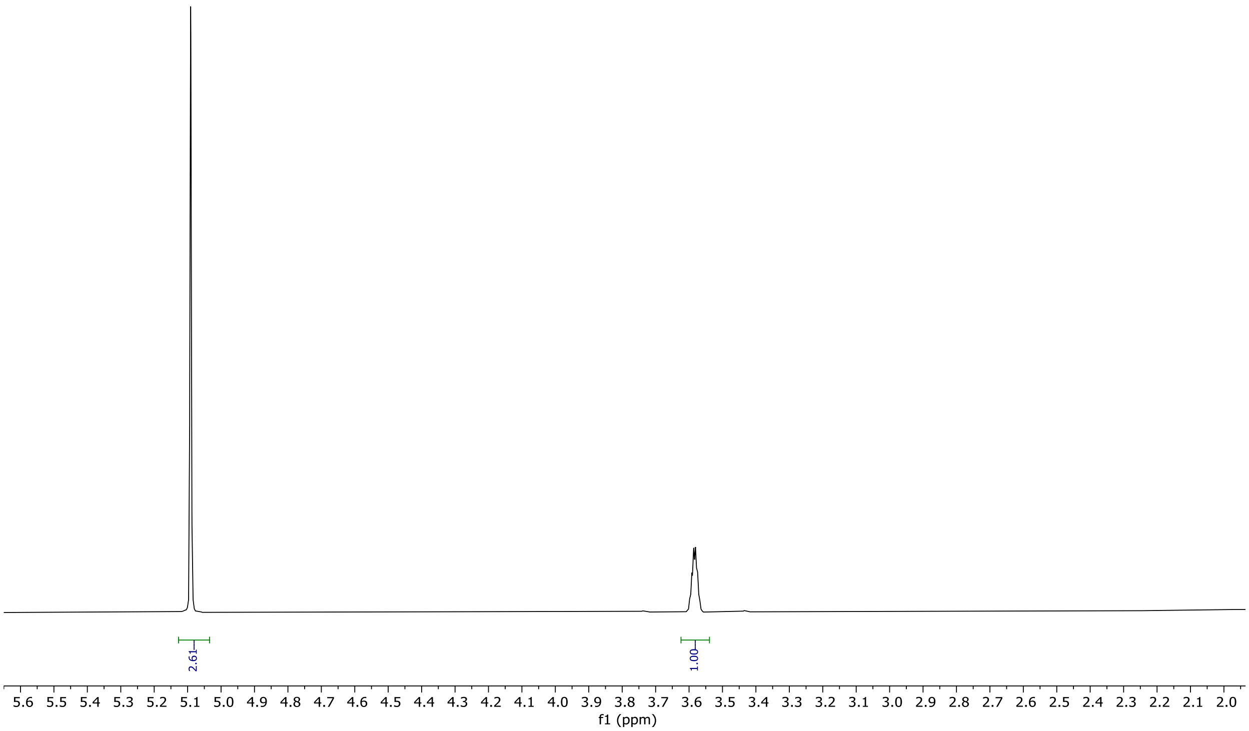

Figure S102. Quantitative ^{1}H NMR analysis to determine the ethylene amount dissolved in n-Hexane. Et_2MeSiH was used as an internal standard for quantification with acquisition parameter D1 is ~500 s.

In the 9 mL reaction glass vial containing 1 mL solvent, the 9 mL head space volume was additionally filled with ethylene during the ethylene bubbling process. The ideal gas equation PV=nRT (P = 1 atm, T = 298 K) was used to calculate the amount of ethylene in gas phase, which shows that ~0.32 mmol ethylene was present in the gas phase (around 1.7 equiv). Based on ^{1}H NMR analysis (Figure S100, Figure S101 and Figure S102), it was thus estimated that ethylene amount dissolved in solution and a total amount of ethylene were listed below (Table S42). (note: the data in C_6D_6 was adapted from our previously report)[61]

Table S42. Total amount of ethylene in reaction vial when different solvent were employed.

Solvent	Ethylene dissolved in solution	Ethylene in headspace	In total
C_6D_6	0.7 equiv	1.7 equiv	2.4 equiv
HFIP	0.5 equiv	1.7 equiv	2.2 equiv
C_6H_5F	0.8 equiv	1.7 equiv	2.5 equiv
n-hexane	0.6 equiv	1.7 equiv	2.3 equiv

23.2 Solvent Effect with Individually Prepared Rh(II)-**3**

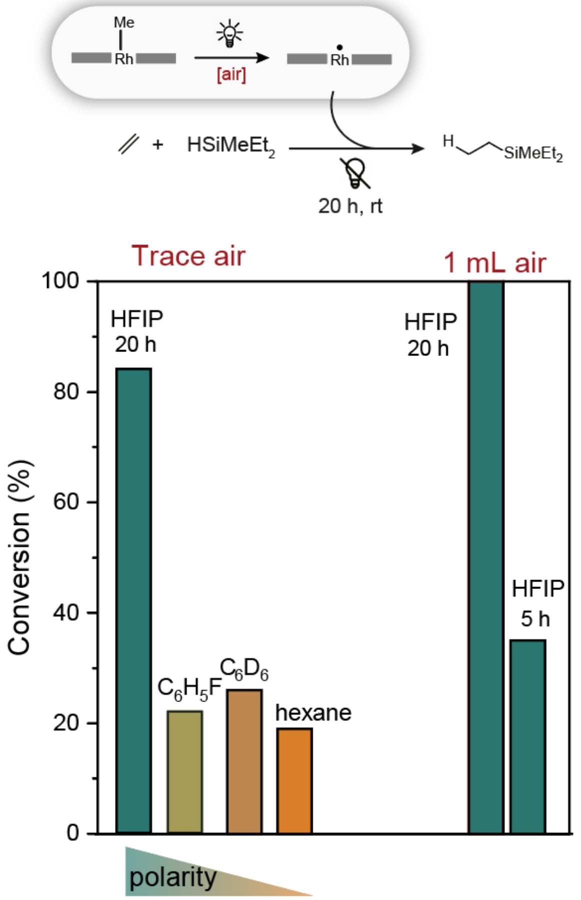

Figure S103. Solvent effect on the yields obtained in thermal ethylene hydrosilylation reactions carried out according to the general experimental procedure. Unless otherwise indicated, reactions were stopped after 20 h, and the conversion was determined by ^{1}H-NMR analysis.

Individual batches of the active Rh(II)-**3** catalyst were prepared according to the general experimental procedure and tested in thermal ethylene hydrosilylation in the presence of the solvent indicated in Figure S98, which is also shown in the main manuscript. To rule out that the results shown were affected by variations in the oxygen content of reactions, we performed additional experiments (see below).

23.3 Solvent Effect with Rh(II)-3 from a Single Batch

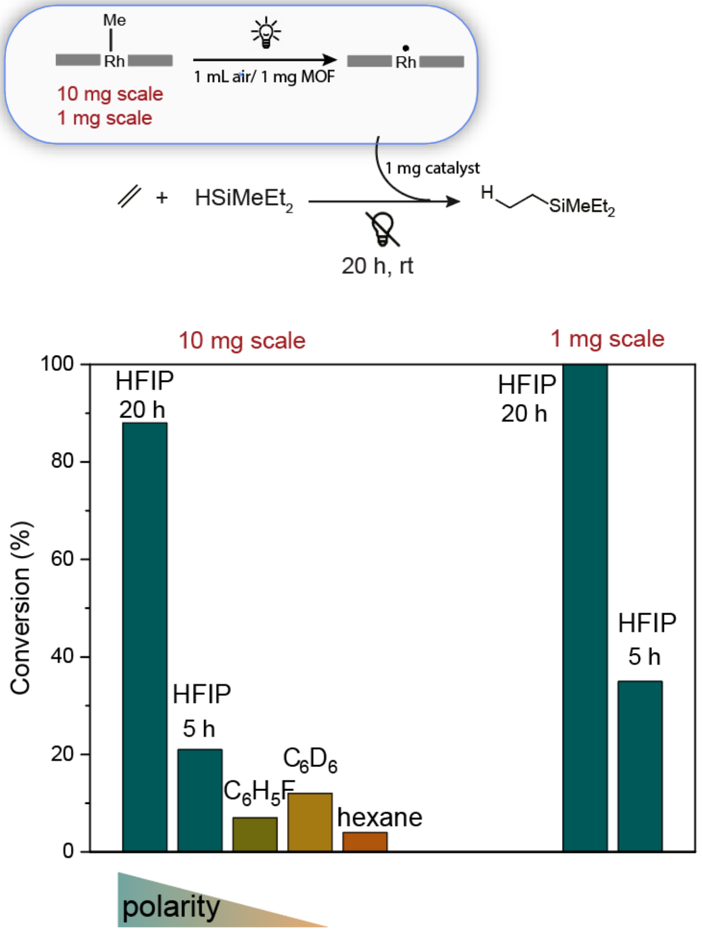

Figure S104. Solvent effect on the yields obtained in thermal ethylene hydrosilylation reactions carried out according to the general experimental procedure. Unless otherwise indicated, reactions were stopped after 20 h, and the conversion was determined by ^{1}H-NMR analysis.

The active Rh(II) catalyst used for experiments in Figure S99 was prepared according to the general experimental procedure for the photolysis of Rh(III)Me-**3** was carried out on a sufficiently large scale to ensure that all the ethylene hydrosilylation reactions shown could be carried out with the same catalyst batch. Specifically, 10 mg Rh(III)Me-**3** (7.1 µmol) were used in conjunction with 10 mL rigorously deoxygenated C_6D_6 to which 10 mL air were deliberately added.

It should be noted that a slight decrease in reaction yields was always observed with Rh(II)-**3** catalyst that was prepared on a larger scale. We attribute the reproducible differences based on batch size to the difficulty of keeping the MOF powder suspended in the reaction medium during irradiation. We had previously reported that ensuring good dispersion during irradiation is vital for obtaining optimal yields.[61] While the absolute performance of catalyst used in Figure S104 was thus reduced, the larger batch size used for the preparation of Rh(II)-**3** ensured that reactions with all solvents could be carried out with a

single batch of Rh(II)-**3**. We could thus exclude that the presence of varying amounts of oxygen during catalyst preparation affected the results of the solvent comparison shown in Figure S99.

24 List of DFT-Optimized Atomic Coordinates

Unless otherwise noted, all porphyrin complexes were truncated by saturating the four carboxylate groups by methyl groups. Structures that are labeled “dimer models” have a nearby porphyrin rhodium unit included.

Rh(II)-**3**

Rh	6.04665929872951	8.50880095768840	14.95915350138356
N	6.55965227154998	8.31669998188366	12.98955465347558
N	7.93935909592648	8.00435541977085	15.50096713298560
N	5.54578348241886	8.73904002356881	16.92781548530795
N	4.15429225812048	8.99485148133245	14.41986714735208
C	5.74904627963724	8.56864471998696	11.89216840249955
C	6.52668118815943	8.46114250916042	10.67053388346950
H	6.14395627485697	8.64566681605086	9.66546815678137
C	7.80050357024861	8.10860354110551	11.03626394697253
H	8.66559440639060	7.95255118445749	10.38971563876074
C	7.81312213808299	8.00470245851480	12.48467502730505
C	8.94455750777110	7.65566162447129	13.24348679511397
C	8.97995617026952	7.63883298777239	14.65142136064140
C	10.12479525045283	7.23050125696586	15.43142474033110
H	11.06914722788080	6.87128779334431	15.01910018617803
C	9.78317750548913	7.37258250398894	16.75819503236941
H	10.39603372595625	7.15080257430628	17.63335803717002
C	8.42749561232038	7.86935251145717	16.79779031967237
C	7.72511187800605	8.16609475850454	17.98222853988767
C	6.38807058891134	8.60072756480881	18.02088455222412
C	5.68976914823008	8.99056216742402	19.23303387083158
H	6.13156103253935	9.01327005434952	20.23054847032742
C	4.41507998711327	9.33961182953964	18.86694036223402
H	3.60830690709727	9.70031533794497	19.50698997502263
C	4.32252729267425	9.16002382970807	17.42902457114428

C	3.15302926099455	9.36754857480295	16.67612052346190
C	3.08355025334681	9.24524162629706	15.27417737190258
C	1.86729947413601	9.36881281996453	14.50542479679567
H	0.87181709161629	9.51922978732106	14.92649876862689
C	2.20990666683214	9.22991221650862	13.17833868169472
H	1.54670870723469	9.24498985243022	12.31181108911090
C	3.63766345904171	9.01944445457289	13.12706191949935
C	4.37525665292994	8.86583889534626	11.93644238483787
C	10.18933415879474	7.28215216757060	12.50817340757689
C	11.35122546853687	8.08159444043840	12.60424301982927
H	11.32922283760022	8.98906593008573	13.21965244202730
C	12.51438883029435	7.73141866740681	11.90947487370934
H	13.41793751670184	8.34915759759720	11.97135681528392
C	12.54243252986693	6.57212958084776	11.10630336051216
C	11.38692848792998	5.76813993194956	11.00652098058411
H	11.40952999241302	4.86497117593497	10.38771332550839
C	10.22302352454185	6.12309855077957	11.70011111422717
H	9.32731966874221	5.49428682648438	11.62915435741652
C	8.44536963054813	8.01195913016601	19.28118556351912
C	9.55583937763097	8.82935137978055	19.59149215925669
H	9.88166217665246	9.58663044435518	18.86824221170778
C	10.22606077789288	8.69150481113812	20.81365658336381
H	11.08036409361300	9.33352003727782	21.05263080228618
C	9.79759230062347	7.72670775534572	21.75023399740913
C	8.69147269201408	6.90602958237380	21.44527761905531
H	8.37517884510118	6.15760119445147	22.18114669328336
C	8.02172372204587	7.04836946353296	20.22504033503778
H	7.16659124575130	6.40476580017621	19.98630897648600
C	1.90373366274376	9.73394928335096	17.40659527678887
C	1.28238158692105	10.98479072799286	17.18661809816831
H	1.73459611853753	11.69091447553492	16.47991286343182
C	0.11256160826343	11.32814015955850	17.87335676542043
H	-0.37238448780442	12.29859003194948	17.71599328444735
C	-0.46383379517139	10.42666963831635	18.79255796755807

C	0.14863597956308	9.17505703993967	19.01563692728548
H	-0.30265207839860	8.47235896381666	19.72384818377062
C	1.32125751259225	8.83543681262072	18.32935639256964
H	1.79181103782158	7.85896679807449	18.49547831302837
C	3.64986226676438	9.02445670423641	10.64145232600126
C	3.07182523574354	10.26544983884035	10.28946835598192
H	3.16892052619141	11.11489714869810	10.97624436848142
C	2.39716609959356	10.42084228657898	9.07191058209881
H	1.95948246621561	11.38714851007033	8.80025026420916
C	2.28428264038803	9.33149751037917	8.18188993961669
C	2.85492055158266	8.08906366796235	8.52919035872992
H	2.75223191546121	7.25094204891136	7.83010542355275
C	3.53126036627328	7.93764848426929	9.74476512465738
H	3.96630938063307	6.96841853779898	10.01652285808728
C	1.57969277287678	9.43713079992787	6.86906363533953
O	1.46540566315417	8.52111315637622	6.06696033768843
O	1.07350360991256	10.67599217997889	6.65734263126510
C	10.47113041543659	7.53382922413688	23.06936032781571
O	10.13098284586534	6.71214556731979	23.90884176908340
O	11.51510176038309	8.38048648821315	23.24092329614345
C	13.81158007929819	6.24670509138704	10.38915024068256
O	14.83373794835669	6.91544561784832	10.44886231487832
O	13.70874295842291	5.11333807533082	9.65356127034741
C	-1.71283439898976	10.84558269660138	19.49650240081220
O	-2.27726765664113	11.91753721868925	19.32988864396030
O	-2.16102070548731	9.89651603930721	20.35342002032716
C	-3.36194938473835	10.22371695147032	21.07391208813183
H	-4.19997392238567	10.39676596378505	20.37631865430182
H	-3.57254673686196	9.35921280429590	21.71988402804585
H	-3.21549464610839	11.13336941819017	21.68194163455300
C	0.38331600825768	10.86281576928704	5.40957746357359
H	1.05981566534531	10.67329469128070	4.55792721356804
H	-0.47895923945361	10.17750135030205	5.33430020943484
H	0.04392747676553	11.90871548582687	5.40499502870530

C	14.89113366324359	4.73100544574860	8.92942811769249
H	15.17343843245300	5.51419282274025	8.20425061640678
H	15.73644206206005	4.56901366421517	9.62086692164081
H	14.63745626212134	3.79748441960131	8.40656183974362
C	12.21644913084886	8.25957411449784	24.49069153184070
H	13.01792325065781	9.01186886551861	24.46211924247468
H	11.53685418020397	8.45218757192785	25.33911862255884
H	12.64194786882707	7.24693375425251	24.60232961164609

Rh(II)-**3**-$(C_6H_6)_2$

Rh	6.44213312865572	9.48397532422599	14.93461320800847
N	6.98466757320474	9.33151916332409	12.96802255135636
N	8.33437611789269	8.94525751437956	15.49837379085925
N	5.91803996006402	9.69273724088848	16.89822765279256
N	4.53568947084832	9.95791614595176	14.37156446602149
C	6.17270203513255	9.54816385817443	11.86872397849100
C	6.95491416691624	9.42418061850599	10.65127530131424
H	6.57283176146143	9.58175744280972	9.64120875195462
C	8.23497523590649	9.10061169740634	11.02833438360752
H	9.10318502318517	8.94132579504596	10.38664620090733
C	8.24207443226524	9.02111653053009	12.47886140308478
C	9.36522973805956	8.66613528934004	13.25156762288138
C	9.38484812673924	8.61582663511401	14.65923535072218
C	10.53467581697477	8.21325246735806	15.45039713871169
H	11.48664729502399	7.86814548800719	15.04335639377637
C	10.17571801603449	8.33735180019656	16.77000112523232
H	10.77689132808286	8.11262628827092	17.65264852577338
C	8.80456431075934	8.81595338425634	16.79351085846465
C	8.08630682738342	9.11358521620172	17.96721614339536
C	6.74729832243825	9.54574939515459	17.99562088301347
C	6.02914639899013	9.91094595228975	19.20418890082729
H	6.45564728374656	9.92369428119397	20.20848418803771
C	4.75275571373251	10.24490927924110	18.82492590515132
H	3.93184272541067	10.58443527292056	19.45865988207491

C	4.68440405363312	10.08661880980988	17.38285962372903
C	3.52588742138002	10.30604192258181	16.61519333267346
C	3.46848442442635	10.21282179794220	15.21266581278313
C	2.25522291247952	10.37121493889881	14.43016312194761
H	1.26008047593770	10.54366132805357	14.84345166526540
C	2.60990380561212	10.23866599694771	13.11059551411748
H	1.96194563184781	10.28277739016484	12.23367695758294
C	4.04229786480638	10.00055609807975	13.07987458314744
C	4.79458942513558	9.83414840780132	11.90254651120844
C	10.62281745553922	8.31464846458404	12.52681749328470
C	11.77242912421443	9.12894071734132	12.64226730688424
H	11.72989397413096	10.03025610487066	13.26554065951673
C	12.94925186493455	8.79978482882659	11.96031250027831
H	13.84362726291847	9.42897627965771	12.03783910215434
C	13.00359758401329	7.64647097453710	11.14975673241640
C	11.86071702989614	6.82703167368337	11.03061423957944
H	11.90410352582967	5.92839788863666	10.40637984342804
C	10.68380029031560	7.16122641700231	11.71239026558374
H	9.79774297507140	6.52071450601493	11.62691551535234
C	8.80017036249697	8.99183888559093	19.27234961633652
C	9.88509356988392	9.84566832222937	19.57537100415098
H	10.19533613023183	10.59523288465754	18.83774604422789
C	10.54951228817528	9.75107687785590	20.80461032452424
H	11.38417761229452	10.42051377687648	21.03748718100558
C	10.14022694290803	8.79323727014624	21.75705934626844
C	9.06002992809602	7.93584003346434	21.45974859263508
H	8.75931145076882	7.19349845540614	22.20817688589268
C	8.39675133305478	8.03534818819615	20.23170538699417
H	7.56074507768744	7.36507568698897	19.99890804085771
C	2.26995226796253	10.66093409455606	17.33846290545281
C	1.66753805626726	11.92678914456833	17.15596790594177
H	2.13923938686676	12.65134155466849	16.48155894109643
C	0.49036979440848	12.25966944700347	17.83515120612400
H	0.01964846799582	13.24125932715902	17.70614219877327

C	-0.11340964224244	11.33178051901656	18.70973061651312
C	0.48001199924073	10.06487707456643	18.89590012519000
H	0.00780011478828	9.34227332758197	19.56965947815145
C	1.66045922945944	9.73691931843068	18.21733340343663
H	2.11815012557572	8.75014111000747	18.35564774413183
C	4.07578786819329	9.93629625711476	10.59846704779703
C	3.50964347668765	11.16000846232487	10.17599585737936
H	3.61215807994980	12.04471391679423	10.81586517843688
C	2.83766436060608	11.25278042867741	8.95046963032322
H	2.40878347524897	12.20587635236379	8.62374597814158
C	2.71599512888043	10.11597351733188	8.12297930036808
C	3.27447589444564	8.88978280359655	8.54116946735180
H	3.16499235992838	8.01476824977539	7.88994220798862
C	3.94815013318568	8.80191382412964	9.76434762795213
H	4.37649510927726	7.84702655562447	10.09150096174769
C	2.01523257966172	10.15399825733561	6.80498074678033
O	1.88927463267232	9.19444621486397	6.05719879816027
O	1.52650015203061	11.38531156718129	6.51907615030700
C	10.80789261788700	8.64521419366150	23.08440305647313
O	10.48270794369327	7.83286595125289	23.93890553186907
O	11.82971799933892	9.52090778192419	23.24562818522060
C	14.28566414559781	7.34337605362369	10.44677191357136
O	15.29785190423420	8.02562869004527	10.52286652129610
O	14.20828935821874	6.21292888059267	9.70313183211302
C	-1.36912329208284	11.74003988628324	19.40681327675779
O	-1.91512039293504	12.82639019109020	19.27487621206650
O	-1.84807453002880	10.76335119475887	20.21527223871411
C	-3.05775852082197	11.07960557888513	20.92559366676437
H	-3.87822089372011	11.29880599702588	20.22018823784563
H	-3.29651675379421	10.19116414798454	21.52781707512609
H	-2.90870546496065	11.95836668835452	21.57693425024384
C	0.84204268997054	11.50774380222851	5.26047477571600
H	1.51666548782900	11.25537001297472	4.42379581540417
H	-0.03189096958503	10.83410093247592	5.22404258198633

H	0.52069588435617	12.55704670638377	5.19144684833179
C	15.40494534260973	5.85154445369781	8.99223456829552
H	15.68827467204011	6.64453007185940	8.27822390714507
H	16.24251682634311	5.69298705308929	9.69372426558432
H	15.16990810100788	4.91995356659314	8.45749228635350
C	12.52462418761068	9.44239811799391	24.50211073353380
H	13.31039829619185	10.21050930667904	24.46210317323132
H	11.83543775922499	9.63940898386228	25.34179645942374
H	12.97028956671525	8.44169202307557	24.63917369490758
C	3.36368826379890	6.96858176976426	14.95009300200196
C	3.76745097110760	6.82342252890610	13.60755670193347
C	5.12564752700563	6.66995231540057	13.29206389590974
C	6.09092135177221	6.68755715125709	14.31819178530462
C	5.68729388523237	6.83677722615481	15.66418408313703
C	4.31824389124495	6.96197563574585	15.97753536641886
H	2.30175394419186	7.10065426981673	15.18933737045021
H	3.01784355720264	6.84001228340981	12.80707098427547
H	5.44279338417077	6.56263364847767	12.24812556739348
H	7.15294685341870	6.56043232523033	14.07897933665933
H	6.43691467065049	6.82016211773735	16.46348127317439
H	4.00822683045284	7.08437188912880	17.02176309179443
C	6.24783759971372	12.91995840370649	16.90271779511592
C	5.76254840774709	12.79248545052303	15.59326668999311
C	6.60369682675821	12.29346087156202	14.57729128408279
C	7.94289449144417	11.95503758977046	14.87557538390189
C	8.43167771273188	12.10573423248525	16.18828860656606
C	7.58133862997688	12.57460616848996	17.20089016953879
H	5.58452838170574	13.27526423243385	17.70035626574098
H	4.72221251324984	13.04819335043840	15.36172732169121
H	6.23412530290490	12.20433859005220	13.54938495469912
H	8.60560861371814	11.59848076328415	14.07861657667872
H	9.46803463441293	11.83105580503060	16.41733097925300
H	7.95107954272681	12.66292802249004	18.22972154847599

5

Rh	6.03847494864907	8.49856015243787	14.96134796953764
N	6.55873281939489	8.34594434518388	12.99794821156016
N	7.94665479660285	8.02316367728221	15.50299928065873
N	5.55172068735161	8.77016936959877	16.92033696217885
N	4.14661072153700	9.03056859484018	14.41805854806128
C	5.74158513931387	8.58016049924060	11.90047561144124
C	6.51740003284633	8.47072596315242	10.68029943070270
H	6.13202460728646	8.65005593551553	9.67531173806450
C	7.79636353589904	8.13206188034084	11.04571963266860
H	8.66133851705737	7.98020033971412	10.39807909312713
C	7.81290400008821	8.03246032028914	12.49222432445970
C	8.94260332091328	7.67144644709182	13.24929649869087
C	8.97853675473903	7.64475227998380	14.65527958551052
C	10.12234586379904	7.21531510071480	15.43608612454585
H	11.05979734691614	6.84243096777160	15.02021657973542
C	9.78214077699472	7.35595592216869	16.75850584985999
H	10.38691637641787	7.12016626351014	17.63559309343367
C	8.42809199800783	7.87351934034738	16.79630130292868
C	7.72753323065011	8.17891823770862	17.97746735099266
C	6.39453550462811	8.62805245764168	18.01416061524936
C	5.69540782647403	9.01463477341731	19.22426516121168
H	6.13753740436963	9.03998955110977	20.22154303660723
C	4.41646711429862	9.35377891994588	18.85913831508450
H	3.60852686236075	9.71135211946183	19.49947326457223
C	4.32327667037525	9.17656308592627	17.42301283249459
C	3.15278889012796	9.39015961510630	16.67172202707181
C	3.08179458811974	9.28919572413192	15.27024843480374
C	1.86447960036760	9.44963579853272	14.49893466594139
H	0.87515122076466	9.63353391766595	14.92097002363139
C	2.20457224614569	9.30854139883949	13.17654301456030
H	1.54739740965651	9.35429474364808	12.30664015378405
C	3.63232370948152	9.06015954109287	13.12903556677447
C	4.36782146224771	8.88191113206284	11.94301853728853

C	10.18437653402714	7.29440194995437	12.51048311632959
C	11.35098265273858	8.08588124267461	12.61349265682595
H	11.33441927171603	8.98772423399378	13.23720297579646
C	12.51204601182592	7.73458601530163	11.91586477086707
H	13.41924494469074	8.34633186840177	11.98276550502616
C	12.53328114740328	6.58162613138129	11.10349843414172
C	11.37329553809545	5.78496340201480	10.99767129556204
H	11.39089569525784	4.88645845241469	10.37200161188222
C	10.21128485658365	6.14143259882928	11.69373067831196
H	9.31189185672558	5.51860668786357	11.61767335439126
C	8.44279582180190	8.01705427349955	19.27841578407941
C	9.56109704243569	8.82292325774826	19.59013344064600
H	9.89653906164379	9.57571668610762	18.86665799769823
C	10.22778662196472	8.67856102978293	20.81345640484437
H	11.08830804720608	9.31170351431046	21.05365146701996
C	9.78814266917647	7.71811755652316	21.74927759008700
C	8.67459906939099	6.90832798661426	21.44243788668017
H	8.34991409659404	6.16286906207528	22.17763064539962
C	8.00802193592228	7.05767823805779	20.22129849249080
H	7.14688376079070	6.42266144940589	19.98128733659916
C	1.90578448897734	9.74755506604920	17.41160527068575
C	1.29573015725256	11.00927920806505	17.22663343812249
H	1.75330885485275	11.73028020273804	16.53866075261356
C	0.12927262071412	11.34369631078235	17.92350771187158
H	-0.34755106188405	12.32215636468156	17.79326617873381
C	-0.45440191234993	10.42225817541408	18.81799727940372
C	0.14744263566295	9.15987977038365	19.00638490217569
H	-0.30913291895904	8.44216247340563	19.69589584001068
C	1.31684136882305	8.82928444150373	18.31013915278783
H	1.78021672683736	7.84526348755021	18.44992831940277
C	3.64630793525273	9.02424118595790	10.64341310270379
C	3.09253326195375	10.26693711412803	10.26079811402695
H	3.20318364486043	11.13022462813771	10.92794723266664
C	2.42355381965948	10.40581965316878	9.03808341277635

H	2.00406889405304	11.37317707428701	8.74260907562328
C	2.29276122653862	9.29795872226579	8.17378758721736
C	2.84003967828149	8.05400575163627	8.55168224592114
H	2.72414626894176	7.20179088405810	7.87202309283454
C	3.51085811009774	7.91914026853793	9.77226948171434
H	3.92918824201827	6.94942030581613	10.06744633729406
C	1.59281064686727	9.38513674061929	6.85718864794966
O	1.46277087511279	8.45237867742775	6.07712128286592
O	1.11026642075726	10.62774649354222	6.61443709945818
C	10.45761803702440	7.51848882813178	23.06945274331026
O	10.10944508225084	6.69862228383937	23.90738771855238
O	11.50831196108409	8.35615923798303	23.24359721440520
C	13.80037615244357	6.25501556655863	10.38325425727820
O	14.82559009032279	6.91865181992572	10.44665411708408
O	13.69191555196102	5.12721663836314	9.64018233021826
C	-1.69926874841653	10.83252710791423	19.53420127818993
O	-2.25311688916609	11.91442920240908	19.39938606830352
O	-2.15610020846842	9.86376008306307	20.36413057988318
C	-3.35316951263029	10.18206910396955	21.09493394643387
H	-4.18970692344589	10.38424695482215	20.40343516379085
H	-3.57242291785089	9.30117452940922	21.71539027361581
H	-3.19696213670315	11.07185835091930	21.72938304754616
C	0.42545665092774	10.79692152923628	5.36123907441006
H	1.09953664952674	10.57431627664821	4.51572649806046
H	-0.44921783854510	10.12601569763578	5.30092880092394
H	0.10554223022135	11.84846659544338	5.33064472244455
C	14.87201021899356	4.74462348975910	8.91246746108613
H	15.15711200268183	5.53129109623305	8.19222087504292
H	15.71710809780885	4.57461148423276	9.60205360589392
H	14.61392822595521	3.81569098158620	8.38382840272099
C	12.20680652619896	8.22778408724231	24.49417024548859
H	13.01468421939610	8.97319126738353	24.46761290863734
H	11.52770412575227	8.42520952836754	25.34188495415371
H	12.62343334747776	7.21146262825775	24.60519220674832

H	5.63211817100392	7.04170077807881	15.01672145500424

Et_2MeSiH

Si	5.53126039401505	5.75651419288340	14.95610931100300
C	5.16297656152315	5.36183512935875	13.13604349750288
H	4.79070359345982	4.31479259719400	13.10326809816140
H	4.31241876092587	5.99291110493362	12.81106089821763
C	6.94449002490888	4.67466436039188	15.59602122884131
H	7.86772921759302	4.82836499940128	15.00980790171541
H	7.17428862236045	4.89491132967486	16.65354682003841
H	6.66726561556872	3.60360784871045	15.52239805898779
C	3.96769433490960	5.49682379567683	16.00077486409275
H	3.15821465376340	6.11978638091657	15.57186517750174
H	3.65013327902481	4.44246444870333	15.84756013727567
C	6.35593121456362	5.53063947881229	12.17857955453024
H	6.08426848050321	5.28248030316427	11.13561000586562
H	6.73413695570983	6.56980952086287	12.18469995044842
H	7.19974914097389	4.87535829562468	12.46270020374316
C	4.13304798523700	5.78725672642758	17.50299679616623
H	4.43682775080681	6.83574162453838	17.67994033709507
H	3.19392546545763	5.61478796234918	18.06112218764776
H	4.90781336869521	5.14274022037577	17.95736267116538

6

Rh	5.89887558672342	8.27868822317812	15.04144003950286
N	6.01018148525502	8.02425035880378	13.01212577079808
N	7.90270416118840	7.91135472624641	15.19171655884455
N	5.80631486400742	8.62087631383685	17.06029397901477
N	3.92123133650896	8.76746568281106	14.86858106893634
C	4.96238303521525	8.11893405301944	12.10948876950665
C	5.40402704994082	7.67690886955677	10.79982399742270
H	4.77354633482280	7.62179314503612	9.91088405852534
C	6.72850532811052	7.33235506139705	10.91720147183295
H	7.39023893224588	6.94280435245626	10.14194918290083

C	7.10916543024330	7.57370861708810	12.29637030540884
C	8.40554532989802	7.38009334861497	12.81245848352369
C	8.76597075549098	7.58287710979589	14.15847688493554
C	10.11695527604243	7.45427980848842	14.67333289754632
H	11.00073005515118	7.24338370338975	14.06900715843976
C	10.05299436802241	7.66799477782639	16.02789907500841
H	10.87452272326802	7.66543155728957	16.74611965722112
C	8.66165024103718	7.92638814506863	16.35180263531018
C	8.17136866157648	8.12461157364272	17.65723003617732
C	6.83073289571702	8.42184230201017	17.97195565619475
C	6.31067779787938	8.53006908164806	19.32277678003460
H	6.89605509406782	8.39376698266299	20.23355238986525
C	4.96969267948069	8.80422597462110	19.21637388938641
H	4.24644891978768	8.93373914744470	20.02301374308251
C	4.66007197911857	8.86757589132978	17.79958222585279
C	3.37366938082623	9.10084108878680	17.27463068703516
C	3.05594963703799	9.08710531346865	15.90253078240112
C	1.75277580971382	9.42876768702808	15.36112516866949
H	0.89522637430549	9.75768642104387	15.95048985460125
C	1.83521974324328	9.29059196579667	13.99771861667625
H	1.05733298620937	9.48250822063460	13.25679028915565
C	3.18594258398017	8.85242853416969	13.69622678771527
C	3.65090424599508	8.53860212398401	12.40543929010412
C	9.46858273488768	6.91601629429136	11.87317337502828
C	9.88612444059565	7.72647577426265	10.79329647651856
H	9.42767859173640	8.71318944929270	10.65608003806733
C	10.88447454922966	7.28807405507855	9.91411264495591
H	11.20945573412491	7.92372978439626	9.08379219610758
C	11.48403811005772	6.02368668246982	10.09799086059718
C	11.07112566767272	5.20950493454383	11.17351239083189
H	11.54284474202376	4.22811353989538	11.30013238645425
C	10.07606915072798	5.65193914960008	12.05193298837385
H	9.75199777625637	5.01536276079737	12.88352785634767
C	9.13304598274366	7.97162766235041	18.78865386134121

C	9.44704251539285	9.06481574694814	19.62730928758440
H	8.98823391833064	10.04073638229374	19.42810158152562
C	10.34279155557931	8.91379863712401	20.69357622756502
H	10.58813851773121	9.76571323173096	21.33632392246708
C	10.94034517205114	7.65983425587646	20.94349826401525
C	10.63129959345223	6.56463313663810	20.10955920231317
H	11.09944722496576	5.59612158670515	20.32051439008633
C	9.73901180348456	6.71958198462777	19.04321054661381
H	9.49115875926455	5.86574541472018	18.40127773384181
C	2.26318165333099	9.34953047378458	18.24021248052777
C	2.26909108620467	10.48782957141474	19.07797255026616
H	3.09509009086794	11.20503529529485	19.00045648843022
C	1.22928751786260	10.71096182579781	19.98956245103196
H	1.23588792522347	11.59837213234631	20.63115634235970
C	0.16074548352398	9.79358265777915	20.08251026359596
C	0.14826668729888	8.65608276004743	19.24830425150020
H	-0.68590254831699	7.95017607337136	19.33581937027450
C	1.18634636327912	8.43800676253804	18.33617542497549
H	1.17869764568134	7.54834350600963	17.69553690155066
C	2.67920957874941	8.61250288304276	11.27471631009761
C	2.86514742859793	9.54460052239478	10.22886727693766
H	3.72412108411098	10.22546935962450	10.26557790095141
C	1.96016503570848	9.61302827662464	9.16218164758400
H	2.10445306800416	10.34247570435414	8.35831948837644
C	0.84973871751630	8.74298237711755	9.12025397415808
C	0.66013537145544	7.80832876706616	10.15979508287934
H	-0.20340573838934	7.13489616605615	10.10881328316051
C	1.56393554715219	7.74506419676252	11.22630339094475
H	1.42069895016259	7.01135165020893	12.02843823777429
C	-0.14667775621383	8.76782501237538	8.00824420100375
O	-1.12130784804833	8.03293320807164	7.93199682994372
O	0.14874634432574	9.70601581799428	7.07590047876055
C	11.89733645322204	7.43626510392106	22.06783959916803
O	12.43963264519043	6.36858635473436	22.31650106499180

O	12.10452560560711	8.55998714672670	22.79654261903813
C	12.55139128211030	5.50221544525334	9.19316063211833
O	13.09732909283749	4.41490174826957	9.31754927834363
O	12.85661298933159	6.37575386694892	8.20293169205694
C	-0.97270066525303	9.97406718507658	21.03759376912303
O	-1.91713284951003	9.20473255568782	21.14766310253525
O	-0.84127502700503	11.09723980206390	21.78450726581169
C	-1.89428805453171	11.34023560185921	22.73336635765459
H	-1.96485168519941	10.51024960818598	23.45789933900041
H	-1.62996442525898	12.27630024453747	23.24619107528589
H	-2.86602352182605	11.44325730798002	22.21958147450412
C	-0.76496259955533	9.79454574646118	5.96904875861775
H	-1.78235314121931	10.03831895326062	6.32168023886288
H	-0.38048873884103	10.59643266225869	5.32230114143060
H	-0.80262682566317	8.83912784337419	5.41713632449032
C	13.87838752982070	5.94675202397560	7.28644460419558
H	13.57820026087310	5.01484203597966	6.77630803915369
H	13.99448268648062	6.76278054643965	6.55863810612580
H	14.82898062551774	5.76705801726286	7.81842908565810
C	13.01663695136139	8.42754207703766	23.90037720867241
H	12.65014524568371	7.67561678716155	24.62094333735389
H	14.01549563353308	8.11836065312452	23.54605810923990
H	13.06662858861434	9.41929713690268	24.37256453988920
H	5.53795284395443	6.62207112383469	15.53603133419791
Si	5.12147268484642	5.19932837131537	15.06319628449642
C	4.57942818857358	4.44834682872812	16.70443965643785
H	4.19947631469962	3.42339339398796	16.51634908953334
H	5.47663362842390	4.33574777241456	17.34307160646408
C	3.70945307841550	5.32734597389436	13.84420159564381
H	4.05064291352051	5.74265631350531	12.88111546805825
H	3.27686128447681	4.32668144861309	13.65907205855591
H	2.91221319994675	5.98360383188110	14.23416793812370
C	6.67291431585019	4.43861787490462	14.32800245221311
H	6.41912537586514	3.43741914559102	13.92454175717566

H	6.94811372402501	5.05845709044964	13.45214205054089
C	3.51954037574810	5.30682331793813	17.42022477982208
H	3.91098124805936	6.31460949068851	17.64537361284292
H	2.61584550352432	5.44280169792156	16.79796954315026
H	3.19796757581055	4.85331655722439	18.37561175935175
C	7.84812810005981	4.34892718646750	15.31737980178726
H	8.76851359899258	3.98005237791881	14.82845115778930
H	8.08181839503134	5.33595775385023	15.75388808484124
H	7.62433534442563	3.66245812291729	16.15397596519119

6-C_6H_6

Rh	5.76693909549175	8.24895622434700	15.19212355819938
N	5.86675761011583	7.96631561168329	13.16876834996846
N	7.74635213462535	7.75874356548402	15.35351027397168
N	5.67819387360385	8.58416899840512	17.21461834701992
N	3.79622261843408	8.77644981911160	15.02160586686661
C	4.84183810720183	8.15798075770576	12.25977345603994
C	5.27666041693621	7.74282524582796	10.93830375732844
H	4.65628223009300	7.75415106053248	10.04072391592421
C	6.57652135558897	7.31706745639900	11.05922798847250
H	7.22380264228676	6.91451324382199	10.27865290263606
C	6.95026774681788	7.48807414581018	12.45194651637316
C	8.23306599304864	7.22943342583753	12.97128473670841
C	8.59631479575721	7.40837267844065	14.32021492193115
C	9.95089873402118	7.27774308774396	14.82660595561238
H	10.82753004590980	7.04263320514829	14.22088676358212
C	9.90172206125007	7.53156075096231	16.17534492025606
H	10.73035434512478	7.54234677966492	16.88526497242653
C	8.51439447208033	7.80978767726668	16.50373890187098
C	8.04062303096404	8.07000837190639	17.80527662462919
C	6.70553671529278	8.39134920833225	18.12246873378131
C	6.19426419582144	8.53032450865084	19.47463315613022
H	6.78213291335026	8.40031419223450	20.38474476038588
C	4.85553865187236	8.81713338467261	19.37029451713931

H	4.13768350607766	8.96663264890622	20.17833033982862
C	4.54065733693706	8.86395933085682	17.95346291009244
C	3.26081274598252	9.12876575034570	17.42771470502744
C	2.94478435537424	9.12706017579057	16.05451855505245
C	1.66564766675435	9.54926364247609	15.51051067640844
H	0.82050449563674	9.90904314002966	16.09982589978764
C	1.75625528940544	9.44225925590515	14.14446303823708
H	0.99717983598791	9.69356294683215	13.40190573409533
C	3.08416450717911	8.93582361847560	13.84573641111504
C	3.55512433311377	8.64849341068971	12.55108313701329
C	9.28754312325570	6.75097066895398	12.03120460656258
C	9.71455844310550	7.55231240980819	10.94780873317640
H	9.26447279271510	8.54162477255945	10.80499658667109
C	10.70890054173549	7.10156874736214	10.07081217030023
H	11.04015808190990	7.73092686018824	9.23816805620290
C	11.29690227440281	5.83248575634030	10.25977270749916
C	10.87597519074758	5.02706142145955	11.33885164178789
H	11.33816431227448	4.04170447933791	11.46979011859893
C	9.88458706691514	5.48218459932725	12.21471802133643
H	9.55418811754657	4.85202099200614	13.04869600920677
C	9.01255939887687	7.96037158319875	18.93224354548840
C	9.32193274960397	9.08270348589241	19.73383333965078
H	8.85125216351116	10.04701566632081	19.50746588833447
C	10.22645530030480	8.97542131750915	20.79782755613002
H	10.46718965405206	9.84991810051965	21.41136061731742
C	10.83905189003520	7.73637989331019	21.08333068326220
C	10.53521587312406	6.61208013039181	20.28697233510824
H	11.01434690663352	5.65541591614716	20.52559474196675
C	9.63364054048072	6.72346681890345	19.22318221304462
H	9.38917274836414	5.84625918984905	18.61228514901904
C	2.15690471096811	9.41934763139926	18.38850199225880
C	2.19816969717870	10.55739455623675	19.22565699848850
H	3.05114330607502	11.24311392890977	19.15508036791421
C	1.15856750794697	10.82210110314804	20.12621109763002

H	1.19282728144252	11.71002434273620	20.76619767654373
C	0.05392095316994	9.94743200845598	20.20877167458792
C	0.00630361518660	8.80973450670905	19.37596332998665
H	-0.85573779968030	8.13717593186387	19.45538493267928
C	1.04442107059412	8.55047238935145	18.47487901350773
H	1.00928311629979	7.66145707948988	17.83420549038483
C	2.62495550609508	8.86299585384418	11.40515814786043
C	2.92453293546002	9.82759881734458	10.41606019162348
H	3.84518018866416	10.41568554113475	10.50844382538038
C	2.05789699112850	10.04046116357989	9.33698563930630
H	2.29152637903464	10.79491947752845	8.57857331184261
C	0.87146146717874	9.28425933084061	9.22379529891643
C	0.56935640436064	8.31580125072814	10.20447798322540
H	-0.35146419218621	7.73088636918372	10.09679349883286
C	1.43569318630516	8.10856657506363	11.28367469966927
H	1.20586037831397	7.34770172655453	12.03898786524766
C	-0.09024903283328	9.46456883220074	8.09598651825709
O	-1.12693067742048	8.83041855272991	7.95852470356871
O	0.31355628237423	10.42184017854937	7.22533809186363
C	11.80557222551084	7.55835740141533	22.20735762594832
O	12.36158212241567	6.50503682402353	22.48526918825364
O	12.00405114522870	8.70607369672691	22.90058140705189
C	12.35831258412484	5.29690643055412	9.35660503853992
O	12.89455173262568	4.20530571374636	9.48545867520061
O	12.66980785293217	6.16240020835015	8.36087745524029
C	-1.08280704866502	10.17506205927640	21.14956032366121
O	-2.05767248141338	9.44345669500714	21.25102082826194
O	-0.91830614506998	11.29684251389117	21.89239000107773
C	-1.97478725939472	11.58688869140633	22.82377267801080
H	-2.08755153368473	10.76614956946765	23.55352291465086
H	-1.68260299635823	12.51636192462520	23.33347535456948
H	-2.93438664936875	11.72247027385459	22.29492745781192
C	-0.56121173739780	10.65758283367287	6.10863631367656
H	-1.56034736481411	10.97392070002104	6.45572446522477

H	-0.08940370474886	11.45605494137252	5.51791952862653
H	-0.67256594821961	9.74292560670866	5.50042948592606
C	13.68502413919919	5.71825056953417	7.44440437539145
H	13.37421571556109	4.78650372205773	6.94037147116935
H	13.80738098800017	6.52894892168138	6.71168518792927
H	14.63510676844200	5.53200674171406	7.97504592287663
C	12.92296561876569	8.61662143377156	24.00301221390686
H	12.56918749087618	7.88121755264288	24.74658993061355
H	13.92386475029078	8.30958316966647	23.65257391171484
H	12.96298370163397	9.62207802119266	24.44629052990417
H	5.33482504140189	6.56936331212706	15.68505130397241
Si	4.85620093715748	5.17972401448621	15.19945610814254
C	4.25215648323691	4.40050125593392	16.80741976446057
H	3.76013753228192	3.43527757810087	16.56965376350515
H	5.13119262135014	4.15440209486659	17.43364448078144
C	3.45379462175197	5.39200607046642	13.97741973163201
H	3.80765537602000	5.84547799633795	13.03633120877648
H	3.00007065529316	4.41097727507043	13.74441962052586
H	2.66874644268182	6.04759590485775	14.39309278162851
C	6.37091385526174	4.35939785481512	14.44841461687652
H	6.07898362840330	3.37444295739760	14.03153377770551
H	6.66949162554404	4.98200055630120	13.58241677481696
C	3.29376787080017	5.33139440116318	17.57487497388779
H	3.79909958098638	6.27052099327414	17.86260637601272
H	2.41938147803108	5.61372738981240	16.95977148231504
H	2.90989240415409	4.86170319766633	18.49910354092546
C	7.54370848241082	4.21140225819223	15.43424792001237
H	8.45043610329635	3.81880368231894	14.93792452292652
H	7.81159528490244	5.18266443299856	15.88677078665438
H	7.29670081014552	3.51887604380465	16.25937959871667
C	5.71145457379149	11.24519366588444	14.58565342966194
C	6.98936527359723	10.88071601797068	15.06017304691653
C	8.03678412717099	10.64108080354765	14.14814090196737
C	7.79554960818328	10.73033021679167	12.76853546056580

C	6.51709201772898	11.08134632739156	12.29572808733504
C	5.47833419181418	11.34991128215293	13.20289725128804
H	4.90101137647331	11.44207056448804	15.29699806337865
H	7.17538607407325	10.81406877164073	16.13844786248364
H	9.02869793520440	10.35670818683306	14.51878333682269
H	8.60489995225862	10.52309309231500	12.05819636372787
H	6.33110980468361	11.13911807355314	11.21604325513544
H	4.48069320004629	11.61950116378930	12.83689586704418

6 (dimer model)

O	6.64331998204081	8.55736589262400	12.11972597114174
O	-6.64331998797181	8.55736597939021	12.11972599438323
O	6.64331990378182	12.11972607796240	29.95463428232155
O	6.64331995744802	26.39227403405161	29.95463416425953
O	6.64332012409193	8.55736598209691	26.39227382546850
N	2.03065946406279	19.06177203666575	28.93222065175128
C	6.42141491954130	12.72086147446104	28.84263833578861
C	5.24949378513800	13.62018767334821	28.78887850361587
C	4.41545881157386	13.74640850515176	29.91647053609536
H	4.62164674070421	13.13597741200151	30.80207826135294
C	3.37536179191879	14.67531501487505	29.89685063176199
H	2.73501397942042	14.82347236657414	30.77435423499212
C	3.18594586574439	15.48622414005548	28.75912200303962
C	2.21347156888128	16.59899828268225	28.82030882132662
C	2.75823193064938	17.89013725773972	28.88178526944189
C	4.17951004592113	18.18763785675792	28.92622369038291
H	4.97788568522021	17.44438996600059	28.93707911446651
C	6.51422158129492	25.67380001906288	28.98069687544715
C	5.63095556717067	24.48712199853607	28.95444530317067
C	4.92245997605918	24.21504643672972	30.14075411630265
H	5.10862687170998	24.84595674145719	31.01680132557851
C	3.98362169369551	23.18668291949296	30.16088456662606
H	3.39730804045599	22.97538764677602	31.06288089165511
C	3.75382472280580	22.43051888185235	28.99410682314748

C	2.64137018209601	21.45419460806665	28.98498574916164
C	2.95120272123862	20.09085469330611	28.96434671566141
C	4.29786790345879	19.55387632581016	28.97890951883305
H	5.20905914487564	20.15202223966803	29.03667018483911
O	7.13627416608199	12.61267985658396	27.81336563348829
N	-0.17548010703885	17.20537881274927	28.97935804556648
C	5.02003681662850	14.37730782697056	27.62336224173557
H	5.68020885546109	14.23469394796502	26.76069934267325
C	3.98728375414154	15.31483168511161	27.61156936028339
H	3.80853280570894	15.94525777374520	26.73278133712035
C	0.84970724006149	16.28812385255766	28.86043176372867
C	0.30876060737999	14.94659868828087	28.76302012558774
H	0.90252764102386	14.03896911378901	28.64087534464753
O	7.13627404838613	25.89931996168832	27.81336582777790
N	0.17548978726513	21.26725505604482	28.99847491226554
C	5.44607053624976	23.70029574655021	27.80197927311703
H	6.01479937876166	23.92587591950010	26.89387882816766
C	4.50608863091441	22.66553363758368	27.82623684605327
H	4.31375701588460	22.06049253707909	26.93220306361602
C	1.34808360597374	21.99558465829288	28.98269761300201
C	1.05064405732227	23.41470805613592	28.90571231802225
H	1.79523711471915	24.21093973279910	28.86434810068104
O	-7.13627431677238	12.61268025357116	27.81336603842117
C	-6.54687934036026	12.83501916456518	28.89417065325634
C	-5.63779439726599	14.00090154032794	28.99208837138937
C	-5.50232915391686	14.87190394935253	27.89479897356231
H	-6.11823257237061	14.70128038418896	27.00463560331694
C	-4.56520551619406	15.90483009151606	27.95453537157406
H	-4.41758301737412	16.58051444798787	27.10389270467730
C	-3.75517749331976	16.04792730591438	29.10016470822485
C	-2.63877028317624	17.02007457068790	29.09430733777855
C	-1.34950722022136	16.47910967186910	28.98392253808280
C	-1.05693891334021	15.06377139454352	28.83994814325606
H	-1.80361657261158	14.26960420989659	28.79432592433661

O	-6.64331985241702	12.11972583614804	29.95463389365367
N	-2.02585020497314	19.41237841595785	29.05863185811975
C	-4.88167243450667	14.18474970879208	30.16582144845118
H	-5.03523177554060	13.50091250106074	31.00718779932053
C	-3.93420658169760	15.20672094090895	30.21652197884377
H	-3.30506615726085	15.34965345651126	31.10299339589742
C	-2.94431170878268	18.38367514050321	29.14320109400216
C	-4.28740211284502	18.92154964640503	29.22708649033016
H	-5.19493541456601	18.32484104477362	29.33491475254076
O	-7.13627400189993	25.89931999735387	27.81336599801052
C	-6.42196305309256	25.76398688090559	28.78958849847535
C	-5.24443547429878	24.86917945425359	28.83716477291287
C	-4.99712538817917	24.08607836702529	27.69339779377929
H	-5.64199719576147	24.22005894151024	26.81777150429598
C	-3.97327213608501	23.14054042511247	27.71656625894862
H	-3.78245065311959	22.49412193372090	26.85165037097756
C	-3.19415629099443	22.98478170149997	28.88069104997814
C	-2.21668093739294	21.87594942524039	28.95003297638258
C	-0.85344879876410	22.18769555217935	28.94352325774893
C	-0.31641498161505	23.53248957511777	28.87181724573961
H	-0.91478065334506	24.44274283505862	28.80399953174595
O	-6.64332000014371	26.39227400487468	29.95463399981440
C	-4.43711327230558	24.75323700386305	29.98480016534944
H	-4.64490838276405	25.37859361217918	30.85898592560916
C	-3.40493829005813	23.81204916307938	30.00151169485641
H	-2.78362959946826	23.67525447553470	30.89455058444072
C	-2.75533819697723	20.58405713742390	29.03918396128444
C	-4.17219031237047	20.28795081945024	29.15336683844342
H	-4.96787835829605	21.03319774171261	29.19613855187880
O	7.13627381897857	10.69863408344637	25.89932025570022
O	-6.64332016090612	8.55736616902918	26.39227399409451
O	-7.13627400939468	10.69863402375223	12.61268000348740
Rh	0.00415413814346	9.64264930256730	19.26298521649475
N	2.02917675423402	9.56011182767199	19.42004772347339

C	6.54441330245497	9.66623537929870	12.86902376641280
C	5.61920062407665	9.50232779846415	14.00924456187578
C	4.84526611737310	8.33884293800074	14.18067122967692
H	4.97753111820060	7.49467938265652	13.49638801134552
C	3.89191178589866	8.29923548721143	15.20037832318869
H	3.24925713813704	7.42132109168824	15.33587091389373
C	3.72496239231852	9.41299734479430	16.04664306991087
C	2.61588771685972	9.44856991653617	17.02444622897461
C	2.93686145426061	9.43727568693044	18.38548718600993
C	4.28372570344074	9.32885581537082	18.91205975537665
H	5.18352310117233	9.19544606175134	18.30880702393745
C	6.42578330603954	9.66468007847345	25.78706800256317
C	5.27532526143924	9.70517530691369	24.86373735895569
C	4.44746329828131	8.57310566283957	24.73419968707235
H	4.65153832669520	7.69135220908345	25.35097401047524
C	3.41592976642329	8.58661325518609	23.79584320544656
H	2.77470222969216	7.70973622728521	23.64805717391431
C	3.23074326811870	9.72161220714230	22.98093292044947
C	2.24641796714209	9.67967329714507	21.87847005636785
C	2.77194808049628	9.57153788687825	20.58421460854125
C	4.18305547421084	9.42087545735169	20.27774257752371
H	4.98537268369647	9.37337855484238	21.01580972182871
O	7.13627402873236	10.69863411482687	12.61268002806461
N	0.16050000441408	9.64614764058972	17.23669437573317
C	5.48906681333682	10.59829692100141	14.88332273545605
H	6.11245303950020	11.48287930391864	14.71159271425901
C	4.54791953273786	10.54944567684270	15.90923981976133
H	4.40225387520032	11.40232593288457	16.58254389873506
C	1.32459284011305	9.58212330327367	16.49543409003551
C	1.02208710422142	9.71174413510250	15.08130412776956
H	1.76150462980407	9.72359907222963	14.27910525112844
N	-0.14978548602888	9.64815076139314	21.29105077419283
C	5.05633330179776	10.86437536083225	24.09445823894477
H	5.71444286034613	11.72822190036501	24.23770837634248

C	4.03377861482551	10.86849910250639	23.14729357729290
H	3.85926882125748	11.74205963626380	22.50834269220613
C	0.88722917851475	9.70901186842784	22.20244772469913
C	0.36366416561900	9.78659742335904	23.55201056305624
H	0.97006728603981	9.86980234473429	24.45582757130119
C	-6.38649242199741	9.58968882862257	12.71188869732504
C	-5.24674048458395	9.75514417197127	13.63823890592093
C	-5.05801874841020	10.93259146301771	14.38698006587065
H	-5.72859961493560	11.78436814271435	14.23481332583540
C	-4.03768205424498	10.97896931959825	15.33942878839683
H	-3.88827786385260	11.87067247199233	15.95964869214085
C	-3.20819568707213	9.85668367727924	15.53443777366670
C	-2.23182732368781	9.83063738560065	16.64536391194752
C	-0.87052832092748	9.77961042794365	16.32585309356550
C	-0.34034699820386	9.83896837367118	14.97732154666712
H	-0.93851913422024	9.97143271491357	14.07403500354478
N	-2.02275418504690	9.71635620923993	19.10612819069333
C	-4.38825701008988	8.65014230422550	13.79421146694260
H	-4.57118664447324	7.75467164128548	13.18977405852593
C	-3.36344392425488	8.70427124471671	14.73655376613537
H	-2.70442373219671	7.84394046305952	14.90235862676586
C	-2.76391043751196	9.76682236946181	17.94135558274412
C	-4.18112677913219	9.69768221418441	18.25036376074472
H	-4.98550239972807	9.69129557684614	17.51330119906074
O	-7.13627368945244	10.69863374453235	25.89932008157691
C	-6.51662462292924	9.61605127785236	25.68532911536157
C	-5.57604226108262	9.57660357714819	24.55112138080263
C	-5.42673588170745	10.72353068690694	23.74560712635729
H	-6.02203021090291	11.61474128207682	23.97277273646564
C	-4.49774420181867	10.71233093174204	22.70655282453614
H	-4.33560891153763	11.60020616924357	22.08429233603186
C	-3.71005989043132	9.56378421280655	22.48859798921840
C	-2.61061575167104	9.60608586508561	21.50143082882494
C	-1.31415605039456	9.64829384485718	22.03229384945552

C	-1.00433875252149	9.73974015813982	23.44812445598495
H	-1.74049533474161	9.78329605092034	24.25226750511736
C	-4.83121403783438	8.40771328230696	24.30282876337833
H	-4.98864414083346	7.53043755451471	24.93920066070360
C	-3.89388714859031	8.40496543025435	23.27080424264454
H	-3.27718961379400	7.52097289469169	23.07098893190720
C	-2.93524490668773	9.64359170986584	20.14187985917210
C	-4.28621917338403	9.61619398046512	19.61653651403485
H	-5.19240450384555	9.53476569646569	20.21967079523890
C	-7.78535606831904	27.26664509872800	29.99175485234395
H	-7.68638120234367	28.06765719204418	29.23876513002339
H	-8.71219275192347	26.70284277620577	29.78782604315798
H	-7.80841180771093	27.69161328793432	31.00544203277996
C	7.99293416741992	27.05455184513812	27.76933381592163
H	8.41631991064993	27.07878912014060	26.75501826273358
H	7.41535424046613	27.97372738116872	27.96941908434193
H	8.79613964335587	26.97128307022315	28.52180360008787
C	-8.28286333372225	10.60915858487804	11.74923756290676
H	-7.97454774962057	10.37259997992908	10.71599052552173
H	-8.97334962426462	9.82229602329135	12.09971538315575
H	-8.77002448248635	11.59400923430020	11.78923176233446
C	7.50376668077598	8.64746740776185	10.97088965447425
H	7.46373897809592	7.66238821996870	10.48420392559280
H	7.15059632080071	9.43365767286850	10.28100342558516
H	8.53745316126438	8.88537774564725	11.27664008106437
Rh	-0.00110473146189	19.23737475455284	28.91216519539691
H	0.17227968011728	19.13560375043311	27.21065070664263
Si	-0.58138485843117	19.33666286833578	25.83914566666889
C	0.58067939045904	18.33436945657559	24.74783070487219
H	0.14639475079827	18.31632955114252	23.72682002327009
H	1.54339474764340	18.87382614435409	24.66712232273966
C	-2.29550502498985	18.59871008470832	25.88113593533451
H	-2.69294686534242	18.56008917973280	24.84956751296332
H	-2.27840127635441	17.57049445590787	26.28044373840762

H	-2.98449876522532	19.19909366957599	26.49807792583366
C	-0.57832469310379	21.18440438635809	25.52605680873310
H	-1.12739362833694	21.36747589627150	24.57967692227447
H	-1.18605647745021	21.65089096535115	26.32510265502778
C	0.80613312689919	16.90608131563293	25.27640807939993
H	1.43944854981289	16.31152024404781	24.59304621542341
H	1.30296832026086	16.92347996047242	26.26199362783381
H	-0.14433865678730	16.35717582622444	25.40642177186357
C	0.82510652424674	21.81213931891548	25.47686187648054
H	1.41357245491749	21.42600686094023	24.62529008161632
H	0.77394254088303	22.91111588710451	25.37581213915208
H	1.39251158466445	21.59667757327406	26.39912011033421
Zr	-10.01324948579335	8.14036989046674	30.99959848581302
O	-12.13181802911513	7.66685607628949	30.71370449613389
O	-8.49271088584230	7.82385100535530	32.81746971663993
O	-9.07866068451731	10.08419017864004	31.27408102016127
O	-7.88845029301121	8.05159218410435	30.16998091396846
C	-12.90923096169856	7.28921458911142	29.78580502548553
O	-12.76078671447181	7.49946864442383	28.54796996307007
Zr	-8.07120522595956	8.04531226663553	27.94551405710424
O	-6.70706972274680	6.56464160076715	28.40499326157952
O	-10.02561401727489	7.44137631441062	28.81172241056475
Zr	-7.18709629418693	10.19858390062871	30.62955829647973
O	-5.25082171087443	9.72293473898396	29.50652865901198
O	-7.97455719387224	10.00465522361783	28.67037707196586
Zr	-11.40305164350777	8.88030413219248	27.46877252739223
O	-13.13623916491503	10.15471736769511	28.31252737992313
O	-9.82559984452213	6.16544726929956	31.19640312694530
O	-10.62136876544667	9.75410664961268	29.43556501083056
O	-4.80803880000536	7.33726022607927	30.13727539199709
O	-6.24336206663287	8.87120541045886	31.98263111245263
O	-12.42112247990340	9.12944212447498	25.76980581305513
Zr	-10.56733524856716	11.61179098524866	30.86002353727095
Zr	-8.91187479175798	11.31010676941450	27.11974733482347

O	-7.04678578612299	11.48120138405787	32.44693045327119
O	-8.80946718201159	6.50985145141436	26.50363068685584
O	-11.00111877323746	8.80416720094174	32.83927674757764
O	-11.03508883288366	6.93672334143672	26.30837893644390
O	-9.44120946294204	9.17686586569967	26.82688451849957
O	-9.58557659558233	12.18452887768358	29.04013809775410
O	-11.09749990930877	11.09997639299099	27.01730727734231
C	-11.24547621400755	9.89665182275725	33.45310955383724
O	-11.10936164257658	11.05918994938479	32.99253805499277
O	-9.57042954917873	10.72210050226329	24.78635670731340
O	-9.17010781506238	13.09842404348077	26.26289454040287
O	-8.93914993621911	12.68555491002041	32.10504013563150
C	-7.87762759418041	12.40616420590186	32.72605711979605
O	-11.18978412193404	13.49802314460183	30.98007501600774
O	-12.53027730805310	11.09155457629930	30.60634600809678
C	-9.97154844297693	6.31900140372103	26.01758425560247
H	-11.38731325192980	11.14434962589286	26.08487023245539
H	-7.45298708161566	7.22956998817455	30.46202847777656
H	-9.68267612685854	13.14395377543138	28.89830530220762
H	-10.19783606928143	6.48078265762892	28.79902277524429
H	-9.90509271573537	13.22503968585708	25.63947115796222
H	-10.62492469121387	14.14255885030197	31.44012937339035
H	-6.42086587926895	5.91760895395408	27.73848927921049
H	-9.24398762160993	5.82139625968363	31.89571232425385
H	-5.71802949693177	9.29758672319517	32.68379461627889
H	-8.82634137550955	8.34444097509878	33.56980500534621
H	-7.57266241427144	8.21100880270551	32.59568041172101
H	-5.18703258737698	9.94091773365999	28.56051083942409
H	-4.92477839809871	8.73670487781832	29.65640497374584
H	-5.28080818662591	7.58530337875021	30.97218724213683
H	-5.49221098936824	6.90916519198799	29.53578305138710
H	-12.50303441853949	8.35076328871046	25.19270096014407
H	-9.59977937514938	9.78343843532137	25.10709576815936
H	-8.75504184264221	10.79222005369640	24.25638105322032

H	-13.19368322318791	11.65472373804929	31.04088318198902
H	-9.66370231864610	9.90393081279170	29.19475603827520
H	-12.96816526668857	10.44600381937263	29.28513563719194
H	-12.84636159894789	10.96024566977191	27.82610412104537
H	-13.81218107022678	6.71762523516906	30.09559674583283
H	-10.05610456861693	5.50662334430955	25.25917241842058
H	-11.61256538975597	9.79591272735534	34.49929549067637
H	-7.62488792085937	13.03352810597772	33.61248200993013
Zr	10.24127588851936	7.67167163999658	30.27415931639817
O	12.35319066212475	8.09057451560406	30.64815438527076
O	8.83437122424358	5.79300363280971	30.68827084747567
O	9.22323832962680	7.32296493531057	28.36655492125407
O	8.09410785605091	8.40745255025471	30.44821734940402
C	13.06730270754573	9.05385718702013	31.06941334155546
O	12.85153601870629	10.28341023492120	30.88050139187491
Zr	8.16989297001637	10.65476496509871	30.40925988414213
O	6.92442419429712	10.14264418417108	31.96822948733863
O	10.17945181939221	9.89370175619424	30.91477512379423
Zr	7.30589077168923	7.89003399452765	28.32840000620828
O	5.31085262686919	8.85799222092345	28.88600285263037
O	7.96990994279261	9.87868055309764	28.49544744033843
Zr	11.47575461727488	11.23357661489424	29.39038383674524
O	13.27509863967850	10.31811641544289	28.27065618724928
O	10.15392934723925	7.51433256342518	32.25690085457268
O	10.70593164415733	9.21557676053942	28.58990807320231
O	5.08670988055275	8.27975749212555	31.31759346377553
O	6.52193732870520	6.48969331535047	29.70801664286527
O	12.49934095603371	12.89516725052269	29.02413566210360
Zr	10.64514809038267	7.74886787512124	26.76129108698626
Zr	8.90683891472593	11.36536326680770	27.12100132625636
O	7.15440227822005	6.08845924876048	27.04306950648332
O	8.91944887985030	12.19318092472701	31.82288498700694
O	11.29219354956019	5.88007666396151	29.59071099987566
O	11.09814084286493	12.49827086943524	31.25332001792488

O	9.45735704318776	11.79647485856203	29.12790583565848
O	9.53866946075953	9.49013129688261	26.15015943981003
O	11.08225826635162	11.47328871109120	27.21407980861390
C	11.49536316148830	5.23928394835660	28.50619558786812
O	11.27184912724064	5.65706219124129	27.34051975852078
O	9.36899768438503	14.51113173943530	28.44327191836952
O	9.08172252574758	12.96211929168293	25.97955070085165
O	8.98691123771880	6.47071783493390	25.76441881747079
C	7.95335032544769	5.82390604609571	26.08649091725277
O	11.24081299657108	7.50200658803272	24.88245735972034
O	12.60677490030326	8.11945029735639	27.19219953754464
C	10.05721670221522	12.76014715219826	31.91811292789784
H	11.37211724579039	12.36256275815112	26.93943291178751
H	7.72182844001456	8.11830842391627	31.30143728762598
H	9.16771007077775	9.37813138228978	25.25695037849987
H	10.40934339519028	9.94431175829939	31.86198498862607
H	9.15670489933862	13.79643297230284	26.49754063977423
H	10.68327268482855	6.97004258938327	24.28880002864512
H	6.62620263320169	10.80302450367070	32.61640472328563
H	9.61352166799752	6.80784417554997	32.64983948689292
H	6.01061450017865	5.76059604161046	29.31241833510331
H	9.18438482511555	5.03648768305086	30.18512981078867
H	7.89135453357790	5.96096984499359	30.32427360621708
H	5.13248951724349	9.78672795865148	28.65843512621522
H	5.07438285518435	8.70931104235775	29.89831368300421
H	5.58549545718238	7.46189563316980	31.06765051027913
H	5.75834886539267	8.92882746885984	31.69086954104283
H	12.55431213607293	13.56361815690780	29.72847824334158
H	9.44141225038914	13.61861979127759	28.86405796291091
H	8.42917685361957	14.73189743843218	28.55573202779491
H	13.26478492840658	7.70258519495081	26.60944293512007
H	9.72869770640198	9.38512092202513	28.50864813190386
H	13.04160598189380	9.39613910052139	27.86424088677640
H	13.32943368851690	10.89657543153929	27.48711368851756

H	13.97011845191876	8.78106256817372	31.65982329001907
H	10.13875700881902	13.56690188864032	32.68192214547644
H	11.90933585953730	4.21190306152044	28.61517955537109
H	7.69769210633538	4.92853085567287	25.47393536276178

7

Si	6.01017138987227	5.60177267307493	14.86750091916435
C	4.43863350356010	6.25319007897477	14.04676772449111
H	4.48247277527647	5.98050625494899	12.97353625967583
H	3.57550363009124	5.69573788449038	14.46260496105728
C	6.14705276616398	3.74532545509822	14.58711370416432
H	6.17910400031143	3.51463906274248	13.50686653903192
H	7.06478540705239	3.33800137519701	15.04973829515474
H	5.28285726674330	3.20979856955071	15.02047648495298
C	5.98680819819767	5.98720214158842	16.72023442298598
H	6.93004206465902	5.60673360397072	17.16150120665118
H	6.02118679119933	7.08801779847504	16.84529914175533
C	4.22299894470654	7.76858295498975	14.20616587099496
H	5.05642276406723	8.34518153979510	13.76341654297905
H	3.29272142812874	8.11102897993660	13.71542974141480
H	4.15714203107043	8.05992053085278	15.27102018954021
C	4.77319609204059	5.40567104887780	17.46864950069926
H	4.79761788778030	5.64360420239320	18.54883478820900
H	3.82222133232870	5.80304134753796	17.06803625840395
H	4.72864003181697	4.30454636884157	17.37754604804475
C	7.53171828961708	6.46755098899087	14.08230348919790
H	7.45554901609456	7.54167308967247	14.34586360899419
H	8.42393902085876	6.05954693822253	14.59894658677714
C	7.58116541794710	6.25147762054841	12.61832040423647
H	8.03222470773932	5.34538111864440	12.19695973581574
H	7.01998380712696	6.89986538691673	11.93500530172136

8

Si	5.53126039401505	5.75651419288340	14.95610931100300

C	5.16297656152315	5.36183512935875	13.13604349750288
H	4.79070359345982	4.31479259719400	13.10326809816140
H	4.31241876092587	5.99291110493362	12.81106089821763
C	6.94449002490888	4.67466436039188	15.59602122884131
H	7.86772921759302	4.82836499940128	15.00980790171541
H	7.17428862236045	4.89491132967486	16.65354682003841
H	6.66726561556872	3.60360784871045	15.52239805898779
C	3.96769433490960	5.49682379567683	16.00077486409275
H	3.15821465376340	6.11978638091657	15.57186517750174
H	3.65013327902481	4.44246444870333	15.84756013727567
C	6.35593121456362	5.53063947881229	12.17857955453024
H	6.08426848050321	5.28248030316427	11.13561000586562
H	6.73413695570983	6.56980952086287	12.18469995044842
H	7.19974914097389	4.87535829562468	12.46270020374316
C	4.13304798523700	5.78725672642758	17.50299679616623
H	4.43682775080681	6.83574162453838	17.67994033709507
H	3.19392546545763	5.61478796234918	18.06112218764776
H	4.90781336869521	5.14274022037577	17.95736267116538

11

Rh	5.95047240976278	8.37302653493117	14.95184980186731
N	6.51512266212382	8.36148527495446	12.97611634533642
N	7.87004207574211	7.92946198542788	15.49449650465468
N	5.49969063968930	8.79245517735568	16.91229585617848
N	4.06759773862981	8.95009249045562	14.40489833851858
C	5.70410136814829	8.62388326465797	11.88599917112903
C	6.49682106160382	8.57737795511119	10.67089229886629
H	6.12340291169801	8.79627553017632	9.66906033023719
C	7.77514387378299	8.23186059461261	11.03792939959708
H	8.64872923928465	8.11392724902117	10.39455349478815
C	7.77259752863036	8.06612159560259	12.47986677554247
C	8.87878588514374	7.63311575037831	13.23999310571836
C	8.89750559226082	7.54808212405139	14.64676903014747
C	10.02772236802107	7.08217202923467	15.43104139425486

H	10.96062351130725	6.69412738538706	15.01862584561476
C	9.68620181922372	7.22623830160552	16.75317698496172
H	10.28555027059326	6.97853729322494	17.63083708932141
C	8.34478503550441	7.78205668860823	16.78785996624308
C	7.66047092165620	8.15053414310520	17.96365812766783
C	6.35035415751469	8.67139547610075	17.99696916669463
C	5.67274557442509	9.12731047148602	19.19679834107833
H	6.12562239765734	9.19001200954193	20.18777751322749
C	4.39354490674516	9.46998791239698	18.83010815311413
H	3.59897696225160	9.86819602797962	19.46342590381511
C	4.28067747787875	9.22515452293402	17.40381154778467
C	3.09388855795295	9.37038468102915	16.65529963128674
C	3.00669631885770	9.20899112094439	15.25748642474505
C	1.78303371428098	9.33696531810603	14.48540298368757
H	0.79060877114031	9.50911822660178	14.90525511212522
C	2.12542246087020	9.19788793285240	13.16296586724362
H	1.46676374499157	9.23406646625724	12.29363645612874
C	3.56085804730225	8.98191055344237	13.11569524893011
C	4.31473898962223	8.86284670636547	11.93048681990639
C	10.11790733986552	7.24929796059215	12.50089511248383
C	11.31287899560996	7.98729466504348	12.66302666400809
H	11.31973147870605	8.85626138060437	13.33185045022385
C	12.47159724799650	7.62665580337508	11.96643785496871
H	13.40043870452647	8.19784684638451	12.07912488757608
C	12.46281039465338	6.51699128076448	11.09550467069382
C	11.27423274131549	5.77469215161766	10.92843217721854
H	11.26821526727952	4.91021464511653	10.25631690296582
C	10.11478364724886	6.14121507133181	11.62332334125589
H	9.19284585245537	5.56043722958538	11.49940049800744
C	8.37033024187290	7.98709251626701	19.26705502844397
C	9.53300154848309	8.73712172409526	19.55594437613983
H	9.90798087810113	9.45014875497377	18.81193248840802
C	10.19297967313286	8.58997556723736	20.78255287178276
H	11.08774445516185	9.18086729783422	21.00471614676990

C	9.70254869839090	7.68196520942698	21.74522084677054
C	8.54423556142640	6.92826477252641	21.46205511068618
H	8.17970917205807	6.22320911567847	22.21816475916197
C	7.88424567225096	7.08145384058638	20.23794250168350
H	6.98753469572176	6.49007273422706	20.01718780253605
C	1.84828185593220	9.72824747235997	17.39635369458699
C	1.18534118341358	10.95222476776603	17.14769531574771
H	1.60290680753396	11.64754924187673	16.40972957030015
C	0.01863883048344	11.28398294165451	17.84526678627463
H	-0.49816429907143	12.23375805038870	17.66470877477530
C	-0.51305712461356	10.39823833837173	18.80583367623291
C	0.14255015039343	9.17478849093247	19.06051115504492
H	-0.27270420640993	8.48451978974153	19.80223706847482
C	1.31168420821762	8.84698211280105	18.36275470345825
H	1.81636535253132	7.89251554780649	18.55472798390236
C	3.59872139827062	9.01163769379773	10.62887198124728
C	2.98172541178052	10.23498040474490	10.28114719171397
H	3.03976674836544	11.07996778338975	10.97763842317448
C	2.31908598372416	10.37962832573457	9.05574893518499
H	1.85136154650409	11.33279587106393	8.78784142518296
C	2.25798661445153	9.29700444380971	8.15251014084907
C	2.86906627885107	8.07244694213148	8.49442568019887
H	2.80764887613807	7.23958807139207	7.78431648883917
C	3.53339177082584	7.93223419357486	9.71786814519674
H	4.00228005986820	6.97739691886511	9.98417705617668
C	1.56960286934797	9.39226019384335	6.83059849845896
O	1.50059591175115	8.48230334945501	6.01640507085063
O	1.02250751122804	10.61464016637923	6.62441772009033
C	10.36258317238303	7.48149862563170	23.06980066080201
O	9.97436171133685	6.70133458204872	23.92793207358654
O	11.45379113738055	8.26993592569806	23.22386694565930
C	13.72921830714496	6.17658931136640	10.38089225839037
O	14.77784907082353	6.79574115907711	10.49333872989377
O	13.59237068106832	5.08907611129662	9.58397835545866

C	-1.76155599075322	10.80305872983906	19.51834002153769
O	-2.36391296602781	11.84947072342162	19.32394056369740
O	-2.16362349563680	9.87206996653583	20.41727358674311
C	-3.36212068843050	10.18682845522797	21.14706137755998
H	-4.21704490390448	10.30730496190228	20.45895384282192
H	-3.53359519069985	9.34028824002385	21.82750142687533
H	-3.23541592308555	11.12236410032988	21.71926328969158
C	0.34534511221226	10.79087418253266	5.36813852774469
H	1.04042215531228	10.63257252738509	4.52515382428359
H	-0.49232873915942	10.07802716998113	5.27307331609687
H	-0.02878708876364	11.82483168641871	5.36834312854142
C	14.77103984573613	4.69501350611121	8.86018912308603
H	15.09492883095252	5.49913542280226	8.17648032588730
H	15.59804261419220	4.46903291530528	9.55558403613407
H	14.49073371398963	3.79724115402529	8.29058648828915
C	12.14578049650350	8.13798525803931	24.47763177424762
H	12.98461705510097	8.84768978341039	24.43668703789659
H	11.47535174732771	8.38112741957238	25.32032400241674
H	12.51913119299245	7.10745591190980	24.61003580394209
C	5.17032227833346	6.22124265827717	15.69999737260732
H	5.88992796091087	5.96188707375865	16.48583941731224
H	4.14494027633344	6.43465171544865	16.02492133598688
C	5.48585842641611	6.08070783166574	14.36783439976136
H	4.72340041050366	6.17885517874468	13.58572228290325
H	6.46778265943327	5.70797437912040	14.05227327198011

11-C_6H_6

Rh	5.92223000180978	8.27898232772590	14.93050298455722
N	6.46790350358207	8.16904635823099	12.95569912914886
N	7.82918627157284	7.79161617605149	15.48189218911619
N	5.46115006629092	8.68237407779868	16.89348291512123
N	4.03568994705700	8.84027296870453	14.37892877196787
C	5.66668505036729	8.44921113034683	11.86525515491667
C	6.45988335697556	8.38643466870738	10.65012733543329

H	6.09069652483861	8.60689275729991	9.64718455434309
C	7.73291056933541	8.02930728780989	11.02103095799169
H	8.60677242279965	7.90083697518188	10.38025081793172
C	7.72543869192735	7.87349469578285	12.46470491789175
C	8.84154490629313	7.48636199964602	13.23122956812171
C	8.86451177502208	7.43031611126747	14.63817796737384
C	10.01592642680538	7.02365353572568	15.42578630317809
H	10.95844392591589	6.65640343101174	15.01605852735166
C	9.67613060528671	7.18520256526637	16.74618739917823
H	10.28684808596344	6.97632211530515	17.62611218934991
C	8.31565239299083	7.69589346781711	16.77448010638259
C	7.63220365930618	8.08039857625025	17.94539135669774
C	6.31458625081175	8.58122140919930	17.97684002133341
C	5.64067459084642	9.05715155891593	19.17205582541430
H	6.09438890781883	9.12972048575420	20.16199029625048
C	4.36372215403711	9.40316256234421	18.80149941099894
H	3.57193251497182	9.81479302622081	19.42975717164849
C	4.24968386907555	9.14085751435121	17.37760805756351
C	3.07098465035932	9.31318446080227	16.62276950822746
C	2.98380207038754	9.14456449461098	15.22607479649030
C	1.76857658034339	9.32110925527048	14.44876132442483
H	0.78133746019736	9.52967480223369	14.86434732458883
C	2.11228960632690	9.17125003361722	13.12796749333175
H	1.45998920150218	9.23186662192287	12.25513738908773
C	3.53848904378424	8.89605696219795	13.08863587637576
C	4.29074111126239	8.74903875298149	11.90641165383441
C	10.10394190450347	7.17247173789357	12.49987292527983
C	11.24303541672947	7.99567626795929	12.65689919317747
H	11.18607909529398	8.86478923090898	13.32291251843848
C	12.42447804005083	7.71631242872263	11.96140524070260
H	13.31053884992883	8.35260113534764	12.06999228377690
C	12.49441447892674	6.60450034093175	11.09567205168345
C	11.36214565694114	5.77767362769107	10.93361551022806
H	11.41836002127916	4.91219224533176	10.26520539874678

C	10.17978986982476	6.06278644860194	11.62830279828163
H	9.30110958083119	5.41781515824451	11.50816428178783
C	8.35618072868467	7.96819069566848	19.24664893055295
C	9.49581340302752	8.76136887537354	19.51065567489042
H	9.84313792283312	9.46796320649176	18.74731426855536
C	10.16834220924778	8.66349954833728	20.73538408231714
H	11.04539759960485	9.28708545517571	20.93786138849114
C	9.71320715045067	7.76260279107167	21.72186365000037
C	8.57746875459457	6.96647422593836	21.46410708633775
H	8.24038548793434	6.26805201137718	22.23886769430795
C	7.90532551849298	7.07032857914731	20.24135974174928
H	7.02566054393905	6.44731356296883	20.04009246004671
C	1.83419948360677	9.71500275156406	17.35609108629948
C	1.21728779740513	10.96274377670633	17.10779900435409
H	1.66416102664850	11.64447810745246	16.37425912191937
C	0.05805619187130	11.33390789612156	17.79810158586366
H	-0.42346200995768	12.30191528334136	17.61678600002669
C	-0.51211328405507	10.46439395509900	18.75148654886143
C	0.09802153185109	9.21764437165113	19.00663577194926
H	-0.34696880836480	8.54031835317904	19.74302917804883
C	1.25975721725434	8.85078718636803	18.31605432059596
H	1.72940913302475	7.87867322593276	18.50853049813313
C	3.59242246463702	8.96192089967520	10.60480128862269
C	3.04994833429072	10.22671887740785	10.28095529087761
H	3.14881304642252	11.05014163884521	10.99829400202493
C	2.40673819064750	10.43821791212143	9.05495573108897
H	1.99614700260056	11.42256941408012	8.80671743598393
C	2.29077902059367	9.38220543855795	8.12584090845935
C	2.82791897059832	8.11693349442432	8.44301126613074
H	2.72460784850511	7.30554006760785	7.71317183248089
C	3.47246684646412	7.91014876103310	9.66759003537591
H	3.88309294493325	6.92390007202210	9.91492681137786
C	1.61677870084555	9.54660158902552	6.80359302092913
O	1.50077734134032	8.66094832894809	5.96815421247030

O	1.13902960186772	10.80209008747260	6.62405639369495
C	10.38814671544685	7.61284934817206	23.04546838173977
O	10.03037662251850	6.84089963988063	23.92406990430899
O	11.45645721801569	8.43687567096783	23.17319497464091
C	13.78163672818257	6.35125702244125	10.38234515149264
O	14.78481119565375	7.04200846077882	10.49332281350345
O	13.72032635433908	5.25589338074014	9.58659362161649
C	-1.75136553831198	10.91005707880941	19.45534389689104
O	-2.31334452610254	11.97909096159296	19.26290986070375
O	-2.19512932277358	9.98889820303746	20.34491836786338
C	-3.38817065842217	10.34223317982245	21.06573947991808
H	-4.23093930082706	10.50124363766463	20.37051174073155
H	-3.59835796712189	9.49671807689518	21.73655414955180
H	-3.23217136730582	11.26704966639799	21.64809882149610
C	0.47464050801924	11.04307407648830	5.37170308456421
H	1.16417060715045	10.87546894783827	4.52590872496608
H	-0.39457256874273	10.37234953823651	5.25632492957435
H	0.14885134269182	12.09292569541658	5.39881687309040
C	14.92375079025718	4.94095805965050	8.86518034806745
H	15.19807183883808	5.76642121701772	8.18520904221734
H	15.76154786293370	4.76524959800505	9.56226782367781
H	14.70318661383433	4.02914316825742	8.29160613268318
C	12.16119156165666	8.35342218197736	24.42394621476104
H	12.97887291519404	9.08579608654276	24.36026414529913
H	11.49035450292908	8.59635660880241	25.26638931647966
H	12.56506580349039	7.33729711212656	24.57660771726722
C	5.07371367680816	6.03267716965401	15.72935581958727
H	5.78696608816577	5.82165953361556	16.53523350684813
H	4.05171615633660	6.29335611890667	16.02977533095248
C	5.41295057982773	5.86903543133282	14.41523604801600
H	4.67282558759331	5.98303695269904	13.61398312114422
H	6.40680837260709	5.51034643564452	14.12135005687911
C	7.40071378732902	10.97171278945136	15.25722509715802
C	6.12237170512452	11.31478034054047	14.77218990405005

C	5.92517267473781	11.51949136932036	13.39411714581833
C	6.99792054751178	11.36016696611308	12.50183189817812
C	8.27247813042689	11.01117757973512	12.98566026554533
C	8.47650933322973	10.82611654316123	14.36323569856618
H	7.55138205479822	10.80942125881698	16.33096278708532
H	5.28427798452586	11.42437461973221	15.47052749968927
H	4.92731012948855	11.77251314699095	13.01666202799221
H	6.83888859014021	11.48946938519808	11.42460113225843
H	9.10399082463739	10.87004369005848	12.28440963708630
H	9.46549881315166	10.54136157329425	14.74232064549155

12

Rh	6.09245874185308	8.33062302251929	15.10701803169008
N	6.31905506214658	8.33743038175330	13.07236049440308
N	8.03389625281717	7.72752450514109	15.32166579769977
N	5.89031958525808	8.39696346679435	17.14458409759548
N	4.19274957292920	9.05172219681177	14.89893431299024
C	5.33653045597965	8.62092492150402	12.13699483763911
C	5.81702954671737	8.28990677663374	10.80783466684180
H	5.23853558837881	8.39023875934222	9.88801894917289
C	7.09838727034519	7.81799831090127	10.94976769107328
H	7.76936522277255	7.45771679430400	10.16838328575042
C	7.41249337980117	7.86084696400103	12.36635851670629
C	8.64054759689109	7.45254600441745	12.92314967446531
C	8.92533892600167	7.43333403702681	14.30239342406371
C	10.22003845657555	7.09760483892143	14.86692746857620
H	11.11478363281229	6.86339901431661	14.28769380138823
C	10.09270150898159	7.16548239793327	16.23219081789189
H	10.86283096205456	6.99597211236186	16.98655148985375
C	8.71688828189363	7.53517854341641	16.51258396107254
C	8.17091454606302	7.64779642096479	17.80514074028928
C	6.84046785677029	8.01922738459144	18.08047144872351
C	6.24455075948540	8.00588184234382	19.40364632527772
H	6.76027687234885	7.71930892413430	20.32156122025893

C	4.93423960531163	8.39151746160198	19.25848884196555
H	4.17356490742535	8.48376120323817	20.03538670155540
C	4.72339623326392	8.65623803355963	17.84755425092277
C	3.51088070313412	9.10953420658274	17.29199157273222
C	3.29708875853966	9.33335290632160	15.91833853620458
C	2.07874933784653	9.88940474152966	15.35908797041788
H	1.22427864910475	10.23956550158960	15.94059603667629
C	2.23477502110831	9.90775988687086	13.99524440053293
H	1.53205797579017	10.27359411027877	13.24496821699886
C	3.54624282216331	9.35443636219271	13.71030858920305
C	4.05164398125231	9.12722726349546	12.41543433673203
C	9.70761618784207	6.98851664385675	11.98830309950572
C	10.28161888229982	7.87372864710255	11.04810307821334
H	9.94541666294839	8.91718689953454	11.02185479912619
C	11.27803673296866	7.43398497942990	10.16751869878728
H	11.72499839346916	8.12584516512630	9.44599015179397
C	11.71763790946969	6.09345579762782	10.20902107455913
C	11.14883485988246	5.20479583628924	11.14546917237116
H	11.49756656990326	4.16563502584491	11.16136227966511
C	10.15687657433220	5.64833300287304	12.02688246276906
H	9.70936711354902	4.95527204015507	12.74893746520697
C	9.04246348107385	7.29580784024827	18.96462722490629
C	9.39277625981211	8.26974804336374	19.92675907188795
H	9.03189326436030	9.29779560635770	19.80234585736825
C	10.19998660519999	7.93574034910190	21.02160466044051
H	10.47431830535888	8.69591951664473	21.76047693117383
C	10.67033592898571	6.61411737593068	21.17671017950592
C	10.32266365945428	5.63680393551178	20.22052290696907
H	10.69008541739080	4.61332052603127	20.35889512556570
C	9.51912122529341	5.97451047887316	19.12606577636696
H	9.23907162747034	5.21152162495890	18.39015892574733
C	2.36634697559311	9.35199018958983	18.21842939504596
C	2.43130912499051	10.36051978265875	19.20611580506180
H	3.33180294845554	10.98223646077132	19.27770170598732

C	1.35590017126778	10.57926795906168	20.07639781401994
H	1.40801843925288	11.36674455542800	20.83548923275475
C	0.19172932862006	9.78777333724431	19.97572459237165
C	0.12008340943239	8.78000336963059	18.99103633804959
H	-0.78922324110465	8.17091102424737	18.92864489644804
C	1.19471628480901	8.56634300208527	18.12103735162898
H	1.14250498446216	7.77832311237803	17.36045066629881
C	3.15152971383206	9.41350499151314	11.25970314976356
C	3.48257405427472	10.41123969307915	10.31445249270991
H	4.40710157536847	10.98623985339922	10.44478555277566
C	2.63541644150452	10.67483148653184	9.23235596930583
H	2.87906037368292	11.45158055838126	8.49823325991690
C	1.43961583208833	9.94417272998141	9.06938744093362
C	1.10306679632591	8.94458337372893	10.00738528587362
H	0.17843143393047	8.37251587600716	9.87770320387165
C	1.95159694733191	8.68524599536601	11.09070684543790
H	1.69680317876651	7.90301916007353	11.81578384157339
C	0.57493806588494	10.26748338758535	7.89580018479240
O	0.83146804390421	11.12068949824827	7.05798832072186
O	-0.54343784823653	9.50278003253199	7.85617577180880
C	11.52986789357827	6.19722422208454	22.32447849016270
O	11.95729135889574	5.06428966040004	22.49652238333283
O	11.79030738876742	7.22346303923219	23.17057703142752
C	12.77306954334311	5.56630372350268	9.29356651717527
O	13.18001936790268	4.41297893749223	9.29386667824928
O	13.24037685969069	6.51676687354500	8.44788447963998
C	-0.98450836332236	9.97315596492286	20.87680171011906
O	-2.01388873303317	9.31522559070195	20.81966021823458
O	-0.78712851261702	10.96387025163746	21.78030631020236
C	-1.87601060390938	11.20702851230575	22.68743649390656
H	-1.54562767092175	12.02394357736651	23.34514144236660
H	-2.78545327440073	11.50134568687025	22.13496775622546
H	-2.10005674194930	10.30228987818847	23.27907572315382
C	-1.43118982619531	9.75416213076117	6.75324241547297

H	-2.27190057878646	9.05617290075296	6.87608625250098
H	-1.79083795880854	10.79780135917879	6.77230326261950
H	-0.91902330164401	9.57541308110075	5.79168439311233
C	14.26362955910256	6.08841628215653	7.53297010662363
H	14.53034137690015	6.97525880886437	6.94012669113122
H	15.14456497613154	5.71191113739722	8.08157413248248
H	13.88780773565608	5.28482566521395	6.87569128353620
C	12.61289230815911	6.90146476463222	24.30535642779248
H	13.60278922900928	6.53768993230117	23.97878468200564
H	12.71676412429051	7.83412285129444	24.87844050357181
H	12.13630274623121	6.12028538387851	24.92292998227191
H	5.56226992734317	6.66316800919636	14.87765828745287
Si	5.07966980140152	5.31229481291267	15.48543890382383
C	5.23675456481940	4.21828768059166	13.95920980101048
H	4.90180770636378	3.19367594353833	14.21167393513283
H	4.52863045966341	4.60003392341412	13.19851019435741
C	6.24573741276304	4.82738089983284	16.86247647626726
H	6.05214080934659	3.78255943242113	17.16546795457225
H	7.29721077106288	4.91287797308150	16.53831539008057
H	6.10605875681793	5.47722675974215	17.74310591093987
C	3.32519989424778	5.63146986378770	16.06801791325102
H	2.96539273947157	4.72647572464782	16.59576084034394
H	3.38463246912738	6.43121307560616	16.83173953816877
C	6.66970681417444	4.20648205264941	13.39519078696446
H	6.74529709030343	3.59951974478406	12.47436472140286
H	7.00898734225532	5.22802211824363	13.14606818978152
H	7.38989140178958	3.79263038191889	14.12447193447660
C	2.36042053719490	6.03350908502849	14.93931518655882
H	2.21432821908415	5.21024029195370	14.21661612823406
H	1.36356767559514	6.30936886718493	15.33012404109692
H	2.74386894144620	6.90367760739644	14.37793088349081
C	3.13683029554515	1.99249598128671	16.34535294428636
H	2.35829573227414	2.15546787794985	17.10225308932591
H	2.85643518263500	2.19341796069271	15.30284294006114

C	4.36720618657754	1.56757574377454	16.67748424438300
H	4.64666747889582	1.36541525907586	17.71990089187335
H	5.14511039425789	1.40121132675077	15.92042900356244

13

Rh	6.02213224993273	8.27287707792292	15.17027220704321
N	6.20129656447035	8.13482851441514	13.12881269592663
N	7.94668192715224	7.61379701624153	15.38782447316107
N	5.78352352442716	8.23903182609235	17.21367402664793
N	4.09793567411202	8.93136994279106	14.95712493919837
C	5.21449679333692	8.40520117338690	12.19926369725577
C	5.67203046212661	8.00968100133060	10.87826286603572
H	5.08142672142157	8.07508587514443	9.96291418340606
C	6.94986118267105	7.52734578944015	11.02371961101742
H	7.60417453161737	7.12268152902386	10.24990908886500
C	7.28533160485379	7.63198464582601	12.43375295002052
C	8.53150405502141	7.27763608141139	12.99209068562231
C	8.83445489862763	7.31691107289177	14.36869821826757
C	10.14102929555384	7.02355718606547	14.93298519659030
H	11.03747216764763	6.79327349986383	14.35469709437913
C	10.01841972681446	7.11267255429577	16.29781143247018
H	10.79495145922593	6.96729140004934	17.05069997627762
C	8.63334944339711	7.45140740981263	16.57905164306259
C	8.07849037419251	7.54184815270187	17.87146718694133
C	6.73221836156727	7.86070505228435	18.14483254594065
C	6.12217509530714	7.80322812123866	19.46201842553796
H	6.63052244583575	7.49489785947005	20.37710025785481
C	4.80792340895578	8.17620418114613	19.31249305260458
H	4.03701910290769	8.23513850393372	20.08264354498625
C	4.61042340300241	8.47872424974188	17.90563891419589
C	3.41144338040679	8.96902622481099	17.34871449543111
C	3.20940817735162	9.22342955098347	15.97704951975526
C	2.00181345983456	9.80529470323669	15.41714019057694
H	1.15016565001119	10.16504297254029	15.99703846917015

C	2.15924064282517	9.82279330459504	14.05304657627256
H	1.46064463913563	10.19763603438804	13.30333615621522
C	3.45979335112877	9.24077438898860	13.76809542268856
C	3.95272582924492	8.97248282686292	12.47441497742860
C	9.59458331765784	6.79845595633532	12.06096701871108
C	10.11661984646861	7.64680485505179	11.05808147940732
H	9.74578474076011	8.67603662054881	10.98275095395717
C	11.10352708263303	7.18949479828143	10.17585736252107
H	11.50848635872847	7.85301511509909	9.40461014237428
C	11.58741283756366	5.86765997074959	10.27934098593006
C	11.07267711304535	5.01629704515166	11.27965060606778
H	11.45470354388284	3.99076608013314	11.34312667717768
C	10.08897028233076	5.47721916815905	12.16123809205392
H	9.68060971078697	4.81111864237872	12.93029653067350
C	8.95480617347066	7.21445135739623	19.03408778084620
C	9.24316039726777	8.18766892767831	20.01770511542475
H	8.83167896149693	9.19805636558453	19.90619513990251
C	10.05236247770611	7.87637964209436	21.11769395167219
H	10.27813636589705	8.63652817695525	21.87287215887599
C	10.58734616734618	6.57780191228845	21.25745629050581
C	10.30151039067989	5.60074330199914	20.28069795736691
H	10.71731556769912	4.59446047395023	20.40762904039491
C	9.49586622550175	5.91613445433707	19.18134011549748
H	9.26259020258389	5.15182936707157	18.43075059966796
C	2.26600293619171	9.21523822157916	18.27249587572196
C	2.34961425606811	10.19188179202117	19.29061528964697
H	3.26695484276514	10.78459736938486	19.38932761975940
C	1.27202994313271	10.41698111723686	20.15644767361030
H	1.33872214702204	11.18053530092130	20.93850582159159
C	0.08635116929127	9.66337759457783	20.02120800355984
C	-0.00326251941622	8.68555079798535	19.00811023638071
H	-0.92864231002096	8.10449667544154	18.91993152461167
C	1.07387424515873	8.46560196809184	18.14276562985213
H	1.00834052106713	7.70031455214940	17.36027154368647

C	3.06327354530828	9.27318896701816	11.31461098458855
C	3.44669837384112	10.22217982787827	10.33903235620725
H	4.40243207541499	10.74819998575627	10.45028222342542
C	2.61268516361811	10.50180649080025	9.25093335807050
H	2.89759730404596	11.24179923522910	8.49402146076492
C	1.37678001644382	9.83595212163527	9.11138822657122
C	0.98802082622595	8.88395875548713	10.07831015541009
H	0.03277817165867	8.36044306318906	9.96648635844184
C	1.82414064445625	8.60817395339549	11.16722436288703
H	1.52894812438649	7.86058903939363	11.91320026962714
C	0.52714406596032	10.17516553469837	7.93142452508780
O	0.82956123659325	10.98683563325402	7.06789793712343
O	-0.63503643254215	9.47788041573808	7.91857648739624
C	11.45147767474360	6.18471616548417	22.41002525315497
O	11.93455087064172	5.07235407749919	22.56823020269831
O	11.64622875722067	7.20694924760689	23.27860532406002
C	12.63216123377733	5.32128145646360	9.36309141986789
O	13.08358461227674	4.18616967609235	9.42252491137082
O	13.03246635368564	6.23031331079194	8.44069069259221
C	-1.09442078538413	9.85969316750608	20.91384964337649
O	-2.14221882695219	9.23482437837718	20.82769925451579
O	-0.87882159955120	10.82053008028789	21.84527794771139
C	-1.97145568596519	11.07352855199708	22.74513383403886
H	-1.62580430141891	11.86429115097126	23.42654271697958
H	-2.86494529951727	11.40759351196966	22.18926367039264
H	-2.22919235104235	10.16180615654952	23.31180915106616
C	-1.51273947381531	9.74943724097626	6.81241453821466
H	-2.39382715551334	9.10851554567637	6.96066732938204
H	-1.80776920577513	10.81326978393076	6.80084743759275
H	-1.01887643354604	9.51000629154000	5.85447794323977
C	14.03653227559086	5.77844118595621	7.51579535420684
H	14.23444113316238	6.62699841516326	6.84507318688337
H	14.95735956212380	5.48763591494318	8.05098986487991
H	13.67354942364620	4.90875164522734	6.94073180416025

C	12.46724438214079	6.90562137438200	24.42022603495139
H	13.47768335084168	6.59403172495114	24.10278599169794
H	12.51865834452981	7.83258005885940	25.00944306844074
H	12.01946920388115	6.09287364125071	25.01835715849004
H	5.46102449654895	6.48945142227931	15.03796901728686
Si	4.96809285196600	5.13898223984675	15.58536665804832
C	5.14948769676803	3.96823458305545	14.11775225291571
H	4.85893900553940	2.94458324264284	14.43020912340375
H	4.42064090661082	4.27304542982069	13.34174733133221
C	6.09273000366952	4.65803472324815	17.00690564350188
H	5.90333641612523	3.61666953525987	17.32644239449445
H	7.15271910846680	4.74257510729108	16.70920209560260
H	5.93550434950783	5.32153688130774	17.87478039426431
C	3.19082733274814	5.42312857921311	16.13632529173123
H	2.80446915481461	4.49744773166820	16.60863968635876
H	3.22899678397170	6.18469631054464	16.94005775438579
C	6.57761780672388	3.97886935506927	13.53875069491588
H	6.67407432590888	3.31678372950417	12.65851434327162
H	6.87206959681842	4.99598148000209	13.22228779808288
H	7.32044765927213	3.64458821273523	14.28620656981226
C	2.25962813020274	5.89127292393455	15.00384058262235
H	2.15194210679938	5.12056457541710	14.21893386742526
H	1.24453490332538	6.12618011034232	15.37500319035186
H	2.65089944294669	6.80301313827078	14.51893252473222
C	6.76580159715218	10.73405965904715	15.88909349595270
H	5.83146353603408	11.06934466058471	16.35529623627319
H	7.59221868810245	10.48124580272047	16.56443128397802
C	6.90632857313234	10.67563805734600	14.54013730587047
H	7.85475294630309	10.38289363770530	14.07359549683495
H	6.09605497646492	10.97552404965512	13.86448518868648

13 (dimer model)

O	6.64332000314853	8.55736600481065	12.11972600632506
O	-6.64331999020214	8.55736598482907	12.11972599581542

O	6.64331995762059	12.11972605052529	29.95463421932882
O	6.64331998556009	26.39227400431880	29.95463404212522
O	6.64332002682989	8.55736598309261	26.39227393220110
N	2.04330733693389	19.06950651443720	28.75560522436592
C	6.42195263999546	12.71755760237659	28.84168566615729
C	5.24911174462639	13.61738672448699	28.77196294750688
C	4.44522804099728	13.81712328104867	29.90955925120237
H	4.66946777261820	13.25961113543144	30.82530990275911
C	3.41165329889129	14.75269663942877	29.85779558339429
H	2.79601053291226	14.95972144222534	30.74119443526059
C	3.19441714903952	15.48988192397768	28.67511057821660
C	2.22304420121909	16.60501800126650	28.69991707607379
C	2.76282207225068	17.90106395412414	28.65186108748007
C	4.17855902738132	18.21000792059708	28.56504516293858
H	4.97937836365806	17.47429581313747	28.48164614784806
C	6.51328304568632	25.67663179394359	28.97900691861223
C	5.62818692492434	24.49090752396759	28.94525577982057
C	4.96271231135532	24.17190142682868	30.14415250448033
H	5.18121383713925	24.76648257386690	31.03805590849498
C	4.02519225397095	23.14186886831262	30.15643176517809
H	3.47627971109570	22.89092177043590	31.07173005270667
C	3.74921962627689	22.43631073667828	28.96754222275533
C	2.63989138014876	21.45833038223221	28.95509592912210
C	2.95013392835822	20.10522796133669	28.76678249803875
C	4.29371778371646	19.57690202945057	28.63530189479167
H	5.20464678714123	20.17759354403390	28.62038830917631
O	7.13627404467137	12.61267989400874	27.81336576529922
N	-0.16558243496395	17.19926841259342	28.94964123948691
C	4.99285977999614	14.30175620953461	27.56767766378468
H	5.62926395705133	14.09990438265070	26.69925853986276
C	3.96265107122128	15.24027776852080	27.51990081786105
H	3.76124413362638	15.80740972488758	26.60399524598097
C	0.86398407001868	16.29128068232986	28.83069881505708
C	0.32735335568798	14.94419893087282	28.79089336974893

H	0.92238151342483	14.03754414982552	28.66859591500630
O	7.13627401753351	25.89931998861222	27.81336595054914
N	0.17457120605320	21.26703934386741	29.10468312691841
C	5.39875726689268	23.75361298143177	27.76841357137445
H	5.93344963322554	24.01773467890973	26.85026345949946
C	4.45537138496026	22.72282812839953	27.78209947406843
H	4.22384398233540	22.16269102132143	26.86830062748892
C	1.34769174400448	21.99008102200491	29.10271486105381
C	1.04516887318357	23.41089680983924	29.12511235758620
H	1.78617695883608	24.21100490139236	29.10774428197471
O	-7.13627415340156	12.61268010119941	27.81336610377518
C	-6.54486338759740	12.82929877013117	28.89154863490106
C	-5.62681516216810	13.99085409910141	28.97329838167048
C	-5.45891376678525	14.81117615580622	27.84228701566244
H	-6.05289029187607	14.60178321862749	26.94576562903157
C	-4.51654567662328	15.83906833682365	27.87789743852931
H	-4.33551786445163	16.46789794330628	26.99824945755960
C	-3.74251880196863	16.03321770459473	29.04028597509569
C	-2.63356184504777	17.01098041093642	29.02142349614562
C	-1.33711122798856	16.47452762850622	28.98993817596428
C	-1.03695141720448	15.05654825321453	28.89098269352597
H	-1.78055643858209	14.25880298150648	28.86525426661081
O	-6.64331992517189	12.11972591159996	29.95463387844207
N	-2.04385790442419	19.40699617427033	28.92973051714807
C	-4.89846317972252	14.21808499329462	30.15562436457691
H	-5.07472119113018	13.56803914412020	31.01928154901423
C	-3.95111883221547	15.24161801348147	30.18736026029104
H	-3.34665424233558	15.42295259027345	31.08407346883257
C	-2.95284592011082	18.37304338227158	28.96215089962953
C	-4.29947708385195	18.90762899084785	28.94739274173388
H	-5.21120370694070	18.30851179596171	28.97453827484549
O	-7.13627400410220	25.89931999545903	27.81336599434226
C	-6.42203423576618	25.76631360817032	28.79002085024892
C	-5.24026197349798	24.87610902631329	28.84613605048930

C	-4.94215093364188	24.13694815493897	27.68635574271575
H	-5.54825860236299	24.30162139807629	26.78847236149881
C	-3.91740348857267	23.19298409988346	27.71941416672609
H	-3.68675658902616	22.58561362169864	26.83643867171417
C	-3.19248209771418	22.98784544968046	28.91174393844499
C	-2.22143759103214	21.87343647092226	28.97919544379013
C	-0.85916600624037	22.18047944344636	29.08985267338374
C	-0.32212759554370	23.52724375981908	29.10905366149836
H	-0.92074797557532	24.43913067844548	29.08010094967899
O	-6.64331999884045	26.39227400226115	29.95463400344306
C	-4.48211526191661	24.71786749826660	30.02222637987675
H	-4.72886678671991	25.31043798813776	30.90883429018793
C	-3.45379340023772	23.77296252485077	30.05197318940463
H	-2.87730180482000	23.60068059961337	30.96872645165461
C	-2.76380306554804	20.57936105345999	28.91463488036371
C	-4.18346324902204	20.27630703544918	28.90811439718065
H	-4.98416894879235	21.01707691976199	28.90295577301778
O	7.13627398164307	10.69863407157064	25.89932006935463
O	-6.64332007943922	8.55736611606052	26.39227405703932
O	-7.13627400767058	10.69863401807223	12.61268000146853
Rh	0.00361705315473	9.64791361080388	19.26384125114258
N	2.02288405136380	9.56535615627658	19.42318872622668
C	6.54432464955033	9.66631544915332	12.87146101706238
C	5.62196883488729	9.50234613378932	14.01260209343828
C	4.84473964841849	8.34076732096387	14.18450287850613
H	4.97350595389628	7.49675986493552	13.49922882349839
C	3.89243297286006	8.30316868246571	15.20525136092373
H	3.24693547098225	7.42732811521854	15.34050710197760
C	3.72681290750518	9.41648202286761	16.05301074756369
C	2.61706593847617	9.45418541529967	17.02988631636990
C	2.93596962448634	9.44397738589182	18.39083287263520
C	4.28283812190457	9.33543604205921	18.91808574254157
H	5.18345506119602	9.20138438804958	18.31590635648637
C	6.42129871985063	9.66550946800893	25.78698818715869

C	5.27001644946720	9.70813558368831	24.86924402591231
C	4.42929002613497	8.58327550336104	24.75217189789394
H	4.62491348541478	7.70503306417956	25.37673997032133
C	3.39722755675840	8.59773243491076	23.81477170522289
H	2.74681678347910	7.72616515979503	23.67651816241804
C	3.22317851874751	9.72386174904575	22.98416457101787
C	2.24065441645561	9.68077804826476	21.88109737331815
C	2.76833825005389	9.57667402287766	20.58774349998692
C	4.18060348299777	9.42634387251178	20.28377130086585
H	4.98267346219526	9.37628104037211	21.02214720407514
O	7.13627400052845	10.69863400287677	12.61268000047629
N	0.16277742180590	9.65501177251009	17.24337972622576
C	5.49484457899084	10.59753238402327	14.88879181211016
H	6.11968878386381	11.48117628074228	14.71727007539010
C	4.55427305485402	10.54991082889725	15.91518064876434
H	4.41079939979107	11.40219346398563	16.58968268435629
C	1.32705526910505	9.58788019338901	16.49941710535255
C	1.02740315796092	9.71524697785206	15.08391921975747
H	1.76765472457984	9.72476556812358	14.28227929580507
N	-0.15323226092285	9.64833674335506	21.28643168579070
C	5.06148530282797	10.86097165643870	24.08627296111216
H	5.72901216317828	11.71944174680385	24.21801634752563
C	4.03929110534987	10.86398541541062	23.13940866829493
H	3.87403966116260	11.73072374819475	22.48888802249513
C	0.88125318457896	9.70673505345235	22.20344792573087
C	0.35633276408635	9.78188861357181	23.55293025147638
H	0.96077590987171	9.86422633122183	24.45836299110817
C	-6.38385592200006	9.58955014375049	12.71193667949990
C	-5.24334266362953	9.75718471195472	13.63511171551202
C	-5.05565365312872	10.93510409287472	14.38404600849204
H	-5.72822071946590	11.78554681484912	14.23278047860546
C	-4.03442117241338	10.98347918507033	15.33526472862884
H	-3.88627766159640	11.87510917038621	15.95586822303189
C	-3.20199627035038	9.86324250892980	15.53190044901454

C	-2.22684893629070	9.83908033639546	16.64381736411106
C	-0.86573221216232	9.78687114411091	16.32684733162138
C	-0.33467250857321	9.84359568683094	14.97805010823604
H	-0.93158530600464	9.97479633792040	14.07355659579884
N	-2.01753995823037	9.72179811206737	19.10380382254859
C	-4.38135799732641	8.65439483011658	13.79094721372564
H	-4.56256989132835	7.75793405936573	13.18736462820110
C	-3.35609142083100	8.71129650852521	14.73259744016100
H	-2.69458019514214	7.85290398198700	14.89834485321170
C	-2.76083792575106	9.77565154531449	17.93849016508259
C	-4.17919150067092	9.70639135297617	18.24394943613066
H	-4.98253458644248	9.70046754624873	17.50555507444568
O	-7.13627385341568	10.69863386675975	25.89931997167880
C	-6.51751475416684	9.61482260433033	25.68053384522900
C	-5.58301142146468	9.57298192772725	24.54459870143958
C	-5.44572965280068	10.71360733711320	23.72687795322605
H	-6.04939746946276	11.60128025207049	23.94593555295634
C	-4.51738467911368	10.70189919984336	22.68771911989463
H	-4.36493019597811	11.58467975849568	22.05590276761632
C	-3.71411660837129	9.56135176708769	22.48075231364886
C	-2.61359110199482	9.60639462482826	21.49622263898758
C	-1.31829034103215	9.64562494888134	22.02971871854063
C	-1.01175806510627	9.73487996791233	23.44675355062401
H	-1.74890833808108	9.77746110807237	24.25026729390349
C	-4.82588409322891	8.40932734900522	24.30545508194703
H	-4.97420692544903	7.53561524381666	24.94895162160750
C	-3.88827151459226	8.40787108337967	23.27420252316134
H	-3.26190227988051	7.52909950568600	23.08194662153810
C	-2.93582139683861	9.64874350669310	20.13669342699087
C	-4.28673669212312	9.62203600942378	19.60975880605876
H	-5.19447542755474	9.53758003483114	20.21042898598337
C	-7.79093790671310	27.26021288477806	29.99677077554383
H	-7.69903868470383	28.06161840596832	29.24346905034101
H	-8.71486900275235	26.69056084540906	29.79633202139683

H	-7.81231015409236	27.68472106650601	31.01059968884187
C	7.99401097731249	27.05450057977560	27.76655475235797
H	8.41825538655933	27.07523094263007	26.75263396117669
H	7.41631069821008	27.97421431772653	27.96335363441593
H	8.79612902142899	26.97220089782864	28.52012397455491
C	-8.28355527396960	10.60522533398100	11.75202530996781
H	-7.97754618099149	10.36718641803045	10.71828872383267
H	-8.97246112299920	9.81770907175323	12.10456880224704
H	-8.77293897096281	11.58917157975259	11.79077355945711
C	7.50022259077231	8.65123912612933	10.96958789702873
H	7.46121629845680	7.66692615815492	10.48101354017802
H	7.14426700537673	9.43777797822649	10.28133209622624
H	8.53452550463437	8.89089133839589	11.27234393073146
Rh	0.00898282508638	19.22995960689867	29.05421797088169
H	0.02527328847026	19.32239652527769	27.26625057711860
Si	-0.47496417812365	19.32340106056041	25.79400675315852
C	1.08539109023324	18.87196207977324	24.85171078557195
H	0.89982752691080	19.05704531738261	23.77381355017194
H	1.87618085083063	19.58594452916435	25.15337447671559
C	-1.82432358958309	18.03702805296873	25.68130894420677
H	-2.08196969563075	17.84603959589520	24.62314200885418
H	-1.50193051181411	17.08394074409398	26.13480685159956
H	-2.73395903558445	18.37729556943065	26.20501465218457
C	-1.07569794557294	21.07793578682680	25.52804331554998
H	-1.57883688018956	21.13011038193774	24.54135970005199
H	-1.86569619033504	21.25270962479653	26.28321102326332
C	1.54386659947387	17.42206820469319	25.08686397701011
H	2.48896768858387	17.20301938985304	24.55749575092560
H	1.71215706778697	17.22553292866668	26.16018586349295
H	0.79142048423206	16.69387264915251	24.73564068801578
C	0.03038927244242	22.14117404610786	25.64006819741251
H	0.76087714112144	22.05356954443555	24.81593165188336
H	-0.38479114338487	23.16439930189547	25.60863417923916
H	0.58974264063808	22.04358489876766	26.58681530549484

C	-0.60382783830834	19.19129153503025	31.25219128505288
H	-1.08179910140324	20.16075181009984	31.43590838617340
H	-1.24263236650336	18.30576997802542	31.35193746677301
C	0.77654007503514	19.07532498141433	31.18963129693098
H	1.26571260455900	18.09544592141951	31.24081891849519
H	1.42391201202495	19.94965454687506	31.32658976450959
Zr	-10.03215402058725	8.14082035908506	30.98699590946298
O	-12.15144099212980	7.67601778123965	30.67571780718325
O	-8.53005748786102	7.83183869981202	32.81423168568547
O	-9.10174391514999	10.10012395570152	31.26689450205188
O	-7.90237031485177	8.05631664190268	30.17249399800914
C	-12.88021635347323	7.20945287408513	29.74435882203765
O	-12.70266157025375	7.36346917632556	28.50403774676961
Zr	-8.06871851181971	8.04531060376903	27.94134760949173
O	-6.70559468959406	6.56727219263435	28.41429375351308
O	-10.01801536029198	7.43946911792946	28.80054231795431
Zr	-7.20728367672790	10.19845684128367	30.62880142502337
O	-5.26888153638491	9.70910627627187	29.51422996339880
O	-7.98894285386917	10.00621297837454	28.67134308985219
Zr	-11.40396769530153	8.87223722446804	27.47144105049122
O	-13.22573350858523	9.95858622995586	28.37039815990834
O	-9.85025954234428	6.16447707017218	31.18173228028802
O	-10.65688665738077	9.74031354318709	29.44878411693191
O	-4.82441355144374	7.33017426241730	30.16562671220583
O	-6.27390701352608	8.86987919795020	31.99284799300418
O	-12.49669480817179	9.14709839336776	25.81924649516611
Zr	-10.56655998670849	11.65930777196647	30.83847908996935
Zr	-8.91619782177972	11.29956777777278	27.11632684976116
O	-7.04428398968108	11.47287543727625	32.45286565387176
O	-8.80259602749383	6.50931615762609	26.49958500837156
O	-11.03828491828956	8.77758331052547	32.83162241217851
O	-11.01641578515432	6.97140008330220	26.25473946663823
O	-9.43906721323346	9.17008385912841	26.81920217107385
O	-9.55481878743480	12.20023182034127	29.04563772543591

O	-11.08975962632597	11.06702968921585	27.08418486542464
C	-11.35967268613285	9.87766566418216	33.38960532269194
O	-11.25803597494602	11.02664155139268	32.88582481446185
O	-9.58110592615787	10.73097388442409	24.79464446461633
O	-9.19346579521176	13.09575050274933	26.27558179387017
O	-8.95255911112949	12.66767011410038	32.17078390328854
C	-7.88170760965760	12.37819880660194	32.77061273127855
O	-11.10763635839060	13.57250484152691	31.00130362892267
O	-12.53180800955565	11.34295001692848	30.37526128030019
C	-9.95416575008377	6.34431315358630	25.98018693583313
H	-11.45180000927326	11.19476142769924	26.18737382206156
H	-7.46581990669677	7.23695323638259	30.47011305846890
H	-9.69509735460170	13.14859840064155	28.86683365481884
H	-10.19269085468735	6.47952350095493	28.78394251409203
H	-9.90535057300898	13.21311935822677	25.62439427166142
H	-10.50097431162267	14.14146574899532	31.50689983964437
H	-6.41074350522130	5.92102164802612	27.75072647410193
H	-9.27676329787667	5.81873641850476	31.88682576563995
H	-5.75923985995230	9.30410931727951	32.69707738032307
H	-8.87449455696676	8.35675487164980	33.55884792085409
H	-7.60718796970071	8.21749310131181	32.59889278652319
H	-5.19931193617151	9.91484961348907	28.56574045500370
H	-4.94190032493221	8.72446087329750	29.67755510934473
H	-5.30139105688288	7.58007936664115	30.99753398928367
H	-5.50649041090698	6.90336576775776	29.56002201253833
H	-12.58775299212903	8.37668711680460	25.23216504308204
H	-9.61514986148890	9.79117931493313	25.11268254644987
H	-8.76586029748798	10.79466392483596	24.26297361024312
H	-13.15742877765304	12.01989115327096	30.68573644388634
H	-9.70391230534292	9.90612253198501	29.20289295944449
H	-12.96464057026307	10.51929058538018	29.20214604529810
H	-13.44812324900550	10.61942104157581	27.69021203698529
H	-13.76134953022819	6.60741779091352	30.06034061675433
H	-10.02989274754963	5.54767341460876	25.20437677495151

H	-11.76610502193643	9.80236098500739	34.42296134495979
H	-7.62727878708054	12.97762494547475	33.67608544017246
Zr	10.23584919570252	7.66452057816995	30.27153437416949
O	12.34892262182640	8.07739878976869	30.64727641183203
O	8.82236269672414	5.79069926239620	30.68860041941770
O	9.21888273998653	7.31945681139951	28.36706174711487
O	8.09138168510314	8.40641185231103	30.44597166179454
C	13.07027376067147	9.04048482311041	31.05478809620718
O	12.85878728397254	10.26987093251753	30.85767298984271
Zr	8.17453749079706	10.65145232817771	30.40541901010569
O	6.93171155398853	10.14500258718255	31.96841879301941
O	10.18136187498216	9.88952421076490	30.91121311359113
Zr	7.30145243334069	7.88775817701420	28.32366860445801
O	5.30776652840988	8.85872468198636	28.88174112742596
O	7.96776999456551	9.87822992580866	28.49386535445533
Zr	11.47847620093771	11.22721493983145	29.38151269097580
O	13.26932577342219	10.31499684595502	28.24397972223327
O	10.14679883575064	7.50932030886265	32.25477593530150
O	10.70430585386843	9.21035337940061	28.58581869179047
O	5.08732389053298	8.28545362057079	31.31635549444894
O	6.51319910015283	6.49146608772040	29.70464308312627
O	12.49294977835853	12.89395603653011	29.01443188780399
Zr	10.64644549356160	7.73885486441304	26.76357474632243
Zr	8.90556976973610	11.35678730506165	27.11487452235828
O	7.15566639525590	6.08764060267680	27.03865343332930
O	8.92478386935035	12.19521963686068	31.81631947616688
O	11.27981542283175	5.86828618605647	29.59170057796449
O	11.10617845152489	12.48860314315478	31.24978458963529
O	9.46035982722576	11.79227448385960	29.12194637391595
O	9.53780744015032	9.48050299956387	26.14873425498734
O	11.08349567131814	11.46517874118131	27.20358591253144
C	11.47698416582210	5.22389542671839	28.50783531456894
O	11.25370592015735	5.64031135248706	27.34180893608586
O	9.36512206855246	14.50854514723672	28.46843338859774

O	9.08580823788417	12.96577086694398	25.99082455655912
O	8.98751439695676	6.47435325835831	25.76069663249626
C	7.95340624890843	5.82666527353390	26.08001592747527
O	11.25858604319151	7.49478740125777	24.89157409000661
O	12.60772817554982	8.08693075671076	27.22116262131266
C	10.06581168433120	12.75538494430569	31.91323524837082
H	11.36154323424296	12.35975541109996	26.93322714612626
H	7.71997436427145	8.11866216044233	31.30003548083341
H	9.14205434698183	9.35401252728684	25.26802758884888
H	10.41280697528904	9.93931591869251	31.85806613809406
H	9.15022871224699	13.79940644856012	26.51043328298634
H	10.70947024129400	6.97431980155956	24.28033471476530
H	6.63868284349175	10.80544511815851	32.61869852152987
H	9.60404860809580	6.80408398045621	32.64678257402306
H	5.99713844279517	5.76421525162239	29.31174875819752
H	9.17089047337277	5.03180206797730	30.18800795403512
H	7.88027087386481	5.96006805162271	30.32273475652091
H	5.12423732832830	9.78377063257506	28.64400505186367
H	5.07394096403784	8.71821294262269	29.89463326959026
H	5.58398268467726	7.46761771185421	31.06100469820172
H	5.76123184429232	8.93132032458509	31.68981338661182
H	12.54448907309767	13.56249535519456	29.71888233875604
H	9.43225314713259	13.60739026835590	28.87247920383414
H	8.42476688661588	14.72829169097873	28.57695632717810
H	13.26798813650684	7.65996437838498	26.64828701403829
H	9.72728457771525	9.37913028440147	28.50372671334749
H	13.04429718084580	9.37668197889855	27.87127608188655
H	13.26724738569738	10.86729687766042	27.43912620626969
H	13.97653262621431	8.76883227279439	31.64040791732587
H	10.15102882546302	13.56101895516131	32.67804611667586
H	11.88500696610748	4.19431197936956	28.61843107473088
H	7.69645410191389	4.93480585579509	25.46325837817402

Rh(III)-**14**

Rh	6.06208819241752	8.59985776817458	14.95766393300307
N	6.59369502349025	8.48262557389714	12.99917718130486
N	7.97432248329289	8.13649728290046	15.50060585523785
N	5.59169406478109	8.93407193153796	16.90685588875400
N	4.17870479609198	9.16007734593720	14.41143775964576
C	5.78291660531051	8.73440558205499	11.90050549577937
C	6.56573332950562	8.64534974495389	10.68307652685031
H	6.18606376016903	8.84122471315058	9.67906863026366
C	7.84103174358809	8.29419381946429	11.05066230155370
H	8.70937498416020	8.14900351736154	10.40607882996293
C	7.84816279715743	8.16978191921623	12.49539133198497
C	8.96504645635330	7.76767335064144	13.25071763182874
C	8.99934978577460	7.73156793726561	14.65626257926489
C	10.12778449639704	7.26966166477432	15.44103551570811
H	11.05545508425482	6.86884458817494	15.02932849711789
C	9.79114714088790	7.43045355659927	16.76260474348702
H	10.38964706108807	7.18606935165117	17.64161850434179
C	8.45419551976156	7.99106332638491	16.79527348440440
C	7.76059865080162	8.33329373729030	17.97006693062120
C	6.44067181383923	8.81906875238279	17.99846573856885
C	5.75060834640560	9.24013039796724	19.20219299441281
H	6.20009371402475	9.29271956084838	20.19509042996757
C	4.46779912338532	9.56634481834978	18.83737558629286
H	3.66297280602839	9.93851932130786	19.47320739396144
C	4.36475004772397	9.34742917196764	17.40750871487448
C	3.18228703953231	9.50728214555202	16.66246833239156
C	3.10818907970181	9.39433880043143	15.26248929117284
C	1.88436329992691	9.50871869474296	14.49295625850528
H	0.89029749423689	9.66483747467943	14.91476900209572
C	2.22716864428731	9.37123931361492	13.17075125481346
H	1.56822387122401	9.39339128544562	12.30130207418191
C	3.66216289162101	9.16826200090317	13.12366749527588
C	4.40286254291830	9.00238638375671	11.93976136119281
C	10.18513702914214	7.33442898007397	12.50908158150690

C	11.39450843297772	8.05762659502439	12.61789577470141
H	11.42832282024432	8.95540493798259	13.24673694715097
C	12.53410090105277	7.64480229483107	11.91868557869835
H	13.47468030598468	8.20331713654305	11.98943864586959
C	12.49024192449802	6.49726630153383	11.09946454483333
C	11.28658231660355	5.76892904695302	10.98731210392343
H	11.25336831039691	4.87498223151341	10.35574164412930
C	10.14604555716610	6.18733271722427	11.68420196763046
H	9.21145057835064	5.61946258189711	11.60272970384177
C	8.45303711068445	8.13209562096388	19.27670670680037
C	9.60592764209660	8.87637320778211	19.61250227694377
H	9.98718180889712	9.62227330967477	18.90472125610604
C	10.24998846461270	8.67974751767913	20.84086165648032
H	11.13809836299991	9.26522919519154	21.10079156721149
C	9.75279685581630	7.72738271419476	21.75601647891136
C	8.60359825175619	6.97930330507484	21.42472910705489
H	8.23351138062593	6.23932310715876	22.14385640753704
C	7.95910490599076	7.18163956290305	20.19964191897782
H	7.06960959973123	6.59550493520694	19.93972835392463
C	1.92545925302707	9.79990410314144	17.41213620160118
C	1.22771217722219	11.01372042711158	17.21860395209668
H	1.62697552567421	11.75632551344156	16.51749072434222
C	0.04748167406574	11.27434103002007	17.92385185233888
H	-0.49806632533476	12.21532986482355	17.78734812753295
C	-0.46204448859518	10.32569340606217	18.83519164953151
C	0.22925552740195	9.11151773454661	19.03409808304879
H	-0.16867994274047	8.37265727208391	19.73749409611243
C	1.41226373077846	8.85510674877872	18.32999787655871
H	1.94703538935453	7.90901499750677	18.47685122738539
C	3.67914059774208	9.10191273024418	10.63825037897004
C	3.07875086547035	10.31587155316486	10.23538618807355
H	3.15491867612163	11.19313544314405	10.88897826626778
C	2.40878124803125	10.40908676713393	9.00887214091754
H	1.95385375350540	11.35470039136458	8.69603587085131

C	2.32345775217800	9.28296750535816	8.16259148211075
C	2.91615804827828	8.06669043747916	8.56207385144521
H	2.83473202482162	7.19998029846643	7.89586784674024
C	3.58872498103878	7.97766157922442	9.78578016909985
H	4.04416135772159	7.03056752290219	10.09901552096253
Si	5.47981308440231	6.33616866363426	15.08581467957309
C	5.82418635761049	5.61721208570471	16.80984863632965
H	5.97668843688369	4.53200051232036	16.63397011880760
H	6.79714655266551	5.99455426883026	17.17429335662895
C	3.64843211599185	6.11733379002432	14.70013809067544
H	3.39724136301805	5.03991783159371	14.74375232594417
H	3.01410285459804	6.64881748694451	15.42962165961572
H	3.39774717815296	6.49141520825464	13.69316917327405
C	6.48745358025659	5.29121679327195	13.86092869738354
H	7.54176185590712	5.62345108260490	13.88014818135109
H	6.48989942307128	4.27239754389973	14.30126240741982
C	4.73332172555357	5.81415491254269	17.87346753027927
H	3.77625491319666	5.35911782853119	17.55965510673790
H	5.01553229146857	5.34880305895920	18.83703503378310
H	4.53543682193516	6.88046252305300	18.07185606586464
C	5.96747030133935	5.22436708458792	12.41717464033862
H	6.59661865045521	4.56664067631201	11.78804373601504
H	4.93656899196345	4.82847209067446	12.37620954167052
H	5.95397845671948	6.21715312311520	11.93777229097056
C	1.62704826356160	9.32329979961508	6.84184520495057
O	1.53326130966514	8.37368455822567	6.07703475580746
O	1.10244506229240	10.54399197656200	6.57605033298490
C	10.39716057530690	7.47295013898593	23.07924558148263
O	10.00180694326574	6.65506687568295	23.89800636237428
O	11.48329054543752	8.25770522481010	23.28023240969166
C	13.73740312445362	6.10423611123292	10.37777192438690
O	14.79808471369545	6.70927807902723	10.44500562389891
O	13.56743201907252	4.98730867737191	9.62976101222638
C	-1.72722962770118	10.65604997336166	19.55697446853257

O	-2.35870098759611	11.69328916220857	19.41213753549018
O	-2.10749464295432	9.66835129355169	20.40311523595694
C	-3.32020282833768	9.90895272013637	21.13767790435771
H	-4.17420276675653	10.03421976806443	20.44923887995653
H	-3.46819753323873	9.02565753479296	21.77556850252204
H	-3.22790828856756	10.81971300296494	21.75462600847574
C	0.41971641074448	10.66885193459387	5.31664321299928
H	1.10353279633869	10.44639319380865	4.47898858916379
H	-0.43690943783384	9.97415547387588	5.26656020914004
H	0.07228878003661	11.71069238387491	5.26267456959618
C	14.72521038719869	4.54454264862542	8.90029216020582
H	15.05405185296038	5.31892374811761	8.18541592280601
H	15.55889433742898	4.32384711165965	9.58941402651616
H	14.41664971950762	3.63464775351706	8.36564871339487
C	12.16106379822507	8.07446986205816	24.53530692213140
H	12.99887849837323	8.78654458672293	24.53388782008084
H	11.48046897014241	8.28118842900789	25.37958297296239
H	12.53490444686129	7.03986503936096	24.62875323547808

9

Rh	6.04012310623291	8.48679771257014	14.95630934812325
N	6.57653200564134	8.40984288501367	12.98725130489767
N	7.97142706632825	8.09005198202369	15.50705087620136
N	5.54998975150582	8.78080119115199	16.92249373855588
N	4.14780004291227	9.05723067215067	14.40781319719367
C	5.76514016940780	8.65473414576510	11.89164563293594
C	6.54373033270467	8.53193059036225	10.67406526554696
H	6.16529289578569	8.71689126957974	9.66747910733623
C	7.81355029797864	8.16302742148588	11.04408993516157
H	8.67664698694510	7.98966513058856	10.39935268247201
C	7.82340823297145	8.06713994142335	12.49083627835089
C	8.94383526324938	7.68525295699964	13.25324929352197
C	8.99169254634894	7.68830696218679	14.65998732708679
C	10.13721202027087	7.26792203023503	15.44243467778049

H	11.07059386325526	6.88205624728798	15.02947418422239
C	9.80466361090741	7.43610101043241	16.76412680003286
H	10.41214153128803	7.21156946882992	17.64227300440437
C	8.45039985690179	7.95362860672069	16.79956409434726
C	7.74153440815420	8.24866799123419	17.97994243402540
C	6.39674205272615	8.66431950644113	18.01430640908530
C	5.68622437168350	9.03712651453774	19.22205753264267
H	6.12649743663690	9.07984732262510	20.21952381289225
C	4.39821270609091	9.33917171435940	18.85482041836802
H	3.58061954129542	9.67782511867683	19.49315582927373
C	4.31176218354043	9.15788308111098	17.41885552276869
C	3.13946828570572	9.35440230866477	16.66394247811160
C	3.07719929299660	9.28371908991948	15.25915330420544
C	1.86528672551098	9.45537614654119	14.48233472193306
H	0.87125307104260	9.62192437098472	14.90037126315534
C	2.21753589144330	9.35385172381762	13.15890366032736
H	1.56716166756349	9.41977787469335	12.28520418995679
C	3.64643318709743	9.11359237803747	13.11622106427069
C	4.39091475302848	8.95833099491830	11.93154056822029
C	10.16822257560658	7.25707840536677	12.51404936715671
C	11.35584157713318	8.02021186572503	12.58235664471468
H	11.37021036145648	8.93963065709107	13.17992500484054
C	12.49873533558513	7.61824827637448	11.88215684047651
H	13.42263441486155	8.20686849390863	11.92161731401381
C	12.47943232210078	6.44266354271314	11.10292298205356
C	11.29763481642400	5.67468147458926	11.03228009742143
H	11.28392435396087	4.75871788209911	10.43234979521407
C	10.15336526241308	6.08151495805112	11.73023657420998
H	9.23637191468283	5.48193129842375	11.68298049693976
C	8.45864117429464	8.10268631927439	19.28099451197890
C	9.57088185148314	8.92020083154508	19.58457609208915
H	9.89838853971221	9.67094171378926	18.85537824145884
C	10.24076763425422	8.79010425512270	20.80768116089619
H	11.09629385360838	9.43212571250780	21.04196331627013

C	9.81084283435113	7.83253902025066	21.75088503547143
C	8.70393999423484	7.01085012903476	21.45178173968108
H	8.38759574801496	6.26723078432405	22.19241332035247
C	8.03376369261907	7.14573448985043	20.23101560303698
H	7.17905258611493	6.50033081903887	19.99598898343525
C	1.88351297153580	9.67463773879685	17.40542672975981
C	1.24972989255697	10.92746130491717	17.24101644901003
H	1.69379086205119	11.66916919549071	16.56635038426835
C	0.07711584233117	11.22753048566429	17.94309153705993
H	-0.41834738292068	12.19877034545279	17.82955566918320
C	-0.48844522097043	10.28024765580360	18.82201768621151
C	0.13746754507169	9.02676618173566	18.98978570810986
H	-0.30491606461586	8.28911546148646	19.66739081599752
C	1.31315320489441	8.73026472314406	18.28869582448806
H	1.79585924604594	7.75338841279384	18.41221153898320
C	3.67489246388638	9.11614558180664	10.63080675874963
C	3.12732487380949	10.36475927220118	10.25916882511520
H	3.23982200934775	11.22133148798894	10.93463167918051
C	2.46159970955393	10.51742529097979	9.03626929134287
H	2.04649383350519	11.48905496334217	8.74877887563732
C	2.32810345580438	9.41796036196797	8.16184808985546
C	2.86919273529877	8.16823204920292	8.52929896613647
H	2.75084462360607	7.32268547023249	7.84181260691532
C	3.53686012853514	8.01913265184671	9.74997610843032
H	3.94936804477656	7.04478141982397	10.03800082284632
C	1.63057876872492	9.51995914856116	6.84474081454165
O	1.49858904427570	8.59479660034359	6.05615978325855
O	1.15289566733039	10.76637129788572	6.61305244507995
C	10.48456542552060	7.64778097421055	23.07130155191491
O	10.14367740561953	6.83188830768389	23.91596278481234
O	11.52943465333967	8.49420529366883	23.23665792894327
C	13.72913611637049	6.06253657043677	10.37809487998894
O	14.77185166668845	6.70055706422857	10.41181200215163
O	13.58244918324426	4.91822428632957	9.66807247514039

C	-1.74067838210994	10.65519189233103	19.54523012997225
O	-2.31483376800529	11.72844321101820	19.42763146220588
O	-2.17833395609901	9.66470947203648	20.35926955810546
C	-3.38118381075566	9.94787284095401	21.09524446878245
H	-4.22139975923944	10.14594264917637	20.40706047942776
H	-3.58361209920089	9.05250222602137	21.70047556451494
H	-3.24164997893513	10.82944336920374	21.74486899662274
C	0.46992898858947	10.94964486036698	5.36068700214082
H	1.14420721776997	10.73268429746765	4.51388995561895
H	-0.40684774573940	10.28219199209778	5.29340243673713
H	0.15357717834414	12.00248529921867	5.33968951447283
C	14.74343409530006	4.48526227106649	8.93759982193040
H	15.03956575481246	5.24450360667984	8.19283538285841
H	15.59214471679954	4.31239515083589	9.62197623708746
H	14.45505843795734	3.54926596366023	8.43791323540391
C	12.23177783846141	8.38028418278163	24.48659973235577
H	13.03445563576826	9.13095191125623	24.45228420995801
H	11.55325284885787	8.57965480732227	25.33430199411411
H	12.65551667513385	7.36768682596313	24.60434394912086
O	5.54795152480664	6.56291410999344	15.00756373480294
O	6.32241828420184	5.73533053207113	14.45412401153388

Rh-OH_2

Rh	5.84467874490232	8.24348386364433	15.08293557658292
N	6.18303957931436	8.08814762419386	13.06983636319168
N	7.72800199400046	7.53072486673006	15.45332078115780
N	5.54573349546621	8.49026594097215	17.08700062563856
N	3.97989868413827	8.99087624923595	14.70981826025294
C	5.31753792003177	8.46356931086388	12.05210291491169
C	5.98006680679445	8.31737724065200	10.76985034123921
H	5.54300932916347	8.57936212865985	9.80484631715611
C	7.23586916031224	7.82039570615187	11.01919870679930
H	8.02358256506770	7.59879799527525	10.29722892188301
C	7.35263942326208	7.66036863366368	12.45643673121424

C	8.48964752429052	7.14789612272073	13.11231719916808
C	8.63632459235798	7.06707543560720	14.51175135694730
C	9.78040361119560	6.48189717266761	15.18516386351954
H	10.62991347332986	6.01314101808662	14.68566709606460
C	9.56837529697080	6.62160142353125	16.53508707165917
H	10.21137967336380	6.28790618646051	17.35128888815970
C	8.29283105221696	7.29321543726572	16.69842189116285
C	7.72743738240284	7.64490958264491	17.94065136245594
C	6.45782134615982	8.23424192948581	18.10012299195423
C	5.90609711647923	8.66076814049152	19.37351766771746
H	6.42861645457712	8.61514331824309	20.33038664897023
C	4.64432643572430	9.14002812190291	19.12431509637487
H	3.93412333441190	9.56175252193284	19.83755049825537
C	4.41572465118407	9.01281662202637	17.69649167979229
C	3.21890842151176	9.37459198952301	17.04794629289879
C	3.02435569387325	9.33395434700948	15.65355852048416
C	1.77424004570523	9.65600215734498	14.98974102436124
H	0.84705326397785	9.92892283749976	15.49632176548688
C	1.99198456979047	9.53089674305613	13.64035182315943
H	1.27712583699419	9.68055266968073	12.82956921405963
C	3.37683968818368	9.13062737667685	13.46876807849889
C	3.99458741816190	8.91889607014702	12.22036918667467
C	9.61988022517372	6.66388272161086	12.26505722753083
C	10.88034063514390	7.30202223820559	12.31535986345780
H	11.01633114779072	8.16368619544784	12.97983919760532
C	11.94166183277397	6.85570295613815	11.51795061773679
H	12.91317428081774	7.35949506892110	11.55514007242729
C	11.76264476792986	5.75460840751254	10.65344536217441
C	10.50839489649048	5.11132659233469	10.59994168352545
H	10.38674717304308	4.25390745129958	9.92786440621125
C	9.44915306290501	5.56240896272942	11.39513083699430
H	8.47713489371045	5.05625189654327	11.35566635582402
C	8.52925045317510	7.37409053091454	19.17047928112732
C	9.76728181631245	8.02450795558905	19.38026088856218

H	10.12789814453047	8.74233370258068	18.63384812312609
C	10.51905408940151	7.77009936551282	20.53256203556440
H	11.47603645000863	8.27557123500657	20.70679272965087
C	10.05132187236787	6.85700395553937	21.50081243342090
C	8.81703233990343	6.20307897840561	21.29920566107740
H	8.45654900235238	5.48977248533300	22.04776949351423
C	8.06563241156733	6.46218956240550	20.14611206446119
H	7.11110825718662	5.94659588238735	19.98583881866213
C	2.07859245572551	9.82992236959172	17.89753186913688
C	1.58370967750028	11.15041731462816	17.79644051741862
H	2.05589320011354	11.84672712340267	17.09313638367440
C	0.51327375891100	11.57181488407416	18.59316526056244
H	0.12698091852844	12.59563269605835	18.52744103092018
C	-0.08872742343194	10.68071607373640	19.50618933365797
C	0.39949131047503	9.36085842092990	19.61317118033189
H	-0.07123908471951	8.66726776788438	20.31771196788467
C	1.47359829939055	8.94347163780446	18.81731830397485
H	1.84789470751275	7.91549748853626	18.89301605520751
C	3.19490236370680	9.19754945270395	10.99059624898465
C	2.74711991107840	10.50776575963755	10.70586936058848
H	3.00340228169396	11.32144999853569	11.39486505080921
C	1.99788966167055	10.77392126325210	9.55296377829388
H	1.65989945720239	11.79216236708757	9.33387314657718
C	1.67861749293448	9.72862332157047	8.66013581311644
C	2.12181496993122	8.41860083230013	8.93814118578276
H	1.86073075715867	7.61701849525093	8.23741286677592
C	2.87262921970744	8.15674625233896	10.08941345022198
H	3.20811640569423	7.13591868977944	10.30806561811316
C	0.88182875218146	9.95022026856613	7.41672462124702
O	0.58796686600824	9.07471585049877	6.61489906804974
O	0.51237952344739	11.24552121772420	7.26837724394572
C	10.89595758084432	6.62199713838199	22.70967556864181
O	11.97233362005937	7.16268581037092	22.92098991714235
O	10.33425746307129	5.73094019047647	23.56254503713658

C	12.85550552783442	5.23389355067753	9.77890308138391
O	12.74259328829017	4.28213585354998	9.01918220950717
O	14.00428103490956	5.93867577629324	9.91880027194216
C	-1.23114239141846	11.18259892847067	20.32645871615943
O	-1.68439555542880	12.31688406677404	20.26621120166577
O	-1.72233542246162	10.23176164981113	21.15822610220108
C	-2.83005896899922	10.63566970947961	21.98122265710612
H	-2.54169745672885	11.47341113652636	22.63999852826467
H	-3.68293680398626	10.95542989840051	21.35737343419923
H	-3.10089700199147	9.75332631872872	22.57890103527984
C	-0.25815910102807	11.54328481992060	6.09130181145518
H	-0.47504129606218	12.62032276574745	6.13585171595956
H	0.31539770602879	11.30325491700006	5.17897909523511
H	-1.19603521844035	10.96099163164641	6.08153990015748
C	15.10706014746177	5.50134464206257	9.10582475197483
H	14.84706968891703	5.55616320550921	8.03432277789404
H	15.94013672821763	6.18153293142547	9.33456980842196
H	15.38205846638300	4.46014652659427	9.34914744315249
C	11.08829381561538	5.44823859901242	24.75390941790750
H	12.07879339434275	5.03265465613418	24.49895407547121
H	11.23408970642788	6.36600976157762	25.35001505526058
H	10.49714562511354	4.71333987734132	25.31916822728995
O	5.10850889772593	5.96434994999086	15.04443670425649
H	5.95012668333822	5.54901118129947	15.31387828471282
H	5.09874702945643	5.86550851846838	14.07308954670915

Silane-Rh-O-O·

Rh	6.01109748213143	8.16196548560099	15.15658533664774
N	6.04312467796927	8.09361666127120	13.10967046225478
N	7.96901228464226	7.56525212030710	15.21711710805173
N	5.98276122376550	8.27019274696894	17.21029170259622
N	4.04813387130656	8.76189998026801	15.10826716397520
C	5.00959196054902	8.44263602290029	12.26085877040076
C	5.48157134680436	8.41403117791029	10.88834708755970

H	4.88249892018699	8.67453352884745	10.01433940688248
C	6.79805288782141	8.02529486232518	10.92415285184996
H	7.48505562716095	7.90781924715708	10.08471188101407
C	7.14019076081028	7.81003184620302	12.31826859065904
C	8.40797968265763	7.39690963616874	12.77337004345819
C	8.76104379518545	7.25113515140611	14.12908366642720
C	10.03230978891614	6.72555159703447	14.59183321230810
H	10.83478876404736	6.36619548661361	13.94571058925056
C	9.99800609203504	6.74059484638068	15.96431109115372
H	10.76695957620846	6.39740600873373	16.65813871502474
C	8.70820115173062	7.28293506094316	16.35132439697614
C	8.30115751525852	7.49556629691550	17.68260233508568
C	7.03861857575137	7.99449445745355	18.05952458141644
C	6.67365660902980	8.35418081889764	19.41703297682961
H	7.33006879178299	8.27292188224633	20.28481752053167
C	5.38494406466549	8.82840061480501	19.37528519876952
H	4.78366676434646	9.20730700679346	20.20311675243345
C	4.94680395497069	8.74732466714395	17.99444239408077
C	3.65861213692309	9.09837965637806	17.54330109930661
C	3.24703145565842	9.05231797337678	16.19676624578719
C	1.89695815416399	9.33102262667655	15.74350801199140
H	1.05098835865667	9.55511574784566	16.39518586025560
C	1.90443932120126	9.23254583205629	14.37392754743512
H	1.06590764913841	9.36182811294957	13.68793642019782
C	3.26138835171111	8.90136491170815	13.97944732617193
C	3.69927321394200	8.78967658038469	12.64498229494611
C	9.45074060311212	7.09737142278477	11.74963324731087
C	10.63144005385897	7.87238637717345	11.68257081070303
H	10.76880593696715	8.70133456003508	12.38690149829479
C	11.60823774827375	7.59738558956731	10.71941559881699
H	12.52332457632407	8.19720053838925	10.65281930288173
C	11.42816060841305	6.53787061216253	9.80551307468338
C	10.25438182994513	5.75681044260299	9.86944050293196
H	10.11799204563148	4.92865740174318	9.16622181148281

C	9.27620935758862	6.03682348604368	10.83180082224295
H	8.36885851079910	5.42382791186498	10.89018055624065
C	9.28945195764272	7.22532468166029	18.76696879880514
C	10.48840907719566	7.97094188804446	18.84495501194362
H	10.68198900675751	8.75346334660764	18.10173686978316
C	11.41106738475281	7.72891346288196	19.86828118848387
H	12.33953594346659	8.30682236352641	19.94274645801888
C	11.15700815314618	6.73323836800574	20.83490663989151
C	9.96332740752478	5.98373433680261	20.76317322062150
H	9.76843050606059	5.20625973496724	21.50916147897568
C	9.03951096959439	6.23099392208136	19.73985987050247
H	8.11523486126110	5.64416863370374	19.67797173953712
C	2.67222512801736	9.57981540186491	18.55409753462675
C	2.18161591249679	10.90465859363227	18.49044469022614
H	2.53438859767294	11.56827674938764	17.69216002027783
C	1.26897691984882	11.37221504038837	19.44186481011880
H	0.89014196315787	12.40022649334508	19.40646670737492
C	0.82276987721554	10.52335258649085	20.47643838161597
C	1.30185307324352	9.19752170855763	20.54271655666559
H	0.94829151077271	8.53570898726217	21.34024159924348
C	2.21821239270027	8.73311205385288	19.59054997111384
H	2.58275618647678	7.69989765463163	19.63576943840526
C	2.71176049031012	9.07931924920932	11.56378197493598
C	2.16364142162345	10.37483685760216	11.42126638859893
H	2.47978691397793	11.16816948496150	12.10914565888388
C	1.23969791114532	10.64811690049834	10.40676319214889
H	0.81388439068197	11.65064619251143	10.28330385881052
C	0.84119687365551	9.63071481699075	9.51442340027977
C	1.38343702291278	8.33495951895852	9.65075675300114
H	1.06916964473852	7.54363091492655	8.96225742019377
C	2.31099195523205	8.06515197296197	10.66500438152535
H	2.72553381841224	7.05625120659091	10.77812751779734
C	-0.15127759593916	9.97815143900314	8.45374198593546
O	-0.64594389578248	11.08626766199757	8.30224075792331

O	-0.45096378381435	8.91827376578555	7.66461425415392
C	12.17732536380239	6.51887505881890	21.90426378876800
O	13.22185463385692	7.14734843147401	22.00275981787775
O	11.81752721096308	5.53898809923401	22.76815764518045
C	12.50391998091351	6.28935160998688	8.79987969421781
O	13.53440606158562	6.94185706777205	8.70986777574546
O	12.21267824645947	5.24753251599693	7.98375483792213
C	-0.14750378512342	11.07745525735683	21.46755875971249
O	-0.58918600526943	12.21763936281572	21.44332209471251
O	-0.48997988913678	10.16955747378441	22.41319271385192
C	-1.41882563437389	10.62908704141573	23.41055546878165
H	-1.00465152731661	11.49277694857811	23.95934627676423
H	-2.37292198851474	10.93165124155674	22.94476479386526
H	-1.57635664097655	9.78036726929081	24.09159451904256
C	-1.40564034180813	9.16957084739978	6.61889054090422
H	-2.36904385682923	9.50278778194216	7.04269082089789
H	-1.03452433361380	9.94924846135412	5.93096499713078
H	-1.52981688324790	8.21544721236844	6.08669323119850
C	13.20144887697864	4.94284922291872	6.98497510839915
H	13.35861237415333	5.80680015451449	6.31590857454676
H	14.16487118477790	4.68240136862702	7.45697531334183
H	12.80683208312514	4.08610195892127	6.41991797510026
C	12.74951398279538	5.27203197957872	23.83054420722307
H	12.89308098430593	6.16943526850347	24.45737231135261
H	12.30850088249982	4.45695256210017	24.42227251345097
H	13.72867751413293	4.96643966382518	23.42247253639065
H	6.49340848706496	9.81860124214596	14.87903216568005
Si	6.95691729059045	11.16372288757298	15.48686649039778
C	5.40783790713444	11.98547965907563	16.14603722952751
H	5.01550779432699	11.32483311684040	16.94273252744063
H	5.70902920259876	12.92560420612605	16.65117342089204
C	8.26060834305495	10.87870587284640	16.79195280775025
H	8.99813257949329	10.12904035700792	16.45932261513435
H	8.79336028797341	11.82743858788849	16.98901214770202

H	7.81605338561293	10.52622377872442	17.73754195787035
C	7.66266816649524	11.95008170453504	13.93151502563180
H	6.83470637714975	12.09510261852245	13.21125943111813
H	8.02213369734556	12.96538229125056	14.19737831304886
C	4.33151316882175	12.25473016503519	15.08041126772735
H	3.39817104254532	12.63708829402693	15.53225051399262
H	4.67026399245400	13.00141655544462	14.33995308350324
H	4.07443581666403	11.33347111155059	14.52908059451665
C	8.79062887728431	11.11764473138612	13.29442999783321
H	9.64541791196578	10.99351187080895	13.98387892307955
H	8.43710442399900	10.10615645253702	13.02735154517311
H	9.17491057910595	11.58830903292002	12.37130043010720
O	5.39035720036426	6.23815903334966	15.27343321807372
O	5.97769397615412	5.39588419000465	14.51622128054660

Silane-Rh-OH_2

Rh	5.87275493407886	8.00292190301732	15.05297878915641
N	6.20460581732505	7.81788828786218	13.03837080698170
N	7.71521038896637	7.19417886171843	15.42992693863156
N	5.54529131056344	8.18904150282792	17.06708246237941
N	3.99898489431708	8.72515867486691	14.67911182484448
C	5.36095719067136	8.24383632700567	12.02439796382123
C	6.04749711151537	8.15147584585183	10.74873563367681
H	5.62759051330958	8.45015054484277	9.78671151972763
C	7.30052826004304	7.64840095874262	11.00102068769682
H	8.10109402985198	7.45680628979850	10.28457728111469
C	7.38926848616357	7.42686209486962	12.43298198761435
C	8.51718629630880	6.89806960410859	13.09217345588482
C	8.63391025165707	6.75439715930205	14.48923438642575
C	9.77610944267171	6.16446457930750	15.16357452772350
H	10.62934826412155	5.70224489433965	14.66437021811047
C	9.55889414576006	6.29753018059576	16.51358638037383
H	10.19958615928631	5.96238844148840	17.33108733699576
C	8.28026707012887	6.96557246819428	16.67467966931792

C	7.72715727967799	7.34405689566787	17.91441619230271
C	6.45760563659336	7.93309431113655	18.07750361441335
C	5.92274770899425	8.39286473704999	19.34714335278492
H	6.44532125150714	8.34450006666606	20.30375824354315
C	4.67520931213695	8.90672321025654	19.09431044363888
H	3.97978205765186	9.35668601085270	19.80470280872706
C	4.43812270798354	8.76341897029837	17.66852134629244
C	3.25861120473932	9.16677171033909	17.01234111346426
C	3.05439616362327	9.09562010464171	15.62067420787295
C	1.81172505983871	9.44465440229337	14.95509498035386
H	0.88878487505905	9.73402145602516	15.46032678870973
C	2.02920367048832	9.31509856011389	13.60585367618695
H	1.31804989814012	9.47694071967525	12.79421527358840
C	3.40807622270450	8.88986962680278	13.43719749769522
C	4.04137760184886	8.71069657223885	12.19098703002396
C	9.68513167678388	6.49315056112786	12.25578022252967
C	10.92096794245650	7.16773395822012	12.38522665452158
H	11.00612450899106	7.99229951600031	13.10317337893766
C	12.02126595034127	6.80461653964482	11.59890196353810
H	12.97317320674218	7.33675637532372	11.69793914185952
C	11.90682435000372	5.75192496702090	10.66562364913059
C	10.67751518001398	5.07269585446243	10.53315167288379
H	10.60652166425701	4.25340495660250	9.80826995321366
C	9.57896429819372	5.44056173632288	11.31812443593632
H	8.62665743714718	4.90605774397457	11.21782145913703
C	8.56339719915217	7.14372785908707	19.13387730933136
C	9.78579526767117	7.84044724425230	19.27772609173909
H	10.10418094307059	8.52788435408609	18.48482775391663
C	10.57294601621094	7.67011315902163	20.42158208749557
H	11.51769982867308	8.21224305053440	20.54461335904820
C	10.15672469937916	6.79616945400103	21.44797665534847
C	8.93905007659696	6.09541982283749	21.31156030808352
H	8.61894738406570	5.41211166355438	22.10509829542122
C	8.15223011040971	6.27020900274254	20.16602063760426

H	7.21026754342625	5.71939496510613	20.05688204330147
C	2.15773983894232	9.73596298172061	17.84377090664881
C	1.75871754771270	11.08147940876957	17.67038847446276
H	2.27135263965989	11.69818533858357	16.92259380164364
C	0.73257982943602	11.62592173019744	18.44995664906478
H	0.42188187233075	12.67007841848265	18.32808012427218
C	0.07817468282316	10.83545060696171	19.41822012730723
C	0.46826526262597	9.49058590392711	19.59547065441707
H	-0.04425461104413	8.87417875130281	20.34145668965697
C	1.49898280987290	8.94991052235891	18.81623505622122
H	1.79545236031394	7.90224153245649	18.94684877666511
C	3.26639880174954	9.05708586204150	10.96357712836007
C	2.83083097283669	10.38383708632338	10.74198548952213
H	3.08135127505101	11.15690322612914	11.47831528170514
C	2.10087556182177	10.71599959330724	9.59402065908207
H	1.77133687689551	11.74649876139414	9.42505096773431
C	1.78981785702018	9.72173067948389	8.64167565942234
C	2.22315177575879	8.39627514929563	8.85529564222648
H	1.96862546364627	7.63482165798137	8.10888598379778
C	2.95425501002810	8.06829802244827	10.00230880159836
H	3.27986558466155	7.03500906177636	10.17178622068118
C	1.01008054320459	10.01311619086316	7.40211110423014
O	0.72444459787118	9.18373551877256	6.54978997516969
O	0.64381439975608	11.31556287823787	7.32030390979018
C	11.03601683831136	6.65280265666047	22.64618867967746
O	12.09994654201736	7.23593176629889	22.80082579815421
O	10.52180153440969	5.79489790678556	23.56085341750996
C	13.04366370867752	5.32037072909887	9.79902068320729
O	12.98716802995365	4.41569471547490	8.97796591472158
O	14.16354225479784	6.05102183192704	10.02049844476359
C	-1.01177902065994	11.46763961175023	20.21911019204847
O	-1.37978013821711	12.62776373310625	20.09816717133033
O	-1.55942846987324	10.60614303151622	21.11087640815905
C	-2.61952655035174	11.13983896803550	21.92250747369583

H	-2.25797760301388	11.99067637060497	22.52608892385210
H	-3.45705379179717	11.48587982384569	21.29188910471525
H	-2.94479456478056	10.31693565090082	22.57529488459650
C	-0.11451026087865	11.67724944846929	6.15328983961428
H	-0.33652610573369	12.74921398300911	6.25654911814249
H	0.47043695073083	11.49178484407278	5.23542827286969
H	-1.04949557375842	11.09276227406970	6.09950110337433
C	15.30543134359752	5.69977094596411	9.22006156767361
H	15.07925417974296	5.81679784552735	8.14585560784444
H	16.10899042116866	6.38750711026925	9.52068217853316
H	15.60453883547112	4.65329786926995	9.40539077851671
C	11.31182020736155	5.60176595096612	24.74678037740644
H	12.30457086457303	5.19180977387169	24.49129408644814
H	11.45220126151417	6.55733019376390	25.28160689029284
H	10.75188849598366	4.89067908979673	25.37117582134164
H	6.63158802266598	9.66362259885272	14.91035324389895
Si	7.20899144606293	11.04906377933027	15.29208746840273
C	6.14529980859204	12.31689845868952	14.39177060106194
H	6.55077723753712	13.32373737332295	14.62215345805903
H	6.29383748819346	12.17105816695796	13.30358532430211
C	7.09240845549386	11.20335783826691	17.15507816826493
H	7.65352196039823	10.39178278858525	17.64994216775045
H	7.50375147478837	12.17147941304001	17.49571540744617
H	6.04371114293619	11.13158409586303	17.49219138162134
C	8.99080236534827	11.06258313386649	14.67766536321086
H	8.97078069494999	10.86359553944887	13.58789141405168
H	9.37958133982310	12.09569737200287	14.79038221660810
C	4.64823415667412	12.24139603650404	14.74191913831449
H	4.06247378074555	13.01692360470900	14.21471880182383
H	4.22683941293556	11.25917462878213	14.46895647591345
H	4.47675827603014	12.37871698702567	15.82540635567378
C	9.91225859374244	10.05653338396490	15.38928873987904
H	10.94790519211907	10.10332644714887	15.00450730629236
H	9.95663259030680	10.24423623626003	16.47765546376255

H	9.55344529853411	9.02272376912077	15.25279886525528
O	4.96776826529189	5.58452339910381	14.99783492900409
H	5.74322179574958	5.22390391746816	15.46474004885374
H	5.21570410923257	5.52500119974173	14.05686124431577

Ethylene-Rh-O_2

Rh	6.03437228479557	8.77104486962560	14.90994662983902
N	6.50363763033884	8.54483040775847	12.93252502034527
N	7.93377474249955	8.15313513689020	15.42981045255276
N	5.56249584809475	8.97093369440856	16.89553937994245
N	4.06740500624747	9.11282018729983	14.41121422659149
C	5.65687267139955	8.76486887804863	11.86258616795097
C	6.41435673513400	8.68049753978633	10.62667499722047
H	6.00607277826068	8.84726062712757	9.62861558482275
C	7.70624110773659	8.36148182931835	10.96650619601069
H	8.55801103100332	8.21554675005692	10.30028829837056
C	7.75017187737659	8.24893559268133	12.41360793014553
C	8.88370077607176	7.85144906838057	13.15193159015605
C	8.93222674382930	7.76745170554077	14.55894103590204
C	10.07183134554442	7.29004249185248	15.32078788313369
H	10.98605930375121	6.87936184419355	14.88907431526807
C	9.75810261484915	7.43853274394345	16.65029615450747
H	10.36591648788059	7.17286274269797	17.51670044396503
C	8.42418416785664	8.00824235086398	16.71154422327990
C	7.76360636998517	8.38412741213556	17.89976271339035
C	6.43938225053224	8.86565903634327	17.95943669774459
C	5.77118091552501	9.29182609163064	19.17594227418504
H	6.23685795408745	9.34515267372098	20.16130700882907
C	4.47857705126592	9.60825830627092	18.83607034162736
H	3.68399831829971	9.97086949479517	19.49014302319512
C	4.34493474021772	9.37822137386803	17.40878394454102
C	3.14280866464083	9.51538986576162	16.68384502883880
C	3.02694496245378	9.33341431876442	15.28986822028868
C	1.77970779144763	9.38445264622540	14.54839182699470

H	0.79025281175004	9.49839930293944	14.99412717024273
C	2.09534608351520	9.23698709489641	13.21935169817267
H	1.41362904793055	9.20793291929550	12.36779302709400
C	3.53745324173418	9.09425278186221	13.13757654576083
C	4.26743366455635	8.99044809654767	11.93602088162448
C	10.11290544818161	7.47581207270600	12.39293771264774
C	11.30143212818816	8.22926386238924	12.52814647099735
H	11.31012070920662	9.10413894993440	13.18930646977981
C	12.45263572016889	7.87536676165045	11.81570095466208
H	13.37658662465895	8.45808369161215	11.90745333656652
C	12.44214604585200	6.75674512909473	10.95647130412681
C	11.26072643063555	5.99717925014471	10.81920968563586
H	11.25438843342875	5.12379466412208	10.15879075516571
C	10.10863340748310	6.35632431011704	11.52988560946609
H	9.19364029169021	5.75980799929725	11.43250715802103
C	8.51405981513257	8.24885436080060	19.18278479316469
C	9.68352881884440	9.00844385442171	19.41671640938251
H	10.03021321622582	9.70893681014384	18.64758429174265
C	10.38446573488995	8.88271879076482	20.62115105564028
H	11.28696158075097	9.47377066376663	20.81551281151993
C	9.93392105019727	7.99084936755113	21.61684742953282
C	8.77008239334934	7.22577369328726	21.38935289036173
H	8.42512772271818	6.52731284728233	22.15882002977275
C	8.06831035663297	7.35650504431213	20.18455728885789
H	7.17050121144385	6.75391329185148	20.00254753753970
C	1.90597519335384	9.86630658288644	17.44139600312662
C	1.21959571009954	11.07391644561846	17.17661235290598
H	1.61320644677763	11.75877602594251	16.41602346120263
C	0.05905855305011	11.40278667776426	17.88548448505536
H	-0.47554088555137	12.34021533931333	17.69286708645771
C	-0.44383552863184	10.52911362464453	18.87214581442092
C	0.23326202829552	9.32029668156120	19.14013210061154
H	-0.16138810667194	8.63767364977743	19.89988643372241
C	1.39673633117527	8.99530742118644	18.43162100964522

H	1.91539199307335	8.04993502329389	18.63076853745275
C	3.50968015674255	9.09760518000442	10.65500211623903
C	2.84415139298232	10.29587985420620	10.30853519663083
H	2.90097224513589	11.15577150593068	10.98665983994928
C	2.13322524892088	10.39275144797761	9.10719796497414
H	1.62099319017790	11.32005174914219	8.82552670449912
C	2.06782384084190	9.29128361554716	8.22841997058187
C	2.72540922290401	8.09003850294324	8.56956368586158
H	2.66691186475025	7.23202046089537	7.89178353245168
C	3.43969736524795	7.99705798466994	9.77052908777648
H	3.93999798447792	7.06006835919753	10.04278371373991
C	1.29372529248231	9.45058095074401	6.96093907939364
O	0.71236248198810	10.47320137917513	6.62653455910628
O	1.30420668678590	8.32366497395576	6.20887909296157
C	10.72152809640772	7.89746156167881	22.88261206182419
O	11.73420630540590	8.54107899725147	23.11853326490331
O	10.18586680893486	7.01069154671291	23.75560928785973
C	13.70028507699207	6.42433287940613	10.22303028412060
O	14.74291174144570	7.05725507720918	10.31155516877821
O	13.56146820349957	5.32911584456012	9.43771546132886
C	-1.68744559151286	10.93069016892459	19.59558113138596
O	-2.30370527763501	11.96738800848205	19.39318748909291
O	-2.06576195556090	10.01047841430873	20.51514020905618
C	-3.25829529974816	10.32350289619265	21.25579419172968
H	-3.13267284487766	11.26673103060487	21.81545077670770
H	-4.12223708330785	10.42853533011600	20.57658170215947
H	-3.41397390628344	9.48356899985308	21.94804331415983
C	0.57758471517088	8.39389939048157	4.96982607340302
H	0.68877077622301	7.40729205194609	4.49736693736893
H	-0.48807022341310	8.61480405026001	5.15508537775823
H	0.99328524619796	9.18232340235692	4.31839225303635
C	14.73128858538146	4.94064041680162	8.69658800909647
H	15.04142850705796	5.74627432307234	8.00841772410724
H	15.56919731703096	4.71822567200470	9.37997834544631

H	14.44684040938666	4.04141339350110	8.13143636923997
C	10.88719821732490	6.86216986009434	25.00250047312011
H	10.32265454375122	6.11626476103364	25.58030696260635
H	11.92045617818775	6.51428182598018	24.82917507918707
H	10.92455059959283	7.82290876989703	25.54497063011998
C	5.94815772681658	11.08471393232301	14.65109319795705
H	5.23378927300753	11.41135168892001	15.41600002012447
H	5.64237557450201	11.22017656324513	13.60696735765181
C	7.24744747966858	10.76521735779312	14.97811796344590
H	8.01232738739172	10.63493363110027	14.20332979669994
H	7.60410853349224	10.82747983512311	16.01295662722690
O	5.55160161572028	6.78085683303411	15.04588504598026
O	4.87125677158043	6.28323470709180	14.08251064444856

6-silane

Rh	5.92171406631910	8.18094212427032	15.15234743278822
N	5.99084910819275	8.03349707160699	13.11263624808672
N	7.86798392277480	7.56254794062131	15.26311860628703
N	5.83285803577535	8.27809307464214	17.19533029855131
N	3.99492526220634	8.86363141469541	15.03847979984075
C	4.97797659965684	8.36207078244218	12.23047831280102
C	5.35541788285408	7.96010514663680	10.88644893002192
H	4.72818814091378	8.06814414355285	9.99988270974602
C	6.61012191317246	7.40728086168499	10.96964528277390
H	7.20738251265543	6.97965403232727	10.16275752127729
C	7.01762585358584	7.48728267153253	12.36156162227019
C	8.29221317754320	7.13470951250223	12.84837849320879
C	8.68824598183215	7.23596317327321	14.19782945453266
C	10.03882059337816	7.00339170590540	14.67973363871408
H	10.89784000272847	6.76602984467804	14.04987568785201
C	10.01169401841778	7.16158440378287	16.04434260014503
H	10.84441309445235	7.07906085188700	16.74455193211208
C	8.64275046651092	7.48126593716651	16.40701764099786
C	8.17866741676565	7.63852436184442	17.72747001098422

C	6.85223034051511	7.96232699555053	18.07625426737925
C	6.34415748140567	7.98994551851976	19.43690058233094
H	6.92486639540979	7.74778630256507	20.32856947211496
C	5.01880697359486	8.34378786147542	19.36379296382435
H	4.30743758288980	8.44666799499537	20.18465570005955
C	4.71115621864261	8.54829106387410	17.95962567793967
C	3.46480200323421	8.98386093554969	17.46697265389364
C	3.16965280948378	9.17981857329954	16.10246910785720
C	1.94858147572819	9.79038883542910	15.60556336942407
H	1.13595758032365	10.16706176631924	16.22913196832537
C	2.04690502572381	9.82919207309811	14.23625074999558
H	1.32759239312682	10.23831361421318	13.52503276113172
C	3.31803494403125	9.22060152767940	13.88456371852822
C	3.75445842483634	8.97769685342964	12.56658502194752
C	9.30657057604178	6.65284934495802	11.86632065428934
C	9.75771611426400	7.49156453273197	10.82141130277813
H	9.36075845822522	8.51067863655081	10.74507843875421
C	10.71102065020459	7.03823289951810	9.90113315415778
H	11.06142056562978	7.69459244213579	9.09770236208305
C	11.23193652777965	5.73077366244203	10.00780753950573
C	10.78820817764814	4.88954411081584	11.04992709738131
H	11.19899489140081	3.87535497899720	11.11596246113672
C	9.83802164710535	5.34648616719572	11.96955077799287
H	9.48696589359228	4.68897726219019	12.77392539365366
C	9.15167527855473	7.42176843089856	18.83721094067770
C	9.49052170102797	8.47977275265714	19.71161312022089
H	9.03817905117757	9.46662744458883	19.55685829861614
C	10.40162404964167	8.27723990237738	20.75407639003296
H	10.67759816994969	9.09301177913136	21.43238132340403
C	10.98991083284457	7.00977072242533	20.94882551466791
C	10.65446271297716	5.94737004266406	20.08220171428910
H	11.10512483516864	4.96162070880579	20.23789504427140
C	9.74612205461463	6.15481641833151	19.03679800411085
H	9.47671539102525	5.32812462239169	18.36896346703867

C	2.38919432032895	9.28583433699023	18.45465597998250
C	2.55096095145149	10.31376442801846	19.41146090520646
H	3.47706483263802	10.90047104438495	19.41269280825442
C	1.53823585602940	10.59656690790025	20.33627405663787
H	1.66639542519154	11.39970871094950	21.06948962345402
C	0.33955633132458	9.85136014243084	20.32310553293661
C	0.17095959254460	8.82480265806269	19.37004125983792
H	-0.76396650020937	8.25247408305635	19.37578572526925
C	1.18343814017507	8.54711043966754	18.44526404988364
H	1.05417738929172	7.74337932623472	17.71037334630627
C	2.84951867344511	9.36352916201104	11.44492432833913
C	3.25611073987004	10.33646822767760	10.50283191306533
H	4.23796050238085	10.81192217313324	10.61452927708918
C	2.41318492568140	10.70159515026209	9.44753978342603
H	2.71581642187729	11.46072998889379	8.71688221239233
C	1.14553758922640	10.09833514752499	9.30762729078110
C	0.73439190441463	9.12189090530493	10.24021890634893
H	-0.24551202835276	8.64642851694930	10.12703678400614
C	1.57930616974748	8.76094073610731	11.29721592650493
H	1.26639399116795	7.99356233408780	12.01499992278413
C	0.28756702477362	10.52735414093261	8.16347510387243
O	0.60688306320875	11.36453391008787	7.33091327099219
O	-0.90379508867262	9.88110192357277	8.14499380142604
C	11.95262327903500	6.84937078354796	22.07885365039567
O	12.27265210106747	7.74195462466120	22.85137248286648
O	12.44261726466286	5.58815516722886	22.16001537175832
C	12.24367230713599	5.18929578871028	9.05254128969406
O	12.72414357252073	4.06609500143730	9.11188330751387
O	12.57777015700473	6.08800587756308	8.09438121878357
C	-0.77437150414086	10.10574084016435	21.28409070874813
O	-1.83085366706507	9.48972532197828	21.30224951483372
O	-0.48686737756392	11.10863305677904	22.14949613634111
C	-1.51264295381831	11.41679967669354	23.10891193100883
H	-1.73380536741653	10.53783164421659	23.73927287313619

H	-1.11662900705610	12.23915482875834	23.72209637513469
H	-2.44157835776999	11.72828448498907	22.60007496014718
C	-1.79090199877249	10.23981607033264	7.07174032502035
H	-2.04267966922536	11.31385007317927	7.11636438565709
H	-1.32571836787110	10.02714328903656	6.09335351235439
H	-2.69435667396230	9.62809892160825	7.20834201262019
C	13.54427417035286	5.63875253336166	7.12904107484458
H	13.17162589137020	4.75071359345928	6.58932170234365
H	13.69036669132829	6.47780910758797	6.43355228990238
H	14.49654304723827	5.37736111434951	7.62278388928164
C	13.37947205096906	5.35012152618727	23.22458402231160
H	14.26849090784260	5.99517483211778	23.11291589920978
H	12.91595048273831	5.55508686257048	24.20536398608983
H	13.66251146275205	4.29037941284257	23.14753841625764
H	5.41310560435014	6.37594040137102	15.12167190917146
Si	4.74313732378278	5.05989486195138	15.55859798371235
C	6.11097428793426	3.76313703322468	15.54386952533343
H	5.67171556874918	2.79542449777644	15.86204467015702
H	6.43487895573212	3.62083791783623	14.49401324062810
C	4.03945349730897	5.31441978565748	17.27703944054156
H	4.82782565826853	5.59878814539528	17.99538040830971
H	3.28472707775075	6.11918212227733	17.28025785517361
H	3.55910870011173	4.38589476592100	17.63777550112527
C	3.39748395428759	4.69654645885500	14.29119801746945
H	3.88836821127345	4.56232551233933	13.30711417973760
H	2.94769290492088	3.71443713590699	14.54417631758465
C	7.31515880866651	4.13210156675275	16.42989302150385
H	8.09006451657625	3.34320051210285	16.42305591479901
H	7.78648877101233	5.06857578399282	16.08633993522316
H	7.01392689014666	4.29059975643093	17.48175886525176
C	2.31318160410077	5.78538799919981	14.20330772617198
H	2.74748050664047	6.75261688588456	13.89929741562804
H	1.52827945549369	5.52549353217120	13.46896633528580
H	1.81443927358897	5.94775511986018	15.17624377211344

H	6.40981954898550	9.98838085250979	15.01041604912975
Si	7.25239822027667	11.27300604295468	15.11173985963791
C	6.23506984401653	12.46999214594419	16.15175278100926
H	6.78689623969407	13.43021377637725	16.21527138591099
H	5.30173461205519	12.69063470877180	15.59705174323209
C	8.87483800196538	10.82716158687677	15.93794430027434
H	9.42033678364041	10.05980600619069	15.36187159746685
H	9.52113079478051	11.72010591382331	16.02683612311703
H	8.70277539754643	10.42052901711665	16.94945871189751
C	7.49852720168153	11.88461596550960	13.34681441413708
H	6.50075348851942	12.12816848382347	12.93138073950095
H	8.05310703994227	12.84452617305883	13.38891859243057
C	5.90699762761833	11.93623941291331	17.55783752424722
H	5.33159068459756	10.99668794490093	17.50091273401364
H	6.82266679134633	11.71934271718056	18.13786540517283
H	5.30915583674044	12.65942116130964	18.14318004065763
C	8.22501625376121	10.87536615344276	12.43942270792232
H	7.66024164791647	9.93055417336660	12.36757573906815
H	8.35889850797038	11.26522697703444	11.41329837954060
H	9.22744132095968	10.62259099463691	12.83083263705692

11-ethylene

Rh	5.96399764459397	8.48520588106191	14.90147362949566
N	6.46805541480658	8.30671430610203	12.92276385395957
N	7.86875240081839	7.94745995357309	15.43746941102711
N	5.49783610534709	8.80460460436149	16.87271108653021
N	4.02857283379913	8.90357785149963	14.37474944401665
C	5.64131599852463	8.54549882942889	11.84023124589173
C	6.41942573963722	8.48152256486510	10.61430580688219
H	6.03097317893684	8.67229805296629	9.61243678379131
C	7.70506493160272	8.15505142217663	10.97129274788336
H	8.56960836184014	8.02750072999245	10.31757561640066
C	7.72301054228118	8.01922101891499	12.41815438854541
C	8.84558923320173	7.62099270633047	13.17431116492966

C	8.88161739148089	7.55243909435964	14.58292791806037
C	10.01802802762101	7.08842254830178	15.36119935320954
H	10.94073146176414	6.68080872877281	14.94450678124692
C	9.69205065606783	7.25356968867462	16.68534231228733
H	10.29642078791942	7.00652542031664	17.55982959378564
C	8.35435900441114	7.82057326288328	16.72615563572964
C	7.68277535446921	8.21334722055737	17.90308878132780
C	6.36443544104301	8.71420828961966	17.94651516544603
C	5.69270529348763	9.17517721891474	19.15001323878697
H	6.15340103712726	9.25262627040232	20.13635646399016
C	4.40467423887262	9.49364746897430	18.79415178049175
H	3.61050557166618	9.88281170530361	19.43363302587136
C	4.27949331161958	9.22922263042501	17.37053030865728
C	3.08335314276707	9.35026739916188	16.63156344321134
C	2.97744912247134	9.15292384787556	15.23827955939335
C	1.73812947790214	9.22574557672418	14.48238369984363
H	0.74607936431107	9.36910730313187	14.91399137880084
C	2.06568803739158	9.06892520275497	13.15758536601161
H	1.39269046477576	9.05889265111151	12.29846160799562
C	3.50724415009533	8.89619103670785	13.09377340988480
C	4.25095443000938	8.77849044223117	11.90045342609634
C	10.08499122559771	7.24858362200943	12.43032931329598
C	11.27267965664721	7.99977873461146	12.58752989174464
H	11.27198185231607	8.86885271732360	13.25627925285826
C	12.43334527936695	7.65215229456482	11.88773895534164
H	13.35593583421313	8.23411800372155	11.99683639792937
C	12.43468291756053	6.54157281152303	11.01784740794277
C	11.25428908127696	5.78491810324771	10.85638060262395
H	11.25639200140566	4.91895506381424	10.18613065117740
C	10.09303638114165	6.13859782257332	11.55485982153747
H	9.17818538537843	5.54568443233320	11.43620995000079
C	8.41570249084157	8.08379938451801	19.19726252762120
C	9.58761616732750	8.83540443231807	19.44252175556229
H	9.95030361373743	9.52574204291696	18.67151834606173

C	10.27326049892438	8.71775079589979	20.65815325950037
H	11.17523669339224	9.30971603243427	20.84557182623157
C	9.79983005069613	7.83759009947049	21.65468420420444
C	8.63269969271983	7.08196646324678	21.41567400752494
H	8.28216558962018	6.39801114764637	22.19736467070543
C	7.94769114694140	7.20563278179029	20.20209534673647
H	7.04512329515336	6.61160805439947	20.01515466141193
C	1.84101117112873	9.70922216743059	17.37720377064985
C	1.15206154741022	10.91203905439104	17.09699456325718
H	1.54847792396135	11.59031377200847	16.33195310029536
C	-0.01390033134435	11.24471661950184	17.79529143536021
H	-0.55015481524479	12.17847588920040	17.58949519238968
C	-0.52007815728743	10.38048424656674	18.78872969253191
C	0.16075372462775	9.17783057951951	19.07495299933827
H	-0.23497629288763	8.50332382246205	19.84147543796299
C	1.32899998952622	8.84945763510537	18.37604606531944
H	1.85197691166615	7.91007480928849	18.59191980367724
C	3.51555764384125	8.91466761103473	10.60865883490459
C	2.86860103899073	10.12659880804365	10.27451259536893
H	2.91888495944423	10.96915881679195	10.97458334668206
C	2.18596545304645	10.26377712664012	9.05940175111149
H	1.69558378844850	11.20859609439487	8.80257604238326
C	2.13371873891724	9.18416994139538	8.15189254694708
C	2.77284781917135	7.97020230846249	8.48043113782763
H	2.71691324210769	7.13892850324446	7.76797485881597
C	3.45670566909294	7.83778453851481	9.69393070987966
H	3.94592706745251	6.89042996394159	9.95011628595794
C	1.42623871919728	9.27201913984283	6.83988029107841
O	1.36371628278181	8.36478165082315	6.02205390297778
O	0.85297079324746	10.48473045757627	6.64661017754052
C	10.48740580252361	7.66795363169060	22.96924863603961
O	10.11476671320096	6.91178927160268	23.85541149963447
O	11.58591426094228	8.45396391225269	23.07940910001922
C	13.70226353152040	6.21544059941924	10.29915552552808

O	14.74391570899938	6.84736640154818	10.40651961990076
O	13.57531283596016	5.12596927736434	9.50310930025498
C	-1.76937182339087	10.78418341710826	19.49997778448840
O	-2.39237655734640	11.81347053871769	19.28021617190695
O	-2.14612577176935	9.87428332579597	20.43106304263304
C	-3.34357424285669	10.18936613241027	21.16217777154033
H	-3.49414599357517	9.36040523131774	21.86872783458290
H	-3.22878867142780	11.14344497263296	21.70562671882800
H	-4.20647558315531	10.27448676527488	20.47873113110675
C	0.15710084111328	10.65382591942476	5.39974173796898
H	-0.22990995942908	11.68303467270347	5.40610797361996
H	0.84261822633465	10.50477668490144	4.54731202500570
H	-0.67310346225917	9.93091961029127	5.31495563033503
C	14.75568611001921	4.74529192031397	8.77514748317919
H	15.59048491242338	4.53779073353834	9.46694626279910
H	14.48629628067925	3.83909301211093	8.21365595621779
H	15.06252945574151	5.54932032748834	8.08341349152929
C	12.30448884334808	8.34952773666193	24.32065280857342
H	12.68351152979058	7.32277568075696	24.46613085430475
H	13.14051149047144	9.05997371390314	24.24735276174503
H	11.65139610662636	8.60809446623771	25.17229657857518
C	5.14047973980158	6.06667320874449	15.71570338881994
H	5.87188358739402	5.90804343906070	16.51748143795384
H	4.12260883380981	6.33611888648422	16.02269547625545
C	5.46590062139629	5.88890885707989	14.41100056975088
H	4.72438424315204	6.00871126324754	13.61177524091864
H	6.47374134561512	5.58118230200963	14.10686028529277
C	7.29705707131166	10.86883501048483	14.91696065259949
H	8.05623122093478	10.64262246931105	14.15848116670123
H	7.62749367820251	10.86637788067247	15.96280659473193
C	6.01816228123165	11.16050674405495	14.57641887032149
H	5.26646310199516	11.41912388250056	15.33208211656051
H	5.69558021979005	11.19388301806903	13.52871004925268

6-TS-b

Rh	6.02071079117755	8.14503874605181	15.15554884849994
N	6.24964629378292	8.10791538705873	13.12646223647436
N	7.95446022436064	7.54321645896558	15.37997266783026
N	5.83434236073145	8.29857224631832	17.18102010714803
N	4.15416226919645	8.92549594534299	14.92514787516899
C	5.25159076030379	8.30867010425778	12.18145891343371
C	5.75128938622211	7.96902177429541	10.86278157686665
H	5.16683020936071	8.00220014025823	9.94199590846572
C	7.06770371531706	7.60761475037810	11.01263553422743
H	7.76587263260679	7.28325942349566	10.23930695548865
C	7.37681984378565	7.70275658777953	12.42613903661057
C	8.62706065925317	7.38224721594977	12.98797681592080
C	8.88456065095654	7.32993605804526	14.37093876493482
C	10.17307360895623	7.00463247234391	14.95334789693464
H	11.09004700162068	6.82932703855625	14.38848400862473
C	10.00556910948369	6.98755267113851	16.31586426042976
H	10.75924256664038	6.79824132299727	17.08210829340648
C	8.61751675332063	7.31853765690701	16.57782852964894
C	8.05887228334655	7.42233905725235	17.86530175730415
C	6.75259555261991	7.87256323894330	18.13012919780473
C	6.16377063558990	7.95629828264116	19.45353443006592
H	6.65477013375418	7.65352775270417	20.37968719106573
C	4.89934290828119	8.46927618532000	19.29901892708414
H	4.15662800066007	8.66616584168904	20.07380166526223
C	4.70389058093536	8.69692973576823	17.87985519849205
C	3.52605557366038	9.22129088261140	17.31333789164312
C	3.29807299655353	9.34961592668017	15.93079321087204
C	2.09432041826259	9.91068310044174	15.34647132290404
H	1.27502966931005	10.35919566077841	15.91062449903988
C	2.21016999133012	9.77906049454798	13.98511527893864
H	1.50457683615464	10.09955243402362	13.21684324117467
C	3.48908361653621	9.14409015309389	13.72764526358969
C	3.95930782424818	8.79927174116982	12.44658349425958

C	9.74090524280511	7.04884796105218	12.05172198734565
C	10.23727079243325	8.02529952196955	11.15827241724042
H	9.80231840412579	9.03189262053293	11.17199422195986
C	11.28003720145044	7.72287018738622	10.27363443431960
H	11.66543967721717	8.48613982452632	9.58958873738329
C	11.84586356980381	6.43008717577150	10.26330212682957
C	11.35241042202176	5.44963945924579	11.14938920581532
H	11.79723949530375	4.44806069042861	11.12378946238822
C	10.31268532747017	5.75593803832984	12.03412041781508
H	9.92409083759796	4.98693629378371	12.71174725976137
C	8.90436192028944	7.00838573229802	19.02279516792512
C	9.29879142234185	7.94120738691892	20.00824759741398
H	8.98998952453439	8.98861483775097	19.90752391629831
C	10.08664745173090	7.54278445434880	21.09555497535826
H	10.39544104467533	8.27115112111879	21.85261148913709
C	10.49392426379198	6.19704946360728	21.21864146899413
C	10.10446986817693	5.26134701421291	20.23728176433619
H	10.42501613333238	4.21907202596810	20.34951675223021
C	9.32023924383311	5.66305493566811	19.15044560532355
H	9.00860250946584	4.93359626694099	18.39352295952492
C	2.42427582070620	9.61459518559397	18.23989318675116
C	2.58587444992835	10.68829949674755	19.14380595671154
H	3.53086666031030	11.24466403031390	19.15003369584916
C	1.55034096380857	11.05143638325173	20.01440541523081
H	1.67750611585691	11.88958770001657	20.70745786830252
C	0.33098144550272	10.34097243075416	19.99851863051973
C	0.16516585641573	9.26567575224364	19.10033336047799
H	-0.78578810049039	8.72046891864976	19.10491386935167
C	1.20016427859551	8.90731036537969	18.22992169721993
H	1.07524883534536	8.06474490423472	17.53949909044941
C	3.02108267901534	8.97226567722105	11.29883501648306
C	3.29281629513199	9.89709396017589	10.26506240254712
H	4.20468736070552	10.50404787095843	10.31503876382023
C	2.40200640350755	10.05056635802737	9.19676656441886

H	2.60000434151352	10.77043963933694	8.39414273701343
C	1.22115872770491	9.28101714986048	9.13781052325138
C	0.94246987429531	8.35605597653092	10.16677728060050
H	0.02790066627353	7.75580107431227	10.11913168069199
C	1.83428776014428	8.20698742216808	11.23608147854400
H	1.62281082160984	7.48396590787097	12.03304458496510
C	0.30843557939425	9.48541231259048	7.97351589921347
O	0.51443243004806	10.27093264865518	7.05910803389405
O	-0.79084638560141	8.69545042277248	8.03837554161402
C	11.33079157337325	5.71288067687053	22.35681188111895
O	11.70768242696576	4.55823940891551	22.50002568735052
O	11.63298257048678	6.70465567494490	23.22966225743798
C	12.95912539998731	6.04818067564600	9.34422133772327
O	13.47923746850828	4.94194949556538	9.30553403776215
O	13.33894774610640	7.07426538839853	8.54460799899296
C	-0.80885823289787	10.68212016522627	20.90107131125659
O	-1.88308382989225	10.09763034698637	20.91435428258748
O	-0.52294212000380	11.72557946844147	21.71714857120156
C	-1.57433710904600	12.12092075779778	22.61497364476109
H	-1.17529541597577	12.96543847246530	23.19515062444184
H	-2.47197207897366	12.43052430578263	22.05170510762487
H	-1.84883494587232	11.28794769572144	23.28552280689131
C	-1.72222441123607	8.83269621956662	6.95129539483688
H	-2.54355047413912	8.13393833515012	7.16626126171111
H	-2.10197742680262	9.86757575094658	6.89155388723902
H	-1.24090729492381	8.57779602275962	5.99096396663342
C	14.41108555787319	6.78801594138518	7.63004148589700
H	14.58470592215791	7.71634374875810	7.06691143823164
H	15.32301021648768	6.49370913128299	8.17837821880510
H	14.13115667050495	5.96837550432167	6.94550067950227
C	12.43866393578841	6.31913821289174	24.35667819145243
H	13.41412081791335	5.92500127583383	24.02194123101601
H	12.57830344881042	7.23097417190610	24.95510473133955
H	11.92949335437111	5.54123750688199	24.95196817072728

H	5.45720586951188	6.61063765390295	15.13410916579248
Si	4.76479946977110	4.89439694756697	15.66853571819564
C	4.03695636277658	3.15376343233303	15.35151230395861
H	3.52057767010750	2.94984624095769	16.31445142211809
H	3.22880248060022	3.23576550700161	14.59845338341491
C	6.09787482323848	4.72132042451519	16.98617941897525
H	6.10700671816128	3.69977495545676	17.40630776332090
H	7.09857023086746	4.93857957913034	16.57689421078578
H	5.91200913320957	5.43636661382280	17.80525434384858
C	3.21607646929140	5.84348523473254	16.22320066684027
H	2.72012791049277	5.21814710049316	16.99454609744888
H	3.53308997664681	6.77230828937351	16.72212424315817
C	4.96802912086884	1.98397686825760	15.01553870798838
H	4.44033582176963	1.01429522847895	15.08814866662686
H	5.36566166534302	2.05238046450201	13.98728300828325
H	5.83407272527777	1.93559068035999	15.70174739025934
C	2.24659869207870	6.15232673980398	15.07255972114615
H	1.83837696052970	5.23071107383252	14.61897494096103
H	1.38929361242886	6.76368535215556	15.41067446271462
H	2.75142999125158	6.72242229266201	14.27267591663428
C	5.75243377881521	4.81941435064121	13.46615644474471
H	5.00284275876644	4.11043250747009	13.08924101775361
H	5.60990691085766	5.85867473759327	13.15764276185297
C	7.01587297714623	4.36085547866879	13.79711844534265
H	7.22864146355915	3.29307361503870	13.92037662859317
H	7.81779284661663	5.06159171946907	14.05142060775876

6-TS

Rh	6.04721036006527	8.23016098685243	15.20506397275020
N	6.26285727123313	8.15950563181673	13.17654003216888
N	7.98494636991428	7.63839972769190	15.42934235396102
N	5.84604349134087	8.34031521504535	17.23311207956475
N	4.17109839044579	8.98945902938422	14.97777761717704
C	5.26651818727637	8.38030811547718	12.23613968824303

C	5.73343121151708	7.97325068382555	10.92417914298552
H	5.14081451436792	8.00575120412636	10.00858188929367
C	7.02860181254775	7.54224975716152	11.07486128698786
H	7.69854590992640	7.15341662143517	10.30643945148935
C	7.36073719214019	7.67731364887096	12.48054644059839
C	8.61218335409622	7.35161839737802	13.03826864379038
C	8.89701168192946	7.37712495575768	14.41677932354949
C	10.20070284199379	7.10232146874647	14.99172919689603
H	11.10997038683947	6.90965407870664	14.41998114548419
C	10.05860900978596	7.15897533778516	16.35612823044486
H	10.82847183905285	7.01998867026966	17.11698085605177
C	8.66722503915769	7.46988936901531	16.62591774375127
C	8.10544253414697	7.55303199484441	17.91382976049188
C	6.77257225236577	7.92231869985954	18.17647969578331
C	6.16189983020617	7.91609030812623	19.49287332503621
H	6.65646758896979	7.60032314819771	20.41286658605834
C	4.87126158643883	8.35975233125642	19.33943563860682
H	4.10716105756335	8.47761665703536	20.10951398200545
C	4.68509434020058	8.64370176890912	17.92883454464198
C	3.49959827169859	9.15635528601738	17.36712776454065
C	3.29620079552407	9.35790535731956	15.98886154283142
C	2.10294100291626	9.94922853942790	15.41282032105706
H	1.27298865801393	10.36865990203468	15.98382935451795
C	2.24483217002902	9.89089560797643	14.04840795496635
H	1.55322268668082	10.25227454885057	13.28565474006219
C	3.52386096292154	9.26032241290345	13.78132632465043
C	3.99610780033460	8.92850756047059	12.49722678394633
C	9.69537671202294	6.91927217025230	12.10827032363030
C	10.18811037341674	7.79986577623174	11.11828770054516
H	9.77622103260669	8.81404723841871	11.05292734307715
C	11.19978055589668	7.39446465056928	10.23896916494302
H	11.58304807105927	8.08390600038704	9.47948517544410
C	11.73737062757801	6.09275176584831	10.33115996331308
C	11.24983522256989	5.20847181341800	11.31639681601873

H	11.67370695040692	4.19898076381182	11.37088777426198
C	10.24195685170598	5.61789912168513	12.19620340257321
H	9.85787345841457	4.92606810423821	12.95485491436749
C	8.96853626696035	7.18826902175593	19.07471767753616
C	9.28369095804009	8.13932770678668	20.07151030999701
H	8.89976487841228	9.16200667409646	19.97528627214826
C	10.08766710188690	7.79070701611010	21.16403942583762
H	10.33545408157607	8.53364767526987	21.92933636949969
C	10.58995586277523	6.47701791085029	21.28238129536050
C	10.27788442985476	5.52252809203754	20.29141435675661
H	10.67037170683880	4.50486134312142	20.40080727421760
C	9.47804129743561	5.87496487021043	19.19897965225735
H	9.22661691811236	5.13024059913528	18.43452607445954
C	2.37089631903487	9.47470146320971	18.28930858269088
C	2.49747200845364	10.48641586751081	19.26786965686858
H	3.43524956716208	11.05081219031352	19.33557572343401
C	1.43641871364909	10.78105378787625	20.13333982892886
H	1.53688113222556	11.57194690153965	20.88398234460875
C	0.22505113557928	10.06318037260533	20.03773476937130
C	0.09303919398953	9.05046087400098	19.06442752475782
H	-0.85214451013973	8.49819111907106	19.00689022659292
C	1.15358003424369	8.76075953981057	18.19918036589223
H	1.05302110674999	7.96631839747154	17.45003713375021
C	3.07868700775178	9.15133531026705	11.34169943581513
C	3.41603798885566	10.05756117235200	10.31025161525216
H	4.36016936128765	10.61171817845906	10.37251920789322
C	2.55051791292894	10.25964073588551	9.22945900210481
H	2.79980931521112	10.96631344368194	8.42939535287855
C	1.32924859631228	9.55742538906427	9.15388248358443
C	0.98562299091850	8.64982931801871	10.17874404858822
H	0.04066469480366	8.10001173171788	10.11755143661438
C	1.85235528990820	8.45233275712368	11.26058671741713
H	1.58995415002459	7.74038349157456	12.05214930394235
C	0.44518370329616	9.81175187282356	7.97769109356809

O	0.70673984251399	10.58387188391396	7.06601882463973
O	-0.69767413069277	9.08485211119853	8.02833308878885
C	11.44843181385427	6.04592261165078	22.42572042019829
O	11.90403085940011	4.91967015126354	22.56618603505287
O	11.67293340891063	7.05098820855658	23.30685473825797
C	12.81458995507965	5.60321205968476	9.42030211156160
O	13.31140425871766	4.48669425080358	9.46892527095896
O	13.18999225632006	6.54281342528538	8.51852847577285
C	-0.94049801686798	10.33292490432732	20.93135462288411
O	-2.00966401321161	9.74159867387755	20.87594867936718
O	-0.68439974337414	11.31894364043098	21.82512599828027
C	-1.76134190229263	11.64338164341195	22.72110029833221
H	-1.38334298086835	12.44573296924987	23.37103420872264
H	-2.64608129427172	11.98847236630272	22.15804541092358
H	-2.04752805887176	10.76278011912525	23.32230179496640
C	-1.60596923146967	9.27358759445010	6.92962517790761
H	-2.46567239610409	8.61845673533435	7.13160097225040
H	-1.93035629231569	10.32710124598378	6.86844762842163
H	-1.12619786446361	8.99630037478111	5.97474726685592
C	14.22821845442806	6.15038718065564	7.60442856415956
H	14.40350289006998	7.01952323367224	6.95406611518656
H	15.15019756588009	5.88661226539951	8.15149477647715
H	13.91200379820722	5.27849635106860	7.00548412558504
C	12.49248231475802	6.71474497814986	24.43970257196023
H	13.49481091024334	6.38741160442529	24.11249079803153
H	12.56599638963491	7.63115425631357	25.04290943373680
H	12.03056468407821	5.90223528243322	25.02725907778434
H	5.49356709231422	6.70906495519909	15.22760525107550
Si	4.87388139102433	4.98469892119366	15.41882098366764
C	5.25745161799185	4.84442592153065	13.56599850411311
H	4.54719528156265	4.12928603280289	13.10877590853542
H	5.09670095050999	5.81995368570630	13.07671357433778
C	6.25682618417172	4.75361852060052	16.67646253964642
H	6.44376856769804	3.68839817438412	16.89665851541622

H	7.19299386827822	5.21319253966276	16.32060341702044
H	5.97719272339231	5.26778428602827	17.61353822239661
C	3.23711860974375	5.75324747488464	15.99329190645290
H	2.72997428631069	5.04612764588484	16.67802668379837
H	3.46658364994793	6.65404920633680	16.58756706858012
C	6.70407476969362	4.37263302977924	13.36270223342761
H	6.95473212785067	4.27876207712437	12.29002353889915
H	7.41957493527212	5.08516342819066	13.80727763153718
H	6.87835303355214	3.38607641605011	13.83392569170609
C	2.32277103988834	6.11098105644213	14.81183681765930
H	2.01354684007348	5.21362773883097	14.24424623228108
H	1.40285810473881	6.62147207847542	15.15201387865435
H	2.83120484413899	6.79082748293028	14.10739822453178
C	4.04321791730034	2.90188761471191	15.58754317969583
H	3.91053419294023	2.96532395462532	16.67897973939660
H	3.13345198869142	3.11381170684833	15.00432538654891
C	4.93032320747833	1.94664166764947	15.09104727442407
H	5.72355994909132	1.52076175969949	15.71799247763215
H	4.93135952759336	1.66203667148782	14.03179294631390

6-TS-f-1

Rh	5.41532041020871	8.58990495036675	14.82743235296366
N	5.85196114161749	8.36221103934457	12.84699752762479
N	7.19817862330317	7.71865765095921	15.30097131294998
N	5.06683957704326	8.95457986131815	16.79728445235580
N	3.66185862887154	9.50846294989289	14.34296551388134
C	5.02371279755142	8.68694553884959	11.78278367594425
C	5.72748738444614	8.47322228124777	10.53223201243551
H	5.32395755972176	8.68939582097394	9.54171550150246
C	6.97693121152469	8.00150696993387	10.84986674419293
H	7.79287016851367	7.75389529410863	10.16925497641842
C	7.03674271437024	7.89058090644627	12.29494457303058
C	8.11080637147625	7.32900367417284	13.01041000828533
C	8.12165116381100	7.18313679667114	14.41373383064856

C	9.12947837634174	6.44144663377538	15.15104836522644
H	9.96206178095907	5.89900261728450	14.70182263138530
C	8.81459049944393	6.54563064959170	16.48325376362880
H	9.33556070487895	6.09820689608024	17.33024672709621
C	7.61781847857517	7.35762917095742	16.57337825075592
C	7.00157554873777	7.73597062237364	17.78130251137753
C	5.84130857861160	8.52743837762732	17.86628904285896
C	5.26696519719994	9.00691535907097	19.10936994651177
H	5.70046830484172	8.85258774293834	20.09960486316843
C	4.12311763158670	9.69342643379797	18.78445050042384
H	3.43586532740588	10.20784434909912	19.45798485844122
C	3.98120083958845	9.62543814914174	17.34142268531907
C	2.86545938196761	10.10207615618794	16.62733856507177
C	2.71820437495646	10.00825566262213	15.22913533115417
C	1.51512775173625	10.37435269161193	14.50649494242345
H	0.60964380945730	10.77873573387451	14.96193497140485
C	1.73942689049920	10.09230022828297	13.18097411131183
H	1.05239672347080	10.22176512142084	12.34297833701934
C	3.08670170455535	9.56563184076282	13.08036583043007
C	3.70973267817038	9.18716541240943	11.87495804607372
C	9.29107666610886	6.80544131803160	12.26095393012008
C	10.58337102584134	7.31312733762433	12.53634758845138
H	10.69325250473702	8.12179925475876	13.26862075057695
C	11.71192288587611	6.79421872433297	11.89267638094074
H	12.71364658793161	7.18034790780447	12.11225449284661
C	11.57946863873216	5.75424574238222	10.94864120223681
C	10.29383338547401	5.25723995495504	10.64893037271700
H	10.18523549833018	4.44437385887393	9.92341072503629
C	9.16588521526627	5.77636349493080	11.29762532249908
H	8.18396526218090	5.34111724430366	11.08411245211024
C	7.62927028471055	7.27241646752408	19.05582431648983
C	8.91217329328203	7.72747692462547	19.43745130071999
H	9.43616373581005	8.44772219230093	18.79704479264774
C	9.51044363106768	7.27457249070047	20.62055269532226

H	10.50370334712521	7.63317000723646	20.91111906827277
C	8.83365140332987	6.35246383810323	21.44828805634908
C	7.55026399414549	5.89887897509695	21.07553673803049
H	7.03843158779540	5.18006449951668	21.72647189099113
C	6.95439660434843	6.35486345283004	19.89365704526001
H	5.96326799305693	5.98941316148995	19.59859046638374
C	1.72252449744133	10.66647771171729	17.40860531561447
C	1.35035065155278	12.02536458473700	17.28705566783482
H	1.92415323135524	12.68143200230431	16.62071934891450
C	0.26680027598073	12.53463027548147	18.01459305790881
H	-0.02340739292262	13.58902535045640	17.93154610347371
C	-0.47305298895098	11.69513256436961	18.87512156315353
C	-0.10812294934619	10.33662507799965	18.99940432293939
H	-0.68470955510965	9.68138935527386	19.66144321416486
C	0.97926765375625	9.83178931147579	18.27520284610301
H	1.25627815808166	8.77405171975090	18.36590450865177
C	2.94637096697782	9.33303094492528	10.59869225888995
C	2.54194018005729	10.60148734865777	10.12402901488904
H	2.79090553822093	11.49586842325597	10.70750314809785
C	1.85458836299714	10.72633228596848	8.90877930714522
H	1.55636291442967	11.71371242014030	8.54072672693163
C	1.55207821100210	9.57942777692148	8.14369120292450
C	1.94683162083197	8.30948366429798	8.61588876920892
H	1.70265029383389	7.42690375490460	8.01344370412752
C	2.63701010438740	8.18861557444698	9.82707979955705
H	2.94377220107836	7.20065488586068	10.19170586464903
C	0.83797941332667	9.64680034475710	6.83194849673264
O	0.57401868892566	8.67440620664168	6.13830617815658
O	0.51467756336802	10.91638270024023	6.48150709949583
C	9.42368641627931	5.82458497331342	22.71564344935626
O	8.86642707847193	5.03168141612560	23.46160359656867
O	10.66156292355966	6.32297220124055	22.95443604231765
C	12.81876554324925	5.20426634674027	10.32140732744594
O	13.95466547665306	5.58190303068649	10.57143121483210

O	12.54697174859255	4.21335633641598	9.43776867756258
C	-1.62650354911964	12.28255142334241	19.62377098364079
O	-1.97005116177347	13.45514644349894	19.57244612764919
O	-2.26601052574792	11.35689316862842	20.38067394646866
C	-3.39566339126453	11.83429733728770	21.13181819156372
H	-4.17234005700023	12.23259473528016	20.45614549051773
H	-3.77923099967214	10.96610592744822	21.68678632307067
H	-3.09154373886802	12.63377252377502	21.82916129568631
C	-0.16106601892189	11.06411854604991	5.21823299625200
H	0.46910095935339	10.68441825006670	4.39442438812380
H	-1.11558373324221	10.50865530148233	5.21949682699406
H	-0.34299708994722	12.14243449356695	5.09791504562410
C	13.69042594157539	3.60895696664983	8.81039742462010
H	14.27711123901727	4.36209023104381	8.25688543994844
H	14.34488697211539	3.13853998357615	9.56521204227312
H	13.29149993403934	2.85003562609111	8.12212722189304
C	11.30233531482179	5.85751128981536	24.15554924097645
H	12.28813712421656	6.34387669957934	24.17941163162049
H	10.71140516647040	6.13870947755361	25.04470696787738
H	11.41353736117945	4.76013868745073	24.13740892415478
C	3.45829961418127	6.54946301909260	15.96105059924331
H	3.94590729672666	6.54550486661451	16.94989982240296
H	2.64966685385910	7.30099892893855	15.97722437589939
C	4.44962473454559	6.78418245118297	14.83913842354171
H	3.97360728093231	6.72064412179095	13.84419600097001
H	5.29580748615291	6.07333539214069	14.88341796048429
Si	6.72530677945463	4.10168008994429	14.49118213388131
C	5.31194438036123	3.79909971600912	13.24898672030260
H	5.14913494451432	2.70514933082284	13.14526277986155
H	4.37725840661900	4.20635249004316	13.67941047483167
C	8.33379290688136	3.35046684632765	13.82891988440049
H	8.80998317693758	4.05069588581215	13.12377259676848
H	9.04713626065966	3.17094067451234	14.65317355427842
H	8.15293446211691	2.39338340043553	13.30200836433820

C	6.31944406109422	3.43574214382141	16.22243895517486
H	7.20114532171688	3.64825010472859	16.86500466598122
H	5.49173591487373	4.03393593148741	16.65451027838804
C	5.57994577951241	4.44107451868496	11.87303419720427
H	4.67040011124670	4.49843114022587	11.24485194742639
H	5.97911685850109	5.46776887091817	11.97771277477583
H	6.33512968614156	3.86685619327032	11.30101240408798
C	5.98565999737039	1.92995232214624	16.26520163032070
H	6.79728771836414	1.32497875610198	15.83373423271522
H	5.83182194950169	1.57385283532622	17.30643423995926
H	5.06337700816277	1.70077904568340	15.69699917600433
H	2.98258557876549	5.55725725010193	15.81478945236397

Rh-Et

Rh	5.67183558593493	8.48694306540139	14.88259754879939
N	6.17600917637315	8.28877948025274	12.91860172068558
N	7.53821499511213	7.85265688231019	15.42025495244306
N	5.21798777595551	8.79997500749798	16.84366977185680
N	3.85676870429871	9.24762352393364	14.33876792076671
C	5.38712077433095	8.60647522314474	11.82146131358685
C	6.13601161202784	8.38834234131937	10.59866620281196
H	5.76914725166753	8.60078714023053	9.59315577205303
C	7.36633375166958	7.90109685037214	10.96290261203172
H	8.20305648568194	7.63846916149609	10.31354992211668
C	7.37961227285820	7.82052307715135	12.41105124569080
C	8.46826780197674	7.34868226929365	13.16745162794200
C	8.51808302749961	7.35433047661664	14.57332611979578
C	9.61650351492254	6.81840966741362	15.35393868453761
H	10.50673407769967	6.34208075470008	14.94004766252205
C	9.30324340950299	7.01600549576498	16.67582693859672
H	9.88635222519482	6.73228188713884	17.55343240799823
C	8.00903738033669	7.66842885785985	16.71190798277632
C	7.33410998780995	8.01888901896027	17.89552505945936
C	6.03953286391874	8.56775478153389	17.93768757939466

C	5.36378997183317	8.97629322210875	19.15449680444635
H	5.80025376758472	8.94341719334169	20.15410555266433
C	4.11679792259437	9.42027848912283	18.79163496362894
H	3.33375286665378	9.82274091631626	19.43635045298993
C	4.02267351145824	9.28953480552466	17.34991553361226
C	2.88287220518285	9.62807674734918	16.59735063525219
C	2.81751890901972	9.58978615768283	15.19204618382190
C	1.62850320119595	9.89545014722128	14.42009027077444
H	0.66130515686426	10.17339399191813	14.84195457808514
C	1.96018118375454	9.74729415319658	13.09630863785860
H	1.31662059295709	9.87995824755399	12.22512402273087
C	3.35288443715405	9.34535098322256	13.05003454294574
C	4.06124608130524	9.07573312835612	11.86453248459322
C	9.65201855459468	6.81860162977219	12.42771088904019
C	10.90289550311963	7.47222649353517	12.51057700310548
H	10.99310660000790	8.38013873452832	13.11886541848692
C	12.01122677296146	6.97766048853809	11.81440338947508
H	12.98351497675418	7.48112474011058	11.86647351769339
C	11.89388015424708	5.81676086106845	11.02158094214911
C	10.64888644734419	5.15799627505659	10.93443903803514
H	10.55876276434338	4.25369394576622	10.32355990496672
C	9.54046721233178	5.65724712837609	11.63018940944227
H	8.57467145449419	5.14143973750498	11.56872529223653
C	8.03220951895254	7.77848914692257	19.19351448113352
C	9.20361519634747	8.49648048890053	19.52345263287490
H	9.59070456208378	9.24227618620608	18.81869234033564
C	9.86008671004864	8.27297816864870	20.74043691187601
H	10.76312859758713	8.83765761840198	20.99486689420178
C	9.35657196385108	7.31961637297299	21.65100247078916
C	8.18927228623481	6.59748232463881	21.32578796049628
H	7.81485887990612	5.85563477546819	22.04076988529999
C	7.53311892339800	6.82631630563264	20.11123343678483
H	6.63047381276266	6.25808050881378	19.85640005716589
C	1.66289872090598	10.05990923861265	17.34257365973051

C	1.16724229257171	11.37746308137111	17.21433019718884
H	1.69430064892547	12.08764347732418	16.56599619000595
C	0.02582071005973	11.77879195162800	17.91741114158323
H	-0.36241541585353	12.80033199782352	17.83123296582377
C	-0.64674033222750	10.87024056483389	18.76130805778227
C	-0.15858804792633	9.55275440344540	18.89355858456655
H	-0.68346643592839	8.84590085371205	19.54461586522662
C	0.98598916069215	9.15504605086510	18.19125967180341
H	1.36218575190441	8.12947006122263	18.28770556269059
C	3.35729807369467	9.29925352295182	10.56680782096963
C	2.95900153567905	10.59939965109646	10.18152002418319
H	3.18293680405589	11.44522382812256	10.84258039142067
C	2.29809096543403	10.81408818223147	8.96553814284807
H	1.99785704919348	11.82437660679142	8.66844579192084
C	2.01929159664557	9.72650710091730	8.11049210639642
C	2.41329778072370	8.42621074808114	8.48988037455399
H	2.18426912267294	7.59131850479016	7.81748606659774
C	3.07638455624998	8.21539785815358	9.70393649331013
H	3.37398019987848	7.20280800000435	10.00162669536404
C	1.31548306740101	9.89303920231790	6.80380814107578
O	1.05697292400932	8.97954332039274	6.03289573666842
O	0.98754607322197	11.18480051024796	6.55899348187515
C	10.01344529308863	7.03714737146862	22.96230779930418
O	9.61367620296446	6.21721555235856	23.77685284888704
O	11.11680753210162	7.79909645063125	23.15760856362703
C	13.10991290004729	5.33417442915911	10.30111925036596
O	14.20702763965581	5.87193438735067	10.35358657695786
O	12.86489877463898	4.21966395820506	9.57028043118426
C	-1.86004539288952	11.35293123648791	19.48626606101960
O	-2.31738784698719	12.48368300397731	19.39841413052986
O	-2.40907179261624	10.38960365306434	20.26525533819221
C	-3.58317612231963	10.77625540302853	21.00028993249956
H	-4.38993110174942	11.08399903331010	20.31232872326591
H	-3.88631119443285	9.88928049984230	21.57508804836379

H	-3.35824523807001	11.61749896085109	21.67879864823803
C	0.30454894804480	11.42975462903172	5.31730708945792
H	0.92974667543712	11.12138564172423	4.46123127755138
H	-0.64647334878447	10.87044972570315	5.27903323429490
H	0.11525923036711	12.51238362042790	5.28431384244797
C	13.98823084055735	3.69133297528281	8.84416070650899
H	14.37847568645336	4.43914166655596	8.13197781925863
H	14.79990343513355	3.40595039174005	9.53599115041205
H	13.61333099056056	2.80804274852829	8.30725043357086
C	11.80807836804242	7.58834812908643	24.40092284128206
H	12.65853788314515	8.28527817095826	24.39651401906752
H	11.14289168144341	7.79680670049794	25.25696835885533
H	12.16450671192341	6.54618280157989	24.47679655605981
C	4.78112423534674	6.63524987559428	14.93619586998508
H	4.57921652058491	6.40177677199209	13.87614057532688
H	3.82311135496156	6.81795038585367	15.45436496282096
C	5.59560509876864	5.55474964149236	15.62181893846337
H	6.54072918892571	5.33942645406389	15.09420658581470
H	5.83776976000027	5.81750302589349	16.66642378546186
H	5.00499556997148	4.61536956115106	15.64610794247965

6⁺

Rh	5.82889612346930	8.00423423522202	15.09981211779556
N	5.97652091085803	7.90020145527396	13.06047827859036
N	7.84322669836203	7.69605371181227	15.24433966164461
N	5.77667120893098	8.52886808796337	17.09068958733361
N	3.87975644963276	8.59592034153135	14.90943044704060
C	4.92934740584830	8.00411849726338	12.14541566274484
C	5.40313933752149	7.65107675789509	10.82807191850483
H	4.78674347571178	7.63079232246977	9.92821228668011
C	6.74105245015877	7.35359886940482	10.94109344175394
H	7.42744623011028	7.04453685106600	10.15136724850018
C	7.10125127974051	7.52101529132442	12.32879696030189
C	8.39291698463258	7.31768857109813	12.84420231813040

C	8.72478053400748	7.43139420883438	14.20602512823291
C	10.06788993605770	7.29945858599003	14.73018294990291
H	10.96008272721632	7.13644493947202	14.12400197466158
C	9.98807276872977	7.45521200433546	16.09158850526186
H	10.80258231918954	7.44525240842787	16.81717567442459
C	8.59603192712862	7.69111167471093	16.41260622048604
C	8.11386899009911	7.91953839105920	17.71387273389299
C	6.79207498861886	8.29348449471324	18.01474954434738
C	6.27826652585010	8.48610774477352	19.35075691467969
H	6.85939293530802	8.36383938744394	20.26591383546943
C	4.95226776236391	8.82861940238770	19.23290322715845
H	4.24062279719714	9.03770606211037	20.03285780612886
C	4.63571835248870	8.84208059161025	17.82356469610845
C	3.35317220932456	9.08300987918522	17.29856071018287
C	3.02743572330289	8.99017481532468	15.93445955400249
C	1.73745625683599	9.34898159597662	15.38375536330294
H	0.89744139202713	9.73487537053541	15.96311298444240
C	1.81104187460098	9.15988146868366	14.02637041708061
H	1.04293622265951	9.36057584490336	13.27797883144122
C	3.14536301018012	8.67963457970108	13.73420956402783
C	3.60845830421844	8.38095414620059	12.44047781259646
C	9.47739005291596	6.93926296343945	11.89144008620002
C	9.90751840536260	7.84526860172680	10.89673491597514
H	9.44587878097192	8.83815740228137	10.83567731799052
C	10.92937379597264	7.48956599593274	10.00672355908008
H	11.26926971722125	8.19606153711046	9.24239773776452
C	11.53139411599660	6.21701581841845	10.09463469050399
C	11.10230580820337	5.30895722818585	11.08410301645911
H	11.57979491870314	4.32350370548358	11.13324762937367
C	10.08714120820390	5.66742358071174	11.97790602312378
H	9.75295156051883	4.95855944332035	12.74468637952322
C	9.07399845918464	7.76119209553772	18.84486869800713
C	9.43261026025963	8.86935938517425	19.64432629597511
H	9.00609959467532	9.85515230988605	19.42299993811039

C	10.34297390472601	8.71872789364159	20.69825480760909
H	10.62918842191866	9.57992984914181	21.31082535880700
C	10.90307481088660	7.45362894962724	20.97272953489383
C	10.54555266168483	6.34480944479919	20.17859544944327
H	10.98861757986164	5.36934184357056	20.41020344058683
C	9.64201605454772	6.49694200155597	19.12096687890548
H	9.36060875694781	5.63348338468270	18.50615197892614
C	2.25722215098243	9.41428785192317	18.25485299032266
C	2.29390092859551	10.60433006943995	19.01474829687581
H	3.13141400418641	11.30087533567890	18.88757701451881
C	1.25934515406128	10.90594009769121	19.91013912612733
H	1.28246598166126	11.83388944635604	20.49084553753574
C	0.17591780349781	10.01585814482006	20.06423538101318
C	0.13832389590926	8.82510841891160	19.31021746630960
H	-0.70941056838853	8.14400344732113	19.44768308878216
C	1.16744796781944	8.52781382989026	18.41024007911213
H	1.14071418211272	7.59925826690534	17.82767359276650
C	2.63127408754420	8.44558017921213	11.31449437000411
C	2.78676968900069	9.39325757032457	10.27868844718071
H	3.62347317935379	10.10131357946244	10.31585033646876
C	1.87153681997338	9.44339959629944	9.21914974134512
H	1.98761727316152	10.18332941037942	8.42042828132167
C	0.78880930706189	8.54038201870686	9.17726418769351
C	0.62961116176134	7.59391296072427	10.20988316862008
H	-0.21579824779111	6.89803239663994	10.15838688898688
C	1.53984324797946	7.54868975773763	11.27169306469436
H	1.41826652686415	6.80707332341983	12.06996815194155
C	-0.21663490396880	8.53993699412589	8.06763998374162
O	-1.16628618948643	7.77379325514094	8.00256382247076
O	0.04706001722898	9.48713952027913	7.14111362635954
C	11.87999270347610	7.23121343790785	22.08565258935469
O	12.38726245934289	6.15109026106329	22.34820945264457
O	12.13887726469095	8.36717986872580	22.76969513805762
C	12.62660094941110	5.77915413575544	9.17216062216647

O	13.17154645001479	4.68675285145480	9.22381415738250
O	12.94615634329967	6.73467811036736	8.27215555669961
C	-0.95811742142863	10.28306987083716	21.00488831969652
O	-1.91352842486799	9.53675368854898	21.15730435666992
O	-0.80501834648632	11.44967437817650	21.66880117948208
C	-1.85742557846825	11.78816282042947	22.59266827304669
H	-1.94287343969080	11.02035677980117	23.38088413500066
H	-1.57647836413411	12.75750919879393	23.02820470778065
H	-2.82424752107290	11.86603011709241	22.06625138200527
C	-0.87309048986219	9.55511754313060	6.03454024097740
H	-1.89427452089733	9.77138413643544	6.39287465518059
H	-0.51074868582066	10.36837748371200	5.38990431763257
H	-0.88519335463940	8.59973249887292	5.48221229125023
C	13.99774337543074	6.39863422065060	7.34653850786502
H	13.71877516384044	5.51509382638645	6.74685877427589
H	14.12347638192697	7.27871829436950	6.70008689268860
H	14.93432337803556	6.17957953188897	7.88770766877001
C	13.07502316053104	8.24494928143786	23.85795217364054
H	12.70473154910631	7.52540146271245	24.60833994713256
H	14.05565971150378	7.89941869280841	23.48784890524123
H	13.16096835392468	9.24926426287297	24.29638035149861
H	5.55694672438237	6.82048612168646	16.06080726278577
Si	5.24709828678424	5.54719740113212	14.85124276410613
C	4.83009191769788	4.85727752054678	16.57885292588002
H	4.73069022687710	3.77367855590084	16.34505693773182
H	5.70264472767587	4.93849460511369	17.25326171657199
C	3.73684678902004	5.39312387717877	13.77450935077387
H	3.93903418965004	5.63532636627151	12.71978312608710
H	3.43313704186949	4.32992568490934	13.83160989430935
H	2.89623351158384	6.00876397786582	14.13121588164301
C	6.72800415334652	4.62765385175861	14.17281697178714
H	6.27715170072224	3.70828167201031	13.74203098303281
H	7.14815316786798	5.17277378307775	13.31009164434604
C	3.55815487856906	5.39335357033139	17.24803713555653

H	3.69617128983348	6.42862431770679	17.60181087904279
H	2.69415141173218	5.39004715997694	16.56056554764540
H	3.28588264377542	4.78283955661915	18.12722128542216
C	7.82270372276985	4.25796429425992	15.18605605729465
H	8.65606060693444	3.72805213654546	14.69114005861523
H	8.24765844907300	5.14780680509796	15.67986743141788
H	7.43718007347981	3.59216824788052	15.97780460039443

Rh(I)-**3** (the single electron reduction product of Rh(II)-**3**)

Rh	6.04345551199744	8.51555870359676	14.95882653939353
N	6.55643381613664	8.32555673825302	13.00437516378846
N	7.92915239483718	7.99885695267125	15.50436389018222
N	5.54327581252333	8.75182792066203	16.91161853618955
N	4.14630667796931	8.98760474680388	14.41574920752016
C	5.74785095820012	8.57049867399142	11.89780427215881
C	6.53053555299780	8.48073428994647	10.67968052179324
H	6.15276115841547	8.67142780434604	9.67346999591526
C	7.80922520020182	8.14086579663808	11.04857799766264
H	8.67745364197865	8.00031039685600	10.40204422489892
C	7.81599928438579	8.02301349260022	12.49439538038150
C	8.94140232145742	7.65671004619108	13.25604880520033
C	8.97070364093565	7.61886953036203	14.66243219982837
C	10.10681999031269	7.17193159872985	15.44603215965581
H	11.03945105102494	6.78071764030270	15.03515114974154
C	9.76423697804473	7.31740441243283	16.76818181942496
H	10.36259734751382	7.06757056476125	17.64630668708216
C	8.41678162061981	7.85410728414799	16.80026355732949
C	7.71789530974636	8.17641052985825	17.97845765859393
C	6.38548181712168	8.62828545941719	18.01325780659720
C	5.69001071762947	9.03444681042267	19.21970518549310
H	6.13200688874528	9.07606018223265	20.21695970757181
C	4.40960276127806	9.36853926062487	18.85141742214636
H	3.60446791650400	9.73676019445442	19.48992743166701
C	4.31485340770888	9.16755800696583	17.41781653092278

C	3.14360365272078	9.36699266987039	16.66283630250242
C	3.07177263390810	9.24643809116479	15.26234052672461
C	1.85077710474382	9.37921944878097	14.49021575003502
H	0.85567675829460	9.53965085695225	14.90922015441273
C	2.19515093921077	9.24070480530135	13.16779587529650
H	1.53534799851887	9.26547387296248	12.29852253474425
C	3.62834862090578	9.02053265115422	13.12387649048444
C	4.37170476005147	8.86328011670536	11.93914519283842
C	10.18480036630902	7.28182457293952	12.52265836790107
C	11.37273084328643	8.03584010574761	12.67356180299912
H	11.36566101121190	8.91171738227565	13.33320533165723
C	12.53737259577074	7.68411251005145	11.98317477769492
H	13.45721601534456	8.27106452923556	12.09019334452924
C	12.54713367932829	6.56392618296799	11.12391278673196
C	11.36707061716139	5.80357460962291	10.96727770404393
H	11.37270085622163	4.92963819600316	10.30722947541173
C	10.20345604966093	6.16209458392586	11.65794086185844
H	9.28982246685326	5.56673948130540	11.54312693563261
C	8.43497820648272	8.02695967035371	19.27776518151956
C	9.58662485433484	8.79759055746493	19.56330292678870
H	9.94195417289153	9.51589683524566	18.81486954240212
C	10.25916587579441	8.66398864365168	20.78381032307240
H	11.14392591644663	9.27280314418835	20.99828013707825
C	9.79453985395451	7.74715738494553	21.75270865617010
C	8.64775098693336	6.97167860507760	21.47527732482759
H	8.30200537306951	6.25801888256030	22.23235703210707
C	7.97753334507823	7.11191092553244	20.25569355741206
H	7.09182889599342	6.50297306414255	20.03901090057595
C	1.89633023485745	9.72793219304695	17.39656182576225
C	1.24213027216527	10.95964180130139	17.15559235047944
H	1.67242496379399	11.65498124624833	16.42508426605036
C	0.07327263610291	11.29768919762870	17.84554760454776
H	-0.43178502453342	12.25450025400097	17.66794062121197
C	-0.47597836112395	10.41052585514300	18.79664084504787

C	0.16817497676464	9.17803361786718	19.04394969937567
H	-0.25929585358885	8.48389159795029	19.77529195694465
C	1.33846222485101	8.84551121517428	18.35172843330537
H	1.83263469455066	7.88443431689208	18.53724850553960
C	3.65494769873778	9.02106605069952	10.64085393931237
C	3.04779509711099	10.25145721985541	10.29500013600009
H	3.11844409543751	11.09210891866032	10.99544759525833
C	2.37997237674166	10.40847219426899	9.07477941932443
H	1.92227326390122	11.36848645859467	8.81337109080334
C	2.30072397185669	9.33016707581157	8.16584011878761
C	2.90061407547153	8.09757255193961	8.50367105323041
H	2.82532265181157	7.26580397459338	7.79338013865184
C	3.56839231321452	7.94683409614224	9.72348187633749
H	4.02591476465723	6.98579661959549	9.98678606526194
C	1.60760151031477	9.43859020484617	6.85269571671849
O	1.51718376232288	8.53738630344025	6.02886586233784
O	1.07119627937144	10.67169388505650	6.65173290669854
C	10.46764543565807	7.56081304353237	23.06753755537301
O	10.10911447241001	6.77536051177673	23.93565954083213
O	11.54964183974321	8.37104614891883	23.21428295142338
C	13.81369744992342	6.23281620385522	10.41483679443794
O	14.85919618607422	6.86250340480037	10.51231800392668
O	13.69251316478551	5.13145659514874	9.62675591635093
C	-1.72106176361595	10.81932743875235	19.50312234247050
O	-2.31838574939040	11.87273905968080	19.32256257094719
O	-2.14064914171439	9.88241423027082	20.39474142680801
C	-3.33758931690209	10.20665138371179	21.11722748730203
H	-4.18832730275338	10.34392951799258	20.42635357544761
H	-3.52544332409326	9.35767443026019	21.79092658604326
H	-3.20555502844519	11.13655715901312	21.69820276595567
C	0.39051903848616	10.85501734958919	5.40167689220558
H	1.07767240380663	10.69147570069788	4.55260660301651
H	-0.45505225547313	10.15073666228198	5.30750816761464
H	0.02536975703744	11.89261556141189	5.40380740091669

C	14.87584757669027	4.75014649539039	8.90950406349161
H	15.19329806060851	5.55348384464834	8.22116414733934
H	15.70615931128201	4.53848334538952	9.60621338448764
H	14.60999998687604	3.84512582885625	8.34367524690417
C	12.25050973579461	8.24790578394479	24.46070068532593
H	13.08015871714954	8.96869296861493	24.41537676262206
H	11.58466146960807	8.48123636700854	25.31039084824171
H	12.63946392289612	7.22265719829579	24.59283995493501

9^{-} (single electron reduction product of **9**)

Rh	6.02895409065685	8.47013534654238	14.95536024433478
N	6.56078419374246	8.38063258480106	12.98437333271612
N	7.96009083621003	8.04710238961182	15.50476085757277
N	5.55776182482237	8.78997465263740	16.92131080656481
N	4.14005425846258	9.05695416339249	14.40489420892178
C	5.73675509770659	8.58826697243885	11.89111725492084
C	6.52482491030321	8.54600265383123	10.67273840411896
H	6.13894008513013	8.73133170726870	9.66899958421951
C	7.82199039578295	8.27475955930123	11.04126091419459
H	8.69647739520028	8.18471447416580	10.39421489606735
C	7.83590747227626	8.14232450781769	12.48189089293518
C	8.96557726516579	7.78957037525104	13.24184408053311
C	9.00112024321995	7.71504220657706	14.64753816978500
C	10.12947240460744	7.23676514063881	15.41496844500262
H	11.06800903429517	6.87320629272090	14.99247080530796
C	9.76870270803072	7.29448301806072	16.74179017191194
H	10.35850206150831	6.99161726004724	17.60825611831394
C	8.42238996660916	7.83202078914854	16.79160900033813
C	7.71271397646659	8.11595094093781	17.98022799444743
C	6.39568666362469	8.62801263529852	18.01133311943046
C	5.72352899304082	9.08334643324505	19.21354356514459
H	6.17282549200162	9.12196561202355	20.20730233880660
C	4.45762194204330	9.47573596878369	18.84457124468951
H	3.67380397122515	9.89080387896479	19.48081261123143

C	4.34641119259899	9.26179556492819	17.41776030773536
C	3.17874548478959	9.48574418062842	16.66414573602759
C	3.08639050839141	9.34884042412594	15.26518114043387
C	1.86340913872287	9.48407781902259	14.50622786526685
H	0.87731451781827	9.67902640432221	14.93161725631393
C	2.18696100207935	9.29036881826151	13.18211027069561
H	1.51377941365590	9.29732233673842	12.32350297634327
C	3.61439859915135	9.04670111597827	13.12442175074591
C	4.34677810051854	8.83683886728571	11.93269307500001
C	10.21608976669964	7.45204534897176	12.50205807412578
C	11.38542713377933	8.23200591842650	12.65925893792448
H	11.35978599670002	9.10068759676243	13.32785563122374
C	12.55588541468704	7.91194149132284	11.96272313922013
H	13.46328661579332	8.51744861226979	12.07196728635374
C	12.58733583856486	6.79869136347376	11.09537059463211
C	11.42546035554266	6.01175488371861	10.93596683858025
H	11.45021906143338	5.14250323899109	10.27037753251002
C	10.25496624880152	6.33847159447118	11.63123255241248
H	9.35489910459414	5.72250886888646	11.51897495485174
C	8.39875383471252	7.89963153376017	19.28177092217178
C	9.61688612948029	8.55629600077254	19.58704353477260
H	10.04956852067373	9.24181529146104	18.84871032889274
C	10.25437818521428	8.36229153248614	20.81690003853126
H	11.18817697626376	8.88736925857732	21.04473085164568
C	9.69082940010175	7.49698636210864	21.78250984451811
C	8.47740375083877	6.83573918720986	21.48725777389108
H	8.05338003502038	6.15871963409945	22.23822791722323
C	7.84204874978217	7.03627108617393	20.25864928700348
H	6.90731358932820	6.51056543949523	20.03001264112227
C	1.94581075644112	9.88395794229099	17.40345577522980
C	1.32554102898197	11.13212706686068	17.16070389775590
H	1.77079414187074	11.81326241590698	16.42580322348921
C	0.16925756765064	11.50295650159275	17.85557343492201
H	-0.31128009494050	12.47224571990475	17.67800689114516

C	-0.39896421096141	10.63204814954805	18.81016946517146
C	0.21193002516462	9.38315929427280	19.05784708775128
H	-0.23161621879495	8.70235993064917	19.79202497755626
C	1.37059371716092	9.01758865507238	18.36221147604271
H	1.83942136944847	8.04373164042469	18.54651833563686
C	3.62274266913103	8.91760196357059	10.63608564468777
C	2.91948565261896	10.08880993807835	10.25952514239870
H	2.92465206340516	10.95124530911050	10.93660651929829
C	2.24685911376129	10.16659431722676	9.03542883497559
H	1.71878187460742	11.08325719152815	8.75180834999627
C	2.25552735120876	9.06662483952453	8.14763153287644
C	2.95403869207749	7.89396537088024	8.51363335667643
H	2.94729955714823	7.04305399661469	7.82230510878281
C	3.62785665677511	7.82256306378032	9.73607073713132
H	4.15845034569505	6.90589352713521	10.01992113034783
C	1.56013065954429	9.09134288447302	6.83420549274279
O	1.54397856426144	8.16939702454346	6.02738660609123
O	0.92473865214251	10.27381889209867	6.60659118791152
C	10.32203523957212	7.25309968216913	23.10572513307613
O	9.87310274988802	6.51675070117557	23.97621225120974
O	11.48164805768624	7.94921952998257	23.26274224192754
C	13.86028938532892	6.50113784352316	10.38076481360138
O	14.89085963123144	7.15408514642859	10.48178787027030
O	13.76164789267325	5.40393035633311	9.58541331634326
C	-1.63122220749076	11.07540628773880	19.52052136989026
O	-2.19769151704860	12.14564119164286	19.34123590077327
O	-2.07482136041144	10.14979228061582	20.41073943935838
C	-3.26157368263228	10.50525614338896	21.13624808027977
H	-4.10794132998246	10.67106993425956	20.44643581737134
H	-3.47348890002228	9.65845991442641	21.80541221562781
H	-3.10132755122015	11.42761545177288	21.72204323092031
C	0.23503058679821	10.37346295405013	5.35263084997139
H	0.93303271032347	10.23711581858771	4.50753751829440
H	-0.55746470861917	9.60764259198667	5.27727059999317

H	-0.20479363369373	11.38146412316020	5.32666864387808
C	14.95167811795025	5.05190149182871	8.86368566462955
H	15.24939590451816	5.86514377653821	8.17821267275618
H	15.78771683009909	4.85556693775113	9.55791484616131
H	14.70429358371625	4.14379756433937	8.29455926454461
C	12.14698743324479	7.76617592280938	24.52044820137487
H	13.04454769044696	8.40125397056556	24.48524153814537
H	11.49459884943682	8.06908094581854	25.35870017348126
H	12.43145926438679	6.70861914677521	24.66475162021626
O	5.47949099547192	6.53183721322993	15.02219907562179
O	6.33661848607891	5.66028646577700	14.60129930998502

10

Rh	6.04284988999713	8.51693219456467	14.95230730210820
N	6.57944277109945	8.43653073484470	12.98556263872885
N	7.97327219941595	8.09903269341844	15.49958474007571
N	5.56034179490409	8.81275408231251	16.91614068034292
N	4.15337304333602	9.07651106059881	14.40757337520728
C	5.76135211730952	8.66312575068315	11.89108508458679
C	6.53930279148820	8.55887290188920	10.67069672533522
H	6.15421790869972	8.73789642947929	9.66556894903431
C	7.81807748249057	8.22169842369613	11.03796013532619
H	8.68429524019893	8.07145925812285	10.39156234691056
C	7.83192906353612	8.12022962697344	12.48502693545367
C	8.95648653619496	7.74307292744094	13.24214189565506
C	9.00082029794651	7.72052359188917	14.64908299659458
C	10.14208469457022	7.28420354269823	15.42861969625263
H	11.07994829483907	6.91256524611835	15.01242330616072
C	9.80144487434527	7.41980267276014	16.75289913139904
H	10.40633227387510	7.17983854977265	17.62878413254318
C	8.44841745944743	7.93907250885421	16.79270651019796
C	7.73895844853064	8.22879061934020	17.97370315200973
C	6.40346372689935	8.67262256973996	18.00808777254576
C	5.70002840025289	9.05217360843908	19.21843420883614

H	6.14148498590190	9.08208649573072	20.21586119334828
C	4.41800240891824	9.37943487143884	18.85264737017962
H	3.60625239644894	9.72890536433380	19.49266440458211
C	4.32757188988128	9.20240102001100	17.41615443752502
C	3.15449336432224	9.40163244050263	16.66377908850283
C	3.08605819101610	9.31182731316497	15.26085545457296
C	1.86860879759784	9.46580932215601	14.48850290234133
H	0.87663932142437	9.63614537293380	14.90999611750384
C	2.21263500939432	9.33892338837284	13.16550441833014
H	1.55693461845502	9.38542721710163	12.29457934113690
C	3.64294086685056	9.10547796761776	13.11904939940541
C	4.38253760176952	8.94166948148312	11.93311995282222
C	10.18522293023946	7.33305158227562	12.50014418385732
C	11.37120733984601	8.09684988003823	12.58666847151279
H	11.38081254374711	9.00685940986258	13.19859392476073
C	12.51864511095232	7.70793335821257	11.88637546036291
H	13.44114707525611	8.29768240280858	11.93983645310015
C	12.50602780739226	6.54449344617141	11.08893059947472
C	11.32598240663095	5.77567394247780	10.99972230231382
H	11.31718538980787	4.86943307593287	10.38508980092838
C	10.17740878464696	6.16968785428558	11.69781205336149
H	9.26126574643507	5.57005635639504	11.63536510257246
C	8.44763083687460	8.05439142194944	19.27614529239498
C	9.57025944649264	8.85064876997545	19.59742771295830
H	9.91294091696424	9.60701566815967	18.88109997999748
C	10.23156870132242	8.69322251564476	20.82201980199391
H	11.09517423222896	9.31923097181585	21.06973599545931
C	9.78247926491064	7.72898291962549	21.74936794621256
C	8.66444811087340	6.92907338813270	21.43321149543730
H	8.33210229875453	6.18071355521094	22.16201445593045
C	8.00292819703918	7.09156842332057	20.21102722473867
H	7.13818799943626	6.46409455401227	19.96404048664881
C	1.90263937089346	9.73722147720940	17.40526357352217
C	1.27588632477031	10.99173635605075	17.22731602718124

H	1.72267939471609	11.72225824711562	16.54231441337514
C	0.10679274767106	11.30733937373526	17.92842700553640
H	-0.38302897463138	12.28015758245428	17.80438024884467
C	-0.46254454459672	10.37392880028626	18.81970346988172
C	0.15578628709663	9.11841135047091	19.00027485728717
H	-0.28997506736730	8.39127585231766	19.68697560748563
C	1.32802786680728	8.80646641952073	18.30015239680351
H	1.80452726579153	7.82781077768443	18.43314087561631
C	3.65984286798554	9.06953188458371	10.63283477093096
C	3.09144574665553	10.30342033095341	10.24374372809477
H	3.19076480351685	11.17141917992267	10.90656233465476
C	2.42241950619794	10.42753210446451	9.01943442853557
H	1.99142604663553	11.38801370989854	8.71820159056628
C	2.30649915496078	9.31343116249575	8.16110655445100
C	2.86822434573518	8.07815276429260	8.54623280989507
H	2.76335395307638	7.22098284083157	7.87104510405562
C	3.53936383579367	7.95773401363603	9.76816579421049
H	3.96888353928781	6.99501958233776	10.07006733095202
C	1.60764469975946	9.38534144890387	6.84290102085995
O	1.49057770384306	8.44730048597621	6.06720713794513
O	1.11098365288606	10.62093420803137	6.59274506624855
C	10.44644284830408	7.51522585513895	23.07029303901550
O	10.08965402406575	6.69181939310814	23.90101649551692
O	11.50061089593198	8.34606379588331	23.25458103886429
C	13.76034432082392	6.17759199878769	10.36539225154701
O	14.80180860909427	6.81664660340225	10.41456127442234
O	13.61963004413297	5.04364530837240	9.63758973887124
C	-1.71044411354451	10.76514901195816	19.54147630578789
O	-2.27909954413283	11.83998594842517	19.41189834469140
O	-2.15312353197853	9.78655577574785	20.36735215880505
C	-3.35301599928361	10.08570677069220	21.10167096524456
H	-4.19295067573295	10.28162619241373	20.41251424814794
H	-3.56044609409822	9.19851429233306	21.71718472806006
H	-3.20684476181519	10.97358532376109	21.74118667314454

C	0.42689180026316	10.77548295761867	5.33729546714331
H	1.10517780336189	10.55586473085253	4.49436750538170
H	-0.44006098088042	10.09445181035454	5.27886296915902
H	0.09520067275895	11.82317093892105	5.30028250328420
C	14.78521080451596	4.62373055962808	8.90683065313465
H	15.08303021669624	5.39393995784365	8.17409066151225
H	15.63108632003333	4.44341895916874	9.59279503947640
H	14.50160394151262	3.69412383430073	8.39268596441032
C	12.19344240652575	8.20545704557594	24.50711581862412
H	13.00552071226546	8.94651092284817	24.48870520557224
H	11.51199806176064	8.40108234592837	25.35334612400554
H	12.60392531407953	7.18610021184222	24.61291231283550
O	5.49519624226986	6.63055746568843	15.04973948358006
O	6.39613433627434	5.76291184789755	14.40163152336253
H	7.05984438810208	5.58247308354293	15.10213956989295

10^{-} (single electron reduction product of **10**)

Rh	6.02950831336871	8.49946867108928	14.95736567932213
N	6.56767732292786	8.36685529394216	12.98973482004446
N	7.95924253647826	8.05007682852193	15.50182947007509
N	5.54934867493222	8.80777924512074	16.91444021149545
N	4.14263273104600	9.03800627682898	14.41070499785432
C	5.75387951270817	8.61767884509424	11.89409381719702
C	6.53833018464503	8.55606533696370	10.67458879352457
H	6.15949178029616	8.75526043287525	9.67059565284923
C	7.82026367758427	8.22741428795537	11.04266787231007
H	8.69232903971645	8.11466424098711	10.39617417545740
C	7.82635258919806	8.08532813272400	12.48803733210453
C	8.96187489048593	7.72536794858712	13.24547803543057
C	8.99362843077114	7.68241575114411	14.65733015983216
C	10.12711097564729	7.23030650836417	15.44219805912653
H	11.06204812523137	6.84383768726271	15.03272588339745
C	9.78185401791602	7.36639102200471	16.76647678004258
H	10.37855312802715	7.10950757690877	17.64344434942810

C	8.43328292002291	7.89912332947404	16.79776258928151
C	7.72029310780073	8.19996796237572	17.97463521939361
C	6.38986956037495	8.66450362232156	18.00939358027836
C	5.70039858523870	9.08120891093579	19.21613185027793
H	6.14478218324632	9.11988878494536	20.21236970633396
C	4.42549268571674	9.43818653289074	18.84817991441000
H	3.62580941850312	9.81732559558760	19.48691647152582
C	4.32657056598363	9.23415833924368	17.41507844873585
C	3.15176572910079	9.42495992385049	16.66079644922927
C	3.07436567180600	9.29671785248139	15.26024008154097
C	1.85060846910026	9.41637662962662	14.48994043161235
H	0.85610943114256	9.57818900125990	14.90936855948506
C	2.19190358912145	9.25574620875729	13.16857666688026
H	1.52976271666387	9.26770494384893	12.30097347339766
C	3.62540189157627	9.04037318574364	13.12316950176936
C	4.37185917063508	8.88143029940088	11.93815550183148
C	10.19499750487179	7.34239368301552	12.50856988357009
C	11.41537804444603	8.03558954714531	12.70596064187201
H	11.43879316917505	8.88303958140983	13.40149238957637
C	12.57068376517237	7.66812366533636	12.01037836932956
H	13.51208657284320	8.21214721394610	12.15165512999963
C	12.54491237800877	6.58985675350232	11.09701163398983
C	11.33337395262747	5.88905535074634	10.89475377169054
H	11.30981917080616	5.04527489160824	10.19686582730237
C	10.17871160218011	6.26336626531416	11.58964261540068
H	9.24406917834354	5.70872367537070	11.44378813158772
C	8.42308929882847	8.02409626697024	19.27822217945054
C	9.57922150308697	8.77895681396192	19.58710134953706
H	9.94990972539354	9.50577338861042	18.85452336195989
C	10.23638299659004	8.62018467889675	20.81297039681454
H	11.12484779743431	9.21654525041350	21.04624074396909
C	9.75095146468246	7.69480689082655	21.76310048879078
C	8.59902947965232	6.93572874478510	21.46208888094761
H	8.23693302074879	6.21519076879344	22.20482056882514

C	7.94384754831344	7.10047948763484	20.23738278668597
H	7.05438815117051	6.50389805229764	20.00220249799453
C	1.90490375276070	9.78151916830956	17.39706244988311
C	1.24021769836307	11.00627827711574	17.15028512778737
H	1.66114489025211	11.70084917533715	16.41359135540580
C	0.07164816261706	11.33883632381448	17.84341217810685
H	-0.44210304414844	12.29029786151543	17.66222439961195
C	-0.46623851819932	10.45268893716336	18.80185350386660
C	0.18854692381085	9.22677896345387	19.05380446550428
H	-0.23072757335823	8.53307717584042	19.79022606593898
C	1.35901071314625	8.89955943424940	18.35938996724463
H	1.86088116052737	7.94294147485425	18.54716664610942
C	3.65245966370270	9.01413055644589	10.63853169132454
C	3.02006832004810	10.22778265638158	10.28017926712332
H	3.07181617732014	11.07739224475031	10.97143756978367
C	2.35201132418266	10.35786068963050	9.05685234186963
H	1.87473802587641	11.30509744185599	8.78427944142409
C	2.29792911988089	9.26822265213719	8.15960130333091
C	2.92342127782568	8.05235644755693	8.51087046424368
H	2.86739704458642	7.21189562004890	7.80915430113536
C	3.59224155785208	7.92809069395052	9.73317577926968
H	4.06935859515190	6.98003905843929	10.00835761186506
C	1.60516261555527	9.34811237237009	6.84377661795732
O	1.53752369455574	8.43704794378351	6.02884125569657
O	1.04135796205343	10.56649769230141	6.62996960690140
C	10.40711544826167	7.48353906765956	23.08307873463333
O	10.02925383221955	6.69094160715304	23.93626396581802
O	11.49592154653626	8.27959568623982	23.25230897601442
C	13.80164984253802	6.24061473199539	10.38565353762364
O	14.87405628258582	6.81802120844585	10.52325826186209
O	13.64280400259259	5.18501809585555	9.54024190742266
C	-1.71225541531450	10.85555370957629	19.51076424069362
O	-2.31889788385784	11.90271374181565	19.32479107040126
O	-2.12030381785194	9.92086542667698	20.40931318464822

C	-3.31829847230058	10.23818359406332	21.13354995700203
H	-4.17161971496153	10.36537819982861	20.44401634615935
H	-3.49725057500057	9.39046216644389	21.81118080784477
H	-3.19226072438156	11.17154309325009	21.71025839496392
C	0.35984509933761	10.72231008813957	5.37642276145960
H	1.05119574878624	10.55912935698608	4.53078013159501
H	-0.47374162539366	10.00285260307493	5.29034566079964
H	-0.02268462626562	11.75354421463695	5.36475424629280
C	14.81738250780987	4.78931529013353	8.81820983849665
H	15.18729432056868	5.61560038677338	8.18531050846385
H	15.62460945175470	4.49311203390135	9.51161337320582
H	14.51737037233348	3.93480614141051	8.19370437319607
C	12.18150484746385	8.13332246006416	24.50499069596561
H	13.01503963672979	8.85059335246804	24.48071666062512
H	11.50658434491273	8.35628178868734	25.35020093411607
H	12.56417098393850	7.10422392336429	24.62489251550587
O	5.56510204121213	6.39140595292723	15.04455345700895
O	6.54162321777452	5.57218334496648	14.39638903558317
H	7.29040432221953	5.62094533245226	15.02505557644364

O_2

O	0.00000000000000	0.00000000000000	-0.00753337489278
O	0.00000000000000	0.00000000000000	1.20753337489278

O_2^-

O	0.00000000000000	0.00000000000000	-0.07710705371013
O	0.00000000000000	0.00000000000000	1.27710705371013

$Zr_6O_4(\mu_3\text{-}OH)_4(\mu_1\text{-}OH)_6(OH_2)_6(OOCPh)_6$

Zr	-8.91585102984179	27.09350051730823	8.25227419976680
O	-10.25249008677396	25.78442787249973	7.15839704141020
O	-6.58459348536798	27.02803933315274	8.76012494695265
O	-8.82691763319620	27.54239865468296	10.42364633454318
O	-8.18720328485920	29.23709916892750	8.55537737248537

C	-11.17899656927913	25.84744090923640	6.28042987629013
C	-11.30253403214971	24.70944618714164	5.32460670895263
C	-10.37072722279235	23.65018978547161	5.36841384389492
H	-9.56862640391325	23.68810443929508	6.11405073124235
C	-10.48959429110019	22.57725494873877	4.47368318925490
H	-9.76631701159551	21.75331857379652	4.50695838162436
C	-11.53890892630652	22.55729682670524	3.53546186725304
O	-12.00077537970902	26.81576153767578	6.18337957253897
C	-12.35576368834977	24.68573074481047	4.38586556328791
H	-13.07350479158291	25.51376495121553	4.37788119010442
C	-12.47152030188293	23.61091326581721	3.49281198790930
H	-13.29056280008601	23.59027100106553	2.76355333070366
Zr	-9.88123415325167	30.58643385549366	8.00353912178323
O	-8.22916334939133	31.23997491946219	6.95133242332634
O	-10.24608682324985	28.61550952604131	7.09572726406242
Zr	-8.16229383647259	29.38299607167356	10.84895174138735
O	-7.45891640766479	31.53468480092873	10.63246660573599
O	-9.98050372206273	30.03038425442574	10.02548521154799
Zr	-12.51197982878905	28.30546671783751	7.74541141034041
O	-13.23222247056936	26.21961649350574	8.45508406418157
O	-7.98919241104673	27.20511378197375	6.48011775607225
O	-10.92909838658825	27.54410487148837	9.21313842605961
O	-6.05375109418079	31.48050421601758	8.54494554542177
O	-6.16273559782451	29.16251699366236	10.14317252306533
O	-14.45567291766180	28.47688080293547	7.32491615968976
Zr	-10.53141430845925	26.56516852986413	11.37685392034738
Zr	-12.11974288698265	30.19699415060682	10.60999637187257
O	-7.25790936384572	28.28689041373636	12.54245475120691
O	-9.97646775754006	32.59975922578031	8.77027539035937
O	-10.92354764919183	31.17162965605107	6.17796208003827
O	-8.99982080967029	30.34515868591754	12.56837968017815
O	-8.55925298258819	25.15121703709000	9.10103008152022
O	-12.52746104403876	29.60368500726564	5.88105434090644
O	-11.93958459229398	30.20114480259860	8.49550303354838

O	-11.77847280159733	28.30510574331679	11.68367693478402
O	-13.41036998692672	28.56868686249970	9.79958506495084
C	-8.27552220328578	24.69344375639864	10.27060633531535
C	-7.02003999874595	23.90217957278015	10.41754844318947
C	-6.26275195379821	23.55031080716183	9.27721324596145
H	-6.64214490711761	23.81641794424149	8.28349642543902
C	-5.04995608986236	22.86189201875118	9.42909179374234
H	-4.46324041877485	22.58585803259880	8.54464595227499
C	-4.58999005858153	22.52406269437864	10.71586103900063
O	-8.98372884079698	24.92547060831495	11.29268975488096
C	-6.55943044110879	23.55410080983635	11.70592848671085
H	-7.15625586551548	23.84118011662148	12.57779530120384
C	-5.34560089857234	22.86984204230392	11.85219842811311
H	-4.98665523067381	22.61183533541040	12.85539522259692
C	-10.50677906610279	32.94074000602786	9.89612624433549
C	-9.79981843057377	33.97533574374824	10.70778807407215
C	-8.59826480166200	34.56005694397711	10.24486201710793
H	-8.23915315248906	34.31540356450407	9.23835108580120
C	-7.87977658276741	35.42943016710800	11.07953875902946
H	-6.94279068330141	35.87635299990050	10.72620345274758
C	-8.36008465358834	35.72255211071658	12.37022681745836
O	-11.53319485812061	32.38967440545360	10.37551631576470
C	-10.28615032773988	34.28830683354410	11.99572012725310
H	-11.21630906937599	33.81914189108066	12.33136061575633
C	-9.56594261180304	35.15768550614652	12.82470180935324
H	-9.93449178002116	35.37672755034409	13.83342556802712
O	-13.95145752487132	32.06566611055352	8.23309674442577
O	-13.85356466695091	31.07769702219616	10.89204161133685
O	-11.20883678971089	30.80603624422919	12.53062318030969
O	-9.18316654011977	27.25712269069288	13.06448006402715
C	-7.91800180164030	27.37139904314046	13.14875177196327
C	-7.15166883539973	26.36404782101068	13.94139810025769
C	-7.84003721886099	25.34670977557477	14.63584957614268
H	-8.93424948782140	25.33476719840787	14.60699922600116

C	-7.12444681416200	24.35386778958924	15.32004051409767
H	-7.66304928167392	23.56147580438104	15.85340920186564
C	-5.71670023678725	24.37102968480494	15.31333050672924
C	-5.74068054362686	26.37819116589445	13.93864107690047
H	-5.22121378533584	27.16831793989068	13.38552185911874
C	-5.02622429567530	25.38316891914982	14.62091018067203
H	-3.92961110253725	25.39137680915937	14.60996208670501
O	-11.36993009893060	25.68843430920451	12.96429590591176
O	-11.74749863690609	25.30773880072933	10.33272934639649
C	-10.04770433506526	30.93152030752443	13.01844856080778
C	-9.86458418848179	31.84420745895006	14.18674349719623
C	-8.56626507491671	32.18825772231703	14.61667337763202
H	-7.70484736729468	31.73957840221895	14.10979284078778
C	-8.39528638473007	33.10860762899668	15.66069483532452
H	-7.38530264419974	33.38529769260759	15.98654935603669
C	-9.51940847547916	33.67673974820031	16.28817599527628
C	-10.99071683277305	32.41461831588299	14.81708635767047
H	-11.99053690679335	32.13816645529134	14.46334074086773
C	-10.81700967368986	33.32513785109771	15.86887558662137
H	-11.69225905035352	33.76712606155071	16.36023367296177
C	-11.95969468018760	30.69959759976733	5.58061414270148
C	-12.52313344129467	31.50080300901480	4.45210737448245
C	-11.93174192973627	32.72963192666783	4.08825284561884
H	-11.05348353663853	33.07809132513700	4.64293081058609
C	-12.47117883723762	33.48390365723769	3.03636006033697
H	-12.01177284575975	34.43957582788452	2.75578020063227
C	-13.60192675297242	33.01456428964313	2.34203240077663
C	-13.65729385101410	31.03414568223295	3.75396574719193
H	-14.10326509149719	30.07856993630955	4.05127684234536
C	-14.19396433363497	31.78932310954723	2.70163793502832
H	-15.07579783209730	31.42497354343215	2.16078716856723
H	-5.15728785528064	23.59171184175703	15.84562380974615
H	-7.79027003254713	36.39322923627211	13.02506923543261
H	-9.38495947027186	34.39565763324048	17.10599816241106

H	-14.02320919915424	33.60530583986240	1.51905427693809
H	-11.63158946578880	21.71651195710187	2.83657243449911
H	-3.63943961655570	21.98882699191032	10.83284673604949
H	-14.30229224651686	28.93104958671826	9.64023203991018
H	-7.45710306648903	29.40323329515918	7.93044210933535
H	-12.65447627259932	27.93461372576602	11.41027883028577
H	-10.05380395247116	28.54695501535806	6.14155364995268
H	-14.16578612309322	31.63760509182346	10.13863519781606
H	-11.06127467474731	25.96096278915002	13.84474531339143
H	-8.31700782879143	31.84946415626102	6.19953472982572
H	-7.01707201017843	27.21605085518172	6.47036173159281
H	-5.49407862654123	29.08016073003062	10.84658447448511
H	-6.34208275638806	26.23851900146548	9.27786196009841
H	-6.31200361959386	27.83911897657623	9.33373742244426
H	-7.99271895470341	32.32974872231927	10.82026054757982
H	-6.87244710078837	31.70102179649720	9.78684911182214
H	-5.84697092967437	30.56550846546667	8.86240366045708
H	-6.77675719040415	31.39508593203708	7.85153471416083
H	-14.68233186321583	28.95300344589336	6.50750861967213
H	-13.22062766843207	31.39793277422635	8.17017115929402
H	-13.47976486780528	32.91513688823435	8.20630781274389
H	-12.08260245214153	24.53193394102486	10.81538202625920
H	-10.58470415615619	28.40384463177805	9.58372314985148
H	-12.61365142117013	25.81534142435549	9.17284491709930
H	-14.04430855367927	26.44224002002083	8.94641133858833

$[Zr_6O_4(\mu_3\text{-}OH)_4(\mu_1\text{-}OH)_6(OH_2)_6(OOCPh)_6]^-$

Zr	-7.45573632845510	28.50939025097390	8.54230704005664
O	-7.69451717342044	27.05181067883890	6.83988120765246
O	-5.42686411658791	29.48154966576130	9.63700516623508
O	-7.78212714075245	28.54977849837796	10.64460504100661
O	-7.99842398358917	30.60931439652295	9.21243528243028
C	-8.65043779331003	26.24852853577801	6.60421494584911
C	-8.32027611375689	24.79017387382290	6.53032056958787

C	-6.99937111333332	24.37503142123813	6.80252997765705
H	-6.23800922091903	25.14152562422801	6.97745322290924
C	-6.69211917859109	23.01033063286056	6.89142784586250
H	-5.67095296211745	22.69897360814915	7.14403176711237
C	-7.70345942897868	22.05312767303665	6.68743819286519
O	-9.87736933559852	26.60891317288353	6.50271356924085
C	-9.33075074640327	23.82872983682921	6.32301607287594
H	-10.35420007707021	24.17452138261977	6.14505559131193
C	-9.01937094141737	22.46291162382555	6.39718256763283
H	-9.80661685223428	21.71341090680826	6.24778380194678
Zr	-10.13844529577243	31.04056144761695	8.49947369228100
O	-9.15833204725890	32.02092747338733	7.01945498722531
O	-9.34964997268872	29.16385976739171	7.52630188055575
Zr	-8.29404722100975	30.30639782455821	11.46691747181932
O	-6.75555843330236	33.00884494546717	9.27388166698326
O	-10.06760816386749	30.79674633982156	10.62612606545654
Zr	-10.95144301605930	27.54267511486033	8.22354517030259
O	-11.21839078910229	25.26722997536470	8.48466647937100
O	-6.34730491847229	29.34812093323874	7.08936264323858
O	-9.14426607632451	27.15984170467940	9.11026541491479
O	-9.43304621980822	33.15920158396561	9.32418275589642
O	-6.51486459138929	31.44544777651376	11.29825046392790
O	-13.04424771474276	27.03076042446317	8.46538041978051
Zr	-9.05332208863150	26.94828853176012	11.13731151985995
Zr	-11.71925717822457	29.48757008426630	11.17491496422155
O	-7.21510609708741	29.31577140101412	13.15824548770934
O	-11.91548510061615	32.24373118507577	8.97893368075257
O	-11.53308538100675	30.73454462432364	6.73562928863018
O	-9.18196880976745	31.56147707299782	13.03249166458917
O	-6.18149673803952	26.86298660430369	9.17403169066972
O	-11.98163601550059	28.53962312334534	6.48354802993321
O	-11.33964973001314	29.34929175936356	9.08323613596052
O	-9.85113609612012	28.76406036445054	12.19595653533191
O	-11.21851000117394	27.29770218369204	10.56313255658790

C	-6.40375725708699	25.76361059444007	9.81114009827781
C	-5.41366131635434	24.68553705601321	9.71928261593698
C	-4.22196551320698	24.87179889038228	8.96746140542588
H	-4.05879267709852	25.83884713237270	8.47904313302936
C	-3.28548246744591	23.83859216362012	8.86226077999021
H	-2.36765134813856	23.99458142058919	8.28070313755141
C	-3.51342884059104	22.59836485597565	9.49877660908338
O	-7.48552090153168	25.55582579262484	10.49908007427368
C	-5.64067416696494	23.43456229856221	10.35235401563387
H	-6.56486770036005	23.29383257483918	10.92403043765077
C	-4.69895529364481	22.40646196382223	10.24302366257052
H	-4.88592186169764	21.44365982441937	10.73591352060266
C	-12.89463194758968	32.12513611784155	9.78587635788357
C	-13.96214481860390	33.15504529573501	9.74454267787631
C	-13.82677329776265	34.28121113834401	8.89902352353147
H	-12.92045547752723	34.37296599570283	8.28985368963709
C	-14.82956125544741	35.25819521572013	8.86434337345267
H	-14.71677435213509	36.13276016985542	8.21135676227868
C	-15.97941279676644	35.12290663760507	9.66968214534383
O	-13.02539573733313	31.15594657271678	10.61875427883878
C	-15.11828261325064	33.01920547458223	10.54767470974623
H	-15.20479945636005	32.14207241947874	11.19899450932869
C	-16.11920205873175	33.99877077310857	10.50969933124195
H	-17.01338228341126	33.88987840178022	11.13627087204229
O	-13.73128816366862	28.62565228972255	10.28935320068673
O	-12.68563749423521	28.65674592143168	12.74699339483630
O	-11.37088385684304	31.01650872399236	12.94093690389819
O	-7.81025421159583	27.14094911196173	12.96511779866352
C	-6.89987901812141	28.06516474769046	13.01630013427189
C	-5.50060235897692	27.68806411327267	12.77951081890064
C	-5.15060284463977	26.32724171679124	12.55213277629288
H	-5.93048117757028	25.56501949785243	12.64052106504725
C	-3.84347873840335	25.98035495209551	12.19120753132520
H	-3.59979935183759	24.92927040814837	11.99498140265161

C	-2.85719681087399	26.97398055948602	12.03815475289389
C	-4.48746533807016	28.68135825448048	12.65753503315627
H	-4.75696273444488	29.72750351095670	12.83856211571265
C	-3.18757845776936	28.32871041597354	12.28328053203876
H	-2.42294885156949	29.10816359307166	12.17091311509962
O	-9.90905343445148	25.35785106611221	12.13386870522004
O	-9.66487852438823	23.83252806375577	9.97437127370847
C	-10.43757593915836	31.87065931974736	12.92206136737638
C	-10.77142340050462	33.29829859063459	12.64051251952387
C	-9.74673635413888	34.24944839240373	12.41810245474485
H	-8.70348136114072	33.94549479102343	12.56551311526447
C	-10.07112778272486	35.54809252394075	11.99890946348142
H	-9.27289242248624	36.27650037617565	11.80975661857074
C	-11.41991317990549	35.91250469225762	11.80933467724819
C	-12.12175888825347	33.67611911622996	12.46369894750233
H	-12.89713982022329	32.92041117462364	12.62524696522998
C	-12.44246712227836	34.97620080609806	12.05053643632987
H	-13.49177328107814	35.25251249990621	11.89076030334511
C	-12.33169171713552	29.77213338928490	6.52042648562094
C	-13.79032715905311	30.09459899959601	6.39719824311614
C	-14.23061229556732	31.38832272542787	6.74919035733404
H	-13.47666711316560	32.13552462597038	7.01672488291208
C	-15.60085794497134	31.68092693261209	6.79377266884977
H	-15.93235404699083	32.68025588995584	7.10222594900115
C	-16.54022315721933	30.68696449180237	6.45953088284658
C	-14.73463555700731	29.10270518531949	6.05728783861331
H	-14.37955131232742	28.10561488173099	5.76861468025706
C	-16.10560793549636	29.40160340015274	6.08198447578300
H	-16.83879915213512	28.63019670365637	5.81554239328702
H	-1.84123427000134	26.70019627945280	11.72861185723910
H	-16.76333956290147	35.89008434438050	9.64284054019722
H	-11.67206741985704	36.92352440758213	11.46628122977441
H	-17.61356709194941	30.91235870230588	6.49564077127569
H	-7.46870959431788	20.98390884771485	6.76643583077499

H	-2.77708047821326	21.78960326588372	9.41387541079308
H	-11.79152545459360	26.63990006209266	10.99543937222520
H	-7.45099950762925	31.37600064267960	8.90416042647353
H	-9.91505188931331	28.68986511026315	13.16520439931743
H	-9.25024873824172	29.17776503126379	6.55857637522407
H	-13.45642059658955	28.13530593229148	12.46324514659244
H	-9.57185122774084	25.04314060068800	12.98877173180773
H	-9.23598138194419	31.65630143457964	6.12253231509172
H	-5.62949110035567	29.91754787111141	7.41466313671730
H	-6.02373610836422	31.60789880900863	12.12198847232316
H	-5.13448635485832	28.74607230531658	10.21303443244675
H	-5.70271812801877	30.19756899697260	10.27630455311881
H	-6.46791025316578	32.52062801718603	10.11564396656341
H	-5.97987600427243	33.16956626834679	8.71368797600197
H	-8.44863535265557	33.27508331562368	9.22569666311442
H	-9.64468326671970	33.29447492509249	10.26893384105395
H	-13.51575967053047	27.31832708221800	7.66300659688921
H	-14.12163865272571	29.40806571848010	9.85817622558344
H	-13.49614390503566	27.95493369978520	9.51221330477884
H	-8.75321635709975	24.15297474061952	9.79819373280962
H	-9.83121544827006	24.22512065882341	10.88497116139975
H	-12.14378437048203	25.21110526166605	8.79096389246554
H	-10.61246342212733	24.66756914446882	9.05245010779601

13^{+}

Rh	6.03035793768898	8.27991142291372	15.19111260425648
N	6.18545841686736	8.05600060474429	13.15285069297541
N	7.94379744456723	7.58466726903197	15.41314903876230
N	5.75661592337957	8.16244284115260	17.22825037577246
N	4.09741234417091	8.91895642830634	14.97397122062004
C	5.18086322278931	8.28506959304604	12.23051531112545
C	5.63092865219514	7.84087877898089	10.92687768800606
H	5.02609906181230	7.85773500820203	10.01896966562097
C	6.92300310262556	7.39438653665958	11.07302950479828

H	7.57739904744812	6.97336477446674	10.30833906507515
C	7.27843331582984	7.55967077943674	12.46785094449433
C	8.54393131964552	7.26642419992533	13.01736694609285
C	8.84422561052556	7.32895393518457	14.39334335625912
C	10.15134498311794	7.07192052961834	14.96572695225289
H	11.05722421802280	6.87369877876020	14.39084787073841
C	10.01560456412544	7.13509530159666	16.33142649136818
H	10.78860025830237	6.99727392685358	17.08905345202504
C	8.62315596752678	7.42812271031762	16.60997786921152
C	8.05500161073135	7.47179027647923	17.89868954108567
C	6.70072540278199	7.76426651934100	18.15810276650852
C	6.07544238964913	7.69676482335726	19.46281051965213
H	6.57067752078046	7.36077665604571	20.37506666102648
C	4.77179752054890	8.10834893895886	19.31127142714770
H	3.99645986915259	8.17564955172921	20.07597809318913
C	4.58689062134541	8.44058754952502	17.91380765867794
C	3.41218094008337	8.99486750475543	17.36612014995168
C	3.22445422477386	9.25545651530903	15.99406317250324
C	2.03641520620814	9.86001948315168	15.42434737232334
H	1.19700116482918	10.25228929057727	16.00047217757362
C	2.18644911395308	9.83963016695255	14.05873296408486
H	1.49288561899068	10.20950107659533	13.30219535021531
C	3.46481541249493	9.21596364038260	13.77871997914832
C	3.93024701997185	8.88110703680168	12.49101128343237
C	9.62257000874166	6.83529953000851	12.08210376032478
C	10.08133193416954	7.70380823571342	11.06590137419233
H	9.65024519869411	8.70869578364347	10.98184049708422
C	11.08955769945688	7.29721534681775	10.18269697721439
H	11.44938686242105	7.97538560269846	9.40199285513852
C	11.65220625626206	6.00884061402225	10.29802671417644
C	11.19659054098811	5.13726933155017	11.30848235523982
H	11.64128429746330	4.13776935868554	11.37793148931274
C	10.19384942164825	5.54673146715976	12.19413766995193
H	9.83370139884291	4.86316004057259	12.97206966646812

C	8.92504055552138	7.13551889805932	19.06286487114219
C	9.18114374390017	8.09099310411187	20.07183034430211
H	8.75196830068488	9.09627799174801	19.98282112874448
C	9.98781666228371	7.76758341849674	21.17058194205193
H	10.19205500708559	8.51213178264972	21.94699985901376
C	10.54721766495992	6.47736815964794	21.28112061524489
C	10.29242195855053	5.51927495009175	20.27844820160174
H	10.72969133914818	4.51977426122907	20.38466766361425
C	9.49145593094037	5.84526786886408	19.17865643057473
H	9.28399195148647	5.09502797058412	18.40642899802219
C	2.28392872779081	9.29331106576189	18.29501569575956
C	2.42419175710801	10.26209606582606	19.31383953980039
H	3.36933077635273	10.81010135112050	19.40937000059473
C	1.36248171299782	10.53750979997892	20.18527916716886
H	1.46965073619663	11.29555272957967	20.96810356511410
C	0.14244254171907	9.84171366255181	20.05459710886747
C	-0.00146778497566	8.87107982050164	19.04198420167060
H	-0.95480946813475	8.33633054932509	18.95984439415638
C	1.05764943684154	8.60101129962469	18.16855355877863
H	0.94714405242054	7.83950061870096	17.38721786937861
C	3.03291962430574	9.14968031456372	11.33017755498122
C	3.42537668184236	10.04880900168306	10.31235472476667
H	4.39138349196870	10.56211338084705	10.38925617387743
C	2.58151975197430	10.29903521504181	9.22458200715993
H	2.86934593117017	11.00128680152502	8.43369013852852
C	1.33261629127237	9.65142570153052	9.12937662914775
C	0.93665261704423	8.74893370539034	10.13872586200536
H	-0.02995768968115	8.24079363309157	10.05925338958670
C	1.77987835858571	8.50322195666958	11.22971324420749
H	1.47800035779311	7.79288764266744	12.00874468716079
C	0.47367749120482	9.95901266038780	7.94276954387971
O	0.78820363339156	10.72946357248058	7.04799259896088
O	-0.69827361182620	9.28625955323536	7.97422434115261
C	11.41156742210610	6.07033656276561	22.43354362422554

O	11.91343343725922	4.96320590173567	22.55814723318632
O	11.57419493615520	7.07079363299108	23.32767219953091
C	12.72827182341455	5.51582062298938	9.38175082694409
O	13.24047593874243	4.40891742497856	9.45640959151669
O	13.07156232245630	6.43891560770280	8.45605915948131
C	-1.02692431438608	10.09223178336168	20.95482893513103
O	-2.09840894287298	9.51133090241041	20.86695558733274
O	-0.76268428028690	11.03992257551523	21.88147150144943
C	-1.83886157776035	11.34698799874925	22.78856377622803
H	-1.45361916021680	12.12612872030022	23.46150900464416
H	-2.71915293017457	11.71547966007874	22.23422218521378
H	-2.13017458565144	10.45017251707269	23.36207597444203
C	-1.58910945206808	9.52693505596303	6.86804898445078
H	-2.47831320864103	8.90879888821028	7.05662533808509
H	-1.86418653895687	10.59457787389279	6.81736992930946
H	-1.11195575863077	9.23744170714561	5.91590365411408
C	14.10429014494160	6.04609342041863	7.53185531465772
H	14.25254682076151	6.90491178253139	6.86193515697387
H	15.03805464403194	5.81105259725817	8.07123064499422
H	13.79261135509427	5.15683915867846	6.95721553690091
C	12.39272487532232	6.76080480648694	24.47203231065165
H	13.41211248749441	6.48085045332413	24.15503408299470
H	12.41642298674145	7.67451657200613	25.08267566879360
H	11.95651871924720	5.92413575741318	25.04450175433765
H	5.50319952134683	6.66467963548757	15.01915888966754
Si	4.97492963604959	5.26835871100926	15.49826232721700
C	5.15986534689378	4.36330586851589	13.86828043330321
H	4.57370799424358	3.42493850401930	13.95945734821226
H	4.65075982127309	4.96204739109212	13.08859466892110
C	6.12584714327988	4.69353343732273	16.84035663974750
H	5.96321137538403	3.61298551662279	17.01220732960813
H	7.17910119455009	4.84030898841410	16.54726866802873
H	5.94494611434968	5.22670374042683	17.78802819333624
C	3.21557758914722	5.58571533677250	16.03827688104997

H	2.87399209791353	4.66316513982166	16.55160452132214
H	3.24075473811465	6.36803235058572	16.81962878074121
C	6.61482259197514	4.06285170544766	13.46959528205162
H	6.66477082401890	3.55155342162800	12.49194416773184
H	7.21055309106510	4.98836468038112	13.38485788849127
H	7.11442473318712	3.41327766394632	14.20990776294338
C	2.25176702934676	5.96549193011653	14.90024897738710
H	2.12690646536741	5.13706156410518	14.18088564346681
H	1.25018011409436	6.21758142312667	15.29173674778606
H	2.61500020691372	6.84130050681118	14.33617159762448
C	6.64922152141020	10.28526073091761	15.91577326737820
H	5.76680520945045	10.73364383371670	16.38719642693154
H	7.51721465223312	10.11962565959411	16.56472955298466
C	6.78010558344424	10.25407523488260	14.52876594832138
H	7.75442496669939	10.07133045804288	14.06043069584679
H	6.00237626657737	10.67757761561290	13.88214640821216

[**6**-C_6H_6]$^+$

Rh	5.70977430134526	8.09740644026031	15.22080913109576
N	5.85606278622791	7.91413679318681	13.18355478949712
N	7.69193475331125	7.60562070309629	15.40273677974877
N	5.63768460221141	8.53115382296023	17.22944859387814
N	3.75067541160936	8.66867786382239	15.02230701021695
C	4.82827794522801	8.06894943715504	12.26336381846573
C	5.31951097911437	7.75648307774931	10.93912362710816
H	4.71712459952340	7.77301967108781	10.02983745262340
C	6.65126554179438	7.44009958891395	11.06410548234484
H	7.34782435078654	7.14796282371618	10.27690602683085
C	6.98698549640942	7.54929775173164	12.46669989387348
C	8.26614273147128	7.30467230853908	12.99786159531988
C	8.57734400544624	7.35259524104797	14.36899780500546
C	9.90862948575316	7.16617968746242	14.90967427684890
H	10.80476041620247	6.98600568099693	14.31435142438768
C	9.81163543654308	7.28633834966341	16.27409460044121

H	10.61261579078361	7.22596980765119	17.01222080655426
C	8.42095102681200	7.55849343960309	16.57931127830412
C	7.92934504422984	7.79520178573200	17.87739235965092
C	6.62324035121897	8.23493530410889	18.16180249696585
C	6.10077871624974	8.45348393619427	19.49311651006343
H	6.65647299307500	8.28612433548163	20.41688030674485
C	4.80333582980865	8.88482930479930	19.35592256172292
H	4.09254466772701	9.13340770291751	20.14533237060543
C	4.51011922204688	8.91948291868627	17.93968577214703
C	3.24951740755702	9.23500376610370	17.39772322863994
C	2.92014366620226	9.12265090312860	16.03356254607241
C	1.64703202181107	9.52391020253030	15.46778421822636
H	0.81800890133076	9.94950747034736	16.03498953934556
C	1.71769153011708	9.29830316615332	14.11549675845180
H	0.95792682754314	9.50505977011014	13.36032975040605
C	3.03695063732189	8.76389540109540	13.84063317592078
C	3.51027469589976	8.46351936376150	12.55026761204903
C	9.36433121150542	6.95898710796285	12.04902157672340
C	9.81994673543778	7.90402555827937	11.10331875326213
H	9.36386405401844	8.90025124096248	11.07607211075264
C	10.85672915423305	7.58256986360558	10.21780269187233
H	11.21468880294396	8.31990031993907	9.49178345374356
C	11.45098940430283	6.30406902536631	10.26089039915477
C	10.99750840550950	5.35613752360637	11.20107093904342
H	11.46859308181026	4.36648308996010	11.21624076087978
C	9.96632432382731	5.68097695293757	12.08935901092283
H	9.61301548806083	4.94086003328594	12.81713374381877
C	8.86506967233232	7.58166563819274	19.01933998891799
C	9.25572506847448	8.65983325853089	19.84462770873128
H	8.87118369256547	9.66538553618189	19.63557899601113
C	10.14341776733020	8.45526354334070	20.90875077960009
H	10.45340993455515	9.29344932426777	21.54137820456083
C	10.65072459262911	7.16474655020017	21.16765753522350
C	10.26251365975713	6.08536915121075	20.34757998760489

H	10.66399726084921	5.08930749941100	20.56705946543065
C	9.38067322273412	6.29178924290401	19.28100655467964
H	9.07473372901856	5.45093229326284	18.64685028692930
C	2.18540513633069	9.68991399551918	18.33915678639730
C	2.32975217616365	10.90261697964601	19.04958068183235
H	3.22670402882128	11.51389919572831	18.89306517226534
C	1.33119794725485	11.33256504146184	19.93285418222516
H	1.43934518997417	12.27710662719609	20.47590546198546
C	0.17292096440363	10.55039756251240	20.12391052353912
C	0.02681681216486	9.33706122107523	19.42044550125376
H	-0.87732890999822	8.74024734214961	19.58727728932612
C	1.02233107398614	8.91092714910597	18.53424581033865
H	0.91179458344939	7.96298401896483	17.99428481384021
C	2.54649269606757	8.57182090471803	11.41622814898697
C	2.72826542255552	9.53751561005781	10.40168417072841
H	3.58278343624079	10.22204108543701	10.45537061833611
C	1.81775854741333	9.63651447713890	9.34161989521051
H	1.95551031944260	10.39174937030763	8.56084419847674
C	0.71109613223134	8.76433174299768	9.27736265658056
C	0.52583271507642	7.79831305004378	10.28742166295067
H	-0.33784282147351	7.12675981826016	10.21868568892267
C	1.43263675021982	7.70453607312188	11.34905290834678
H	1.28938307751952	6.94873895400955	12.13044073805802
C	-0.29334504712967	8.81873405173811	8.16877055744445
O	-1.26390974086458	8.08119277783964	8.08440345999026
O	-0.00380458281147	9.78154895940909	7.26551337332313
C	11.60147219177651	6.88466224694238	22.28954539649816
O	12.06397679900011	5.78150851035870	22.53911038967783
O	11.89304343780539	7.99707026319723	22.99960784334894
C	12.56112232093223	5.90117781866529	9.34111782421097
O	13.10027876324096	4.80472985255548	9.35425499601283
O	12.90227426666525	6.89364093592119	8.48952022608951
C	-0.92765973315513	10.95651351923767	21.05377708397150
O	-1.94695024092630	10.30784271874071	21.23644078486020

O	-0.66538652404073	12.12914054475381	21.67264129462066
C	-1.67754459648141	12.59625692996139	22.58515480694161
H	-1.84465870705401	11.85900545363707	23.38915585771334
H	-1.29706535573380	13.54029179760393	23.00031358101225
H	-2.63020907551324	12.76407194207085	22.05370732421527
C	-0.92348077972551	9.90356070438508	6.16333753361047
H	-1.93812959803442	10.13681305966861	6.52953892929142
H	-0.54074265384377	10.72419059632343	5.54012045593529
H	-0.96188808104174	8.96433349608923	5.58502383621229
C	13.96889071676440	6.59144169946662	7.56950540582048
H	13.69694811349306	5.73509742579920	6.92861160072294
H	14.10987522586415	7.49718112695922	6.96282206521364
H	14.89473391532759	6.34523006140007	8.11745785218553
C	12.80738657265726	7.81799920099938	24.09828118579706
H	12.39871992940239	7.09935701039696	24.82943072050569
H	13.77947297158374	7.44168559575104	23.73564557995831
H	12.92542398093295	8.80977157187325	24.55738256519225
H	5.22987512968146	6.87613411255981	16.04751288792492
Si	5.12533352815360	5.54379740752667	14.98265438866041
C	4.74688961071282	4.87432881183605	16.72235724696758
H	4.67586951493182	3.77907596367019	16.54403289959567
H	5.63258283964252	5.02169148077693	17.36841302176713
C	3.60196274506022	5.44957208811938	13.92158699562584
H	3.80241207129991	5.69632319600649	12.86746822741820
H	3.25363684908246	4.39992279245886	13.97132084968246
H	2.79359486071052	6.09792701456974	14.29482955617160
C	6.58908621732201	4.62963693625668	14.26842275364294
H	6.10782297123214	3.74239618831616	13.80337071325286
H	7.01891472309136	5.19662164895127	13.42583133670088
C	3.47297024958396	5.40377297138164	17.39443636909751
H	3.54382943750444	6.48485580301594	17.60634981445001
H	2.58100858793105	5.24793747738748	16.76264671868879
H	3.29120659081393	4.89529082165706	18.35778530891288
C	7.67380810139076	4.18385597125564	15.26135901532038

H	8.48342631470279	3.64209501546963	14.74057783105329
H	8.13318285098641	5.03895066778907	15.78362708070286
H	7.26793614292046	3.50405644901678	16.03060010438029
C	5.78353819204518	10.92724970119901	14.91896515878781
C	7.15084234237140	10.57130407725596	14.97683635471796
C	7.91400569456463	10.51245800453651	13.79221421303421
C	7.30507822524186	10.78598454180016	12.55985018361044
C	5.94123920745176	11.13830318772327	12.50197935511000
C	5.18237531562420	11.22185464338971	13.67688089759653
H	5.20776390980757	11.02866422068390	15.84585416224951
H	7.62783898350850	10.39465449395456	15.94753109916572
H	8.97694993174108	10.25049009728377	13.83971574831151
H	7.89128260652019	10.73440596687999	11.63467936308257
H	5.47928201714693	11.36106506082980	11.53291980073817
H	4.12530027567576	11.50822345203574	13.63657527616890

[**6**-silane]$^+$

Rh	5.99641498054282	8.16235963426609	15.14256927792628
N	6.06313824033039	7.93932135825280	13.10758275778552
N	7.95591707704271	7.58479276039620	15.27043364326696
N	5.90508242307082	8.33431703259484	17.18613277469955
N	4.05226557021652	8.81238383768507	15.00867401427426
C	5.03855792256883	8.21346707318919	12.21864175978929
C	5.43353954580211	7.78787237863004	10.89026940388818
H	4.80304246565287	7.84835566232679	10.00190107257241
C	6.71224212731748	7.29445923814122	10.98650745505449
H	7.33067634608176	6.87550398000142	10.19131950254143
C	7.11695486180330	7.42506384133748	12.37254038345168
C	8.40587667118910	7.13789981951316	12.86223166339272
C	8.78782111658324	7.26624961814475	14.21202683134841
C	10.12796175816810	7.03244981902129	14.71391479382525
H	10.99580105208292	6.80353061020852	14.09351459990905
C	10.07715753898080	7.16359264670273	16.08082048392851
H	10.89572470079255	7.06492098542185	16.79528459493768

C	8.70610682258543	7.47920140020000	16.42848620227600
C	8.22526428139376	7.61648027063478	17.74464960733340
C	6.90246132742033	7.97124773052569	18.07511951308175
C	6.37598097207022	8.00588476319625	19.42534765177786
H	6.93764775961007	7.73951866722169	20.32183615441804
C	5.06577846566866	8.41065299511725	19.33829244626774
H	4.34820555756752	8.53612851918894	20.15028096159579
C	4.77887494592908	8.63071534449420	17.93532011316325
C	3.54124299802598	9.07805458130186	17.43316740881612
C	3.24930971382410	9.21726706939717	16.06115079088267
C	2.05021370190071	9.83997919187766	15.53606804156732
H	1.25539281354703	10.27620889418382	16.14283698314211
C	2.13716406544582	9.79384869214880	14.16545598074893
H	1.42575833958338	10.18232218915653	13.43537120535596
C	3.38247909029011	9.12904116151408	13.83855748958272
C	3.80847845571866	8.82604716909974	12.53024133440365
C	9.43755829488477	6.67618615326560	11.88780267564359
C	9.87814620388872	7.52804570296678	10.85002036065323
H	9.46332702008540	8.54030198472660	10.77303251995851
C	10.84712683691167	7.09465581871742	9.93587428103042
H	11.19225198077175	7.75904591348196	9.13685887803152
C	11.38904056306031	5.79668064928872	10.04330975079199
C	10.95264664736210	4.94266068223467	11.07708867202500
H	11.38155820168230	3.93590958442979	11.14088460276232
C	9.98823176878976	5.37860058384388	11.99240080185856
H	9.64369888763285	4.71028310116509	12.79065277316798
C	9.17339203923536	7.35495539763921	18.86598210268372
C	9.52303079614213	8.38921048174556	19.76362925668558
H	9.10281134889489	9.39198629536028	19.61947428481264
C	10.40687589089938	8.14068068512622	20.81969642494391
H	10.69265331782199	8.93455367577484	21.51949294961742
C	10.95222421220521	6.85306138262885	21.00227326830160
C	10.60624678613507	5.81599520078119	20.11064844908215
H	11.02562510160580	4.81541632662342	20.25846431848068

C	9.72635137076572	6.06772656791614	19.05026485196968
H	9.44675653754579	5.26032507964409	18.36274605325723
C	2.47322055265585	9.43851180134627	18.40757759543604
C	2.67284686782385	10.47207240588210	19.35079156468190
H	3.62305142284262	11.01871346556464	19.35507799393141
C	1.66352926794701	10.81292269781119	20.25991879766420
H	1.81800880898020	11.62106648572160	20.98232849649682
C	0.43493604247681	10.11966302922926	20.24428329400895
C	0.23057200267712	9.08587972977300	19.30710388976653
H	-0.72854417858382	8.55524986922001	19.31329584932684
C	1.23782712536619	8.75033766811518	18.39610774602762
H	1.07937419208353	7.94153268702025	17.67273682096875
C	2.89388763356123	9.15823781922240	11.40053753968714
C	3.29123981415121	10.08435418846645	10.40906884812253
H	4.27363297873929	10.56567899916469	10.48472516577809
C	2.43343978889929	10.39971648176166	9.34972131583049
H	2.72524299381936	11.12311042782771	8.57958620178820
C	1.16458852364474	9.79145535137725	9.25677666479992
C	0.76334531624293	8.86243123070429	10.23976168442318
H	-0.21915171096096	8.38539371991804	10.16210957919009
C	1.62114599277942	8.55172201586926	11.30262067371066
H	1.31488429016372	7.82306491931345	12.06262400626749
C	0.29147915229533	10.16879737018897	8.10147435755355
O	0.60866499844130	10.96654535451040	7.23168009105329
O	-0.89689649969061	9.52486648330529	8.12861498853367
C	11.88417410969029	6.64434566407055	22.15456750829498
O	12.20469516971439	7.51764454520594	22.94707702577016
O	12.33332933270410	5.37102786895012	22.22220426605347
C	12.42171870512277	5.27474470798444	9.09425372795264
O	12.91572520055337	4.15895843878967	9.16097241866304
O	12.74850784086565	6.18182299852619	8.14649361823228
C	-0.68138133819845	10.43883182829904	21.18844162508244
O	-1.76017579513656	9.86469419643294	21.19673422237745
O	-0.36111533290385	11.44169875270953	22.03645710591492

C	-1.38513330627335	11.81752833665888	22.97724543206548
H	-1.64946960960504	10.96356986242536	23.62454374076312
H	-0.96018666297980	12.63654314177484	23.57472024568992
H	-2.29242736078803	12.15555782868966	22.44756514923399
C	-1.80094077925616	9.83306568725525	7.05039892362742
H	-2.04972353540504	10.90835338280741	7.04866909187345
H	-1.34837442229693	9.57179525957336	6.07828557167159
H	-2.70207509948227	9.22974810703491	7.23017677789800
C	13.73508797133823	5.75688883571509	7.18687015810891
H	13.38203707487352	4.86780968234001	6.63645109937365
H	13.87399485592723	6.60460105573333	6.50096393913190
H	14.68479684652330	5.50824160341974	7.69121616673214
C	13.23458265789194	5.08053122497393	23.30752768799464
H	14.14351808613046	5.70222727977780	23.23369175705341
H	12.74802124171664	5.27678868037766	24.27850934072050
H	13.48830970480410	4.01518998519841	23.21228131532834
H	5.83945617220298	6.53278425669209	15.29009692698605
Si	4.72089816026428	5.42120028193851	15.41190932241232
C	5.98675857548299	4.10075488289289	14.98583647454602
H	5.44929073276868	3.13035352378197	15.02665289471151
H	6.27267361897697	4.24931613181878	13.92611627090407
C	4.18574852197886	5.43454319661812	17.19144905244726
H	5.04985410014929	5.54420270317078	17.86848899655197
H	3.47330573092690	6.24923632562362	17.39917072348565
H	3.68786351294003	4.47314677430476	17.41942334982958
C	3.35402412710969	5.56806950613700	14.14477873767894
H	3.70474217444251	6.20935910276099	13.31533297423397
H	3.26091459621365	4.55297006176600	13.70694790588646
C	7.22802277939438	4.08429883905988	15.89399746980878
H	7.92574493735953	3.27859348636142	15.60434847439687
H	7.77858642394088	5.03887196177481	15.83462991946579
H	6.95817659426573	3.92308186043297	16.95293955426026
C	1.99375060200453	6.04273488633559	14.68273831285930
H	2.05252305060825	7.05356283774892	15.11865030685773

H	1.23852914452067	6.07242286168784	13.87722428137703
H	1.60935191581240	5.36606174070158	15.46652207514734
H	6.35092203866219	9.72733143981850	14.74506508330373
Si	6.97412741506336	11.06789847864970	15.29821210704456
C	5.89254027236800	11.76153302221578	16.65710745170346
H	5.71384353949813	12.82256280421315	16.38838745900465
H	4.90188572320038	11.27385461891296	16.59471965584345
C	8.73835821937008	10.73211543414155	15.76900004285429
H	9.26465870684555	10.15886126630719	14.98762543945291
H	9.26011679437767	11.69870721669010	15.90148961332599
H	8.80246389289835	10.17396735230811	16.71704692449222
C	6.74351988880780	11.89909595675248	13.63126384486615
H	5.65486639902195	11.93385670873479	13.42997879716010
H	7.06810425716424	12.95341839853478	13.75425258390082
C	6.47674706077187	11.65782234796889	18.07672156543030
H	6.70048178813893	10.61439332052865	18.35471255246837
H	7.41488120742919	12.23282374382964	18.17147941790707
H	5.76962556461551	12.05522079952491	18.82644068986516
C	7.49611114674226	11.21924903009745	12.47457049883105
H	7.16842767401276	10.17344221382005	12.34175954913411
H	7.32222832027703	11.74619457057128	11.51954056372580
H	8.58641753350884	11.20417735938556	12.65014076461227

6^{+}-TS1

Rh	6.08454534906716	8.20111308770202	15.18563341189861
N	6.34869056825327	8.23729340475026	13.15937313991322
N	8.03192679226004	7.65707234937290	15.42999252418613
N	5.86644135539655	8.31085163125826	17.21401741702733
N	4.19969095127280	8.93629802510749	14.94858797434346
C	5.34439102808964	8.38588458616077	12.21078506819361
C	5.85917005665031	8.04892268296486	10.89956159318168
H	5.27819068614426	8.06244631401013	9.97623174223519
C	7.18316333479173	7.72077445704089	11.05832571210259
H	7.89308238548998	7.40823292791513	10.29111436622887
C	7.48400043392884	7.83232394884794	12.47048149636740

C	8.72896477058749	7.50966665082726	13.04262302134477
C	8.97140557334699	7.45170479397797	14.42702501552156
C	10.25376123903018	7.13247603353848	15.02140171264825
H	11.17889116566315	6.97618137479101	14.46460748904139
C	10.07049016312978	7.09493644057604	16.38125933828092
H	10.81663733137210	6.90472100360261	17.15425406879470
C	8.67701084033701	7.40291516142380	16.63210813345109
C	8.09182978115229	7.44149038588100	17.91231072770425
C	6.77035995898570	7.85494727495603	18.16632987167696
C	6.15382831112967	7.88394615996653	19.47649931542442
H	6.63182076331675	7.55654206570962	20.40098750898770
C	4.88817669836698	8.39273494975906	19.31592948125430
H	4.13036988347334	8.56035405298783	20.08285567885917
C	4.71382873773589	8.66541151832703	17.90438194712189
C	3.53672189333151	9.18768884243408	17.33535431760253
C	3.32251041264589	9.32858051315881	15.95095919327999
C	2.11459699327047	9.86453560569381	15.35760609248262
H	1.28216441757406	10.29418443771281	15.91675370305943
C	2.24250678506846	9.73782844852426	13.99626352932683
H	1.53576867191894	10.04455306364249	13.22366519006573
C	3.53682947260488	9.13865301665777	13.74452157011126
C	4.02963660057160	8.81918902660111	12.46580586721785
C	9.84222881750405	7.14579488618164	12.11838775210832
C	10.36090548253033	8.09611951884023	11.21077283493810
H	9.95248485230141	9.11387300546241	11.20543672123764
C	11.39799710888735	7.75049331750573	10.33461645590858
H	11.80496270390223	8.49089017898967	9.63814392628145
C	11.92797652763923	6.44325656581046	10.34747313242004
C	11.41116805690687	5.49041288580519	11.24924461245189
H	11.83219667141075	4.47834597237818	11.24028468945923
C	10.38010920856840	5.83857918833107	12.12855088661591
H	9.97351818547639	5.09383236930969	12.82320403053447
C	8.92745693895880	7.00732755704932	19.06971547661712
C	9.27901289838405	7.91827533370255	20.09063639763524

H	8.94376670880328	8.96012966285665	20.02174600017144
C	10.06498752936795	7.50406604877366	21.17382559588304
H	10.34450439734016	8.21404493363149	21.95909001622646
C	10.50731114971854	6.16709076751269	21.25600810366882
C	10.15862814406067	5.25424472291000	20.23946929035499
H	10.50963038461523	4.21904285950109	20.32153720343517
C	9.37965615536495	5.67079443794604	19.15440832894995
H	9.10701361438024	4.95922975632986	18.36573716625380
C	2.43485682974835	9.58341229226843	18.26070506465953
C	2.61492444304907	10.65360381779225	19.16525164073570
H	3.56700501341423	11.19774798974194	19.17185963793697
C	1.58396314613812	11.03107214459127	20.03535804346354
H	1.72240311509642	11.86728925995681	20.72845066722268
C	0.35615475526128	10.33688271101342	20.01934284511660
C	0.17370930518926	9.26460648618840	19.12201131680664
H	-0.78536302202841	8.73393168924009	19.12815033946866
C	1.20196637232177	8.89294207093752	18.24899138558309
H	1.06035619366196	8.05427511454300	17.55734814532078
C	3.09090037900554	8.94830789837307	11.31365439315817
C	3.33183155264749	9.88013686225972	10.27914172819491
H	4.21558730323080	10.52736595519488	10.33004454435927
C	2.43884398202164	9.99207469023102	9.20739745450038
H	2.60873713287506	10.71689978093103	8.40289472068341
C	1.29263670730830	9.17299454492323	9.14680663912030
C	1.04661605367435	8.24068702950597	10.17638693253665
H	0.15754715842865	7.60356722859298	10.12594051518114
C	1.93779567380014	8.13363827104359	11.25152921181635
H	1.75014035884220	7.40606690457164	12.05055384406720
C	0.37647701262316	9.33655621540199	7.97443789341389
O	0.56247071940759	10.12697760533317	7.06151211329772
O	-0.68816596404526	8.50559946687302	8.03946203422028
C	11.34636044714889	5.66530408079821	22.38939557173830
O	11.74432286685290	4.51441139890588	22.49303724966301
O	11.61654654178237	6.63471029274821	23.29144683358373

C	13.03200984979589	6.01315479841939	9.43308021618316
O	13.51477536984203	4.89046697006962	9.41909246176625
O	13.43799951089502	7.01256038650199	8.61884144576091
C	-0.78187465244550	10.69340458885584	20.92383446265479
O	-1.85877876275354	10.11560562142733	20.93391986194245
O	-0.48304954178762	11.73382141343754	21.73282934286659
C	-1.52801649178841	12.14893438320889	22.63314455132727
H	-1.11571821089436	12.99200961045940	23.20547574078724
H	-2.42214521536370	12.46548753938042	22.06889522190150
H	-1.80853163819035	11.32288354504710	23.30916783073786
C	-1.62347692098611	8.59999491405546	6.94793804041882
H	-2.41710628452664	7.87143525273765	7.16647332304152
H	-2.04132751011557	9.61936142327227	6.88276750973090
H	-1.12879897750789	8.35746361108006	5.99158700918602
C	14.50326739607880	6.68447973070942	7.70659031436253
H	14.70162345165330	7.60045783648474	7.13214908253525
H	15.40545394846025	6.37271950024011	8.26061457091879
H	14.19869155678972	5.86443952440418	7.03361164661888
C	12.42440562497082	6.23760833426703	24.41598716003794
H	13.41147878825171	5.87919975639107	24.07632568731388
H	12.53512666664796	7.13565178420902	25.04015019605426
H	11.92932538125780	5.43065480540455	24.98317791868731
H	5.71820669400969	6.73224589457057	14.91624525351038
Si	4.85539985806249	5.24624673183502	15.43441358111570
C	4.87562889136075	5.02432912586160	13.56648416527081
H	4.08276832170337	4.29513298322004	13.31324657389221
H	4.54770741425787	5.98322641959660	13.12223786328434
C	6.25152095936118	4.69404133295836	16.52711399932408
H	5.91809358215798	3.90015975644792	17.21551049842178
H	7.09896179063519	4.32186026946134	15.92761857278777
H	6.60230842331417	5.54356779475819	17.13681358079366
C	3.27371296601151	5.82050655034332	16.22974602159837
H	2.97301388196834	5.06885512530180	16.98277702298728
H	3.52957463869123	6.72309634374598	16.81674031084959

C	6.24255766408815	4.60004236379635	13.01079049466398
H	6.22908223955773	4.58326562056776	11.90743578886071
H	7.03855406280627	5.30057696694070	13.31653984884523
H	6.53230007255961	3.59395500910139	13.36157776440868
C	2.13673711901495	6.11679471732465	15.23847994682147
H	1.83056054761069	5.21404962835927	14.68096283533517
H	1.24526277106450	6.49785984177681	15.76587110448019
H	2.43238744405742	6.88172364361990	14.50059833103285
C	3.57334489460012	2.65225286251674	15.79063925959773
H	3.55063370790704	2.72196838268848	16.88577885659015
H	2.65235873203370	2.92373566359858	15.25848712710226
C	4.65275672919300	2.17322261770347	15.13796014903464
H	5.55693087067373	1.85612369230104	15.67264973770094
H	4.66026458547993	2.05423535487072	14.04736108091602

6^+-IM

Rh	6.02912272801703	8.09479922532259	15.12827070263608
N	6.33807393288470	8.29046182703255	13.09985613346005
N	7.99274631433783	7.58147758527139	15.36921103616676
N	5.84206233564364	8.26631231754130	17.15700924177681
N	4.15602225077660	8.87545345009738	14.90266330131343
C	5.34008208383562	8.48786755410723	12.14882091851618
C	5.87194435672860	8.21848790885926	10.83319513896046
H	5.30496922290652	8.29110253497155	9.90398174970404
C	7.18850954249627	7.85630014079664	10.99140039173303
H	7.90239909679528	7.57255245392326	10.21681300661248
C	7.47503423052580	7.88393386500677	12.40710486795338
C	8.70022519547205	7.49399660138289	12.97855533414835
C	8.92901350401050	7.38221723513811	14.36153036695386
C	10.21101000681239	7.05237655090026	14.94876823883255
H	11.13182839328119	6.89187625301886	14.38618243136077
C	10.03771102593763	7.02847031390305	16.31000851210399
H	10.78920958865591	6.84626207247801	17.07966882082986
C	8.64896209235977	7.34771594233557	16.56884296018596

C	8.08646439475316	7.43322142158553	17.85525481379430
C	6.77147800916400	7.85626053503263	18.11237343678173
C	6.17684827505977	7.92987686242679	19.42599729972905
H	6.67578151736899	7.64863425689411	20.35447346206765
C	4.89821290966358	8.41138040416785	19.26881458111885
H	4.15345701542695	8.59781357095462	20.04395653425130
C	4.69307941707745	8.63106188264963	17.85665756243063
C	3.50264016910364	9.12396593491570	17.29348779842691
C	3.28045471943933	9.25484321641693	15.91080686308803
C	2.07661749938460	9.80728671073529	15.32636914133506
H	1.24513720145249	10.22826568949773	15.89327934426831
C	2.21249108798433	9.72174341891538	13.96295648090551
H	1.51488329184090	10.05965469597749	13.19532685129236
C	3.50714304892783	9.12933888340190	13.70054740060392
C	4.01891858441702	8.89146073863658	12.41215916363331
C	9.80973081879584	7.12131763581176	12.05355280262887
C	10.37867661768663	8.08078481329077	11.18696307587522
H	10.01276355368056	9.11433378446444	11.21182219866138
C	11.41364613511055	7.72235222533387	10.31336186093410
H	11.86090716337544	8.46835046458717	9.64832160398664
C	11.88949353201983	6.39465963082778	10.28839804496485
C	11.32235544692483	5.43365421788269	11.15019730941826
H	11.70235991039470	4.40617371142957	11.11216333293957
C	10.29398626668003	5.79356489580338	12.02791426807169
H	9.84771899246440	5.04372587012027	12.69197836168794
C	8.93649561761552	7.03448353884399	19.01476333141825
C	9.31384624301138	7.97952446370962	19.99451571830342
H	8.99090418343437	9.02239874805456	19.89059649143005
C	10.11005283888078	7.59633774707825	21.08170451228650
H	10.40951532584231	8.33173780821905	21.83557769472991
C	10.53653425027271	6.25775930163248	21.20838137823888
C	10.16232384775209	5.31164477381203	20.23239699071846
H	10.50085946868517	4.27565343955800	20.34921381688910
C	9.37294280173107	5.69625585502638	19.14297905267143

H	9.07878903804481	4.95755741358439	18.38804077277138
C	2.39607045702887	9.50460090534135	18.21896024688222
C	2.55290619871379	10.58223799155731	19.11864492748029
H	3.49229085882773	11.14810197074538	19.12361035958786
C	1.51253202494348	10.94069100541673	19.98553798377779
H	1.63162469230845	11.78261073776778	20.67529172762540
C	0.29994362518404	10.22030743909012	19.96997351909903
C	0.14076220365425	9.14183871366916	19.07573785392637
H	-0.80735997001088	8.59186552440877	19.08148153892405
C	1.17769230061913	8.78847628133018	18.20525631740505
H	1.05445296078296	7.94633137432219	17.51385893445397
C	3.09537362142439	9.08573471576988	11.25726041514394
C	3.34547590901099	10.08162013709470	10.28639094198644
H	4.22565444055233	10.72758808470482	10.38907529697673
C	2.46366884809414	10.25832637159678	9.21421164771872
H	2.63880440647989	11.03377191288128	8.45956063395252
C	1.32176607586955	9.44056848314231	9.08939163970794
C	1.06828055652418	8.44362025182603	10.05453778164891
H	0.18285415815151	7.80763481341015	9.95294350495720
C	1.94702992456798	8.27179590952244	11.13150053801131
H	1.75410980122454	7.49408688353988	11.88036332114524
C	0.41695035676726	9.67624820682958	7.91994924721953
O	0.60773288938531	10.52788082335340	7.06502240547735
O	-0.64127632246474	8.83566357466884	7.91710025757076
C	11.38453262544984	5.78742608558621	22.34917082226427
O	11.77152950249577	4.63671176324893	22.48795037250916
O	11.67487036426128	6.78478122783170	23.21325871699304
C	12.98835683883348	5.95037862120602	9.37363125311579
O	13.42509185760197	4.81003093644779	9.32895721040595
O	13.44501775667138	6.95851603437808	8.59862549031306
C	-0.84691222765176	10.55667365352609	20.87178452831746
O	-1.91265897260221	9.95897072098002	20.87942885115059
O	-0.56857358660012	11.60282035481774	21.68026792326561
C	-1.62260540224769	11.99989905512063	22.57845893523229

H	-1.22547361400037	12.84886643262733	23.15276695553188
H	-2.52017087974665	12.30262596862659	22.01210431000339
H	-1.89129621714540	11.16852641567057	23.25270298179290
C	-1.56582748547417	8.99610717404022	6.82381827826586
H	-2.35135173503766	8.24318245057974	6.97988092223537
H	-1.99732821159205	10.01187490223045	6.82760234248385
H	-1.05690535778086	8.83001464750678	5.85873460423547
C	14.50818378958289	6.61711866638085	7.68829102953986
H	14.75385895557026	7.54354721849054	7.15011919772510
H	15.38786161088582	6.24640068879858	8.24208210490835
H	14.17760359686098	5.83584384310396	6.98231766406956
C	12.49022117869032	6.41782665287935	24.34302514752299
H	13.46904254613519	6.03529218725789	24.00595230543712
H	12.61835898689765	7.33601121638135	24.93359164418417
H	11.99126689401472	5.63773280130856	24.94336637561429
H	5.76245779374200	6.81671643096505	14.29693460330582
Si	5.12323622284133	5.75555923719695	15.56488875458959
C	5.07849820800675	4.86792421573613	13.87751440191883
H	4.46801399395980	3.97567954765721	14.13633683517289
H	4.47904440200394	5.44850826406869	13.15141816626555
C	6.31467631933404	4.88898036633576	16.69769926104216
H	5.99235959655028	3.83066899729583	16.71820063664308
H	7.35172315189770	4.92977855222623	16.32863440671849
H	6.28227930931996	5.28192197894780	17.72607166132581
C	3.38801763238113	5.86248425282667	16.24902361391870
H	3.28522803427806	4.89116753579767	16.77734826608195
H	3.33276874529269	6.63120144590468	17.03815233522963
C	6.43066998164664	4.44797071134293	13.28697960650195
H	6.29167744209742	3.85035907036107	12.36862145578922
H	7.05128971714241	5.31969598255382	13.01666930676631
H	7.01154322559377	3.83454777817284	13.99748351734915
C	2.25912991068051	6.02847996541690	15.22013990820840
H	2.23032458022212	5.18267399182957	14.51142108117418
H	1.27452286839263	6.07349114031872	15.71878185840854

H	2.37060080141269	6.95304438086287	14.62972208458623
C	3.33655018138428	2.20253598412597	16.33608603673644
H	3.35129124954541	2.36278732630194	17.42239704523898
H	2.41775845908111	2.49145597849333	15.80878723037690
C	4.38260194266669	1.66064060966811	15.68879237036593
H	5.29553611602291	1.35659681730488	16.21795660816213
H	4.36267658103537	1.48158363543647	14.60552353794910

6$^+$-TS2

Rh	6.08634527921125	8.24494153480421	15.16499045647517
N	6.28438677089959	8.15434249839947	13.13769132809954
N	8.02777942944003	7.67642282871905	15.37863918019718
N	5.89077582316034	8.34773450004969	17.19271979969566
N	4.20195348893557	8.98093276344505	14.94379006356576
C	5.27398771374645	8.35037521798499	12.20485020421957
C	5.72103458757541	7.90116105008066	10.90185102910944
H	5.11710236658744	7.91509912385969	9.99321311556132
C	7.01578914752110	7.46549063983030	11.04740954250541
H	7.67382308227005	7.05375378424777	10.28068041362221
C	7.37122026089098	7.64328273258850	12.44080903403725
C	8.63633857435526	7.35151130371776	12.98599569139734
C	8.93656413811395	7.41933694506530	14.35923049600611
C	10.24662895868602	7.17375211269998	14.92792925743843
H	11.15594212087596	6.99897571003108	14.35084818674630
C	10.11167286006402	7.22839972019580	16.29319258683641
H	10.88864852286641	7.10772776060948	17.04974057512161
C	8.71841843312264	7.51359161622468	16.57271667056284
C	8.16238178858991	7.58246927416170	17.86433169032911
C	6.82552871003483	7.93265822444008	18.13136725539195
C	6.21078818834877	7.88539487310527	19.44254156004382
H	6.71304181721023	7.57113378154469	20.35877011652446
C	4.90679248895530	8.29224150323707	19.29249930601872
H	4.13898970511749	8.37800034750640	20.06303556503572
C	4.71599773622600	8.60145943156722	17.88979148315827

C	3.52152545320970	9.10117603135091	17.33549938880178
C	3.32349446163734	9.33143642936369	15.96011594448944
C	2.13267210980795	9.92931515352773	15.39037216073880
H	1.30386076589459	10.34336588732264	15.96677676752409
C	2.27742263841751	9.89297464068735	14.02504694367935
H	1.58865721544381	10.26881871513780	13.26697291252428
C	3.55225445043669	9.26241347543449	13.74834323048930
C	4.01350207129601	8.92322567789586	12.46326992981505
C	9.71380130065357	6.91490316239388	12.05168758766238
C	10.16250008334154	7.77367393518802	11.02260441903294
H	9.72532856518531	8.77509152543252	10.93009584719819
C	11.16780291654106	7.36283169523891	10.13807769196176
H	11.51874428998772	8.03450919711226	9.34775332043840
C	11.73888416445837	6.07906543354351	10.26411493150370
C	11.29612603483538	5.21794091564918	11.28922616151345
H	11.74920288538017	4.22297252757542	11.36925431129303
C	10.29661934309825	5.63258609444950	12.17617950620819
H	9.95132089587578	4.95876693407995	12.96899465113764
C	9.03367767589968	7.22069155748355	19.02000728368305
C	9.33352366128683	8.16858905582107	20.02399053306189
H	8.93518892740166	9.18670287122830	19.93828486813653
C	10.14546818866895	7.82173616990360	21.11169885307659
H	10.38398627561364	8.56114084601278	21.88326431418577
C	10.66682730306983	6.51509174005644	21.21688394658701
C	10.36954921347256	5.56457966076624	20.21851103457954
H	10.77999739941849	4.55312041037583	20.31895371178159
C	9.56405204433029	5.91466368577652	19.12957032437625
H	9.32821729091195	5.17278288574420	18.35715603901140
C	2.38236351450294	9.37722256269808	18.25839418050724
C	2.49031721324545	10.35938582650724	19.26832406039167
H	3.41748626719606	10.93789058163562	19.35915050946553
C	1.41971863659776	10.60892726320451	20.13683718538450
H	1.50298057645199	11.37663238306277	20.91314519664869
C	0.22197291000211	9.87410471568728	20.01252071639739

C	0.10813614424420	8.89327748267744	19.00578528333883
H	-0.82923713596298	8.33051090013147	18.92631507673048
C	1.17622238763049	8.64980030662493	18.13570989265266
H	1.08846570295795	7.88287665521412	17.35684646475257
C	3.10531849721230	9.16824234701894	11.30504341515348
C	3.48108073308745	10.06085697647280	10.27509703432512
H	4.44243881409609	10.58415582390128	10.34102600980489
C	2.62753797236474	10.29238240227306	9.19067860498643
H	2.90397202550419	10.98988541165194	8.39156303351464
C	1.38374714990002	9.63272806849440	9.11006017674536
C	1.00311575823201	8.73794463317376	10.13207640601389
H	0.03954559348554	8.22215603756243	10.06567230185577
C	1.85651032735620	8.51158679499533	11.21924194443117
H	1.56262317739154	7.81008167080091	12.00886764168108
C	0.51334166367988	9.91979509194229	7.92757100461742
O	0.81309821405289	10.68184591032608	7.02057418111321
O	-0.65343144248247	9.23705109404043	7.97524055205750
C	11.53483475579268	6.08362229543216	22.35675897695619
O	11.99849180046305	4.95949928306811	22.48112330885055
O	11.75048496740608	7.08295938473285	23.24121827373891
C	12.80993711484619	5.58108182922720	9.34570424455046
O	13.32667797119587	4.47630487002446	9.42617714048887
O	13.14429337756232	6.49636720232041	8.40861814623610
C	-0.95311942097874	10.09161939071904	20.91291770564700
O	-2.00518196689266	9.47453561202983	20.83184133472403
O	-0.71915736567620	11.05333583887024	21.83341098977421
C	-1.80232588235595	11.32930255979177	22.74168877791472
H	-1.43933982738588	12.12036250930147	23.41313415253776
H	-2.69411801551046	11.67125902777799	22.18862153741780
H	-2.06623215961755	10.42496594460288	23.31670471639806
C	-1.55413337160678	9.45964030887418	6.87375020611192
H	-2.43632263198666	8.83501536344266	7.07399029259091
H	-1.83988351971877	10.52414293115077	6.81551055552079
H	-1.08148345972486	9.16660571326451	5.92038308587783

C	14.17161075194308	6.09808268836883	7.48130096348400
H	14.31177819386186	6.95055393872835	6.80154403354420
H	15.11033346055065	5.87221967367142	8.01605645615798
H	13.85949518652960	5.20175841245588	6.91790641392224
C	12.57905579608134	6.75105526627316	24.37162519371349
H	13.58337372539281	6.43815408539728	24.03757849440355
H	12.64291357268510	7.66567850561797	24.97806898160400
H	12.12744647575430	5.93050580537100	24.95542666409752
H	5.64557885062485	6.79476555110340	15.15440399910889
Si	4.76283321524126	4.92024375679678	15.59177069072517
C	5.41890391131749	4.73082154544074	13.84204936965967
H	4.79343733186436	3.98196688833772	13.31846300802484
H	5.23200755461625	5.68420135147437	13.31425911229957
C	5.91996033105793	4.87752402470494	17.05081788209824
H	6.15120606791984	3.86329048949074	17.40969273319808
H	6.87104522010898	5.36281297758777	16.77197011717215
H	5.47772216266523	5.45527746656784	17.88124957348704
C	3.14577298922281	5.82674864067177	15.84996804933483
H	2.53606004562129	5.31947044865409	16.61892711552460
H	3.47192776911320	6.77257275650047	16.32570445716367
C	6.91134036508051	4.36928388986746	13.78163681529706
H	7.26115453265734	4.33340735128947	12.73572372057214
H	7.52428628235603	5.12259208074434	14.30490748106045
H	7.12246115473604	3.38793787238155	14.24331456535848
C	2.34875121815511	6.12032053856592	14.57201784784538
H	1.90159425890458	5.20758464602468	14.14011250664353
H	1.52918713781659	6.82966873241826	14.78207323598190
H	2.98520502240213	6.58280811915330	13.79874161918332
C	3.38133869589417	2.84062405527186	15.72490605685723
H	2.85698945885822	2.97263630515985	16.67950167202050
H	2.81384356883476	3.05866234175671	14.81011186918426
C	4.62340928345720	2.29153568539270	15.67664199709573
H	5.14879849501945	1.99174261667514	16.59126916791934
H	5.12589087871493	2.07902831468453	14.72500146615162

6^{+}-P

Rh	6.11802177486160	8.24262335583608	15.17322647293326
N	6.34653007226380	8.18459983095492	13.14639110319776
N	8.05613428679769	7.66874447681505	15.40684375023744
N	5.90400879984734	8.35243740502209	17.19954672482471
N	4.21687171193038	8.93476228308610	14.93675565764399
C	5.34025611304054	8.34184553744519	12.20104018734094
C	5.84193362690225	7.98236137401480	10.88980667639485
H	5.25504460799311	7.99267445126914	9.97007450694439
C	7.16297088704870	7.64028865829029	11.04393896909101
H	7.86498305768076	7.31255228152676	10.27566210905979
C	7.47499453101052	7.76968481498185	12.45296044333431
C	8.72761026638135	7.46515895068965	13.01722246894177
C	8.98532611991905	7.43952251253349	14.39992082862650
C	10.27285565248170	7.12710169358806	14.98830139094205
H	11.19191019267196	6.95498525121873	14.42601723190451
C	10.10208829342519	7.11569164884000	16.35023394362568
H	10.85432648987047	6.93432086124413	17.11957369247498
C	8.71157794764748	7.43398869360094	16.60785305402806
C	8.13577732773983	7.49132330013123	17.89084906369284
C	6.81485937898731	7.90464984989268	18.14857595029101
C	6.19989481682873	7.92136113510724	19.46072221063290
H	6.68401125156034	7.59815543106908	20.38355708979356
C	4.92489616048655	8.40637248237394	19.30254689016135
H	4.16439127727442	8.55630892907157	20.07050670195059
C	4.74529343949406	8.68137244849680	17.89120245056442
C	3.55680185115512	9.18034536305105	17.32433826067588
C	3.33870040668366	9.32128644095310	15.94093527706976
C	2.12425758920007	9.84757244459313	15.35086743826832
H	1.28868021357973	10.26861565256913	15.91209763828112
C	2.25267923118355	9.72749635628343	13.98910764583382
H	1.54245633607503	10.03085744639455	13.21830893590168
C	3.54990815215298	9.13390450007595	13.73462007645870

C	4.03535846240266	8.80360562445737	12.45592701692798
C	9.83497066308001	7.09512927302468	12.08780866116666
C	10.34004969106586	8.03798144232190	11.16471011242595
H	9.92308019618377	9.05217423368987	11.15045416801811
C	11.37359528628333	7.69084144306242	10.28507354609571
H	11.76926359291261	8.42638058012681	9.57701289196286
C	11.91536291929083	6.38856739737799	10.31002541433500
C	11.41246176580893	5.44256349795747	11.22690205596128
H	11.84251561936679	4.43422679702955	11.22765376955909
C	10.38420101008862	5.79282466421480	12.10886805715574
H	9.98976661665450	5.05364404531273	12.81632774537200
C	8.97547091260499	7.06534779239293	19.04876108474842
C	9.33098555825894	7.98156710641051	20.06364432706697
H	8.99758971760393	9.02356991173993	19.98823326996160
C	10.11613135925601	7.57273414449266	21.14959286548553
H	10.39748482181939	8.28731442799249	21.93004993117709
C	10.55519601394673	6.23517243374704	21.24102641069772
C	10.20348853364478	5.31667790466786	20.23052045214718
H	10.55180961371131	4.28110144119328	20.31942614153567
C	9.42438318729632	5.72824815966377	19.14375193709314
H	9.14838091460196	5.01197495520919	18.36051418758917
C	2.44498699610273	9.54352341899320	18.25147696316870
C	2.59551764134986	10.60747356641799	19.16866100224990
H	3.53295329180282	11.17638569510730	19.18241790105434
C	1.55548340119578	10.94660349441806	20.04393452520580
H	1.67249950482793	11.77745390294529	20.74742534535201
C	0.34621884524807	10.22059889969504	20.02008196841069
C	0.19161259933270	9.15590024938623	19.10852207828617
H	-0.75336536088247	8.60038384473113	19.10764365728164
C	1.22972780471089	8.82249125667338	18.23189898432027
H	1.11028415190618	7.98860292980770	17.53028015758486
C	3.10199383373795	8.95562662649307	11.30120574326415
C	3.36370512884443	9.89252358844473	10.27620307458562
H	4.26075279784869	10.52032621303296	10.33665160619534

C	2.47725128462087	10.03307581944060	9.20237082464106
H	2.66463199528115	10.76260416280963	8.40600938453800
C	1.31465414024890	9.23830772282899	9.12992336139960
C	1.04794050124310	8.29981507242055	10.14883982812299
H	0.14650766859544	7.68111020281727	10.08893951236558
C	1.93421862028915	8.16367848470380	11.22479305684424
H	1.73022207101106	7.43090945544147	12.01486744054954
C	0.40443892529930	9.43376018349461	7.95856709453234
O	0.60759693416631	10.22970899149416	7.05399518226297
O	-0.67859134521741	8.62507424386117	8.01293713270498
C	11.39194672766697	5.73814628514799	22.37752142270174
O	11.78490455861117	4.58615200562539	22.49034432258749
O	11.66699417093882	6.71268072844777	23.27307551240393
C	13.01751466574811	5.95801955951121	9.39443606112244
O	13.50972718870855	4.83916932667907	9.38815599790237
O	13.41202250986038	6.95281766199585	8.56825255552554
C	-0.79922915264649	10.53340323060270	20.93048700568994
O	-1.86078209646851	9.92733454938808	20.93372534238888
O	-0.52715761852307	11.56905380604367	21.75536501991043
C	-1.58144018900254	11.94177032622285	22.66294252128116
H	-1.19004425762938	12.78442438727016	23.25041910931433
H	-2.48360729780340	12.24625052557120	22.10479075664682
H	-1.84129457808353	11.09711524846571	23.32414741733314
C	-1.60991500717564	8.75307211769458	6.92179859983009
H	-2.41927230008826	8.03886492686900	7.13001884503845
H	-2.00625557201663	9.78175403696289	6.86884796278556
H	-1.11947195204582	8.51241145496717	5.96275848196427
C	14.47595675233453	6.62469967335906	7.65498463684472
H	14.66493423225736	7.53686075875711	7.07129433221404
H	15.38296563975388	6.32516324485326	8.20796277834727
H	14.17553199360929	5.79615196733870	6.99054629581971
C	12.47292269964696	6.31919668959013	24.39995391446283
H	13.45852755962323	5.95425911493346	24.06287490640430
H	12.58765620140957	7.22061861871131	25.01854380874162

H	11.97451165070117	5.51794913276189	24.97237633859428
H	5.70898797552057	6.78459483960597	15.17809994631648
Si	4.68091211200072	4.91677503115776	15.50615530513597
C	4.97278533648404	4.97629219800824	13.65432640756548
H	4.21857816421406	4.32559633961280	13.17012943775850
H	4.72591067303855	6.00220464502475	13.32415771068079
C	6.06663209500168	4.66822225361478	16.72193219121931
H	5.76324434871854	3.97141428719125	17.52253359278333
H	6.97598011220559	4.28479222676839	16.23004120125417
H	6.31122207868442	5.63452653057834	17.19542924931430
C	3.16421062446372	5.79243611346342	16.16280545522496
H	2.73342523378791	5.23425717100371	17.01336440741279
H	3.58648842142384	6.71475156147149	16.61076936850112
C	6.40283303118683	4.60861164620770	13.22809574466382
H	6.53085974353080	4.74274342219743	12.14050947086360
H	7.14603186453417	5.25470194472760	13.72560704824245
H	6.65556870387218	3.56076891508692	13.47067896196587
C	2.11075917947340	6.14651590774767	15.10453777007201
H	1.63307388061077	5.25012121449584	14.67087593061665
H	1.31130394059813	6.76896636280529	15.54180017154539
H	2.55384506925490	6.72756911572919	14.27820939164572
C	3.37475802183178	2.82685294302526	15.72362648210631
H	3.12941992913413	2.85926850553440	16.79281343815897
H	2.57331678383656	3.08606417782276	15.01888168072430
C	4.57267997511722	2.34914113303300	15.29181072618301
H	5.34081701358424	2.00914433825875	15.99705743620247
H	4.79393547473290	2.23425827923467	14.22375062861801

13-TS

Rh	5.96404032101512	8.15959542018041	15.26469799097196
N	6.12231950250506	7.94766724901645	13.22598431142427
N	7.89055310250675	7.50816197079867	15.48164946458069
N	5.73894246423289	8.18396430579380	17.30765369278779
N	4.05946110321810	8.87001452574101	15.04468416371981

C	5.12478301741613	8.19903874163899	12.29939495979131
C	5.55448464407705	7.72671955026968	10.99598611990304
H	4.94880075909832	7.75092948851635	10.08858245666814
C	6.83068812081135	7.23865633537118	11.14451861139876
H	7.46658892277062	6.78941399738437	10.38029862915648
C	7.19750676087043	7.41788569801160	12.53909132620714
C	8.46391495605029	7.12211262042144	13.08677041663894
C	8.78370366775462	7.23365957659533	14.46156058841652
C	10.10720345806394	7.04425770392626	15.02044460767275
H	11.01333207299463	6.86143619996875	14.44057990658391
C	9.99127551136361	7.16709499000263	16.38764170198102
H	10.78217191436191	7.08995031702719	17.13559873037772
C	8.59616759553251	7.42567801729260	16.67319988047337
C	8.03537326415273	7.49739595729456	17.96776750878842
C	6.68175543069574	7.78053630997160	18.23592156051598
C	6.05772105542990	7.69391729386223	19.54397330196031
H	6.55585870033196	7.36118004275252	20.45614695570786
C	4.74772013931885	8.08256348687848	19.39274529703041
H	3.97036403694105	8.12680866654002	20.15736748798952
C	4.56374543113365	8.42484461364985	17.99256651011717
C	3.38246905360820	8.96187034491648	17.43853999238208
C	3.19606035625986	9.23201858633861	16.06129530454229
C	2.02753504323089	9.87482252179064	15.49529297136155
H	1.20187117868025	10.29648573295586	16.07075432361547
C	2.17625156671756	9.84840984744041	14.12584203001242
H	1.49840604668267	10.24997544607578	13.37093205034567
C	3.43182193293984	9.18342463907155	13.84905462566084
C	3.89275834939834	8.82914771403037	12.56180019684091
C	9.52884848588032	6.64380389280343	12.16268768938142
C	9.94092124576718	7.42757597275932	11.05852570331829
H	9.48285134346568	8.41146282990397	10.90178270318717
C	10.93177374253274	6.97071376811552	10.18143151572457
H	11.24812838939642	7.58786695612277	9.33391602899048
C	11.53697776130621	5.71233768388517	10.38907142551302

C	11.13699455550282	4.92648307559575	11.49119317429714
H	11.61083526547963	3.94885430540416	11.63784657688208
C	10.14872138553796	5.38712640078991	12.36654717311071
H	9.83329509800502	4.76662300863882	13.21319525026101
C	8.91823796760526	7.19024114120217	19.12896275350726
C	9.13524803340855	8.14712891503308	20.14711924315190
H	8.66400127284643	9.13361798340435	20.06197399076752
C	9.95141978025680	7.85177731279245	21.24627790489813
H	10.12167804994934	8.60017735157299	22.02731674593492
C	10.56595780754523	6.58559671835960	21.35152112770799
C	10.35249933889034	5.62477750096497	20.34029688610607
H	10.82931561513589	4.64283717801661	20.44044574620385
C	9.54054690668673	5.92463565115047	19.24164325223237
H	9.36501332310003	5.17224244290915	18.46377561943637
C	2.24399346114106	9.24254677221939	18.35699423423691
C	2.37804841560293	10.15929434342359	19.42585734338117
H	3.33296202826836	10.67979588855429	19.56624047838817
C	1.30471788703155	10.42027086573775	20.28634640122702
H	1.41272573880665	11.13898022307259	21.10550043477511
C	0.06935918674824	9.76389215111912	20.09730037876279
C	-0.07205346356474	8.84570199131122	19.03461458336024
H	-1.03485668647631	8.33765964010011	18.90538589141330
C	1.00069303339692	8.59094439982577	18.17447230648570
H	0.89291366555829	7.86984136692756	17.35548808438249
C	2.99705547107824	9.10859368072707	11.40277583655243
C	3.41011397766494	9.97254046940170	10.36195890376611
H	4.39477428060873	10.45117084190691	10.42318814499941
C	2.56931983156265	10.23033196340398	9.27366821913694
H	2.87775175175707	10.90579109639300	8.46722569162689
C	1.29647360920573	9.62670639035429	9.19830919060169
C	0.87716869274327	8.76023152948716	10.23105984813843
H	-0.10714069256977	8.28442886648891	10.16947598610494
C	1.71936238207789	8.50774212676139	11.32064390161796
H	1.39855280700951	7.82641759982920	12.11752992688033

C	0.44163661001910	9.93819576474028	8.01481080649631
O	0.76691692200942	10.68006664181700	7.09831657310623
O	-0.75445949253281	9.30207974647014	8.06398172359125
C	11.44083344772181	6.21017056212585	22.50150819442619
O	11.99300686200275	5.12631774012661	22.62938830368360
O	11.56007532316597	7.21311177540782	23.40578002703994
C	12.59094265569254	5.16859368393783	9.48432780155067
O	13.14319752969421	4.08662481912212	9.63025034685402
O	12.87670309595918	6.01310674870690	8.46206642812875
C	-1.10910210268685	10.00195056075305	20.98090316840304
O	-2.19589757638456	9.45481158666432	20.85370579384582
O	-0.84504027915239	10.90556577041592	21.95704643999499
C	-1.93419570869090	11.19404673918249	22.84998603269598
H	-1.55404353537194	11.94287992324303	23.55985843551419
H	-2.79938683475185	11.59470567479004	22.29342127353623
H	-2.25172837211975	10.28232336332775	23.38570736315215
C	-1.63903965344313	9.55170151900238	6.95822818861793
H	-2.54656176992675	8.96373168895824	7.15771075284574
H	-1.88360554835376	10.62611969450487	6.88935164521945
H	-1.17528056282148	9.23417230279114	6.00797390526839
C	13.88151249452272	5.55711610398678	7.54061500504170
H	13.98113316867066	6.34909998077035	6.78428051105928
H	14.84308830848470	5.39671837062081	8.05900582632521
H	13.57436011357115	4.60826093076548	7.06705060723853
C	12.38640130446893	6.92671569253649	24.54730865929675
H	13.41650440037603	6.68449229230247	24.23261006276268
H	12.37709337566399	7.83702192925637	25.16404125664572
H	11.98166190491407	6.07161242590379	25.11641583509381
H	5.42295743395383	6.63537154338857	15.31294034103154
Si	4.80779055366096	4.93532334498636	15.54155306507493
C	5.32466819897601	4.64459928737047	13.74648248983706
H	4.61345299801954	3.93709734978469	13.28026359738125
H	5.24640409395937	5.59347991664996	13.18881963715366
C	6.06908844135959	4.71659975126708	16.91526891139908

H	6.25810413742056	3.65419781203101	17.14336461769017
H	7.02252699795683	5.19462584627210	16.63650116633469
H	5.70498825657110	5.21927881248068	17.82854968788885
C	3.11004438568275	5.65911080725183	15.94641965487420
H	2.55686535436237	4.96024956275504	16.60219817323237
H	3.27230517128871	6.57653476853183	16.53983208666804
C	6.75829247566385	4.09948772517542	13.67585902849373
H	7.07440346515206	3.92283538316084	12.63149837855812
H	7.47615780922386	4.80896040786969	14.12196434388795
H	6.85154080458783	3.14112309675102	14.22161608300252
C	2.30099656546971	5.97144480684051	14.67821849401272
H	2.05740484086579	5.05343789550376	14.11209315758696
H	1.34714156180084	6.47524027754831	14.92065317192776
H	2.86165345983233	6.63867017125706	14.00154158202782
C	3.92176896015164	2.73543042397204	15.76870906908689
H	3.74539958360141	2.89866974597856	16.84211644166453
H	3.06766829319164	2.95462459654694	15.11126270876235
C	4.85423788557801	1.79377823940190	15.37235697402091
H	5.61022903015210	1.40595400754574	16.06653208754090
H	4.92189039004043	1.45694139644711	14.33069845244971
C	6.71476476394617	10.52377674158737	15.88113680123070
H	5.79273931340799	10.90120360031204	16.33925852101107
H	7.54697594638376	10.29997438398118	16.55944594463141
C	6.85431488759789	10.43303537192267	14.52969591847642
H	7.80431613568638	10.13352417092166	14.07073894014645
H	6.05184549763878	10.73960451428054	13.84796753340507

6-TS-MOF (dimer model)

O	6.64332001047661	8.55736603328983	12.11972600947056
O	-6.64331999967597	8.55736601021384	12.11972600177412
O	6.64332003810068	12.11972628764197	29.95463443646558
O	6.64331998552927	26.39227401484816	29.95463403057785
O	6.64331993380942	8.55736581297579	26.39227392862314
N	2.02313540488987	19.10830931061423	28.88325439677295

C	6.43422232501312	12.73269077241411	28.84826379336955
C	5.28912249073753	13.66461772425653	28.80162366784386
C	4.46121420460695	13.80978493634625	29.93017163731833
H	4.65682053925758	13.19722423080555	30.81665045771688
C	3.43548914561802	14.75359309062295	29.90390622335335
H	2.79162559204190	14.91323275497820	30.77677970682503
C	3.25802086317869	15.55552915889504	28.75851640419246
C	2.26981212549743	16.65260973646075	28.80142084404524
C	2.78168679261331	17.95424356493937	28.85674833484848
C	4.19395165927008	18.28523257877098	28.88137071686677
H	5.00898455158150	17.56058152571115	28.90320983669433
C	6.49584496081556	25.69024412172781	28.97166700009240
C	5.57388366564160	24.53589303974627	28.91484138782884
C	4.83555205633504	24.26817303160482	30.08264134249618
H	5.01662050600070	24.88514142304536	30.96966748907471
C	3.88241543024158	23.25369869478599	30.07149649762265
H	3.27805346122446	23.03655304027291	30.96004412725303
C	3.67220504962894	22.50892194189916	28.89337860693766
C	2.57945657648794	21.51355109890983	28.88020193463574
C	2.92129429148503	20.15890763155159	28.87890112775199
C	4.28009523814293	19.65482957387212	28.89171798773205
H	5.17711432008439	20.27556900721514	28.92360789081984
O	7.13627387549823	12.61267951147867	27.81336568089086
N	-0.12946429954786	17.20862199166343	28.96031963430809
C	5.07950821341128	14.42278205511533	27.63379636536498
H	5.73876588583580	14.26274858648738	26.77368891265827
C	4.06225184891013	15.37560757868535	27.61547524950494
H	3.89244403685194	16.00147188129811	26.73197409221560
C	0.91437006205985	16.31410774893338	28.82264207887807
C	0.40365231415666	14.96192035793539	28.71236852676016
H	1.01762708328791	14.06959951587387	28.57860802801829
O	7.13627401470468	25.89931998473380	27.81336596585005
N	0.12332437662888	21.26618167163297	29.01111397161632
C	5.39813387564824	23.76882743470631	27.74771939420980

H	5.99101969728462	23.99582887250022	26.85585885452419
C	4.44444103447150	22.74738791369045	27.74076934285490
H	4.26440373756143	22.15041439423208	26.83872126617107
C	1.27540285434222	22.02273708336802	28.91749202436198
C	0.94193813667645	23.43370441565731	28.85938331828535
H	1.66470886727713	24.24607574780879	28.77106151474865
O	-7.13627438802847	12.61268013566864	27.81336590054624
C	-6.52769807232487	12.82016807927531	28.89358738295003
C	-5.58493090898224	13.95635878982524	28.96808468207620
C	-5.41383750419351	14.79103348796690	27.84808909740591
H	-6.01558733200755	14.60659950244498	26.95123937444309
C	-4.46198080944979	15.80950491678051	27.89997850033366
H	-4.28550009623177	16.46291143851934	27.03742774985539
C	-3.68132812385722	15.97166821142505	29.06165587673132
C	-2.58371340263520	16.96094997487919	29.07015691983925
C	-1.28486058733131	16.45241111988082	28.96152326840247
C	-0.96343143194653	15.04719992023949	28.80034205785965
H	-1.69367649602512	14.23808138560744	28.75036489321991
O	-6.64331981592480	12.11972594127121	29.95463399530425
N	-2.02448042438581	19.36371214555908	29.11045460289892
C	-4.84296125378884	14.14872095991376	30.14897312664914
H	-5.02286575778141	13.48519722764550	31.00122592986710
C	-3.88312532212479	15.15797974923358	30.19324147147608
H	-3.26895405529017	15.31738876566688	31.08727869866387
C	-2.92148397492708	18.31325066090623	29.15234128051089
C	-4.27513605275025	18.81465101548704	29.27971670234073
H	-5.16677094388065	18.19144132438958	29.36806708052650
O	-7.13627401584549	25.89931998690631	27.81336595404832
C	-6.43849235910247	25.74689042197101	28.79867547220737
C	-5.29419644908923	24.81511216703213	28.88199084747951
C	-5.06949491573156	23.97763721472633	27.77437438108159
H	-5.71205763392609	24.08719631316624	26.89363277806599
C	-4.06312092822577	23.01669915284938	27.84003620064675
H	-3.88523618661415	22.32923344688770	27.00508659934849

C	-3.27839732920276	22.90693493758751	29.00626137915386
C	-2.27976372003108	21.82122842604795	29.08453304774774
C	-0.92696502528905	22.16460886836262	29.02042939580074
C	-0.42632371553412	23.52050796120266	28.91957889096840
H	-1.04813771517918	24.41658053130894	28.88827649552475
O	-6.64331999059254	26.39227401488554	29.95463404360304
C	-4.49264923804486	24.73191534568772	30.03649726153515
H	-4.68998537838805	25.39894458559023	30.88135280790184
C	-3.47505342392559	23.77804654369990	30.09397855106927
H	-2.85004427009948	23.67310141702636	30.98863099262712
C	-2.78325246191400	20.51684582393608	29.15947433971660
C	-4.19020095555546	20.18436292997126	29.28262028909954
H	-4.99990643019002	20.90958681914034	29.37603699281056
O	7.13627416777492	10.69863436959970	25.89931994544828
O	-6.64332023374424	8.55736604279354	26.39227388319956
O	-7.13627400155919	10.69863399304564	12.61267999679241
Rh	0.00225905532675	9.66847480708982	19.26354770462156
N	2.02244007018782	9.58163490557482	19.42921007464387
C	6.54640413233969	9.66544653878311	12.87275565157294
C	5.62761591639450	9.50124896460019	14.01770596497100
C	4.84675818193914	8.34226108636410	14.19053611962641
H	4.97061110245619	7.49851922159893	13.50399131857934
C	3.89601941637527	8.30706925139509	15.21317154118116
H	3.24714186032453	7.43359518537019	15.34762827757234
C	3.73566377938765	9.42004610737422	16.06252605388708
C	2.62363111306145	9.46354057616963	17.03738613558408
C	2.93755500561881	9.45229099392427	18.39989012175946
C	4.28157583641646	9.33581934942533	18.93256764906271
H	5.18347997837595	9.19514614776188	18.33384728428141
C	6.42068136011023	9.66643445404652	25.78906217828771
C	5.26513956493189	9.71398603171921	24.87435940912263
C	4.41969708498802	8.59313149999151	24.75506186194371
H	4.61342269109869	7.71164742816043	25.37575311067987
C	3.38459938160559	8.61514106298111	23.82030065807288

H	2.73089456379242	7.74619575281056	23.68076243332175
C	3.21260079495644	9.74472019777234	22.99400836003997
C	2.23152473754462	9.70705061594746	21.88803333780924
C	2.76279485408827	9.59077975154551	20.59670222113311
C	4.17496014350806	9.42984220957957	20.29777458499492
H	4.97399641509452	9.37670998370556	21.03918377184836
O	7.13627399364561	10.69863396918804	12.61267999195112
N	0.16848555399960	9.66998785951841	17.24207398239180
C	5.50659815842399	10.59527256499667	14.89629171465478
H	6.13406905584742	11.47702050173769	14.72462200290026
C	4.56808189888912	10.54991673287007	15.92488345710239
H	4.42954854408316	11.40222103852523	16.60041595016706
C	1.33539817102148	9.60286425767451	16.50289824525526
C	1.04018315850694	9.73316835355230	15.08670394789813
H	1.78301728873939	9.74334299002330	14.28750633081911
N	-0.16179005056618	9.68283925356408	21.28667385867986
C	5.05754238187112	10.87069264360528	24.09686144947274
H	5.72810927710697	11.72646545687100	24.23060120721973
C	4.03297708580936	10.88117420020576	23.15230077432136
H	3.87020555313686	11.75110607844670	22.50536586541483
C	0.87097609503738	9.74751223601377	22.20514179983533
C	0.34218983737442	9.83941846476077	23.55191727444003
H	0.94468693195474	9.92971601816813	24.45792078433147
C	-6.38241715831633	9.59060103991195	12.70935427357441
C	-5.23786932623806	9.76082311298922	13.62809898300210
C	-5.05304465162316	10.93767858524379	14.37937924297067
H	-5.72988767489760	11.78544786825920	14.23241200464013
C	-4.03054432396231	10.98757193404674	15.32917877210637
H	-3.88623888296477	11.87771802189673	15.95281355044938
C	-3.19321873920285	9.87022037403194	15.52149352574688
C	-2.22003659387514	9.84719493145068	16.63569481221397
C	-0.85736486287376	9.80224478860808	16.32315077123816
C	-0.32155305208856	9.86215608790785	14.97649650334618
H	-0.91565374133918	9.99416439949355	14.07028543679417

N	-2.01990737312723	9.73728526624923	19.09760610587022
C	-4.37040885279112	8.66166878415308	13.77879331365937
H	-4.54938086572326	7.76570395767941	13.17378521044660
C	-3.34322258257533	8.72024885091647	14.71865068245636
H	-2.67818634274501	7.86383703175068	14.88063024953712
C	-2.75792387495947	9.77903293575305	17.92883710299867
C	-4.17652923341565	9.69387215414169	18.22887147855185
H	-4.97674050864615	9.67555079855630	17.48735502630639
O	-7.13627357416872	10.69863388222439	25.89932022711879
C	-6.52566151228530	9.62043633394123	25.67754881430036
C	-5.59969196030259	9.57112795345116	24.53207795135590
C	-5.50513888420635	10.69406381079788	23.68447108490097
H	-6.14726438623864	11.56015021038565	23.88001197583420
C	-4.57436847039880	10.69003964910804	22.64665000357832
H	-4.45452481739413	11.56164888767745	21.99264243308140
C	-3.72787876218307	9.57562700380782	22.47033330574125
C	-2.62398367311453	9.63246079782354	21.48949059516164
C	-1.32934112435062	9.68862233286165	22.02562485916535
C	-1.02576125463498	9.79529398419525	23.44225230565686
H	-1.76513679218162	9.84936609865007	24.24292349396195
C	-4.80561456621457	8.42769174659986	24.31874200603207
H	-4.92487988086720	7.56469422972735	24.98243759255496
C	-3.86592959378086	8.43467350188294	23.28918696223713
H	-3.20968169448087	7.57336773836627	23.11864112954832
C	-2.94080511231581	9.65887454983826	20.12758324616760
C	-4.28887588773300	9.61252427414155	19.59429547515182
H	-5.19838922878577	9.51853476603046	20.19064498175830
C	-7.74879445433440	27.31356156519178	29.97736034467517
H	-7.60939372794806	28.10668058392540	29.22242303486217
H	-8.69585345579324	26.78733137263322	29.76644291206195
H	-7.76420873903738	27.74340662255184	30.98907042564365
C	8.03944569226926	27.01952098151048	27.78418542079896
H	8.46909615730641	27.03575897960834	26.77240634403741
H	7.49816954494964	27.95927841002868	27.98977066569298

H	8.83450450422693	26.89689421489306	28.53989934787941
C	-8.28897087747447	10.60287445850325	11.75920443529509
H	-7.98881724767601	10.36554129681168	10.72361732474797
H	-8.97383675170608	9.81394073497922	12.11627873558912
H	-8.78002450584319	11.58581859584631	11.80116975114619
C	7.49680125711129	8.65238070514349	10.96689522753185
H	7.45977828924952	7.66712816298629	10.48012958711154
H	7.13591931554681	9.43646961532028	10.27842914653164
H	8.53098874307332	8.89619239842763	11.26653476691429
Rh	-0.00411980422308	19.23708103043451	28.93217821134438
H	-0.10187394818292	19.30109443741598	27.37358304891013
Si	-0.37276858890363	19.32072582745818	25.47343999539490
C	1.48432692336920	19.66009885536587	25.45408401222717
H	1.70904342413213	20.28588542751057	24.56929580815850
H	1.73792832164763	20.26873612319999	26.34008928362875
C	-1.05128975513226	17.58911555766680	25.70491074301506
H	-1.49843292235359	17.19150125004848	24.77825461424969
H	-0.25881464385559	16.89655578601791	26.03574702042352
H	-1.82593299422109	17.60621945055332	26.48992847551913
C	-1.55291955711168	20.76553031702801	25.73748309864416
H	-2.38297753256216	20.68686899339680	25.00981081131057
H	-2.00699917005673	20.62292902367588	26.73466557378258
C	2.30783602179563	18.36533617603095	25.44229521538864
H	3.39129554235992	18.58119478857194	25.45123355118155
H	2.08584401980414	17.74733788024448	26.32817722214316
H	2.09653672838983	17.75084388899968	24.54713537980086
C	-0.86347196661373	22.13448120567138	25.64866074741497
H	-0.48248960819110	22.33984644900535	24.63148933666068
H	-1.56235646475231	22.94860746285149	25.91163919693789
H	-0.00985505663188	22.20571420936151	26.34453476446377
C	-0.64513159190892	19.41611302394775	23.16299468086018
H	-1.71165580167716	19.15937252634341	23.23356740890501
H	-0.40963634666120	20.48800867958412	23.09576893729946
C	0.24419112973151	18.49104829591155	22.66473142524588

H -0.02615448139635 17.43301725240484 22.55909113547050

H 1.27429170039504 18.76741191526440 22.40900624443811

Zr -10.13922723502628 8.20413712019426 30.89596801823864

O -12.26053310413953 7.82704904525194 30.51822742078518

O -8.68782508507440 7.81015934282309 32.74851302345112

O -9.13720078179597 10.12672975934832 31.22946796934562

O -7.99931642211119 8.04405440201323 30.12725335179486

C -12.97828547636953 7.37764225074617 29.56875873061438

O -12.77429717356464 7.54104417356920 28.33426584504711

Zr -8.11871723470399 8.08108596341987 27.88301629637208

O -6.83744361773423 6.54285112718918 28.36748256713998

O -10.10983774593924 7.56300298782572 28.67926623745863

Zr -7.23000390451084 10.16811292890717 30.62586790507645

O -5.27626728158373 9.66386727137875 29.55774973792965

O -7.97140156242388 10.00222038174801 28.66686378149555

Zr -11.44062222031098 9.06693604374981 27.36123697361361

O -13.24458499443397 10.13913383763738 28.31153145093854

O -10.04136376836237 6.22303044251383 31.05619636266840

O -10.66391274883651 9.87532957813218 29.37468908030390

O -4.94751996358710 7.25156204725782 30.14706301149231

O -6.37947122477054 8.79106541554384 31.98918460856002

O -12.53991268084891 9.40857720085435 25.74041167417787

Zr -10.55367272113723 11.73709689378661 30.81605828518317

Zr -8.89424910195109 11.35947273659253 27.16401697487644

O -7.03764536191993 11.43184611430041 32.44320842210237

O -8.88792986309054 6.64699356079020 26.37272525193756

O -11.16164808642744 8.85673755078966 32.72051921784509

O -11.06997717391494 7.21219649808930 26.08612517967816

O -9.43181837680936 9.34418104186443 26.74639487049412

O -9.51291050838607 12.34027837967695 29.03906052320430

O -11.06919445755431 11.23818486194978 27.09230498945481

C -11.42941535975220 9.95101306529210 33.31702426968412

O -11.25916757222095 11.11068900402431 32.85764232281085

O -9.45421953049564 10.03571134417598 24.03231387275024

O	-9.10658984729774	12.50161884521772	25.57065334868030
O	-8.90600862624963	12.68608046095163	32.15940487379711
C	-7.84642973721701	12.36363630741642	32.76140939771189
O	-11.05179846588976	13.65478723192948	31.03844520760758
O	-12.52013832602661	11.46127585494763	30.35041292612075
C	-10.03021875704743	6.54860488492389	25.81588793754472
H	-11.39363087847988	11.49768049906141	26.21075362461011
H	-7.60373235531005	7.20210440132677	30.41903821203928
H	-9.46301147479888	13.31307101580826	29.02005634490502
H	-10.33407235759107	6.61435752702621	28.63033883150867
H	-9.16255880290373	11.98628349486203	24.73352424034895
H	-10.45274683077208	14.18781158178492	31.59035279361708
H	-6.54266417970401	5.90830756563808	27.69217068350591
H	-9.48323536553282	5.82727337801268	31.74701363400006
H	-5.85728862561497	9.19116686903819	32.70808364900084
H	-9.02653623163657	8.32539437889485	33.50235965843120
H	-7.74951084279451	8.16936108280531	32.55984708131121
H	-5.18692546400328	9.88120798992321	28.61309787514465
H	-4.99607008648183	8.65983137785246	29.70223263773600
H	-5.43713746902034	7.48903530514809	30.97486602397654
H	-5.62294312721731	6.85261593147293	29.51661531728429
H	-12.60086254104888	8.69950850309379	25.07732431440518
H	-9.48110659675572	9.60584368601295	24.92279026239693
H	-8.58623605096353	9.78038286598213	23.67708024963011
H	-13.14023912666430	12.12908370377508	30.69055243251504
H	-9.69160744559912	9.97213075556644	29.17415245041835
H	-12.97285685144184	10.67944792149479	29.15088753953908
H	-13.48147533907575	10.81091071164991	27.64709138901542
H	-13.87261730659593	6.78432804466677	29.86295815947266
H	-10.11752284216810	5.78368329879321	25.01090550528165
H	-11.85162365868810	9.85999953998504	34.34254329329418
H	-7.57407900425965	12.95322433060474	33.66766278896574
Zr	10.22333991041352	7.65437924796639	30.27308556291559
O	12.33631539491632	8.06865737077412	30.65184490319337

O	8.81068220816769	5.77955224286181	30.68230896178169
O	9.20814140618312	7.31216001173486	28.36315116987206
O	8.07752854659165	8.39600325493871	30.44600162594216
C	13.04915551668839	9.02873202806162	31.08290252687585
O	12.83544686298618	10.25964497976554	30.90087973411606
Zr	8.16086324907181	10.64206270078883	30.40909534986870
O	6.90811608846078	10.13523556636605	31.96394979125243
O	10.16542280584353	9.87644727148526	30.91888105848112
Zr	7.29136790875498	7.88279506372489	28.32521982287827
O	5.29946649146948	8.85949045935828	28.87836369884369
O	7.96347383259094	9.87095293368669	28.49516624805622
Zr	11.46946433660652	11.21140447839558	29.39952492568366
O	13.27186263284033	10.28886935132810	28.29398077512545
O	10.13187705738724	7.49632709196024	32.25627968319657
O	10.69826081493201	9.19705408831086	28.59544796304543
O	5.06585248686153	8.27696781293379	31.30813922601281
O	6.49955968003699	6.48572106861377	29.70509876621566
O	12.50948846997541	12.86491885335509	29.03867994682160
Zr	10.63043095273898	7.73518943262117	26.75510980717529
Zr	8.90503447995113	11.35621826274974	27.12364778682348
O	7.13469466106396	6.08188210999236	27.04205458232403
O	8.91118059673160	12.17815389416747	31.82843632220729
O	11.27196671584228	5.86061167843242	29.59424744984063
O	11.09105119853420	12.47961129569968	31.26055082330533
O	9.45246380889431	11.78410092109194	29.13368113093016
O	9.53461942872363	9.48303406878609	26.14827863978419
O	11.07993315731576	11.45394662160033	27.22527581168215
C	11.48203680997955	5.22563595812923	28.50758021179816
O	11.26473880878242	5.64949416567513	27.34310780596964
O	9.38478589563369	14.49637245992560	28.47029012579672
O	9.08947588432105	12.95710096535082	25.98858362244614
O	8.96936036170762	6.45668893209008	25.76450802789442
C	7.93291101803313	5.81397231906961	26.08573380148565
O	11.22002426193255	7.47844119805370	24.87597919496830

O	12.59282240339556	8.11973693608149	27.16741812884943
C	10.05013446224687	12.74237059269823	31.92491859725827
H	11.37900079622703	12.33872403423841	26.94661542700501
H	7.70249327455211	8.10511944706963	31.29741530506726
H	9.14856290913474	9.36899368884080	25.26160947007126
H	10.39352700999805	9.92342194124049	31.86670276178523
H	9.16044741770550	13.79299053745068	26.50330996406674
H	10.65600277376089	6.94565849852584	24.28898943332708
H	6.61367296396794	10.79666097887614	32.61271041052365
H	9.59047436879949	6.78881182034179	32.64602232070656
H	5.98444877158648	5.75932478129303	29.30938085552397
H	9.15808145032493	5.02326025248890	30.17695487848852
H	7.86772903389753	5.95094855461765	30.31968286765509
H	5.12231845248403	9.78729060948609	28.64625386716560
H	5.05836739056577	8.71296318908433	29.88946407209088
H	5.56662139671785	7.46120260663596	31.05373523886530
H	5.73546611781328	8.92604255068063	31.68426898803568
H	12.56482631300249	13.53070383493886	29.74554310174720
H	9.44169487913081	13.59752320346553	28.88083974638579
H	8.45482180364631	14.74364239103588	28.60568333672007
H	13.24723470490301	7.71250060600723	26.57383996439250
H	9.72205139663871	9.36938144530837	28.50861885556946
H	13.02979954156384	9.38077227065132	27.86069934680481
H	13.36827972777190	10.88582750837137	27.52908630293256
H	13.94867042578767	8.75117517177489	31.67621228119855
H	10.13304317271527	13.54802503289815	32.68991349586607
H	11.89637403747155	4.19814975336443	28.61358837189355
H	7.67404933664298	4.91975266917734	25.47306178291257

13-TS-MOF (dimer model)

O	6.64332001103041	8.55736603548380	12.11972600929308
O	-6.64332000064399	8.55736601065557	12.11972600058853
O	6.64331983175341	12.11972587415293	29.95463420426999
O	6.64331998886805	26.39227399962811	29.95463401636033

O	6.64332008763375	8.55736591869673	26.39227398301756
N	2.03631535357255	19.08378089957785	28.67182889938498
C	6.42513584216170	12.72121575861238	28.84333715832404
C	5.26154185041265	13.63118928066362	28.76923997937447
C	4.45305952338922	13.84368044648385	29.90083498559232
H	4.66774734176317	13.29052629315147	30.82159046957650
C	3.42478814853896	14.78434409155186	29.83473727507106
H	2.80223082704702	14.99991082197138	30.71118892615412
C	3.21868168880148	15.51290222773050	28.64471029572577
C	2.24120155284055	16.62207969065849	28.64744853470515
C	2.76906945699035	17.92125199163032	28.59468809117622
C	4.18422202895024	18.23814290493836	28.52478076472285
H	4.99107146896506	17.50640990364137	28.46866679046540
C	6.50532306963630	25.68255969510810	28.97545647708683
C	5.60620534329536	24.50895532241090	28.91831122116468
C	4.90233833810604	24.19051416977053	30.09446044320100
H	5.09632526351967	24.77963517086038	30.99773332521324
C	3.96074096730255	23.16414538788240	30.07191745756787
H	3.38247549421029	22.91085494919582	30.96826358573176
C	3.72048470842513	22.46214097836095	28.87260924246896
C	2.61703099219859	21.47906176684001	28.83278974194194
C	2.93825707069214	20.12549579912275	28.67565357486660
C	4.28839814218568	19.60612808314679	28.57372517655343
H	5.19467129517503	20.21383346031186	28.56585092383982
O	7.13627428184877	12.61268012753037	27.81336571397630
N	-0.15114064394267	17.20117441584856	28.88264686952176
C	5.01984795619660	14.31099662584561	27.55928496088450
H	5.66175688717455	14.09921430215132	26.69746553116568
C	3.99607141278626	15.25459996807889	27.49808316704850
H	3.80516805042749	15.81741739077616	26.57759576463124
C	0.88413691491937	16.29930913579611	28.76398056933288
C	0.35998021299171	14.94654079434003	28.74277821134055
H	0.96264470283681	14.04371478378208	28.63012080808914
O	7.13627401274732	25.89931999158645	27.81336597586270

N	0.15562115629814	21.26696231962958	28.99606952445149
C	5.40962595326226	23.77707534766385	27.73157428418305
H	5.97512721941929	24.04209318430270	26.83261029165450
C	4.46358718919208	22.74961796705369	27.71105561690817
H	4.26119922834518	22.19077468723795	26.78951786218497
C	1.32068864666407	22.00167050483353	28.96326936404848
C	1.00832466085059	23.41995058718470	28.99398127565435
H	1.74251445963641	24.22577213285323	28.95891451529689
O	-7.13627408319980	12.61268005080183	27.81336602543003
C	-6.53487986968886	12.82478509397444	28.89601826275193
C	-5.60699526464252	13.97490133578016	28.96856231596706
C	-5.43235237413369	14.78996301672685	27.83455578759338
H	-6.02356933018550	14.58237924050551	26.93584241064577
C	-4.48594817782449	15.81339979480415	27.87183311169010
H	-4.30153059932515	16.44244330938890	26.99306449060983
C	-3.71464093541045	16.00612850614672	29.03586794636178
C	-2.61095372652147	16.98908607770555	29.01651664031581
C	-1.31312069330472	16.46395243446134	28.95030730769668
C	-1.00381299724688	15.04803990217126	28.85879776177080
H	-1.74049882406964	14.24343709014613	28.85707684297250
O	-6.64331997681414	12.11972597732214	29.95463392842355
N	-2.04170884967989	19.38917354569629	28.95369186512237
C	-4.87778845175659	14.19816413436372	30.15160042793379
H	-5.05874063236629	13.54961083215834	31.01534108573904
C	-3.92593684858452	15.21676721057345	30.18346293151369
H	-3.32165140975131	15.39758935186486	31.08027373079986
C	-2.94181640805469	18.34841621690168	28.99513329747963
C	-4.29432295490419	18.86907002138835	29.04843838782362
H	-5.19760252980010	18.25922733777114	29.10471103728734
O	-7.13627400725374	25.89931999668467	27.81336599873142
C	-6.42813149678219	25.75922635945108	28.79329020860553
C	-5.26017363693875	24.85346287349564	28.85810030879856
C	-4.99138231106341	24.07802332128236	27.71523618338938
H	-5.61254980159352	24.22339460712874	26.82436019843796

C	-3.97413692293694	23.12706319042294	27.75797222050289
H	-3.76433003154081	22.48898352606752	26.89171841222235
C	-3.22631528811853	22.95588871030591	28.94149391461620
C	-2.24536940245902	21.85166314053889	29.00369401781931
C	-0.88342391234030	22.17316156089313	29.03152458846981
C	-0.35945710689106	23.52489720499337	29.02844887733688
H	-0.96692156875229	24.43131264402721	29.02914943249875
O	-6.64331999461925	26.39227400551629	29.95463399925258
C	-4.48480194374722	24.72210414804777	30.02608648537001
H	-4.71196247759902	25.34236137008620	30.89882377769603
C	-3.46130103020884	23.77254223888102	30.06424134751014
H	-2.86613467914826	23.62407024227046	30.97312266608934
C	-2.77561094788385	20.55276586421489	28.98007025841283
C	-4.19229144837005	20.23813434098034	29.03404278928947
H	-4.99722114964857	20.97293291551069	29.08001414755139
O	7.13627380164728	10.69863407539230	25.89932010143166
O	-6.64332006778550	8.55736611819835	26.39227393924687
O	-7.13627400221618	10.69863399198491	12.61267999706181
Rh	0.00212816507279	9.67366510381347	19.26426446847426
N	2.01995194780548	9.58813970700567	19.43230728440632
C	6.54684216302955	9.66532033088265	12.87414659062730
C	5.63070370865077	9.50077402894046	14.02074887889266
C	4.84832142247871	8.34278677838186	14.19452046383640
H	4.97008409087258	7.49894518678618	13.50765627987272
C	3.89830870206871	8.30893544340409	15.21790429705286
H	3.24812272027498	7.43646628717342	15.35253818238129
C	3.73880116628115	9.42200547399312	16.06769273342382
C	2.62597641518283	9.46718400642614	17.04161408787357
C	2.93806256651351	9.45902420041950	18.40437037604131
C	4.28161927556359	9.34458623667251	18.93840572111309
H	5.18438475316384	9.20376569317255	18.34089525489304
C	6.41752776487394	9.66718129555115	25.79029116009299
C	5.26025860584693	9.71746275411585	24.88009267779627
C	4.40798248335385	8.60079457424756	24.76629252318868

H	4.59709554310575	7.72053838079541	25.39018973686920
C	3.37287990006664	8.62428410926785	23.83176091514457
H	2.71462079991083	7.75819338231490	23.69616679003381
C	3.20689945236164	9.75006519711066	22.99844738674281
C	2.22754364442948	9.71187258814547	21.89146703585632
C	2.76067692853705	9.59904512116399	20.60064330260997
C	4.17347397460584	9.43967745115659	20.30352258571817
H	4.97208477970674	9.38683713412200	21.04548603910941
O	7.13627399154089	10.69863397696245	12.61267999293950
N	0.17074681660835	9.67450566818911	17.24520107106351
C	5.51169640503370	10.59443066123575	14.90039417193638
H	6.13984281508558	11.47568120217807	14.72850812920630
C	4.57378601020072	10.55008557117954	15.92958345260918
H	4.43659061977324	11.40253758594641	16.60519636866320
C	1.33837797333616	9.60488795388083	16.50566902622355
C	1.04495395957261	9.73210452837420	15.08876326879126
H	1.78851342253453	9.74023649722836	14.29015672403393
N	-0.16455429044136	9.68460007395559	21.28520053270751
C	5.05769864276035	10.87191056187588	24.09723910415145
H	5.73308543251187	11.72466524445730	24.22591218179197
C	4.03335399266459	10.88309954651498	23.15276781976114
H	3.87552127501455	11.75033731224299	22.50105268054790
C	0.86652798117230	9.74790980226127	22.20690175108681
C	0.33593766679335	9.83650977917154	23.55312988824642
H	0.93672043991144	9.92615189228950	24.46042816118118
C	-6.38110162089413	9.59068265337921	12.70906426074110
C	-5.23544489595293	9.76231729252441	13.62557038722790
C	-5.04964072997886	10.94030810926173	14.37514764856667
H	-5.72648936113492	11.78798640396106	14.22749680034262
C	-4.02625861373476	10.99167026738180	15.32388944805195
H	-3.88152394103859	11.88270117799678	15.94615674696754
C	-3.18867364544430	9.87470233222536	15.51845979948676
C	-2.21644994348766	9.85272645610856	16.63353378353311
C	-0.85367288555799	9.80531213674409	16.32310225511972

C	-0.31661508801684	9.86173514292975	14.97681626990836
H	-0.90958593762701	9.99242918750760	14.06959470156811
N	-2.01767629412770	9.74204341222003	19.09553143774517
C	-4.36746201463856	8.66355797152795	13.77773810902893
H	-4.54655581021405	7.76643126695674	13.17444020159960
C	-3.33953408935947	8.72389152066888	14.71670662870215
H	-2.67451793582925	7.86771406652192	14.87994478945769
C	-2.75585732154783	9.78648415436209	17.92592935051675
C	-4.17503075850286	9.70370286428985	18.22383740402133
H	-4.97447817665059	9.68741482498871	17.48137557856824
O	-7.13627387982907	10.69863385075172	25.89932010048647
C	-6.52642564761232	9.61933344310504	25.67530738709130
C	-5.60416867689703	9.56927705414134	24.52821972935384
C	-5.51110127403484	10.69165227643684	23.67913746813353
H	-6.15327458206258	11.55778813386003	23.87450959958476
C	-4.58099915655611	10.68770519637703	22.64086689707872
H	-4.46233712652726	11.55881531954925	21.98600336840033
C	-3.73192305993906	9.57486106153637	22.46504037269946
C	-2.62694750352669	9.63311340221076	21.48595032890843
C	-1.33314036046237	9.68661900726842	22.02415656540627
C	-1.03199075746490	9.79038773922240	23.44144230319730
H	-1.77241385121389	9.84188910166135	24.24141425154733
C	-4.80881290728884	8.42643532763108	24.31456758076715
H	-4.92654442785294	7.56350681260140	24.97865566758927
C	-3.86952715811721	8.43417332000397	23.28485342373374
H	-3.21220160212626	7.57369797120320	23.11443885292674
C	-2.94171780609187	9.66403876146296	20.12389020342962
C	-4.28927539429056	9.62057070826076	19.58907044166852
H	-5.19988270698019	9.52658326294732	20.18383751519076
C	-7.77561647940162	27.28035811170701	29.98981711876821
H	-7.66692150291536	28.07801368767801	29.23473246268927
H	-8.70860913356113	26.72641875302225	29.78733182383631
H	-7.79362668962933	27.70804572062356	31.00237596421336
C	8.01509306286042	27.03868331337236	27.77423356772426

H	8.44310466637982	27.05624167013511	26.76181424349496
H	7.45375835519473	27.96804719215953	27.97317577119608
H	8.81326301619648	26.93879805632303	28.52993006160582
C	-8.28960741754134	10.60108196222011	11.76083935035280
H	-7.99095106453806	10.36120110162083	10.72535646203835
H	-8.97449310030627	9.81313844083525	12.12024418126118
H	-8.78061623080446	11.58418167916884	11.80099464530270
C	7.49295951722429	8.65495407655072	10.96468817061587
H	7.45271054772490	7.67147456688068	10.47450328149124
H	7.13152350381037	9.44212648021732	10.27999096652971
H	8.52871142357320	8.89587307188051	11.26148134254316
Rh	0.00367408877068	19.23339846795423	28.92268529572140
H	-0.10416119322764	19.31357361425578	27.33965622725552
Si	-0.39764446741077	19.28567001211917	25.43078639168802
C	1.48319918217218	19.30163187991889	25.32369805101789
H	1.76769810527538	19.91507945297850	24.44786587145964
H	1.88494552565494	19.81836980121582	26.21247622742128
C	-1.31726697720987	17.68291373469265	25.73271752951848
H	-1.69228531781586	17.23351463286144	24.79871189053974
H	-0.66407404171201	16.95043880511681	26.23517403743440
H	-2.17408408039757	17.87674987573303	26.40002264867455
C	-1.31271572304485	20.91395342989772	25.68442006952063
H	-2.10866677102021	21.00343800304393	24.92132079117068
H	-1.82950909835603	20.83960470689756	26.65692617198861
C	2.06502140100101	17.88556945937903	25.21820261997439
H	3.16566946456612	17.91083217752399	25.12477390815440
H	1.81948425882361	17.28693499000714	26.11129227249654
H	1.66939452472786	17.34339497834584	24.33880614403957
C	-0.38107402412199	22.13431225247263	25.65830259756445
H	0.11353083954960	22.25424335096532	24.67689832626322
H	-0.93878275912844	23.06513189419556	25.86509099468240
H	0.41097079916775	22.05174407664839	26.42235218071400
C	-0.75289878332347	19.38058957677782	23.14064405267057
H	-1.83959288589891	19.26563066519335	23.26385154939319

H	-0.38737794534207	20.41193648884015	23.03108028601726
C	-0.02280774883272	18.34202084759889	22.60149039455385
H	-0.43359437417218	17.32734753569758	22.52799309205328
H	1.01365799868023	18.48073459477216	22.27097300486627
C	-0.50897936991887	19.19375236971031	31.34525246144167
H	-1.00320297884677	20.16340914358986	31.47936549788250
H	-1.14392445865176	18.30279558529577	31.42093446145305
C	0.84933000817645	19.09430509786962	31.24314021978359
H	1.35296236757661	18.12020669681606	31.23145018622482
H	1.49352433165165	19.98036561319051	31.29525416474398
Zr	-10.14932938948556	8.20727831846930	30.89298257138429
O	-12.26781494775005	7.82802632044382	30.50302495069343
O	-8.70908262657059	7.81874918419462	32.75329783210127
O	-9.14685936572169	10.13314678515015	31.22790794159194
O	-8.00583641978905	8.04607505350030	30.13369255343310
C	-12.97758908241527	7.36568173897489	29.55329477551913
O	-12.76887927443236	7.52139907796841	28.31878363661977
Zr	-8.11425126020115	8.08120716196918	27.88674588644324
O	-6.82067962851945	6.55532881536920	28.37600488250104
O	-10.10819378972127	7.56312784785438	28.67835318202361
Zr	-7.23830185754143	10.17062355392123	30.62889611873470
O	-5.28234799694351	9.65566145038188	29.57354275593999
O	-7.97451255621950	10.00318160863485	28.67033157386271
Zr	-11.43713046633588	9.06178993526112	27.35650501672375
O	-13.24389221406104	10.12008956229384	28.31016542034100
O	-10.05159823932605	6.22625999738918	31.05650286563742
O	-10.66972023621877	9.87477131915763	29.37131015166810
O	-4.95455782320258	7.24873252944242	30.18273358032097
O	-6.39819591036833	8.79780580362235	32.00519975035332
O	-12.54753870678136	9.39753144767013	25.74154799386666
Zr	-10.55850812524043	11.74508169056987	30.80502206723715
Zr	-8.89228961177609	11.35758195984708	27.15674316738152
O	-7.04661078975066	11.43951735106400	32.44504476035517
O	-8.87991321318354	6.64180253537279	26.38140485035687

O	-11.18318322524795	8.86431397349039	32.70983873061695
O	-11.05863979310937	7.21095837952863	26.07840277066666
O	-9.42720462348690	9.34105412163279	26.74598680030663
O	-9.51274994171779	12.34151712899854	29.03036555776788
O	-11.06590517558457	11.23103637638435	27.08260179648160
C	-11.45870775118600	9.96002812093275	33.29952681467933
O	-11.28706703526229	11.11826351163406	32.83664759033682
O	-9.46449700882849	10.05185166779416	24.03943229210245
O	-9.10097580049758	12.52045513942612	25.57739259652257
O	-8.91632032525705	12.69120623807042	32.15888656421706
C	-7.85772281316790	12.36910879016106	32.76282603145511
O	-11.04643054441511	13.66740152427549	31.02148221477712
O	-12.52174629701598	11.49474878969428	30.31308155457621
C	-10.01823065186381	6.54527328650593	25.81606526451395
H	-11.40273679666419	11.49648283025807	26.20780685585785
H	-7.61050452462349	7.20606831241211	30.43100390259453
H	-9.49494040174488	13.31510786578900	28.99510570356117
H	-10.33167770068774	6.61431375723791	28.62922281988846
H	-9.18416562284946	12.00887795444302	24.74025666853539
H	-10.44352272321729	14.19302144380470	31.57636997691426
H	-6.50754862958216	5.93122187947269	27.69918084854140
H	-9.49708784576235	5.83232018733563	31.75126152590540
H	-5.88441807800011	9.20373805830719	32.72686999103554
H	-9.05244438112722	8.33593832822280	33.50368695736184
H	-7.76985492704903	8.17865278069947	32.56815242692998
H	-5.18462391287642	9.86392564065142	28.62764265619756
H	-5.00000281285521	8.65375364255349	29.73161276692205
H	-5.44623473495520	7.49060851079797	31.00809521349338
H	-5.63107976673392	6.85392012578343	29.55045620866834
H	-12.60408052386661	8.68887861795291	25.07757447158421
H	-9.47328400549056	9.60768790879149	24.92316952893048
H	-8.59557942864203	9.81863879334259	23.67120268719761
H	-13.13370758151954	12.17990633155514	30.63280080509562
H	-9.69736746651766	9.97295958635307	29.17123995188748

H	-12.97271611253686	10.68995103006125	29.13004136970590
H	-13.52455149982903	10.76009297809727	27.63190787174662
H	-13.86859937405982	6.76759084901701	29.84809553924783
H	-10.10090003796333	5.78023479973857	25.01076811186750
H	-11.88964549508287	9.87245289746456	34.32168446087729
H	-7.58886052050059	12.95746112713098	33.67107105035049
Zr	10.23657416853359	7.66519761600236	30.27252885790898
O	12.34952125282712	8.07176229945497	30.65100862520405
O	8.82217872580357	5.79167366114971	30.69111550902284
O	9.22218220546470	7.32097480403487	28.37023374800233
O	8.09202934343299	8.40625599767978	30.44645386044770
C	13.08020250130536	9.03646242789734	31.03517113240345
O	12.87219497791398	10.26445690022748	30.82411327314277
Zr	8.17455149133977	10.65031182985195	30.40509245642039
O	6.92503072832348	10.14426710787603	31.96265131069391
O	10.18429941185659	9.88986096002255	30.91006036108917
Zr	7.30432235470398	7.88724178436560	28.32176648677596
O	5.31003418333023	8.85650340739511	28.88157347832530
O	7.96715467706573	9.87851424148715	28.49365620773888
Zr	11.47786626195866	11.23571391483677	29.37996365998413
O	13.25231888729215	10.33893686576461	28.20160026085968
O	10.14561323236554	7.51002896393071	32.25559012163542
O	10.70325770278464	9.21810718684724	28.58318581220275
O	5.08695064676796	8.27612941438444	31.31536615553527
O	6.51584439199611	6.48819733257362	29.69884544176909
O	12.47441589952417	12.91394025754719	29.02050563518899
Zr	10.65673002967000	7.74195213898040	26.77402553904611
Zr	8.90304529665972	11.35954378498680	27.11348156512549
O	7.16943230744527	6.08953989262577	27.03111498258413
O	8.92385222206685	12.19216294910058	31.81874031862477
O	11.27762015608991	5.86699090603808	29.59103549299545
O	11.10747068579421	12.48179637253360	31.25906106765134
O	9.45872425724365	11.79383774084390	29.12165410193714
O	9.54393700751942	9.48318193137228	26.15295043444585

O	11.08181793541202	11.48477665035206	27.19769752699319
C	11.46444399893717	5.21779384414048	28.50806597065576
O	11.23987821868794	5.63252310787704	27.34162084898820
O	9.35744244078829	14.50989689276590	28.46520583089635
O	9.07525852789990	12.96580741547472	25.98320627484636
O	8.99709871838363	6.49740587463039	25.75408511566604
C	7.96801152252537	5.83982762390574	26.07024512091958
O	11.29400846214654	7.50554620343316	24.90994390765434
O	12.61523685524008	8.06160073303993	27.26909123657478
C	10.06630968790744	12.74818141115428	31.92163167517499
H	11.34626888729498	12.38742536602279	26.93993796065049
H	7.71950378006717	8.11790846759963	31.29979428194914
H	9.13445329033261	9.34644210911495	25.28002503180945
H	10.41394979487502	9.93861880747442	31.85744843530926
H	9.14765218034822	13.79992920126077	26.50073533187313
H	10.75200701504015	6.99991485734365	24.28036778328068
H	6.62482302603987	10.80701477779675	32.60732297232421
H	9.60032075953121	6.80649585687545	32.64714332932796
H	6.00146645818903	5.76009433215266	29.30534985310459
H	9.17253946284469	5.03074182520041	30.19498485931710
H	7.88128721520409	5.95931351895696	30.32158682473831
H	5.12949622905469	9.78389471838084	28.65074172327808
H	5.07134964679889	8.70857107939037	29.89182464285746
H	5.58590699732874	7.46032231045310	31.05831061844997
H	5.75954083801467	8.92514416854446	31.68560788603980
H	12.51631991298278	13.58287768983537	29.72514020876543
H	9.42589023987838	13.61044399088922	28.87265667377067
H	8.41831974313535	14.73218806913273	28.57901080606049
H	13.27912384894037	7.61747318559858	26.71367850141582
H	9.72568396753695	9.38446197796039	28.50303710768387
H	13.05161846006094	9.37563041899803	27.88720322474896
H	13.14303419204889	10.84344729117104	27.37122246699418
H	13.99314658538243	8.76905392323201	31.61219619733034
H	10.15229332351706	13.54914407319513	32.69123746735355

H	11.86412301495379	4.18502126015415	28.61991237705170
H	7.71695818316697	4.94998916865476	25.44826715869977

6-P

Rh	6.10967863380396	8.37171514310598	15.17215250041814
N	6.31741631100832	8.27198770376594	13.14636126157091
N	8.03722127581462	7.76156594364030	15.40146630231006
N	5.89845380009221	8.46611418995077	17.19966624036636
N	4.20587138237709	9.05358571651964	14.94152076429213
C	5.31180970600283	8.44576700791280	12.20489223664552
C	5.79464904191915	8.05619665160899	10.89463377519228
H	5.20054668589302	8.06648162431323	9.97952162079822
C	7.10385015397143	7.67169108173343	11.04682098903795
H	7.78889869130102	7.30479823616951	10.28090985413603
C	7.42762113662466	7.81263740143952	12.45291184057392
C	8.67724800294724	7.49030563738831	13.01265564771100
C	8.95136649844301	7.49241140768732	14.39171505034538
C	10.23642057976665	7.15593261567736	14.97294870071688
H	11.14410363926214	6.94468082087968	14.40539513157153
C	10.07721962634893	7.16903184350394	16.33645018004468
H	10.82909311658814	6.97261124080800	17.10254811820882
C	8.69675415251593	7.52587815683897	16.59962530895937
C	8.12743725573334	7.59852872252015	17.88391333177944
C	6.81224668822282	8.02193260577569	18.14572564397601
C	6.20514752865389	8.04301108173045	19.46201537483898
H	6.69410196921697	7.71996182117442	20.38240952638047
C	4.92740346621919	8.52102197686806	19.30781024811899
H	4.16703408544536	8.66513118087118	20.07705218685989
C	4.74150084876354	8.78859078955917	17.89517950600383
C	3.54581665667659	9.26787911449919	17.33042837001609
C	3.32297163045938	9.41412787523010	15.94979146738065
C	2.09695215702410	9.92441527463668	15.36814292454440
H	1.25236142285624	10.31890926262149	15.93534071072467
C	2.22588686277167	9.82263995672822	14.00535069536475

H	1.50731438150127	10.11754668171111	13.23903237944583
C	3.53297744159720	9.25165007989480	13.74433206183422
C	4.01601962818132	8.92825272367486	12.46347317721341
C	9.76448756053761	7.06698053188019	12.08172020125149
C	10.29510965971565	7.97536748125781	11.13853450040640
H	9.90942136196078	9.00154198460862	11.10886357800548
C	11.31036699363798	7.57979324592462	10.25851371700532
H	11.72440738553343	8.28946616388395	9.53468423911023
C	11.81071973772150	6.26103321679272	10.30352034201315
C	11.28185369559323	5.34844963491853	11.24031077904142
H	11.67621302589965	4.32577234823922	11.25712139021057
C	10.27059334702219	5.74752187793379	12.12140677652073
H	9.85262731285671	5.03434053389117	12.84159379042869
C	8.96395877170531	7.15619664438132	19.03823586934779
C	9.36084144053561	8.06648472870199	20.04283576389961
H	9.05997883134333	9.11800842083820	19.96218584867374
C	10.14098006445168	7.63969953550790	21.12530014656166
H	10.45280610691826	8.35001972786293	21.89808758569841
C	10.53552225104275	6.28825146951534	21.22331030565652
C	10.14058306406586	5.37471979578630	20.22342722248532
H	10.45055726401706	4.32746701738764	20.31723375898423
C	9.36467217638414	5.80436629308119	19.14142208917556
H	9.04815009086850	5.09280151707739	18.36943663966342
C	2.41971679669981	9.58939086398979	18.25620566992913
C	2.51769606913002	10.66458495014099	19.16705398994857
H	3.42871451534869	11.27506251093913	19.17960342888129
C	1.46169686638419	10.96022081924977	20.03873312785416
H	1.53898449796123	11.79923193740463	20.73811325893407
C	0.28660146112977	10.17912112022191	20.01650359066146
C	0.18509982588396	9.10188680914403	19.11123823485282
H	-0.73151507495009	8.50066900172138	19.11190148370143
C	1.24010464246231	8.81098116899727	18.23945571741334
H	1.16647213723146	7.96537232657785	17.54537807806899
C	3.08263542352586	9.08822446151714	11.30977097515877

C	3.34933203781603	10.02474784756577	10.28538624576179
H	4.25145015923097	10.64508204749543	10.34765223841267
C	2.46563268624375	10.17184151131972	9.21035668774151
H	2.65875538102745	10.90114045420636	8.41510818942955
C	1.29862009249341	9.38301556564425	9.13495895330106
C	1.02771819260675	8.44325389209447	10.15238605078972
H	0.12500238681518	7.82658261701166	10.09089713164178
C	1.91189488184533	8.30075629574003	11.22907496184005
H	1.70690408276424	7.56554069890671	12.01644081758929
C	0.39102605630569	9.58447325248672	7.96572063202709
O	0.59175587221936	10.38229592292926	7.06093182882389
O	-0.69610817927150	8.77743328899814	8.01526726519408
C	11.36491775963530	5.77429793526344	22.35432570505904
O	11.73048769759091	4.61367305591726	22.47560066539600
O	11.67396991868430	6.74613400144516	23.24663022806366
C	12.89122099287239	5.78168648949320	9.39033775834591
O	13.35468629784711	4.65011908314638	9.39729721095034
O	13.31125352091517	6.75011079378085	8.54066275069122
C	-0.87106034478052	10.44484578532105	20.92230704564642
O	-1.90745957968957	9.79589657045811	20.93238732645507
O	-0.64665271408271	11.49676245168553	21.74618625048194
C	-1.71686535960054	11.81938622940794	22.65104984780553
H	-1.36785390899100	12.68130145535514	23.23784501057786
H	-2.63379615114415	12.07872313886436	22.09354960330029
H	-1.93725794689410	10.96515441309985	23.31476767494465
C	-1.62271637970774	8.91154066449254	6.92349008507038
H	-2.43540318333146	8.19945526885032	7.12710162577919
H	-2.01635020656140	9.94158843325198	6.87091062966870
H	-1.13184492197185	8.67210773991359	5.96404931718328
C	14.35659800619629	6.37010343639273	7.62930431063147
H	14.57568858200635	7.26529615421205	7.02956312762565
H	15.25612488566661	6.04612209672198	8.18126045720928
H	14.02503617802236	5.54227165186408	6.97838925388207
C	12.47426147455709	6.33174464844288	24.36735755339934

H	13.44672758972653	5.93558871535647	24.02641705437400
H	12.62087906042506	7.23052849218335	24.98354718363002
H	11.95723598738831	5.54696490207966	24.94661025796421
H	5.65590256970328	6.93859676729712	15.18235620778595
Si	4.59759058793218	4.55465008978579	15.56950400470005
C	5.16258603507138	4.85698857919839	13.78016532364354
H	4.51424346194926	4.24058056583383	13.12532706092232
H	4.95806570508772	5.90538158188087	13.49194732520243
C	6.04069847115919	4.63652578773676	16.77919035673813
H	6.72950827682855	3.78514210807024	16.63481806310851
H	6.61826366425934	5.56724187927709	16.65100967788706
H	5.67236572507070	4.61144858089533	17.82110526954656
C	3.25531627675579	5.78450404278922	16.09224761322815
H	2.75409045621077	5.37737837285017	16.99339601297707
H	3.75988605102639	6.71035419904293	16.42201341027384
C	6.64420366180287	4.53290474472906	13.52301563895489
H	6.92525004422645	4.70392757639253	12.46707786665027
H	7.30293136128436	5.16855330126036	14.14111968475792
H	6.87698553084465	3.48011937741243	13.76842520554942
C	2.22717673907529	6.11827736067608	14.99981096559958
H	1.67223361266720	5.22158427675240	14.66581290409229
H	1.48272429696841	6.86045704479579	15.34390338995446
H	2.71892955207583	6.55151993468902	14.11016817248328
C	3.85634204958186	2.78164412597709	15.65005774549854
H	3.57203553048970	2.61451128494502	16.70872464114537
H	2.92494236915419	2.81053644091484	15.04896281392731
C	4.81034957161661	1.76649970963587	15.15446527306667
H	5.57480587491183	1.34048501529418	15.81512072756792
H	4.89087528673866	1.54314143400362	14.08406527337996

6-P (dimer model)

O	6.64331998532292	8.55736588269933	12.11972596889896
O	-6.64331999316239	8.55736596895456	12.11972598681922
O	6.64331988427622	12.11972608742188	29.95463399240335

O	6.64332000512041	26.39227399578020	29.95463398823460
O	6.64332019111676	8.55736597971832	26.39227386632682
N	2.02103763249779	19.08646464690806	28.95335984644567
C	6.42803965712692	12.72777408112194	28.84584329797272
C	5.27004234442753	13.64465003731118	28.80244824892933
C	4.44832529904920	13.77684556252745	29.93799287228091
H	4.65643883143606	13.16194189360874	30.81990428020538
C	3.41613746067139	14.71383063525758	29.92759863992960
H	2.78159221701911	14.86495838539861	30.80879113507749
C	3.22319932191077	15.52395994992924	28.79045307804732
C	2.24292813789101	16.62837768590964	28.85630457009872
C	2.76794073097192	17.92489866288306	28.91076021983118
C	4.18364731297650	18.24203322088625	28.91876956519106
H	4.99189620232473	17.50959011834104	28.92567373600771
C	6.50150780374130	25.68440888485008	28.97466153195851
C	5.59389036064686	24.51740008860978	28.92830481180869
C	4.86688926201268	24.24665692713508	30.10291730423535
H	5.04870187571725	24.86892589784794	30.98607603562791
C	3.92068997092027	23.22541167469429	30.10300485198917
H	3.32307968469469	23.00981919470051	30.99651589063160
C	3.70373434487979	22.47567964213671	28.92919229582138
C	2.60382301662226	21.48616908241265	28.92488104535471
C	2.93040613359226	20.12821408369416	28.93718972436653
C	4.28370434634984	19.61063437102879	28.93438992739161
H	5.18690437369248	20.22297138526415	28.95437301122803
O	7.13627424292156	12.61267987955530	27.81336590903543
N	-0.15022935096970	17.20990704943560	29.03818228561146
C	5.04072248295388	14.40600068494330	27.64007651538329
H	5.69268460980214	14.25729655203762	26.77220904889216
C	4.01499010483724	15.35120553287503	27.63695120993377
H	3.82980865419424	15.98094028059297	26.75906232377927
C	0.88419307183129	16.30455816277484	28.89160100030024
C	0.35820781069006	14.95949602871683	28.77141533487211
H	0.96257322026345	14.06179778083503	28.62981127441589

O	7.13627399729915	25.89931999623724	27.81336600994378
N	0.14406804836436	21.26246674041327	29.04084697272406
C	5.41445519540454	23.74312512074135	27.76631448727582
H	5.99755556070856	23.97080999726458	26.86806668774606
C	4.46695144315965	22.71507636371198	27.77049146327192
H	4.28231696112099	22.11574313489655	26.87095539780629
C	1.30442733154124	22.00708621322451	28.94303140993885
C	0.98494264254360	23.41915478950298	28.84826738105118
H	1.71673389092192	24.22199052105536	28.74772386883249
O	-7.13627393205882	12.61267984272758	27.81336595402575
C	-6.53400274838450	12.82753700076437	28.89678073592003
C	-5.60720585895505	13.97678768031750	28.98490062019190
C	-5.42210165309813	14.81473838311421	27.86913956764639
H	-6.00578205980427	14.62732775712369	26.96090205735933
C	-4.47630423845825	15.83906943029693	27.93697208851141
H	-4.28563969396127	16.49017655698259	27.07573650965443
C	-3.71524851888917	16.00690405987866	29.11123281063808
C	-2.60903688371711	16.98881207099118	29.13312352603780
C	-1.31491900784116	16.46704420032486	29.02601584019362
C	-1.00809778442727	15.05958630510038	28.85581468504091
H	-1.74730668920558	14.25928360946821	28.79641609217594
O	-6.64332004225329	12.11972612477142	29.95463403238239
N	-2.02209760894118	19.38617817530997	29.17014038568029
C	-4.88781322885183	14.17776064322475	30.17902265779221
H	-5.07631989414617	13.51328732667566	31.02861473680290
C	-3.93626683194601	15.19411613961640	30.24015181660913
H	-3.33849626770002	15.35602882333891	31.14481538341930
C	-2.93131996487132	18.34482761423572	29.21424683340220
C	-4.28044758683005	18.86193698917812	29.31969030382078
H	-5.17959434848052	18.24885999980242	29.40363174636629
O	-7.13627399201281	25.89932000461465	27.81336603445708
C	-6.43036920680830	25.75449717415167	28.79438164502741
C	-5.27113911788696	24.83927838798212	28.86986066857886
C	-5.03762201906364	24.00558571298996	27.76046308076798

H	-5.68113248219333	24.11028596022889	26.87975573693181
C	-4.02503553931754	23.05008655759516	27.82499276699503
H	-3.84097466405129	22.36235991411202	26.99144539286710
C	-3.24325667202065	22.94012103967132	28.99318800376413
C	-2.25413499688140	21.84515812882851	29.08536337681103
C	-0.89757066155047	22.17168344461317	29.01813435725220
C	-0.38300975699598	23.51974129015380	28.88971694991462
H	-0.99680328848046	24.41993513654349	28.82935909574911
O	-6.64332000980866	26.39227399348999	29.95463397122287
C	-4.46565319977112	24.76187899227155	30.02235057009150
H	-4.66616466348901	25.42683240610640	30.86817020974043
C	-3.44280095134415	23.81322734104772	30.07912745604445
H	-2.81811467332345	23.71105006892495	30.97439000132064
C	-2.77034124916493	20.54761787784319	29.18725400791111
C	-4.18148385067459	20.23070290140482	29.30116783076626
H	-4.98505724589156	20.96538307768784	29.37044654731626
O	7.13627367701940	10.69863405522650	25.89932022732864
O	-6.64331999643007	8.55736597114140	26.39227388251014
O	-7.13627400997179	10.69863403314297	12.61268000559182
Rh	0.00242935859185	9.65794170037342	19.26309971922007
N	2.02563321505355	9.57366734289168	19.42948699578704
C	6.54738659561323	9.66506916171860	12.87238241207139
C	5.62877299859694	9.49977028368080	14.01810652595630
C	4.85170398461690	8.33849047141981	14.19173188995440
H	4.97834787393659	7.49459863213684	13.50595167996549
C	3.90088340513074	8.30107596721046	15.21420434997852
H	3.25505754821937	7.42542826044164	15.34931797928550
C	3.73782785427356	9.41446916234889	16.06225366862842
C	2.62530743713875	9.45553146077441	17.03687743604133
C	2.93915938246724	9.44588946041747	18.39975888970863
C	4.28309419796330	9.33381048132833	18.93319965719931
H	5.18551286216278	9.19611113152649	18.33471242117983
C	6.42171284290603	9.66635075693930	25.78993541313065
C	5.26516695195112	9.71286875366452	24.87446836020771

C	4.42687696190571	8.58756481514366	24.74993047891011
H	4.62582560449795	7.70471813959994	25.36695528861928
C	3.39199596163599	8.60731711914038	23.81478512476915
H	2.74406640005540	7.73470413144849	23.67101650957798
C	3.21305170616797	9.74053918001277	22.99540398690028
C	2.23194149353588	9.70069062972624	21.88895738600617
C	2.76357883032039	9.58514971840626	20.59740578821636
C	4.17590620900766	9.42837731810955	20.29829063019551
H	4.97475164856558	9.37922237877287	21.04005781797423
O	7.13627403140485	10.69863411381051	12.61268002444056
N	0.16908412970919	9.65827697888501	17.23861898274649
C	5.50488202443654	10.59409797232278	14.89559442013685
H	6.12996414839244	11.47742481403278	14.72340523349838
C	4.56632969141148	10.54703710019265	15.92423423419993
H	4.42536682223351	11.39989751164403	16.59857833094499
C	1.33653857461028	9.59203548616051	16.50188761227296
C	1.04093331592664	9.72137258535712	15.08592812860786
H	1.78391212694961	9.73166257062408	14.28697263157521
N	-0.16211166356163	9.67238862873895	21.28903463091640
C	5.05042386613870	10.87225843340696	24.10350888003929
H	5.71535939095140	11.73166694160827	24.24192907674214
C	4.02525844583422	10.88171489737085	23.15913856543982
H	3.85673503226127	11.75476057884885	22.51787379858065
C	0.87100169069853	9.73814317371862	22.20571444092971
C	0.34141693003371	9.82836315853373	23.55214697460930
H	0.94378126139099	9.91913537716135	24.45806033097781
C	-6.38341474124748	9.59079457094874	12.70903624553250
C	-5.23807707714955	9.75974083144646	13.62802587948734
C	-5.05035156464333	10.93649586603950	14.37841172896778
H	-5.72490460310706	11.78598862032800	14.23097452830976
C	-4.02749299549853	10.98440200173645	15.32812488527047
H	-3.88089596218062	11.87476845473169	15.95092325431305
C	-3.19340445433178	9.86473659068544	15.52008061834036
C	-2.22019516515620	9.83879700229194	16.63436416389315

C	-0.85724101396713	9.79141699711712	16.32165760810793
C	-0.32076948072476	9.85080952088339	14.97549744390536
H	-0.91466395470392	9.98293926756200	14.06926978989688
N	-2.02251322949032	9.72742718070624	19.09682695500061
C	-4.37387035865535	8.65826455864851	13.77902422780408
H	-4.55534583504178	7.76272094804051	13.17419474161573
C	-3.34624475470175	8.71438750301689	14.71853578629889
H	-2.68402257444690	7.85585223397447	14.88089121895341
C	-2.75831553633515	9.77114586947951	17.92778556920402
C	-4.17680585477575	9.68888964410618	18.22831399100418
H	-4.97700642927670	9.67349887331574	17.48683452491631
O	-7.13627402819988	10.69863406147319	25.89932013275097
C	-6.52662721028881	9.62031811074108	25.67864483726507
C	-5.60005746718308	9.56987774900199	24.53196628156800
C	-5.49743528889113	10.69558755230543	23.68963094204572
H	-6.13317838366847	11.56544695135256	23.88907538929142
C	-4.56616995844896	10.68956763982690	22.65194403266799
H	-4.43925382389609	11.56392935052631	22.00292889094905
C	-3.72955361586911	9.56884585259385	22.47057243969055
C	-2.62491578643953	9.62229335081338	21.48957900407395
C	-1.33009642342021	9.67726695362067	22.02577565898116
C	-1.02628708037726	9.78281932624197	23.44227349293923
H	-1.76594945565273	9.83578927815774	24.24261820741262
C	-4.81505709763725	8.42144643641299	24.31369984508850
H	-4.94054253854969	7.55698080927595	24.97427174884454
C	-3.87544816243082	8.42528231313960	23.28379818716816
H	-3.22616555845263	7.55937857786587	23.10963805462847
C	-2.94149148691147	9.64962442321941	20.12736299505734
C	-4.28923376812144	9.60699266588312	19.59368043440419
H	-5.19867932410386	9.51568466062658	20.19037195012751
C	-7.76382854155711	27.29453936731707	29.98261898679076
H	-7.64021469504217	28.09174522261559	29.22918005082480
H	-8.70271300043436	26.75319774636891	29.77311646891115
H	-7.78361311825077	27.72176482024708	30.99541667171031

C	8.02337629137603	27.03185457560344	27.77946887547858
H	8.45236266870689	27.05017958919755	26.76740930479561
H	7.46911852576137	27.96482594362846	27.98155185822586
H	8.82037655541167	26.92357147529614	28.53535668923372
C	-8.28899738587829	10.60453646691466	11.75837910178208
H	-7.98830437470462	10.36605927773651	10.72328607507904
H	-8.97525699874947	9.81709633509676	12.11590230775447
H	-8.77808311669406	11.58841756390975	11.79967556331600
C	7.49556504948805	8.65188918053378	10.96546725131242
H	7.45413292303711	7.66787618511702	10.47667260361131
H	7.13634221669457	9.43898894429343	10.27969605756354
H	8.53095902166379	8.89110508341910	11.26447218759624
Rh	-0.00305033789314	19.23510678913008	29.02695774062330
H	-0.10112456018660	19.19795834867683	27.52801140042558
Si	-0.54996907676518	19.48811348999632	25.04429584297833
C	1.29340724690590	19.65515178287484	25.44550024079046
H	1.73008093127200	20.30848171584369	24.66329726129869
H	1.40678709742973	20.21389679008021	26.39285101927566
C	-1.35024503607389	17.88486838581416	25.62822036642137
H	-2.05158543580084	17.49950875952833	24.86605258519332
H	-0.60966042581798	17.09445353579325	25.83995317982754
H	-1.92335400753590	18.05571983206430	26.55572390419920
C	-1.53839665719375	20.98219307332693	25.63540403560834
H	-2.50545779719280	20.99127428043217	25.09383730065997
H	-1.78806860617696	20.78960623024982	26.69563401067335
C	2.05983391815687	18.32623114328709	25.52703767565039
H	3.13361273378983	18.48740645492649	25.73610111927172
H	1.66449173478492	17.68580991095252	26.33403619000711
H	1.98345743559948	17.75114953488098	24.58529318046590
C	-0.81735309811963	22.33157899992656	25.50056630012260
H	-0.55500480585303	22.55697264137003	24.45005679617542
H	-1.44202869189044	23.16472752341113	25.87177613751350
H	0.11855297807366	22.34696500684017	26.08724869644635
C	-0.67583311796352	19.49913239249319	23.08154441354016

H	-1.76427237922352	19.40990485189234	22.90232659189827
H	-0.30889926607174	20.50062786371870	22.78990648324585
C	0.10277209103172	18.40317511685245	22.54960205957898
H	-0.31625373293172	17.39274153342404	22.47411491894430
H	1.16381528556911	18.52552242488623	22.30301946198567
Zr	-10.13039819947925	8.20466169468935	30.90662826897105
O	-12.25090016646979	7.82171227102202	30.53037338110833
O	-8.67711502072371	7.81537885541692	32.75800202091663
O	-9.13034078279069	10.12865473401199	31.23575187712902
O	-7.99070255023525	8.04572907604036	30.13550799072064
C	-12.96867895251982	7.36952956057280	29.58228431947172
O	-12.76633270700995	7.53170001636849	28.34732439083211
Zr	-8.11363165948007	8.08083341069345	27.89126720924207
O	-6.82258017217191	6.54997189607240	28.37148053165175
O	-10.10164444885396	7.56002090324972	28.68978990621368
Zr	-7.22344412301265	10.17164779082654	30.63088690408143
O	-5.27040063410026	9.66671121375937	29.56426941646511
O	-7.96779817899956	10.00159637848565	28.67162212002862
Zr	-11.43729601404023	9.06117435369552	27.37255903945416
O	-13.24097733066985	10.12917489443206	28.32694466704899
O	-10.02781969773566	6.22346385134012	31.06809189050252
O	-10.65917493026888	9.87203686303070	29.38378930940680
O	-4.93857692489217	7.25524208217338	30.15643983219644
O	-6.37107944272204	8.79812767223189	31.99718838501371
O	-12.54206184287501	9.39907575474177	25.75474746408590
Zr	-10.54881253897833	11.73716722737912	30.82288411336187
Zr	-8.89418817852957	11.35764782184244	27.16950612037914
O	-7.03123896471424	11.43800682087457	32.44640017044414
O	-8.88199344250709	6.64351470474628	26.38250392125006
O	-11.15281524257912	8.85949503510255	32.72928274434147
O	-11.06514360342042	7.20587765124208	26.09800298911312
O	-9.42949523324585	9.34089145973372	26.75407705065922
O	-9.51146708512224	12.33949093935688	29.04315858446262
O	-11.06932509165620	11.23215069687614	27.10159922167673

C	-11.41949389133042	9.95372169687319	33.32664209766768
O	-11.24988323916299	11.11336429788776	32.86721157789990
O	-9.46006671981102	10.01820049574307	24.04209653220233
O	-9.11196308894882	12.49462526181427	25.57348140258620
O	-8.90111105590662	12.68976712904355	32.16183604278910
C	-7.84022268270136	12.37021503031299	32.76309662902645
O	-11.04966568572302	13.65409895076055	31.04149180975714
O	-12.51566063229779	11.45624048649772	30.36169622724860
C	-10.02507311681238	6.54306211670717	25.82754629045171
H	-11.39619477702322	11.49141377785692	26.22090878238656
H	-7.59440750430467	7.20554057305687	30.43109987902152
H	-9.46812396702459	13.31262084296327	29.02143523518330
H	-10.32520733709258	6.61111649332788	28.64289090921450
H	-9.17116642220119	11.97984488810580	24.73657149477127
H	-10.45099474800817	14.19155572955021	31.58949195887685
H	-6.52164770908211	5.91891321378767	27.69573152973618
H	-9.46930724543518	5.83088428717304	31.76043872272503
H	-5.85163637453802	9.20218273326094	32.71590666976747
H	-9.01559456531580	8.33050177219896	33.51202251604203
H	-7.73926326889146	8.17593588742259	32.56807802644814
H	-5.17618912336717	9.88641494157151	28.62074112537107
H	-4.98932490102722	8.66337086251238	29.70983876435171
H	-5.42101506033639	7.49389891349180	30.98811395952205
H	-5.62085532395055	6.85709577871120	29.53288434615214
H	-12.60242356980091	8.68964722292540	25.09201309117904
H	-9.47093357617536	9.59290791313679	24.93543469713103
H	-8.59646167511571	9.76551591641945	23.67474823413090
H	-13.13643856378163	12.12338939333046	30.70192269312362
H	-9.68735565446337	9.97051868749292	29.18200165661559
H	-12.96878663791815	10.67230217586297	29.16448537636366
H	-13.48485665462085	10.79810619624941	27.66219276281327
H	-13.86138889737772	6.77464831108769	29.87825806385589
H	-10.11271567968014	5.77665710716915	25.02393677210522
H	-11.84000784651012	9.86235682963873	34.35281008561939

H	-7.56684666371422	12.96289889636738	33.66695059613144
Zr	10.23976596077393	7.66809273164461	30.27285457438634
O	12.35106285725550	8.08956467478426	30.64927983566059
O	8.83359332992675	5.78861569553017	30.68473213079887
O	9.22268082468834	7.32043376346432	28.36435956393870
O	8.09233605550632	8.40328109313807	30.44638450539097
C	13.06272230623579	9.05242289007737	31.07587431589469
O	12.84623325257757	10.28228333733208	30.88992711954179
Zr	8.16758287216521	10.65073619980645	30.40799125931626
O	6.92001568110662	10.13867319993825	31.96536246559758
O	10.17610415224387	9.89022181392548	30.91530868969685
Zr	7.30503479775455	7.88683475058862	28.32607167740585
O	5.31056705738847	8.85539620942447	28.88539859722582
O	7.96871442088458	9.87612791225313	28.49463075609355
Zr	11.47378686901957	11.22902326416083	29.39242991704806
O	13.27633329654215	10.31131483160105	28.28163516626412
O	10.15070566096898	7.51010050359400	32.25575808627224
O	10.70572283418875	9.21186790595351	28.59098248140684
O	5.08519124946451	8.27208338628500	31.31607198655595
O	6.52096990877752	6.48548766493496	29.70508385933545
O	12.50133195647891	12.88861649602933	29.02616561141030
Zr	10.64479446139403	7.74703549156436	26.75824098566684
Zr	8.90654144925714	11.36237763888168	27.12061991851474
O	7.15395608226759	6.08654444528151	27.03992405003824
O	8.91596804432158	12.18923962528100	31.82357716475265
O	11.29290728045567	5.87841504549447	29.58967994581104
O	11.09436037646098	12.49633380219502	31.25355920535174
O	9.45550728200249	11.79359473627038	29.12842946547599
O	9.53734256024933	9.48767703841956	26.14858364440854
O	11.08159737089097	11.46655767998354	27.21684066065408
C	11.49740018241875	5.23848791629593	28.50486357251499
O	11.27432347806727	5.65698173496484	27.33943335692553
O	9.36967097059142	14.50879675781484	28.44771278938975
O	9.08462307468602	12.96061655848986	25.98141462236764

O	8.98656405234567	6.46961694837733	25.76185500901838
C	7.95283115279136	5.82258276773829	26.08313981584740
O	11.24164042122663	7.49679538219111	24.88047581536555
O	12.60617575790869	8.12338883283972	27.18496341811823
C	10.05328342507960	12.75711224626197	31.91849825544596
H	11.37528707069109	12.35312125194556	26.93786220672472
H	7.72002847724304	8.11317440977611	31.29924039551289
H	9.15187859095644	9.37019420508596	25.26219399622981
H	10.40534570348207	9.94022396381234	31.86269560960146
H	9.15935230160632	13.79488593931720	26.49929745365969
H	10.68299498834015	6.96614582407453	24.28664741901650
H	6.62179423501651	10.79874131209412	32.61380495713409
H	9.61102849014642	6.80207391307129	32.64695735162953
H	6.00976546848645	5.75656865308308	29.30897812171213
H	9.18340276196248	5.03256199763176	30.18074948233993
H	7.89043430620340	5.95686964158714	30.32107325883699
H	5.13206803537624	9.78413513229190	28.65796220492472
H	5.07337020912866	8.70578653697715	29.89705552551941
H	5.58466217640876	7.45543508535639	31.06299814560438
H	5.75657314574041	8.92125257321403	31.68930627486258
H	12.55743378270060	13.55598586905863	29.73146362295657
H	9.44357390668548	13.61522786472225	28.86615906571748
H	8.42865101934714	14.72577661741339	28.55723101258297
H	13.26350098585075	7.70994290796319	26.59899386089637
H	9.72866306426573	9.38117278370079	28.50780977793676
H	13.04025869771629	9.39479361507802	27.86383097962768
H	13.35145340422853	10.89762704620556	27.50596427631798
H	13.96379628626769	8.77874213778728	31.66857395813950
H	10.13431288396315	13.56397591574092	32.68231712022623
H	11.91219448624299	4.21141351609084	28.61341707750406
H	7.69715346748645	4.92782837199663	25.46979439387320

13-P (dimer model)

O	6.64331997752741	8.55736587561710	12.11972596695819

O	-6.64331999677109	8.55736597979291	12.11972599130840
O	6.64331991859188	12.11972597895924	29.95463395311418
O	6.64332000009550	26.39227399920187	29.95463399953710
O	6.64332009642590	8.55736599322544	26.39227401559848
N	2.03020886112587	19.07982939505348	28.77630187627003
C	6.42379584023893	12.72300006367322	28.84359171225114
C	5.25860779861634	13.63028119567288	28.78157596577715
C	4.46832429864880	13.83748659689803	29.92790324809836
H	4.70080423655413	13.28223330536075	30.84291261208323
C	3.43791406992169	14.77633005835662	29.88452005991480
H	2.83223675871094	14.98896014423721	30.77341050572218
C	3.20776764846618	15.50867743936020	28.70138140951881
C	2.23191692716172	16.61941010519520	28.72907876158023
C	2.76086554194727	17.91735648679909	28.67670206521246
C	4.17589274314480	18.23102322543750	28.58317554876152
H	4.98022409827468	17.49789488415422	28.51133985735764
C	6.50599892858559	25.68062594991093	28.97658153172704
C	5.60923153146195	24.50541324444143	28.92932296987833
C	4.92213441304040	24.18967595075552	30.11658863172341
H	5.12858057443184	24.78220969196672	31.01477956854911
C	3.98120049722439	23.16291202390439	30.11166965513076
H	3.41600792904753	22.91246322193550	31.01709861394986
C	3.72270024538406	22.45700383789028	28.91859808114065
C	2.61773273500950	21.47428960427220	28.90132231504912
C	2.93472277975568	20.11888262122167	28.75514313902708
C	4.28303478357458	19.59851924430520	28.63106595327256
H	5.18953858001696	20.20552073648572	28.60620269735835
O	7.13627415851174	12.61267997977371	27.81336598701752
N	-0.15604118220735	17.20488031896491	28.98201832951857
C	4.99228865378880	14.31266341724374	27.57813732084375
H	5.61868331282685	14.10637600783046	26.70343523167341
C	3.96270258965968	15.25226847923664	27.53878908892359
H	3.74770894400646	15.81493942610114	26.62338427329842
C	0.87589090462299	16.29930637680366	28.85709851601261

C	0.34887933841852	14.94765258803490	28.82623882082249
H	0.95009402996685	14.04401259302496	28.71172363592592
O	7.13627400186727	25.89931999772752	27.81336599813492
N	0.15810062222228	21.26557623207799	29.07712367734154
C	5.39490563159050	23.76997687577704	27.74792733923996
H	5.94628568433384	24.03243762060481	26.83929017310454
C	4.44820353659621	22.74224684174922	27.74515941487008
H	4.23104316002656	22.18093391019463	26.82852436417883
C	1.32342898340090	21.99995794912070	29.03175724113843
C	1.01404756023013	23.41968964308888	29.04146380194672
H	1.74997367448542	24.22347656373269	28.99568205282356
O	-7.13627391199565	12.61267987242540	27.81336593403064
C	-6.53517676622395	12.82838650855535	28.89715795437581
C	-5.61213108184530	13.98046109256767	28.98050696650102
C	-5.41872346409057	14.79969858991336	27.85213206111302
H	-5.99408255576123	14.59525681286993	26.94234222561416
C	-4.47360388704122	15.82430947294927	27.90894584650334
H	-4.27292206867051	16.45650311238152	27.03598844349552
C	-3.72286870601542	16.01588694559446	29.08684594541145
C	-2.61684308413425	16.99740877629714	29.09172000053552
C	-1.32038440380426	16.46917552316380	29.03111060723684
C	-1.01520403711750	15.05239609032913	28.93291062950649
H	-1.75424585223945	14.24994398535730	28.92231361434020
O	-6.64332004814728	12.11972605985598	29.95463402684172
N	-2.03813569515277	19.39477794832199	29.06062222883540
C	-4.90310967951885	14.20303287689650	30.17675808296489
H	-5.09706649377992	13.55226031684555	31.03584841660535
C	-3.95497073743802	15.22364492361454	30.22847567629370
H	-3.36703210639769	15.40332279868056	31.13626823893962
C	-2.94322100828782	18.35722332862147	29.08486127059015
C	-4.29585558996208	18.88011590327737	29.11777472052245
H	-5.20115474397744	18.27212486251741	29.16182916959188
O	-7.13627400228405	25.89931999686614	27.81336599625516
C	-6.42655427750248	25.75834298182913	28.79260068311828

C	-5.25688407258419	24.85529109129504	28.86499091645607
C	-4.96792779908314	24.08085933389488	27.72604025144138
H	-5.57559068796811	24.22364189083416	26.82535826903982
C	-3.94741244508555	23.13293486035923	27.78394428086346
H	-3.71832909911601	22.49630919752773	26.92131304575032
C	-3.21899467622739	22.96174534947187	28.97955066633871
C	-2.24014583304254	21.85642918543563	29.06352148501347
C	-0.87857898322762	22.17545653190593	29.09257418340149
C	-0.35324914813081	23.52704220429695	29.07015927613602
H	-0.96000266697424	24.43394291600248	29.05674394728003
O	-6.64331999966355	26.39227400519562	29.95463400678555
C	-4.49696592255947	24.72594555751911	30.04391980781013
H	-4.73796353422255	25.34588657330890	30.91302281679606
C	-3.47279480949276	23.77847263829645	30.09794307887711
H	-2.89075813129883	23.63130642163075	31.01550334486414
C	-2.77202345157278	20.55883786949911	29.06013988284508
C	-4.19054734837702	20.24866796212572	29.09699785045419
H	-4.99405612797642	20.98603430755928	29.12492992170978
O	7.13627382466933	10.69863404253341	25.89932004416727
O	-6.64331998025232	8.55736602789251	26.39227395069078
O	-7.13627400561893	10.69863402295172	12.61268000077446
Rh	0.00243466840868	9.65944868772562	19.26328347244621
N	2.02521990150625	9.57678445984973	19.42976761244223
C	6.54666093292890	9.66536040212451	12.87208551975252
C	5.62870931525965	9.49996385080903	14.01821235626037
C	4.85158376762531	8.33875019707247	14.19221623508714
H	4.97801513490088	7.49473634183907	13.50653262756140
C	3.90103999258465	8.30150796186667	15.21495379564582
H	3.25518099274908	7.42592267385898	15.35030557902318
C	3.73785608169804	9.41504566445222	16.06284715850170
C	2.62529870137001	9.45631755001278	17.03741777601916
C	2.93910395725419	9.44799788489022	18.40027979702730
C	4.28327613131034	9.33796829591168	18.93366824659100
H	5.18583963313263	9.20086093050018	18.33523609536665

C	6.42133089366891	9.66649640000015	25.79009949165208
C	5.26467385612122	9.71351176011573	24.87512744494894
C	4.42455989919694	8.58939616290701	24.75184817087968
H	4.62215449790210	7.70684701091534	25.36974797386985
C	3.38971757368412	8.60977173186490	23.81669014842626
H	2.74044786110964	7.73801480584539	23.67381951337066
C	3.21243680137609	9.74223606908947	22.99580272059675
C	2.23155043830529	9.70261589866538	21.88923710587095
C	2.76337555303338	9.58768344129050	20.59773756726843
C	4.17599659119798	9.43282061804003	20.29872313899813
H	4.97491645657157	9.38431329212529	21.04047392221840
O	7.13627403227885	10.69863412653931	12.61268002247473
N	0.16922292875020	9.65969885312739	17.23929741725498
C	5.50464552346280	10.59455442802638	14.89541772786108
H	6.12950578300842	11.47797127183862	14.72286945354052
C	4.56633517498077	10.54759315898955	15.92427474378497
H	4.42529209729616	11.40062482035932	16.59838304797945
C	1.33663673564207	9.59288621365864	16.50230563990783
C	1.04131370200571	9.72273261304869	15.08626341413563
H	1.78435195310819	9.73299880994461	14.28734936295454
N	-0.16224987051956	9.67407252099357	21.28874411308144
C	5.05156452911027	10.87236380707554	24.10279489830718
H	5.71805472668750	11.73076875208494	24.23990693810061
C	4.02643345505837	10.88235274364634	23.15847055256435
H	3.85935513665288	11.75480579205206	22.51602977457408
C	0.87062094892703	9.74012760310176	22.20586244298972
C	0.34095935465207	9.83051328491097	23.55231009591327
H	0.94316946515315	9.92132154967885	24.45834124843624
C	-6.38326492867858	9.59073858245164	12.70915429105197
C	-5.23791214874152	9.75988494762291	13.62794313750477
C	-5.05023823448053	10.93677804091714	14.37819266770430
H	-5.72497709850884	11.78613203499209	14.23077710518962
C	-4.02735009671263	10.98492239990087	15.32784181829125
H	-3.88086488701567	11.87534993603825	15.95057767182083

C	-3.19301491838148	9.86544737621604	15.52007039739775
C	-2.21984885606104	9.83989546410493	16.63439901523073
C	-0.85691939784726	9.79243383755330	16.32186836111098
C	-0.32046345139349	9.85101216227600	14.97560591511426
H	-0.91428142891016	9.98302804153878	14.06929344064551
N	-2.02202344381958	9.72963427089825	19.09681965343594
C	-4.37328107668758	8.65869806135009	13.77890190028178
H	-4.55456803200536	7.76302603748200	13.17419623812494
C	-3.34568783187651	8.71511254863832	14.71843768655811
H	-2.68317152245677	7.85680380603740	14.88078267506923
C	-2.75807150165414	9.77297084776074	17.92776142166655
C	-4.17671125382549	9.69169299148515	18.22811709521703
H	-4.97688424267357	9.67653958178439	17.48658351484266
O	-7.13627407052679	10.69863404279075	25.89932008739151
C	-6.52640404852615	9.62028014776106	25.67847510375448
C	-5.60000158193454	9.57022295111384	24.53194750040248
C	-5.49837374398199	10.69585574451094	23.68925763315274
H	-6.13471453453081	11.56532031517472	23.88855275468514
C	-4.56730769830198	10.69034058011966	22.65144217255808
H	-4.44127972030016	11.56455318625442	22.00206074221236
C	-3.72952059115033	9.57043404285232	22.47025413642228
C	-2.62495913484151	9.62455964291286	21.48931693678419
C	-1.33025375060112	9.67948076378302	22.02567247490786
C	-1.02670341010325	9.78365592792385	23.44237262203411
H	-1.76644813822428	9.83665477867933	24.24266675264564
C	-4.81391204234636	8.42244493503678	24.31386804926116
H	-4.93852390697898	7.55797399077520	24.97460809182995
C	-3.87437430798044	8.42699622526493	23.28394150298104
H	-3.22424183506270	7.56171519142986	23.10989622517311
C	-2.94144912468771	9.65194076395848	20.12714358597853
C	-4.28927311920981	9.60988982987272	19.59346700285446
H	-5.19883626167278	9.51891952876055	20.19006312666981
C	-7.77664267940256	27.27810339794913	29.98836161793157
H	-7.66865924854627	28.07644865601024	29.23376623685263

H	-8.70884946451783	26.72323013427343	29.78433413013644
H	-7.79704243392379	27.70570416508191	31.00100263702515
C	8.00867405001527	27.04299981430386	27.77384346161471
H	8.43651761068864	27.06262148253859	26.76131990811563
H	7.44277903092810	27.96971621681996	27.97256127459639
H	8.80763205003331	26.94802004126913	28.52943079256965
C	-8.28893165695992	10.60426862118316	11.75844802942477
H	-7.98829827473421	10.36562155636997	10.72336444106483
H	-8.97516548860104	9.81683123233568	12.11606313809761
H	-8.77814249079931	11.58810813873391	11.79955008959611
C	7.49589724440996	8.65197847051736	10.96581233038915
H	7.45508312322418	7.66782929052732	10.47721664331139
H	7.13662794016592	9.43874777022234	10.27966800125126
H	8.53111159588488	8.89172548592337	11.26505358105237
Rh	0.00194071016457	19.23437443948484	29.02679654153674
H	-0.10955526206436	19.29273959004250	27.50501978682494
Si	-0.51238233424942	19.32630314430233	25.05521255953785
C	1.36047863133223	19.30028513532001	25.31150383094589
H	1.80498802643267	19.92920471203874	24.51412440824892
H	1.60145034716250	19.80783209559883	26.26317590873914
C	-1.42930091777908	17.80465177516622	25.68363033430157
H	-1.93765057660335	17.26992275759059	24.86217246523567
H	-0.75267185085253	17.09273044002672	26.18582109686876
H	-2.19054369893383	18.10460935361489	26.42418455985987
C	-1.31921121384042	20.93250683016173	25.64451133727392
H	-2.21083682633388	21.11663872868961	25.01224622976623
H	-1.70504989662096	20.74338844997364	26.66275181889105
C	1.98332114132313	17.89636901391073	25.30126524180593
H	3.07971239036931	17.94061948482053	25.43825277040032
H	1.57784001786794	17.27220536273565	26.11640573719546
H	1.78534196305773	17.36441533505640	24.35143849593568
C	-0.39054960604695	22.15564929665397	25.65083480851026
H	-0.00547062384102	22.38930389261307	24.64073233703592
H	-0.91006374646224	23.05665801694810	26.02484656722381

H	0.48109605842746	21.99004780903796	26.30856544037767
C	-0.76967570509798	19.36077019258883	23.09096629517672
H	-1.87262274120879	19.35289889668974	23.00310271602119
H	-0.35138693454211	20.34016307142063	22.79411833600469
C	-0.11682393956700	18.22511646982750	22.49178597406384
H	-0.61208628538187	17.24857455238098	22.43245861470232
H	0.92850060763065	18.27579719184652	22.16541250422240
C	-0.49581371528279	19.20751403853355	31.55489419300783
H	-0.99743786770255	20.17784587951831	31.65362405218706
H	-1.13173419336095	18.31588916923075	31.61741391246704
C	0.85568036772214	19.11144590050822	31.45588916700609
H	1.36234669530522	18.13881477323914	31.43279108317597
H	1.49818213673045	20.00032960192186	31.47436970775952
Zr	-10.13126449441701	8.20504676002710	30.90580514477712
O	-12.25165164025935	7.82197814808969	30.52866487606860
O	-8.67901106537708	7.81615503541623	32.75824779826023
O	-9.13115696770676	10.12916024145891	31.23540185399227
O	-7.99142373457058	8.04591481444675	30.13557186958660
C	-12.96882861971267	7.36915437431025	29.58039019805036
O	-12.76613810793652	7.53115497317309	28.34549024540669
Zr	-8.11337015537890	8.08100066165381	27.89108114236812
O	-6.82251237319973	6.55014718947698	28.37222628652466
O	-10.10179304116092	7.56028887526376	28.68930526739454
Zr	-7.22409351862147	10.17179482363709	30.63069597639663
O	-5.27070872896343	9.66604574957361	29.56530869522732
O	-7.96788606519236	10.00180809095687	28.67179997940985
Zr	-11.43680685116051	9.06132418634595	27.37159829443655
O	-13.24086416997009	10.12862072627445	28.32617767522495
O	-10.02894511563735	6.22390317733219	31.06797291763358
O	-10.65957462633494	9.87234852918894	29.38310342110134
O	-4.93943305553728	7.25469370882183	30.15832954562206
O	-6.37267595867478	8.79822902303443	31.99789554409732
O	-12.54205983170350	9.39913317213717	25.75403066999898
Zr	-10.54936099097862	11.73776814562941	30.82152908216171

Zr	-8.89346542250458	11.35805759434443	27.16845114476780
O	-7.03241690889346	11.43846938174435	32.44672296168785
O	-8.88163941171230	6.64337598436564	26.38275948554870
O	-11.15480305562451	8.86017465832435	32.72807033466653
O	-11.06442176988526	7.20637631105099	26.09681338339369
O	-9.42901499673611	9.34099901640275	26.75387516390294
O	-9.51127691184879	12.33954500785869	29.04231180474780
O	-11.06872609360518	11.23206901775904	27.10026445085962
C	-11.42235272821432	9.95450466781888	33.32475499986854
O	-11.25243389580064	11.11407220262512	32.86519002170660
O	-9.46144505793031	10.02383876634390	24.04247843291961
O	-9.11069048593576	12.49843020624402	25.57472836356696
O	-8.90252906154540	12.69001436852284	32.16215200590573
C	-7.84190280516198	12.37008467773030	32.76365590521909
O	-11.04919597692614	13.65525311549196	31.03935402212838
O	-12.51596785192237	11.45912458077327	30.35841368668386
C	-10.02434586209191	6.54326726366756	25.82699075021175
H	-11.39706478706672	11.49202680731235	26.22036081754638
H	-7.59519064410650	7.20558430847582	30.43085303399056
H	-9.47140195757515	13.31279322466242	29.01880496462520
H	-10.32516792540747	6.61135385285485	28.64218875159248
H	-9.17707689833295	11.98384471428272	24.73814597641321
H	-10.45052511248475	14.19182515417856	31.58820816995416
H	-6.52131248556809	5.91923262854912	27.69644243904632
H	-9.47077713832219	5.83135863574847	31.76060963399327
H	-5.85343913879020	9.20230677146150	32.71672886935168
H	-9.01823569745757	8.33218350306468	33.51132026152456
H	-7.74127333916728	8.17713776799347	32.56857407660763
H	-5.17594214987728	9.88493921990558	28.62163653753692
H	-4.98939163783179	8.66292657884363	29.71199384053725
H	-5.42321322218959	7.49319662288402	30.98924658564557
H	-5.62072729532818	6.85680535754769	29.53346647987231
H	-12.60263445043130	8.68963983759931	25.09138139125075
H	-9.47273075184025	9.59583101430319	24.93445867516352

H	-8.59415753954081	9.77916565938316	23.67832766988824
H	-13.13601827365852	12.12752929921890	30.69749860470998
H	-9.68775686273633	9.97086839583493	29.18122535908016
H	-12.96878646755225	10.67379364874110	29.16226777571105
H	-13.48770222230420	10.79516740740344	27.66015660927487
H	-13.86130845909024	6.77383305053680	29.87619714791991
H	-10.11165760587659	5.77695733829334	25.02327072188917
H	-11.84401215624381	9.86339787131342	34.35048693671858
H	-7.56927652870062	12.96205764938994	33.66826777619186
Zr	10.24492391468708	7.67085764338957	30.26899586560600
O	12.35614381813368	8.09352958129061	30.64411141738930
O	8.84068602009181	5.79003061645397	30.68221006127160
O	9.22638971333541	7.32289331811649	28.36176959258371
O	8.09732539613219	8.40423616777906	30.44469418250907
C	13.06811509224065	9.05685932816229	31.06888327732014
O	12.85105163967892	10.28654053349719	30.88221002844924
Zr	8.17051934476927	10.65178395226615	30.40638434530656
O	6.92528621719353	10.13777278192296	31.96511733227075
O	10.18000879319976	9.89310416069797	30.91247961877237
Zr	7.30806898876044	7.88747240356405	28.32491598365780
O	5.31305828520668	8.85306380851211	28.88771112035051
O	7.96970387428202	9.87750184223064	28.49344598973641
Zr	11.47540545643617	11.23359791407426	29.38862111148070
O	13.27619425285743	10.31802013626675	28.27241711311961
O	10.15734268637692	7.51250330983048	32.25211011603857
O	10.70756765231763	9.21608570067135	28.58729050376732
O	5.09203266751189	8.26759897502154	31.31872930306206
O	6.52703088699760	6.48459004055010	29.70423814352297
O	12.50142194217777	12.89424358551116	29.02351371118557
Zr	10.64722301718387	7.75064731716304	26.75521859378212
Zr	8.90583986744397	11.36524240488202	27.11855768951344
O	7.15850998044392	6.08733860284572	27.03843096866315
O	8.91815808993698	12.19128202357198	31.82195057409433
O	11.29861369784100	5.88213829670609	29.58426107162672

O	11.09626374542357	12.49926078560590	31.25112385216104
O	9.45646841574350	11.79556828214173	29.12657927241623
O	9.53852048657426	9.49116637163165	26.14689087000934
O	11.08108536811293	11.47333860679937	27.21348882213278
C	11.50154363857936	5.24168574534607	28.49940530839159
O	11.27658745194599	5.65948551814877	27.33411304309173
O	9.37319004308724	14.50790169100516	28.44199549900736
O	9.08030481290963	12.95976705224114	25.97342703798492
O	8.98939514593113	6.47348905312072	25.75884477624423
C	7.95666316397461	5.82504855565970	26.08058734408003
O	11.24410893986189	7.50299413357483	24.87737777951399
O	12.60846701855217	8.12421828276468	27.18558348607953
C	10.05538232793814	12.75937412022179	31.91657562468969
H	11.37430036697000	12.36020925045774	26.93504130257384
H	7.72630575841259	8.11426904815786	31.29815307813142
H	9.15323839169308	9.37406400261811	25.26035341247033
H	10.40967947729727	9.94259355672456	31.85978736831375
H	9.16482394167618	13.79442741934750	26.48893409859653
H	10.68676230047734	6.97275485292442	24.28198774052584
H	6.62649986992053	10.79710000713562	32.61401128732638
H	9.61866270621184	6.80349957673453	32.64293082135503
H	6.01601005031330	5.75535584823931	29.30847639821763
H	9.19097808023755	5.03426835575200	30.17812639953065
H	7.89713631729514	5.95731983514276	30.31894587344947
H	5.13311268648472	9.78184079587988	28.66163390037378
H	5.07663950710113	8.70174360186842	29.89910576335654
H	5.59278081385063	7.45233767613110	31.06352474896898
H	5.76283020331542	8.91819206821350	31.69032704518978
H	12.55612046881912	13.56168124982653	29.72883534575951
H	9.43853029523774	13.61525289010500	28.86393596926044
H	8.43306069757763	14.73177733899830	28.54532549384235
H	13.26679504635703	7.71153845987612	26.60021981754597
H	9.73028628953640	9.38437732090538	28.50496702115383
H	13.04213516169561	9.39836480724127	27.86046348041793

H	13.34046237109049	10.90027536269477	27.49259737716229
H	13.97013775876331	8.78398257598077	31.66050674848045
H	10.13656940703940	13.56592164750971	32.68075692807263
H	11.91675205513441	4.21476260083284	28.60790616214410
H	7.70129621667615	4.93059419958282	25.46669925553804

C_2H_4

C	-0.00000000015860	-0.67142605353808	0.00000000057401
H	0.93402228748798	-1.24921573558273	0.00000000082891
H	-0.93402228621172	-1.24921573705412	-0.00000000146717
C	-0.00000000072501	0.67142605203319	0.00000000086644
H	-0.93402228687412	1.24921573915977	0.00000000074695
H	0.93402228648147	1.24921573498197	-0.00000000154913

$Zr_6O_4(\mu_3\text{-}OH)_4(\mu_1\text{-}OH)_6(OH_2)_6(OOCH)_6$

Zr	-7.71280092894852	28.40472924478357	8.23544543550537
O	-8.07574856853772	26.72996075124609	6.72358557505243
O	-5.63428846197923	29.38894122296026	8.79036403708569
O	-7.76521208570799	28.65114340733867	10.36821257836354
O	-8.15397250984210	30.61161471632704	8.80547560822623
C	-9.11940857805888	26.19139650607798	6.26260600499500
O	-10.32664182193357	26.43578937936394	6.58498769320940
Zr	-10.38726212052911	30.92097918082715	8.32599672651970
O	-9.56242109822553	31.73218199779984	6.67854725205128
O	-9.70437593298674	28.97968266967385	7.40681136818906
Zr	-8.18034436176977	30.46739509527205	11.14369915607157
O	-7.79165557790485	32.89272413256295	10.73854426784144
O	-10.03915177120372	30.81710313397227	10.44447487410605
Zr	-11.17961299958491	27.43558978794657	8.36767955411561
O	-11.37719027026258	25.19751825455554	8.79557030050700
O	-7.04691848265873	29.15268074573536	6.49860070971219
O	-9.25814531356572	27.10181157088718	9.10602353316225
O	-9.75484131079933	33.14185221993252	8.94706204241463
O	-6.12459146220597	31.08695929935425	10.70712557156896

O	-13.21471645044538	26.98767116841570	8.87730971432270
Zr	-8.96276592672776	27.11568641509608	11.10891505180725
Zr	-11.60671386059132	29.57824099068200	11.26823632623563
O	-6.98552939596342	29.59727794514641	12.85322609841317
O	-12.17911674653123	32.15754746468930	8.90476941454027
O	-11.97259710717823	30.51968519402757	6.73033159471491
O	-8.93051335650147	31.61360156304068	12.88296538223006
O	-6.30413382442338	26.77044888007194	8.81103322869235
O	-12.36165567970396	28.28068866096961	6.67466226814680
O	-11.47697936842066	29.29221583884424	9.16385692752724
O	-9.61054815086391	28.95576648554973	12.15091226806084
O	-11.15577723797638	27.35961329450498	10.75980102606020
C	-6.42181965784504	25.82337744956906	9.64217532775101
O	-7.32843418140133	25.69123051503393	10.52248700762930
C	-13.03631420819104	32.04942815786584	9.82824455003643
O	-13.03668657648451	31.19549280467939	10.76660676838166
O	-13.65345836728215	28.59584828589593	10.77029957199183
O	-12.19038559379589	28.80024277916981	13.04212974141547
O	-11.18001614099665	31.25964490587877	12.83208536967364
O	-7.46179505085336	27.37077007974167	12.81159531298441
C	-6.84630118501401	28.39324146478448	13.23556115481093
O	-9.62407761388000	25.60596387994637	12.32926016171815
O	-9.72088926565929	23.90550877580459	10.30754237582178
C	-10.14330796893200	31.83236299553596	13.24339404720967
C	-12.55679069534217	29.48371808992524	6.30887532025156
H	-11.66632225600823	26.74367636353161	11.31560364164078
H	-7.67216507483020	31.21096623203914	8.20574116552669
H	-9.74385204121136	28.88324018799525	13.11458318932312
H	-9.59654901856655	29.04121820104926	6.44056420089188
H	-12.94802167114223	28.19215731251700	13.00272350129262
H	-9.15873387688412	25.31447732704133	13.13105010505408
H	-9.74941732855929	31.39354959674916	5.78803901751943
H	-6.33105846280436	29.80963905453729	6.52864548051393
H	-5.53331887868151	30.88659023781061	11.45504648411426

H	-5.09274039141529	28.66954997962196	9.16344855291135
H	-5.76254188096178	30.06854108309096	9.54751109975841
H	-6.84536178143413	32.51299943391844	10.67629051628412
H	-7.89862552693851	33.28666116577233	11.62525767009685
H	-8.98833648238777	33.13767487732304	9.58061468660589
H	-10.51160648668931	33.48272735601758	9.45688252095253
H	-13.79756463321954	27.22837851675877	8.13518383876480
H	-14.23563997885852	29.30378316371677	10.44081014762504
H	-13.53060867541467	27.91744545318222	9.97050272310913
H	-8.80483150107202	24.05252020132936	10.00370141469218
H	-9.73516787982369	24.39237341155331	11.18816082169953
H	-12.27937246259688	25.12761073539247	9.16337539053125
H	-10.72439789382872	24.66447710395807	9.37555857282386
H	-13.87758674175363	32.77848473369976	9.82081201231419
H	-10.27277563545255	32.62480419889267	14.01781012464613
H	-6.09812139593934	28.22146834447134	14.04480448112012
H	-5.64940012096763	25.02293321850291	9.60024916310012
H	-8.97480167204693	25.41726829990147	5.47315920058008
H	-13.32889047280035	29.63240231813549	5.51796169738163

HCOOMe

C	2.67637382958351	11.34662788436662	-0.00021806649886
O	3.88576319697379	11.45111879344386	-0.00443745941983
H	1.95389910817215	12.19448137710213	0.00196476960333
O	1.98944577929939	10.18929087398265	0.00260282150993
C	2.78960978812352	8.98637111372766	0.00045109078738
H	2.07712393874432	8.14930004689273	0.00278973389843
H	3.42787014718074	8.94650374690772	-0.89910343600781
H	3.43326360192256	8.94691333357658	0.89617384612743

6-TS-light

Rh	6.40026708134057	8.44107196738372	15.40161932527501
N	6.55261874864230	8.16263932955598	13.38366628508348
N	8.39503276272191	8.09035071205466	15.60428236253452

N	6.25089131072602	8.74153226836862	17.41191344953321
N	4.42130981430103	8.87853847646784	15.18624344640742
C	5.49830133880428	8.07973464734260	12.48236709773033
C	5.98891157931523	7.61917184787562	11.19777377192525
H	5.36840553309738	7.43364750926227	10.32000154883840
C	7.34544529391888	7.45022463253664	11.32199081219809
H	8.05030580906192	7.10167898227964	10.56544619976793
C	7.69275721111816	7.78741015544529	12.68890587694962
C	8.99353761762798	7.69723102001406	13.21971877215847
C	9.30751445608368	7.86765862535581	14.58042705616860
C	10.64952712801840	7.80897141649184	15.12583447013507
H	11.56040848616173	7.68737554301727	14.53748820006812
C	10.53789083638697	7.95470977525657	16.48632524947990
H	11.33971363906264	7.97715353745508	17.22618428007807
C	9.12765728549996	8.11381013478831	16.78316299200976
C	8.60480675928328	8.28673537547165	18.07836300684727
C	7.25280530493088	8.56037703103280	18.35555317255645
C	6.70438882410782	8.71717181817148	19.68864356763249
H	7.26778125845511	8.59936360248664	20.61569124218510
C	5.37374121456096	9.02200647298303	19.54326908943558
H	4.63651136478530	9.19990707546809	20.32787030172346
C	5.09603140445677	9.04608772452466	18.12056459002817
C	3.83256114239627	9.31685648876305	17.56440568269582
C	3.53753609811903	9.24669881759327	16.19025930025974
C	2.22906059455582	9.50181378900447	15.61868355667079
H	1.35970220806770	9.85473699433887	16.17549271087998
C	2.31436078365836	9.22046560531240	14.27779791531337
H	1.52967199200191	9.30083174920923	13.52392493548004
C	3.67883613971876	8.80577799420466	14.01604542430021
C	4.15272468099497	8.38792974825740	12.75784128366998
C	10.10516393810840	7.36687279431594	12.27970064622867
C	10.44483083647788	8.25172932629332	11.23127487244638
H	9.89020159408067	9.19169931365837	11.12419125464723
C	11.48555117172208	7.94518742196284	10.34545105727499

H	11.75027651051027	8.63811973821969	9.53999983154798
C	12.20481010447675	6.73965138285197	10.48989648608991
C	11.86799094188680	5.85020961691175	11.53182411141016
H	12.43193294931248	4.91486790946411	11.62512394777230
C	10.83074240555741	6.16119852135322	12.41782817367639
H	10.56354900625553	5.46428165256700	13.22106557235076
C	9.55280485010830	8.18851243195299	19.22766890884069
C	9.81858513416444	9.31132902678286	20.04362311852939
H	9.32422926651017	10.26439736580603	19.82019628123265
C	10.71431954492177	9.21980936092673	21.11643911295259
H	10.92302494105634	10.09498546506976	21.74058512537250
C	11.36064059196485	7.99679166381081	21.39574329490640
C	11.09870284336545	6.87204351375536	20.58566854773004
H	11.60497206963990	5.92812215993055	20.81884820833816
C	10.20629264392173	6.96751547420130	19.51217351123463
H	9.99885053025119	6.09052965857520	18.88738921019375
C	2.71848001847840	9.65923772627729	18.49872888942151
C	2.74527989113818	10.86010765390387	19.24247442145818
H	3.59125327907557	11.54721995769253	19.12021882294332
C	1.69929614907463	11.18253031872562	20.11673976333683
H	1.72047039688901	12.11854075562828	20.68455408541884
C	0.60557276331508	10.30321087524209	20.26556160665565
C	0.57575047417644	9.10050295853905	19.52898266632150
H	-0.27753479921666	8.42523813620120	19.66118224277505
C	1.62058286062222	8.78267637647144	18.65440430758291
H	1.60144967101238	7.84330122889726	18.08900575715782
C	3.15610224998544	8.22821755845827	11.65778976416360
C	3.23508158823262	9.00389853796316	10.47905818858322
H	4.02721027566657	9.75633438030620	10.38459942312931
C	2.30816033535535	8.82754955596234	9.44352527125126
H	2.37087568269949	9.43563302651905	8.53503460135590
C	1.28285846952937	7.86509234639125	9.56645125990848
C	1.19571261476541	7.09110261037819	10.74230548683584
H	0.40244089058381	6.33897652473961	10.82602485030333

C	2.11864321717382	7.27499415208910	11.77647652217410
H	2.05227400393558	6.65993826851508	12.67999024192317
C	0.27270972589973	7.62680337874958	8.49264339762148
O	-0.63552496736887	6.81108542580161	8.56226886342050
O	0.47316784103772	8.42260542025202	7.41384104082333
C	12.32427415975127	7.83786440220228	22.52578396388965
O	12.90666019880367	6.79828788628696	22.80048296421227
O	12.48704803969004	8.98642168027737	23.22614439744370
C	13.32312860789101	6.35755331840082	9.57654250363058
O	13.97488965234711	5.32711414512035	9.67086521858300
O	13.54481456139632	7.29028940632120	8.61902166517251
C	-0.53963000420143	10.59414863794459	21.17929344501877
O	-1.50904579440132	9.86442066212118	21.33126417127603
O	-0.38934847824838	11.77251527673784	21.83127681001039
C	-1.45644975148981	12.12959047755281	22.72684442766004
H	-1.56798637727296	11.37241448117067	23.52252906802361
H	-1.17696691796378	13.10153097136840	23.15837921206601
H	-2.41248295702125	12.21057135044927	22.18077339641329
C	-0.46090641581131	8.25381437768160	6.33359817941381
H	-1.48746632135898	8.48779025010416	6.66591945952492
H	-0.14369068740537	8.95338405274256	5.54683322655020
H	-0.44018098765678	7.21564339776412	5.95884039893321
C	14.61185142360551	6.99883287704815	7.69990384917143
H	14.40366819315517	6.06854411019089	7.14297652641667
H	14.66063765965509	7.85486233782283	7.01157424018743
H	15.56856874180109	6.88105447179788	8.23792907723198
C	13.40272604930604	8.91695932641206	24.33295183786770
H	13.06490662483929	8.16940798326474	25.07175911278479
H	14.41302301864990	8.63812639941633	23.98601347923380
H	13.41372399211620	9.92134614819344	24.78024263469533
H	5.86448153761232	7.03773895302743	15.38366957469579
Si	2.00880664030139	3.57796828344084	13.25675677349851
C	2.54534024402798	4.79890454834977	14.61870752394161
H	3.19302518121405	4.22776130718167	15.31856022673244

H	3.20851532583897	5.56042826561482	14.16261956688379
C	1.21801668826976	2.03601177449638	14.00972637080129
H	0.89628275194635	1.32703622152551	13.22610105924443
H	1.94505472665804	1.51524406320276	14.66472027076769
H	0.33445530366959	2.29364573287663	14.62043946024359
C	3.51804175134072	3.12094846746943	12.19839413667693
H	4.20415168678902	2.54339952558153	12.85560912512311
H	3.19247087637270	2.41365066631538	11.41054167432793
C	1.40791629609447	5.49013016529280	15.38964016841833
H	0.76512598854880	6.08283604839010	14.71275830288242
H	0.75535450095466	4.75790972637691	15.89973703796073
H	1.79851905194041	6.18278903838250	16.15828133233472
C	4.26198368426116	4.31782064107530	11.57701059503563
H	5.14455816577539	3.99487984823003	10.99448792131307
H	3.60703590624849	4.89396481835862	10.89850361808942
H	4.62300231469600	5.02324939094999	12.34724408113385

Et_3SiMe

Si	6.01045078911752	5.59940682354152	14.87620386899093
C	4.42581539162332	6.23993402347131	14.06364376388297
H	4.44937605998000	5.95794633753028	12.99179069208917
H	3.56713771131304	5.68701078516018	14.49465126001210
C	6.12388648100887	3.73573138989171	14.60831862223445
H	6.14267286936277	3.48785636091995	13.53118134479026
H	7.04158515287779	3.32254571032570	15.06613669176360
H	5.25851478447577	3.21223378321412	15.05424291771404
C	5.98016095370596	5.98537337842362	16.72836832713958
H	6.92263149518088	5.60590450482677	17.17259846875929
H	6.01648102415371	7.08661228891356	16.85093972121305
C	4.21194395185655	7.75717429022016	14.21136206965137
H	5.04138249433606	8.32941249611274	13.75559143488593
H	3.27644524755638	8.09668651737445	13.72821547266926
H	4.15830570645595	8.05740368367499	15.27447591129274
C	4.76634149669570	5.40910993430944	17.48015846266761

H	4.79122643874943	5.65057021808368	18.55950179461085
H	3.81580156035032	5.80651463086471	17.07832924587096
H	4.71949280070741	4.30769524734854	17.39278145040480
C	7.51454075750582	6.45142026507588	14.10613480026709
H	7.47548750861058	7.52693658703782	14.37228935974347
H	8.42230016501290	6.05551640515648	14.60509896785943
C	7.63148765834356	6.28705381623009	12.58004758481200
H	7.69526269929050	5.22198042602691	12.28940474269102
H	6.75398180173973	6.71223557816111	12.05845819028571
H	8.52846609998945	6.79077319810422	12.17326638369821

Et_3SiH

C	1.80127923706658	4.77024515097979	0.03382953376053
H	2.13795181136845	5.32113329367065	-0.86785405832241
H	0.69483343151608	4.82445250402048	0.02125657705216
Si	2.41922101541518	5.70177288612024	1.55784558292679
H	1.91453831594463	4.98752215329666	2.78341275438728
C	1.74761352472194	7.46833574673832	1.58195192214212
H	2.16300005307988	7.98062142620988	2.47209596922309
H	2.16286319964136	8.00769236647290	0.70639603795728
C	4.30882861918572	5.70814437068353	1.61368354482299
H	4.65710575448899	4.65702843098435	1.58045111352264
H	4.68046698053600	6.17616421370850	0.67950233629567
C	2.25778042444662	3.30150047696827	-0.03453373639932
H	1.84931752195769	2.78094367912198	-0.92061814958482
H	3.35884767948239	3.21868870247937	-0.08679047010683
H	1.93194187786564	2.73648188580879	0.85844242255705
C	4.90225197025917	6.42144565995470	2.84220023575057
H	6.00665853092083	6.36712079249812	2.85967726114968
H	4.62846948765048	7.49214434280141	2.86345620843518
H	4.53581704076783	5.97377297474147	3.78466658641600
C	0.21101582938220	7.56162023085330	1.58919743770665
H	-0.14018276192733	8.60894774802775	1.64152612489810
H	-0.22955009331500	7.11584058578160	0.67859464383494

H	-0.22104013045537	7.02585984807788	2.45470514157463

6 (with Et_3SiH ligand instead of Et_2MeSiH)

Rh	6.04441178261936	8.22223188578047	15.01806145860074
N	6.27946049292170	8.13780263838085	12.99151090321876
N	8.02451830196850	7.77553625178243	15.26893889570772
N	5.85210864201110	8.49059403934031	17.03211200596330
N	4.08062156218649	8.73200284517512	14.76381251097752
C	5.33593678528855	8.46869309949477	12.02960264121247
C	5.94261879868241	8.40471181715759	10.71229460161292
H	5.44449449221167	8.66440307947396	9.77698685638441
C	7.23646839627326	7.97875023419857	10.88543559668589
H	7.99935954647031	7.82508305272349	10.12035797493469
C	7.43864030632724	7.80138161560888	12.31159087231128
C	8.64591460574561	7.36864015387139	12.89580717863191
C	8.89705477693954	7.34503589671819	14.28174525050764
C	10.12596599506321	6.87031630454310	14.89103501399814
H	10.97171655020878	6.44416234181520	14.34918309362907
C	9.99927055078315	7.05300955486846	16.24630675666612
H	10.72189012104371	6.80470262924621	17.02546125666872
C	8.68787043177458	7.63123032745439	16.47654533968871
C	8.17943602373757	7.97961346911916	17.74441651289352
C	6.86236404165852	8.42064190060668	17.97941629358101
C	6.33721534207213	8.79494268256851	19.27955248543613
H	6.92572932070392	8.87430153657382	20.19498769256714
C	4.99400578295895	9.03040677926870	19.11748522488965
H	4.27251378634729	9.34388452067258	19.87380808173013
C	4.69161892852548	8.81906766643363	17.71368477033022
C	3.40336968791777	8.92141059769934	17.15017413932888
C	3.13164871848562	8.85272169037386	15.76844225183091
C	1.80925816645822	8.94382852930134	15.17826104944073
H	0.87396944457908	9.00564346299661	15.73677168477755
C	1.97590333857549	8.93203729668820	13.81418711572382
H	1.20314320357081	8.97871700591587	13.04520921073816

C	3.39965333825100	8.81204624254978	13.55990478631808
C	3.97921827025046	8.75846938905128	12.27527343245320
C	9.72508840611064	6.89972041196935	11.97928823068315
C	10.96812447414557	7.56945084046410	11.91421763906085
H	11.13182820748046	8.45403375903061	12.54113133806478
C	11.97527658205732	7.12464800600799	11.04878348928376
H	12.93327305490665	7.65219198263418	10.99485931271772
C	11.75799505877375	5.99443249493360	10.23184272835246
C	10.51999076434215	5.32060943008251	10.29299386032872
H	10.36938534116614	4.44185658310574	9.65528136371875
C	9.51413557683316	5.76989983640111	11.15529305267299
H	8.55459888072022	5.24218666385839	11.20909018116916
C	9.08710110784719	7.83326061278479	18.91951507296315
C	10.27058269749040	8.60078563426674	19.01604161314870
H	10.50816490999662	9.32102250534360	18.22408273964446
C	11.12377514298510	8.45713114816463	20.11569806761671
H	12.03962369883947	9.05298796597450	20.20377729997614
C	10.81511536844307	7.53952617063754	21.14184619473121
C	9.63686268565436	6.76779869497734	21.05197372939892
H	9.39957971906732	6.05003479103948	21.84409735388169
C	8.78317240536119	6.91658328831684	19.95205426219751
H	7.87313450048855	6.30998191175023	19.87560446000616
C	2.25629579473915	9.11254620434339	18.08443113722667
C	1.44788533066791	10.27052122785262	18.02042898017165
H	1.68033311012727	11.04472472612459	17.27953228565744
C	0.37337301003996	10.44377986065333	18.90179911432891
H	-0.24333994957177	11.34719866879340	18.85167265057023
C	0.08341882925445	9.45616072714577	19.86747114854529
C	0.88694724895086	8.29889470538106	19.93856192241979
H	0.64652709374032	7.53909430042388	20.69120683419685
C	1.96201515564007	8.13100975432311	19.05962002056434
H	2.58047465410308	7.22731453577964	19.11392033872030
C	3.08544759299739	8.99654889446235	11.10502802070778
C	2.40906939157681	10.23078498057996	10.96578665258128

H	2.56358811181138	11.01193303399326	11.71966855305700
C	1.56374546261281	10.46693836949203	9.87490078690755
H	1.05041013156638	11.42837624780882	9.76928555522438
C	1.37357838399858	9.46624353725129	8.89809784118096
C	2.04301135061640	8.23172561533754	9.03096684537156
H	1.87991149127178	7.46178366363893	8.26787568926919
C	2.89031385563109	8.00041042095393	10.11996937219460
H	3.39966585914800	7.03592265174813	10.22447444193894
C	0.48524826980081	9.65935981863331	7.71357483387760
O	0.29545645531919	8.82120445469097	6.84342139563004
O	-0.09729201549846	10.88303457597443	7.70376689960683
C	11.76355889054932	7.42645656188697	22.28970267080534
O	12.79588284869978	8.07221131171640	22.40383702500232
O	11.35303626761624	6.51756595626302	23.20747355313436
C	12.79395159694894	5.47427706971447	9.29044511548903
O	12.64739241429234	4.50000426554047	8.56576029306375
O	13.93262017937007	6.20831727068587	9.32415838488526
C	-1.04903063546666	9.58323606258327	20.83241071514864
O	-1.32979216727989	8.75380644864174	21.68626686615091
O	-1.74479515015274	10.73319358669460	20.65938560324442
C	-2.85200496127716	10.93181309514346	21.55532289177320
H	-2.50558551181292	10.95786117939414	22.60326508998309
H	-3.29917686303168	11.89638116759584	21.27484538681784
H	-3.59087283772868	10.11850065164344	21.44860861014961
C	-0.96572921186563	11.15150567367992	6.58966337274830
H	-1.34281897222719	12.17354433071080	6.73902054044012
H	-0.41161171932460	11.08060379659593	5.63741610646838
H	-1.80263346271712	10.43186385000966	6.56466691623864
C	14.98119443397677	5.77371767780025	8.44144742185888
H	15.29457285952248	4.74350202364083	8.68505080118576
H	14.64287494597518	5.80020451907105	7.39088919135426
H	15.81489199324993	6.47428914596133	8.59384913349228
C	12.21520093034893	6.34925334169377	24.34598476089575
H	12.32102604178048	7.29893460833661	24.89890549461749

H	11.73613522076327	5.58806273545350	24.97856192606207
H	13.21776464257758	6.01204722549089	24.02971040236800
H	5.76916888041141	6.46323776736798	15.10166499305457
Si	4.83816153941221	5.23052309302731	15.33970282057195
C	6.00285571898039	3.79001539700057	14.96882977102290
H	6.49015466248066	3.48147711533567	15.91358508521283
H	5.38014811121963	2.92922621389544	14.64961412724908
C	4.25504869235338	5.32158925438225	17.12109586925320
H	3.49043207373148	4.53341520032878	17.27535320844862
H	3.72202169805731	6.28588500243277	17.22243291080789
C	3.40092183526680	5.35065277392764	14.13937815291787
H	2.68034977418773	4.55487493317383	14.41976834216080
H	2.88049849578463	6.30804740181817	14.33051000716724
C	7.07009332552752	4.11492106418133	13.90551838057673
H	6.61561642200851	4.46175041901313	12.96027270173689
H	7.74180739829124	4.92038157955942	14.24979759975101
H	7.69614195218643	3.23441711706694	13.67064877217776
C	3.78279774214011	5.22794713714443	12.65502092255607
H	2.90375258473361	5.36635398931847	11.99943059900733
H	4.53031656564615	5.98848486111080	12.37031535071256
H	4.21346213563660	4.23635494502571	12.42604365960755
C	5.37640164489064	5.21078410638118	18.16690967254431
H	5.00275833661667	5.40650596354728	19.18875516828962
H	5.83343593381157	4.20482137938189	18.16772051280652
H	6.18174622764962	5.93924413756317	17.96838214156843

6-TS (with Et_3Si instead of Et_2MeSi)

Rh	5.95057770982749	8.19789963705171	15.04247051185711
N	6.11958298199629	8.00197714541476	13.01966066059016
N	7.92090954152072	7.70539993873984	15.25595597864724
N	5.81803462367742	8.53524723503818	17.04878682467027
N	4.00428780271279	8.77511392240688	14.81783755863523
C	5.13233652316025	8.23891008147000	12.07298539708535
C	5.67668418847382	8.05568024661864	10.74054869745977

H	5.12857931347787	8.21988846617676	9.81138170439630
C	6.98567404627982	7.67068144289945	10.89055818404321
H	7.71651320266778	7.45843966640035	10.10847740397717
C	7.25364533436183	7.62359307203252	12.31560067606702
C	8.48901633690988	7.25006106766463	12.87730310042833
C	8.78233037640525	7.28709908265485	14.25254675378140
C	10.03780860530982	6.85057202101162	14.83446486662216
H	10.88104759636431	6.44434958707060	14.27385532125605
C	9.93182053712150	7.02016773610152	16.19258159962413
H	10.67406419129367	6.78465365493938	16.95693821354029
C	8.61292158227148	7.56792840878241	16.45062896170510
C	8.12454370178483	7.89812371296888	17.72916189494167
C	6.82866772953699	8.38373587943581	17.98553345980426
C	6.34822729748341	8.78516250955018	19.29491611614064
H	6.95132846373289	8.80729702412438	20.20403763618830
C	5.03075174052308	9.14111572330665	19.14714045410227
H	4.34407552128857	9.50780043117895	19.91187602895358
C	4.69641560521924	8.95686707312450	17.74744426973650
C	3.41157461890460	9.14708420859784	17.20648977647257
C	3.10009417095048	9.03453764120735	15.83856877455460
C	1.76546023174326	9.17429024318894	15.28764603110781
H	0.86080642598245	9.35036984774427	15.87175071764021
C	1.87582452016271	9.02938759664864	13.92676590098138
H	1.07823194414996	9.06186383397074	13.18293689350390
C	3.27742770226761	8.79314713808703	13.63681324756681
C	3.79563419756906	8.58395080352187	12.34466909162665
C	9.54468171871230	6.74623552308901	11.95055620203930
C	10.75674912403809	7.44980828230240	11.77297081150967
H	10.91543796826046	8.38887858295897	12.31664296430004
C	11.74221370770802	6.96688566918413	10.90237454542249
H	12.67649049148019	7.52034909908251	10.76135425568893
C	11.53322041129486	5.76418892949214	10.19408910590784
C	10.32521455237329	5.05643431495435	10.36818118241348
H	10.18037592315145	4.12189673242771	9.81384618540151

C	9.34099457645477	5.54375663114157	11.23489942007510
H	8.40358846858536	4.99232253514586	11.37477471702191
C	9.03147135816887	7.69465151091479	18.89671372790520
C	10.21643185330232	8.45192165757036	19.03456765054957
H	10.46044359363658	9.20573134823817	18.27644321258875
C	11.06513052610462	8.25792414198220	20.13173666424964
H	11.97811446774714	8.85287848029521	20.23889417975508
C	10.74407816260472	7.29577447801842	21.11318540900603
C	9.56334002757440	6.53503552693268	20.98000733749773
H	9.33105103297238	5.78790795346426	21.74776805074462
C	8.71566954321398	6.73391142180735	19.88483482929120
H	7.80154579886777	6.13797625255474	19.77685401991343
C	2.29987170270156	9.46474419778630	18.15082882610117
C	1.62480637474609	10.70450137454461	18.08620739839718
H	1.93801284740437	11.44547896398190	17.34097408523762
C	0.57626790590144	10.99473018798339	18.96846990172624
H	0.06062175250409	11.95944708389631	18.91872137492760
C	0.18003782055705	10.04422071690176	19.93366243417627
C	0.85075239650769	8.80506126057167	20.00378103643442
H	0.52842663390502	8.07539190557975	20.75560884675453
C	1.89992157085066	8.51962389078797	19.12341359867124
H	2.41365060964960	7.55227138709034	19.17232604637379
C	2.85062521516391	8.68834305479854	11.19469447898076
C	2.21814831882532	9.91536401621804	10.89085937623388
H	2.44673107899289	10.79909337615294	11.49858394582024
C	1.31875646398774	10.01187938928519	9.82166581543612
H	0.83678170368165	10.96674271207445	9.58710221280507
C	1.03064798817111	8.87614980048513	9.03489395703835
C	1.65882497626342	7.64853343262181	9.33227899159845
H	1.42014361839349	6.77424957120344	8.71552820203788
C	2.56007911072569	7.55638173975095	10.39842267755147
H	3.03923651526571	6.59882052541814	10.63311215784059
C	0.07649156579606	8.91530540605423	7.88703583667712
O	-0.20208613990632	7.95528290746107	7.18244681426525

O	-0.45824168136825	10.14740923294533	7.70567723610836
C	11.60970089106312	7.04459084102090	22.30373636960805
O	11.36922922085440	6.22194655379127	23.17617052195810
O	12.70820038099313	7.83830867228509	22.31606239236235
C	12.54614408347198	5.20118244634315	9.25229513055227
O	12.40616417865825	4.16298844562115	8.62113799848948
O	13.65486730216310	5.97594550062672	9.16786059642392
C	-0.93347005234460	10.29381938000589	20.89685662880168
O	-1.30354274180596	9.50071737215969	21.75114093598107
O	-1.50147266719498	11.51162625270992	20.72076336893860
C	-2.58564592356287	11.82762165430990	21.61112925738172
H	-2.24483272384452	11.81310549183852	22.66111801687026
H	-2.92346929030094	12.83579375115389	21.33093316054831
H	-3.40798296402942	11.09985684558067	21.49739940763778
C	-1.39153963027440	10.26725593430337	6.61825253244599
H	-0.90869779425062	10.00927444149753	5.65955584814400
H	-2.25474511860953	9.59587682545384	6.77008307106106
H	-1.71809446540659	11.31720628228939	6.61369514897606
C	14.67852249524970	5.50393719452782	8.27505548210826
H	15.04461372327007	4.51144441481288	8.59105859324051
H	14.29070629333533	5.42498316794607	7.24443488511582
H	15.48894113854547	6.24545108548455	8.32464180663558
C	13.59439490307242	7.65914094369643	23.43424254163323
H	13.07101456567606	7.86705574739716	24.38378807840573
H	13.98095230350179	6.62545965396504	23.46379029492085
H	14.41761301983279	8.37272264176916	23.28544142298395
H	5.52014741045617	6.64637059398768	15.25537582885882
Si	5.09663998828440	4.85478613918887	15.45351006549219
C	5.79378698471861	4.99779404858032	17.20883576511525
H	6.21486139414659	4.01297729478584	17.48922342427977
H	6.63788799099418	5.70851798002166	17.19893679089475
C	3.32377519872225	5.42697819295043	15.06823691894751
H	3.17879857288929	6.45079474668803	15.45049913853261
H	2.60463221197614	4.77956267362699	15.60552815066847

C	6.30394446622017	4.49617556734453	14.03831960229182
H	5.88508687490606	3.68738483641053	13.40925048830231
H	6.39380671780620	5.39013949706699	13.39833657832962
C	4.73316938671353	5.44972723091361	18.22323138549990
H	4.30731056638113	6.42826014388127	17.94280264853504
H	3.89545728798026	4.73100014030784	18.29387040803891
H	5.16017878559481	5.55891650559655	19.23717107962574
C	7.67535135819411	4.08882604037502	14.59701759082938
H	8.39167562739365	3.85291982851577	13.78853137223182
H	8.12154549119705	4.89387829878214	15.20483089969602
H	7.59691392817941	3.19175417129510	15.24018188115548
C	4.39546990433526	2.73327472519606	15.68475442585159
H	3.79726280229946	2.95252165387798	16.58336406717971
H	3.81958966751867	2.72419826440931	14.74629404468357
C	5.44909989592888	1.82683816496562	15.80789618177307
H	5.90575494617423	1.60558734723624	16.78051762242580
H	5.91590225186241	1.36934246189052	14.92719414798983
C	3.06185709645784	5.38421649729355	13.55407355709329
H	3.11041991763143	4.35350327914244	13.15570188936472
H	2.06420281272204	5.79106184223178	13.30720382868988
H	3.80483306441485	5.98758843685315	13.00493809208388

1-pentene

C	8.94266137423983	14.01663415781228	15.38926629994787
H	9.08591543438628	14.62007305020300	16.30137843484927
C	9.49960176405030	12.79255007102707	15.32716472680486
H	10.09561045380538	12.38668509394852	16.15432944272823
H	9.37953425342785	12.15426391867883	14.44013151987832
C	8.09988055877960	14.64794506675927	14.31374184445849
H	8.57207358181090	15.60024991267003	13.99059408104441
H	8.07419122691276	13.99074130923419	13.42186718470881
C	6.65894343322705	14.95276575573037	14.77562759307594
H	6.69582864517973	15.58935639010991	15.68250554910591

H	6.17657215976705	14.00546067973637	15.08767675786273
C	5.81543761478216	15.63908972128850	13.69454185795456
H	4.78880973758457	15.84105825707982	14.04956582593324
H	6.26246123585670	16.60473763916928	13.39197070189440
H	5.73914147618974	15.00997981655237	12.78785574975277

6-pentene

Rh	6.00340087645136	8.14129491293941	15.14717866641330
N	6.21304584840266	8.09620573745170	13.11013647386344
N	7.90697600870554	7.41324256071149	15.36845805511548
N	5.79012657195635	8.19195582183642	17.18377141112095
N	4.05477498358651	8.75561311437769	14.92588409070471
C	5.24650662999946	8.41663104441874	12.17146982915240
C	5.75409194671590	8.14685846398958	10.83734226334636
H	5.19151841945219	8.28405481685049	9.91239570286679
C	7.02811088991581	7.65763205494492	10.98356587694027
H	7.70866051237962	7.31846848600761	10.20102370416846
C	7.31395880427657	7.63530753608887	12.40774821374086
C	8.51562004620663	7.15933021592748	12.96917121069593
C	8.78124393710121	7.07819057848197	14.35064257945653
C	10.05775310841325	6.67710818764624	14.91685743815034
H	10.93610909159302	6.38391227626941	14.33944657445020
C	9.94089011895805	6.77827559197531	16.28113037147339
H	10.70467197794332	6.58070297657026	17.03505548221181
C	8.58828089476316	7.22910953839825	16.55896886973530
C	8.07019036893945	7.44671477766539	17.85029572519493
C	6.75423198504506	7.87113367237158	18.12388609861603
C	6.18474435974717	7.96152840553879	19.45744486263445
H	6.71529905820628	7.73361391144253	20.38334593207486
C	4.87485124563005	8.34591191618680	19.30919223251900
H	4.12733491226945	8.49654210084517	20.08988758079106
C	4.63702464935919	8.50159175246195	17.88514131678257
C	3.40206155831788	8.87825408235769	17.32342293080408
C	3.14866186928870	8.99640408771626	15.94386751106553

C	1.88285487548998	9.42968179381610	15.37929932131584
H	1.00131238568299	9.71133708656158	15.95730509054069
C	2.03530785938140	9.43980791724433	14.01486160864391
H	1.30179658607590	9.73393120644121	13.26295125904497
C	3.39220867221347	9.00174327728692	13.73597274595308
C	3.93800120544792	8.86269890780644	12.44379312090242
C	9.57957119283540	6.68944130186885	12.03346485680563
C	10.21741352631092	7.58953489643911	11.15037801918876
H	9.93059226811349	8.64790004444296	11.16650283511884
C	11.21437124561285	7.14517330669496	10.27270147123613
H	11.71081592155785	7.84816945324706	9.59556917031782
C	11.59144825163985	5.78512153265157	10.26072802331770
C	10.95783226970278	4.88115503707415	11.13924067920904
H	11.25822590125172	3.82717856035077	11.11356181010102
C	9.96433424981699	5.32899273263571	12.01682244282484
H	9.46578481534726	4.62495055591059	12.69351673825427
C	8.95527089231514	7.15732840476033	19.01712985683813
C	9.35785228477293	8.19429750572049	19.88862960836263
H	9.02685578417927	9.22012742179514	19.68666995469406
C	10.17770713774429	7.92444566472683	20.99173690065735
H	10.49210310734031	8.73282030295344	21.66014017392937
C	10.60919402169511	6.60502699675046	21.24622121306590
C	10.20939493108872	5.56502550276194	20.38084211596943
H	10.54701394985739	4.54462857188178	20.59621049334975
C	9.39249804271983	5.83840067140010	19.27823324727028
H	9.07283212126715	5.02720771800812	18.61354449363726
C	2.27353610722661	9.16425602769552	18.25919776859381
C	2.30473933519696	10.30076973217100	19.09752759320032
H	3.16652410228376	10.97740424028086	19.04897612003229
C	1.24615229846091	10.57251052974612	19.97377268060377
H	1.27216382916630	11.45869879872743	20.61652372627852
C	0.13353823687742	9.70592085682128	20.02839401993050
C	0.09807528080744	8.56829230007110	19.19501669291838
H	-0.77057014270447	7.90214590888770	19.25334108576271

C	1.15610393595364	8.30142784962005	18.31891148240715
H	1.13016957425880	7.41282391491866	17.67733920087804
C	3.07123816713879	9.21081119675426	11.28033805786690
C	3.41804386855434	10.29008294813593	10.43493264673961
H	4.32882382431532	10.86298016439375	10.64523615913544
C	2.60540844477931	10.63414059770539	9.34927062876491
H	2.86141539978059	11.47455440580010	8.69377338530759
C	1.42910955221274	9.90321647945964	9.08132881623941
C	1.08023324269978	8.81911582583160	9.91496397618916
H	0.17296700476135	8.24438525098577	9.70133538436695
C	1.89435188958859	8.47937210498768	11.00288729724016
H	1.62869656867894	7.63069730025807	11.64443969571058
C	0.59914729929658	10.31561754382999	7.91071071130384
O	0.86659281884426	11.24562534595329	7.16275720171850
O	-0.50096703860039	9.53796005488537	7.76210027022909
C	11.48060116052427	6.25491972147275	22.40712956493274
O	11.87924505233910	5.12692355198407	22.66112528987577
O	11.78834656631257	7.33693481138604	23.16331209274351
C	12.64676684318389	5.25306084139989	9.34825823870275
O	13.00371491468198	4.08405392049741	9.30584414472912
O	13.17751873729485	6.21879181753342	8.55880838018566
C	-1.02434637750776	9.94281946076223	20.94099949905554
O	-2.00695905765030	9.21884982646576	21.01900173864473
O	-0.86926611177686	11.06398841315592	21.68654470981978
C	-1.94627929324652	11.36499909187636	22.59044339228466
H	-1.65893935726824	12.29292641194519	23.10569017432584
H	-2.89095146902492	11.50811269721946	22.03724184157506
H	-2.08472739815126	10.54686174350389	23.31868097046461
C	-1.35451438730428	9.87138525276813	6.65384327080311
H	-2.18540316409538	9.15174683822609	6.68426753799725
H	-1.73442834311565	10.90345690413378	6.75111699386354
H	-0.80568131227590	9.78641428317089	5.69963601082412
C	14.20638383704573	5.78653961133061	7.65212601304128
H	14.52631158800571	6.68579884370893	7.10619111525739

H	15.05543608995570	5.34891883428051	8.20567048691469
H	13.81525522976430	5.02908285476655	6.95055716765126
C	12.62529807289104	7.08135013996305	24.30439252158518
H	13.59600076228102	6.65974979084545	23.98975839716649
H	12.77067990445041	8.05360528518790	24.79713251483648
H	12.13834384772792	6.36908373258005	24.99319920632412
H	5.40007150309218	6.39936909264943	14.92719430824255
Si	5.00256264103794	5.03726224599371	15.52066378234110
C	5.04254857911936	3.89086702517342	14.02254662090074
H	4.90658045442585	2.84550697812557	14.36682574956651
H	4.17067478582946	4.12203232626006	13.38061227631540
C	6.28642124685226	4.55596866493301	16.79742871099475
H	6.08804314671522	3.53965694273231	17.18490002955554
H	7.30054587204166	4.56978798981424	16.36119658952722
H	6.27582471499811	5.26078564629320	17.64627160038044
C	3.29667636671299	5.27160654277092	16.27814915544895
H	3.00855128732915	4.34307690247803	16.81117085833673
H	3.40355894508656	6.05073921248809	17.05881112978387
C	6.34827590803113	4.03797510715445	13.21746726174028
H	6.39086313185912	3.33802638808193	12.36263796396294
H	6.45397190650884	5.06140957830004	12.81509113995857
H	7.23798131853713	3.84664122220914	13.84563827285882
C	2.21606565893488	5.67421720444640	15.25936331860969
H	2.02834010295441	4.86757295964585	14.52743102749484
H	1.25112737944789	5.90243228247828	15.74918025385037
H	2.51381911358542	6.57291221699945	14.69097127179753
C	6.24186151240832	11.14265962396062	15.34700574798085
H	5.88024103997760	11.36253737887753	16.36399911410910
C	7.32219824620127	10.31821685396072	15.21135938443732
H	7.90743539348956	10.01295214285959	16.08582806871181
H	7.77809841509924	10.13868931702475	14.22862960473433
C	5.47679254041797	11.76037242426025	14.20816889634075
H	6.07431548374933	12.59572812363992	13.78169163067588
H	5.38144299943597	11.02077724621778	13.38832100435550

C	4.09145028619061	12.28750722407984	14.60903975214242
H	4.21824406890645	13.10446181988767	15.34785840840466
H	3.53816708231881	11.48643139895315	15.13237793746404
C	3.26755700144201	12.78192061430732	13.41762972528976
H	2.28417329470160	13.17049260635730	13.73747223586753
H	3.78872162322798	13.59169769337793	12.87203027831926
H	3.08301667162817	11.96275519241772	12.69883900983512

[**6**-pentene]$^+$

Rh	6.03198209991990	8.27167198242381	15.18671508986227
N	6.25134440111885	8.20198219320244	13.14505828333969
N	7.89275412888890	7.40919726960875	15.40948670160434
N	5.77340237736376	8.24614875284179	17.22104131252531
N	4.04965511346024	8.80634897435654	14.95613139057354
C	5.24700736610651	8.37613386218880	12.20563541072239
C	5.77704521002493	8.10788024691786	10.88306723452227
H	5.19838875580550	8.14305900461933	9.95904015608895
C	7.10089799921336	7.77945976856019	11.03585898843525
H	7.81366530937586	7.48889554394844	10.26277876741119
C	7.38815035836262	7.81698728805527	12.45660381248406
C	8.60174452942697	7.37551321668762	13.02138108446799
C	8.81193872222921	7.18147919255627	14.40012484849353
C	10.05831559552413	6.73073304835543	14.98487835725050
H	10.96039444188796	6.48732539545743	14.42160970484488
C	9.87956515637122	6.69930752287623	16.34686797258730
H	10.60799767579314	6.42605087816621	17.11165684432818
C	8.52417423016435	7.13704263459387	16.61090984601242
C	7.97735060167267	7.29560642998161	17.89898669748749
C	6.68882970743696	7.80260070596614	18.16118204968952
C	6.10114194458207	7.89772161143265	19.48291618235528
H	6.58726678852176	7.58065529063857	20.40670240705647
C	4.84051269593543	8.42044616761920	19.32947922442726
H	4.09485505268451	8.61185184846646	20.10259061068287
C	4.63796714360279	8.64032078544678	17.91125818283266

C	3.43050477384269	9.09986316199865	17.34862252901869
C	3.17027193517162	9.15561865529999	15.96610850924740
C	1.91461110473339	9.57517413951663	15.37847573618886
H	1.05016240539328	9.93241305501340	15.93952505591545
C	2.04297178774583	9.44896288711708	14.01577610362285
H	1.30452306984517	9.68607992066504	13.24895431467614
C	3.38327489127168	8.96329996649012	13.75525980369020
C	3.90868066112235	8.72876411852459	12.46768058091550
C	9.70969813122423	7.02688977724371	12.08506020048794
C	10.31105154483825	8.02469614740016	11.28607309192663
H	9.96962911329756	9.06318923025317	11.37477786140520
C	11.34203839711231	7.69777792488239	10.39571020480538
H	11.81230829700162	8.47345391244495	9.78247853719337
C	11.78393295984651	6.36266052075631	10.28661430959026
C	11.18327677710289	5.36251817399162	11.07873464544189
H	11.53458569933652	4.32950422228747	10.97348733662099
C	10.15667802628885	5.69083366054381	11.97101128750480
H	9.68027125549884	4.90977877253265	12.57556778226575
C	8.81292308506861	6.87881196747208	19.06311166730102
C	9.24886543857786	7.82719425548733	20.01520708403957
H	8.97979496883489	8.88252930433271	19.88601346268990
C	10.03220776878310	7.43210978360839	21.10737726898419
H	10.37602767480605	8.17044950879748	21.83917088553832
C	10.38914085975700	6.07680441350287	21.26718719846038
C	9.95523941477202	5.12615505705547	20.32054093007273
H	10.23815702678606	4.07686822916469	20.46379452337598
C	9.17688952077837	5.52274940530088	19.22747733371659
H	8.83447201451770	4.78023405993893	18.49684659502329
C	2.33588065163617	9.49294040194406	18.28349128255544
C	2.48326766240101	10.61859536934046	19.12388230203392
H	3.40511317147481	11.21056611623807	19.07341884899049
C	1.45891257545588	10.98697618250947	20.00585059757391
H	1.57144334489396	11.86418824471002	20.65132886658967
C	0.27057263627283	10.22922561097628	20.06387582117049

C	0.12105440264143	9.10203418736132	19.22984513058098
H	-0.80664947657784	8.52188454984785	19.29491482896730
C	1.14304171604315	8.73760462038299	18.34650872128072
H	1.03081189264475	7.85494564366523	17.70586128565603
C	2.98231255568494	8.88593176603479	11.30971635731729
C	3.20986931938339	9.88147330923453	10.33218843548686
H	4.07886165040602	10.54320433713637	10.42797133744814
C	2.32531976880805	10.03496142095048	9.25875617070112
H	2.48489063811041	10.81029917124579	8.50055066304714
C	1.20101343557408	9.19290011408693	9.13741956728060
C	0.97170094829729	8.19280981034586	10.10551616467301
H	0.10230738777944	7.53482305843674	10.00517327714302
C	1.85415085678939	8.04430061425065	11.18298852369295
H	1.67949123085917	7.26265590202967	11.93212069766847
C	0.29038993698358	9.40523537671216	7.96858747829625
O	0.45877308003203	10.26125659059024	7.11310342837307
O	-0.74575153699059	8.53721923502663	7.96533874197739
C	11.22253433444869	5.59365037541764	22.41274814044311
O	11.55396718302833	4.42917666379457	22.57847602870479
O	11.56967627828096	6.59702035226185	23.24926239850781
C	12.87764361428161	5.95121742703048	9.35140689841312
O	13.28837771801617	4.80616478009670	9.23559632934313
O	13.36271722879361	6.99556461910425	8.64350853733684
C	-0.85807624064435	10.57168780299441	20.98547033708964
O	-1.90191889566974	9.94042990371210	21.05770932133620
O	-0.59132242110581	11.66515857977135	21.73376203345635
C	-1.62943768161778	12.06792035118954	22.64757160621050
H	-1.24480454972940	12.95614346549877	23.16858893308897
H	-2.55518993821140	12.31261428428798	22.09872880191803
H	-1.84672440762316	11.26069084453740	23.36813761160692
C	-1.67358576272753	8.67346246597750	6.87176416762561
H	-2.43724071792386	7.89783711550571	7.02534332903487
H	-2.13435993847947	9.67631088479284	6.87754532196537
H	-1.15949158824810	8.52450114349659	5.90662185589674

C	14.42199214650619	6.68667387707481	7.71750916708285
H	14.68823621815018	7.63783619214413	7.23505855371759
H	15.29231410419927	6.26660237087146	8.25058423197627
H	14.07869313535994	5.95467061716500	6.96627475984042
C	12.37655828185514	6.21850891752856	24.38099152387455
H	13.33193374122533	5.77912813024252	24.04586694697229
H	12.55595567417322	7.14372420574631	24.94679542379500
H	11.84554962772238	5.47972799577501	25.00565236061164
H	5.44403692459794	6.69778241549276	14.90735326074577
Si	4.94661895036914	5.29450098708118	15.40637204353713
C	4.82841533709173	4.51930532702289	13.70358746386751
H	4.34549071455943	3.52796580610364	13.82939981309177
H	4.12410883072608	5.12935471403651	13.10603537157318
C	6.28239242488511	4.58858572723553	16.49004570818837
H	6.08192215868276	3.51103934681278	16.63988472376160
H	7.27863760188086	4.69495394975475	16.03007627920902
H	6.29348214527512	5.07854036824824	17.47801862671946
C	3.31907604997706	5.55779966853795	16.28400870480683
H	3.13722699617279	4.62790408557009	16.86181437567796
H	3.46513081466858	6.34943479339460	17.04310873801914
C	6.18216146768739	4.38624644047501	12.98485205427165
H	6.06028772329833	3.95585396293162	11.97517071293748
H	6.67261351377717	5.36780348648513	12.86618652619050
H	6.87780230493510	3.73379848944536	13.54184951167397
C	2.12243926364935	5.86871913853579	15.36984211751720
H	1.91326466036720	5.03265428617096	14.67925087322312
H	1.20440128778922	6.04577611749978	15.95812479786293
H	2.29761195613105	6.76831351748999	14.75638226379292
C	6.13758127541669	10.58823013526544	15.30917170811842
H	5.59653863360892	10.75927337305256	16.25216248113784
C	7.39659735534623	9.99361994081282	15.39504535898354
H	7.86619551607167	9.82817612292510	16.37131166257899
H	8.07089785965251	10.00273154746158	14.52870990854297
C	5.63605257058849	11.34888776736416	14.10790250467020

H	6.45011263439912	12.02901756057615	13.78235654393850
H	5.48757080605649	10.66544032682107	13.25238756660353
C	4.35497542341526	12.15313251042729	14.37231535968510
H	4.58211363533626	12.97436369945209	15.07998559933626
H	3.61594540821029	11.50876965238984	14.88087464628921
C	3.73735712532153	12.71524364889241	13.08966581463650
H	2.84135055000445	13.32275113725173	13.30614662696557
H	4.45255205169004	13.35535953536497	12.54017711583297
H	3.43058085686516	11.89635680794983	12.41304023784816

6-silane-TS-MOF

O	6.64332001133424	8.55736604333311	12.11972601321004
O	-6.64332000050271	8.55736601295085	12.11972600115762
O	6.64331973893925	12.11972590747428	29.95463426695757
O	6.64331998259040	26.39227401206998	29.95463405666830
O	6.64332020404917	8.55736587915013	26.39227393408865
N	2.03180048474998	19.08185121968790	28.70023726363936
C	6.42640366624256	12.72384506699411	28.84415645488564
C	5.26619168880536	13.63806142806443	28.77029302820965
C	4.45232409229888	13.84881602704166	29.89861259130537
H	4.66275398723650	13.29423738637199	30.81953502581560
C	3.42194548640518	14.78742331609826	29.82797670408975
H	2.79266665949636	15.00110842608482	30.69988098671400
C	3.22073067958109	15.51455319277772	28.63669106495574
C	2.23997583834455	16.62085219429839	28.63040045342061
C	2.76859835089301	17.91894276907540	28.63653181644727
C	4.18640218759599	18.23158045363368	28.63833424192213
H	4.99228002995807	17.49698423691086	28.61733733156782
C	6.50612433531886	25.68136306742370	28.97614425987914
C	5.60792788615087	24.50685013253566	28.92141819588530
C	4.87422941119223	24.21530334054030	30.08648660096709
H	5.04659412531420	24.82458934181059	30.98070906063995
C	3.92967832555860	23.19153831232630	30.06307198955320
H	3.32490297700131	22.96307436346282	30.94840674349807

C	3.71996221518476	22.46154417873060	28.87503748207389
C	2.61589972986373	21.47897266362029	28.82589885269630
C	2.93863937906856	20.12099775347646	28.73612514382401
C	4.29108863676316	19.59864465966074	28.70216526160074
H	5.19748750802622	20.20507222504749	28.74051737504907
O	7.13627446366233	12.61267999605534	27.81336555730997
N	-0.15406619584977	17.20017643593236	28.79641751581890
C	5.03120322845083	14.32043716985656	27.56024599607544
H	5.67864363426846	14.11107769164519	26.70202731706890
C	4.00594569451749	15.26198740983224	27.49465690929551
H	3.81858779253842	15.82728944570565	26.57467946197295
C	0.88158968369896	16.29707450755923	28.69153114535756
C	0.35831863404602	14.94406634868855	28.66036752125936
H	0.96314051496906	14.04166399732479	28.55530952498127
O	7.13627402553741	25.89931998559263	27.81336594123346
N	0.15320895511910	21.26584154000626	28.91619950456767
C	5.44063050144735	23.74771159384122	27.74736691922732
H	6.02928003237846	23.99089207133670	26.85708636967155
C	4.49505634742496	22.71960962749917	27.72759308148592
H	4.31706809512594	22.13692172106205	26.81581552118692
C	1.31722751131378	22.00342921672420	28.88932449232316
C	1.00379975894455	23.42112725130891	28.91308074476792
H	1.73763576338873	24.22743310254862	28.88231174538212
O	-7.13627417898341	12.61268005377110	27.81336600539828
C	-6.53442645054466	12.82529461494535	28.89601875230495
C	-5.60686311029514	13.97567208247403	28.96591175918944
C	-5.44817381859560	14.79830465398469	27.83478097088233
H	-6.05184763104717	14.59645072398115	26.94310554056210
C	-4.50172815952250	15.82178940529560	27.86558778511542
H	-4.33039707034601	16.45874000221362	26.98983347689027
C	-3.71278839074585	16.00579137081154	29.01894999924576
C	-2.60717316262035	16.98689375146957	28.98537772365645
C	-1.31400784424254	16.46125209565541	28.86968595822364
C	-1.00594711870364	15.04531486665990	28.77117467972912

H	-1.74297711284502	14.24092596443218	28.77205629261847
O	-6.64331993679057	12.11972599105414	29.95463394833777
N	-2.03072254214834	19.38660865735456	28.93672701346538
C	-4.86156906338344	14.19201169404253	30.14028466558655
H	-5.03064845261706	13.53823614827531	31.00255913813087
C	-3.90795078986716	15.20965193138562	30.16454950535810
H	-3.28906822042299	15.38363668480202	31.05270031151096
C	-2.93277240119734	18.34623262983361	29.00655269625244
C	-4.28351669864577	18.86607715437036	29.10134032881510
H	-5.18338743908183	18.25546420615379	29.19334331418050
O	-7.13627401967535	25.89931999081159	27.81336596739215
C	-6.43009825786972	25.75632906206799	28.79425119324754
C	-5.26676511892968	24.84454653010197	28.85461216049627
C	-4.99910626394878	24.08453588492456	27.70085736850506
H	-5.62118548399519	24.24298199537108	26.81291942003736
C	-3.98095057716355	23.13428297476278	27.72831874510272
H	-3.77122065814262	22.50853415057982	26.85305895414936
C	-3.23147619792342	22.94880486014801	28.90797508430578
C	-2.24563691084361	21.84832625482363	28.95216797954304
C	-0.88587501834129	22.17245493603948	28.95595759514952
C	-0.36435798171849	23.52482954495080	28.95154948528065
H	-0.97376788447993	24.42997921872665	28.95776220541250
O	-6.64331998318618	26.39227402292692	29.95463403062439
C	-4.49306907121528	24.69481083747201	30.02144208924355
H	-4.72046823659268	25.30218237383919	30.90327439138585
C	-3.46851170921648	23.74527699156838	30.04456821083041
H	-2.87181568098955	23.58181918818359	30.94979230354508
C	-2.77069360629140	20.54905960509198	28.96162110366211
C	-4.18440010689165	20.23447226011711	29.06401071913735
H	-4.98741053232390	20.97016872441077	29.12688386632191
O	7.13627359059424	10.69863420732181	25.89932022454693
O	-6.64332013564517	8.55736611829579	26.39227390407535
O	-7.13627400246662	10.69863398985309	12.61267999664717
Rh	0.00207830697123	9.66872987569570	19.26410771658563

N	2.02037751920211	9.58519568255153	19.43159741127922
C	6.54676853246188	9.66533964363722	12.87394505804664
C	5.63023452265908	9.50058428530813	14.02023533951299
C	4.84856716229035	8.34208946052008	14.19365928183008
H	4.97111577454919	7.49838273275343	13.50678235943067
C	3.89842584515224	8.30747361172353	15.21686036928091
H	3.24885944198199	7.43452552733168	15.35137703663110
C	3.73820459473016	9.42036995412516	16.06666953143562
C	2.62546607045527	9.46448759353708	17.04071508100998
C	2.93798601134993	9.45622617064741	18.40340058732530
C	4.28186237241429	9.34347846369252	18.93706031652699
H	5.18460803365599	9.20380255600108	18.33926808163535
C	6.41821951373162	9.66694784337045	25.78981824575202
C	5.26149063962160	9.71635514022250	24.87855728392913
C	4.41081816058515	8.59861293032764	24.76397144894523
H	4.60110130886270	7.71831502085148	25.38744468447653
C	3.37560511850731	8.62137309090755	23.82955297753775
H	2.71841179740946	7.75454852346578	23.69344427157011
C	3.20805190799681	9.74766781710844	22.99737144329832
C	2.22828444856679	9.70891736699618	21.89069590464851
C	2.76108346759330	9.59587726657843	20.59975305144440
C	4.17397912977545	9.43828152954686	20.30219894094827
H	4.97277921823940	9.38632331940302	21.04401188100200
O	7.13627398877378	10.69863397501728	12.61267999195933
N	0.17039255225006	9.67272340065418	17.24465793837938
C	5.51029372768585	10.59421673027028	14.89973774840946
H	6.13792170660309	11.47586048208368	14.72799426712323
C	4.57220115997246	10.54918604212336	15.92875009197500
H	4.43435205548810	11.40146226747746	16.60445146679368
C	1.33780916043909	9.60320032534397	16.50507958479430
C	1.04428066173363	9.73436137013985	15.08855495124593
H	1.78778611903363	9.74448198789269	14.28993368217539
N	-0.16403452370469	9.68270626762368	21.28537274236468
C	5.05766738609565	10.87097139464320	24.09640017241163

H	5.73186283497080	11.72455113765591	24.22579350426141
C	4.03311965382905	10.88158554378558	23.15206981044441
H	3.87399986897193	11.74917941775127	22.50113939310919
C	0.86738946609218	9.74618740548142	22.20645951533306
C	0.33718022531530	9.83763150185196	23.55267395164249
H	0.93828599435012	9.92867321944996	24.45959901127627
C	-6.38144964438428	9.59058379322803	12.70932312336642
C	-5.23602098813246	9.76174235951047	13.62625461310067
C	-5.05030691510746	10.93922891064949	14.37659955030190
H	-5.72717321520138	11.78698558709736	14.22951107033058
C	-4.02705606886047	10.98985839163937	15.32554674998844
H	-3.88237015673741	11.88041438703958	15.94850465969591
C	-3.18955376151700	9.87269617514956	15.51918396217571
C	-2.21710970567831	9.84983922806465	16.63402525987066
C	-0.85434732976523	9.80342305640696	16.32315892428227
C	-0.31740624522909	9.86305588055997	14.97695208621730
H	-0.91052590703052	9.99529040818133	14.07006698950632
N	-2.01800425391905	9.74022962779431	19.09603583677578
C	-4.36809729787346	8.66285829081196	13.77757220350906
H	-4.54723104416394	7.76621462086596	13.17357846863063
C	-3.34030894438740	8.72241985687795	14.71669764794259
H	-2.67526431112124	7.86614117293116	14.87928559432137
C	-2.75620133374902	9.78418780018101	17.92659307259821
C	-4.17545373605484	9.70460010707022	18.22503644182524
H	-4.97516027479513	9.68978977412990	17.48282839094827
O	-7.13627375352258	10.69863383683767	25.89932014147364
C	-6.52640230296157	9.61964720924582	25.67544771560806
C	-5.60362785719662	9.56920853614841	24.52867021364510
C	-5.51035059508924	10.69120348214005	23.67921484225219
H	-6.15268177911278	11.55731650808830	23.87403526692286
C	-4.57990592697927	10.68678211359884	22.64122431619324
H	-4.46092119242338	11.55768666738525	21.98614795002693
C	-3.73119035039315	9.57359600694750	22.46601294828504
C	-2.62633181495841	9.63098158396378	21.48666395363099

C	-1.33236602822488	9.68522819810060	22.02444849340381
C	-1.03073169945037	9.79138455741913	23.44149070491574
H	-1.77091542451861	9.84441896783570	24.24155919445588
C	-4.80847274808674	8.42614199837850	24.31573401398615
H	-4.92645327011306	7.56359418413462	24.98026788167347
C	-3.86896291286329	8.43326993568300	23.28621155197717
H	-3.21183889340050	7.57255118759850	23.11623261903638
C	-2.94151996979553	9.66202951789327	20.12465358463539
C	-4.28932925599588	9.62092448603557	19.59024240351773
H	-5.19984997959866	9.52835557712224	20.18534486622714
C	-7.77224539831236	27.28455688123001	29.98758423914941
H	-7.65952420826344	28.08131797722745	29.23212428194878
H	-8.70713290560597	26.73415319560645	29.78415695209229
H	-7.79001420571028	27.71306701195771	30.99981521308026
C	8.01543160757239	27.03828774886862	27.77507246991779
H	8.44184516600700	27.05763224320000	26.76199491373579
H	7.45481414219461	27.96751486022132	27.97667144562263
H	8.81486165017608	26.93683244289680	28.52926926109880
C	-8.28927577904270	10.60149355325293	11.76026870600416
H	-7.99015861269625	10.36166148936135	10.72491492186200
H	-8.97450315750299	9.81368941881223	12.11930595450288
H	-8.78002171341424	11.58472014981576	11.80032991427647
C	7.49335310669607	8.65465952738225	10.96490413351936
H	7.45310821195058	7.67111418801718	10.47486313753878
H	7.13221343019508	9.44178754988250	10.28000890696906
H	8.52903094141825	8.89546972133518	11.26202218151876
Rh	-0.00037451847725	19.23349581816920	28.82242244889670
H	-0.09796089811729	19.33187596824543	27.24515012781805
Si	-0.36665586436795	19.34049914383080	25.35302634478889
C	1.51340338960331	19.49716481350914	25.31289523297357
H	1.78733023597133	20.10834195652835	24.43209518050727
H	1.83446206764527	20.06701931960666	26.20266315881334
C	-1.19286044025271	17.67935149425466	25.62184216887700
H	-1.59186907004966	17.25256372239787	24.68688660807439

H	-0.47905057687862	16.96185055354571	26.05983537883526
H	-2.02281186711386	17.79942827541846	26.33877155809241
C	-1.38842870864727	20.90448640589684	25.61370483732397
H	-2.18860176873607	20.94503462197716	24.85083832897140
H	-1.89750504267681	20.79545022396176	26.58769849518480
C	2.20780260973288	18.12998847066922	25.27185010947552
H	3.30744686147699	18.23865286359942	25.27028639468431
H	1.93780656392616	17.51922783401178	26.14930994758681
H	1.92850808065945	17.55301314632086	24.37026859310854
C	-0.53832727736330	22.18359397751528	25.59115197233653
H	-0.04950541096043	22.33762209103169	24.61164029233105
H	-1.15664312597605	23.07554331028376	25.79764700698174
H	0.25403081069440	22.15298127233992	26.35826047451267
C	-0.66284291953378	19.43378183419287	23.07589557371704
H	-1.74633170248717	19.26377530967765	23.15645182358374
H	-0.34788100344698	20.48270170648835	22.97509011555637
C	0.13859635310913	18.43394465727637	22.56185399621536
H	1.17921240616038	18.62520399319967	22.27328570409055
Zr	-10.13631740002556	8.20169227236548	30.89770071479168
O	-12.25696705734936	7.82221627054720	30.51888157199333
O	-8.68699941939549	7.81181166369227	32.75189806046264
O	-9.13628806523140	10.12696301894912	31.23073947065814
O	-7.99548200704172	8.04343695299945	30.13119788196417
C	-12.97224725127829	7.36773092264258	29.56968958892795
O	-12.76728071637593	7.52779142851006	28.33506410574893
Zr	-8.11306388665829	8.07918261718168	27.88553450472319
O	-6.82410777519484	6.54767558416439	28.37076811455390
O	-10.10419291769607	7.55958804006353	28.68271529063376
Zr	-7.22911734843316	10.16818061055515	30.62675728543055
O	-5.27618386883243	9.66113546991777	29.56093054882248
O	-7.97124722673517	10.00114721866251	28.66959136922054
Zr	-11.43691334175732	9.06024099866987	27.36335517885719
O	-13.24209622547257	10.12572555026629	28.31601979788487
O	-10.03665524163535	6.22068209412273	31.05967918477429

O	-10.66420692168166	9.87072195323885	29.37752729402324
O	-4.94330814753415	7.25164674403770	30.15807594381750
O	-6.37953876084872	8.79467949606131	31.99550685571024
O	-12.54450713055890	9.39793744206882	25.74715600427512
Zr	-10.55198263981033	11.73673670115307	30.81397838911717
Zr	-8.89237843709334	11.35614584438260	27.16117009544642
O	-7.03665076980691	11.43358721644792	32.44505265639465
O	-8.88206167581345	6.64161767239953	26.37921156360747
O	-11.16162248035770	8.85460591149833	32.72150664272509
O	-11.06328599592880	7.20723533577822	26.08760798524938
O	-9.42861394189022	9.33938289196297	26.74892353743623
O	-9.51151994571652	12.33691543612943	29.03659912000556
O	-11.06660545738757	11.23087695699417	27.09059878101398
C	-11.43595285900004	9.94968086633714	33.31304280546000
O	-11.26873878772360	11.10850326850758	32.85004085536365
O	-9.46076900266961	10.03743186794797	24.03961577949110
O	-9.10482898604223	12.51103563648786	25.57677295936253
O	-8.90797283720868	12.68431155745773	32.16405372776370
C	-7.84769344588215	12.36249091122420	32.76516936057687
O	-11.04312774590384	13.65797791560986	31.03101493520779
O	-12.51726920079718	11.47616439349725	30.33542991138294
C	-10.02308604257722	6.54325508599160	25.81983920555101
H	-11.39798438213998	11.49143962253753	26.21207621995247
H	-7.59874013438380	7.20311060753394	30.42584455626755
H	-9.48845599775164	13.31055194139498	29.00665072660864
H	-10.32726456083044	6.61068023207839	28.63365756600017
H	-9.17006971975994	12.00110843528341	24.73736254934393
H	-10.44058299128296	14.18654889661371	31.58344141366698
H	-6.51970067018374	5.91833120516809	27.69485903257089
H	-9.47839813026016	5.82626806882459	31.75114794813876
H	-5.86282009997780	9.19987939195255	32.71547854036105
H	-9.02859567112782	8.32684897884396	33.50456241455997
H	-7.74920999644698	8.17347994435244	32.56409754098181
H	-5.18583915104674	9.87423344822012	28.61535183364062

H	-4.99459826396496	8.65794625303125	29.71077389146870
H	-5.42727717298608	7.48909714904132	30.98907064424940
H	-5.62433245974218	6.85347122578931	29.53273673169216
H	-12.60128798407944	8.69031378111544	25.08214265696234
H	-9.47189438922631	9.60018139325060	24.92676519328236
H	-8.59678682240588	9.78995265182070	23.66922068805329
H	-13.13242628152005	12.15382018993706	30.66484165743991
H	-9.69245156489319	9.96962373492160	29.17493840397656
H	-12.96991573963216	10.68160170718219	29.14488368170228
H	-13.50207860954546	10.78108270172214	27.64403658265563
H	-13.86514923572076	6.77269806237332	29.86490688795085
H	-10.10878263208986	5.77810435012632	25.01495121199071
H	-11.86144509190248	9.86081938957498	34.33739925748520
H	-7.57700598444354	12.95055236379401	33.67307096370404
Zr	10.23634012256020	7.66593128293210	30.27172583329337
O	12.34950929839545	8.07795811001440	30.64715854542595
O	8.82256975520968	5.79214139549834	30.69039966397435
O	9.22018059905553	7.32016063178363	28.36833176543826
O	8.09158320852129	8.40681057593129	30.44615241291806
C	13.07282283721617	9.04156786214612	31.04935166326306
O	12.86184992491801	10.27064674263922	30.84915269095817
Zr	8.17383829690454	10.65151254158894	30.40495670348533
O	6.92998265766788	10.14456997854380	31.96721505661073
O	10.18200561897814	9.89056397462204	30.91063133908328
Zr	7.30234041258837	7.88759406892234	28.32322056994929
O	5.30960374251675	8.85941904760423	28.88274286550622
O	7.96800988100995	9.87806859226403	28.49350736856659
Zr	11.47726384183559	11.23053103542522	29.38098002552319
O	13.26440612087174	10.32246224791275	28.23326825676150
O	10.14744900340373	7.51092406410138	32.25508669203668
O	10.70347852850833	9.21361924833048	28.58477745195923
O	5.08704251339614	8.28330634517411	31.31691949431110
O	6.51425338703171	6.49056018693505	29.70306667288056
O	12.48523001986489	12.90144848779324	29.01526776327254

Zr	10.64935993722817	7.74035067830250	26.76673118687170
Zr	8.90403728728515	11.35796808565604	27.11420720385347
O	7.16041423497910	6.08829969414991	27.03668384575982
O	8.92369478335270	12.19443435356200	31.81727671457263
O	11.27925179880709	5.86845912823276	29.59254737965201
O	11.10535736087602	12.48817986796516	31.25205079872600
O	9.45899863163412	11.79355839960540	29.12162790560520
O	9.53922422629846	9.48138394467298	26.14987388016483
O	11.08255117752615	11.47123371586242	27.20165607395954
C	11.47421887421430	5.22298920037773	28.50884795645863
O	11.25089390499896	5.63888875542953	27.34266022857530
O	9.35915320777024	14.51105665943360	28.46598473273730
O	9.08207736637538	12.96650901695154	25.98827136899889
O	8.99179735952604	6.47947242669631	25.75944519303149
C	7.95845463295495	5.82988393171793	26.07764254538986
O	11.26869222839725	7.49865623353177	24.89694115703671
O	12.60994150302554	8.08036842941321	27.23563618230086
C	10.06471712103261	12.75426694129917	31.91542524450632
H	11.35620153857051	12.36844139591274	26.93529299316984
H	7.71986311182805	8.11896532778778	31.30003030364959
H	9.13700325771778	9.35065972236388	25.27270114790818
H	10.41243521999186	9.94018582801629	31.85774887494667
H	9.14910999026196	13.79986963093955	26.50799862314155
H	10.72238734358262	6.98148830419165	24.28047389554839
H	6.63496176580832	10.80639547920538	32.61520456461695
H	9.60397436015102	6.80592460311952	32.64652798386814
H	5.99940166956129	5.76267865152745	29.30977033131681
H	9.17264900148263	5.03227739446103	30.19240762724822
H	7.88113646776891	5.96026268074432	30.32241352844031
H	5.12857283728276	9.78605810650837	28.64920222751981
H	5.07322800951209	8.71475557101127	29.89428170061041
H	5.58369901311701	7.46534154959992	31.06248204901389
H	5.76071076662941	8.93023123545869	31.68908087207533
H	12.53531065201397	13.56920905329853	29.72054225756889

H	9.43203131673831	13.61082719351670	28.87092944120461
H	8.41598913922814	14.72170679940266	28.56829466869939
H	13.27162603162888	7.64833288081034	26.66823156309801
H	9.72616600136297	9.38144067818455	28.50329050806286
H	13.04546870689558	9.37682837257289	27.87628159646986
H	13.23316231677345	10.86255300391897	27.42030075319231
H	13.98078127116238	8.77110392010611	31.63286969684428
H	10.14978060114945	13.55887532852440	32.68134510426978
H	11.88028432667274	4.19265011769980	28.61986819088596
H	7.70266304605783	4.93877796902874	25.45935619432255
H	0.08506720916362	19.00091947906009	30.61807949883764
Si	0.32837054257215	19.29423765321595	32.09563397270746
C	1.41566735888739	17.86950775412621	32.66421333903944
H	1.35203687114938	17.82044190248534	33.77032307038129
H	0.95003434822359	16.93479237734675	32.29320908163746
C	1.15745821604752	20.96587967122421	32.19888933367950
H	2.12994895440714	20.94502017873358	31.67791830342906
H	0.53506642200743	21.74457956231623	31.72540555376089
H	1.33297205073813	21.24975622585175	33.25274010566145
C	-1.38804531818654	19.24410817022245	32.85835580419891
H	-1.86577160369520	18.30089104724903	32.52758394761985
H	-1.26674089527144	19.15491250165357	33.95715309251167
C	2.88282789976260	17.96167366673307	32.21051539796115
H	3.46728691967066	17.08299357438664	32.53923628086005
H	2.96372966474837	18.01496495590260	31.11175023630067
H	3.37900221998167	18.85987196718541	32.61942783367646
C	-2.27575532989113	20.45051609921047	32.50637210958303
H	-2.40135339345972	20.55216795557778	31.41519939577439
H	-3.28501823851375	20.35659202798737	32.94638210248393
H	-1.83904552292626	21.39598954768724	32.87530752916946
H	-0.21667087717012	17.39949610223641	22.47609802050654

13-TS-MOF (but with 1-pentene instead of ethylene)

O	6.64332001085071	8.55736603533582	12.11972600911177

O	-6.64332000001680	8.55736600977905	12.11972600004999
O	6.64331986047245	12.11972591645613	29.95463422817308
O	6.64331996917532	26.39227402163869	29.95463410888584
O	6.64332007336519	8.55736591316483	26.39227395651056
N	2.03018524566451	19.05969543900138	28.63748049685501
C	6.42057940147665	12.71449051860762	28.84109390681048
C	5.24455691458295	13.60803909913101	28.76702225846188
C	4.41663260200224	13.78865677946401	29.89036005236690
H	4.62459612757894	13.22071791903947	30.80356548392939
C	3.37864507939376	14.71903368445256	29.82939196144644
H	2.74192112400771	14.90674694787230	30.70160163660028
C	3.18481079090857	15.47729323393542	28.65474212968451
C	2.21610882242754	16.59341387730979	28.67221044838662
C	2.75302087321298	17.88845818586551	28.57899937383033
C	4.16948014603075	18.19244525059719	28.47967990552426
H	4.97069383438069	17.45412366424846	28.44001058592169
C	6.50447386631646	25.68068410784571	28.97565073156684
C	5.60916649918638	24.50178064358270	28.89510201623047
C	4.85240595710537	24.17715987763220	30.03601110819304
H	4.99739962051311	24.76516180380031	30.94942566388946
C	3.91750950946054	23.14445443897070	29.96667836171842
H	3.29696664342759	22.88969125098732	30.83395084714586
C	3.73637417144225	22.43865640888344	28.75799479678226
C	2.63106493155471	21.45684290015653	28.67465103611178
C	2.94209152330986	20.09509511880437	28.58639938681613
C	4.28614887531048	19.56041762611533	28.48848203173289
H	5.19764646695703	20.15935747444216	28.45578691197338
O	7.13627423344803	12.61268003507523	27.81336566831930
N	-0.16256713138504	17.20386666672089	28.99934214901811
C	5.00851516839231	14.30595101701704	27.56715260155850
H	5.66113764323992	14.11624721369904	26.70820076748820
C	3.97933133335505	15.24319435330388	27.51353188877865
H	3.79972174729266	15.82055794098441	26.60110853904151
C	0.86167761664776	16.28823789925424	28.86189505099404

C	0.32595653823393	14.94148336695038	28.89848022085433
H	0.91517814538038	14.02937791222751	28.79141276249166
O	7.13627403637332	25.89931997380708	27.81336588910666
N	0.16430809773519	21.26228445309877	28.76072578155828
C	5.47096340452906	23.76448499328518	27.70189942023352
H	6.07597923683522	24.03143415695181	26.82998240735054
C	4.53428389512922	22.73022463305470	27.63520498950215
H	4.38038434378028	22.17216142349421	26.70387133672153
C	1.33342415462445	21.99064852422323	28.71870094754474
C	1.02826761094452	23.40962036310777	28.67158875061959
H	1.76883471383631	24.20815705089141	28.60885631993186
O	-7.13627420074678	12.61268019282818	27.81336599325511
C	-6.54180779754943	12.83112426849024	28.89951252686314
C	-5.62748386916351	13.99242171568942	28.99611858191455
C	-5.44015540852238	14.82843921604834	27.88003381075414
H	-6.01492490913719	14.63379557526516	26.96759199433432
C	-4.49947770955149	15.85612415009863	27.95034867496867
H	-4.30849265132183	16.49954373668001	27.08352289604212
C	-3.74118989702430	16.03542900043733	29.12727107051938
C	-2.62945517808864	17.01118289132409	29.12130458667630
C	-1.33057956365933	16.47745689018353	29.10940404884108
C	-1.03252238873596	15.05782456000134	29.05815668685582
H	-1.77306244259761	14.25816926597820	29.10011970512603
O	-6.64331991688649	12.11972589329534	29.95463393553034
N	-2.04253473678926	19.39821171688036	28.88613995371906
C	-4.91847809679576	14.20621387103452	30.19439393184847
H	-5.10986360014332	13.54521949047835	31.04617999716113
C	-3.97171256982442	15.22817506799337	30.25881577351705
H	-3.38476161319277	15.39632201786433	31.16950594744201
C	-2.94945429340518	18.36886797867747	29.00123309227001
C	-4.29888933785970	18.90189107716773	29.02318946029380
H	-5.20631041697125	18.30378908057300	29.12179926326368
O	-7.13627402302773	25.89931998929027	27.81336595823270
C	-6.42233127516318	25.76624668555541	28.78934156335496

C	-5.24474328964976	24.86986972047973	28.81490348604570
C	-4.99596141451306	24.14122534312587	27.63556560635812
H	-5.63756445982131	24.32125724409431	26.76605525111439
C	-3.97684302256823	23.19182774474141	27.61490036240699
H	-3.78523815219843	22.58862985687542	26.71951255978650
C	-3.20663987568463	22.97465196007338	28.77431542047728
C	-2.23169440426749	21.86145806691115	28.79018162004031
C	-0.86917212403971	22.17512406002376	28.75586273975745
C	-0.34002685746712	23.52242450636463	28.68728999921965
H	-0.94494108378232	24.42972641203695	28.64360291720459
O	-6.64331998132619	26.39227401997499	29.95463403941723
C	-4.44489474693540	24.69495914145266	29.95929670405400
H	-4.65304468747399	25.27923075566475	30.86169553284152
C	-3.41881049819284	23.74583731500223	29.93408285153941
H	-2.80201552729843	23.56240499321535	30.82199394792162
C	-2.76984735190484	20.56761668450501	28.84447812702996
C	-4.18804438147482	20.26656724371992	28.92673093482061
H	-4.98782244960507	21.00851249259695	28.93049016290632
O	7.13627383823350	10.69863413288300	25.89932012701413
O	-6.64332010718305	8.55736613281503	26.39227392956897
O	-7.13627400212228	10.69863399291892	12.61267999790152
Rh	0.00394092515885	9.82400848773243	19.27048090096849
N	2.01993171105845	9.73092329089721	19.43483466582553
C	6.54476231481350	9.66632378160545	12.87348473548895
C	5.62673263598860	9.51512547347848	14.02052112036523
C	4.82210520733272	8.37391250099858	14.20207913507235
H	4.92677491263106	7.52306910179662	13.52069754509243
C	3.87143553121665	8.36591610959125	15.22633360615485
H	3.20466071467190	7.50690817023158	15.36634508706209
C	3.73231470368384	9.48725155488730	16.06981060260257
C	2.62205288967506	9.56295650426750	17.04492690646567
C	2.93653055348633	9.57801143693684	18.40752091044357
C	4.27921783869450	9.45380986031160	18.94179954813419
H	5.17989346746759	9.29595685152331	18.34535771353689

C	6.41460773563121	9.66907792078747	25.79362932846555
C	5.25572892806688	9.74411656160085	24.88779319894541
C	4.39067341146313	8.64187154044412	24.74131722925084
H	4.56681925968336	7.74179014974905	25.34072956872628
C	3.35843509189857	8.70682488120409	23.80460115849763
H	2.68882037583339	7.85417863009257	23.64195829846219
C	3.20862839120997	9.85851498618191	23.00354670782730
C	2.23252932090837	9.86223538482676	21.89337096863685
C	2.76340131250347	9.74067092954993	20.60306479453631
C	4.17371542916246	9.56221440776583	20.30628405564025
H	4.97253642150960	9.50642698615039	21.04796662634172
O	7.13627399243066	10.69863398278601	12.61267999270562
N	0.16804324522062	9.78603882198422	17.25312465867611
C	5.52811490749287	10.61607745528475	14.89439858327786
H	6.17232922320965	11.48429069114564	14.71641931244842
C	4.59020939393856	10.59713082958508	15.92369175486840
H	4.47007017131520	11.45680366065661	16.59333934780688
C	1.33374612127921	9.69938523234575	16.51042742795769
C	1.03669332883366	9.80079937306265	15.09219416693700
H	1.77810191042231	9.79472562559724	14.29155078409485
N	-0.15888307586847	9.86888763110907	21.28925704085823
C	5.06816920113341	10.92369327922316	24.13778576202553
H	5.75422685329785	11.76318251984065	24.29229193213129
C	4.04683156538350	10.97651946917044	23.19267748807138
H	3.90129680079593	11.86363157253545	22.56525745994022
C	0.87371076462074	9.92548415917599	22.21026228668069
C	0.34709121628183	10.02030719097846	23.55752857987285
H	0.95075188863996	10.10497495047812	24.46354806840866
C	-6.38162611782458	9.58988046925072	12.71150630249089
C	-5.24073603432558	9.77111147681329	13.63265999498010
C	-5.06097145673964	10.96331347898188	14.36195182774854
H	-5.74034688709586	11.80552552828412	14.19685076466967
C	-4.03954101163005	11.03777219330983	15.31054838030056
H	-3.89916494919001	11.94173206045331	15.91490816704452

C	-3.19609755633473	9.92941227697373	15.52766957257817
C	-2.22129660329295	9.94014601031259	16.64113142395897
C	-0.85892606453682	9.89505972999181	16.32949319098906
C	-0.32534139005627	9.92669840484457	14.98116327683320
H	-0.92081609431232	10.03912295093441	14.07306190614204
N	-2.01475728515847	9.88563515851595	19.10489745719596
C	-4.36892594976581	8.67901769505238	13.80827686738426
H	-4.54152920511159	7.76975163337466	13.22123030919164
C	-3.34276831056454	8.76228948537135	14.74850865743564
H	-2.67383175055168	7.91252687664056	14.92851868202594
C	-2.75700389665092	9.90044711623544	17.93603996051532
C	-4.17456265790120	9.80865692013296	18.23818091807899
H	-4.97531816479894	9.77225714619802	17.49774787045297
O	-7.13627378303987	10.69863377517537	25.89932014200602
C	-6.52269332016921	9.62131647224338	25.67765578042349
C	-5.59504214960155	9.59659566596842	24.53485131678596
C	-5.54260122798845	10.72786027790527	23.69275143404952
H	-6.21931153670301	11.56602681638779	23.89211001215622
C	-4.61079784676022	10.76889621533378	22.65803574995695
H	-4.52643422244703	11.64726726965870	22.00757820309376
C	-3.71776615730795	9.69235113307558	22.47876197222989
C	-2.61904552168832	9.79325289822819	21.49748018087264
C	-1.32548860509409	9.87096667638903	22.03236050227888
C	-1.02204395994465	9.97952268581722	23.44834998046985
H	-1.76108088773605	10.03590318373605	24.24933272117295
C	-4.75463867829693	8.48776731375964	24.31833581673493
H	-4.83952738372697	7.61591181216827	24.97617126075882
C	-3.81188313427961	8.54209079551960	23.29253501731445
H	-3.11959704891855	7.71029258926797	23.11840806643772
C	-2.93748161775818	9.81041907255242	20.13624655190142
C	-4.28543804669328	9.74337601215855	19.60471536605773
H	-5.19422065411688	9.64642230548280	20.20178797293939
C	-7.79090437691610	27.26045250444935	29.99569409399639
H	-7.69831636768630	28.06166159795633	29.24230909325146

H	-8.71474245884897	26.69086004324267	29.79484214998697
H	-7.81253510469101	27.68499175185291	31.00949034456936
C	8.01785540662411	27.03644356883681	27.77693308812847
H	8.44495071940641	27.05624543950508	26.76414573055549
H	7.45913029684337	27.96663663288884	27.97936910897867
H	8.81666705272319	26.93242935069523	28.53145958016816
C	-8.28547983162075	10.60233354065693	11.75553741536382
H	-7.98242417489133	10.36287454066080	10.72118522349940
H	-8.97268411121996	9.81459726142184	12.11106508713290
H	-8.77624614336479	11.58566820509095	11.79390529605324
C	7.49561309378213	8.65515008580791	10.96682933794850
H	7.45681771853918	7.67153596492266	10.47675351372621
H	7.13566068807012	9.44207885474190	10.28104771059512
H	8.53061304398142	8.89647110566140	11.26594835856731
Rh	-0.00095577190595	19.23217203551119	28.86439632239227
H	-0.10644536847758	19.09620331122475	27.28569524585225
Si	-0.39626494929717	18.83963802730783	25.39647912237363
C	1.47486169891068	18.98695896464703	25.25174263530810
H	1.70262142419368	19.57484689907518	24.34214453064357
H	1.82595541070662	19.59306091748523	26.10588032190694
C	-1.21657942461574	17.22676572477029	25.85898664243939
H	-1.64382661252801	16.70467576783970	24.98895377829579
H	-0.49758744210275	16.55241690420008	26.35360008919904
H	-2.02908934208558	17.43172873948694	26.57484421448784
C	-1.40034213641052	20.43320613085758	25.48602239110389
H	-2.17543168076475	20.43003602244547	24.69763130337276
H	-1.94187697375565	20.40701623448761	26.44870918939133
C	2.19692805126414	17.63489694680067	25.22896835622553
H	3.29316168221104	17.77143676240070	25.24354506945406
H	1.92739229468662	17.02325119106861	26.10580826455244
H	1.94752794268411	17.04350842273688	24.33064317320240
C	-0.52178158579554	21.69132573517008	25.39249473190381
H	-0.02281248577062	21.77213876562613	24.40954826541239
H	-1.12235965934838	22.60687375713566	25.53807908060810

H	0.26365347179232	21.69029871939519	26.16745188229888
C	-0.77927653435877	18.67768743115858	23.07333624786608
H	-1.87089329941352	18.59803259972816	23.18219279257596
H	-0.37226759323644	19.68571733092720	22.91369631753400
C	-0.06582790905758	17.58298255967735	22.63858386421101
H	0.99443473672834	17.72552513680424	22.37497937860866
C	-0.52622986396755	19.46401679812177	31.28425393392604
H	-1.12086563981242	20.37633686240704	31.16883314371411
H	-1.07234972547502	18.54315277023577	31.52862449787688
C	0.83946008038413	19.51358152338467	31.33801517780131
H	1.32459351078580	20.48916682551172	31.17781097712087
Zr	-10.14279095157251	8.20458555805969	30.89397452546775
O	-12.26309935048125	7.82603349739433	30.51245733570128
O	-8.69728932258117	7.81569021744158	32.75046223428505
O	-9.14126158154807	10.13027021643901	31.22683781499565
O	-8.00095566470648	8.04446436887282	30.13023403796925
C	-12.97710724702555	7.37050786535274	29.56275062467178
O	-12.77043223759996	7.52927501102205	28.32825396290499
Zr	-8.11536719231112	8.07959399309654	27.88404581510050
O	-6.82614262314486	6.54915537338271	28.37156322942791
O	-10.10781493662564	7.56151912383395	28.67955797242230
Zr	-7.23337602125536	10.16869557568426	30.62521536772879
O	-5.27980330064776	9.65904871169813	29.56282224242129
O	-7.97403572732055	10.00192398986422	28.66787024078659
Zr	-11.43850764321516	9.06165854749937	27.35834681123647
O	-13.24333478046106	10.12684397502015	28.31059648079809
O	-10.04457487217310	6.22366573902086	31.05683099029463
O	-10.66833922236885	9.87286593227656	29.37313603086560
O	-4.94902445598239	7.25038376357241	30.16291447609382
O	-6.38764670924897	8.79573859033381	31.99705013597369
O	-12.54680692885132	9.39839454453090	25.74229866926463
Zr	-10.55404109965431	11.74111448555243	30.80733899132014
Zr	-8.89185555603651	11.35578555475442	27.15539743924180
O	-7.04106807846930	11.43545986851832	32.44378518515446

O	-8.88374305065164	6.64143720521975	26.37883730177888
O	-11.17079156831104	8.86052809521350	32.71531577812050
O	-11.06394158032787	7.20869760040256	26.08313504641308
O	-9.42944289127258	9.33944763478232	26.74575774972979
O	-9.51176150642159	12.33785366391447	29.02994777789042
O	-11.06580636160026	11.23194415120379	27.08224477590015
C	-11.44688986707365	9.95625250340398	33.30462481553078
O	-11.27850246665443	11.11450601978474	32.84038991170672
O	-9.46158006488101	10.04604544023239	24.03834767624437
O	-9.10000189509865	12.51603941369357	25.57349138589299
O	-8.91198045095196	12.68656628899089	32.16158835517442
C	-7.85222880269271	12.36440315669893	32.76345638668471
O	-11.03835275153265	13.66535595533718	31.01759255068946
O	-12.51861747279674	11.48962732860925	30.32130085335953
C	-10.02381210893022	6.54370365564578	25.81743978469353
H	-11.39939160615186	11.49208107364991	26.20448435844669
H	-7.60518117548017	7.20385876730181	30.42538594847617
H	-9.50941534259941	13.31158135974170	28.99190762572989
H	-10.33099860083724	6.61261748749537	28.63068572257217
H	-9.17219224250755	12.00408426364570	24.73565053246103
H	-10.43443607562521	14.19198185662280	31.57040489827812
H	-6.51931130310172	5.92114002458588	27.69545542558219
H	-9.48811632452504	5.82888300179419	31.74953954878999
H	-5.87266861889586	9.20150693784861	32.71795839627298
H	-9.03870824863548	8.33261009137061	33.50193950086602
H	-7.75846374135351	8.17521232588017	32.56328637620001
H	-5.18904021745503	9.86967566988187	28.61672664896983
H	-4.99866600299319	8.65583541306037	29.71542563776725
H	-5.43456088303723	7.48764869720995	30.99298776979787
H	-5.62911309103999	6.85306139997615	29.53582467521623
H	-12.60421061888348	8.68996421748858	25.07817764038598
H	-9.47377960001924	9.60328870732672	24.92270181423845
H	-8.59978170893252	9.79564339845698	23.66476918445161
H	-13.13130482387552	12.17215933774092	30.64520393993018

H	-9.69651237849398	9.97150280707885	29.17080010737198
H	-12.97092044992895	10.68972791949219	29.13466142732493
H	-13.51248247926851	10.77432047080876	27.63465695327566
H	-13.87041336641058	6.77580480039517	29.85739306363489
H	-10.10869041533542	5.77829737553034	25.01274425939917
H	-11.87515539014701	9.86885346400278	34.32792747275378
H	-7.58223071695213	12.95224296457616	33.67172502113954
Zr	10.24235874950535	7.66997485387907	30.27067635017199
O	12.35601687624540	8.08109829401134	30.64270381135775
O	8.82876297878212	5.79733034354031	30.69399194032431
O	9.22483740308612	7.32319854887576	28.36948976758428
O	8.09792104644851	8.41073748452572	30.44609724176882
C	13.08298441127782	9.04560436352008	31.03512587548385
O	12.87260450392360	10.27408722493078	30.82950664516322
Zr	8.17971393753716	10.65458346855243	30.40309159105842
O	6.93906440164834	10.14855015070705	31.96815508834426
O	10.18938574326011	9.89568963994318	30.90678327361294
Zr	7.30664231117141	7.88883014713173	28.32308888339479
O	5.31250669200027	8.85731160588414	28.88508151885609
O	7.96820120460473	9.88104317174543	28.49259825143815
Zr	11.48128336276490	11.23657804409144	29.37335603034759
O	13.26070051105446	10.33321163857589	28.20963366749337
O	10.15584944075609	7.51755897671376	32.25420144473907
O	10.70664311997579	9.21872863595991	28.58011166715545
O	5.09618985163399	8.28526852127492	31.32140501508401
O	6.52111769478660	6.49214372071468	29.70398587263387
O	12.47948252051677	12.91258208657032	29.00600605380213
Zr	10.65603762006418	7.74130311770150	26.76865618801685
Zr	8.90383806097852	11.35811338276931	27.10994113905340
O	7.16929184290635	6.09025936015125	27.03596139833557
O	8.92946208857004	12.20054672327453	31.81188446575815
O	11.28247775555598	5.87033481691771	29.59244094543215
O	11.11167964328918	12.49140670022170	31.24726743070396
O	9.46224250599198	11.79588577518027	29.11640233184646

O	9.54039726865280	9.48028738738986	26.14990031719057
O	11.08301655599794	11.47743384255526	27.19176699048427
C	11.47374135599667	5.22253279052132	28.50939147668818
O	11.24918784804615	5.63687962821516	27.34287306581457
O	9.35403688076865	14.51226985759875	28.46325811944202
O	9.07713640377839	12.96931194450358	25.98639294390424
O	8.99956003006733	6.48510214189580	25.75836188666688
C	7.96733687960469	5.83353712834563	26.07650186486737
O	11.28341115955865	7.50374831386698	24.90168753747144
O	12.61579874031284	8.07036812567210	27.25162569596845
C	10.07128335125585	12.75871920521019	31.91053457603535
H	11.34994480334232	12.37789475587876	26.92914808942716
H	7.72761663422534	8.12418203710987	31.30103120623000
H	9.12634394417029	9.34151256421027	25.27944785272476
H	10.42098054926613	9.94576596588188	31.85361234827685
H	9.14495731640171	13.80081340575671	26.50924413313806
H	10.74165627962198	6.99277515531136	24.27623107007592
H	6.64563051351062	10.81020042831503	32.61703549882408
H	9.61239485167694	6.81361637711784	32.64757593346570
H	6.00619111082400	5.76358132929445	29.31203124802962
H	9.17914511487724	5.03586483554381	30.19867057364788
H	7.88737761210473	5.96403350305085	30.32516001773015
H	5.12850997890738	9.78301631864943	28.65044027007650
H	5.07832507294738	8.71354525849635	29.89694374766109
H	5.59270504460682	7.46729722715761	31.06698673398559
H	5.77025395074322	8.93291854433597	31.69145802606269
H	12.52705304368116	13.58132626656638	29.71049081754104
H	9.42739877964641	13.61155524309008	28.86739756177354
H	8.41108822960216	14.72264888824675	28.56788407007349
H	13.27873110080450	7.63190008622381	26.69061884199318
H	9.72911463069370	9.38456121534485	28.49976372300931
H	13.05082807154875	9.37756648288877	27.87567036893661
H	13.18795856213563	10.85493075829552	27.38663024993285
H	13.99417800661873	8.77728833877724	31.61451321773273

H	10.15727622710149	13.56287524340488	32.67682629173292
H	11.87746600142935	4.19137974994701	28.62139927439523
H	7.71279036841996	4.94242336117049	25.45782058275373
C	-0.60508673478219	16.20308803353838	22.46603244358429
H	-0.66012086305147	16.02867671247574	21.36696302782869
H	-1.64981438116511	16.14457125972075	22.82911503591817
C	0.25459665015678	15.07069814590456	23.07291299109715
H	1.29503276956891	15.17420754528845	22.70660229171571
H	0.29925666650843	15.20214815920680	24.17125379199507
C	-0.28299275806368	13.67560753807754	22.73231730885670
H	0.33740102923239	12.88042224252714	23.18052840082681
H	-1.31361796725075	13.53650052519514	23.10736730795157
H	-0.29967813225116	13.50106121029105	21.64110008428317
C	1.74134942346050	18.37443964378095	31.74741276548143
H	1.67489836270985	17.56238101596215	30.99983921068726
H	1.35354401770216	17.93258401108314	32.68773052093822
C	3.20802407937025	18.79236691601941	31.92937960109126
H	3.54196923068554	19.33964518190065	31.02858618807654
H	3.27833802803673	19.51266137144853	32.76866568299128
C	4.14416594209561	17.60617996623521	32.17219127188841
H	5.18532089159761	17.93890106351029	32.32987516661601
H	3.83950903207924	17.02160802259351	33.06075650076876
H	4.14061789402599	16.92082067785432	31.30477134988521

6-silane-TS-MOF (but with 1-pentene instead of ethylene)

O	6.64332001085071	8.55736603533582	12.11972600911177
O	-6.64332000001680	8.55736600977905	12.11972600004999
O	6.64331986047245	12.11972591645613	29.95463422817308
O	6.64331996917532	26.39227402163869	29.95463410888584
O	6.64332007336519	8.55736591316483	26.39227395651056
N	2.03018524566451	19.05969543900138	28.63748049685501
C	6.42057940147665	12.71449051860762	28.84109390681048
C	5.24455691458295	13.60803909913101	28.76702225846188
C	4.41663260200224	13.78865677946401	29.89036005236690

H	4.62459612757894	13.22071791903947	30.80356548392939
C	3.37864507939376	14.71903368445256	29.82939196144644
H	2.74192112400771	14.90674694787230	30.70160163660028
C	3.18481079090857	15.47729323393542	28.65474212968451
C	2.21610882242754	16.59341387730979	28.67221044838662
C	2.75302087321298	17.88845818586551	28.57899937383033
C	4.16948014603075	18.19244525059719	28.47967990552426
H	4.97069383438069	17.45412366424846	28.44001058592169
C	6.50447386631646	25.68068410784571	28.97565073156684
C	5.60916649918638	24.50178064358270	28.89510201623047
C	4.85240595710537	24.17715987763220	30.03601110819304
H	4.99739962051311	24.76516180380031	30.94942566388946
C	3.91750950946054	23.14445443897070	29.96667836171842
H	3.29696664342759	22.88969125098732	30.83395084714586
C	3.73637417144225	22.43865640888344	28.75799479678226
C	2.63106493155471	21.45684290015653	28.67465103611178
C	2.94209152330986	20.09509511880437	28.58639938681613
C	4.28614887531048	19.56041762611533	28.48848203173289
H	5.19764646695703	20.15935747444216	28.45578691197338
O	7.13627423344803	12.61268003507523	27.81336566831930
N	-0.16256713138504	17.20386666672089	28.99934214901811
C	5.00851516839231	14.30595101701704	27.56715260155850
H	5.66113764323992	14.11624721369904	26.70820076748820
C	3.97933133335505	15.24319435330388	27.51353188877865
H	3.79972174729266	15.82055794098441	26.60110853904151
C	0.86167761664776	16.28823789925424	28.86189505099404
C	0.32595653823393	14.94148336695038	28.89848022085433
H	0.91517814538038	14.02937791222751	28.79141276249166
O	7.13627403637332	25.89931997380708	27.81336588910666
N	0.16430809773519	21.26228445309877	28.76072578155828
C	5.47096340452906	23.76448499328518	27.70189942023352
H	6.07597923683522	24.03143415695181	26.82998240735054
C	4.53428389512922	22.73022463305470	27.63520498950215
H	4.38038434378028	22.17216142349421	26.70387133672153

C	1.33342415462445	21.99064852422323	28.71870094754474
C	1.02826761094452	23.40962036310777	28.67158875061959
H	1.76883471383631	24.20815705089141	28.60885631993186
O	-7.13627420074678	12.61268019282818	27.81336599325511
C	-6.54180779754943	12.83112426849024	28.89951252686314
C	-5.62748386916351	13.99242171568942	28.99611858191455
C	-5.44015540852238	14.82843921604834	27.88003381075414
H	-6.01492490913719	14.63379557526516	26.96759199433432
C	-4.49947770955149	15.85612415009863	27.95034867496867
H	-4.30849265132183	16.49954373668001	27.08352289604212
C	-3.74118989702430	16.03542900043733	29.12727107051938
C	-2.62945517808864	17.01118289132409	29.12130458667630
C	-1.33057956365933	16.47745689018353	29.10940404884108
C	-1.03252238873596	15.05782456000134	29.05815668685582
H	-1.77306244259761	14.25816926597820	29.10011970512603
O	-6.64331991688649	12.11972589329534	29.95463393553034
N	-2.04253473678926	19.39821171688036	28.88613995371906
C	-4.91847809679576	14.20621387103452	30.19439393184847
H	-5.10986360014332	13.54521949047835	31.04617999716113
C	-3.97171256982442	15.22817506799337	30.25881577351705
H	-3.38476161319277	15.39632201786433	31.16950594744201
C	-2.94945429340518	18.36886797867747	29.00123309227001
C	-4.29888933785970	18.90189107716773	29.02318946029380
H	-5.20631041697125	18.30378908057300	29.12179926326368
O	-7.13627402302773	25.89931998929027	27.81336595823270
C	-6.42233127516318	25.76624668555541	28.78934156335496
C	-5.24474328964976	24.86986972047973	28.81490348604570
C	-4.99596141451306	24.14122534312587	27.63556560635812
H	-5.63756445982131	24.32125724409431	26.76605525111439
C	-3.97684302256823	23.19182774474141	27.61490036240699
H	-3.78523815219843	22.58862985687542	26.71951255978650
C	-3.20663987568463	22.97465196007338	28.77431542047728
C	-2.23169440426749	21.86145806691115	28.79018162004031
C	-0.86917212403971	22.17512406002376	28.75586273975745

C	-0.34002685746712	23.52242450636463	28.68728999921965
H	-0.94494108378232	24.42972641203695	28.64360291720459
O	-6.64331998132619	26.39227401997499	29.95463403941723
C	-4.44489474693540	24.69495914145266	29.95929670405400
H	-4.65304468747399	25.27923075566475	30.86169553284152
C	-3.41881049819284	23.74583731500223	29.93408285153941
H	-2.80201552729843	23.56240499321535	30.82199394792162
C	-2.76984735190484	20.56761668450501	28.84447812702996
C	-4.18804438147482	20.26656724371992	28.92673093482061
H	-4.98782244960507	21.00851249259695	28.93049016290632
O	7.13627383823350	10.69863413288300	25.89932012701413
O	-6.64332010718305	8.55736613281503	26.39227392956897
O	-7.13627400212228	10.69863399291892	12.61267999790152
Rh	0.00394092515885	9.82400848773243	19.27048090096849
N	2.01993171105845	9.73092329089721	19.43483466582553
C	6.54476231481350	9.66632378160545	12.87348473548895
C	5.62673263598860	9.51512547347848	14.02052112036523
C	4.82210520733272	8.37391250099858	14.20207913507235
H	4.92677491263106	7.52306910179662	13.52069754509243
C	3.87143553121665	8.36591610959125	15.22633360615485
H	3.20466071467190	7.50690817023158	15.36634508706209
C	3.73231470368384	9.48725155488730	16.06981060260257
C	2.62205288967506	9.56295650426750	17.04492690646567
C	2.93653055348633	9.57801143693684	18.40752091044357
C	4.27921783869450	9.45380986031160	18.94179954813419
H	5.17989346746759	9.29595685152331	18.34535771353689
C	6.41460773563121	9.66907792078747	25.79362932846555
C	5.25572892806688	9.74411656160085	24.88779319894541
C	4.39067341146313	8.64187154044412	24.74131722925084
H	4.56681925968336	7.74179014974905	25.34072956872628
C	3.35843509189857	8.70682488120409	23.80460115849763
H	2.68882037583339	7.85417863009257	23.64195829846219
C	3.20862839120997	9.85851498618191	23.00354670782730
C	2.23252932090837	9.86223538482676	21.89337096863685

C	2.76340131250347	9.74067092954993	20.60306479453631
C	4.17371542916246	9.56221440776583	20.30628405564025
H	4.97253642150960	9.50642698615039	21.04796662634172
O	7.13627399243066	10.69863398278601	12.61267999270562
N	0.16804324522062	9.78603882198422	17.25312465867611
C	5.52811490749287	10.61607745528475	14.89439858327786
H	6.17232922320965	11.48429069114564	14.71641931244842
C	4.59020939393856	10.59713082958508	15.92369175486840
H	4.47007017131520	11.45680366065661	16.59333934780688
C	1.33374612127921	9.69938523234575	16.51042742795769
C	1.03669332883366	9.80079937306265	15.09219416693700
H	1.77810191042231	9.79472562559724	14.29155078409485
N	-0.15888307586847	9.86888763110907	21.28925704085823
C	5.06816920113341	10.92369327922316	24.13778576202553
H	5.75422685329785	11.76318251984065	24.29229193213129
C	4.04683156538350	10.97651946917044	23.19267748807138
H	3.90129680079593	11.86363157253545	22.56525745994022
C	0.87371076462074	9.92548415917599	22.21026228668069
C	0.34709121628183	10.02030719097846	23.55752857987285
H	0.95075188863996	10.10497495047812	24.46354806840866
C	-6.38162611782458	9.58988046925072	12.71150630249089
C	-5.24073603432558	9.77111147681329	13.63265999498010
C	-5.06097145673964	10.96331347898188	14.36195182774854
H	-5.74034688709586	11.80552552828412	14.19685076466967
C	-4.03954101163005	11.03777219330983	15.31054838030056
H	-3.89916494919001	11.94173206045331	15.91490816704452
C	-3.19609755633473	9.92941227697373	15.52766957257817
C	-2.22129660329295	9.94014601031259	16.64113142395897
C	-0.85892606453682	9.89505972999181	16.32949319098906
C	-0.32534139005627	9.92669840484457	14.98116327683320
H	-0.92081609431232	10.03912295093441	14.07306190614204
N	-2.01475728515847	9.88563515851595	19.10489745719596
C	-4.36892594976581	8.67901769505238	13.80827686738426
H	-4.54152920511159	7.76975163337466	13.22123030919164

C	-3.34276831056454	8.76228948537135	14.74850865743564
H	-2.67383175055168	7.91252687664056	14.92851868202594
C	-2.75700389665092	9.90044711623544	17.93603996051532
C	-4.17456265790120	9.80865692013296	18.23818091807899
H	-4.97531816479894	9.77225714619802	17.49774787045297
O	-7.13627378303987	10.69863377517537	25.89932014200602
C	-6.52269332016921	9.62131647224338	25.67765578042349
C	-5.59504214960155	9.59659566596842	24.53485131678596
C	-5.54260122798845	10.72786027790527	23.69275143404952
H	-6.21931153670301	11.56602681638779	23.89211001215622
C	-4.61079784676022	10.76889621533378	22.65803574995695
H	-4.52643422244703	11.64726726965870	22.00757820309376
C	-3.71776615730795	9.69235113307558	22.47876197222989
C	-2.61904552168832	9.79325289822819	21.49748018087264
C	-1.32548860509409	9.87096667638903	22.03236050227888
C	-1.02204395994465	9.97952268581722	23.44834998046985
H	-1.76108088773605	10.03590318373605	24.24933272117295
C	-4.75463867829693	8.48776731375964	24.31833581673493
H	-4.83952738372697	7.61591181216827	24.97617126075882
C	-3.81188313427961	8.54209079551960	23.29253501731445
H	-3.11959704891855	7.71029258926797	23.11840806643772
C	-2.93748161775818	9.81041907255242	20.13624655190142
C	-4.28543804669328	9.74337601215855	19.60471536605773
H	-5.19422065411688	9.64642230548280	20.20178797293939
C	-7.79090437691610	27.26045250444935	29.99569409399639
H	-7.69831636768630	28.06166159795633	29.24230909325146
H	-8.71474245884897	26.69086004324267	29.79484214998697
H	-7.81253510469101	27.68499175185291	31.00949034456936
C	8.01785540662411	27.03644356883681	27.77693308812847
H	8.44495071940641	27.05624543950508	26.76414573055549
H	7.45913029684337	27.96663663288884	27.97936910897867
H	8.81666705272319	26.93242935069523	28.53145958016816
C	-8.28547983162075	10.60233354065693	11.75553741536382
H	-7.98242417489133	10.36287454066080	10.72118522349940

H	-8.97268411121996	9.81459726142184	12.11106508713290
H	-8.77624614336479	11.58566820509095	11.79390529605324
C	7.49561309378213	8.65515008580791	10.96682933794850
H	7.45681771853918	7.67153596492266	10.47675351372621
H	7.13566068807012	9.44207885474190	10.28104771059512
H	8.53061304398142	8.89647110566140	11.26594835856731
Rh	-0.00095577190595	19.23217203551119	28.86439632239227
H	-0.10644536847758	19.09620331122475	27.28569524585225
Si	-0.39626494929717	18.83963802730783	25.39647912237363
C	1.47486169891068	18.98695896464703	25.25174263530810
H	1.70262142419368	19.57484689907518	24.34214453064357
H	1.82595541070662	19.59306091748523	26.10588032190694
C	-1.21657942461574	17.22676572477029	25.85898664243939
H	-1.64382661252801	16.70467576783970	24.98895377829579
H	-0.49758744210275	16.55241690420008	26.35360008919904
H	-2.02908934208558	17.43172873948694	26.57484421448784
C	-1.40034213641052	20.43320613085758	25.48602239110389
H	-2.17543168076475	20.43003602244547	24.69763130337276
H	-1.94187697375565	20.40701623448761	26.44870918939133
C	2.19692805126414	17.63489694680067	25.22896835622553
H	3.29316168221104	17.77143676240070	25.24354506945406
H	1.92739229468662	17.02325119106861	26.10580826455244
H	1.94752794268411	17.04350842273688	24.33064317320240
C	-0.52178158579554	21.69132573517008	25.39249473190381
H	-0.02281248577062	21.77213876562613	24.40954826541239
H	-1.12235965934838	22.60687375713566	25.53807908060810
H	0.26365347179232	21.69029871939519	26.16745188229888
C	-0.77927653435877	18.67768743115858	23.07333624786608
H	-1.87089329941352	18.59803259972816	23.18219279257596
H	-0.37226759323644	19.68571733092720	22.91369631753400
C	-0.06582790905758	17.58298255967735	22.63858386421101
H	0.99443473672834	17.72552513680424	22.37497937860866
C	-0.52622986396755	19.46401679812177	31.28425393392604
H	-1.12086563981242	20.37633686240704	31.16883314371411

H	-1.07234972547502	18.54315277023577	31.52862449787688
C	0.83946008038413	19.51358152338467	31.33801517780131
H	1.32459351078580	20.48916682551172	31.17781097712087
Zr	-10.14279095157251	8.20458555805969	30.89397452546775
O	-12.26309935048125	7.82603349739433	30.51245733570128
O	-8.69728932258117	7.81569021744158	32.75046223428505
O	-9.14126158154807	10.13027021643901	31.22683781499565
O	-8.00095566470648	8.04446436887282	30.13023403796925
C	-12.97710724702555	7.37050786535274	29.56275062467178
O	-12.77043223759996	7.52927501102205	28.32825396290499
Zr	-8.11536719231112	8.07959399309654	27.88404581510050
O	-6.82614262314486	6.54915537338271	28.37156322942791
O	-10.10781493662564	7.56151912383395	28.67955797242230
Zr	-7.23337602125536	10.16869557568426	30.62521536772879
O	-5.27980330064776	9.65904871169813	29.56282224242129
O	-7.97403572732055	10.00192398986422	28.66787024078659
Zr	-11.43850764321516	9.06165854749937	27.35834681123647
O	-13.24333478046106	10.12684397502015	28.31059648079809
O	-10.04457487217310	6.22366573902086	31.05683099029463
O	-10.66833922236885	9.87286593227656	29.37313603086560
O	-4.94902445598239	7.25038376357241	30.16291447609382
O	-6.38764670924897	8.79573859033381	31.99705013597369
O	-12.54680692885132	9.39839454453090	25.74229866926463
Zr	-10.55404109965431	11.74111448555243	30.80733899132014
Zr	-8.89185555603651	11.35578555475442	27.15539743924180
O	-7.04106807846930	11.43545986851832	32.44378518515446
O	-8.88374305065164	6.64143720521975	26.37883730177888
O	-11.17079156831104	8.86052809521350	32.71531577812050
O	-11.06394158032787	7.20869760040256	26.08313504641308
O	-9.42944289127258	9.33944763478232	26.74575774972979
O	-9.51176150642159	12.33785366391447	29.02994777789042
O	-11.06580636160026	11.23194415120379	27.08224477590015
C	-11.44688986707365	9.95625250340398	33.30462481553078
O	-11.27850246665443	11.11450601978474	32.84038991170672

O	-9.46158006488101	10.04604544023239	24.03834767624437
O	-9.10000189509865	12.51603941369357	25.57349138589299
O	-8.91198045095196	12.68656628899089	32.16158835517442
C	-7.85222880269271	12.36440315669893	32.76345638668471
O	-11.03835275153265	13.66535595533718	31.01759255068946
O	-12.51861747279674	11.48962732860925	30.32130085335953
C	-10.02381210893022	6.54370365564578	25.81743978469353
H	-11.39939160615186	11.49208107364991	26.20448435844669
H	-7.60518117548017	7.20385876730181	30.42538594847617
H	-9.50941534259941	13.31158135974170	28.99190762572989
H	-10.33099860083724	6.61261748749537	28.63068572257217
H	-9.17219224250755	12.00408426364570	24.73565053246103
H	-10.43443607562521	14.19198185662280	31.57040489827812
H	-6.51931130310172	5.92114002458588	27.69545542558219
H	-9.48811632452504	5.82888300179419	31.74953954878999
H	-5.87266861889586	9.20150693784861	32.71795839627298
H	-9.03870824863548	8.33261009137061	33.50193950086602
H	-7.75846374135351	8.17521232588017	32.56328637620001
H	-5.18904021745503	9.86967566988187	28.61672664896983
H	-4.99866600299319	8.65583541306037	29.71542563776725
H	-5.43456088303723	7.48764869720995	30.99298776979787
H	-5.62911309103999	6.85306139997615	29.53582467521623
H	-12.60421061888348	8.68996421748858	25.07817764038598
H	-9.47377960001924	9.60328870732672	24.92270181423845
H	-8.59978170893252	9.79564339845698	23.66476918445161
H	-13.13130482387552	12.17215933774092	30.64520393993018
H	-9.69651237849398	9.97150280707885	29.17080010737198
H	-12.97092044992895	10.68972791949219	29.13466142732493
H	-13.51248247926851	10.77432047080876	27.63465695327566
H	-13.87041336641058	6.77580480039517	29.85739306363489
H	-10.10869041533542	5.77829737553034	25.01274425939917
H	-11.87515539014701	9.86885346400278	34.32792747275378
H	-7.58223071695213	12.95224296457616	33.67172502113954
Zr	10.24235874950535	7.66997485387907	30.27067635017199

O	12.35601687624540	8.08109829401134	30.64270381135775
O	8.82876297878212	5.79733034354031	30.69399194032431
O	9.22483740308612	7.32319854887576	28.36948976758428
O	8.09792104644851	8.41073748452572	30.44609724176882
C	13.08298441127782	9.04560436352008	31.03512587548385
O	12.87260450392360	10.27408722493078	30.82950664516322
Zr	8.17971393753716	10.65458346855243	30.40309159105842
O	6.93906440164834	10.14855015070705	31.96815508834426
O	10.18938574326011	9.89568963994318	30.90678327361294
Zr	7.30664231117141	7.88883014713173	28.32308888339479
O	5.31250669200027	8.85731160588414	28.88508151885609
O	7.96820120460473	9.88104317174543	28.49259825143815
Zr	11.48128336276490	11.23657804409144	29.37335603034759
O	13.26070051105446	10.33321163857589	28.20963366749337
O	10.15584944075609	7.51755897671376	32.25420144473907
O	10.70664311997579	9.21872863595991	28.58011166715545
O	5.09618985163399	8.28526852127492	31.32140501508401
O	6.52111769478660	6.49214372071468	29.70398587263387
O	12.47948252051677	12.91258208657032	29.00600605380213
Zr	10.65603762006418	7.74130311770150	26.76865618801685
Zr	8.90383806097852	11.35811338276931	27.10994113905340
O	7.16929184290635	6.09025936015125	27.03596139833557
O	8.92946208857004	12.20054672327453	31.81188446575815
O	11.28247775555598	5.87033481691771	29.59244094543215
O	11.11167964328918	12.49140670022170	31.24726743070396
O	9.46224250599198	11.79588577518027	29.11640233184646
O	9.54039726865280	9.48028738738986	26.14990031719057
O	11.08301655599794	11.47743384255526	27.19176699048427
C	11.47374135599667	5.22253279052132	28.50939147668818
O	11.24918784804615	5.63687962821516	27.34287306581457
O	9.35403688076865	14.51226985759875	28.46325811944202
O	9.07713640377839	12.96931194450358	25.98639294390424
O	8.99956003006733	6.48510214189580	25.75836188666688
C	7.96733687960469	5.83353712834563	26.07650186486737

O	11.28341115955865	7.50374831386698	24.90168753747144
O	12.61579874031284	8.07036812567210	27.25162569596845
C	10.07128335125585	12.75871920521019	31.91053457603535
H	11.34994480334232	12.37789475587876	26.92914808942716
H	7.72761663422534	8.12418203710987	31.30103120623000
H	9.12634394417029	9.34151256421027	25.27944785272476
H	10.42098054926613	9.94576596588188	31.85361234827685
H	9.14495731640171	13.80081340575671	26.50924413313806
H	10.74165627962198	6.99277515531136	24.27623107007592
H	6.64563051351062	10.81020042831503	32.61703549882408
H	9.61239485167694	6.81361637711784	32.64757593346570
H	6.00619111082400	5.76358132929445	29.31203124802962
H	9.17914511487724	5.03586483554381	30.19867057364788
H	7.88737761210473	5.96403350305085	30.32516001773015
H	5.12850997890738	9.78301631864943	28.65044027007650
H	5.07832507294738	8.71354525849635	29.89694374766109
H	5.59270504460682	7.46729722715761	31.06698673398559
H	5.77025395074322	8.93291854433597	31.69145802606269
H	12.52705304368116	13.58132626656638	29.71049081754104
H	9.42739877964641	13.61155524309008	28.86739756177354
H	8.41108822960216	14.72264888824675	28.56788407007349
H	13.27873110080450	7.63190008622381	26.69061884199318
H	9.72911463069370	9.38456121534485	28.49976372300931
H	13.05082807154875	9.37756648288877	27.87567036893661
H	13.18795856213563	10.85493075829552	27.38663024993285
H	13.99417800661873	8.77728833877724	31.61451321773273
H	10.15727622710149	13.56287524340488	32.67682629173292
H	11.87746600142935	4.19137974994701	28.62139927439523
H	7.71279036841996	4.94242336117049	25.45782058275373
C	-0.60508673478219	16.20308803353838	22.46603244358429
H	-0.66012086305147	16.02867671247574	21.36696302782869
H	-1.64981438116511	16.14457125972075	22.82911503591817
C	0.25459665015678	15.07069814590456	23.07291299109715
H	1.29503276956891	15.17420754528845	22.70660229171571

H	0.29925666650843	15.20214815920680	24.17125379199507
C	-0.28299275806368	13.67560753807754	22.73231730885670
H	0.33740102923239	12.88042224252714	23.18052840082681
H	-1.31361796725075	13.53650052519514	23.10736730795157
H	-0.29967813225116	13.50106121029105	21.64110008428317
C	1.74134942346050	18.37443964378095	31.74741276548143
H	1.67489836270985	17.56238101596215	30.99983921068726
H	1.35354401770216	17.93258401108314	32.68773052093822
C	3.20802407937025	18.79236691601941	31.92937960109126
H	3.54196923068554	19.33964518190065	31.02858618807654
H	3.27833802803673	19.51266137144853	32.76866568299128
C	4.14416594209561	17.60617996623521	32.17219127188841
H	5.18532089159761	17.93890106351029	32.32987516661601
H	3.83950903207924	17.02160802259351	33.06075650076876
H	4.14061789402599	16.92082067785432	31.30477134988521

25 Synthesis of **S1**

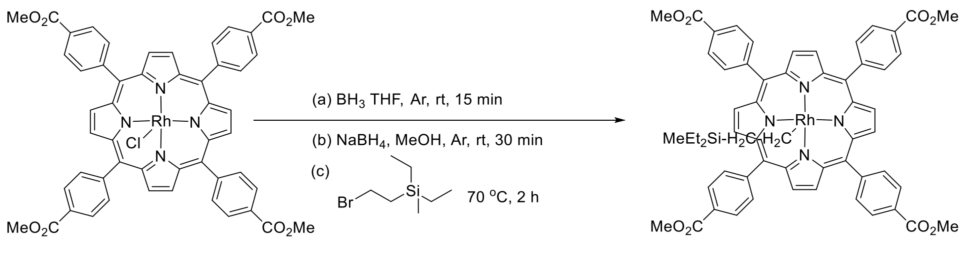

Rh-Cl TCPPOMe (20 mg, 0.020 mmol, 1.0 equiv) was added into a 100 mL Schlenk flask charged with a magnetic stir bar, then dry and degassed THF (0.2 mL) and BH_3 (1M THF) (0.04 mL, 0.04 mmol, 2 equiv) were added sequentially into the reaction flask under argon protection, followed by additional 0.2 mL dry degassed THF. The mixture was allowed to stir at room temperature for 15 min. In the meantime, $NaBH_4$ (25.4 mg, 3.30 mmol, 33.0 equiv) was dissolved in 1 mL degassed MeOH. When the 15 minutes had elapsed, the solution of $NaBH_4$ in methanol was added to the reaction mixture under argon protection. After 30 min at room temperature, Br-CH_2-CH_2-$SiEt_2Me$ (86 mg, 0.40 mmol, 20 equiv) was added under argon protection. The reaction mixture was heated at 70 °C for 1 hour. After removing the solvents under reduced pressure, the product was obtained by liquid extraction with DCM and water. The DCM phase was washed with water (3 x 5 mL), followed by a brine wash (1 x 10 mL). The extracted red-orange DCM solution was evaporated to dryness under reduced pressure, before the residue was washed with pentane (3 x 5 mL), and subsequently dried under vacuum at 70 °C for 1 h. After all solvent residue had been removed, **S1** (22 mg, 0.020 mmol) was obtained in quantitative yield. (note: the product is a light sensitive complex, and all manipulations should be carried out in the dark if possible).

The product was used as a solid-state NMR reference in Figure S47.

NMR Spectroscopy for S1:

^{1}H NMR (600 MHz, C_6D_6) δ 8.77 (s, 8H), 8.51 (dd, J = 7.8, 1.9 Hz, 4H), 8.44 (dd, J = 7.8, 1.9 Hz, 4H), 8.22 (dd, J = 7.8, 1.9 Hz, 4H), 8.17 (dd, J = 7.8, 1.9 Hz, 4H), 3.69 (s, 12H), -0.25 (t, J = 8.0 Hz, 6H), -1.04 – -1.17 (m, 4H), -1.63 (s, 3H), -4.34 – -4.45 (m, 2H), -4.84 – -4.94 (m, 2H).

^{13}C {^{1}H} NMR (151 MHz, C_6D_6) δ 166.8, 147.2, 143.1, 134.3, 134.2, 132.0, 130.4, 128.5, 128.5, 122.1 (d, $^3J_{Rh\text{-}C}$ = 1.1 Hz), 51.9, 14.4, 13.4 (d, $^1J_{Rh\text{-}C}$ = 26.8 Hz), 6.4, 3.7, -7.7.

^{29}Si {^{1}H}-INEPT-NMR (119 MHz, C_6D_6) δ -2.1 (d, $^3J_{Rh\text{-}Si}$ = 3.4 Hz).

^{103}Rh NMR (^{1}H-^{103}Rh HMBC, C_6D_6) δ 2792

HRMS ESI (m/z): calc'd for $C_{59}H_{54}N_4O_8Rh_1Si_1$ $[M+H]^+$: 1077.2761; Found: 1077.2766. Deviation: -0.5 ppm

26 NMR Spectra of **S1**

26.1 ^{1}H NMR Spectrum in C_6D_6

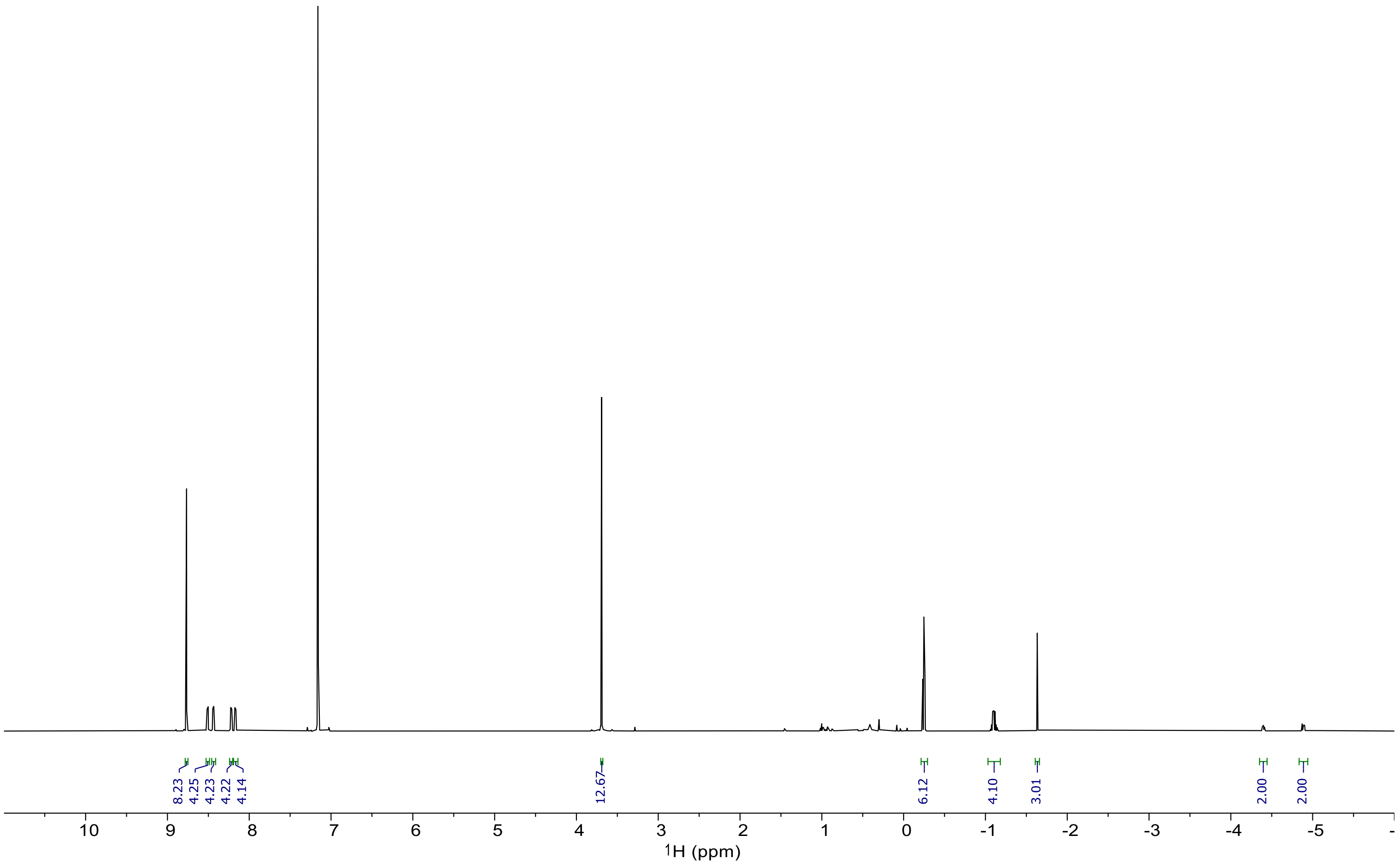

26.2 ^{13}C NMR Spectrum in C_6D_6

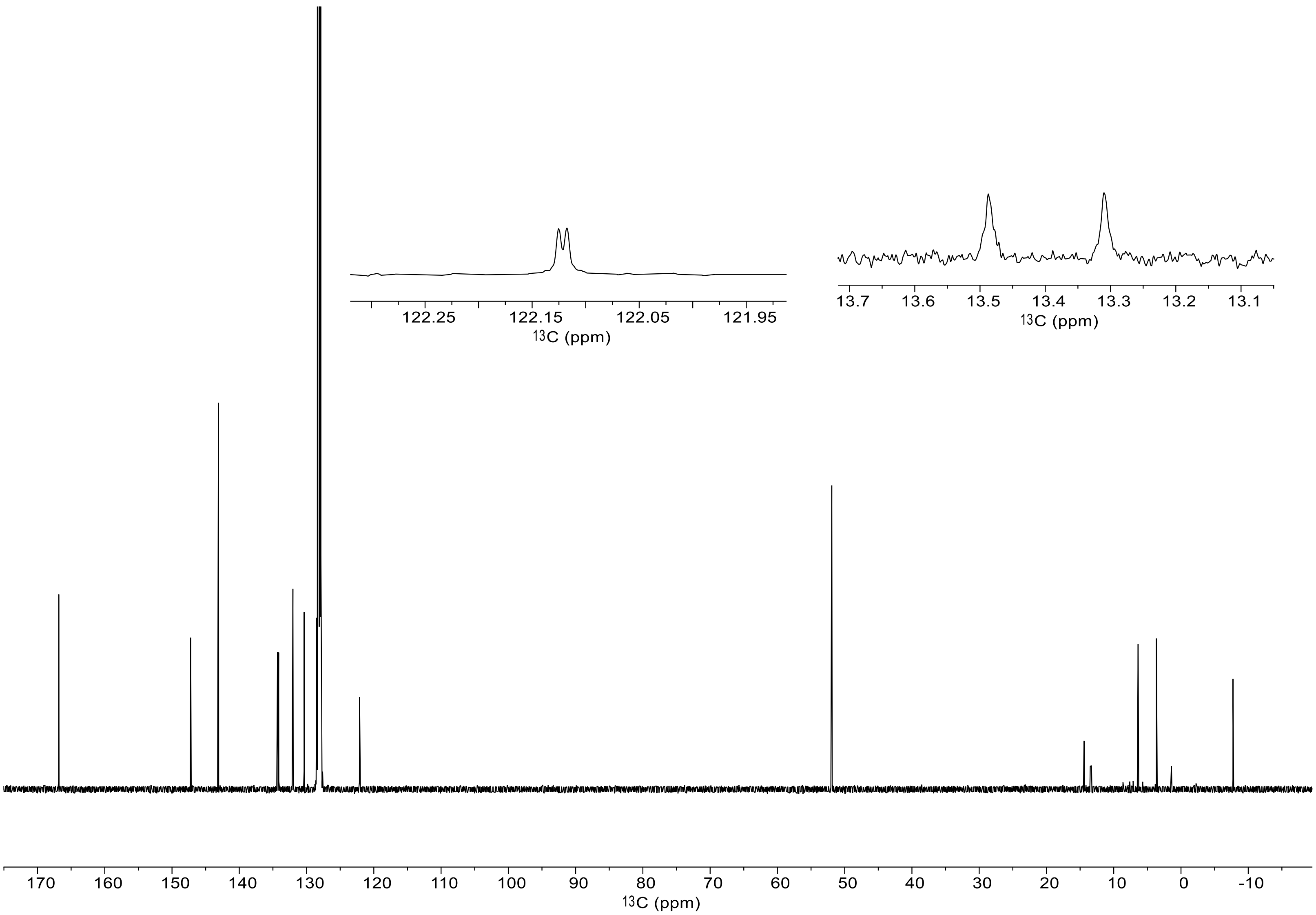

26.3 $^{29}Si\{^{1}H\}$ NMR Spectrum in C_6D_6

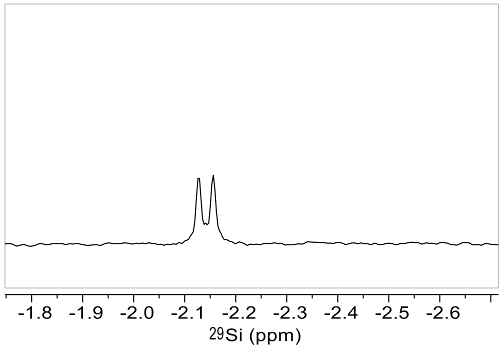

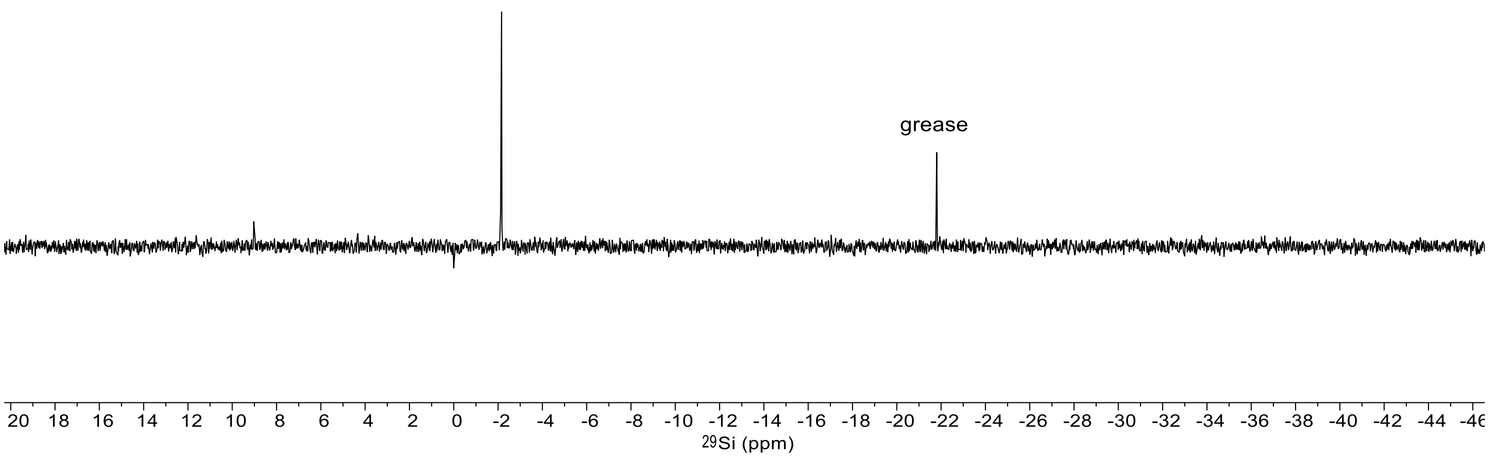

26.4 ^{1}H-^{29}Si HMBC NMR Spectrum in C_6D_6

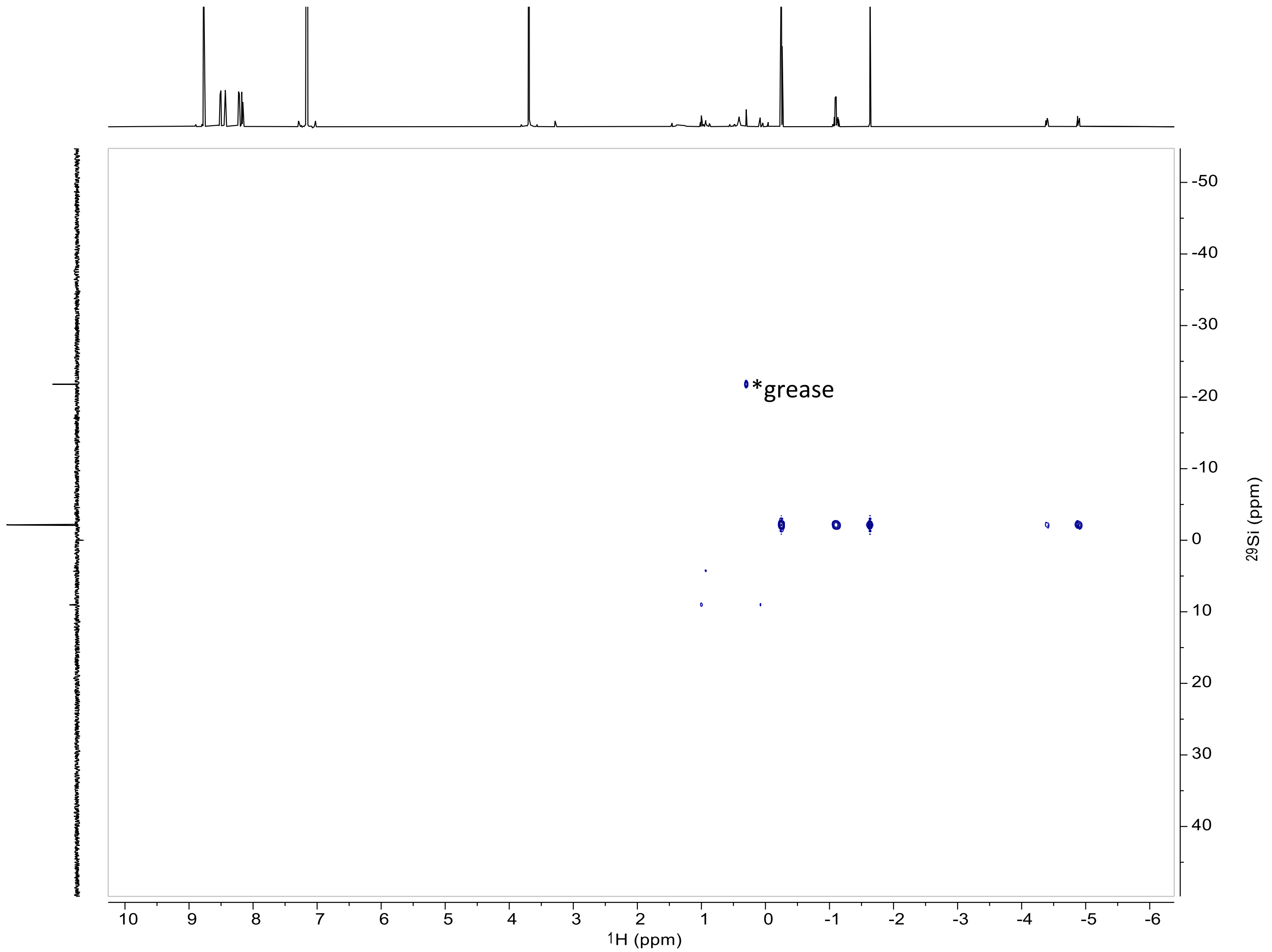

26.5 ^{1}H-^{103}Rh HMBC NMR Spectrum in C_6D_6

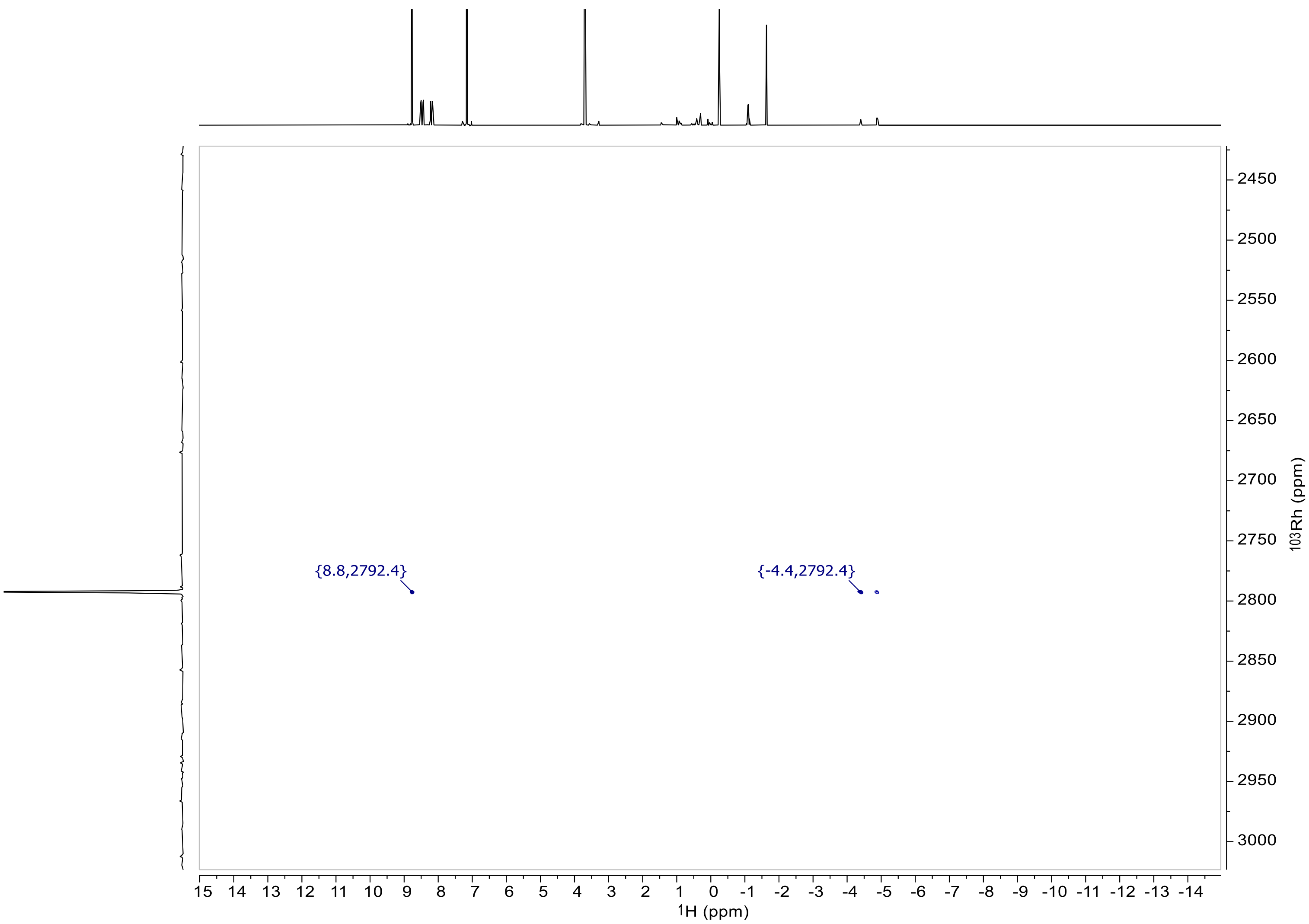

27 References

(61) Qiu, Z.; Deng, H.; Neumann, C. N. Site-Isolated Rhodium(II) Metalloradicals Catalyze Olefin Hydrofunctionalization. *Angew. Chem. Int. Ed.* **2024**, *63* (18), e202401375. DOI: 10.1002/anie.202401375
(62) Fulmer, G. R.; Miller, A. J. M.; Sherden, N. H.; Gottlieb, H. E.; Nudelman, A.; Stoltz, B. M.; Bercaw, J. E.; Goldberg, K. I. NMR Chemical Shifts of Trace Impurities: Common Laboratory Solvents, Organics, and Gases in Deuterated Solvents Relevant to the Organometallic Chemist. *Organometallics* **2010**, *29* (9), 2176-2179. DOI: 10.1021/om100106e
(63) Carmona, D.; Lamata, M. P.; Ferrer, J.; Modrego, J.; Perales, M.; Lahoz, F. J.; Atencio, R.; Oro, L. A. Synthesis, characterization and molecular structure of the hydroperoxo complex [(η5C5Me5)Ir(μ-pz)3Rh(OOH)(dppe)][BF4]; Hpz = pyrazole, dppe β 1,2-bis(diphenylphosphino)ethane. *J. Chem. Soc., Chem. Commun.* **1994**, (5), 575-576, 10.1039/C39940000575. DOI: 10.1039/C39940000575
(64) Reijerse, E.; Lendzian, F.; Isaacson, R.; Lubitz, W. A tunable general purpose Q-band resonator for CW and pulse EPR/ENDOR experiments with large sample access and optical excitation. *J. Magn. Reson.* **2012**, *214*, 237-243. DOI: 10.1016/j.jmr.2011.11.011
(65) Höfer, P.; Grupp, A.; Nebenführ, H.; Mehring, M. Hyperfine sublevel correlation (hyscore) spectroscopy: a 2D ESR investigation of the squaric acid radical. *Chem. Phys. Lett.* **1986**, *132* (3), 279-282. DOI: 10.1016/0009-2614(86)80124-5
(66) Jeschke, G.; Pannier, M.; Godt, A.; Spiess, H. W. Dipolar spectroscopy and spin alignment in electron paramagnetic resonance. *Chem. Phys. Lett.* **2000**, *331* (2), 243-252. DOI: 10.1016/S0009-2614(00)01171-4
(67) Jeschke, G. Determination of the Nanostructure of Polymer Materials by Electron Paramagnetic Resonance Spectroscopy. *Macromol. Rapid Commun.* **2002**, *23* (4), 227-246. DOI: 10.1002/1521-3927(20020301)23:4<227::AID-MARC227>3.0.CO;2-D
(68) Armbrecht, M.; Maringgele, W.; Meller, A.; Noltemeyer, M.; Sheldrick, G. M. Synthesen und Eigenschaften von Piperidin-N-oxyboranen / Synthesis and Properties of Piperidine-N-oxyboranes. *Zeitschrift für Naturforschung B* **1985**, *40* (9), 1113-1122. DOI: 10.1515/znb-1985-0904
(69) Tehfe, M.-A.; Lalevée, J.; Morlet-Savary, F.; Graff, B.; Blanchard, N.; Fouassier, J.-P. Tunable Organophotocatalysts for Polymerization Reactions Under Visible Lights. *Macromolecules* **2012**, *45* (4), 1746-1752. DOI: 10.1021/ma300050n
(70) Qrareya, H.; Dondi, D.; Ravelli, D.; Fagnoni, M. Decatungstate-Photocatalyzed Si−H/C−H Activation in Silyl Hydrides: Hydrosilylation of Electron-Poor Alkenes. *ChemCatChem* **2015**, *7* (20), 3350-3357. DOI: 10.1002/cctc.201500562
(71) Tehfe, M.-A.; Lalevée, J.; Morlet-Savary, F.; Graff, B.; Fouassier, J.-P. A Breakthrough toward Long Wavelength Cationic Photopolymerization: Initiating Systems Based on Violanthrone Derivatives and Silyl Radicals. *Macromolecules* **2011**, *44* (21), 8374-8379. DOI: 10.1021/ma2017265
(72) Chan, Y. W.; Chan, K. S. Base-Promoted Carbon− Hydrogen Bond Activation of Alkanes with Rhodium (III) Porphyrin Complexes. *Organometallics* **2008**, *27* (18), 4625-4635. DOI: 10.1021/om800397p
(73) Mahmood, J.; Ahmad, I.; Jung, M.; Seo, J.-M.; Yu, S.-Y.; Noh, H.-J.; Kim, Y. H.; Shin, H.-J.; Baek, J.-B. Two-dimensional amine and hydroxy functionalized fused aromatic covalent organic framework. *Commun. Chem.* **2020**, *3* (1), 31. DOI: 10.1038/s42004-020-0278-1
(74) Paonessa, R. S.; Thomas, N. C.; Halpern, J. Insertion and oxidative addition reactions of rhodium porphyrin complexes. Novel free radical chain mechanisms. *J. Am. Chem. Soc.* **1985**, *107* (14), 4333-4335. DOI: 10.1021/ja00300a044

(75) Bunn, A. G.; Wayland, B. B. One-electron activation and coupling of ethene by rhodium (II) porphyrins: observation of an. eta. 2-ethene-metalloradical complex. *J. Am. Chem. Soc.* **1992**, *114* (17), 6917-6919. DOI: 10.1021/ja00043a048
(76) Wayland, B. B.; Sherry, A. E.; Bunn, A. G. EPR studies of 1: 1 complexes of rhodium (II) and cobalt (II) porphyrins with. sigma. donor and. pi. acceptor ligands: origins of rhodium (II) metalloradical reactivity. *J. Am. Chem. Soc.* **1993**, *115* (17), 7675-7684. DOI: 10.1021/ja00070a011
(77) Neese, F. Software update: The ORCA program system—Version 5.0. *WIREs Comp. Mol. Sci.* **2022**, *12* (5), e1606. DOI: 10.1002/wcms.1606
(78) Feng, D.; Chung, W.-C.; Wei, Z.; Gu, Z.-Y.; Jiang, H.-L.; Chen, Y.-P.; Darensbourg, D. J.; Zhou, H.-C. Construction of Ultrastable Porphyrin Zr Metal–Organic Frameworks through Linker Elimination. *J. Am. Chem. Soc.* **2013**, *135* (45), 17105-17110. DOI: 10.1021/ja408084j
(79) Koschnick, C.; Stäglich, R.; Scholz, T.; Terban, M. W.; von Mankowski, A.; Savasci, G.; Binder, F.; Schökel, A.; Etter, M.; Nuss, J.; Siegel, R.; Germann, L. S.; Ochsenfeld, C.; Dinnebier, R. E.; Senker, J.; Lotsch, B. V. Understanding disorder and linker deficiency in porphyrinic zirconium-based metal–organic frameworks by resolving the Zr8O6 cluster conundrum in PCN-221. *Nat. Commun.* **2021**, *12* (1), 3099. DOI: 10.1038/s41467-021-23348-w
(80) Bannwarth, C.; Ehlert, S.; Grimme, S. GFN2-xTB—An Accurate and Broadly Parametrized Self-Consistent Tight-Binding Quantum Chemical Method with Multipole Electrostatics and Density-Dependent Dispersion Contributions. *J. Chem. Theory Comput.* **2019**, *15* (3), 1652-1671. DOI: 10.1021/acs.jctc.8b01176
(81) Becke, A. D. Density-functional exchange-energy approximation with correct asymptotic behavior. *Phys. Rev. A* **1988**, *38* (6), 3098. DOI: 10.1103/PhysRevA.38.3098
(82) Perdew, J. P. Density-functional approximation for the correlation energy of the inhomogeneous electron gas. *Phys. Rev. B* **1986**, *33* (12), 8822. DOI: 10.1103/PhysRevB.33.8822
(83) Grimme, S.; Antony, J.; Ehrlich, S.; Krieg, H. A consistent and accurate ab initio parametrization of density functional dispersion correction (DFT-D) for the 94 elements H-Pu. *J. Chem. Phys.* **2010**, *132* (15), 154104. DOI: 10.1063/1.3382344
(84) Grimme, S.; Ehrlich, S.; Goerigk, L. Effect of the damping function in dispersion corrected density functional theory. *J. Comput. Chem.* **2011**, *32* (7), 1456-1465. DOI: 10.1002/jcc.21759
(85) Rolfes, J. D.; Neese, F.; Pantazis, D. A. All ‐ electron scalar relativistic basis sets for the elements Rb–Xe. *J. Comput. Chem.* **2020**, *41* (20), 1842-1849. DOI: 10.1002/jcc.26355
(86) Pantazis, D. A.; Chen, X.-Y.; Landis, C. R.; Neese, F. All-electron scalar relativistic basis sets for third-row transition metal atoms. *J. Chem. Theory Comput.* **2008**, *4* (6), 908-919. DOI: 10.1021/ct800047t
(87) van Lenthe, E.; Baerends, E.-J.; Snijders, J. G. Relativistic regular two ‐ component Hamiltonians. *J. Chem. Phys.* **1993**, *99* (6), 4597-4610. DOI: 10.1063/1.466059
(88) van Lenthe, E.; Baerends, E.-J.; Snijders, J. G. Relativistic total energy using regular approximations. *J. Chem. Phys.* **1994**, *101* (11), 9783-9792. DOI: 10.1063/1.467943
(89) Van Lenthe, E.; Van Leeuwen, R.; Baerends, E.; Snijders, J. Relativistic regular two ‐ component Hamiltonians. *Int. J. Quantum Chem.* **1996**, *57* (3), 281-293. DOI: 10.1063/1.466059
(90) Vahtras, O.; Almlöf, J.; Feyereisen, M. Integral approximations for LCAO-SCF calculations. *Chem. Phys. Lett.* **1993**, *213* (5-6), 514-518. DOI: 10.1016/0009-2614(93)89151-7

(91) Weigend, F. Accurate Coulomb-fitting basis sets for H to Rn. *Phys. Chem. Chem. Phys.* **2006**, *8* (9), 1057-1065. DOI: 10.1039/B515623H

(92) Barone, V.; Cossi, M. Quantum calculation of molecular energies and energy gradients in solution by a conductor solvent model. *J. Phys. Chem. A* **1998**, *102* (11), 1995-2001. DOI: 10.1021/jp9716997

(93) Helmich-Paris, B.; de Souza, B.; Neese, F.; Izsák, R. An improved chain of spheres for exchange algorithm. *J. Chem. Phys.* **2021**, *155* (10), 104109. DOI: 10.1063/5.0058766
(94) Zhang, J.; Zhang, W.; Xu, M.; Zhang, Y.; Fu, X.; Fang, H. Production of Formamides from CO and Amines Induced by Porphyrin Rhodium(II) Metalloradical. *J. Am. Chem. Soc.* **2018**, *140* (21), 6656-6660. DOI: 10.1021/jacs.8b03029
(95) Li, H.; Han, B.; Wang, R.; Li, W.; Zhang, W.; Fu, X.; Fang, H.; Ma, F.; Wang, Z.; Zhang, J. Photochemical conversion of CO to C1 and C2 products mediated by porphyrin rhodium(II) metallo-radical complexes. *Nat. Commun.* **2024**, *15* (1), 7724. DOI: 10.1038/s41467-024-50253-9
(96) Riplinger, C.; Sandhoefer, B.; Hansen, A.; Neese, F. Natural triple excitations in local coupled cluster calculations with pair natural orbitals. *J. Chem. Phys.* **2013**, *139* (13), 134101. DOI: 10.1063/1.4821834
(97) Riplinger, C.; Pinski, P.; Becker, U.; Valeev, E. F.; Neese, F. Sparse maps—A systematic infrastructure for reduced-scaling electronic structure methods. II. Linear scaling domain based pair natural orbital coupled cluster theory. *J. Chem. Phys.* **2016**, *144* (2), 024109. DOI: 10.1063/1.4939030
(98) Chai, J.-D.; Head-Gordon, M. Systematic optimization of long-range corrected hybrid density functionals. *J. Chem. Phys.* **2008**, *128* (8). DOI: 10.1063/1.2834918
(99) Neese, F.; Wennmohs, F.; Hansen, A.; Becker, U. Efficient, approximate and parallel Hartree–Fock and hybrid DFT calculations. A 'chain-of-spheres' algorithm for the Hartree–Fock exchange. *Chem. Phys.* **2009**, *356* (1), 98-109. DOI: 10.1016/j.chemphys.2008.10.036
(100) Izsák, R.; Neese, F. An overlap fitted chain of spheres exchange method. *J. Chem. Phys.* **2011**, *135* (14), 144105. DOI: 10.1063/1.3646921
(101) Izsák, R.; Neese, F.; Klopper, W. Robust fitting techniques in the chain of spheres approximation to the Fock exchange: The role of the complementary space. *J. Chem. Phys.* **2013**, *139* (9), 094111. DOI: 10.1063/1.4819264
(102) Marenich, A. V.; Cramer, C. J.; Truhlar, D. G. Universal solvation model based on solute electron density and on a continuum model of the solvent defined by the bulk dielectric constant and atomic surface tensions. *J. Phys. Chem. B* **2009**, *113* (18), 6378-6396. DOI: 10.1021/jp810292n
(103) Glendening, E.; Badenhoop, J.; Reed, A.; Carpenter, J.; Bohmann, J.; Morales, C.; Karafiloglou, P.; Landis, C.; Weinhold, F., NBO 7.0, Theoretical Chemistry Institute, University of Wisconsin, Madison. 2018.
(104) Mardirossian, N.; Head-Gordon, M. ωB97M-V: A combinatorially optimized, range-separated hybrid, meta-GGA density functional with VV10 nonlocal correlation. *J. Chem. Phys.* **2016**, *144* (21). DOI: 10.1063/1.4952647
(105) Pollak, P.; Weigend, F. Segmented Contracted Error-Consistent Basis Sets of Double- and Triple-ζ Valence Quality for One- and Two-Component Relativistic All-Electron Calculations. *J. Chem. Theory Comput.* **2017**, *13* (8), 3696-3705. DOI: 10.1021/acs.jctc.7b00593
(106) Li, Z.; Xiao, Y.; Liu, W. On the spin separation of algebraic two-component relativistic Hamiltonians: Molecular properties. *J. Chem. Phys.* **2014**, *141* (5). DOI: 10.1063/1.4891567 (acccessed 10/1/2023).
(107) Liu, W.; Peng, D. Exact two-component Hamiltonians revisited. *J. Chem. Phys.* **2009**, *131* (3). DOI: 10.1063/1.3159445
(108) Li, Z.; Xiao, Y.; Liu, W. On the spin separation of algebraic two-component relativistic Hamiltonians. *J. Chem. Phys.* **2012**, *137* (15). DOI: 10.1063/1.4758987
(109) Kutzelnigg, W.; Liu, W. Quasirelativistic theory equivalent to fully relativistic theory. *J. Chem. Phys.* **2005**, *123* (24). DOI: 10.1063/1.2137315
(110) Neese, F. Efficient and accurate approximations to the molecular spin-orbit coupling operator and their use in molecular g-tensor calculations. *J. Chem. Phys.* **2005**, *122* (3), 034107. DOI: 10.1063/1.1829047
(111) Franzke, Y. J.; Yu, J. M. Hyperfine Coupling Constants in Local Exact Two-Component Theory. *J. Chem. Theory Comput.* **2022**, *18* (1), 323-343. DOI: 10.1021/acs.jctc.1c01027

(112) Adamo, C.; Barone, V. Toward reliable density functional methods without adjustable parameters: The PBE0 model. *J. Chem. Phys.* **1999**, *110* (13), 6158-6170. DOI: 10.1063/1.478522
(113) Zheng, J.; Xu, X.; Truhlar, D. G. Minimally augmented Karlsruhe basis sets. *Theor. Chem. Acc.* **2011**, *128*, 295-305. DOI: 10.1007/s00214-010-0846-z
(114) Weigend, F.; Ahlrichs, R. Balanced basis sets of split valence, triple zeta valence and quadruple zeta valence quality for H to Rn: Design and assessment of accuracy. *Phys. Chem. Chem. Phys.* **2005**, *7* (18), 3297-3305. DOI: 10.1039/B508541A
(115) Rappoport, D.; Furche, F. Property-optimized Gaussian basis sets for molecular response calculations. *J. Chem. Phys.* **2010**, *133* (13). DOI: 10.1063/1.3484283
(116) Gouterman, M. Study of the Effects of Substitution on the Absorption Spectra of Porphin. *The Journal of Chemical Physics* **1959**, *30* (5), 1139-1161. DOI: 10.1063/1.1730148
(117) Ryu, H.; Park, J.; Kim, H. K.; Park, J. Y.; Kim, S.-T.; Baik, M.-H. Pitfalls in Computational Modeling of Chemical Reactions and How To Avoid Them. *Organometallics* **2018**, *37* (19), 3228-3239. DOI: 10.1021/acs.organomet.8b00456
(118) Wang, Z.; Yao, Z.; Lyu, Z.; Xiong, Q.; Wang, B.; Fu, X. Thermodynamic and reactivity studies of a tin corrole–cobalt porphyrin heterobimetallic complex. *Chem. Sci.* **2018**, *9* (22), 4999-5007, 10.1039/C8SC01269E. DOI: 10.1039/C8SC01269E
(119) Mewes, J.-M.; Hansen, A.; Grimme, S. Comment on "The Nature of Chalcogen-Bonding-Type Tellurium–Nitrogen Interactions": Fixing the Description of Finite-Temperature Effects Restores the Agreement Between Experiment and Theory. *Angew Chem Int Ed* **2021**, *60* (24), 13144-13149. DOI: 10.1002/anie.202102679
(120) Wang, Z.; Neese, F. Development of NOTCH, an all-electron, beyond-NDDO semiempirical method: Application to diatomic molecules. *J. Chem. Phys.* **2023**, *158* (18). DOI: 10.1063/5.0141686
(121) Ehlert, S.; Stahn, M.; Spicher, S.; Grimme, S. Robust and Efficient Implicit Solvation Model for Fast Semiempirical Methods. *J. Chem. Theory Comput.* **2021**, *17* (7), 4250-4261. DOI: 10.1021/acs.jctc.1c00471
(122) Sigalov, G.; Fenley, A.; Onufriev, A. Analytical electrostatics for biomolecules: Beyond the generalized Born approximation. *J. Chem. Phys.* **2006**, *124* (12). DOI: 10.1063/1.2177251
(123) Pratt, S. L.; Faltynek, R. A. Hydrosilation catalysis via silylmanganese carbonyl complexes: thermal vs. photochemical activation. *J. Organomet. Chem.* **1983**, *258* (1), C5-C8. DOI: 10.1016/0022-328X(83)89515-1
(124) Wayland, B. B.; Coffin, V. L.; Farnos, M. D. Estimation of the Rh-Rh bond dissociation energy in the (octaethylporphyrinato)rhodium(II) dimer by proton NMR line broadening. *Inorg. Chem.* **1988**, *27* (15), 2745-2747. DOI: 10.1021/ic00288a035
(125) Nielsen, C. D. T.; Burés, J. Visual kinetic analysis. *Chem. Sci.* **2019**, *10* (2), 348-353. DOI: 10.1039/C8SC04698K
(126) Burés, J. Variable Time Normalization Analysis: General Graphical Elucidation of Reaction Orders from Concentration Profiles. *Angew. Chem. Int. Ed.* **2016**, *55* (52), 16084-16087. DOI: 10.1002/anie.201609757
(127) Lente, G.; Fábián, I.; Poë, A. J. A common misconception about the Eyring equation. *New J. Chem.* **2005**, *29* (6), 759-760, 10.1039/B501687H. DOI: 10.1039/B501687H
